# Supplementary material for: Examining the psychosocial impacts of the COVID-19 pandemic: an international cross-sectional study protocol
Source: BMJ Open. 2023 Apr 11;13(4):e067886. doi: 10.1136/bmjopen-2022-067886 (PMC10105919; doi:10.1136/bmjopen-2022-067886)
Supplement: Supplementary data [file bmjopen-2022-067886supp001.pdf]

Contents

English Survey 5
Sociodemographic Information 5
Covid Psychosocial Impacts Scale 9
Personal Impacts 9
Family Impacts 10
Employment 13
Income 15
Relationships 16
Changes to Daily Routines and Behaviours 17
Exposure to Information about Covid-19 18
Personal and Family Stress 19
K-10 21
PCL-5 22
WHO-5 24
PTGI 25
International Study – Iraq (Arabic) 27
Sociodemographic Information 27
Covid Psychosocial Impacts Scale (CPIS) 31
الشخصي التأثير 31
الأسرة تأثير 32
العمل ١٩على-كوفيد جائحة تأثير 35
الدخل 37
العلاقات 38
اليومي الروتين و السلوك في تغيرات 39
١٩-كوفيد يخص فيما معلومات 41
١٩-كوفيد جائحة منذ تغيرات 41
K10 43
PCL-5 44
WHO-5 46
PGTI 47

International Study – Indonesia (Bahasa Indonesia) 49
Sociodemographic Information 49
Covid Psychosocial Impacts Scale 53
Dampak terhadap Pribadi 53
Dampak terhadap Keluarga 54
Pekerjaan 57
Pendapatan 59
Hubungan Keluarga 60
Perubahan dalam kegiatan dan perilaku sehari-hari 61
Paparan terhadap informasi mengenai Covid-19 63
Perubahan sejak Covid-19 63
K-10 65
PCL-5 66
WHO-5 68
PTGI 69
International Study – Malaysia (Malay) 71
Sociodemographic Information 71
Covid Psychosocail Impacts Scale 76
Kesan keatas diri sendiri 76
Kesan kepada keluarga 78
Pekerjaan 81
Pendapatan 83
Perhubungan (bersama insan lain) 84
Perubahan kepada Rutin dan Tingkah Laku Harian 85
Pendedahan kepada maklumat tentang Covid-19 87
Perubahan sejak Covid-19 87
K-10 89
PCL-5 90
WHO-5 92
PTGI 93
International Study – Somalia (Somali) 95
Sociodemographic Information 95

Covid Psychosocial Impacts Scale 99
Saameynta Shaqsiga 99
Sameeynta Qoyska 100
Shaqo 103
Dakhliga 105
Xiriika 106
Isbeddelka ku yimid nolol maalmeedka iyo dabeecadaha 107
Soo bandhigista macluumaadka ku saabsan Covid-19 108
Isbeddelada ku yimid Covid-19 dartiis 109
K-10 111
PCL-5 112
WHO-5 114
PTGI 115
International Study - Turkiye (Turkish) 117
Sociodemographic Information 117
Covid Psychosocial Impacts Scale 121
Kişisel etki 121
Aile etkisi 122
İş 125
Gelir 128
İlişkiler 128
Günlük rutin ve davranışlarda değişiklikler 129
Covid-19 hakkındaki bilgilere maruz kalma 131
Covid-19'dan bu yana değişiklikler 132
K-10 134
PCL-5 135
WHO-5 137
PTGI 138
International Study – Pakistan (Urdu) 140
Sociodemographic Information 140
Covid Psychosocial Impacts Scale 144
اثرات ذاتی 144

اثرات پر والوں خاندان 146
کام..... 148
آمدن..... 151
تعلقات 152
تبدیلیاں میں طرزعمل اور معمولات کے روزمرہ 152
معلومات میں بارے کے19 کوویڈ- 154
تبدیلیاں والی ہونے بعد کے 19کوویڈ- 155
K-10 157
PCL-5 158
WHO-5..... 160
PGTI 161
International Study – Iran (Persian)..... 163
Sociodemographic Information 163
Covid Psychosocial Impacts Scale 167
شخصی تاثیرات 167
خانوادگی تاثیرات 168
کووید و کار..... 171
درآمد 173
روابط..... 174
روزانه هایروبه و رفتارها تغییر..... 175
۱۹-کاوید درباره اطلاعات معرض در گرفتن قرار 177
۱۹-کووید زمان از تغییرات..... 177
K-10 179
PCL-5 180
WHO-5..... 182
PTGI 183

English Survey

Sociodemographic Information

1. Gender

- ◯ Male
- ◯ Female
- ◯ Other
- ◯ Prefer not to say

2. Age (in years) ___________________________________

3. Marital status

- ◯ Single
- ◯ In a relationship
- ◯ Married
- ◯ Separated
- ◯ Divorced
- ◯ Widowed

4. What is your religion?

- ◯ No religion
- ◯ Islam
- ◯ Christianity
- ◯ Hinduism
- ◯ Buddhism
- ◯ Other (please specify) ____

Tanveer S, *et al. BMJ Open* 2023; 13:e067886. doi: 10.1136/bmjopen-2022-067886

5. What is your ethnic background? (You can mention more than one)

____

6. In your daily life, which languages do you speak fluently?

____

The following two questions ask specifically about your English language ability. 6a. I can understand English well

- Strongly disagree 1
- Disagree 2
- Neither agree nor disagree 3
- Agree 4
- Strongly agree 5

6b. I speak English well

- Not at all 1
- A little 2
- Somewhat 3
- Quite a lot 4
- A lot 5

7. What is your highest level of education?

- No formal qualification (attended school, ESOL class)
- Secondary school (e.g. NCEA, IB diploma, overseas school qualification)
- Tertiary qualification (e.g. Certificate, Diploma or Trade qualification - less than 3 year course)
- Bachelor degree (3-4 year course)
- Post graduate degree (PG diploma, Master, Doctorate)

8. Current study or work status (Please tick whichever ones apply)

- ☐ Full-time student
- ☐ Part-time student
- ☐ Unemployed / Seeking work
- ☐ Full-time paid work
- ☐ Part-time paid work
- ☐ Full-time self employed
- ☐ Part-time self employed
- ☐ On employer paid parental leave
- ☐ Stay-at-home parent (unpaid)
- ☐ Retired
- ☐ Other (Please specify) ____

9. Occupation

____

10. Monthly household income (in local currency)

____

10a. Compared with the average income, how would you rate the income of your household?

- ○ Below average
- ○ Average
- ○ Above average

11. Are you currently in lockdown or stay-at-home order?

- Yes
- No

12. Have you had a Covid-19 vaccine?

- Yes
- No

12a. If not vaccinated, would you like to get the Covid-19 vaccine in the future?

- Yes
- No

13. We realise that some people may have been exposed to other traumatic incidents in the past which could make it easier or more difficult to deal with new problems.

Before the Covid-19 pandemic, have you ever witnessed or experienced any of the following: (Please tick as many as appropriate)

- [ ] Natural disaster (such as a flood, earthquake etc.)
- [ ] Living in a war zone or being exposed to military conflict
- [ ] Childhood adversity before the age of 16 (such as neglect, bullying, physical or sexual assault)
- [ ] Physical or sexual assault after the age of 16
- [ ] Serious physical accident
- [ ] Other – please specify (optional) ____
- [ ] None of the above

Covid Psychosocial Impacts Scale

Personal Impacts

For the following statements, please respond with Yes / No if this applies to you in response to Covid-19. If Yes, please indicate the amount of stress that this caused you on the scale below.

1. Do you have an underlying health condition that could make you vulnerable to Covid-19?

- ○ No 0
- ○ Yes No stress at all 1
- ○ Yes A little stress 2
- ○ Yes Moderately stressed 3
- ○ Yes Quite a lot of stress 4
- ○ Yes A lot of stress 5

2. Have you felt at risk of being exposed to someone with Covid-19?

- ○ No 0
- ○ Yes No stress at all 1
- ○ Yes A little stress 2
- ○ Yes Moderately stressed 3
- ○ Yes Quite a lot of stress 4
- ○ Yes A lot of stress 5

3. Do you think you have had Covid-19?

- ○ No 0
- ○ Yes No stress at all 1
- ○ Yes A little stress 2
- ○ Yes Moderately stressed 3

- **Yes** Quite a lot of stress 4
- **Yes** A lot of stress 5

3a. Have you had a positive Covid-19 test?

- **No** 0
- **Yes** No stress at all 1
- **Yes** A little stress 2
- **Yes** Moderately stressed 3
- **Yes** Quite a lot of stress 4
- **Yes** A lot of stress 5

3b. Do you have ongoing symptoms of Covid-19?

- **No** 0
- **Yes** No stress at all 1
- **Yes** A little stress 2
- **Yes** Moderately stressed 3
- **Yes** Quite a lot of stress 4
- **Yes** A lot of stress 5

Family Impacts

For the following statements, please respond with Yes / No if this applies to your close family members in response to Covid-19. If Yes, please indicate the amount of stress that this caused you on the scale below.

1. Does any close family member(s) have an underlying health condition that could make them vulnerable to Covid-19?

- **No** 0
- **Yes** No stress at all 1

Tanveer S, *et al. BMJ Open* 2023; 13:e067886. doi: 10.1136/bmjopen-2022-067886

- **Yes** A little stress 2
- **Yes** Moderately stressed 3
- **Yes** Quite a lot of stress 4
- **Yes** A lot of stress 5

2. Do you feel any close family member(s) is at risk of being exposed to someone with Covid-19?

- **No** 0
- **Yes** No stress at all 1
- **Yes** A little stress 2
- **Yes** Moderately stressed 3
- **Yes** Quite a lot of stress 4
- **Yes** A lot of stress 5

3. Do you think any close family member(s) have had Covid-19?

- **No** 0
- **Yes** No stress at all 1
- **Yes** A little stress 2
- **Yes** Moderately stressed 3
- **Yes** Quite a lot of stress 4
- **Yes** A lot of stress 5

3a. Has any close family member(s) tested positive for Covid-19?

- **No** 0
- **Yes** No stress at all 1
- **Yes** A little stress 2

- **Yes** Moderately stressed 3
- **Yes** Quite a lot of stress 4
- **Yes** A lot of stress 5

3b. Has any close family member(s) have ongoing symptoms of Covid-19?

- **No** 0
- **Yes** No stress at all 1
- **Yes** A little stress 2
- **Yes** Moderately stressed 3
- **Yes** Quite a lot of stress 4
- **Yes** A lot of stress 5

3c. Has any close family member(s) passed away from Covid-19?

- **No** 0
- **Yes** No stress at all 1
- **Yes** A little stress 2
- **Yes** Moderately stressed 3
- **Yes** Quite a lot of stress 4
- **Yes** A lot of stress 5

4. Do you have any close family member(s) overseas who you are worried are at risk of getting Covid-19?

- **No** 0
- **Yes** No stress at all 1
- **Yes** A little stress 2
- **Yes** Moderately stressed 3

- **Yes** Quite a lot of stress 4
- **Yes** A lot of stress 5

5. Do you have any close family member(s) overseas who you are not able to visit or who cannot visit you?

- **No** 0
- **Yes** No stress at all 1
- **Yes** A little stress 2
- **Yes** Moderately stressed 3
- **Yes** Quite a lot of stress 4
- **Yes** A lot of stress 5

Employment

For the following statements please respond with Yes / No / Not applicable if this applies to you or anyone in your household in response to Covid-19. If Yes, please indicate the amount of stress that this caused you on the scale below.

1. Are you or anyone in your household considered an essential worker (e.g., in healthcare, law enforcement, emergency services, provider of essential goods/services)?

- **No** 0
- **Yes** No stress at all 1
- **Yes** A little stress 2
- **Yes** Moderately stressed 3
- **Yes** Quite a lot stress 4
- **Yes** A lot of stress 5

2. Have you or anyone in your household had a major change in working hours?

- **No** 0
- **Yes** No stress at all 1

Tanveer S, *et al. BMJ Open* 2023; 13:e067886. doi: 10.1136/bmjopen-2022-067886

- **Yes** A little stress 2
- **Yes** Moderately stressed 3
- **Yes** Quite a lot stress 4
- **Yes** A lot of stress 5

3. Have you or anyone in your household had to change the place of work to home?

- **No** 0
- **Yes** No stress at all 1
- **Yes** A little stress 2
- **Yes** Moderately stressed 3
- **Yes** Quite a lot stress 4
- **Yes** A lot of stress 5

4. Have you or anyone in your household had to change to a different type of work?

- **No** 0
- **Yes** No stress at all 1
- **Yes** A little stress 2
- **Yes** Moderately stressed 3
- **Yes** Quite a lot stress 4
- **Yes** A lot of stress 5

5. Have you or anyone in your household lost their job?

- **Not applicable** 9
- **No** 0
- **Yes** No stress at all 1

- **Yes** A little stress 2
- **Yes** Moderately stressed 3
- **Yes** Quite a lot stress 4
- **Yes** A lot of stress 5

6. Is a business you or anyone in your household owns or works in under threat of survival?

- **Not applicable** 9
- **No** 0
- **Yes** No stress at all 1
- **Yes** A little stress 2
- **Yes** Moderately stressed 3
- **Yes** Quite a lot stress 4
- **Yes** A lot of stress 5

Income

For the following statements please respond with Yes / No if this applies to your household in response to Covid-19. If Yes, please indicate the amount of stress that this caused you on the scale below.
1. Has your household had a major deterioration in financial circumstances?

- **No** 0
- **Yes** No stress at all 1
- **Yes** A little stress 2
- **Yes** Moderately stressed 3
- **Yes** Quite a lot of stress 4
- **Yes** A lot of stress 5

2. Has your household had a major deterioration in your ability to pay your bills or for food?

- ○ **No** 0
- ○ **Yes** No stress at all 1
- ○ **Yes** A little stress 2
- ○ **Yes** Moderately stressed 3
- ○ **Yes** Quite a lot of stress 4
- ○ **Yes** A lot of stress 5

Relationships

For the following statements please respond with Yes / No if this applies to you in response to Covid-19. If Yes, please indicate the amount of stress that this caused you on the scale below. In response to Covid-19…..

1. Has it increased difficulty and tension in the family?

- ○ **No** 0
- ○ **Yes** No stress at all 1
- ○ **Yes** A little stress 2
- ○ **Yes** Moderately stressed 3
- ○ **Yes** Quite a lot of stress 4
- ○ **Yes** A lot of stress 5

2. Has this decreased your contact with friends (physical or online)?

- ○ **No** 0
- ○ **Yes** No stress at all 1
- ○ **Yes** A little stress 2
- ○ **Yes** Moderately stressed 3
- ○ **Yes** Quite a lot of stress 4

- **Yes** A lot of stress 5

Changes to Daily Routines and Behaviours

For the following statements please respond with Yes / No / Not applicable, if this applies to you in response to Covid-19. If Yes, please indicate the amount of stress that this caused you on the scale below. In response to Covid-19.....

1. Has the Covid pandemic led to changes in your daily routine (for example, sleep, exercise, free time, pleasurable activities, or hobbies)?

- **No** 0
- **Yes** No stress at all 1
- **Yes** A little stress 2
- **Yes** Moderately stressed 3
- **Yes** Quite a lot of stress 4
- **Yes** A lot of stress 5

2. Has this caused you to delay / avoid seeking health care when you or a close family member needed it?

- **No** 0
- **Yes** No stress at all 1
- **Yes** A little stress 2
- **Yes** Moderately stressed 3
- **Yes** Quite a lot of stress 4
- **Yes** A lot of stress 5

3. Has this decreased your involvement with religious activities (in person or on-line)?

- **Not applicable** 9
- **No** 0
- **Yes** No stress at all 1

Tanveer S, et al. BMJ Open 2023; 13:e067886. doi: 10.1136/bmjopen-2022-067886

- ○ **Yes** A little stress 2
- ○ **Yes** Moderately stressed 3
- ○ **Yes** Quite a lot of stress 4
- ○ **Yes** A lot of stress 5

4. Has there been any other major change in your daily routines.

- ○ **No** 0
- ○ **Yes (Please specify)** 1 ____

If Yes, please indicate the amount of stress that this caused you on the scale below.

- ○ No stress at all 1
- ○ A little stress 2
- ○ Moderately stressed 3
- ○ Quite a lot of stress 4
- ○ A lot of stress 5

Exposure to Information about Covid-19

1. In regards to Covid-19, what is the source of information for you? (Please tick as many as appropriate.)

- ☐ News
- ☐ Facebook / Twitter
- ☐ Health websites
- ☐ Word of mouth
- ☐ Others (please specify) ____

2. How much stress is the news about Covid-19 causing you?

- No stress at all 1
- A little stress 2
- Moderately stressed 3
- Quite a lot of stress 4
- A lot of stress 5

Personal and Family Stress

For the following statements, please indicate on the given scale. Comparing now with before the Covid-19 pandemic...

1. How would you rate your personal level of stress?

- Considerably less stressed 1
- Somewhat less stressed 2
- About the same 3
- Somewhat more stressed 4
- Considerably more stressed 5

2. How would you rate the overall stress in your household?

- Considerably less stressed 1
- Somewhat less stressed 2
- About the same 3
- Somewhat more stressed 4
- Considerably more stressed 5

Tanveer S, *et al. BMJ Open* 2023; 13:e067886. doi: 10.1136/bmjopen-2022-067886

3. How would you rate your personal psychological wellbeing?

- Much better 1
- Better 2
- About the same 3
- Worse 4
- Much worse 5

4. How would you rate the overall psychological wellbeing of your household?

- Much better 1
- Better 2
- About the same 3
- Worse 4
- Much worse 5

K-10

The following ten questions ask about how you have been feeling in the last 4 weeks. For each question, select the option that best describes the amount of time you felt that way.

	None of the time 1	A little of the time 2	Some of the time 3	Most of the time 4	All of the time 5
1. In the last four weeks, about how often did you feel tired out for no good reason?	○	○	○	○	○
2. In the last four weeks, about how often did you feel nervous?	○	○	○	○	○
3. In the last four weeks, about how often did you feel so nervous that nothing could calm you down?	○	○	○	○	○
4. In the last four weeks, about how often did you feel hopeless?	○	○	○	○	○
5. In the last four weeks, about how often did you feel restless or fidgety?	○	○	○	○	○
6. In the last four weeks, about how often did you feel so restless you could not sit still?	○	○	○	○	○
7. In the last four weeks, about how often did you feel depressed?	○	○	○	○	○
8. In the last four weeks, about how often did you feel that everything was an effort?	○	○	○	○	○
9. In the last four weeks, about how often did you feel so sad that nothing could cheer you up?	○	○	○	○	○
10. In the last four weeks, about how often did you feel worthless?	○	○	○	○	○

Tanveer S, *et al. BMJ Open* 2023; 13:e067886. doi: 10.1136/bmjopen-2022-067886

PCL-5

Below is a list of problems which some people have experienced in response to Covid-19. Please read each problem carefully and then indicate how much you have been bothered by that problem in the past month. In the past month, how much were you bothered by:

	Not at all 0	A little bit 1	Moderately 2	Quite a bit 3	Extremely 4
1. Repeated, disturbing, and unwanted memories of the stressful experience?	○	○	○	○	○
2. Repeated, disturbing dreams of the stressful experience?	○	○	○	○	○
3. Suddenly feeling or acting as if the stressful experience were actually happening again (as if you were actually back there reliving it)?	○	○	○	○	○
4. Feeling very upset when something reminded you of the stressful experience?	○	○	○	○	○
5. Having strong physical reactions when something reminded you of the stressful experience (for example, heart pounding, trouble breathing, sweating)?	○	○	○	○	○
6. Avoiding memories, thoughts, or feelings related to the stressful experience?	○	○	○	○	○
7. Avoiding external reminders of the stressful experience (for example, people, places, conversations, activities, objects, or situations)?	○	○	○	○	○
8. Trouble remembering important parts of the stressful experience?	○	○	○	○	○
9. Having strong negative beliefs about yourself, other people, or the world (for example, having thoughts such as: I am bad, there is something seriously wrong with me, no one can be trusted, the world is completely dangerous)?	○	○	○	○	○
10. Blaming yourself or someone else for the stressful experience or what happened after it?	○	○	○	○	○
11. Having strong negative feelings such as fear, horror, anger, guilt, or shame?	○	○	○	○	○

12. Loss of interest in activities that you used to enjoy?	◯	◯	◯	◯	◯
13. Feeling distant or cut off from other people?	◯	◯	◯	◯	◯
14. Trouble experiencing positive feelings (for example, being unable to feel happiness or have loving feelings for people close to you)?	◯	◯	◯	◯	◯
15. Irritable behaviour, angry outbursts, or acting aggressively?	◯	◯	◯	◯	◯
16. Taking too many risks or doing things that could cause you harm?	◯	◯	◯	◯	◯
17. Being "super alert" or watchful or on guard?	◯	◯	◯	◯	◯
18. Feeling jumpy or easily startled?	◯	◯	◯	◯	◯
19. Having difficulty concentrating?	◯	◯	◯	◯	◯
20. Trouble falling or staying asleep?	◯	◯	◯	◯	◯

WHO-5

Please indicate for each of the five statements which is closest to how you have been feeling over the last 2 weeks.

	All of the time 5	Most of the time 4	More than half of the time 3	Less than half of the time 2	Some of the time 1	At no time 0
1. I have felt cheerful and in good spirits	○	○	○	○	○	○
2. I have felt calm and relaxed	○	○	○	○	○	○
3. I have felt active and vigorous	○	○	○	○	○	○
4. I woke up feeling fresh and rested	○	○	○	○	○	○
5. My daily life has been filled with things that interest me	○	○	○	○	○	○

Tanveer S, *et al. BMJ Open* 2023; 13:e067886. doi: 10.1136/bmjopen-2022-067886

PTGI

Indicate for each of the statements below the degree to which this change occurred in your life as a result of Covid-19, using the following scale.

	Not at all 0	A little bit 1	Somewha t 2	Moderate ly 3	Quite a bit 4	A lot 5
1. I changed my priorities about what is important in life.	○	○	○	○	○	○
2. I have a greater appreciation for the value of my own life.	○	○	○	○	○	○
3. I developed new interests.	○	○	○	○	○	○
4. I have a greater feeling of self-reliance.	○	○	○	○	○	○
5. I have a better understanding of spiritual matters.	○	○	○	○	○	○
6. I more clearly see that I can count on people in times of trouble.	○	○	○	○	○	○
7. I established a new path for my life.	○	○	○	○	○	○
8. I have a great sense of closeness with others.	○	○	○	○	○	○
9. I am more willing to express my emotions.	○	○	○	○	○	○
10. I know better that I can handle difficulties.	○	○	○	○	○	○
11. I am able to do better things with my life.	○	○	○	○	○	○
12. I am better able to accept the way things work out.	○	○	○	○	○	○
13. I can better appreciate each day.	○	○	○	○	○	○
14. New opportunities are available which wouldn't have been otherwise.	○	○	○	○	○	○

Tanveer S, *et al. BMJ Open* 2023; 13:e067886. doi: 10.1136/bmjopen-2022-067886

15. I have more compassion for others.	○	○	○	○	○	○
16. I put more effort into my relationships.	○	○	○	○	○	○
17. I am more likely to try to change things which need changing.	○	○	○	○	○	○
18. I have a stronger religious faith.	○	○	○	○	○	○
19. I discovered that I'm stronger than I thought I was.	○	○	○	○	○	○
20. I learned a great deal about how wonderful people are.	○	○	○	○	○	○
21. I better accept needing others.	○	○	○	○	○	○

International Study – Iraq (Arabic)

Sociodemographic Information

١- الجنس

- ◯ ذكر
- ◯ أنثى
- ◯ اخرى
- ◯ أُفضل عدم الإجابة

٢- بالسنوات (الفئة العمرية)

____

٣- الحالة الإجتماعية

- ◯ أعزب
- ◯ مرتبط
- ◯ متزوج
- ◯ منفصل
- ◯ مطلق
- ◯ أرمل

٤- ماهي ديانتك؟

- ◯ ليس لدي ديانة
- ◯ الإسلام
- ◯ المسيحية
- ◯ الهندوسية
- ◯ البوذية

- ○ أخرى (برجاء الشرح) ____

٥- ما هي أُصولك العرقية؟ (يمكن أن تذكر أكثر من واحدة)

____

٦-ماهي اللغات التي تتحدثها بطلاقة في حياتك اليومية؟

____

السؤالان التاليان يحددان مدى قدرتك في اللغة الإنجليزية.
٦أ- يمكنني فهم اللغة الإنجليزية جيداً

- ○ لا أوافق بشدة 1
- ○ لا أوافق 2
- ○ لا اعلم 3
- ○ أوافق 4
- ○ أوافق بشدة 5

٦ب- يمكنني تحدث الإنجليزية جيداً

- ○ لا على الإطلاق 1
- ○ قليلاً 2
- ○ بقدر معتدل 3
- ○ كثيراً 4
- ○ كثيراً جداً 5

٧- ما هي مؤهلاتك العلمية؟

- ○ لا يوجد مؤهل علمي (التحقت بالمدرسة، فصل تعليم الكبار غير الناطقين بالإنجليزية)
- ○ المرحلة الثانوية (على سبيل المثال، الشهادة الوطنية النيوزيلاندية للتحصيل التعليمي، دبلوم، والمؤهلات المدرسية من خارج نيوزيلاندا)

Tanveer S, *et al. BMJ Open* 2023; 13:e067886. doi: 10.1136/bmjopen-2022-067886

○ مؤهل جامعي (على سبيل المثال، شهادة، دبلوم أو مؤهل تجاري -دورة أقل من 3 سنوات)

○ البكالوريوس (دورة 4-3 سنوات)

○ الدراسات العليا (دبلوم الدراسات العليا، الماجستير، الدكتوراة)

٨- وضعك الدراسي أو الوظيفي (يرجى وضع علامة على كل الحالات التي تنطبق عليك)

- ☐ يدرس بدوام كامل
- ☐ يدرس بدوام جزئي
- ☐ لا يعمل / يبحث عن عمل
- ☐ يعمل بدوام كامل (وظيفة واحدة او أكثر)
- ☐ يعمل بدوام جزئي (وظيفة واحدة او أكثر)
- ☐ يعمل لحسابة بدوام كامل
- ☐ يعمل لحسابة بدوام جزئي
- ☐ أجازة امومة مدفوعة الأجر
- ☐ رب - ربة منزل (غير مدفوع الأجر)
- ☐ متقاعد
- ☐ أخرى (برجاء الشرح) ____________________

٩- الوظيفة

____

١٠- دخل الأسرة الشهري (بالعملة المحلية)

____

١٠أ- مقارنة بمتوسط الدخل، ما هو تقيمك لدخل أسرتك؟

○ أقل من المتوسط

○ متوسط

○ أعلى من المتوسط

١١- هل أنت حالياً في وضع العزل بسبب جائحة كوفيد-١٩؟

○ نعم

○ لا

١٢- هل تلقيت مطعوم كوفيد-١٩؟

- ○ نعم
- ○ لا

١٢أ- إذا لم تتلقى مطعوم كوفيد-١٩، هل ترغب في الحصول عليه في المستقبل؟

- ○ نعم
- ○ لا

١٣- ندرك أن بعض الأشخاص قد تعرضوا إلى أحداث صادمة في الماضي قد تجعل التعامل مع المشكلات الأخرى أسهل أو أصعب في التعامل مع اي مشكلات جديدة. قبل جائحة كوفيد-١٩ هل سبق أن شاهدت أو تعرضت إلى واحدة أو أكثر من هذه المواقف :(يرجى وضع علامة أو اكثر إن وجد)

- ☐ كوارث طبيعية (مثل فيضان، زلزال أو ما إلى ذلك)
- ☐ كنت تعيش في منطقة حروب أو تعرضت إلى نزاع عسكري
- ☐ محنة في الطفولة قبل سن ١٦ عاماً (مثل الإهمال، التنمرأو الإعتداء الجنسي)
- ☐ اعتداء بدني أو جنسي بعد سن ١٦عاماً
- ☐ حادث بدني خطير
- ☐ أخرى - برجاء الشرح (أختياري) ____
- ☐ ليس مما سبق

Covid Psychosocial Impacts Scale (CPIS)

التأثير الشخصي

يرجى الإجابة (بنعم / لا) إذا كان لجائحة كوفيد-١٩ أثرعليك من خلال الإسئلة التالية. إذا كانت اجابتك بنعم، يرجى تحديد مقدار الضغط الذي تشعر به من خلال هذا المقياس أدناه.

١- هل لديك اي مشاكل صحية تجعلك عرضة لمرض كوفيد-١٩؟

- لا 0
- نعم لا يوجد ضغط اطلاقاً 1
- نعم قليلاً 2
- نعم احيانا 3
- نعم غالباً جداً 4
- نعم كثيراً جداً 5

٢- هل شعرت بخطر التعرض لشخص مصاب بكوفيد-١٩؟

- لا 0
- نعم لا يوجد ضغط اطلاقاً 1
- نعم قليلاً 2
- نعم احيانا 3
- نعم غالباً جداً 4
- نعم كثيراً جداً 5

٣- هل أُصبت بمرض كوفيد (كورونا)؟

- لا 0
- نعم لا يوجد ضغط اطلاقاً 1
- نعم قليلاً 2
- نعم احيانا 3

نعم غالباً جداً 4

نعم كثيراً جداً 5

٣أ- هل كانت نتيجة فحص أختبار كوفيد-١٩ إيجابية؟

لا 0

نعم لا يوجد ضغط اطلاقاً 1

نعم قليلاً 2

نعم احيانا 3

نعم غالباً جداً 4

نعم كثيراً جداً 5

٣ب -هل تعاني من أعراض مستمرة بسبب كوفيد-١٩؟

لا 0

نعم لا يوجد ضغط اطلاقاً 1

نعم قليلاً 2

نعم احيانا 3

نعم غالباً جداً 4

نعم كثيراً جداً 5

تأثير الأسرة

يرجى الإجابة (بنعم / لا) إذا كان لجائحة كوفيد-١٩ أثرعلى أسرتك من خلال الإسئلة التالية. إذا كانت اجابتك بنعم، يرجى تحديد مقدار الضغط الذي تشعر به من خلال هذا المقياس أدناه.

١- هل يعاني احد أفراد أسرتك من اي مشاكل صحية تجعله عرضة لمرض كوفيد-١٩؟

لا 0

نعم لا يوجد ضغط اطلاقاً 1

- نعم قليلاً 2
- نعم احيانا 3
- نعم غالباً جداً 4
- نعم كثيراً جداً 5

٢- هل تشعر ان احد أفراد أسرتك معرض للاصابة لكوفيد-١٩ من خلال الإختلاط بأحد أفراد العمل؟

- لا 0
- نعم لا يوجد ضغط اطلاقاً 1
- نعم قليلاً 2
- نعم احيانا 3
- نعم غالباً جداً 4
- نعم كثيراً جداً 5

٣- هل أُصيب احد أفراد أسرتك بمرض كوفيد-١٩؟

- لا 0
- نعم لا يوجد ضغط اطلاقاً 1
- نعم قليلاً 2
- نعم احيانا 3
- نعم غالباً جداً 4
- نعم كثيراً جداً 5

٣أ- هل كانت نتيجة فحص كوفيد-١٩ إيجابية لأحد أفراد أسرتك؟

- لا 0
- نعم لا يوجد ضغط اطلاقاً 1

- نعم قليلاً 2
- نعم احيانا 3
- نعم غالباً جداً 4
- نعم كثيراً جداً 5

٣ب ـ هل أحد أفراد أسرتك مازال يعاني من أعراض كوفيد-١٩؟

- لا 0
- نعم لا يوجد ضغط اطلاقاً 1
- نعم قليلاً 2
- نعم احيانا 3
- نعم غالباً جداً 4
- نعم كثيراً جداً 5

٣ج - هل توفى احد أفراد أسرتك كنتيجة لكوفيد-١٩؟

- لا 0
- نعم لا يوجد ضغط اطلاقاً 1
- نعم قليلاً 2
- نعم احيانا 3
- نعم غالباً جداً 4
- نعم كثيراً جداً 5

٤- هل لديك احد من أفراد أسرتك مقيم بالخارج و تشعر بالقلق عليهم من خطر الإصابة بمرض كوفيد-١٩؟

- لا 0

Tanveer S, et al. BMJ Open 2023; 13:e067886. doi: 10.1136/bmjopen-2022-067886

- نعم لا يوجد ضغط اطلاقاً 1
- نعم قليلاً 2
- نعم احيانا 3
- نعم غالباً جداً 4
- نعم كثيراً جداً 5

٥ـ هل لديك احد من أفراد أسرتك يقيم بالخارج و لا يمكنك زيارتهم و لا يمكنهم زيارتك؟

- لا 0
- نعم لا يوجد ضغط اطلاقاً 1
- نعم قليلاً 2
- نعم احيانا 3
- نعم غالباً جداً 4
- نعم كثيراً جداً 5

تأثير جائحة كوفيد-١٩ على العمل

يرجى الإجابة (بنعم / لا / لا أُفضل الإجابة) إذا كان لجائحة كوفيد-١٩ أثرعليك او على احد أفراد أسرتك المقيمين معك بالمنزل من خلال الإسئلة التالية. إذا كانت اجابتك بنعم، يرجى تحديد مقدار الضغط الذي تشعر به من خلال هذا المقياس أدناه.

١ـ هل تعتبر أنت أو احد أفراد أسرتك المقيمين معك بالمنزل من العمال الأساسيين (مثل عمال الرعاية الصحية، شؤون القانون، خدمات الطوارئ أو مزودي السلع و الخدمات الأساسية)؟

- لا 0
- نعم لا يوجد ضغط اطلاقاً 1
- نعم قليلاً 2
- نعم احيانا 3
- نعم غالباً جداً 4

Page 35 of 184

- ○ نعم كثيراً جداً 5

٢- هل كان هناك تغير كبير في ساعات العمل لك أو احد أفراد أسرتك المقيمين معك بالمنزل؟

- ○ لا 0
- ○ نعم لا يوجد ضغط اطلاقاً 1
- ○ نعم قليلاً 2
- ○ نعم احيانا 3
- ○ نعم غالباً جداً 4
- ○ نعم كثيراً جداً 5

٣- هل اضطررت أنت أو احد أفراد أسرتك المقيمين معك بالمنزل إلى تغيير مكان العمل للعمل من المنزل؟

- ○ لا 0
- ○ نعم لا يوجد ضغط اطلاقاً 1
- ○ نعم قليلاً 2
- ○ نعم احيانا 3
- ○ نعم غالباً جداً 4
- ○ نعم كثيراً جداً 5

٤- هل اضطررت أنت أو احد أفراد أسرتك المقيمين معك بالمنزل إلى تغيير طبيعة العمل؟

- ○ لا 0
- ○ نعم لا يوجد ضغط اطلاقاً 1
- ○ نعم قليلاً 2
- ○ نعم احيانا 3
- ○ نعم غالباً جداً 4

○ نعم كثيراً جداً 5

٥ -هل فقدت أنت او احد أفراد أسرتك المقيمين معك بالمنزل؟

○ لا أُفضل الإجابة

○ لا 0

○ نعم لا يوجد ضغط اطلاقاً 1

○ نعم قليلاً 2

○ نعم احيانا 3

○ نعم غالباً جداً 4

○ نعم كثيراً جداً 5

٦- هل الأعمال التجارية التي تمتلكها او تعمل بها أنت او احد أفراد أسرتك المقيمين معك بالمنزل أصبحت مهددة بالبقاء؟

○ لا أُفضل الإجابة

○ لا 0

○ نعم لا يوجد ضغط اطلاقاً 1

○ نعم قليلاً 2

○ نعم احيانا 3

○ نعم غالباً جداً 4

○ نعم كثيراً جداً 5

الدخل

يرجى الإجابة (بنعم / لا) إذا حدث ذلك لاحد أفراد أسرتك المقيمين معك بالمنزل كنتيجة لجائحة كوفيد-١٩ من خلال الإسئلة التالية. إذا كانت اجابتك بنعم ، فيرجى تحديد مقدار الضغط الذي تشعر به من خلال هذا المقياس أدناه.

١- هل تعرض أحد من أفراد أسرتك لتدهور كبير في الحالة المالية؟

○ لا 0

نعم لا يوجد ضغط اطلاقاً 1

نعم قليلاً 2

نعم احيانا 3

نعم غالباً جداً 4

نعم كثيراً جداً 5

٢- هل تعرض احد أفراد أسرتك المقيمين لتدهور كبير في عدم القدرة على دفع الفواتير او توفير الإحتياجات الغذائية؟

لا 0

نعم لا يوجد ضغط اطلاقاً 1

نعم قليلاً 2

نعم احيانا 3

نعم غالباً جداً 4

نعم كثيراً جداً 5

العلاقات

يرجى الإجابة (بنعم / لا) إذا كان لجائحة كوفيد-١٩ أثرعليك من خلال الإسئلة التالية.
إذا كانت اجابتك بنعم، يرجى تحديد مقدار الضغط الذي تشعر به من خلال هذا المقياس أدناه. كنتيجة لكوفيد-١٩....
١- هل ادى ذلك لزيادة المشاكل والتوتر داخل العائلة؟

لا 0

نعم لا يوجد ضغط اطلاقاً 1

نعم قليلاً 2

نعم احيانا 3

نعم غالباً جداً 4

نعم كثيراً جداً 5

٢- هل ادى ذلك الى انخفاض معدل تواصلك مع أصدقائك سواء تواصل اجتماعياً او من خلال التواصل الإليكتروني؟

- لا 0
- نعم لا يوجد ضغط اطلاقاً 1
- نعم قليلاً 2
- نعم احيانا 3
- نعم غالباً جداً 4
- نعم كثيراً جداً 5

تغيرات في السلوك و الروتين اليومي

يرجى الإجابة (نعم / لا / لا أُفضل الإجابة) إذا كان لجائحة كوفيد-١٩ أثرعليك من خلال الإسئلة التالية. إذا كانت اجابتك بنعم، يرجى تحديد مقدار الضغط الذي تشعر به من خلال هذا المقياس أدناه.

١- هل أدت جائحة كوفيد إلى تغير معدل أنشطتك اليومية (على سبيل المثال :معدل النوم، الأنشطة الرياضية، وقت الفراغ أو استمتاعك بالأنشطة أو الهوايات الخاص بك)؟

- لا 0
- نعم لا يوجد ضغط اطلاقاً 1
- نعم قليلاً 2
- نعم احيانا 3
- نعم غالباً جداً 4
- نعم كثيراً جداً 5

٢- هل ادى ذلك الى تأخير او تجنب طلب الرعاية الصحية عندما تحتاجها أنت أو أحد أفراد أسرتك؟

- لا 0
- نعم لا يوجد ضغط اطلاقاً 1
- نعم قليلاً 2
- نعم احيانا 3

- ○ نعم غالباً جداً 4
- ○ نعم كثيراً جداً 5

٣- هل ادى ذلك الى انخفاض معدل مشاركتك في الأنشطة الدينية (مثل سواء وجهاً لوجه أو عبر الإنترنت)؟

- ○ لا أُفضل الإجابة
- ○ لا 0
- ○ نعم لا يوجد ضغط اطلاقاً 1
- ○ نعم قليلاً 2
- ○ نعم احيانا 3
- ○ نعم غالباً جداً 4
- ○ نعم كثيراً جداً 5

٤- هل لديك اى تغيرات كبيرة اخرى في حياتك الروتينية؟

- ○ لا 0
- ○ نعم (برجاء الشرح) 1 ____________________________

إذا كانت اجابتك بنعم، يرجى تحديد مقدار الضغط الذي تشعر به من خلال هذا المقياس أدناه.

- ○ لا يوجد ضغط اطلاقاً 1
- ○ قليلاً 2
- ○ احيانا 3
- ○ غالباً جداً 4
- ○ كثيراً جداً 5

Tanveer S, *et al. BMJ Open* 2023; 13:e067886. doi: 10.1136/bmjopen-2022-067886

معلومات فيما يخص كوفيد-١٩

١- فيما يخص كوفيد-١٩ ماهو مصدر المعلومات بالنسبة لك؟ (برجاء وضع علامة على أكثر من إختيار.)

- ☐ الأخبار
- ☐ الفيس بوك / تويتر
- ☐ المواقع الصحية المعتمدة
- ☐ كلام شَفهي
- ☐ أخرى (برجاء الشرح) ________________________________

٢- ما مدى الضغط النفسي الذي تسببته الأخبار عن كوفيد-١٩؟

- ○ نعم لا يوجد ضغط اطلاقاً 1
- ○ نعم قليلاً 2
- ○ نعم احيانا 3
- ○ نعم غالباً جداً 4
- ○ نعم كثيراً جداً 5

تغيرات منذ جائحة كوفيد-١٩

من خلال الأسئلة التالية، يرجى الإشارة إلى مقدار الضغط النفسي الذي تأثرت به. مقارنة بين الوضع الحالي و الوضع قبل جائحة كوفيد-١٩....

١- كيف تقيم مستوى ضغطك الشخصي؟

- ○ ضغط قليل جداً 1
- ○ ضغط الى حداً ما 2
- ○ بقى كما هو 3
- ○ ضغط أكثر 4
- ○ ضغط كثير جداً 5

٢- كيف تقيم مستوى الضغط العام في منزلك؟

- ○ ضغط قليل جداً 1
- ○ ضغط الى حداً ما 2

Tanveer S, *et al. BMJ Open* 2023; 13:e067886. doi: 10.1136/bmjopen-2022-067886

- ○ بقى كما هو 3
- ○ ضغط أكثر 4
- ○ ضغط كثير جداً 5

٣- كيف تقيم صحتك النفسية الشخصية؟

- ○ أفضل بكثير 1
- ○ أفضل 2
- ○ بقى كما هو 3
- ○ أسوء 4
- ○ أسوء بكثير 5

٤- كيف تُقيم مقدار الصحة النفسية لأفراد أسرتك بشكل عام؟

- ○ أفضل بكثير 1
- ○ أفضل 2
- ○ بقى كما هو 3
- ○ أسوء 4
- ○ أسوء بكثير 5

K10

الأسئلة العشر التالية سوف يتم سؤالك عن ما كنت تشعر به خلال الأربع أسابيع الماضية. يرجى قراءة الأسئلة التالية و اختيار الإجابة الأقرب التي تمثل وضعك الحالي.

	إطلاقاً 1	قليلاً 2	أحياناً 3	غالباً 4	دائماً 5
١- خلال الأربع أسابيع الماضية، إلى أي مدى كنت تشعر أنك مجهد بدون أي سبب واضح؟	○	○	○	○	○
٢- خلال الأربع أسابيع الماضية، إلى أي مدى كنت تشعر بالعصبية؟	○	○	○	○	○
٣- خلال الأربع أسابيع الماضية، إلى أي مدى كنت تشعر بالعصبية إلى حد أن لاشيء يمكنه أن يجعلك تهدئ؟	○	○	○	○	○
٤- خلال الأربع أسابيع الماضية، إلى أي مدى كنت تشعر باليأس؟	○	○	○	○	○
٥- خلال الأربع أسابيع الماضية، إلى أي مدى كنت تشعر بعدم الراحة أو القلق؟	○	○	○	○	○
٦- خلال الأربع أسابيع الماضية، إلى أي مدى كنت تشعر بعدم الراحة لدرجة أنه لايمكنك الجلوس هادئاً؟	○	○	○	○	○
٧- خلال الأربع أسابيع الماضية، إلى أي مدى كنت تشعر بالإكتئاب؟	○	○	○	○	○
٨- خلال الأربع أسابيع الماضية، إلى أي مدى كنت تشعر إنك تبذل مجهوداً كبيراً عندما تفعل أي شيء؟	○	○	○	○	○
٩- خلال الأربع أسابيع الماضية، إلى أي مدى كنت تشعر بالحزن لدرجة إن لاشيء يحسن من معنوياتك؟	○	○	○	○	○
١٠- خلال الأربع أسابيع الماضية، إلى أي مدى كنت تشعر بأن حياتك ليست لها قيمة أو معنى؟	○	○	○	○	○

PCL-5

فيما يلي قائمة بالمشاكل التى قد يعاني منها بعض الناس كأستجابة لكوفيد-١٩. الرجاء قراءة كل مشكلة بعناية ثم توضيح مدى إنزعاجك لهذه المشكلة خلال الشهر الماضي. خلال الشهر الماضي ما مدى إنزعاجك مما يلي:

	أطلاقاً<br>0	قليلاً<br>1	متوسط<br>2	غالباً<br>3	كثيراً جداً<br>4
١- ذكريات متكررة، مزعجة و غير مرغوب فيها نتيجة التجربة المؤلمة التي مررت بها؟	○	○	○	○	○
٢- أحلام متكررة، مزعجة نتيجة التجربة المؤلمة التي مررت بها؟	○	○	○	○	○
٣ -الشعور أو التصرف المفاجىء كما لو أن التجربة المؤلمة تحدث مرة آخرى (كأنك تعيش الحادث من جديد)؟	○	○	○	○	○
٤- الشعور بالإستياء الشديد عندما يذكرك شيء ما بالتجربة المؤلمة؟	○	○	○	○	○
٥- تعرضك لردة فعل جسدية قوية عند حدوث شيء يذكرك بالتجربة المؤلمة (مثل خفقان بالقلب، صعوبة في التنفس أو التعرق)؟	○	○	○	○	○
٦- تجنب الذكريات، الأفكار أو المشاعر المتعلقة بالتجربة المؤلمة؟	○	○	○	○	○
٧- تتجنب الأشياء الخارجية التي تذكرك بالتجربة المؤلمة (مثل الأشخاص، الأماكن، المحادثات، الأنشطة، الأشياء أو المواقف)؟	○	○	○	○	○
٨- تعاني من مشاكل تذكرك بأجزاء مهمة متعلقة بالتجربة المؤلمة؟	○	○	○	○	○
٩- أصبح لديك معتقدات سلبية قوية متعلقة بك أو عن الأشخاص الأخرين أو العالم الخارجي (مثل لديك أفكار مثل :أنا سيء، هناك شيء خاطىء فيّ، لا يمكن الوثوق بأحد أو العالم بأكمله)؟	○	○	○	○	○
١٠- إلقاء اللوم على نفسك أو على شخص آخر فيما يخص حدوث التجربة المؤلمة أوتبعاتها؟	○	○	○	○	○
١١- وجود مشاعر سلبية قوية مثل الخوف، الرعب، الغضب، الشعور بالذنب أو العار؟	○	○	○	○	○
١٢- فقدان الإهتمام بالأنشطة التي كنت تستمتع بها؟	○	○	○	○	○

Tanveer S, *et al. BMJ Open* 2023; 13:e067886. doi: 10.1136/bmjopen-2022-067886

١٣ـ الشعور بالبعد أو العزلة عن الأشخاص الأخرين؟

١٤ـ صعوبة الإحساس بالمشاعر الإيجابية (مثل عدم التمكن من الشعور بالسعادة أو مشاعر حب تجاه المقربين إليك)؟

١٥ـ سرعة الغضب أو المرور بنوبات الغضب أو التصرف بعدوانية؟

١٦ -الخوض في الكثير من المخاطر أو الأشياء التي قد تسبب لك الضرر؟

١٧ـ البقاء في حالة تأهب قصوى، إحتراس أو حذر شديدين؟

١٨ـ شعور متقلب أو الإستفزاز بسهولة؟

١٩ـ صعوبة في التركيز؟

٢٠ـ مشاكل في الرغبة في النوم أو في البقاء نائماً؟

Tanveer S, *et al. BMJ Open* 2023; 13:e067886. doi: 10.1136/bmjopen-2022-067886

WHO-5

يرجى وضع إشارة عند كل من الأسئلة الخمس التالية، حيث تمثل الإجابة شعورك خلال الإسبوعين الماضيين.

	**دائماً<br>5**	**أكثر الأحيان<br>4**	**أكثر من نصف الوقت<br>3**	**أقل من نصف الوقت<br>2**	**بعض الوقت<br>1**	**بتاتاً<br>0**
١- كنت سعيداً و بمزاجٍ جيد.	○	○	○	○	○	○
٢- كنت أشعُر بالهدوء و الإسترخاء.	○	○	○	○	○	○
٣- كنت أشعر بالحيوية و النشاط.	○	○	○	○	○	○
٤- كنت أستيقظ نشطاً و مرتاحاً.	○	○	○	○	○	○
٥- كانت أيامي مليئةً بأشياء محببة لنفسي.	○	○	○	○	○	○

Tanveer S, *et al. BMJ Open* 2023; 13:e067886. doi: 10.1136/bmjopen-2022-067886

PGTI

من خلال الأسئلة التالية يرجى تحديد الدرجة المناسبة لمدى التغيير الذي احدثته جائحة كوفيد-١٩ على حياتك.

	**لا على الإطلاق 0**	**قليلاً 1**	**إلى حد ما 2**	**بشكلٍ معتدل 3**	**إلى حد قليل 4**	**كثيراً 5**
١- قمت بتغيير أولوياتي عما هو مهم في الحياة.						
٢- أصبح لديَّ تقديراً أكبر لقيمة حياتي.						
٣- طوّرت اهتمامات جديدة.						
٤- لديّ إحساس أكبر بالإعتماد على الذات.						
٥- لديّ فهمًا أفضل للمسائل الروحية.						
٦- أرى بوضوح أكثر أنه يمكنني الإعتماد على الأشخاص في أوقات المشاكل.						
٧- أنشأت مساراً جديداً لحياتي.						
٨- أصبح لديّ إحساس كبير بالقرب من الآخرين.						
٩- أصبحت أكثر استعداداً للتعبير عن مشاعري.						
١٠- أستطيع التعامل مع الصعوبات.						
١١- أستطيع القيام بأشياء أفضل في حياتي.						
١٢- أصبحت أفضل تقبلاً للطرق التي تحل بها الأمور.						
١٣- يمكنني تقدير كل يوم بشكلٍ أفضل.						

١٤- تتوفر فرص جديدة لم تكن ستتوفر بطريقة أخرى.

١٥- أشعر بمزيد من التعاطف تجاه الآخرين.

١٦- أضع مزيداً من الجهد في علاقاتي.

١٧- احاول جاهداً تغيير الأشياء التي تحتاج إلى التغيير.

١٨- إزدادت قوة إيماني.

١٩- اكتشفت أنني أقوى مما كنت أتصور.

٢٠- تعلمت قدراً كبيراً عن مدى حسن خُلق الأشخاص.

٢١- أتقبل بشكلٍ أفضل احتياجي للآخرين .

International Study – Indonesia (Bahasa Indonesia)

Sociodemographic Information

1. Jenis Kelamin

- ○ Laki-laki
- ○ Perempuan
- ○ Lainnya
- ○ Memilih untuk tidak menjawab

2. Umur (tahun)

____

3. Status pernikahan

- ○ Belum menikah
- ○ Sedang memiliki hubungan
- ○ Menikah
- ○ Berpisah
- ○ Cerai hidup
- ○ Cerai mati

4. Apakah agama yang Anda anut sekarang?

- ○ Tidak beragama
- ○ Islam
- ○ Kristen/Katolik
- ○ Hindu
- ○ Buddha

○ Lain-lain (Sebutkan) ____

5. Apa latar belakang etnisitas/suku bangsa yang Anda miliki? (Sebutkan dan Anda boleh menyebutkan lebih dari satu etnis/suku bangsa)

____

6. Pendidikan tertinggi yang ditamatkan?

○ Pendidikan non formal (homeschooling, les privat/les Bersama, pelatihan)

○ SMA/SMK dan sederajat

○ Perguruan Tinggi D3

○ Perguruan Tinggi S1/D4

○ Perguruan Tinggi S2/S3

7. Status pekerjaan dan pendidikan yang saat ini dilakukan (Beri tanda pada pilihan Anda)

☐ Belajar penuh waktu

☐ Belajar paruh waktu

☐ Tidak bekerja

☐ Bekerja penuh waktu (karyawan/pegawai)

☐ Bekerja paruh waktu (karyawan/pegawai)

☐ Bekerja berusaha sendiri/wiraswasta penuh waktu (Full-time self employed)

☐ Bekerja berusaha sendiri/wiraswasta paruh waktu (Part-time self employed)

☐ Cuti diluar tanggungan negara (jika Anda berstatus ASN ataupun tugas belajar)

☐ Bekerja di rumah, tanpa menerima gaji (misal: ibu rumah tangga)

☐ Pensiun/Purna Tugas

☐ Lainnya (Sebutkan) ____

8. Pekerjaan (jika saat ini sedang melakukan kerja penuh waktu dan atau kerja paruh waktu)

____

9. Berapakah pendapatan dan penghasilan Anda (termasuk suami/istri, jika menikah) dalam 1 bulan (per bulan-Rp)

____

9a. Dibandingkan dengan pendapatan rata-rata nasional penduduk Indonesia tahun 2020 berdasarkan data BPS Februari, 2020 (Rp 1,6 juta per bulan), maka bagaimana Anda menilai posisi pendapatan keluarga Anda per bulan?

○ Dibawah rata-rata

○ Rata-rata

○ Diatas rata-rata

10. Apakah saat ini berada pada lingkungan yang melakukan Pemberlakukan Pembatasan Kegiatan Masyarakat (PPKM)?

○ Iya

○ Tidak

11. Apakah Anda sudah mendapatkan vaksin Covid-19?

○ Iya

○ Tidak

11a. Jika belum mendapatkan vaksin, apakah Anda bersedia untuk divaksinasi di kemudian hari?

○ Iya

○ Tidak

12. Kami menyadari bahwa sebagian orang memiliki pengalaman traumatik di masa lalu yang membuat seseorang akan lebih mudah atau sulit untuk menjalani kehidupan, menghadapi tantangan baru maupun mengatasi permasalahan baru yang muncul di kemudian hari.

Sebelum wabah Covid-19, apakah Anda pernah mengalami ataupun menyaksikan beberapa kejadian seperti di bawah ini: (Beri tanda sebanyak mungkin sesuai yang Anda alami)

☐ Bencana alam (banjir, gempa bumi, tsunami, tanah longsor dsb)

☐ Hidup di zona konflik atau peperangan ataupun daerah darurat militer

☐ Pengalaman tidak menyenangkan ketika masa kecil sebelum usia 15 tahun (diabaikan, ditelantarkan orang tua, dibuli, atau kekerasan fisik-mental/seksual)

☐ Kekerasan fisik-mental/seksual Ketika usia diatas 16 tahun

☐ Kecelakaan fisik yang serius

☐ Lainnya, sebutkan pengalaman lainnya yang menyebabkan traumatik di masa lalu ____

☐ Tidak pernah mengalami pengalaman traumatik di masa lalu

Covid Psychosocial Impacts Scale

Dampak terhadap Pribadi

Berikan respon Ya / Tidak terhadap pertanyaaan di bawah ini, jika hal berikut dialami oleh Anda terkait dengan wabah Covid-19. Jika jawaban anda Ya, mohon berikan indikasi penilaian tekanan (stress) yang dialami seusai dengan skala berikut.

1. Apakah Anda mempunyai masalah kesehatan yang membuat Anda rentan/beresiko terhadap Covid-19?

- ○ **Tidak 0**
- ○ **Ya** Tidak membuat stress 1
- ○ **Ya** Sedikit membuat stress 2
- ○ **Ya** Cukup membuat stress 3
- ○ **Ya** Banyak membuat stress 4
- ○ **Ya** Sangat membuat stress 5

2. Apakah Anda merasa berisiko terpapar dengan orang yang terkena Covid-19?

- ○ **Tidak 0**
- ○ **Ya** Tidak membuat stress 1
- ○ **Ya** Sedikit membuat stress 2
- ○ **Ya** Cukup membuat stress 3
- ○ **Ya** Banyak membuat stress 4
- ○ **Ya** Sangat membuat stress 5

3. Apakah Anda merasa pernah terkena Covid-19?

- ○ **Tidak 0**
- ○ **Ya** Tidak membuat stress 1
- ○ **Ya** Sedikit membuat stress 2
- ○ **Ya** Cukup membuat stress 3

- **Ya** Banyak membuat stress 4
- **Ya** Sangat membuat stress 5

3a. Apakah Anda pernah mendapatkan hasil positif dari tes Covid-19?

- **Tidak 0**
- **Ya** Tidak membuat stress 1
- **Ya** Sedikit membuat stress 2
- **Ya** Cukup membuat stress 3
- **Ya** Banyak membuat stress 4
- **Ya** Sangat membuat stress 5

3b. Apakah saat ini Anda merasa mengalami gejala Covid-19?

- **Tidak 0**
- **Ya** Tidak membuat stress 1
- **Ya** Sedikit membuat stress 2
- **Ya** Cukup membuat stress 3
- **Ya** Banyak membuat stress 4
- **Ya** Sangat membuat stress 5

Dampak terhadap Keluarga

Berikan respon Ya/Tidak terhadap pertanyaan di bawah ini, jika hal berikut dialami oleh anggota keluarga dekat Anda terkait dengan wabah Covid-19. Jika jawaban anda Ya, mohon berikan indikasi penilaian tekanan (stress) yang dialami sesuai dengan skala berikut.

1. Apakah **keluarga dekat Anda mempunyai masalah** kesehatan **yang membuat mereka rentan/berisiko terhadap Covid-19?**

- **Tidak 0**

- **Ya** Tidak membuat stress 1
- **Ya** Sedikit membuat stress 2
- **Ya** Cukup membuat stress 3
- **Ya** Banyak membuat stress 4
- **Ya** Sangat membuat stress 5

2. Apakah Anda merasa anggota keluarga Anda berisiko berhadapan dengan orang yang terkena Covid-19?

- **Tidak** 0
- **Ya** Tidak membuat stress 1
- **Ya** Sedikit membuat stress 2
- **Ya** Cukup membuat stress 3
- **Ya** Banyak membuat stress 4
- **Ya** Sangat membuat stress 5

3. Apakah Anda merasa ada anggota keluarga terdekat yang terkena Covid-19?

- **Tidak** 0
- **Ya** Tidak membuat stress 1
- **Ya** Sedikit membuat stress 2
- **Ya** Cukup membuat stress 3
- **Ya** Banyak membuat stress 4
- **Ya** Sangat membuat stress 5

3a. Apakah ada anggota keluarga terdekat anda yang dites hasilnya positif Covid-19?

- **Tidak** 0

- **Ya** Tidak membuat stress 1
- **Ya** Sedikit membuat stress 2
- **Ya** Cukup membuat stress 3
- **Ya** Banyak membuat stress 4
- **Ya** Sangat membuat stress 5

3b. Apakah ada anggota keluarga terdekat anda mengalami gejala Covid-19?

- **Tidak** 0
- **Ya** Tidak membuat stress 1
- **Ya** Sedikit membuat stress 2
- **Ya** Cukup membuat stress 3
- **Ya** Banyak membuat stress 4
- **Ya** Sangat membuat stress 5

3c. Apakah ada anggota keluarga terdekat anda meninggal dunia karena Covid-19?

- **Tidak** 0
- **Ya** Tidak membuat stress 1
- **Ya** Sedikit membuat stress 2
- **Ya** Cukup membuat stress 3
- **Ya** Banyak membuat stress 4
- **Ya** Sangat membuat stress 5

Apakah Anda memiliki anggota keluarga terdekat yang menetap di luar negeri yang Anda khawatirkan berisiko terkena Covid-19?

- **Tidak** 0

- **Ya** Tidak membuat stress 1
- **Ya** Sedikit membuat stress 2
- **Ya** Cukup membuat stress 3
- **Ya** Banyak membuat stress 4
- **Ya** Sangat membuat stress 5

5. Apakah Anda memiliki keluarga dekat di luar negeri yang tidak dapat Anda kunjungi atau mereka tidak dapat mengunjungi Anda saat ini?

- **Tidak** 0
- **Ya** Tidak membuat stress 1
- **Ya** Sedikit membuat stress 2
- **Ya** Cukup membuat stress 3
- **Ya** Banyak membuat stress 4
- **Ya** Sangat membuat stress 5

Pekerjaan

Berikan respon Ya / Tidak / Tidak berlaku terhadap pertanyaaan di bawah ini, jika hal berikut dialami oleh Anda atau anggota keluarga Anda terkait dengan wabah Covid-19. Jika jawaban anda Ya, mohon berikan indikasi penilaian tekanan (stress) yang dialami sesuai dengan skala berikut.

1. Apakah Anda atau anggota keluarga Anda termasuk pekerja di sektor penting (misalnya bekerja di bidang kesehatan, penegak hukum, keuangan, petugas yang berkaitan dengan kedaruratan atau penyedia jasa layanan penting lainnya)?

- **Tidak** 0
- **Ya** Tidak membuat stress 1
- **Ya** Sedikit membuat stress 2
- **Ya** Cukup membuat stress 3

- **Ya** Banyak membuat stress 4
- **Ya** Sangat membuat stress 5

2. Apakah Anda atau anggota keluarga Anda mengalami perubahan (penambahan/pengurangan) jam kerja?

- **Tidak** 0
- **Ya** Tidak membuat stress 1
- **Ya** Sedikit membuat stress 2
- **Ya** Cukup membuat stress 3
- **Ya** Banyak membuat stress 4
- **Ya** Sangat membuat stress 5

3. Apakah Anda atau anggota keluarga Anda harus berubah tempat kerjanya menjadi bekerja dari rumah?

- **Tidak** 0
- **Ya** Tidak membuat stress 1
- **Ya** Sedikit membuat stress 2
- **Ya** Cukup membuat stress 3
- **Ya** Banyak membuat stress 4
- **Ya** Sangat membuat stress 5

4. Apakah Anda atau anggota keluarga Anda terpaksa berubah jenis pekerjaannya?

- **Tidak** 0
- **Ya** Tidak membuat stress 1
- **Ya** Sedikit membuat stress 2
- **Ya** Cukup membuat stress 3

○ **Ya** Banyak membuat stress 4

○ **Ya** Sangat membuat stress 5

5. Apakah Anda atau anggota keluarga Anda kehilangan pekerjaan?

○ **Tidak Berlaku**

○ **Tidak** 0

○ **Ya** Tidak membuat stress 1

○ **Ya** Sedikit membuat stress 2

○ **Ya** Cukup membuat stress 3

○ **Ya** Banyak membuat stress 4

○ **Ya** Sangat membuat stress 5

6. Apakah usaha Anda atau usaha anggota keluarga Anda mengalami kesulitan bertahan saat ini?

○ **Tidak Berlaku**

○ **Tidak** 0

○ **Ya** Tidak membuat stress 1

○ **Ya** Sedikit membuat stress 2

○ **Ya** Cukup membuat stress 3

○ **Ya** Banyak membuat stress 4

○ **Ya** Sangat membuat stress 5

Pendapatan

Berikan respon Ya / Tidak terhadap pertanyaaan di bawah ini, jika hal berikut dialami oleh Anda, keluarga Anda atau anggota keluarga Anda terkait dengan wabah Covid-19. Jika jawaban anda Ya, mohon berikan indikasi penilaian tekanan (stress) yang dialami sesuai dengan skala berikut.

1. Apakah keluarga Anda mengalami penurunan pendapatan?

- **Tidak** 0
- **Ya** Tidak membuat stress 1
- **Ya** Sedikit membuat stress 2
- **Ya** Cukup membuat stress 3
- **Ya** Banyak membuat stress 4
- **Ya** Sangat membuat stress 5

2. Apakah keluarga Anda mengalami penurunan pendapatan yang mengakibatkan Anda kesulitan membayar tagihan atau membeli makanan/bahan pokok makanan?

- **Tidak** 0
- **Ya** Tidak membuat stress 1
- **Ya** Sedikit membuat stress 2
- **Ya** Cukup membuat stress 3
- **Ya** Banyak membuat stress 4
- **Ya** Sangat membuat stress 5

Hubungan Keluarga

Berikan respon Ya/Tidak terhadap pertanyaan di bawah ini, jika hal berikut dialami oleh Anda terkait dengan wabah Covid-19. Jika jawaban anda Ya, mohon berikan indikasi penilaian tekanan (stress) yang dialami sesuai dengan skala berikut. Akibat dari Covid-19……….

1. Meningkatkan pertengkaran/memperburuk komunikasi/hubungan menjadi renggang-jauh dalam keluarga Anda?

- **Tidak** 0
- **Ya** Tidak membuat stress 1
- **Ya** Sedikit membuat stress 2

- **Ya** Cukup membuat stress 3
- **Ya** Banyak membuat stress 4
- **Ya** Sangat membuat stress 5

2. Hubungan Anda dengan teman/kerabat (baik secara fisik maupun daring/online) menurun?

- **Tidak** 0
- **Ya** Tidak membuat stress 1
- **Ya** Sedikit membuat stress 2
- **Ya** Cukup membuat stress 3
- **Ya** Banyak membuat stress 4
- **Ya** Sangat membuat stress 5

Perubahan dalam kegiatan dan perilaku sehari-hari

Berikan respon Ya / Tidak / Tidak Berlaku terhadap pertanyaan di bawah ini, jika hal berikut dialami oleh Anda terkait dengan wabah Covid-19. Jika jawaban anda Ya, mohon berikan indikasi penilaian tekanan (stress) yang dialami sesuai dengan skala berikut.

1. Apakah wabah Covid-19 telah merubah kegiatan sehari-hari Anda (Sebagai contoh, tidur, olah raga, waktu bebas, aktifitas yang menyenangkan atau hobi)?

- **Tidak** 0
- **Ya** Tidak membuat stress 1
- **Ya** Sedikit membuat stress 2
- **Ya** Cukup membuat stress 3
- **Ya** Banyak membuat stress 4
- **Ya** Sangat membuat stress 5

Tanveer S, *et al. BMJ Open* 2023; 13:e067886. doi: 10.1136/bmjopen-2022-067886

2. Membuat Anda menunda/menghindar untuk mencari bantuan kesehatan saat Anda atau keluarga Anda membutuhkannya?

- ○ **Tidak** 0
- ○ **Ya** Tidak membuat stress 1
- ○ **Ya** Sedikit membuat stress 2
- ○ **Ya** Cukup membuat stress 3
- ○ **Ya** Banyak membuat stress 4
- ○ **Ya** Sangat membuat stress 5

3. Menurunkan keterlibatan Anda dalam aktifitas keagamaan (baik tatap muka ataupun online)?

- ○ **Tidak Berlaku**
- ○ **Tidak** 0
- ○ **Ya** Tidak membuat stress 1
- ○ **Ya** Sedikit membuat stress 2
- ○ **Ya** Cukup membuat stress 3
- ○ **Ya** Banyak membuat stress 4
- ○ **Ya** Sangat membuat stress 5

4. Apakah ada perubahan besar terhadap keseharian Anda?

- ○ **Tidak** 0
- ○ **Jika ada, mohon jelaskan** 1 ____

Jika jawaban anda Ya, mohon berikan indikasi penilaian tekanan (stress) yang dialami sesuai dengan skala berikut.

- ○ Tidak membuat stress 1
- ○ Sedikit membuat stress 2

○ Cukup membuat stress 3

○ Banyak membuat stress 4

○ Sangat membuat stress 5

Paparan terhadap informasi mengenai Covid-19

1. Terkait dengan Covid-19, dari manakah Anda mendapatkan informasinya?
(Mohon dipilih sebanyak mungkin sesuai yang Anda alami)

☐ Berita

☐ Facebook/Twitter

☐ Situs kesehatan

☐ Dari mulut ke mulut

☐ Lain-lain (Mohon dijelaskan) ____

2. Seberapa banyak tekanan (stress) yang Anda alami karena pemberitaan mengenai Covid-19?

○ Tidak membuat stress 1

○ Sedikit membuat stress 2

○ Cukup membuat stress 3

○ Banyak membuat stress 4

○ Sangat membuat stress 5

Perubahan sejak Covid-19

Dari pertanyaan di bawah ini, pilihlah yang Anda rasakan dengan melingkari nomor yang sesuai dengan situasi Anda. Mohon bandingkan saat ini dengan sebelum wabah Covid-19...

1. Bagaimana Anda menilai tingkat tekanan/stres yang dialami?

○ Sangat tidak merasakan tekanan/stress 1

○ Sedikit tidak merasakan tekanan/stress 2

- Tidak ada perubahan 3
- Lebih merasakan tekanan/stress 4
- Sangat merasakan tekanan/stress 5

2. Bagaimana Anda menilai tingkat tekanan (stress) yang dirasakan oleh anggota keluarga Anda secara keseluruhan?

- Sangat tidak merasakan tekanan/stress 1
- Sedikit tidak merasakan tekanan/stress 2
- Tidak ada perubahan 3
- Lebih merasakan tekanan/stress 4
- Sangat merasakan tekanan/stress 5

3. Bagaimana Anda menilai kesejahteraan psikologis Anda?

- Sangat baik 1
- Lebih baik 2
- Tidak ada perubahan 3
- Lebih buruk 4
- Sangat buruk 5

4. Bagaimana Anda menilai kesejahteraan psikologis anggota keluarga Anda secara keseluruhan?

- Sangat baik 1
- Lebih baik 2
- Tidak ada perubahan 3
- Lebih buruk 4
- Sangat buruk 5

K-10

Sepuluh pertanyaan berikut ini menanyakan perasaan Anda dalam 4 minggu terakhir. Untuk setiap pertanyaan, mohon lingkari pilihan yang menjelaskan seringnya Anda merasakan hal tersebut.

	Tidak pernah 1	Jarang 2	Kadang-kadang 3	Hampir selalu 4	Setiap saat 5
1. Dalam kurun waktu empat minggu terakhir, seberapa sering Anda merasa lelah tanpa sebab yang jelas?	○	○	○	○	○
2. Dalam empat minggu terakhir, seberapa sering Anda merasa cemas?	○	○	○	○	○
3. Dalam empat minggu terakhir seberapa sering Anda merasa cemas dan tidak bisa menenangkan diri?	○	○	○	○	○
4. Dalam empat minggu terakhir, seberapa sering Anda merasa tidak punya harapan?	○	○	○	○	○
5. Dalam empat minggu terakhir, seberapa sering Anda merasa gelisah atau resah?	○	○	○	○	○
6. Dalam empat minggu terakhir, seberapa sering Anda merasa gelisah yang menyebabkan Anda tidak dapat duduk diam?	○	○	○	○	○
7. Dalam empat minggu terakhir, seberapa sering Anda merasa tertekan?	○	○	○	○	○
8. Dalam empat minggu terakhir, seberapa sering Anda merasa berat sekali untuk melakukan sesuatu?	○	○	○	○	○
9. Dalam empat minggu terakhir, seberapa sering Anda merasa sedih sehingga tidak ada apapun yang dapat menghibur Anda?	○	○	○	○	○
10. Dalam empat minggu terakhir, seberapa sering Anda merasa tidak berharga sama sekali?	○	○	○	○	○

Tanveer S, *et al. BMJ Open* 2023; 13:e067886. doi: 10.1136/bmjopen-2022-067886

PCL-5

Di bawah ini adalah masalah-masalah yang seringkali dialami seseorang dalam menghadapi situasi Covid-19. Mohon dibaca setiap masalah dengan teliti dan kemudian lingkari nomor untuk mengindikasikan seberapa sering Anda terganggu oleh masalah tersebut dalam kurun waktu satu bulan terakhir. Dalam satu Bulan terakhir, seberapa sering Anda terganggu dengan hal berikut:

	**Tidak sama sekali** 0	**Sedikit tergganggu** 1	**Kadang-kadang tergganggu** 2	**Hampir tergganggu** 3	**Sangat tergganggu** 4
1. Terulangnya kenangan yang tidak diinginkan dan membuat tertekan/stres?	○	○	○	○	○
2. Terulangnya mimpi yang menganggu saat mengalami tekanan/stres?	○	○	○	○	○
3. Tiba-tiba merasakan atau bertingkah seakan-akan pengalaman saat tekanan/stres terjadi terulang kembali (seakan-akan Anda kembali ke situasi tersebut?	○	○	○	○	○
4. Merasa marah saat terjadi sesuatu yang mengingatkan Anda pada pengalaman saat tekanan/stres terjadi?	○	○	○	○	○
5. Mengalami reaksi fisik yang kuat saat sesuatu terjadi dimana mengingatkan Anda pada pengalaman saat tekanan/stres terjadi (sebagai contoh, jantung berdebar kencang, mengalami kesusahan bernafas, berkeringat)?	○	○	○	○	○
6. Menghindari kenangan, pikiran ataupun perasaan yang terkait dengan pengalaman saat tekanan/stres terjadi?	○	○	○	○	○
7. Menghindari hal-hal dari pihak luar yang mengingatkan Anda kepada pengalaman saat tekanan/stres terjadi (sebagai contoh, orang, tempat, percakapan, aktifitas, benda atau situasi)?	○	○	○	○	○
8. Kesulitan dalam mengingat hal penting dari pengalaman saat tekanan/stres terjadi?	○	○	○	○	○

Tanveer S, et al. BMJ Open 2023; 13:e067886. doi: 10.1136/bmjopen-2022-067886

9. Mengalami prasangka negatif yang kuat terhadap diri sendiri, orang lain, atau dunia (sebagai contoh, memiliki pikiran seperti: saya jahat, ada hal yang salah di dalam diri saya, tidak ada yang bisa dipercaya, dunia ini sangat kejam)? ○ ○ ○ ○ ○

10. Menyalahkan diri sendiri atau orang lain terhadap pengalaman saat tekanan/stres terjadi ataupun terhadap hal yang terjadi setelahnya? ○ ○ ○ ○ ○

11. Memilik perasaan negatif yang kuat seperti ketakutan, perasaan ngeri, marah, merasa bersalah ataupun malu? ○ ○ ○ ○ ○

12. Tidak tertarik lagi melakukan kegiatan yang biasanya Anda sukai? ○ ○ ○ ○ ○

13. Merasa jauh dan tidak memiliki hubungan dengan orang lain? ○ ○ ○ ○ ○

14. Kesulitan untuk memiliki perasaan positif (sebagai contoh, tidak dapat merasakan kebahagian atau mencintai orang yang dekat dengan Anda)? ○ ○ ○ ○ ○

15. Bertingkah laku yang mengganggu, marah tanpa kendali atau bersikap agresif? ○ ○ ○ ○ ○

16. Melakukan hal yang berisiko tinggi ataupun melakukan hal yang dapat membahayakan diri Anda? ○ ○ ○ ○ ○

17. Bersikap “sangat waspada” atau berhati-hati atau pun berjaga-jaga? ○ ○ ○ ○ ○

18. Merasa gelisah dan mudah terkejut? ○ ○ ○ ○ ○

19. Kesulitan dalam berkonsentrasi? ○ ○ ○ ○ ○

20. Susah tertidur atau tidur lebih lama? ○ ○ ○ ○ ○

WHO-5

Mohon berikan indikasi untuk ke lima pernyataan di bawah ini yang sesuai dengan yang Anda rasakan selama kurun waktu 2 minggu terakhir. Dalam kurun waktu 2 minggu terakhir

	**Setiap waktu 5**	**Kebanyakan waktu 4**	**Sebagian waktu 3**	**Hampir sebagian waktu 2**	**Sesekali 1**	**Tidak sama sekali 0**
1. Saya merasa gembira dan bahagia	○	○	○	○	○	○
2. Saya merasa tenang dan rileks	○	○	○	○	○	○
3. Saya merasa aktif dan semangat	○	○	○	○	○	○
4. Saya terbangun dengan perasaan segar dan cukup istirahat	○	○	○	○	○	○
5. Kegiatan sehari-hari saya penuh dengan hal-hal yang saya sukai	○	○	○	○	○	○

PTGI

Dari pernyataan di bawah ini, mohon indikasikan penilaian terjadinya perubahan dalam hidup Anda sebagai akibat dari Covid-19 dengan menggunakan skala di bawah ini.

	**Tidak sama sekali** 0	**Sedikit** 1	**Agak** 2	**Kadang-kadang** 3	**Hampir** 4	**Sangat** 5
1. Saya merubah prioritas mengenai apa yang penting dalam hidup.	○	○	○	○	○	○
2. Saya memiliki apresiasi besar terhadap nilai hidup.	○	○	○	○	○	○
3. Saya mengembangkan minat baru.	○	○	○	○	○	○
4. Saya memiliki perasaan besar terhadap kepercayaan diri.	○	○	○	○	○	○
5. Saya memiliki pemahaman yang baik terhadap masalah spiritual.	○	○	○	○	○	○
6. Saya melihat bahwa saya bisa meminta bantuan kepada orang lain di saat kesulitan.	○	○	○	○	○	○
7. Saya menempuh jalan baru bagi hidup saya.	○	○	○	○	○	○
8. Saya memiliki rasa kedekatan dengan orang lain.	○	○	○	○	○	○
9. Saya lebih bisa mengekspresikan emosi.	○	○	○	○	○	○
10. Saya tahu jika saya bisa mengatasi kesulitan.	○	○	○	○	○	○
11. Saya bisa melakukan hal-hal baik dalam hidup saya.	○	○	○	○	○	○
12. Saya semakin bisa menerima hal-hal yang terjadi.	○	○	○	○	○	○
13. Saya bisa lebih menghargai setiap hari yang saya lalui.	○	○	○	○	○	○

Tanveer S, *et al. BMJ Open* 2023; 13:e067886. doi: 10.1136/bmjopen-2022-067886

14. Kesempatan baru tersedia dibandingkan sebelumnya.

15. Saya lebih memiliki rasa kasih sayang kepada sesama.

16. Saya lebih memberikan upaya dalam hubungan saya dengan orang lain

17. Saya lebih bisa mengusahakan untuk merubah hal yang memang bisa diubah.

18. Saya memiliki keyakinan yang lebih kuat terhadap agama.

19. Saya menyadari bahwa saya lebih kuat daripada yang saya kira.

20. Saya menyadari bahwa ternyata orang lain itu baik.

21. Saya lebih bisa menerima bahwa saya membutuhkan orang lain.

International Study – Malaysia (Malay)

Sociodemographic Information

1. Jantina

- ◯ Lelaki
- ◯ Perempuan
- ◯ Lain-lain
- ◯ Lebih suka tidak diberitahu

2. Umur (dalam tahun)

____

3. Status perkahwinan

- ◯ Bujang
- ◯ Mempunya hubungan
- ◯ Berkahwin
- ◯ Tidak tinggal bersama
- ◯ Bercerai
- ◯ Duda/Janda

4. Apakah agama anda?

- ◯ Tiada agama
- ◯ Islam
- ◯ Kristian
- ◯ Hindu

- ○ Buddha
- ○ Lain-lain (Sila nyatakan) ____

5. Apakah bangsa anda? (Anda boleh menyebut lebih daripada satu)

____

6. Apakah Bahasa yang paling fasih digunakan dalam kehidupan seharian?

____

Berikut merupakan dua soalan berkenaan kebolehan anda bertutur di dalam Bahasa Inggeris. 6a. Saya faham Bahasa Inggeris dengan baik

- ○ Sangat tidak setuju 1
- ○ Tidak setuju 2
- ○ Natural 3
- ○ Setuju 4
- ○ Sangat Setuju 5

6b. Saya boleh bertutur Bahasa Inggeris dengan baik

- ○ Tidak langsung 1
- ○ Sedikit 2
- ○ Lebih kurang 3
- ○ Agak banyak 4
- ○ Sangat banyak 5

7. Apakah pendidikan tertinggi anda?

- ○ Tiada Pendidikan formal

- ○ Matrikulasi
- ○ Diploma / Sijil
- ○ Sarjana Muda
- ○ Sarjana / PhD

8. Status belajar/bekerja (tandakan yang berkenaan)

- ☐ Pelajar sepenuh masa
- ☐ Pelajar separuh masa
- ☐ Tidak bekerja/Sedang mencari pekerjaan
- ☐ Bekerja sepenuh masa
- ☐ Bekerja separuh masa
- ☐ Bekerja Sendiri sepenuh masa
- ☐ Bekerja Sendiri separuh masa
- ☐ Cuti bergaji
- ☐ Tinggal bersama ibubapa (tidak bergaji)
- ☐ Bersara
- ☐ Lain-lain (Sila nyatakan) ____

9. Pekerjaan

____

10. Pendapatan bulanan isirumah (Ringgit Malaysia)

____

10a. Apakah kadar pendapatan bulanan isirumah, jika dibandingkan dengan purata pendapatan penduduk Malaysia?

○ Bawah purata

○ Sama dengan purata

○ Atas purata

11. Adakah anda sekarang berada di dalam perintah kawalan pergerakan/arahan duduk di rumah?

○ Ya

○ Tidak

12. Adakahand telah menerima suntikan vaksin?

○ Ya

○ Tidak

12a. Jika belum menerima suntikan vaksin, adakah anda akan mendapatkan vaksin COVID-19 di masa akan datang?

○ Ya

○ Tidak

13. Kita sedar bahawa sesetengah orang telah didedahkan dengan insiden trauma pada masa lampau yang mana telah memudahkan atau menyusahkan untuk menangani masalah. Sebelum pandemic COVID-19, adakah anda pernah menyaksikan atau mengalami mana-mana perkara berikut: (Sila tanda sebanyak mana yang berkaitan)

☐ Bencana alam (seperti banjir, gempa bumi dan lain-lain)

☐ Tinggal di dalam zon peperangan atau telah terdedah kepada konflik tetera

☐ Kesukaran kanak-kanak sebelum usia 16 tahun (seperti pengabaian, buli, serangan fizikal atau seksual)

- [ ] Serangan fizikal atau seksual selepas usia 16 tahun
- [ ] Kemalangan fizikal yang serius
- [ ] Lain-lain - sila nyatakan (pilihan) ____
- [ ] Tiada satu pun di atas

Covid Psychosocail Impacts Scale

Kesan keatas diri sendiri

Untuk kenyataan di bawah, sila jawab Ya/Tidak jika kesan daripada Covid-19, berlaku kepada diri anda sendiri. Jika jawapan anda adalah Ya, sila nyatakan tahap tekanan yang dialami, berdasarkan skala dibawah.

1. Adakah anda mengalami masalah kesihatan yang boleh menyebabkan risiko anda untuk dijangkiti Covid-19 meningkat?

- ○ **Tidak** 0
- ○ **Ya** Tiada tekanan langsung 1
- ○ **Ya** Tekanan tahap minima 2
- ○ **Ya** Tekanan tahap sederhana 3
- ○ **Ya** Tekanan tahap tinggi 4
- ○ **Ya** Tekanan yang sangat tinggi 5

2. Adakah anda pernah merasakan bahaya risiko terdedah kepada seseorang yang menghidap Covid-19?

- ○ **Tidak** 0
- ○ **Ya** Tiada tekanan langsung 1
- ○ **Ya** Tekanan tahap minima 2
- ○ **Ya** Tekanan tahap sederhana 3
- ○ **Ya** Tekanan tahap tinggi 4
- ○ **Ya** Tekanan yang sangat tinggi 5

3. Adakah anda fikir bahawa anda telah dijangkiti COVID-19?

- ○ **Tidak** 0

Tanveer S, *et al. BMJ Open* 2023; 13:e067886. doi: 10.1136/bmjopen-2022-067886

- **Ya** Tiada tekanan langsung 1
- **Ya** Tekanan tahap minima 2
- **Ya** Tekanan tahap sederhana 3
- **Ya** Tekanan tahap tinggi 4
- **Ya** Tekanan yang sangat tinggi 5

3a. Adakah anda pernah mendapat keputusan positif bagi ujian COVID-19?

- **Tidak** 0
- **Ya** Tiada tekanan langsung 1
- **Ya** Tekanan tahap minima 2
- **Ya** Tekanan tahap sederhana 3
- **Ya** Tekanan tahap tinggi 4
- **Ya** Tekanan yang sangat tinggi 5

3b. Adakah anda mempunyai simptom-simptom COVID-19?

- **Tidak** 0
- **Ya** Tiada tekanan langsung 1
- **Ya** Tekanan tahap minima 2
- **Ya** Tekanan tahap sederhana 3
- **Ya** Tekanan tahap tinggi 4
- **Ya** Tekanan yang sangat tinggi 5

Tanveer S, *et al. BMJ Open* 2023; 13:e067886. doi: 10.1136/bmjopen-2022-067886

Kesan kepada keluarga

Untuk kenyataan di bawah, sila jawab Ya/Tidak jika kesan daripada Covid-19, berlaku kepada ahli keluarga terdekat anda. Jika jawapan anda adalah Ya, sila nyatakan tahap tekanan yang dialami, berdasarkan skala dibawah.

1. Adakah ahli keluarga terdekat anda mengalami masalah kesihatan yang boleh menyebabkan risiko mereka untuk dijangkiti Covid-19 meningkat?

- ○ **Tidak** 0
- ○ **Ya** Tiada tekanan langsung 1
- ○ **Ya** Tekanan tahap minima 2
- ○ **Ya** Tekanan tahap sederhana 3
- ○ **Ya** Tekanan tahap tinggi 4
- ○ **Ya** Tekanan yang sangat tinggi 5

2. Adakah anda merasakan bahawa setiap ahli keluarga terdekat anda berisiko untuk terdedah kepada seseorang yang menghidap Covid-19?

- ○ **Tidak** 0
- ○ **Ya** Tiada tekanan langsung 1
- ○ **Ya** Tekanan tahap minima 2
- ○ **Ya** Tekanan tahap sederhana 3
- ○ **Ya** Tekanan tahap tinggi 4
- ○ **Ya** Tekanan yang sangat tinggi 5

3. Adakah ahli keluarga terdekat anda pernah menjalani Covid-19?

- ○ **Tidak** 0
- ○ **Ya** Tiada tekanan langsung 1

- **Ya** Tekanan tahap minima 2
- **Ya** Tekanan tahap sederhana 3
- **Ya** Tekanan tahap tinggi 4
- **Ya** Tekanan yang sangat tinggi 5

3a. Adakah keputusan ahli keluarga terdekat anda positif Covid-19?

- **Tidak** 0
- **Ya** Tiada tekanan langsung 1
- **Ya** Tekanan tahap minima 2
- **Ya** Tekanan tahap sederhana 3
- **Ya** Tekanan tahap tinggi 4
- **Ya** Tekanan yang sangat tinggi 5

3b. Adakah ahli keluarga terdekat anda mempunyai simptom-simptom Covid-19?

- **Tidak** 0
- **Ya** Tiada tekanan langsung 1
- **Ya** Tekanan tahap minima 2
- **Ya** Tekanan tahap sederhana 3
- **Ya** Tekanan tahap tinggi 4
- **Ya** Tekanan yang sangat tinggi 5

3c. Adakah ahli keluarga terdekat anda meninggal dunia disebabkan oleh Covid-19?

- **Tidak** 0

- **Ya** Tiada tekanan langsung 1
- **Ya** Tekanan tahap minima 2
- **Ya** Tekanan tahap sederhana 3
- **Ya** Tekanan tahap tinggi 4
- **Ya** Tekanan yang sangat tinggi 5

4. Adakah anda mempunyai ahli keluarga terdekat di luar negara yang dikhuatiri berisiko untuk dijangkiti Covid-19?

- **Tidak** 0
- **Ya** Tiada tekanan langsung 1
- **Ya** Tekanan tahap minima 2
- **Ya** Tekanan tahap sederhana 3
- **Ya** Tekanan tahap tinggi 4
- **Ya** Tekanan yang sangat tinggi 5

5. Adakah anda mempunyai ahli di luar negara yang tidak dapat anda lawati atau tidak dapat melawat anda?

- **Tidak** 0
- **Ya** Tiada tekanan langsung 1
- **Ya** Tekanan tahap minima 2
- **Ya** Tekanan tahap sederhana 3
- **Ya** Tekanan tahap tinggi 4
- **Ya** Tekanan yang sangat tinggi 5

Tanveer S, *et al. BMJ Open* 2023; 13:e067886. doi: 10.1136/bmjopen-2022-067886

Pekerjaan

Untuk kenyataan di bawah, sila jawab Ya / Tidak / Tidak berkenaan jika kesan daripada Covid-19, berlaku kepada anda atau sesiapa sahaja di rumah anda. Jika jawapan anda adalah Ya, sila nyatakan tahap tekanan yang dialami, berdasarkan skala dibawah.

1. Adakah anda atau sesiapa sahaja di rumah anda dianggap sebagai pekerja perkhidmatan perlu (Contohnya, bidang perubatan dan penjagaan kesihatan, penguatkuasaan undang-undang, perkhidmatan kecemasan, pembekal barang dan perkhidmatan penting)?

- **Tidak** 0
- **Ya** Tiada tekanan langsung 1
- **Ya** Tekanan tahap minima 2
- **Ya** Tekanan tahap sederhana 3
- **Ya** Tekanan tahap tinggi 4
- **Ya** Tekanan yang sangat tinggi 5

2. Adakah anda atau sesiapa sahaja di rumah anda mengalami perubahan yang besar pada waktu bekerja?

- **Tidak** 0
- **Ya** Tiada tekanan langsung 1
- **Ya** Tekanan tahap minima 2
- **Ya** Tekanan tahap sederhana 3
- **Ya** Tekanan tahap tinggi 4
- **Ya** Tekanan yang sangat tinggi 5

3. Adakah anda atau sesiapa sahaja di rumah anda perlu bertukar tempat kerja dan bekerja dari rumah?

- **Tidak** 0

Tanveer S, *et al. BMJ Open* 2023; 13:e067886. doi: 10.1136/bmjopen-2022-067886

- **Ya** Tiada tekanan langsung 1
- **Ya** Tekanan tahap minima 2
- **Ya** Tekanan tahap sederhana 3
- **Ya** Tekanan tahap tinggi 4
- **Ya** Tekanan yang sangat tinggi 5

4. Adakah anda atau sesiapa sahaja di rumah anda perlu bertukar jenis pekerjaan?

- **Tidak** 0
- **Ya** Tiada tekanan langsung 1
- **Ya** Tekanan tahap minima 2
- **Ya** Tekanan tahap sederhana 3
- **Ya** Tekanan tahap tinggi 4
- **Ya** Tekanan yang sangat tinggi 5

5. Adakah anda atau ahli rumahtangga anda telah hilang punca pendapatan?

- **Tidak berkenaan**
- **Tidak** 0
- **Ya** Tiada tekanan langsung 1
- **Ya** Tekanan tahap minima 2
- **Ya** Tekanan tahap sederhana 3
- **Ya** Tekanan tahap tinggi 4
- **Ya** Tekanan yang sangat tinggi 5

6. Adakah perniagaan atau sumber pencarian anda atau ahli rumahtangga anda terjejas dan berkemungkinan untuk tidak beroperasi lagi?

- ○ **Tidak berkenaan**
- ○ **Tidak** 0
- ○ **Ya** Tiada tekanan langsung 1
- ○ **Ya** Tekanan tahap minima 2
- ○ **Ya** Tekanan tahap sederhana 3
- ○ **Ya** Tekanan tahap tinggi 4
- ○ **Ya** Tekanan yang sangat tinggi 5

Pendapatan

Untuk kenyataan di bawah, sila jawab Ya/Tidak jika kesan daripada Covid-19 ini telah berlaku kepada ahli rumahtangga anda. Jika jawapan anda adalah Ya, sila nyatakan tahap tekanan yang dialami, berdasarkan skala dibawah.

1. Adakah isu kewangan rumahtangga anda merosot dengan mendadak?

- ○ **Tidak** 0
- ○ **Ya** Tiada tekanan langsung 1
- ○ **Ya** Tekanan tahap minima 2
- ○ **Ya** Tekanan tahap sederhana 3
- ○ **Ya** Tekanan tahap tinggi 4
- ○ **Ya** Tekanan yang sangat tinggi 5

2. Adakah rumahtagga anda menghadapi masalah besar untuk membeli barangan keperluan rumah atau membayar bil-bil semasa?

- **Tidak** 0
- **Ya** Tiada tekanan langsung 1
- **Ya** Tekanan tahap minima 2
- **Ya** Tekanan tahap sederhana 3
- **Ya** Tekanan tahap tinggi 4
- **Ya** Tekanan yang sangat tinggi 5

Perhubungan (bersama insan lain)

Untuk kenyataan di bawah, sila jawab Ya/Tidak jika kesan daripada Covid-19, berlaku kepada diri anda sendiri. Jika jawapan anda adalah Ya, sila nyatakan tahap tekanan yang dialami, berdasarkan skala dibawah. Sebagai tindak balas kepada Covid-19....

1. Adakah ia telah mendatangkan kesusahan dan tekanan kepada keluarga anda?

- **Tidak** 0
- **Ya** Tiada tekanan langsung 1
- **Ya** Tekanan tahap minima 2
- **Ya** Tekanan tahap sederhana 3
- **Ya** Tekanan tahap tinggi 4
- **Ya** Tekanan yang sangat tinggi 5

2. Adakah ia telah mengurangkan perhubungan antara rakan-rakan anda (sama ada secara fizikal ataupun dalam talian)?

- **Tidak** 0
- **Ya** Tiada tekanan langsung 1
- **Ya** Tekanan tahap minima 2

Tanveer S, *et al. BMJ Open* 2023; 13:e067886. doi: 10.1136/bmjopen-2022-067886

- **Ya** Tekanan tahap sederhana 3
- **Ya** Tekanan tahap tinggi 4
- **Ya** Tekanan yang sangat tinggi 5

Perubahan kepada Rutin dan Tingkah Laku Harian

Untuk kenyataan di bawah, sila jawab Ya/Tidak/Tidak berkenaan jika kesan daripada Covid-19, berlaku kepada diri anda sendiri. Jika jawapan anda adalah Ya, sila nyatakan tahap tekanan yang dialami, berdasarkan skala dibawah. Sebagai tindak balas kepada Covid-19....

1. Adakah wabak Covid ini telah mencetus perubahan kepada rutin harian anda (Contohnya, jadual tidur, senaman, waktu rehat, waktu riadah, atau hobi)?

- **Tidak** 0
- **Ya** Tiada tekanan langsung 1
- **Ya** Tekanan tahap minima 2
- **Ya** Tekanan tahap sederhana 3
- **Ya** Tekanan tahap tinggi 4
- **Ya** Tekanan yang sangat tinggi 5

2. Adakah ia telah membuatkan anda menangguhkan atau mengelakkan mencari rawatan perubatan sekiranya anda atau ahli keluarga anda memerlukannya?

- **Tidak** 0
- **Ya** Tiada tekanan langsung 1
- **Ya** Tekanan tahap minima 2
- **Ya** Tekanan tahap sederhana 3
- **Ya** Tekanan tahap tinggi 4

Tanveer S, *et al. BMJ Open* 2023; 13:e067886. doi: 10.1136/bmjopen-2022-067886

- **Ya** Tekanan yang sangat tinggi 5

3. Adakah ia telah mengurangkan penglibatan anda dalam aktiviti keagamaan (sama ada secara peribadi atau dalam talian)?

- **Tidak berknaan**
- **Tidak** 0
- **Ya** Tiada tekanan langsung 1
- **Ya** Tekanan tahap minima 2
- **Ya** Tekanan tahap sederhana 3
- **Ya** Tekanan tahap tinggi 4
- **Ya** Tekanan yang sangat tinggi 5

4. Adakah ia telah menyebabkan sebarang perubahan besar kepada rutin harian anda?

- **Tidak** 0
- **Ya (sila jelaskan)** 1 ____

Jika jawapan anda adalah Ya, sila nyatakan tahap tekanan yang dialami, berdasarkan skala dibawah.

- Tiada tekanan langsung 1
- Tekanan tahap minima 2
- Tekanan tahap sederhana 3
- Tekanan tahap tinggi 4
- Tekanan yang sangat tinggi 5

Pendedahan kepada maklumat tentang Covid-19

1. Apakah sumber maklumat anda berkenaan dengan Covid-19? (Tandakan seberapa banyak yang mungkin)

- ☐ Berita
- ☐ Facebook atau Twitter
- ☐ Laman web kesihatan
- ☐ daripada orang lain
- ☐ Lain-lain (sila jelaskan) ____

2. Sebanyak manakah anda berasa tertekan membaca berita tentang Covid-19?

- ○ Tiada tekanan langsung 1
- ○ Tekanan tahap minima 2
- ○ Tekanan tahap sederhana 3
- ○ Tekanan tahap tinggi 4
- ○ Tekanan yang sangat tinggi 5

Perubahan sejak Covid-19

Untuk soalan-soalan berikutnya, sila nyatakan berdasarkan skala dibawah. Bandingkan keadaan sekarang dengan keadaan semasa sebelum wabak Covid-19 berlaku....

1. Bagaimanakah anda menilai tahap tekanan peribadi anda?

- ○ Sangat tidak tertekan 1
- ○ Kurang tertekan 2
- ○ Lebih kurang sama 3

- ○ Lebih tertekan 4
- ○ Sangat tertekan 5

2. Bagaimanakah anda menilai tahap tekanan ahli rumahtangga anda?

- ○ Sangat tidak tertekan 1
- ○ Kurang tertekan 2
- ○ Lebih kurang sama 3
- ○ Lebih tertekan 4
- ○ Sangat tertekan 5

3. Bagaimanakah anda menilai tahap kesihatan psikologi anda?

- ○ Sangat baik 1
- ○ Baik 2
- ○ Lebih kurang sama 3
- ○ Merosot 4
- ○ Sangat merosot 5

4. Bagaimanakah anda menilai tahap kesihatan psikologi ahli rumahtangga anda?

- ○ Sangat baik 1
- ○ Baik 2
- ○ Lebih kurang sama 3
- ○ Merosot 4
- ○ Sangat merosot 5

Tanveer S, *et al. BMJ Open* 2023; 13:e067886. doi: 10.1136/bmjopen-2022-067886

K-10

Sepuluh soalan berikut bertanyakan tentang perasaan yang anda rasai sejak empat minggu yang lepas. Untuk setiap soalan, sila bulatkan pilihan yang menunjukkan sebanyak mana anda merasai perasaan tersebut.

	**Tidak langsung 1**	**Kadang-kadang 2**	**Sedikit 3**	**Selalu 4**	**Sepanjang masa 5**
1. Dalam tempoh empat minggu yang lepas, berapa kerap anda berasa sangat penat tanpa sebarang sebab yang munasabah?	○	○	○	○	○
2. Dalam tempoh empat minggu yang lepas, berapa kerap anda berasa gugup/gementar?	○	○	○	○	○
3. Dalam tempoh empat minggu yang lepas, berapa kerap anda berasa sangat gugup/gementar sehinggga tiada apa yang boleh menenangkan anda?	○	○	○	○	○
4. Dalam tempoh empat minggu yang lepas, berapa kerap anda berasa putus asa?	○	○	○	○	○
5. Dalam tempoh empat minggu yang lepas, berapa kerap anda berasa gelisah?	○	○	○	○	○
6. Dalam tempoh empat minggu yang lepas, berapa kerap anda berasa sangat gelisah sehingga tidak boleh duduk diam?	○	○	○	○	○
7. Dalam tempoh empat minggu yang lepas, berapa kerap anda mengalami perasaan murung?	○	○	○	○	○
8. Dalam tempoh empat minggu yang lepas, berapa kerap anda berasa seperti segala yang dilakukan memerlukan usaha yang banyak?	○	○	○	○	○
9. Dalam tempoh empat minggu yang lepas, berapa kerap anda berasa sangat sedih sehingga tiada apa boleh menceriakan anda?	○	○	○	○	○
10. Dalam tempoh empat minggu yang lepas, berapa kerap anda merasakan tiada harga diri?	○	○	○	○	○

Tanveer S, *et al. BMJ Open* 2023; 13:e067886. doi: 10.1136/bmjopen-2022-067886

PCL-5

Berikut adalah senarai masalah yang sering dihadapi oleh seseorang individu sebagai tindak balas kepada suatu pengalaman yang sangat menekankan. Sila baca setiap soalan dengan teliti dan pilih satu nombor yang tertera di sebelah kanan untuk menunjukkan sebanyak mana anda terjejas dengan masalah tersebut pada bulan lalu.

Pada bulan lepas, sebanyak mana anda terganggu dengan:

	Tidak langsung 0	Sedikit 1	Sederhana 2	Sering 3	Sangat terjejas 4
1. Kenangan yang tidak diingini, berulang, dan mengganggu disebabkan oleh pengalaman buruk yang dialami?	○	○	○	○	○
2. Mimpi buruk yang berulang disebabkan oleh pengalaman buruk yang dialami?	○	○	○	○	○
3. Tiba-tiba terasa atau berperangai seolah-olah pengalaman buruk yang dialami itu berulang lagi (seakan-akan anda kembali ke situasi tersebut)?	○	○	○	○	○
4. Merasa sangat marah dan sedih apabila anda diingatkan dengan pengalaman buruk yang dialami?	○	○	○	○	○
5.Mengalami tindak balas fizikal yang kuat apabila anda diingatkan dengan pengalaman buruk yang dialami? (Contohnya jantung berdegup dengan laju, sukar bernafas, dan berpeluh).	○	○	○	○	○
6. Mengelak daripada mengingati sebarang kenangan, fikiran, atau perasaan yang berkaitan dengan pengalaman buruk yang dialami?	○	○	○	○	○
7. Mengelak daripada pengaruh luar yang akan mengingatkan kepada pengalaman buruk yang dialami? (Contohnya mengelak daripada berjumpa dengan orang, tempat, perbualan, aktiviti, benda, atau situasi)	○	○	○	○	○
8. Adakah anda berasa susah untuk mengingati hal-hal penting pengalaman buruk yang dialami itu?	○	○	○	○	○

9.Mempunyai tanggapan negatif yang kuat terhadap diri sendiri, orang lain, atau dunia (Contohnya, mempunyai pemikiran seperti: Saya jahat, ada sesuatu yang sangat salah dengan diri saya, tiada siapa boleh dipercayai, segalanya di dunia ini merbahaya)?	○	○	○	○	○
10. Adakah anda menyalahkan diri sendiri atau orang lain kerana berlakunya pengalaman buruk yang telah berlaku atau apa yang terjadi selepas itu?	○	○	○	○	○
11. Adakah anda mempunyai perasaan negatif yang kuat seperti takut/cemas, seram, marah, rasa bersalah, atau rasa malu?	○	○	○	○	○
12. Adakah anda hilang rasa minat kepada aktiviti yang pernah anda gemari?	○	○	○	○	○
13. Adakah anda terasa jauh atau terasing daripada orang lain?	○	○	○	○	○
14. Adakah anda sukar untuk merasakan perasaan positif (Contohnya, sukar atau tidak boleh rasa gembira atau perasaan sayang kepada orang terdekat)?	○	○	○	○	○
15. Adakah anda mudah marah, cepat sentap, atau bertindak ganas?	○	○	○	○	○
16. Adakah anda mengambil banyak risiko atau melakukan sesuatu yang boleh membahayakan diri anda?	○	○	○	○	○
17. Menjadi seorang yang sangat berwaspada atau berjaga-jaga?	○	○	○	○	○
18. Mudah rasa terkejut dan gelisah?	○	○	○	○	○
19. Adakah anda menghadapi masalah untuk menumpukan perhatian?	○	○	○	○	○
20. Adakah anda menghadapi masalah untuk tidur dan kekal tidur?	○	○	○	○	○

Tanveer S, *et al. BMJ Open* 2023; 13:e067886. doi: 10.1136/bmjopen-2022-067886

WHO-5

Untuk setiap kenyataan di bawah, sila nyatakan perasaan yang paling dirasai sejak dua minggu yang lepas. Dalam tempoh dua minggu yang lepas

	Sepanjang masa 5	Selalu 4	Seringkali (lebih daripada separuh masa) 3	Kadang-kadang (kurang daripada separuh masa) 2	Jarang 1	Tidak pernah 0
1. Saya berasa ceria dan bersemangat	○	○	○	○	○	○
2. Saya berasa tenang dan santai	○	○	○	○	○	○
3. Saya berasa aktif dan bertenaga	○	○	○	○	○	○
4. Saya berasa segar dan cukup rehat apabila bangun pagi	○	○	○	○	○	○
5. Kehidupan seharian saya dipenuhi dengan perkara yang saya minati	○	○	○	○	○	○

PTGI

Untuk setiap kenyataan di bawah, sila nyatakan sebanyak mana perubahan yang telah berlaku kepada hidup anda sejak peristiwa Covid-19, menggunakan skala di bawah.

	Tidak langsung 0	Sangat sedikit 1	Agak sedikit 2	Sederhana 3	Agak banyak 4	Sangat banyak 5
1. Saya ubah keutamaan terhadap apa yang penting dalam hidup ini.	○	○	○	○	○	○
2. Saya lebih menghargai nilai hidup saya sendiri.	○	○	○	○	○	○
3. Saya dapat memupuk minat yang baru.	○	○	○	○	○	○
4. Perasaan berdikari saya lebih meningkat.	○	○	○	○	○	○
5. Saya lebih memahami perkara kerohanian.	○	○	○	○	○	○
6. Saya dapat lihat yang saya boleh mengharapkan orang lain ketika saya di dalam kesusahan.	○	○	○	○	○	○
7. Saya memulakan perjalanan hidup yang baru.	○	○	○	○	○	○
8. Saya rasa lebih dekat dan akrab dengan orang lain.	○	○	○	○	○	○
9. Saya lebih bersedia untuk meluahkan emosi saya.	○	○	○	○	○	○
10. Saya yakin bahawa saya boleh melalui saat kesusahan.	○	○	○	○	○	○
11. Saya boleh melakukan perkara yang lebih bermanfaat dalam hidup saya.	○	○	○	○	○	○
12. Saya lebih senang menerima sebarang keputusan kepada perkara yang telah berlaku.	○	○	○	○	○	○

Tanveer S, *et al. BMJ Open* 2023; 13:e067886. doi: 10.1136/bmjopen-2022-067886

13. Saya dapat lebih bersyukur dan menghargai setiap hari yang ada.	○	○	○	○	○	○
14. Banyak peluang baru muncul yang sukar untuk didapatkan sebelum ini.	○	○	○	○	○	○
15. Rasa belas kasihan saya kepada orang lain meningkat.	○	○	○	○	○	○
16. Saya tingkatkan usaha saya dalam hubungan (bersama orang lain).	○	○	○	○	○	○
17. Saya lebih cenderung untuk mengubah perkara yang perlu diubah.	○	○	○	○	○	○
18. Kepercayaan kepada agama saya semakin meningkat.	○	○	○	○	○	○
19. Saya dapati yang saya lebih kuat daripada yang saya sangkakan.	○	○	○	○	○	○
20. Saya dapat belajar dan ketahui betapa baiknya orang-orang disekeliling saya.	○	○	○	○	○	○
21. Saya dapat terima dengan lebih baik bahawa saya memerlukan orang lain.	○	○	○	○	○	○

International Study – Somalia (Somali)

Sociodemographic Information

1. Jinsiga

- ○ Lab
- ○ Dheddig
- ○ Mid Kale
- ○ Ma jecli inaa ka hadlo

2. Da'daada (Sanad)

____

3. Xaalada guurka

- ○ Waligeey ma guursan
- ○ Qof ayaan xiriir la leeyahay
- ○ Waa guursaday
- ○ Kala maqanahay
- ○ Kala tagnay
- ○ Leeyga dhintay

4. Apa agama yang Anda?

- ○ Diin la'aan
- ○ Islam
- ○ Kristen
- ○ Hindu
- ○ Budha

- ○ Mid kale (Fadlan qeex) ____

5. Jinsiyadaada maxeey tahay? (waad sheegi kartaa wax ka badan hal)

____

6. Luqadee uugu hadashaa si aad u fiican nolol maalmeedkaada?

____

Labada su'aalo ee soo socda waxa laguugu weydiinayaa heerka luqadaada Ingiriisiga.
6a. Si fiican ayaan Ingirisiga u fahmayaa

- ○ Aad ayaan uuga soo horjeedaa1
- ○ Waan diidanahay 2
- ○ Mana kuugu raacsani mana diidani 3
- ○ Waan kuugu raacsanahay 4
- ○ Aad ayaan kuugu raacsanahay 5

6b. Si fiican ayaan Ingiriisiga uugu hadli karaa

- ○ Maya haba yaraatee 1
- ○ Kis yar 2
- ○ Kistoo yar 3
- ○ Xoogaa 4
- ○ In aad u badan 5

6. Waa maxay heerka kuugu sareeyo ee waxbarashada?

- ○ Ma heysto waxbarasho rasmi ah (waxaan ka qeybgalay dugsiga, fasalka ESOL)
- ○ Dugsiga sare (tusale ahaan NCEA,dibloomada IB, shahaadada dugsiga oo dibada)
- ○ Shahaadada heerka sadexaad (tusaale shahaado, Diblooma ama shahaadda ganacsiga - koorso 3 sano ka ayar)

○ Shahaadada bajalarka (koorsada 3-4 sanno)

○ Shahaadada jaamacada wixi ka sareeya bajalarka (dibloma PG, Mastarka, Dhaqtarnimada)

7. Heerka waxbarashada ama shaqadaada hada (Fadlan muuji mid kastoo ku quseya)

☐ Waxaan bartaa waqti buuxo

☐ Waxaan bartaa waqti barkii

☐ Baagamuudo

☐ Shaqo waqti buuxo (Full-time)

☐ Shaqo waqti barkii (Part-time)

☐ Iskii u shaqeysta waqti buuxa

☐ Full-time self employed

☐ Fasaxa waalidnimada (umusha) oo lacagteydana ii socoto

☐ Waalid guriga jooga (lacag la'aan)

☐ Ka fariistay

☐ Wax kale (fadlan noo sheeg) ____

8. Shaqada

____

9. Dakhliga qoyska ee bil walba (sarifka/lacagaha wadanakaada)

____

9a. Marka la isla barbardhigo dakhliga dhexdhexaadka (isku celceliska), sidee ayaad u qiimeyn lahayd dakhliga qoyskaada?

○ Wuu ka hooseeya dhexdhexaadka

○ Dhexdhexaad (Waa isku celcelis)

○ Wuu ka sareeyaa dhexdhexaadka

10. Miyaad hadda ku jirtaa bandoow ama laguugu amray in aad guriga joogtid?

○ Haa

○ Maya

11. Talaalka COVID-19 maqaadatay?

○ Haa

○ Maya

11a. Hadii aadan Willi is talaalin, ma jeclaan lahayd in aad is talaashid mustaqbalka?

○ Haa

○ Maya

12. Muuji baayaan walba ee soo socda heerka isbadal ee COVID-19 ku sababay noloshaada, adiga oo isticmaalaaya qiyaasta soo socoto. Halkan waxaa lagu soo koobay dhibaatooyinka ay dadka qaar kala kulmeen COVID-19: (Fadlan noo sheeg intaa kartid)

☐ Masiibo dabiici ah (sida fatahaada, dhulgariirka iwm)

☐ Ku noolaashada dhul dagaal kasocda ama goob jooga qilaafka militariga

☐ Caruurta ka yar 16 oo rafaad kusoo koray (sida dayacaada, xoogsheegsiga, dilka iyo fara xumeeyn)

☐ Dil ama kofsi 16 jir ka weyn

☐ Dhaawac weyn oo jirka

☐ Kuwa kale — fadlan Sii faahfaahi (hadii aad rabtid) ____

☐ Waxaan oo dhan ma aha

Covid Psychosocial Impacts Scale

Saameynta Shaqsiga

Bayaanka soo socda, fadlan kaga jawaab Haa/Maya haddii adiga kuugu dhacday arintaan Covid-19 dartiis. Hadday Haa tahay, fadlan sheeg xaddiga walaaca ay tani ku gaarsiisay adigoo hoos ku qayaaseysid lambarka ku habboon.

1. Ma qabtaa xaalad caafimaad oo kaa dhigi karta mid u nugul Covid-19?

- ○ **Maya** 0
- ○ **Haa** Walaac malahan haba yaraatee 1
- ○ **Haa** Walaac yar 2
- ○ **Haa** Walaac dhexdhexaad ah 3
- ○ **Haa** Walaac badan 4
- ○ **Haa** Walaac tiro badan 5

2. Maka cabsatay inaad la kulantid qof qaba Covid-19?

- ○ **Maya** 0
- ○ **Haa** Walaac malahan haba yaraatee 1
- ○ **Haa** Walaac yar 2
- ○ **Haa** Walaac dhexdhexaad ah 3
- ○ **Haa** Walaac badan 4
- ○ **Haa** Walaac tiro badan 5

3. Ma kulatahay in uu COVID-19 kuugu dhacay?

- ○ **Maya** 0
- ○ **Haa** Walaac malahan haba yaraatee 1
- ○ **Haa** Walaac yar 2
- ○ **Haa** Walaac dhexdhexaad ah 3

Tanveer S, *et al. BMJ Open* 2023; 13:e067886. doi: 10.1136/bmjopen-2022-067886

- **Haa** Walaac badan 4
- **Haa** Walaac tiro badan 5

3a. Waligaa malaaga helay COVID-19?

- **Maya** 0
- **Haa** Walaac malahan haba yaraatee 1
- **Haa** Walaac yar 2
- **Haa** Walaac dhexdhexaad ah 3
- **Haa** Walaac badan 4
- **Haa** Walaac tiro badan 5

3b. Miyaad leedahay astaamaha COVID-19 oo joogta ah?

- **Maya** 0
- **Haa** Walaac malahan haba yaraatee 1
- **Haa** Walaac yar 2
- **Haa** Walaac dhexdhexaad ah 3
- **Haa** Walaac badan 4
- **Haa** Walaac tiro badan 5

Sameeynta Qoyska

Bayaanka soo socda, fadlan kaga jawaab Haa/Maya haddii arintaan ku dhacday dadka qoyskaada Covid-19 dartiis. Hadday Haa tahay, fadlan sheeg xaddiga walaaca ay tani ku gaarsiisay adigoo hoos ku qayaaseysid lambarka ku habboon.

1. Qof ka mid ah qoyskaada ma jiraa oo u nugul Covid-19 maadaamo ey jirto xaalad caafimaad?

- **Maya** 0
- **Haa** Walaac malahan haba yaraatee 1

○ **Haa** Walaac yar 2

○ **Haa** Walaac dhexdhexaad ah 3

○ **Haa** Walaac badan 4

○ **Haa** Walaac tiro badan 5

2. Ma dareensantahay in qof qoyskaada ka mid ah halis u yahay inuu la kulmo qof qaba Covid-19 markuu shaqada joogo?

○ **Maya** 0

○ **Haa** Walaac malahan haba yaraatee 1

○ **Haa** Walaac yar 2

○ **Haa** Walaac dhexdhexaad ah 3

○ **Haa** Walaac badan 4

○ **Haa** Walaac tiro badan 5

3. Ma kulatahay in qof qoyskaada soke ka mid ah in uu ku dhacay COVID-19?

○ **Maya** 0

○ **Haa** Walaac malahan haba yaraatee 1

○ **Haa** Walaac yar 2

○ **Haa** Walaac dhexdhexaad ah 3

○ **Haa** Walaac badan 4

○ **Haa** Walaac tiro badan 5

3a. Ma jiraa qof qoyskaada soke ka mid ah oo laga helay COVID-19?

○ **Maya** 0

○ **Haa** Walaac malahan haba yaraatee 1

- **Haa** Walaac yar 2
- **Haa** Walaac dhexdhexaad ah 3
- **Haa** Walaac badan 4
- **Haa** Walaac tiro badan 5

3b. Majiraa qof qoyskaada soke ka mid ah ee leh astaamaha COVID-19 oo joogta ah?

- **Maya** 0
- **Haa** Walaac malahan haba yaraatee 1
- **Haa** Walaac yar 2
- **Haa** Walaac dhexdhexaad ah 3
- **Haa** Walaac badan 4
- **Haa** Walaac tiro badan 5

3c. Majiraa qof qoyskaada soke ah ee u geeriyooday/dhintay COVID-19?

- **Maya** 0
- **Haa** Walaac malahan haba yaraatee 1
- **Haa** Walaac yar 2
- **Haa** Walaac dhexdhexaad ah 3
- **Haa** Walaac badan 4
- **Haa** Walaac tiro badan 5

4. Majiraan dad qoyskaada soke ka mid ah ee dibada ku maqan oo aad uuga walwalsantahay in COVID-19 uu ku dhoco?

- **Maya** 0
- **Haa** Walaac malahan haba yaraatee 1

Tanveer S, *et al. BMJ Open* 2023; 13:e067886. doi: 10.1136/bmjopen-2022-067886

- **Haa** Walaac yar 2
- **Haa** Walaac dhexdhexaad ah 3
- **Haa** Walaac badan 4
- **Haa** Walaac tiro badan 5

5. Ma leedahay qoyska dhow dibedda ku sugan oo aadan awoodin inaad booqato ama aan kusoo booqan Karin adiga?

- **Maya** 0
- **Haa** Walaac malahan haba yaraatee 1
- **Haa** Walaac yar 2
- **Haa** Walaac dhexdhexaad ah 3
- **Haa** Walaac badan 4
- **Haa** Walaac tiro badan 5

Shaqo

Bayaanka soo socda, fadlan kaga jawaab Haa/Maya/Ma i qusayso haddii adiga ama qof kula nool ku dhacday Covid-19 dartiis. Hadday Haa tahay, fadlan sheeg xaddiga walaaca ay tani ku gaarsiisay adigoo hoos ku qayaaseysid lambarka ku habboon.

1. Ma idinkujiraa qoyskiina qof loo tixgaliyay in uu yahay shaqaalaha daruuriga (muhiimka ah) (shaqaalaha daryeelka caafimaadka, fulinta sharciga, adeegyada degdegga ah, kuwa bixiyaha adeegyada daruuriga ah)?

- **Maya** 0
- **Haa** Walaac malahan haba yaraatee 1
- **Haa** Walaac yar 2
- **Haa** Walaac dhexdhexaad ah 3
- **Haa** Walaac badan 4
- **Haa** Walaac tiro badan 5

2. Isbadal weyn ma ku yimid saacadaha aad adiga ama qof qoyskaada ka tirsan aad shaqeyn jirteen?

- **Maya** 0
- **Haa** Walaac malahan haba yaraatee 1
- **Haa** Walaac yar 2
- **Haa** Walaac dhexdhexaad ah 3
- **Haa** Walaac badan 4
- **Haa** Walaac tiro badan 5

3. Ma dhacday in aad adiga ama qof qoyskaada katirsan in aad ku qasbanaateen in goobtii shaqada isku badashay guriga (guriga adigoo joogta sii wadid shaqadii)?

- **Maya** 0
- **Haa** Walaac malahan haba yaraatee 1
- **Haa** Walaac yar 2
- **Haa** Walaac dhexdhexaad ah 3
- **Haa** Walaac badan 4
- **Haa** Walaac tiro badan 5

4. Majiraa qof qoyskaada ka mid ah ee ku qasbanaaday in uu shaqada badasho?

- **Maya** 0
- **Haa** Walaac malahan haba yaraatee 1
- **Haa** Walaac yar 2
- **Haa** Walaac dhexdhexaad ah 3
- **Haa** Walaac badan 4
- **Haa** Walaac tiro badan 5

Tanveer S, *et al. BMJ Open* 2023; 13:e067886. doi: 10.1136/bmjopen-2022-067886

5. Majiraa qof qoykaada ka tirsan ee shaqadiisii waayay?

- **Ma i qusayso**
- **Maya** 0
- **Haa** Walaac malahan haba yaraatee 1
- **Haa** Walaac yar 2
- **Haa** Walaac dhexdhexaad ah 3
- **Haa** Walaac badan 4
- **Haa** Walaac tiro badan 5

6. Ganacsi/bisinis aad adiga ama qof kula nool lahaa am aad ka shaqeeyneeyseen miyaa halis u ah inuu sii socon waayo?

- **Ma i qusayso**
- **Maya** 0
- **Haa** Walaac malahan haba yaraatee 1
- **Haa** Walaac yar 2
- **Haa** Walaac dhexdhexaad ah 3
- **Haa** Walaac badan 4
- **Haa** Walaac tiro badan 5

Dakhliga

Bayaanka soo socda, fadlan kaga jawaab Haa/Maya haddii uu qoyskaada quseeya Covid-19 dartiis. Hadday Haa tahay, fadlan sheeg xaddiga walaaca ay tani ku gaarsiisay adigoo hoos ku qayaaseysid lambarka ku habboon.

1. Dhaqaalaha reerkaada aad miyuu hoos u dhacay?

- **Maya** 0
- **Haa** Walaac malahan haba yaraatee 1

- **Haa** Walaac yar 2
- **Haa** Walaac dhexdhexaad ah 3
- **Haa** Walaac badan 4
- **Haa** Walaac tiro badan 5

2. Qoyskiina miyaad awoodi wayseen inaad bixisaan biilasha ama adeega cuntada?

- **Maya** 0
- **Haa** Walaac malahan haba yaraatee 1
- **Haa** Walaac yar 2
- **Haa** Walaac dhexdhexaad ah 3
- **Haa** Walaac badan 4
- **Haa** Walaac tiro badan 5

Xiriika

Bayaanka soo socda, fadlan kaga jawaab Haa/Maya haddii adiga kuugu dhacday arintaan Covid-19 dartiis. Hadday Haa tahay, fadlan sheeg xaddiga walaaca ay tani ku gaarsiisay adigoo hoos ku qayaaseysid lambarka ku habboon. Covid-19 dartiis.....

1. Dhibaatada qoyska miyeey sii adkaatay?

- **Maya** 0
- **Haa** Walaac malahan haba yaraatee 1
- **Haa** Walaac yar 2
- **Haa** Walaac dhexdhexaad ah 3
- **Haa** Walaac badan 4
- **Haa** Walaac tiro badan 5

2. Xiriirkii aad la lahayd asxaabtaada miyuu soo yaraaday (si toos ah ama dhinaca qadka)?

- **Maya** 0
- **Haa** Walaac malahan haba yaraatee 1
- **Haa** Walaac yar 2
- **Haa** Walaac dhexdhexaad ah 3
- **Haa** Walaac badan 4
- **Haa** Walaac tiro badan 5

Isbeddelka ku yimid nolol maalmeedka iyo dabeecadaha

Bayaanka soo socda, fadlan kaga jawaab Haa/Maya haddii adiga kuugu dhacday arintaan Covid-19 dartiis. Hadday Haa tahay, fadlan sheeg xaddiga walaaca ay tani ku gaarsiisay adigoo hoos ku qayaaseysid lambarka ku habboon. Covid-19 dartiis…..

1. Cabuqa Korona (Covid-19) miyuu isbadal ku keenay nolol maalmeedkaada (sida hurdada, jimicsiga, firaaqada, hiwaayadaha aad xiiseeysid)?

- **Maya** 0
- **Haa** Walaac malahan haba yaraatee 1
- **Haa** Walaac yar 2
- **Haa** Walaac dhexdhexaad ah 3
- **Haa** Walaac badan 4
- **Haa** Walaac tiro badan 5

2. Miyay taasi sababtay inaad dib u dhigto ama iska dhaaftid inaad raadsato daryeel caafimaad mar ad adiga ama qof qoyskaada u baahateen?

- **Maya** 0
- **Haa** Walaac malahan haba yaraatee 1
- **Haa** Walaac yar 2
- **Haa** Walaac dhexdhexaad ah 3

- **Haa** Walaac badan 4
- **Haa** Walaac tiro badan 5

3. Tani miyay yareeysay ka-qayb-qaadashadaada howlaha diinta (shaqsi ahaan ama khadka tooska ah)?

- **Ma i qusayso**
- **Maya** 0
- **Haa** Walaac malahan haba yaraatee 1
- **Haa** Walaac yar 2
- **Haa** Walaac dhexdhexaad ah 3
- **Haa** Walaac badan 4
- **Haa** Walaac tiro badan 5

4. Miyey jireen isbeddelo kale oo weyn oo ku yimid nidaamka/nolol maalmeedka?

- **Maya** 0
- **Haa (Haddii ay sidaas tahay fadlan sheeg)** 1 ____

Hadday Haa tahay, fadlan sheeg xaddiga walaaca ay tani ku gaarsiisay adigoo hoos ku qayaaseysid lambarka ku habboon.

- Walaac malahan haba yaraatee 1
- Walaac yar 2
- Walaac dhexdhexaad ah 3
- Walaac badan 4
- Walaac tiro badan 5

Soo bandhigista macluumaadka ku saabsan Covid-19

1. Xageed kaheshaa warbixinta ku saabsan Covid-19?
(Fadlan qeex mid kasto ad isticmaashid)

- ☐ News/wararka
- ☐ Facebook / Twitter
- ☐ Websites/boga caafimaadka
- ☐ Hadal isgaarsiis
- ☐ Meela kale (Fadlan qeex) ____

2. Culeys ilaa intee ayuu wararka ku saabsan Covid-19 kuu geystay?

- ○ Walaac malahan haba yaraatee 1
- ○ Walaac yar 2
- ○ Walaac dhexdhexaad ah 3
- ○ Walaac badan 4
- ○ Walaac tiro badan 5

Isbeddelada ku yimid Covid-19 dartiis

Arimaha soo socda fadlan sheeg xaddiga walaaca ay tani ku gaarsiisay adigoo hoos ku qayaaseysid lambarka ku habboon. Isbarbardhiga hadda iyo wixii ka horeeyay Covid-19---

1. Sideed u qiimeyn lahayd heerka walaacaaga shaqsi ahaan?

- ○ Walaac yar 1
- ○ Xoogaa walaac yar 2
- ○ Islameeshii 3
- ○ Walwal batay 4
- ○ Walaaca oo aad u batay 5

2. Maxaad ku qiimeyn lahayd walaaca guud ee reerkaada/gurigaada?

- ○ Walaac yar 1

- Xoogaa walaac yar 2
- Islameeshii 3
- Walwal batay 4
- Walaaca oo aad u batay 5

3. Sideed u qiimeyn lahayd ladnaantaada nafsiyeed?

- Aad uuga fiican sidii hore 1
- Sidii hore ka fiican 2
- Sideedii 3
- Sidii ka daran 4
- Aad uga daran 5

4. Sideed u qiimeyn laheyd wanaagsanaanta nafsiyeed ee dadka qoyskaaga?

- Aad ufiican 1
- Fiican 2
- Ku saabsan isla mid 3
- Ka xun 4
- Aad uga xun 5

Tanveer S, *et al. BMJ Open* 2023; 13:e067886. doi: 10.1136/bmjopen-2022-067886

K-10

Tobanka su'aalo ee xiggo waxay wax ka weydiinayaan sida aad dareemeysay 4 isbuuc ee la soo dhaafay. Su'aal walba, tilmaam intajeer aad dareentay qaabkaas.

	Marnaba 1	Mar dhif 2	Mar mar 3	Inta badan 4	Mar kasta 5
1. Afartii isbuuc ee la soo dhaafay, intee jeer ayaad dareentay daal oo aan lahayn sabab wanaagsan?	○	○	○	○	○
2. Afartii isbuuc ee la soo dhaafay, intee jeer ayaad dareentay walwal?	○	○	○	○	○
3. Afartii isbuuc ee la soo dhaafay, intee jeer ayaad dareentay walwal zaa'id ah oo waxba aanan ku dajineyn?	○	○	○	○	○
4. Afartii isbuuc ee la soo dhaafay, intee jeer ayaad dareentay rajo la' aan?	○	○	○	○	○
5. Afartii isbuuc ee la soo dhaafay, intee jeer ayaad dareentay nasasho la'aan ama samir la'aan?	○	○	○	○	○
6. Afartii isbuuc ee la soo dhaafay, intee jeer ayaad dareentay nasasho la'aan oo aadan fadhin karin?	○	○	○	○	○
7. Afartii isbuuc ee la soo dhaafay, intee jeer ayaad dareentay murugo?	○	○	○	○	○
8. Afartii isbuuc ee la soo dhaafay, intee jeer ayaad dareentay in wax walba ay dadaal/halgan ahaayeen?	○	○	○	○	○
9. Afartii isbuuc ee la soo dhaafay, intee jeer ayaad dareentay murugo badan oo waxba aanan ku farxad gelineynin?	○	○	○	○	○
10. Afartii isbuuc ee la soo dhaafay, intee jeer ayaad dareentay qiimo la'aan?	○	○	○	○	○

Tanveer S, *et al. BMJ Open* 2023; 13:e067886. doi: 10.1136/bmjopen-2022-067886

PCL-5

Hoos waa liiska dhibaatooyinka oo dadka mararka qaar ay qabaan oo jawaab u ah waaya aragnimada walbahaarka badan. Fadlan si taxadar ah u aqri dhibaato walba kadibna tilmaam inta ay ku dhibtay dhibaatadaas bishii la soo dhaafay. Bisha la soo dhaafay, intee ayay ku dhibtay:

	**Maya gabi ahan** 0	**Xooga yar** 1	**Dhexaad** 2	**Ilaa xad** 3	**Xad dhaaf ah** 4
1. Soo nonoqoshada xasuusta aan la rabin oo khibradaha argagaxa leh?	○	○	○	○	○
2. Soo nonoqoshada riyooyinka jahwareerka ah ee ku saabsan khibradaha argagaxa leh?	○	○	○	○	○
3. Dareen lama filaan ah ama u dhaqanka sidii ineey hada khibradii murugada lahayd dhaceysa markale (sidii ineey hada dhaceeyso oo kale)?	○	○	○	○	○
4. Dareenka murugada aadka ah markii wax ku soo xasuusiyaan waaya aragnimada murugada leh?	○	○	○	○	○
5. Falcelinada jirka xoogan markii wax ku soo xasuusiyaan waaya aragnimadii murugada lahyd (tusaale ahaan, garaaca wadnaha, neefsashada oo dhib noqota iyo dhididka)?	○	○	○	○	○
6. Ka fogaanshaha xasuusta, afkaaraha, ama dareemida la xiriirto waaya aragnimada/khibradaha murugada leh?	○	○	○	○	○
7. Ka fogaanshaha wax kastoo kusoo xasuusiya walbahaarkii (tusaala ahaan, dadka, meelaha, wadasheekeysiga, shaqooyinka, waxyaabaha ama xaaladaha)?	○	○	○	○	○
8. Miyaad dhib ku qabtaa inaa xasuusatid qeyba muhiim ah ee khibradihii walbahaarka lahaa?	○	○	○	○	○
9. Ka qabida aaminaad xun oo xoogan nafsadaada, dadka kale, ama caalamka (tusaale ahaan, lahaanshaha afkaaraha sida; Waan xummahay, wax baa iga qaldan,	○	○	○	○	○

Tanveer S, *et al. BMJ Open* 2023; 13:e067886. doi: 10.1136/bmjopen-2022-067886

majiro qof la aamini karo, caalamka oo dhan waa halis)?

10. Ku eedeynta nafsadaada ama qof kale ee khibradihii walbahaarka lahaa ama wixii dhacay markaas kadib?

11. Lahaanshaha dareen xun ee xoogan sida cabsida, naxdinta, xanaaqa, dambiilenimada, ama ceeb?

12. Ka xiisadhicida howlihii aad jeclaan jirtay?

13. Dareemida ka fogaanshaha ama ka go'ida dadka kale?

14. Dhibaato ma ku qabtaa inaad dareento dareen wanaagsan (tusaale ahaan, inaad dareento farxad ama aad jeclaato dadka kuu dhow)?

15. Anshax xumo, xanaaqa, ama ku dhaqmida si dagaal ah?

16. Halis is galis badan ama sameeynta waxyaabaha kuugu sababi karo dhibaato?

17. Ma dareentaa "feejignaan cajiib ah" ama fiirin badan ama illaalo?

18. Miyaad dareentaa xasilaad la'aan ama si sahlan inaad u naxdo?

19. Ma kuugu adagtahay inaad diirad saartid/xogsaartid?

20. Ma kuugu adagtahay ineey hurdo kaa soo dhacdo ama inaad sii hurudo?

WHO-5

Fadlan noo tilmaam shanta arimood ee soo socda sida ugu dhow uu dareenkaaga ahaa labadii isbuuc ee la soo dhaafay. Labadii isbuuc ee lasoo dhaafay

	**Marwalba** 5	**Intabadan wakhtiga** 4	**Inkabadan kalabar wakhtiga** 3	**Inkayar kalabar wakhtiga** 2	**Marmar wakhtiga** 1	**Marnaba** 0
1. Waxaan dareemay farxad iyo hami sare.	○	○	○	○	○	○
2. Waxaan dareemay daganaan iyo xasilooni.	○	○	○	○	○	○
3. Waxaan dareemay firfircooni iyo awoodbadan.	○	○	○	○	○	○
4. Waxaan soo kacay anigoo dareemaya nasasho iyo daganaan.	○	○	○	○	○	○
5. Maalintayda oo dhan waxaa buuxiyay waxyalo ixiiso galiya.	○	○	○	○	○	○

Tanveer S, *et al. BMJ Open* 2023; 13:e067886. doi: 10.1136/bmjopen-2022-067886

PTGI

Muuji baayaan walba ee soo socda heerka isbadal ee Maarso 15-keedii ku sababay noloshaada, adoo isticmaalaaya qiyaasta soo socoto.

	Marnaba 0	Wax yar 1	Xoogaa Roon 2	Dhexaad 3	In badan 4	Wax badan 5
1. Waxaan badelay muhiimadeyda ku saabsan waxa nolosha muhiim u ah.	○	○	○	○	○	○
2. Waxaan qadarin weyn u leeyahay qiimaha noloshayda.	○	○	○	○	○	○
3. Waxaan hormariyay xiiso/dana cusub.	○	○	○	○	○	○
4. Waxaan dareemayaa isku kalsooni.	○	○	○	○	○	○
5. Waxaan si fiican uu fahamsanahay arrimaha diinta.	○	○	○	○	○	○
6. Waxaan si cad u arkaa inaan ku xisaabtami karo dadka waqtiyada dhibaatada.	○	○	○	○	○	○
7. Waxaan nolosheyda u aasaasay wado cusub.	○	○	○	○	○	○
8. Waxaan dareensanahay inaan dadka aad uugu dhawahay.	○	○	○	○	○	○
9. Waxaan diyaar u ahay inaan muujiyo dareenkeyga.	○	○	○	○	○	○
10. Waxaan si fiican u ogahay in aan wax ka qaban karo dhibaatoyinka.	○	○	○	○	○	○
11. Waxaan awooda in aan noloshayda wax fiican ku sameeyo.	○	○	○	○	○	○
12. Waxaan si fiican u awoodaa inaan aqbalo siday wax u dhacayaan.	○	○	○	○	○	○

13. Waxaan si fiican uga mahadcelin karaa maalin kasta.	○	○	○	○	○	○
14. Fursado cusub ayaa la heli karaa oo awal jirin.	○	○	○	○	○	○
15. Waxaan u qabaa naxariis badan dadka kale.	○	○	○	○	○	○
16. Waxaan dadaal badan geliya cilaaqaadkeyga.	○	○	○	○	○	○
17. Waxay u badan tahay inaan isku dayo inaan beddelo waxyaabaha u baahan badal.	○	○	○	○	○	○
18. Waxaan leeyahay caqiido diineed oo xoog badan.	○	○	○	○	○	○
19. Waxaan ogaaday inaan ka xoog badanahay sidaan u maleeynayay.	○	○	○	○	○	○
20. Waxaan bartay ineey dadka aad u wanaagsan yihiin.	○	○	○	○	○	○
21. Waxaan si fiican u qirraa inaan u baahankaro dadka kale.	○	○	○	○	○	○

International Study - Turkiye (Turkish)

Sociodemographic Information

1. Cinsiyet

- ○ Erkek
- ○ Kadın

2. Yaş

____

3. Medeni Hali

- ○ Bekar
- ○ İlişkisi var
- ○ Evli
- ○ Ayrılmış
- ○ Boşanmış
- ○ Dul

4. Dininiz nedir?

- ○ Din yok
- ○ İslâm
- ○ Hıristiyanlık
- ○ Hinduizm
- ○ Budizm
- ○ Diğer (lütfen belirtiniz) ________________________________

Tanveer S, *et al. BMJ Open* 2023; 13:e067886. doi: 10.1136/bmjopen-2022-067886

5. Eğitim düzeyiz nedir?(En son almış olduğunuz derece)?

- Okur Yazar
- İlkokul Mezunu
- Ortaokul Mezunu
- Lise Mezunu
- Üniversite Öğrencisi
- Önlisans Mezunu
- Lisans Mezunu
- Yüksek Lisans Mezunu
- Doktora Mezunu

6. Mevcut Çalışma veya iş durumunuz (Birden fazla işaretleyebilirsiniz.)

- [ ] Öğrenci
- [ ] Çalışmıyor/İş Arıyor
- [ ] Tam zamanlı maaşlı çalışma
- [ ] Yarı zamanlı maaşlı çalışma
- [ ] Tam zamanlı serbest meslek
- [ ] Yarı zamanlı serbest meslek
- [ ] Ücretli ebeveyn izninde
- [ ] Evde ebeveyn yada ücretsiz ebeveyn izninde
- [ ] Emekli
- [ ] Diğer (Lütfen Belirtin) ____

7. Mesleğiniz

____

8. Aylık hane geliri (yerel para biriminde)

____

9. Ortalama gelirle karşılaştırıldığında, hanenizin gelirini nasıl değerlendirirsiniz?

○ Ortalamanın altında

○ Ortalama

○ Ortalamanın üstünde

10. Şu anda karantinada ya da sokağa çıkma yasağında mısınız?

○ Evet1

○ Hayır 0

11. Covid-19 aşısı oldunuz mu?

○ Evet1

○ Hayır 0

11a. Aşı olmadıysanız, gelecekte Covid-19 aşısı olmak ister misiniz?

○ Evet1

○ Hayır 0

12. Bazı insanların geçmişte maruz kaldıkları diğer travmatik olayların yeni sorunlarla başa çıkmayı kolaylaştırabilecek ya da zorlaştırabilecek olduğunun farkındayız.

Covid-19 salgınından önce aşağıdakilerden herhangi birine şahit oldunuz mu veya herhangi birini yaşadınız mı?: **(Lütfen uygun olanları işaretleyin)**

☐ Doğal afet (sel, deprem, vs.)

- ☐ Savaş bölgesinde yaşamak veya askeri çatışmaya maruz kalmak
- ☐ 16 yaş öncesi çocukluk sıkıntısı (mesela ihmal, zorbalık, fiziksel veya cinsel saldırı, vs.)
- ☐ 16 yaş sonrası fiziksel veya cinsel saldırı
- ☐ Ciddi fiziksel kazalar
- ☐ Diğer – isteğe bağlı – bunun ne olduğunu açıklayabilir misiniz lütfen?

____

- ☐ Yukarıdakilerin hiçbiri

Tanveer S, *et al. BMJ Open* 2023; 13:e067886. doi: 10.1136/bmjopen-2022-067886

Covid Psychosocial Impacts Scale

Kişisel etki

Aşağıdaki ifadeler için, Covid-19 döneminde sizde böyle bir durum gerçekleştiyse lütfen Evet / Hayır ile yanıt verin. Evet ise, lütfen aşağıdaki ölçekte bunun sizde neden olduğu stres miktarını belirtin.

1. Covid-19'dan daha olumsuz etkilenmenize neden olabilecek bir sağlık probleminiz var mı (örn. Kalp hastalığı, nefes almada zorluk, zayıf bağışıklık sistemi, veya kanser)?

- ○ **Hayır** 0
- ○ **Evet** Hiç stresli değil 1
- ○ **Evet** Biraz stres 2
- ○ **Evet** Orta derecede stres 3
- ○ **Evet** Oldukça fazla stres 4
- ○ **Evet** Aşırı derecede stres 5

2. Covid-19 pozitif biriyle temas riski altında olduğunuzu hissettiniz mi?

- ○ **Hayır** 0
- ○ **Evet** Hiç stresli değil 1
- ○ **Evet** Biraz stres 2
- ○ **Evet** Orta derecede stres 3
- ○ **Evet** Oldukça Fazla stres 4
- ○ **Evet** Aşırı derecede stres 5

3. Covid-19 geçirdiğinizi düşünüyor musunuz?

- ○ **Hayır** 0
- ○ **Evet** Hiç stresli değil 1
- ○ **Evet** Biraz stres 2

Tanveer S, *et al. BMJ Open* 2023; 13:e067886. doi: 10.1136/bmjopen-2022-067886

- **Evet** Orta derecede stres 3
- **Evet** Oldukça fazla stres 4
- **Evet** Aşırı derecede stres 5

3a. Covid-19 tanısı aldınız mı?

- **Hayır** 0
- **Evet** Hiç stresli değil 1
- **Evet** Biraz stres 2
- **Evet** Orta derecede stres 3
- **Evet** Oldukça fazla stres 4
- **Evet** Aşırı derecede stres 5

3b. Devam eden Covid-19 semptomlarınız var mı?

- **Hayır** 0
- **Evet** Hiç stresli değil 1
- **Evet** Biraz stres 2
- **Evet** Orta derecede stres 3
- **Evet** Oldukça fazla stres 4
- **Evet** Aşırı derecede stres 5

Aile etkisi

Aşağıdaki ifadeler için, Covid-19 döneminde yakın aile üyelerinizde böyle bir durum gerçekleştiyse lütfen Evet / Hayır ile yanıt verin. Evet ise, lütfen aşağıdaki ölçekte bunun sizde neden olduğu stres miktarını belirtin.

1. Herhangi bir yakın aile üyesinin Covid-19'dan daha olumsuz etkilenmelerine neden olabilecek bir sağlık problemi var mı? (örn. Kalp hastalığı, nefes almada zorluk, zayıf bağışıklık sistemi, veya kanser)?

- **Hayır** 0
- **Evet** Hiç stresli değil 1
- **Evet** Biraz stres 2
- **Evet** Orta derecede stres 3
- **Evet** Oldukça fazla stres 4
- **Evet** Aşırı derecede stres 5

2. Pandemi sürecinde herhangi bir yakın aile üyesinin, Covid-19 pozitif olan biri ile temas riski altında olduğunu düşündünüz mü/düşünüyor musunuz?

- **Hayır** 0
- **Evet** Hiç stresli değil 1
- **Evet** Biraz stres 2
- **Evet** Orta derecede stres 3
- **Evet** Oldukça fazla stres 4
- **Evet** Aşırı derecede stres 5

3. Yakın aile üyelerinizden herhangi birinin Covid-19 geçirdiğini düşünüyor musunuz?

- **Hayır** 0
- **Evet** Hiç stresli değil 1
- **Evet** Biraz stres 2
- **Evet** Orta derecede stres 3
- **Evet** Oldukça fazla stres 4
- **Evet** Aşırı derecede stres 5

3a. Yakın aile üyelerinin herhangi biri Covid-19 tanısı aldı mı?

- ○ **Hayır** 0
- ○ **Evet** Hiç stresli değil 1
- ○ **Evet** Biraz stres 2
- ○ **Evet** Orta derecede stres 3
- ○ **Evet** Oldukça fazla stres 4
- ○ **Evet** Aşırı derecede stres 5

3b. Yakın aile üyesi/üyelerinde devam eden Covid-19 semptomları var mı?

- ○ **Hayır** 0
- ○ **Evet** Hiç stresli değil 1
- ○ **Evet** Biraz stres 2
- ○ **Evet** Orta derecede stres 3
- ○ **Evet** Oldukça fazla stres 4
- ○ **Evet** Aşırı derecede stres 5

3c. Yakın bir aile üyesi/üyeleri Covid-19 nedeniyle vefat etti mi?

- ○ **Hayır** 0
- ○ **Evet** Hiç stresli değil 1
- ○ **Evet** Biraz stres 2
- ○ **Evet** Orta derecede stres 3
- ○ **Evet** Oldukça fazla stres 4
- ○ **Evet** Aşırı derecede stres 5

4. Yurtdışında Covid-19'a yakalanma riski altında olmasından endişe duyduğunuz yakın aile üyeniz var mı?

- **Hayır** 0
- **Evet** Hiç stresli değil 1
- **Evet** Biraz stres 2
- **Evet** Orta derecede stres 3
- **Evet** Oldukça fazla stres 4
- **Evet** Aşırı derecede stres 5

5. Covid-19 pandemisi nedeniyle yurtdışında ziyaret edemediğiniz veya sizi ziyaret edemeyen yakın aile üyeniz var mı?

- **Hayır** 0
- **Evet** Hiç stresli değil 1
- **Evet** Biraz stres 2
- **Evet** Orta derecede stres 3
- **Evet** Oldukça fazla stres 4
- **Evet** Aşırı derecede stres 5

İş

Aşağıdaki ifadeler için, Covid-19 döneminde bu durum sizde veya evinizdeki herhangi birinde gerçekleşmişse (Evet / Hayır / Bana uygun değil) ile yanıt verin. Evet ise, lütfen aşağıdaki ölçekte bunun sizde neden olduğu stres miktarını belirtin.

1. Siz veya evinizdeki herhangi biri temel sektör çalışanı mısınız? (örneğin sağlık hizmetleri, gıda sektörü, acil servisler, temel mal / hizmet sağlayıcıları vb.)?

- **Hayır** 0
- **Evet** Hiç stresli değil 1

- **Evet** Biraz stres 2
- **Evet** Orta derecede stres 3
- **Evet** Oldukça fazla stres 4
- **Evet** Aşırı derecede stres 5

2. Covid-19 pandemisi nedeniyle sizin veya evinizdeki herhangi birinin çalışma saatlerinde büyük bir değişiklik oldu mu?

- **Hayır** 0
- **Evet** Hiç stresli değil 1
- **Evet** Biraz stres 2
- **Evet** Orta derecede stres 3
- **Evet** Oldukça fazla stres 4
- **Evet** Aşırı derecede stres 5

3. Covid-19 pandemisi nedeniyle siz veya evinizdeki herhangi biri evden çalışmak zorunda kaldınız mı?

- **Hayır** 0
- **Evet** Hiç stresli değil 1
- **Evet** Biraz stres 2
- **Evet** Orta derecede stres 3
- **Evet** Oldukça fazla stres 4
- **Evet** Aşırı derecede stres 5

4. Covid-19 pandemisi nedeniyle siz veya evinizdeki herhangi biri farklı bir iş türüne geçmek zorunda kaldınız mı?

- **Hayır** 0

- **Evet** Hiç stresli değil 1
- **Evet** Biraz stres 2
- **Evet** Orta derecede stres 3
- **Evet** Oldukça fazla stres 4
- **Evet** Aşırı derecede stres 5

5. Covid-19 pandemisi nedeniyle siz veya evinizdeki herhangi biri işini kaybetti mi?

- **Bana uygun değil**
- **Hayır** 0
- **Evet** Hiç stresli değil 1
- **Evet** Biraz stres 2
- **Evet** Orta derecede stres 3
- **Evet** Oldukça fazla stres 4
- **Evet** Aşırı derecede stres 5

6. Covid-19 pandemisi nedeniyle sizin veya evinizdeki bir başkasının kendi işletmesi veya çalıştığı şirket kapanma tehdidi altında mı?

- **Bana uygun değil**
- **Hayır** 0
- **Evet** Hiç stresli değil 1
- **Evet** Biraz stres 2
- **Evet** Orta derecede stres 3
- **Evet** Oldukça fazla stres 4

Tanveer S, *et al. BMJ Open* 2023; 13:e067886. doi: 10.1136/bmjopen-2022-067886

- **Evet** Aşırı derece stres 5

Gelir

Aşağıdaki ifadeler için, Covid-19 döneminde bu durum evinizdeki herhangi birinde gerçekleştiyse (Evet / Hayır) ile yanıt verin. Evet ise, lütfen aşağıdaki ölçekte bunun sizde neden olduğu stres miktarını belirtin.

1. Covid-19 pandemisi nedeniyle hanenizde büyük bir maddi zorluk yaşadınız mı?

- **Hayır** 0
- **Evet** Hiç stresli değil 1
- **Evet** Biraz stres 2
- **Evet** Orta derecede stres 3
- **Evet** Oldukça fazla stres 4
- **Evet** Aşırı derecede stres 5

2. Covid-19 pandemisi nedeniyle hanehalkınız faturalarınızı ödeyebilmeniz veya mutfak masraflarınızı karşılayabilmeniz konusunda büyük bir zorluk yaşadı mı?

- **Hayır** 0
- **Evet** Hiç stresli değil 1
- **Evet** Biraz stres 2
- **Evet** Orta derecede stres 3
- **Evet** Oldukça fazla stres 4
- **Evet** Aşırı derecede stres 5

İlişkiler

Aşağıdaki ifadeler için, Covid-19 döneminde sizde böyle bir durum gerçekleştiyse lütfen Evet / Hayır ile yanıt verin. Evet ise, lütfen aşağıdaki ölçekte bunun sizde neden olduğu stres miktarını belirtin. Covid-19'a yanıt olarak....

1. Covid-19 döneminde aile üyeleri arasında zorluklar ve gerginlikler arttı mı?

- **Hayır** 0
- **Evet** Hiç stresli değil 1
- **Evet** Biraz stres 2
- **Evet** Orta derecede stres 3
- **Evet** Oldukça fazla stres 4
- **Evet** Aşırı derecede stres 5

2. Covid-19 pandemisi arkadaşlarınızla (fiziksel veya çevrimiçi/online) iletişiminizi azalttı mı?

- **Hayır** 0
- **Evet** Hiç stresli değil 1
- **Evet** Biraz stres 2
- **Evet** Orta derecede stres 3
- **Evet** Oldukça fazla stres 4
- **Evet** Aşırı derecede stres 5

Günlük rutin ve davranışlarda değişiklikler

Aşağıdaki ifadeler için, Covid-19 döneminde sizde böyle bir durum gerçekleştiyse lütfen (Evet / Hayır / Bana uygun değil) ile yanıt verin. Evet ise, lütfen aşağıdaki ölçekte bunun sizde neden olduğu stres miktarını belirtin. Covid-19'a yanıt olarak......

1. Covid-19 pandemisi günlük rutininizde değişikliklere yol açtı mı? (örneğin, uyku, egzersiz, boş zaman, keyif veren aktiviteler veya hobiler)

- **Hayır** 0
- **Evet** Hiç stresli değil 1
- **Evet** Biraz stres 2

○ **Evet** Orta derecede stres 3

○ **Evet** Oldukça fazla stres 4

○ **Evet** Aşırı derece stres 5

2. Covid-19 pandemisi sizin veya bir aile üyenizin ihtiyaç duyduğu sağlık hizmetine başvurmayı ertelemenize / başvurmaktan kaçınmanıza neden oldu mu?

○ **Hayır** 0

○ **Evet** Hiç stresli değil 1

○ **Evet** Biraz stres 2

○ **Evet** Orta derecede stres 3

○ **Evet** Oldukça fazla stres 4

○ **Evet** Aşırı derecede stres 5

3. Covid-19 pandemisi dini faaliyetlere katılımınızı azalttı mı? (yüz yüze veya çevrimiçi/online)

○ **Bana uygun değil**

○ **Hayır** 0

○ **Evet** Hiç stresli değil 1

○ **Evet** Biraz stres 2

○ **Evet** Orta derecede stres 3

○ **Evet** Oldukça fazla stres 4

○ **Evet** Aşırı derecede stres 5

4. Covid-19 pandemisi nedeniyle rutininizde başka büyük değişiklikler oldu mu?

○ **Hayır** 0

Tanveer S, *et al. BMJ Open* 2023; 13:e067886. doi: 10.1136/bmjopen-2022-067886

- ◯ Evet ise lütfen belirtin1 ____

Evet ise, lütfen bunun aşağıdaki ölçekte sizde neden olduğu stres miktarını belirtin.

- ◯ Hiç stresli değil 1
- ◯ Biraz stres 2
- ◯ Orta derecede stres 3
- ◯ Oldukça fazla stres 4
- ◯ Aşırı derecede stres 5

Covid-19 hakkındaki bilgilere maruz kalma

1. Covid-19 ile ilgili bilgi almak için hangi kaynakları kullanıyorsunuz? (Lütfen uygun olanları işaretleyin.)

- ☐ Haberler
- ☐ Facebook / Twitter
- ☐ Sağlık web siteleri
- ☐ Duyum/Başkalarından duyma
- ☐ Diğerleri (lütfen belirtiniz) ____

2. Covid-19 ile ilgili haberler sizde ne kadar strese neden oluyor?

- ◯ Hiç stresli değil 1
- ◯ Biraz stres 2
- ◯ Orta derecede stres 3
- ◯ Oldukça fazla stres 4
- ◯ Aşırı derecede stres 5

Covid-19'dan bu yana değişiklikler

Aşağıdaki ifadeler için size uygun olanı lütfen ilgili ölçekte belirtiniz. Şimdi ile Covid-19 salgını öncesini karşılaştırma...

1. Covid-19 pandemisi öncesi ile kıyasladığınızda salgın sonrası kişisel stres seviyenizi nasıl değerlendirirsiniz?

- ○ Önemli ölçüde daha az stresli 1
- ○ Biraz daha az stresli 2
- ○ Aynı sayılır 3
- ○ Daha stresli 4
- ○ Önemli ölçüde daha fazla stresli 5

2. Covid-19 pandemisi öncesi ile kıyasladığınızda salgın sonrası evinizdeki genel stres düzeyini nasıl değerlendirirsiniz?

- ○ Önemli ölçüde daha az stresli 1
- ○ Biraz daha az stresli 2
- ○ Aynı sayılır 3
- ○ Daha stresli 4
- ○ Önemli ölçüde daha fazla stresli 5

3. Covid-19 pandemisi öncesi ile kıyasladığınızda salgın sonrası kişisel psikolojik iyilik halinizi nasıl değerlendirirsiniz?

- ○ Çok daha iyi 1
- ○ Daha iyi 2
- ○ Aynı sayılır 3
- ○ Daha kötü 4

- Çok daha kötü 5

4. Covid-19 pandemisi öncesi ile kıyasladığınızda salgın sonrası hanehalkı üyelerinizin genel psikolojik iyilik halini nasıl değerlendirirsiniz?

- Çok daha iyi 1
- Daha iyi 2
- Ayni sayilir 3
- Daha kötü 4
- Çok daha kötü 5

K-10

Aşağıdaki 10 soru son 4 hafta içinde kendinizi nasıl hissettiğinizle ilgilidir. Her soru için, kendinizi o durumda hissettiğiniz zamanın uzunluğunu en iyi şekilde tanımlayan seçeneği seçiniz.

	**Hiçbir zaman 1**	**Kısa bir süre 2**	**Bazen 3**	**Coğu zaman 4**	**Her zaman 5**
1. Son 4 hafta içinde, ne kadar sıklıkla kendinizi sebepsiz yere yorgun hissettiniz?	○	○	○	○	○
2. Son 4 hafta içinde, ne kadar sıklıkla kendinizi gergin hissettiniz?	○	○	○	○	○
3. Son 4 hafta icinde ne kadar sıklıkla sizi hiçbir şeyin sakinleştirmeyeceği kadar gergin hissettiniz?	○	○	○	○	○
4. Son 4 hafta içinde ne kadar sıklıkla kendinizi umutsuz hissettiniz?	○	○	○	○	○
5. Son 4 hafta içinde ne kadar sıklıkla kendinizi tedirgin veya huzursuz hissettiniz?	○	○	○	○	○
6. Son 4 hafta içinde ne kadar sıklıkla yerinde duramayacak kadar huzursuz hissettiniz?	○	○	○	○	○
7. Son 4 hafta içinde ne kadar sıklıkla kendinizi bunalımlı hissettiniz?	○	○	○	○	○
8. Son 4 hafta içinde ne kadar sıklıkla en ufak bir şeyin bile çok çaba gerektirdiğini hissettiniz?	○	○	○	○	○
9. Son 4 hafta içinde ne kadar sıklıkla kendinizi sizi hiçbir şeyin neşelendiremeyeceği kadar üzgün hissettiniz?	○	○	○	○	○
10. Son 4 hafta icinde ne kadar sıklıkla kendinizi değersiz hissettiniz?	○	○	○	○	○

Tanveer S, *et al. BMJ Open* 2023; 13:e067886. doi: 10.1136/bmjopen-2022-067886

PCL-5

Aşağıda, bazı kişilerin Covid-19'a tepki olarak yaşadığı sorunların bir listesi bulunmaktadır. Lütfen her bir sorunu dikkatlice okuyun ve geçen ay bu sorundan ne kadar rahatsız olduğunuzu belirtin. Geçen ay içinde aşağıda yer alan durumlar sizi ne ölçüde bunalttı:

	Hiç 0	Çok az 1	Orta derecede 2	Oldukça az 3	Aşırı 4
1. Stresli olayın tekrarlayan, rahatsız eden ve istenmeyen anları sizi ne kadar bunalttı?	○	○	○	○	○
2. Stresli olaya ilişkin tekrarlayan, rahatsız eden rüyalar sizi ne kadar bunalttı?	○	○	○	○	○
3. Aniden stresli olayı sanki gerçekten bir daha yaşıyormuş gibi hissetmek veya davranmak (sanki gerçekten olayın yaşandığı ana geri dönmüş yeniden yaşıyor gibi) sizi ne kadar bunalttı?	○	○	○	○	○
4. Bir şeyler size stresli olayı anımsattığı zaman yaşadığınız üzüntü hissi sizi ne kadar bunalttı?	○	○	○	○	○
5. Bir şeyler size stresli olayı anımsattığı zaman güçlü fiziksel tepkiler vermek (örneğin, kalp çarpıntısı, nefes almada güçlük, terleme gibi) sizi ne kadar bunalttı?	○	○	○	○	○
6. Stresli olayla ilişkili anılardan,düşüncelerden ve duygulardan kaçınmaya çalışmak sizi ne kadar bunalttı?	○	○	○	○	○
7. Stresli olayı anımsatan etraftaki hatırlatıcı şeylerden (örneğin, insanlardan, yerlerden, konuşmalardan, etkinliklerden, nesnelerden veya durumlardan) kaçınmaya çalışmak sizi ne kadar bunalttı?	○	○	○	○	○
8. Stresli olaya ilişkin önemli kısımları hatırlamada yaşanan güçlükler sizi ne kadar bunalttı?	○	○	○	○	○
9. Kendiniz, diğer insanlar veya dünya hakkında güçlü olumsuz düşüncelere sahip olmak (örneğin, kötü biriyim, bende ciddi şekilde yanlış olan bir şeyler var, kimseye güvenilmez, dünya tümüyle tehlikeli bir yerdir gibi düşünceler) sizi ne kadar bunalttı?	○	○	○	○	○

10. Stresli olay veya bu olayın sonrasında ortaya çıkan durumlar için kendinizi veya bir başkasını suçlamak sizi ne kadar bunalttı?	○	○	○	○	○
11. Korku, dehşete kapılma, öfke, suçluluk, veya utanç gibi olumsuz duygular sizi ne kadar bunalttı?	○	○	○	○	○
12. Daha önce yapmaktan keyif aldığınız etkinliklere olan ilginizi kaybetmek sizi ne kadar bunalttı?	○	○	○	○	○
13. Başka insanlardan uzak veya kopmuş hissetmek sizi ne kadar bunalttı?	○	○	○	○	○
14. Olumlu duyguları yaşamak (örneğin, mutluluğu hissetmek vaya size yakın insanlara sevgi dolu hisler duyamamak) sizi ne kadar bunalttı?	○	○	○	○	○
15. Asabi davranışlar, öfke patlamaları veya öfkeli hareketler sizi ne kadar bunalttı?	○	○	○	○	○
16. Çok fazla risk almak veya size zarar verebilecek şeyler yapmak sizi ne kadar bunaltı?	○	○	○	○	○
17. Aşırı tetikte olmak veya temkinli davranmak veya hazırda beklemek sizi ne kadar bunalttı?	○	○	○	○	○
18. Yerinden sıçramak veya kolayca irkilmek sizi ne kadar bunalttı?	○	○	○	○	○
19. Dikkati toplamada güçlükler sizi ne kadar bunalttı?	○	○	○	○	○
20. Uykuya dalma veya uykuyu devam ettirme güçlükleri sizi ne kadar bunalttı?	○	○	○	○	○

WHO-5

Aşağıdaki beş tanımlamadan her biri için, son iki hafta süresince kendinizi nasıl hissettiğinize en yakın olan yanıtı veriniz. Son iki hafta boyunca

	**Her zaman 5**	**Çoğu zaman 4**	**Zamanın yarısından çoğunda 3**	**Zamanın yarısından azında 2**	**Bazen 1**	**Hiçbir zaman 0**
1. Kendimi neşeli ve keyifli hissettim	○	○	○	○	○	○
2. Kendimi sakin ve rahatlamış hissettim.	○	○	○	○	○	○
3. Kendimi aktif ve dinç hissettim	○	○	○	○	○	○
4. Sabahları kendimi taze ve dinlenmiş hissederek uyandım	○	○	○	○	○	○
5. Günlük yaşantım beni ilgilendiren şeylerle dolu	○	○	○	○	○	○

PTGI

COVID-19 sonucunda yaşamınızda ve düşüncelerinizde meydana gelen değişimleri lütfen aşağıda verilen puanlama ölçütlerine göre 0 ve 5 arasında değerlendiriniz.

	Hiç 0	Çok az 1	Az derecede 2	Orta derecede 3	Oldukça fazla 4	Aşırı derecede 5
1. Yaşamda önem verdiğim şeylerin öncelik sırası değişti.	○	○	○	○	○	○
2. Kendi hayatıma verdiğim değerde büyük bir artış oldu.	○	○	○	○	○	○
3. Yeni ilgi alanları keşfettim.	○	○	○	○	○	○
4. Kendime güven hissinde artış oldu.	○	○	○	○	○	○
5. Manevi konuları daha iyi anlamaya başladım.	○	○	○	○	○	○
6. Başım sıkıştığında insanlara güvenebileceğimi daha iyi anladım.	○	○	○	○	○	○
7. Yaşamım için yeni bir yön belirledim.	○	○	○	○	○	○
8. Kendimi diğer insanlarla çok daha yakın hissetmeye başladım.	○	○	○	○	○	○
9. Duygularımı ifade etmeye daha çok istekliyim.	○	○	○	○	○	○
10. Zorlukları göğüsleyebileceğimi daha iyi anladım.	○	○	○	○	○	○
11. Yaşamımda daha iyi şeyler yapabiliyorum.	○	○	○	○	○	○
12. Her şeyi olduğu gibi, daha çok kabullenebiliyorum.	○	○	○	○	○	○
13. Her günümü daha iyi değerlendirebiliyorum.	○	○	○	○	○	○

14. Daha önce var olmayan yeni olanaklara kavuştum.	○	○	○	○	○	○
15. Diğer insanlara karşı daha şefkatliyim.	○	○	○	○	○	○
16. İlişkilerime daha çok emek sarf etmeye başladım.	○	○	○	○	○	○
17. Değişmesi gereken şeyleri değiştirebilmek için daha çok çaba harcıyorum.	○	○	○	○	○	○
18. Daha güçlü bir inanca sahibim.	○	○	○	○	○	○
19. Düşündüğümden çok daha güçlü olduğumu keşfettim.	○	○	○	○	○	○
20. İnsanların ne kadar mükemmel olabildiklerine dair çok şey öğrendim.	○	○	○	○	○	○
21. Başkalarına ihtiyaç duyuyor olmayı daha çok kabullendim.	○	○	○	○	○	○

International Study – Pakistan (Urdu)

Sociodemographic Information

1. جنس

- ○ مرد
- ○ عورت
- ○ دیگر
- ○ جواب نہیں دینا چاہتے

2. عمر (سالوں میں)

____

3. ازدواجی حیثیت

- ○ غیر شادی شدہ
- ○ غیر ازدوجی تعلق
- ○ شادی شدہ
- ○ علیحدگی
- ○ طلاق یافتہ
- ○ بیوہ یا رنڈوا

4. آپ کا دین کیا ہے؟

- ○ کوئی دین نہیں
- ○ اسلام
- ○ عیسائیت
- ○ ہندومت
- ○ بدھ مت

○ دیگر (براہ مہربانی وضاحت کریں) ____________________________________

5. آپ کا نسلی پس منظر کیا ہے؟ (آپ ایک سے زیادہ درج کرسکتے ہیں)

____

6. آپ روزمرہ کی زندگی میں کون سی زبانیں روانی سے بولتے ہیں؟

____

درج ذیل دو سوالات آپ کی انگریزی زبان کی اہلیت کے بارے میں ہیں۔
6a. میں انگریزی اچھی طرح سمجھ سکتا/سکتی ہوں؟*

○ بالکل بھی نہیں1

○ تھوڑی سی2

○ درمیانی حد تک3

○ زیادہ4

○ بہت زیادہ5

6b. میں انگریزی اچھی طرح بول سکتا/سکتی ہوں؟

○ بالکل بھی نہیں1

○ تھوڑی سی 2

○ درمیانی حد تک3

○ زیادہ4

○ بہت زیادہ5

7. آپ کی تعلیم کی اعلی ترین سطح کیا ہے؟

○ کوئی باضابطہ تعلیم نہیں

○ ہائیر سیکنڈری سکول (جیسے ڈپلومہ یا A-level)

○ سرٹیفکیٹ یا ڈپلومہ 3 - سال سے کم کورس

○ بیچلر ڈگری (3-4 سالہ کورس)

○ پوسٹ گریجویٹ ڈگری (پوسٹ گریجویٹ ڈپلومہ، ماسٹر، ڈاکٹریٹ)

8. موجودہ اسٹڈی یا کام کی حیثیت (براہ مہربانی جو بھی لاگو ہوں ان پر نشان لگائیں)

- ☐ فل ٹائم طالب علم
- ☐ پارٹ ٹائم طالب علم
- ☐ بے روزگار/کام کی تلاش
- ☐ فل ٹائم ملازمت
- ☐ پارٹ ٹائم ملازمت
- ☐ فل ٹائم ذاتی کام
- ☐ پارٹ ٹائم ذاتی کام
- ☐ دفتری پیڈ پیرینٹل چھٹی (paid parental leave)
- ☐ ہاؤس وائف یا سٹیے-ایٹ-ہوم والد (stay-at-home dad) بلا معاوضہ
- ☐ ریٹائرڈ
- ☐ دیگر (براہ مہربانی وضاحت کریں) ___________________________________

9. پیشہ

____

10. ماہانہ گھریلو آمدنی (مقامی کرنسی میں)

____

10a. اوسط آمدن کے مقابلے میں، آپ اپنے گھر کی آمدنی کی درجہ بندی کیسے کریں گے؟

○ اوسط سے کم

○ اوسط

○ اوسط سے زیادہ

11. کیا آپ کوویڈ 19-کی وجہ سے لاک ڈاؤن میں ہیں؟

○ ہاں

○ نہیں

12. کیا آپ کو کوویڈ 19-کی ویکسین دی گئی ہے؟

- ہاں
- نہیں

12a. اگر نہیں، تو کیا آپ مستقبل میں کوویڈ 19-کی ویکسین لینا چاہیں گیں؟

- ہاں
- نہیں

13. ہم سمجھتے ہیں کہ کچھ لوگوں کو ماضی میں تکلیف دہ واقعات کا سامنا کرنا پڑا ہوا ہو گا جس کی وجہ سے نئی پریشانیوں سے نمٹنے میں آسانی یا دقت پیدا ہو سکتی ہے۔

کوویڈ-19کی وبا سے پہلے، کیا آپ نے کبھی مندرجہ ذیل میں سے کسی واقعہ کا مشاہدہ یا تجربہ کیا ہے: (براہ مہربانی جو بھی لاگو ہوں ان پر نشان لگائیں)

- ☐ قدرتی آفات (جیسے سیلاب، زلزلہ، وغیرہ)
- ☐ جنگ یا فوجی تنازعات
- ☐ 16 سال کی عمر سے پہلے، بچپن کی پریشانیاں (جیسے نظر انداز ہونا (neglect)، غنڈہ گردی کا شکار (bullying) جسمانی یا جنسی تشدد)
- ☐ 16 سال کی عمر کے بعد جسمانی یا جنسی تشدد
- ☐ سنگین حادثہ / ایکسیڈنٹ
- ☐ دیگر – وضاحت کریں (وضاحت کرنا لازمی نہیں)____
- ☐ مندرجہ بالا میں سے کوئی نہیں

Covid Psychosocial Impacts Scale

ذاتی اثرات

مندرجہ ذیل بیانات کوویڈ-19 کے آپ پر ہونے والے اثرات کے بارے میں ہیں، براہ مہربانی درج ذیل بیانات کے لئے" ہاں / نہیں "میں سے کسی ایک کا انتخاب کریں۔ اگر آپ کا جواب ہاں میں ہے تو، براہ مہربانی نیچے دیے گیے اسکیل پر اس کی وجہ سے ہونے والے دباؤ (stress) کی نشاندہی کریں۔

1. کیا آپ کو کوئی ایسی بیماری لاحق ہے جو آپ کو کوویڈ-19 کی زِد میں لا سکتی ہے؟

- ○ **نہیں** 0
- ○ **ہاں** کوئی دباؤ نہیں 1
- ○ **ہاں** تھوڑا سا دباؤ 2
- ○ **ہاں** درمیانی حد تک دباؤ 3
- ○ **ہاں** زیادہ دباؤ 4
- ○ **ہاں** بہت زیادہ دباؤ 5

2. کیا آپ کو کسی شخص سے کوویڈ-19 کی زِد میں آنے کا خطرہ لاحق ہوا ہے؟

- ○ **نہیں** 0
- ○ **ہاں** کوئی دباؤ نہیں 1
- ○ **ہاں** تھوڑا سا دباؤ 2
- ○ **ہاں** درمیانی حد تک دباؤ 3
- ○ **ہاں** زیادہ دباؤ 4
- ○ **ہاں** بہت زیادہ دباؤ 5

3. کیا آپ کو لگتا ہے کہ آپ کو کوویڈ-19 ہوا تھا؟

- ○ **نہیں** 0
- ○ **ہاں** کوئی دباؤ نہیں 1

ہاں تھوڑا سا دباؤ 2

ہاں درمیانی حد تک دباؤ 3

ہاں زیادہ دباؤ 4

ہاں بہت زیادہ دباؤ 5

3a. کیا آپ کا کوویڈ-19 کا ٹیسٹ مثبت آیا تھا؟

نہیں 0

ہاں کوئی دباؤ نہیں 1

ہاں تھوڑا سا دباؤ 2

ہاں درمیانی حد تک دباؤ 3

ہاں زیادہ دباؤ 4

ہاں بہت زیادہ دباؤ 5

3b. کیا آپ کو آج کل کوویڈ-19 کی علامات ہیں؟

نہیں 0

ہاں کوئی دباؤ نہیں 1

ہاں تھوڑا سا دباؤ 2

ہاں درمیانی حد تک دباؤ 3

ہاں زیادہ دباؤ 4

ہاں بہت زیادہ دباؤ 5

خاندان والوں پر اثرات

مندرجہ ذیل بیانات کوویڈ-19 کے آپ کے قریبی خاندان والوں پر ہونے والے اثرات کے بارے میں ہیں، براہ مہربانی درج ذیل بیانات کے لئے "ہاں / نہیں "میں سے کسی ایک کا انتخاب کریں. اگر آپ کا جواب ہاں میں ہے تو، براہ مہربانی نیچے دیے گیے اسکیل پر اس کی وجہ سے ہونے والے دباؤ (stress) کی نشاندہی کریں.

1. کیا آپ کے خاندان کے کسی قریبی فرد کو کوئی ایسی بیماری ہے جو انہیں کوویڈ-19 کی زِد میں لا سکتی ہے؟

- **نہیں** 0
- **ہاں** کوئی دباؤ نہیں 1
- **ہاں** تھوڑا سا دباؤ 2
- **ہاں** درمیانی حد تک دباؤ 3
- **ہاں** زیادہ دباؤ 4
- **ہاں** بہت زیادہ دباؤ 5

2. کیا آپ کو خدشہ ہے کہ آپ کے خاندان کے کسی قریبی فرد کو کسی شخص سے کوویڈ-19کی زِد میں آنے کا خطرہ لاحق ہوا ہے؟

- **نہیں** 0
- **ہاں** کوئی دباؤ نہیں 1
- **ہاں** تھوڑا سا دباؤ 2
- **ہاں** درمیانی حد تک دباؤ 3
- **ہاں** زیادہ دباؤ 4
- **ہاں** بہت زیادہ دباؤ 5

3. کیا آپ کو لگتا ہے کہ خاندان کے کسی قریبی فرد کو کوویڈ-19 ہوا تھا؟

- **نہیں** 0
- **ہاں** کوئی دباؤ نہیں 1
- **ہاں** تھوڑا سا دباؤ 2

ہاں درمیانی حد تک دباؤ 3

ہاں زیادہ دباؤ 4

ہاں بہت زیادہ دباؤ 5

3a. کیا خاندان کے کسی قریبی فرد کا کوویڈ-19 کا ٹیسٹ مثبت آیا تھا؟

نہیں 0

ہاں کوئی دباؤ نہیں 1

ہاں تھوڑا سا دباؤ 2

ہاں درمیانی حد تک دباؤ 3

ہاں زیادہ دباؤ 4

ہاں بہت زیادہ دباؤ 5

3b. کیا خاندان کے کسی قریبی فرد کو آج کل کوویڈ-19 کی علامات ہیں؟

نہیں 0

ہاں کوئی دباؤ نہیں 1

ہاں تھوڑا سا دباؤ 2

ہاں درمیانی حد تک دباؤ 3

ہاں زیادہ دباؤ 4

ہاں بہت زیادہ دباؤ 5

3c. کیا خاندان کے کسی قریبی فرد کا کوویڈ-19 سے انتقال ہوا ہے؟

نہیں 0

ہاں کوئی دباؤ نہیں 1

ہاں تھوڑا سا دباؤ 2

ہاں درمیانی حد تک دباؤ 3

ہاں زیادہ دباؤ 4

ہاں بہت زیادہ دباؤ 5

4. کیا آپ کو فکر لاحق ہے کہ بیرون ملک مقیم خاندان کے کسی قریبی فرد کو کوویڈ-19 کی زِد میں آنے کا امکان ہے؟

نہیں 0

ہاں کوئی دباؤ نہیں 1

ہاں تھوڑا سا دباؤ 2

ہاں درمیانی حد تک دباؤ 3

ہاں زیادہ دباؤ 4

ہاں بہت زیادہ دباؤ 5

5. کیا بیرون ملک مقیم خاندان کے کسی قریبی فرد کو آپ کوویڈ-19 کی وجہ سے ملنے نہیں جا سکتے یا وہ آپ کوویڈ-19 کی وجہ سے ملنے نہیں آ سکتے ہیں؟

نہیں 0

ہاں کوئی دباؤ نہیں 1

ہاں تھوڑا سا دباؤ 2

ہاں درمیانی حد تک دباؤ 3

ہاں زیادہ دباؤ 4

ہاں بہت زیادہ دباؤ 5

کام

مندرجہ ذیل بیانات کوویڈ-19 کے آپ اور آپ کے گھرانے پر ہونے والے اثرات کے بارے میں ہیں، براہ مہربانی درج ذیل بیانات کے لئے " ہاں / نہیں / قابل اطلاق نہیں "میں سے کسی ایک کا انتخاب کریں. اگر آپ کا جواب ہاں میں ہے تو، براہ مہربانی نیچے دیے گیے اسکیل پر اس کی وجہ سے ہونے والے دباؤ (stress) کی نشاندہی کریں.

1. کیا آپ یا آپ کے گھر کا کوئی فرد ایک ضروری ورکر کے طور پر شمار کیا گیا ہے (مثلاً ہیلتھ کیئر، قانون نافذ کرنے والے ادارے، ایمرجنسی سروسز، اسینشل/ضروری سامان/خدمات فراہم کرنے والے اداروں میں)؟

- ○ **نہیں** 0
- ○ **ہاں** کوئی دباؤ نہیں 1
- ○ **ہاں** تھوڑا سا دباؤ 2
- ○ **ہاں** درمیانی حد تک دباؤ 3
- ○ **ہاں** زیادہ دباؤ 4
- ○ **ہاں** بہت زیادہ دباؤ 5

2. کیا آپ کے یا آپ کے گھرانے کے کسی فرد کے کام کرنے کے اوقات میں ایک بڑی تبدیلی رونما ہوئی ہے؟

- ○ **نہیں** 0
- ○ **ہاں** کوئی دباؤ نہیں 1
- ○ **ہاں** تھوڑا سا دباؤ 2
- ○ **ہاں** درمیانی حد تک دباؤ 3
- ○ **ہاں** زیادہ دباؤ 4
- ○ **ہاں** بہت زیادہ دباؤ 5

3. کیا آپ کو یا آپ کے گھرانے کے کسی فرد کو گھر سے کام کرنا پڑرہا ہے؟

- ○ **نہیں** 0
- ○ **ہاں** کوئی دباؤ نہیں 1
- ○ **ہاں** تھوڑا سا دباؤ 2
- ○ **ہاں** درمیانی حد تک دباؤ 3
- ○ **ہاں** زیادہ دباؤ 4

- ہاں بہت زیادہ دباؤ 5

4. کیا آپ کے یا آپ کے گھرانے کے کسی فرد کے کام (job-related work) کی نوعیت بَدَل گئی ہے؟

- نہیں 0
- ہاں کوئی دباؤ نہیں 1
- ہاں تھوڑا سا دباؤ 2
- ہاں درمیانی حد تک دباؤ 3
- ہاں زیادہ 4
- ہاں بہت زیادہ دباؤ 5

5. کیا آپ کی یا آپ کے گھرانے کے کسی فرد کی ملازمت چھوٹ گئی ہے؟

- قابل اطلاق نہیں
- نہیں 0
- ہاں کوئی دباؤ نہیں 1
- ہاں تھوڑا سا دباؤ 2
- ہاں درمیانی حد تک دباؤ 3
- ہاں زیادہ دباؤ 4
- ہاں بہت زیادہ دباؤ 5

6. کیا ایسا کاروبار جو آپ کی یا آپ کے گھرانے کی ملکیت ہے، یا جس میں آپ یا آپ کے گھر والے کام کرتے ہیں، بقا کے خطرے میں ہے؟

- قابل اطلاق نہیں
- نہیں 0
- ہاں کوئی دباؤ نہیں 1

ہاں تھوڑا سا دباؤ 2

ہاں درمیانی حد تک دباؤ 3

ہاں زیادہ دباؤ 4

ہاں بہت زیادہ دباؤ 5

آمدن

مندرجہ ذیل بیانات کوویڈ-19 کے آپ کے گھرانے پر ہونے والے اثرات کے بارے میں ہیں، براہ مہربانی درج ذیل بیانات کے لئے" ہاں / نہیں " میں سے کسی ایک کا انتخاب کریں۔ اگر آپ کا جواب ہاں میں ہے تو، براہ مہربانی نیچے دیے گیے اسکیل پر اس کی وجہ سے ہونے والے دباؤ (stress) کی نشاندہی کریں۔

1. کیا آپ کے گھرانے کے مالی حالات میں واضح کمی واقع ہوئی ہے؟

نہیں 0

ہاں کوئی دباؤ نہیں 1

ہاں تھوڑا سا دباؤ 2

ہاں درمیانی حد تک دباؤ 3

ہاں زیادہ دباؤ 4

ہاں بہت زیادہ دباؤ 5

2. کیا آپ کے گھرانے کی بلوں کی ادائیگی یا کھانے پینے کے اخراجات کرنے کی صلاحیت میں واضح کمی واقع ہوئی ہے؟

نہیں 0

ہاں کوئی دباؤ نہیں 1

ہاں تھوڑا سا دباؤ 2

ہاں درمیانی حد تک دباؤ 3

ہاں زیادہ دباؤ 4

ہاں بہت زیادہ دباؤ 5

تعلقات

مندرجہ ذیل بیانات کوویڈ-19 کے آپ پر ہونے والے اثرات کے بارے میں ہیں، براہ مہربانی درج ذیل بیانات کے لئے" ہاں / نہیں "میں سے کسی ایک کا انتخاب کریں۔ اگر آپ کا جواب ہاں میں ہے تو، براہ مہربانی نیچے دیے گیے اسکیل پر اس کی وجہ سے ہونے والے دباؤ (stress) کی نشاندہی کریں۔

1. کیا کوویڈ-19 کے نتیجے میں، فیملی کی مشکلات اور باہمی ناچاقی میں اضافہ ہوا ہے؟

- نہیں 0
- ہاں کوئی دباؤ نہیں 1
- ہاں تھوڑا سا دباؤ 2
- ہاں درمیانی حد تک دباؤ 3
- ہاں زیادہ دباؤ 4
- ہاں بہت زیادہ دباؤ 5

2. کیا کوویڈ-19 کے نتیجے میں، دوستوں کے ساتھ آپ کے رابطے (روبرو یا آن لائن) میں کمی واقع ہوئی ہے؟

- نہیں 0
- ہاں کوئی دباؤ نہیں 1
- ہاں تھوڑا سا دباؤ 2
- ہاں درمیانی حد تک دباؤ 3
- ہاں زیادہ دباؤ 4
- ہاں بہت زیادہ دباؤ 5

روزمرہ کے معمولات اور طرزعمل میں تبدیلیاں

مندرجہ ذیل بیانات کوویڈ-19 کے آپ پر ہونے والے اثرات کے بارے میں ہیں، براہ مہربانی درج ذیل بیانات کے لئے" ہاں / نہیں" میں سے کسی ایک کا انتخاب کریں۔ اگر آپ کا جواب ہاں میں ہے تو، براہ مہربانی نیچے دیے گیے اسکیل پر اس کی وجہ سے ہونے والے دباؤ (stress) کی نشاندہی کریں۔

1. کیا کوویڈ-19 کی وجہ سے آپ کے روزمرہ معمولات (مثال کے طور پر، نیند، ورزش، فارغ وقت، خوشگوار سرگرمیوں، یا مشاغل) میں تبدیلی واقع ہوئی ہے؟

- **نہیں** 0
- **ہاں** کوئی دباؤ نہیں 1
- **ہاں** تھوڑا سا دباؤ 2
- **ہاں** درمیانی حد تک دباؤ 3
- **ہاں** زیادہ دباؤ 4
- **ہاں** بہت زیادہ دباؤ 5

2. کیا کوویڈ-19 کی وجہ سے آپ نے اپنے یا اپنے گھر والوں کی ہیلتھ کیئر (healthcare) کے حصول میں تاخیر کی ہے؟

- **نہیں** 0
- **ہاں** کوئی دباؤ نہیں 1
- **ہاں** تھوڑا سا دباؤ 2
- **ہاں** درمیانی حد تک دباؤ 3
- **ہاں** زیادہ دباؤ 4
- **ہاں** بہت زیادہ دباؤ 5

3. کیا کوویڈ-19 کی وجہ سے آپ کی دینی سرگرمیوں(روبرو یا آن لائن) میں شمولیت میں کمی واقع ہوئی ہے؟

- **قابل اطلاق نہیں**
- **نہیں** 0
- **ہاں** کوئی دباؤ نہیں 1
- **ہاں** تھوڑا سا دباؤ 2
- **ہاں** درمیانی حد تک دباؤ 3
- **ہاں** زیادہ دباؤ 4

Tanveer S, *et al. BMJ Open* 2023; 13:e067886. doi: 10.1136/bmjopen-2022-067886

○ ہاں بہت زیادہ دباؤ 5

4. کیا کوویڈ-19 کی وجہ سے معمول میں کوئی اور بڑی تبدیلی واقع ہوئی ہے؟

○ نہیں 0

○ **ہاں (براہ مہربانی وضاحت کریں۔)** 1 ___________________________________

اگر آپ کا جواب ہاں میں ہے تو، براہ مہربانی نیچے دیے گیے اسکیل پر اس کی وجہ سے ہونے والے دباؤ (stress) کی نشاندہی کریں۔

○ کوئی دباؤ نہیں 1

○ تھوڑا سا دباؤ 2

○ درمیانی حد تک دباؤ 3

○ زیادہ دباؤ 4

○ بہت زیادہ دباؤ 5

کوویڈ-19 کے بارے میں معلومات

1. کوویڈ-19 کے حوالے سے، آپ کے لئے معلومات کا ذریعہ کیا ہے؟ (براہ مہربانی جو بھی آپ پر لاگو ہوں انہیں منتخب کریں۔)

- ☐ خبریں
- ☐ فیس بک / ٹویٹر
- ☐ ہیلتھ ویب سائٹس
- ☐ لوگوں کی باتیں
- ☐ دیگر(براہ مہربانی وضاحت کریں) ___________________________________

2. کوویڈ-19 سے متعلق خبریں آپ کے لیے کس قدر دباؤ (stress) کا سبب ہیں؟

○ کوئی دباؤ نہیں 1

○ تھوڑا سا دباؤ 2

○ درمیانی حد تک دباؤ 3

○ زیادہ دباؤ 4

○ بہت زیادہ دباؤ 5

کوویڈ۔ 19کے بعد ہونے والی تبدیلیاں

براہ کرم کوویڈ-19 کی عالمی وبا کا پہلے سے موازنہ کرتے ہوئے، درج ذیل بیانات پر دیئے گئے پیمانے کی مدد سے نشاندہی کریں۔

1. آپ اپنے ذاتی ذہنی دباؤ (stress) کی درجہ بندی کس طرح کریں گیں؟

- پہلے سے بہت کم دباؤ 1
- پہلے سے کم دباؤ 2
- کوئی تبدیلی نہیں 3
- پہلے سے زیادہ دباؤ 4
- پہلے سے بہت زیادہ دباؤ 5

2. آپ اپنے گھرانے کے ذہنی دباؤ (stress) کی درجہ بندی کس طرح کریں گیں؟

- پہلے سے بہت کم دباؤ 1
- پہلے سے کم دباؤ 2
- کوئی تبدیلی نہیں 3
- پہلے سے زیادہ دباؤ 4
- پہلے سے بہت زیادہ دباؤ 5

3. آپ اپنی نفسیاتی صحت کی درجہ بندی کس طرح کریں گیں؟

- پہلے سے بہت بہتر 1
- پہلے سے بہتر 2
- کوئی تبدیلی نہیں 3
- پہلے سے بدتر 4
- پہلے سے بہت بدتر 5

4. آپ اپنے گھرانے کی نفسیاتی صحت کی درجہ بندی کس طرح کریں گیں؟

- پہلے سے بہت بہتر 1
- پہلے سے بہتر 2
- کوئی تبدیلی نہیں 3
- پہلے سے بدتر 4
- پہلے سے بہت بدتر 5

K-10

نیچے دئیے گئے دس سوالات میں آپ سے پوچھا گیا ہے کہ آپ پچھلے **4** ہفتوں سے کیسا محسوس کر رہے ہیں۔ برائے مہربانی ہر سوال کے لئے نشاندہی کریں جو اس بات کی بہترین وضاحت کرتا ہو کہ آپ نے اس کیفیت کو کتنی مرتبہ محسوس کیا ہے۔

	**کبھی نہیں 1**	**بہت کم وقت 2**	**کبھی کبھار 3**	**زیادہ وقت 4**	**ہر وقت 5**
1. پچھلے 4 ہفتوں میں، آپ نے کتنی مرتبہ بغیر کسی معقول وجہ کے تھکاوٹ محسوس کی؟	○	○	○	○	○
2. پچھلے 4 ہفتوں میں، آپ نے کتنی مرتبہ گھبراہٹ محسوس کی؟	○	○	○	○	○
3. پچھلے 4 ہفتوں میں، آپ نے کتنی مرتبہ اتنی گھبراہٹ محسوس کی کہ کوئی بھی چیز آپ کو سکون نہ دے سکی؟	○	○	○	○	○
4. پچھلے 4 ہفتوں میں، آپ نے کتنی مرتبہ نا اُمیدی محسوس کی؟	○	○	○	○	○
5. پچھلے 4 ہفتوں میں، آپ نے کتنی مرتبہ بے سکونی یا بے چینی محسوس کی؟	○	○	○	○	○
6. پچھلے 4 ہفتوں میں، آپ نے کتنی مرتبہ اتنی بے چینی محسوس کی کہ آپ ٹک کے نہ بیٹھ سکتے تھے؟	○	○	○	○	○
7. پچھلے 4 ہفتوں میں، آپ نے کتنی مرتبہ افسردگی (ڈپریشن) محسوس کی*	○	○	○	○	○
8. پچھلے 4 ہفتوں میں، آپ نے کتنی مرتبہ محسوس کیا کہ ہر کام کرنے کے لیے کوشش کرنی پڑ رہی ہے؟	○	○	○	○	○
9. پچھلے 4 ہفتوں میں، آپ کتنی مرتبہ اتنے اُداس تھے کہ کوئی بھی چیز آپ کو خوش نہ کر سکی؟	○	○	○	○	○
10. پچھلے 4 ہفتوں میں، آپ نے کتنی مرتبہ خود کو بے وقعت محسوس کیا؟	○	○	○	○	○

PCL-5

نیچے ان مسائل کی فہرست دی گئی ہے جو لوگوں کو کوویڈ-19 کے نتیجے میں پیش آ سکتے ہیں۔ براہ مہربانی ہر مسئلے کو غور سے پڑھیں اور پھر دیے گئے نمبروں میں سے کسی ایک کو چن کر نشاندہی کریں کہ پچھلے مہینے میں آپ کو اس مسئلے نے کتنا پریشان کیا ہے۔

	**بالکل بھی نہیں** 0	**تھوڑا سا** 1	**درمیانی حد تک** 2	**زیادہ** 3	**بہت زیادہ** 4
1. پریشان کن تجربے کی بار بار آنے والی، تکلیف دہ، اور ناپسندیدہ یادیں؟	○	○	○	○	○
2. پریشان کن تجربے کے بار بار آنے والے، تکلیف دہ خواب؟	○	○	○	○	○
3. اچانک محسوس ہونا یا ایسا لگنا کہ پریشان کن تجربہ واقعی دوبارہ ہو رہا ہے (جیسے آپ ماضی میں چلے گئے ہوں اور اس سے دوبارہ گزر رہے ہوں)۔	○	○	○	○	○
4. بہت گھبراہٹ محسوس کرنا جب کوئی چیز آپ کو پریشان کن تجربے کی یاد دلائے۔	○	○	○	○	○
5. سخت جسمانی رد عمل محسوس کرنا جب کوئی چیز آپ کو پریشان کن تجربے کی یاد دلائے (مثلاً، دل کا زور زور سے دھڑکنا، سانس لینے میں دشواری، پسینہ چھوٹنا)؟	○	○	○	○	○
6. پریشان کن تجربے سے متعلق یادوں، خیالات، یا احساسات سے گریز کرنا؟	○	○	○	○	○
7. پریشان کن تجربے کی بیرونی یاد دہانیوں سے گریزکرنا (مثلاً لوگ، مقامات، گفتگو، سرگرمیاں، اشیاء، یا حالات)؟	○	○	○	○	○
8. پریشان کن تجربے کے اہم حصوں کو یاد کرنے میں دقت محسوس کرنا؟	○	○	○	○	○
9. اپنے، دوسرے لوگوں، یا دنیا کے بارے میں سخت منفی خیالات آنا (مثلاً، اس طرح کے خیالات جیسے: میں برا/بری ہوں، میرے ساتھ واقعی میں کچھ مسئلہ ہے، کسی پر بھی اعتبار نہیں کیا جاسکتا، پوری دنیا خطرناک ہے)؟	○	○	○	○	○
10. پریشان کن تجربے کے لئے یا اس کے بعد جو کچھ ہوا اس کے لئے اپنے آپ کو یا کسی اور کو مورد الزام ٹھہرانا؟	○	○	○	○	○
11. سخت منفی جذبات کا ہونا جیسے خوف، وحشت، غصہ، احساس جرم، یا شرمندگی ؟	○	○	○	○	○
12. ان سرگرمیوں میں عدم دلچسپی جن سے آپ کبھی لطف اندوز ہوتے تھے؟	○	○	○	○	○
13. دوسرے لوگوں سے دوری محسوس کرنا یا تعلقات منقطع کرنا؟	○	○	○	○	○

Tanveer S, *et al. BMJ Open* 2023; 13:e067886. doi: 10.1136/bmjopen-2022-067886

14. مثبت احساسات محسوس کرنے میں دشواری (مثلاً، آپ کا اپنے قریبی لوگوں کے لئے خوشی یا محبت کے جذبات محسوس کرنے سے قاصر ہونا)؟	○	○	○	○	○
15. چڑچڑاپن، برہمی، یا جارحانہ سلوک؟	○	○	○	○	○
16. بہت زیادہ خطرات مول لینا یا ایسی چیزیں کرنا جو آپ کو نقصان پہنچا سکتی ہیں؟	○	○	○	○	○
17. انتہائی الرٹ/چوکنا یا محتاط رہنا؟	○	○	○	○	○
18. بہت تیزی یا آسانی سے چونک جانا؟	○	○	○	○	○
19. توجہ دینے میں دقت پیش آنا؟	○	○	○	○	○
20. سونے میں دشواری یا نیند کے مسائل؟	○	○	○	○	○

Tanveer S, *et al. BMJ Open* 2023; 13:e067886. doi: 10.1136/bmjopen-2022-067886

WHO-5

براہِ مہربانی ان پانچ بیانات میں سے ہر ایک کے لئے نشاندہی کریں کہ آپ پچھلے دو ہفتوں سے کیسا محسوس کر رہے ہیں۔

	**ہر وقت**<br>**5**	**زیادہ تر وقت**<br>**4**	**آدھے سے زیادہ وقت میں**<br>**3**	**آدھے سے کم وقت میں**<br>**2**	**کچھ وقت**<br>**1**	**کسی وقت بھی نہیں**<br>**0**
1. میں نے خوشی اور زندہ دلی محسوس کی	○	○	○	○	○	○
2. میں نے سکون اور راحت محسوس کیا	○	○	○	○	○	○
3. میں نے فعال اور چست محسوس کیا	○	○	○	○	○	○
4. میں نے سو کر اٹھتے ہوئے تازہ دم اور آرام دہ محسوس کیا	○	○	○	○	○	○
5. میری روزمرہ زندگی ایسی چیزوں سے بھری پڑی ہے جن میں مجھے دلچسپی ہے	○	○	○	○	○	○

Tanveer S, *et al. BMJ Open* 2023; 13:e067886. doi: 10.1136/bmjopen-2022-067886

PGTI

درج ذیل بیانات میں سے ہر ایک کی دیے گئے اسکیل کی مدد سے نشاندہی کریں کہ کوویڈ 19-کے نتیجے میں آپ کی زندگی میں کس درجہ کی تبدیلی واقع ہوئی ہے۔

	بالکل نہیں 0	بہت تھوڑا 1	تھوڑا 2	درمیانی حد تک 3	زیادہ 4	بہت زیادہ 5
1. زندگی میں کیا اہم ہے اس کے بارے میں میں نے اپنی ترجیحات کو تبدیل کر دیا ہے						
2. مجھے اپنی زندگی کی قدروقیمت پہلے سے زیادہ ہے						
3. میں نے نئی دلچسپیاں بنا لی ہیں						
4. مجھے احساس خود انحصاری پہلے سے زیادہ ہے						
5. مجھے روحانی امور کی سمجھ بوجھ پہلے سے بہتر ہے						
6. میں پہلے سے بہتر طور پر دیکھ سکتا /سکتی ہوں کہ میں مصیبت کے وقت لوگوں پر بھروسہ کر سکتا / سکتی ہوں						
7. میں نے اپنی زندگی کی ایک نئی راہ متعین کر لی ہے						
8. مجھے دوسروں کے ساتھ قربت کا احساس پہلے سے زیادہ ہے						
9. میں اپنے جذبات کا اظہار کرنے کے لیے پہلے سے زیادہ آمادہ ہوں						
10. میں پہلے سے بہتر جانتا / جانتی ہوں کہ میں مشکلات کا مقابلہ کر سکتا / سکتی ہوں						
11. میں اپنی زندگی میں پہلے سے بہتر کام سَر انجام دے رہا / رہی ہوں						
12. میری چیزوں کا انجام قبول کرنے کی صلاحیت پہلے سے بہتر ہے						
13. میں ہر دن کی قدر پہلے سے زیادہ کرتا / کرتی ہوں						

14. نئے مواقع میسر ہیں جو بصورت دیگر میسر نہ ہوتے						
15. مجھ میں دوسروں کے لئے شفقت پہلے سے زیادہ ہے						
16. میں اپنے تعلقات نبھانے میں پہلے سے زیادہ محنت کرتا / کرتی ہوں						
17. میں ان چیزوں کو تبدیل کرنے کی پہلے سے زیادہ کوشش کرتا / کرتی ہوں جن کو تبدیل کرنے کی ضرورت ہے						
18. میرا ایمان پہلے سے زیادہ مضبوط ہے						
19. میں نے جان لیا ہے کہ میں اپنی سوچ سے ذیادہ مضبوط ہوں						
20. میں نے پہلے سے بھی ذیادہ سیکھا ہے کہ لوگ کتنے اچھے ہیں						
21. میں دوسروں سے ملنے والی مدد کو قبول کرنے کے لیے پہلے سے زیادہ آمادہ ہوں						

International Study – Iran (Persian)

Sociodemographic Information

۱. جنسیت:

- مرد
- زن
- دیگر
- ترجیح می‌دهم بیان نکنم

۲. سن (به سال)

____

۳. وضعیت تأهل

- هرگز ازدواج نکرده ام
- با کسی در رابطه‌ام(پارتنر دارم)
- ازدواج کرده‌ام
- متارکه
- مطلقه
- بیوه

۴. دین شما چیست؟

- بی‌دین
- اسلام
- مسیحیت
- هندو
- بودایی

Tanveer S, et al. BMJ Open 2023; 13:e067886. doi: 10.1136/bmjopen-2022-067886

○ ادیان یگر (لطفا مشخص فرمایید)________________________________

۵. پیشینه قومی شما چیست؟ (می توانید بیش از یک مورد ذکر کنید)

____

۶. بطور روزمره به چه زبانی صحبت می‌کنید؟

____

دو پرسش زیر بطور خاص درباره مهارت شما در به کارگیری زبان انگلیسی می‌پرسد.

۶الف. من انگلیسی را خوب می‌فهمم

○ کاملا مخالف

○ مخالف

○ نه موافق نه مخالف

○ موافق

○ کاملا موافق

۶ب. من خوب انگلیسی حرف می‌زنم

○ هیچی

○ کمی

○ تا حدودی

○ خیلی

○ خیلی زیاد

۷. بالاترین مدرک تحصیلی‌تان چیست؟

○ فاقد مدرک رسمی (حضور در مدرسه، کلاس انگلیسی برای گویشوران به زبان های دیگر ESOL)

○ دبیرستان (مثلاً مدرک ان سی ای ایNational Certificate of Educational Achievement or NCEA ، دیپلم آی بی International Baccalaureate or IB، مدرک مدارس هر جایی)

○ مدرک دانشگاهی (مثل، گواهی نامه، دیپلم یا مدرک فنی – دوره های کمتر از 3 سال)

○ مدرک لیسانس(دوره ۳-۴ ساله)

○ مدرک تحصیلات تکمیل (دیپلم تحصیلات تکمیلی، کارشناسی ارشد، دکترا)

---

۸. وضعیت تحصیلی، کاری و شغلی فعلی (لطفاً گزینه های مربوط را علامت بزنید)

- ☐ تحصیل تمام وقت
- ☐ تحصیل پاره وقت
- ☐ بیکار
- ☐ استخدام تمام وقت
- ☐ استخدام پاره وقت
- ☐ بطور تمام وقت خوداشتغالم (کار و کاسبی خودم را دارم)
- ☐ بطور پاره‌وقت خوداشتغالم
- ☐ در مرخصی با حقوق پس از فرزنددار شدن بسر می‌برم
- ☐ والد خانه‌نشینم (سر کار نمی‌روم و بدون حقوقم)
- ☐ بازنشسته‌ام
- ☐ دیگر (لطفا مشخص کنید)________________________________

۹. شغل

____

۱۰ .درآمد ماهانه خانوار (به واحد پول محلی)

____

۱۰الف. در مقایسه با متوسط درآمد، شما درآمد خانوار خودتان را چطور ارزیابی می‌کنید؟

○ زیرمتوسط

○ متوسط

○ بالای متوسط

۱۱. آیا شما در حال حاضر در قرنطینه‌اید؟

Tanveer S, *et al. BMJ Open* 2023; 13:e067886. doi: 10.1136/bmjopen-2022-067886

- ○ بلی
- ○ خیر

۱۲. آیا واکسن کووید-۱۹ زده‌اید؟

- ○ بلی
- ○ خیر

۱۲الف. اگر هنوز واکسن نزده‌اید، آیا دوست دارید در آینده واکسن کووید-۱۹ بزنید؟

- ○ بلی
- ○ خیر

۱۳. ما می‌دانیم که برخی افراد در گذشته قربانی حوادث آسیب‌زا شده‌اند که ممکن است کنار آمدن با مشکلات جدید را برایشان ساده‌تر یا سخت‌تر کند.

پیش از همه‌گیری کووید-۱۹ هرگز شاهد هیچ‌یک از موارد زیر بوده‌اید؟ یا خودتان هیچ‌کدام را تجربه کرده‌اید:
(لطفاً گزینه‌های انتخابی را تیک بزنید)

- ☐ بلایای طبیعی (مانند سیل، زمین لرزه و از این قبیل موارد)
- ☐ زندگی در یک منطقه جنگی یا در معرض درگیری نظامی
- ☐ بدبیاری دوران کودکی پیش از ۱۶ سالگی(نظیر عدم رسیدگی مناسب، قلدری، آزار جسمی یا جنسی)
- ☐ آزار جسمی یا جنسی پس از ۱۶ سالگی
- ☐ حادثه فیزیکی جدی
- ☐ سایر – لطفاً بگویید چه بوده است (اختیاری) ____
- ☐ هیچ‌کدام از موارد بال

Covid Psychosocial Impacts Scale

تاثیرات شخصی

لطفا در صورتی که هر یک از موارد زیر در مواجهه با کووید-۱۹ برای شما اتفاق افتاده است به صورت بلی/خیر پاسخ دهید.
اگر پاسخ شما به هر موردی بلی است لطفا میزان استرسی که آن مورد برای شما داشته است را بر روی طیف مربوط علامت بزنید.

۱. آیا شما به لحاظ شرایط خاصی دارید که ممکن است شما را دربرابر کووید-۱۹ آسیب‌پذیر سازد؟

- **خیر** ۰
- **بلی** هیچ استرسی ۱
- **بلی** استرس کم ۲
- **بلی** استرس متوسط ۳
- **بلی** استرس زیاد ۴
- **بلی** استرس خیلی زیاد ۵

۲. هیچ‌وقت احساس کرده‌اید که در خطر تماس با اشخاص آلوده به کووید-۱۹ قرار دارید؟

- **خیر** ۰
- **بلی** هیچ استرسی ۱
- **بلی** استرس کم ۲
- **بلی** استرس متوسط ۳
- **بلی** استرس زیاد ۴
- **بلی** استرس خیلی زیاد ۵

۳. فکر می‌کنید به کووید-۱۹ آلوده شده‌اید؟

- **خیر** ۰
- **بلی** هیچ استرسی ۱
- **بلی** استرس کم ۲
- **بلی** استرس متوسط ۳

- بلی استرس زیاد ۴
- بلی استرس خیلی زیاد ۵

۳الف. آیا تست کووید-۱۹ مثبت داشته‌اید؟

- خیر ۰
- بلی هیچ استرسی ۱
- بلی استرس کم ۲
- بلی استرس متوسط ۳
- بلی استرس زیاد ۴
- استرس خیلی زیاد

۳ب. آیا در حال حاضر نشانگان آلودگی به کووید-۱۹ دارید؟

- خیر ۰
- بلی هیچ استرسی ۱
- بلی استرس کم ۲
- بلی استرس متوسط ۳
- بلی استرس زیاد ۴
- بلی استرس خیلی زیاد ۵

تاثیرات خانوادگی

در صورتی که هر یک از موارد زیر برای اعضای خانواده شما در مواجهه با کووید-۱۹ اتفاق افتاده است لطفا با بلی/خیر پاسخ دهید.
اگر پاسخ شما به هر موردی بلی است، لطفا میزان استرسی که آن مورد برای شما داشته را بر روی طیف مربوط علامت بزنید.

۱. آیاهیچیک از اعضای درجه یک خانواده شما شرایط خاص دارد که آنان را نسبت به کووید-۱۹ آسیب‌پذیر بسازد؟

- خیر ۰
- بلی هیچ استرسی ۱

- بلی استرس کم ۲
- بلی استرس متوسط ۳
- بلی استرس زیاد ۴
- بلی استرس خیلی زیاد ۵

۲. آیا احساس می‌کنید هیچ‌یک از اعضای درجه یک خانواده شما به خاطر کارش در خطر تماس با افراد آلوده به کووید-۱۹ قرار دارد؟

- خیر ۰
- بلی هیچ استرسی ۱
- بلی استرس کم ۲
- بلی استرس متوسط ۳
- بلی استرس زیاد ۴
- بلی استرس خیلی زیاد ۵

۳. آیا فکر می‌کنید هیچ‌یک از اعضای درجه یک خانواده شما به کووید-۱۹ آلوده شده باشد؟

- خیر ۰
- بلی هیچ استرسی ۱
- بلی استرس کم ۲
- بلی استرس متوسط ۳
- بلی استرس زیاد ۴
- بلی استرس خیلی زیاد ۵

۳الف. هیچ‌یک از اعضای درجه یک خانواده شما تست کووید-۱۹ مثبت داشته است؟

- خیر ۰

- بلی هیچ استرسی ۱
- بلی استرس کم ۲
- بلی استرس متوسط ۳
- بلی استرس زیاد ۴
- بلی استرس خیلی زیاد ۵

۳ب. هیچ‌یک از اعضای درجه یک خانواده شما در حال حاضر نشانگان آلودگی به کووید-۱۹ دارد؟

- خیر ۰
- بلی هیچ استرسی ۱
- بلی استرس کم ۲
- بلی استرس متوسط ۳
- بلی استرس زیاد ۴
- بلی استرس خیلی زیاد ۵

۳ج. هیچ‌یک از اعضای درجه یک خانواده شما در اثر آلودگی به کووید-۱۹ درگذشته است؟

- خیر ۰
- بلی هیچ استرسی ۱
- بلی استرس کم ۲
- بلی استرس متوسط ۳
- بلی استرس زیاد ۴
- بلی استرس خیلی زیاد ۵

۴. آیا هیچ‌یک از اعضای درجه یک خانواده شما در خارج از کشور هست که نگران باشید در خطر آلودگی به کووید-۱۹ باشد؟

- خیر ۰

Tanveer S, *et al. BMJ Open* 2023; 13:e067886. doi: 10.1136/bmjopen-2022-067886

- بلی هیچ استرسی ۱
- بلی استرس کم ۲
- بلی استرس متوسط ۳
- بلی استرس زیاد ۴
- بلی استرس خیلی زیاد ۵

۵. آیا هیچ‌یک از اعضای درجه یک خانواده شما در خارج از کشور هست که قادر به دیدن آنان نباشید یا آنان قادر به دیدار شما نباشند؟

- خیر ۰
- بلی هیچ استرسی ۱
- بلی استرس کم ۲
- بلی استرس متوسط ۳
- بلی استرس زیاد ۴
- بلی استرس خیلی زیاد ۵

کار و کووید

در صورتی که هر یک از موارد زیر در مواجهه با کاوید ۱۹ برای شما یا برای هر یک از افراد خانوار شما (یعنی افرادی که با شما زیر یک سقف زندگی می‌کنند) اتفاق افتاده است، لطفا به صورت بلی/خیر/نامربوط پاسخ دهید.
اگر پاسخ شما به هر موردی بلی است لطفا میزان استرسی که آن مورد برای شما داشته را بر روی طیف مربوط علامت بزنید.

۱ .آیا شما یا هیچ‌یک از اعضای خانوارتان در مشاغلی که ضروری (مثلا در بخش خدمات درمانی، انتظامات، خدمات اضطراری، ارائه‌دهنده کالاها یا خدمات حیاتی) محسوب شده شاغل است؟

- خیر ۰
- بلی هیچ استرسی ۱
- بلی استرس کم ۲
- بلی استرس متوسط ۳
- بلی استرس زیاد ۴

- بلی استرس خیلی زیاد ۵

۲. آیا شما یا هیچ‌یک از اعضای خانوارتان تغییر عمده‌ای در ساعات کارش داشته است؟

- **خیر ۰**
- **بلی** هیچ استرسی ۱
- **بلی** استرس کم ۲
- **بلی** استرس متوسط ۳
- **بلی** استرس زیاد ۴
- **بلی** استرس خیلی زیاد ۵

۳. آیا شما یا هیچ‌یک از اعضای خانوارتان مجبور شده است که محل کارش را به خانه تغییر دهد؟

- **خیر ۰**
- **بلی** هیچ استرسی ۱
- **بلی** استرس کم ۲
- **بلی** استرس متوسط ۳
- **بلی** استرس زیاد ۴
- **بلی** استرس خیلی زیاد ۵

۴. آیا شما یا هیچ‌یک از اعضای خانوارتان مجبور شده است که نوع کارش را تغییر بدهد؟

- **خیر ۰**
- **بلی** هیچ استرسی ۱
- **بلی** استرس کم ۲
- **بلی** استرس متوسط ۳
- **بلی** استرس زیاد ۴

Tanveer S, *et al. BMJ Open* 2023; 13:e067886. doi: 10.1136/bmjopen-2022-067886

بلی استرس خیلی زیاد ۵

۵. آیا شما یا هیچ‌یک از افراد خانوار شما از کار بی‌کار شده‌اید؟

- **نامربوط**
- **خیر ۰**
- **بلی** هیچ استرسی ۱
- **بلی** استرس کم ۲
- **بلی** استرس متوسط ۳
- **بلی** استرس زیاد ۴
- **بلی** استرس خیلی زیاد ۵

۶. آیا کسب‌وکار شما یا یکی از افراد خانوار شما یا کسب‌وکاری که شما یا یکی از افراد خانوار شما در آن کار می‌کنید در معرض تعطیلی قرار گرفته است؟

- **نامربوط**
- **خیر ۰**
- **بلی** هیچ استرسی ۱
- **بلی** استرس کم ۲
- **بلی** استرس متوسط ۳
- **بلی** استرس زیاد ۴
- **بلی** استرس خیلی زیاد ۵

درآمد

در صورتی که هر یک از موارد زیر برای خانوار شما در مواجهه با کووید-۱۹ اتفاق افتاده است لطفا با بلی/خیر پاسخ بدهید.
اگر پاسخ شما به هر موردی بلی است، لطفا میزان استرسی که آن مورد برای شما داشته را بر روی طیف مربوط علامت بزنید.

۱. خانوار شما از حیث مالی تنگنای عمده‌ای داشته است؟

- **خیر ۰**

- بلی هیچ استرسی ۱
- بلی استرس کم ۲
- بلی استرس متوسط ۳
- بلی استرس زیاد ۴
- بلی استرس خیلی زیاد ۵

۲. آیا خانوار در پرداخت قبوض (برق، تلفن و از این قبیل) یا خرید اقلام غذایی بطور جدی دچار مشکل شده است؟

- خیر ۰
- بلی هیچ استرسی ۱
- بلی استرس کم ۲
- بلی استرس متوسط ۳
- بلی استرس زیاد ۴
- بلی استرس خیلی زیاد ۵

روابط

لطفا در صورتی که هر یک از موارد زیر در مواجهه با کووید-۱۹ برای شما اتفاق افتاده است به صورت بلی/خیر پاسخ دهید.
اگر پاسخ شما به هر موردی بلی است لطفا میزان استرسی که آن مورد برای شما داشته را بر روی طیف مربوط علامت بزنید.
در مواجهه با کووید-۱۹
۱. آیا کووید-۱۹ مشکلات و تنش‌ها را در خانواده افزایش داده است؟

- خیر ۰
- بلی هیچ استرسی ۱
- بلی استرس کم ۲
- بلی استرس متوسط ۳
- بلی استرس زیاد ۴
- بلی استرس خیلی زیاد ۵

در مواجهه با کووید-۱۹
۲. آیا کووید-۱۹ تماس (فیزیکی یا برخط/آنلاین) شما را با دوستان کاهش داده است؟

- **خیر** ۰
- **بلی** هیچ استرسی ۱
- **بلی** استرس کم ۲
- **بلی** استرس متوسط ۳
- **بلی** استرس زیاد ۴
- **بلی** استرس خیلی زیاد ۵

تغییر رفتارها و رویه‌های روزانه

لطفا در صورتی که هر یک از موارد زیر در مواجهه با کووید-۱۹ برای شما اتفاق افتاده است به صورت بلی/خیر پاسخ دهید.
اگر پاسخ شما به هر موردی بلی است لطفا میزان استرسی که آن مورد برای شما داشته را بر روی طیف مربوط علامت بزنید.

در مواجهه با کووید-۱۹
۱. همه‌گیری کووید-۱۹ منجر به تغییراتی در روال عادی زندگی روزمره شما (برای مثال، خواب، ورزش، اوقات فراغت، فعالیت‌های تفریحی یا سرگرمی) گشته است؟

- **خیر** ۰
- **بلی** هیچ استرسی ۱
- **بلی** استرس کم ۲
- **بلی** استرس متوسط ۳
- **بلی** استرس زیاد ۴
- **بلی** استرس خیلی زیاد ۵

در مواجهه با کووید-۱۹
۲. آیا کووید-۱۹ موجب شده است هنگام نیاز به خدمات بهداشتی و درمانی برای خودتان یا هر یک از اعضای خانواده، اقدامی نکنید یا با تاخیر اقدام کنید؟

- **خیر** ۰

- **بلی** هیچ استرسی ۱
- **بلی** استرس کم ۲
- **بلی** استرس متوسط ۳
- **بلی** استرس زیاد ۴
- **بلی** استرس خیلی زیاد ۵

در مواجهه با کووید-۱۹
۳. آیا این شرایط مشارکت شما را در فعالیت‌های دینی (از طریق حضور فیزیکی یا برخط/آنلاین) کاهش داده است؟

- **نامربوط**
- **خیر ۰**
- **بلی** هیچ استرسی ۱
- **بلی** استرس کم ۲
- **بلی** استرس متوسط ۳
- **بلی** استرس زیاد ۴
- **بلی** استرس خیلی زیاد ۵

در مواجهه با کووید-۱۹
۴. آیا تغییر عمده دیگری در عادات زندگی شما روی داده است؟

- **خیر ۰**
- **بلی (لطفا بفرمایید چه تغییر یا تغییراتی)**۱ ____________________

اگر پاسخ شما به هر موردی بلی است لطفا میزان استرسی که آن مورد برای شما داشته را بر روی طیف مربوط علامت بزنید.

- هیچ استرسی ۱
- استرس کم ۲
- استرس متوسط ۳

○ استرس زیاد ۴

○ استرس خیلی زیاد ۵

قرار گرفتن در معرض اطلاعات درباره کاوید-۱۹

۱. در رابطه با کووید-۱۹، از کدام منبع اطلاعاتی استفاده می‌کنید؟

- ▢ اخبار
- ▢ فیس‌بوک / توییتر
- ▢ وب‌سایت‌های بهداشتی
- ▢ گپ و گفت با دیگران
- ▢ منابع دیگر (لطفا مشخص فرمایید)____

۲. اخبار کووید-۱۹ چقدر موجب استرس شما می‌شود؟

○ هیچ استرسی ۱

○ استرس کم ۲

○ استرس متوسط ۳

○ استرس زیاد ۴

○ استرس خیلی زیاد ۵

تغییرات از زمان کووید-۱۹

لطفا برای موارد زیر میزان استرس‌تان را بر روی مقیاس زیر مشخص کنید.

مقایسه حال حاضر با پیش از همه‌گیری کووید-۱۹ ....

۱. سطح استرس خود را چطور ارزیابی می‌کنید؟

○ استرس بسیار کمتر ۱

○ استرس کمتر ۲

○ تقریبا به همان میزان ۳

○ استرس بیشتر ۴

○ استرس بسیار بیشتر ۵

Tanveer S, *et al. BMJ Open* 2023; 13:e067886. doi: 10.1136/bmjopen-2022-067886

مقایسه حال حاضر با پیش از همه‌گیری کووید-۱۹....
۲. بطور کلی سطح استرس در خانوار را چطور ارزیابی می‌کنید؟

- ○ استرس بسیار کمتر ۱
- ○ استرس کمتر ۲
- ○ تقریبا به همان میزان ۳
- ○ استرس بیشتر ۴
- ○ استرس بسیار بیشتر ۵

مقایسه حال حاضر با پیش از همه‌گیری کووید-۱۹.....
۳. سلامت روانی خود را چطور ارزیابی می‌کنید؟

- ○ خیلی بهتر ۱
- ○ بهتر ۲
- ○ تقریبا همانطور که بود ۳
- ○ بدتر ۴
- ○ خیلی بدتر ۵

مقایسه حال حاضر با پیش از همه‌گیری کووید-۱۹...
۴. بطور کلی سلامت روانی اعضای خانوار را چطور ارزیابی می‌کنید؟

- ○ خیلی بهتر ۱
- ○ بهتر ۲
- ○ تقریبا همانطور که بود ۳
- ○ بدتر ۴
- ○ خیلی بدتر ۵

Tanveer S, *et al. BMJ Open* 2023; 13:e067886. doi: 10.1136/bmjopen-2022-067886

K-10

در چهار هفته گذشته چه حس و حالی داشته اید؟

	**هیچگاه** ۱	**به ندرت** ۲	**گاهی اوقات** ۳	**بیشتر وقت ها** ۳	**همیشه** ۵
۱. در چهار هفته گذشته چقدر بی‌دلیل احساس خستگی کرده اید؟	○	○	○	○	○
۲. در چهار هفته گذشته چقدر احساس عصبی بودن کرده‌اید؟	○	○	○	○	○
۳. در چهار هفته گذشته هیچگاه شده است آنقدر عصبی باشید که هیچ چیز نتواند شما را آرام کند؟	○	○	○	○	○
۳. در چهار هفته گذشته چقدر احساس نومیدی کرده‌اید؟	○	○	○	○	○
۵. در چهار هفته گذشته چقدر احساس ناآرامی و بی‌قراری کرده‌اید؟	○	○	○	○	○
٦. در چهار هفته گذشته هیچگاه شده است آنقدر احساس ناآرامی کنید که نتوانید بی‌حرکت یک جا بنشینید؟	○	○	○	○	○
٧. در چهار هفته گذشته چقدر احساس افسردگی کرده‌اید؟	○	○	○	○	○
۸. در چهار هفته گذشته چقدر احساس کرده‌اید که همه کارها را دارید به زور انجام می‌دهید؟	○	○	○	○	○
۹. در چهار هفته گذشته چقدر احساس کرده‌اید که چنان غمگینید که هیچ چیز نمی‌تواند شما را سر حال بیاورد؟	○	○	○	○	○
۱۰. در چهار هفته گذشته چقدر احساس بی‌ارزش بودن کرده ایدِ؟	○	○	○	○	○

PCL-5

فهرست زیر حاوی مشکلاتی است که بعضی از افراد در مواجهه با کووید-۱۹ تجربه کرده‌اند .لطفاً هر مشکل را با دقت بخوانید و سپس مشخص کنید که در یک ماه گذشته چقدر از آن مشکل رنجیده‌اید.

درماه گذشته چقدر موارد زیر شما را آزرده است؟

	اصلا ۰	بسیار کم ۱	متوسط ۲	زیاد ۳	بسیار زیاد ۴
۱- خاطرات تکراری، آزاردهنده و ناخواسته از آن تجربه استرس‌زا داشته‌اید؟	○	○	○	○	○
۲- کابوس‌های تکراری و آزاردهنده از آن تجربه استرس‌زا دیده اید؟	○	○	○	○	○
۳- احساس و عمل ناگهانی داشته‌اید به طوری که انگار آن تجربه استرس‌زا دوباره اتفاق افتاده باشد (در حالی که شما انگار واقعا آنجا حضور داشته‌اید و تجربه‌اش کرده‌اید)؟	○	○	○	○	○
۴- وقتی که یک اتفاق، آن تجربه استرس‌زا را در ذهن شما تداعی کرده احساس ناراحتی زیادی داشته‌اید؟	○	○	○	○	○
۵- هر گاه اتفاق یا چیزی، آن تجربه استرس‌زا را در ذهن شما تداعی کرده، واکنش فیزیکی شدیدی (مثل تپش قلب، مشکل تنفس و عرق کردن) داشته‌اید؟	○	○	○	○	○
٦- از خاطرات، افکار و احساسات مرتبط با آن تجربه استرس زا دوری کرده‌اید؟	○	○	○	○	○
٧- از نشانه‌های یادآور آن تجربه استرس‌زا (مثل اشخاص، مکان ها، گفت‌وگوها، فعالیت‌ها، اشیا و یا موقعیت‌ها) دوری کرده‌اید؟	○	○	○	○	○
۸- بخش‌های مهم آن تجربه استرس‌زا را به سختی به یاد می آورید؟	○	○	○	○	○
۹- در مورد خودتان، بقیه مردم و یا جهان، عقاید خیلی منفی (مثل اینکه من بد هستم، یا مشکل جدی دارم، یا به هیچ کس نمی شود اعتماد کرد، یا جهان خیلی خطرناک است) داشته‌اید؟	○	○	○	○	○
۱۰- خود یا دیگری را در آن تجربه استرس‌زا و یا اتفاقات بعد از آن مقصر دانسته‌اید؟	○	○	○	○	○
۱۱- احساس‌های به شدت منفی مانند ترس، وحشت، خشم، گناه و خجالت داشته‌اید؟	○	○	○	○	○
۱۲- علاقه‌تان به فعالیتی که قبلا از آن لذت می‌بردید از بین رفته است؟	○	○	○	○	○
۱۳- احساس فاصله گرفتن و قطع ارتباط با افراد دیگر را داشته اید؟	○	○	○	○	○

Tanveer S, *et al. BMJ Open* 2023; 13:e067886. doi: 10.1136/bmjopen-2022-067886

۱۴- در تجربه کردن احساسات مثبت و خوب دچار مشکل شده اید؟ مثل ناتوانی در احساس شادی یا احساس دوست داشتن نزدیکان؟	○	○	○	○	○
۱۵- بدخلق و تحریک‌پذیر، دارای خشم شدید و رفتاری تهاجمی بوده‌اید؟	○	○	○	○	○
۱۶- بیش از حد ریسک‌پذیر بوده و یا کارهایی را که ممکن بود به شما آسیب برساند انجام داده‌اید؟	○	○	○	○	○
۱۷- بیش از حد محتاط «یا مراقب و یا گارد گرفته» بوده‌اید؟	○	○	○	○	○
۱۸- مضطرب بوده و به راحتی از جا می‌پریدید؟	○	○	○	○	○
۱۹- به سختی تمرکز داشته‌اید؟	○	○	○	○	○
۲۰- بد خوابیده و خواب خوب و سنگینی نداشته‌اید؟	○	○	○	○	○

WHO-5

در دو هفته گذشته چه حس و حالی داشته‌اید؟

طی ۲ هفته گذشته	تمام اوقات ۵	بیشتر اوقات ۴	کمی بیشتر از نیمی از اوقات ۳	کمی کمتر از نیمی از اوقات ۲	بعضی وقت ها ۱	هیچ وقت ۰
۱. شاد و خوش‌خلق بوده‌ام .	○	○	○	○	○	○
۲. احساس آرامش و راحتی داشته‌ام .	○	○	○	○	○	○
۳. احساس می‌کنم فعال و پرانرژی بوده‌ام .	○	○	○	○	○	○
۴. تر و تازه و سرحال بیدار شده‌ام .	○	○	○	○	○	○
۵. زندگی روزمره‌ام مملو از چیزهای مورد علاقه‌ام بوده است .	○	○	○	○	○	○

Tanveer S, *et al. BMJ Open* 2023; 13:e067886. doi: 10.1136/bmjopen-2022-067886

BMJ Publishing Group Limited (BMJ) disclaims all liability and responsibility arising from any reliance placed on this supplemental material which has been supplied by the author(s)

PTGI

هر یک از عبارات زیر به وقوع تغییری در زندگی شما اشاره دارد .با استفاده از مقیاس ارائه شده نشان دهید که آن تغییر تا چه اندازه در مواجهه با کووید-۱۹ حادث شده است.

	**اصلاً هیچ** ۰	**خیلی کم** ۱	**تا حدودی** ۲	**به طور متوسط** ۳	**تقریبا زیاد** ۴	**بسیار زیاد** ۵
۱. من اولویت‌هایم را درباره اینکه چه چیز در زندگی مهم است تغییر دادم.	○	○	○	○	○	○
۲. من برای زندگی خودم بیشتر ارزش قائلم.	○	○	○	○	○	○
۳. علائق جدیدی پیدا کردم.	○	○	○	○	○	○
۴. بیشتر احساس می‌کنم به خودم متکی ام.	○	○	○	○	○	○
۵. درک بهتری از موضوعات معنوی دارم.	○	○	○	○	○	○
٦. هر چه بیشتر احساس می کنم که می‌توانم در زمان گرفتاری‌ها روی مردم حساب کنم.	○	○	○	○	○	○
۷. مسیر جدیدی برای زندگی‌ام ایجاد کردم.	○	○	○	○	○	○
۸. با دیگران احساس نزدیکی بیشتری می‌کنم.	○	○	○	○	○	○
۹. بیشتر تمایل دارم که ابراز احساسات کنم.	○	○	○	○	○	○
۱۰. به خوبی می‌دانم که می‌توانم از پس سختی‌ها برآیم.	○	○	○	○	○	○
۱۱. می‌توانم کارهای بهتری در زندگی‌ام انجام دهم.	○	○	○	○	○	○
۱۲. بهتر می‌توانم بپذیرم که مسائل چگونه حل می‌شوند.	○	○	○	○	○	○
۱۳. بهتر می توانم قدر هر روز را بدانم.	○	○	○	○	○	○

Tanveer S, *et al. BMJ Open* 2023; 13:e067886. doi: 10.1136/bmjopen-2022-067886

۱۴. فرصت‌های جدیدی پدید آمده‌اند که در غیر این صورت هرگز پیش نمی آمدند.	◯	◯	◯	◯	◯	◯
۱۵. حس دلسوزی بیشتری نسبت به دیگران دارم.	◯	◯	◯	◯	◯	◯
۱٦. برای [حفظ] روابطم بیشتر تلاش می‌کنم.	◯	◯	◯	◯	◯	◯
۱۷. بیشتر احتمال دارد تلاش کنم چیزهایی که باید عوض شوند را عوض کنم.	◯	◯	◯	◯	◯	◯
۱۸. من ایمان مذهبی قوی‌تری دارم.	◯	◯	◯	◯	◯	◯
۱۹. کشف کردم از آنچه که فکرش را می‌کردم قوی‌ترم.	◯	◯	◯	◯	◯	◯
۲۰. کلی درباره اینکه.	◯	◯	◯	◯	◯	◯
۲۱. من راحت تر می‌پذیرم که به دیگران نیاز دارم.	◯	◯	◯	◯	◯	◯

Tanveer S, *et al. BMJ Open* 2023; 13:e067886. doi: 10.1136/bmjopen-2022-067886
