# Supplementary material for: Examining the psychosocial impacts of the COVID-19 pandemic: an international cross-sectional study protocol
Source: BMJ Open. 2023 Apr 11;13(4):e067886. doi: 10.1136/bmjopen-2022-067886 (PMC10105919; doi:10.1136/bmjopen-2022-067886)
Supplement: Author's manuscript [file bmjopen-2022-067886.draft_revisions.pdf]

BMJ Open is committed to open peer review. As part of this commitment we make the peer review history of every article we publish publicly available.

When an article is published we post the peer reviewers' comments and the authors' responses online. We also post the versions of the paper that were used during peer review. These are the versions that the peer review comments apply to.

The versions of the paper that follow are the versions that were submitted during the peer review process. They are not the versions of record or the final published versions. They should not be cited or distributed as the published version of this manuscript.

BMJ Open is an open access journal and the full, final, typeset and author-corrected version of record of the manuscript is available on our site with no access controls, subscription charges or pay-per-view fees (http://bmjopen.bmj.com).

If you have any questions on BMJ Open's open peer review process please email
info.bmjopen@bmj.com

BMJ Open

Examining the Psychosocial Impacts of COVID-19 Pandemic: An International Cross-sectional Study Protocol

Journal:	*BMJ Open*
Manuscript ID	bmjopen-2022-067886
Article Type:	Protocol
Date Submitted by the Author:	02-Sep-2022
Complete List of Authors:	tanveer, sandila; University of Otago Christchurch, Department of Psychological Medicine<br>Sulaiman-Hill, Ruqayya; University of Otago Christchurch, Psychological Medicine<br>Porter, Richard; University of Otago<br>Boden, Joseph; University of Otago<br>Beaglehole, Ben; University of Otago, Psychological Medicine<br>Schluter, Philip; University of Canterbury, School of Health Science; University of Queensland, School of Nursing, Midwifery and Social Work<br>Dean, Shaystah; University of Otago Christchurch, Psychological Medicine; University of Otago Wellington Department of Medicine, Psychological Medicine<br>Bell, Romana; University of Otago, Department of Psychological Medicine<br>Al-Hussainni, Wafaa N; Ibn Sina University of Medical and Pharmaceutical Sciences, Basic Sciences Deptartment<br>Amer Nordin, Amer Siddiq; University of Malaya, Psychological Medicine<br>Dinç, Mehmet; Hasan Kalyoncu University, Department of Psychology<br>Khan, Mussarat ; International Islamic University, Department of Psychology<br>Majid Al-Masoodi, Muthana A; Cultural Relations Mustansiryah University, Department of Scholarships<br>Rahajeng, Anggi; Universitas Gadjah Mada<br>Topçu, Feyza; Hasan Kalyoncu University, Department of Psychology<br>Yapan, Saadet; Hasan Kalyoncu University, Department of Psychology<br>Yunianto, Irfan; Faculty of Teacher Training and Education Universitas Ahmad Dahlan<br>Zoellner, Lori; University of Washington<br>Bell, Caroline; University of Otago, Department of Psychological Medicine
Keywords:	MENTAL HEALTH, COVID-19, PUBLIC HEALTH

SCHOLARONE™
Manuscripts

Examining the Psychosocial Impacts of COVID-19 Pandemic: An International Cross-sectional Study Protocol

Corresponding Author

Associate Professor Caroline Bell, Department of Psychological Medicine, University of Otago Christchurch, P.O. Box 4345, Christchurch 8140, New Zealand, caroline.bell@otago.ac.nz

Authors

Dr Sandila Tanveer, Department of Psychological Medicine, University of Otago, Christchurch, New Zealand

Dr Ruqayya Sulaiman-Hill, Department of Psychological Medicine, University of Otago, Christchurch, New Zealand

Professor Richard Porter, Department of Psychological Medicine, University of Otago, Christchurch, New Zealand

Professor Joseph M. Boden, Department of Psychological Medicine, University of Otago, Christchurch, New Zealand

Dr Ben Beaglehole, Department of Psychological Medicine, University of Otago, Christchurch, New Zealand

For peer review only

Professor Philip J. Schluter, University of Canterbury, Christchurch, New Zealand, and University of Queensland, Queensland, Australia

Dr Shaystah Dean, Department of Psychological Medicine, University of Otago, Wellington, New Zealand

Romana Bell, Department of Psychological Medicine, University of Otago, Christchurch, New Zealand

Dr Wafaa N Al-Hussainni, Basic Sciences Deptartment, Ibn Sina University of Medical and Pharmaceutical Sciences, Baghdad, Iraq

Associate Professor Amer Siddiq Amer Nordin, Department of Psychological Medicine, Universiti Malaya, Kuala Lumpur, Malaysia

Dr Mehmet Dinç, Department of Psychology, Hasan Kalyoncu University, Gaziantep, Turkey

Dr Mussarat Jabeen Khan, Department of Psychology, International Islamic University, Islamabad, Pakistan

Dr Muthana A Majid Al-Masoodi, Department of Scholarships and Cultural Relations, Mustansiryah University, Baghdad, Iraq

Anggi Rahajeng, Faculty of Economics and Business, Universitas Gadjah Mada, Yogyakarta, Indonesia.

Dr Feyza Topçu, Department of Psychology, Hasan Kalyoncu University, Gaziantep, Turkey

For peer review only

Dr Saadet Yapan, Department of Psychology, Hasan Kalyoncu University, Gaziantep, Turkey

Dr Irfan Yunianto, Faculty of Teacher Training and Education, Universitas Ahmad Dahlan, Yogyakarta, Indonesia

Professor Lori A Zoellner, Department of Psychology, University of Washington, Seattle, USA

Associate Professor Caroline Bell, Department of Psychological Medicine, University of Otago, Christchurch, New Zealand

Word Count: 2620

Keywords

Psychosocial impacts, mental health, wellbeing, COVID-19, low and middle-income countries, cross-cultural.

For peer review only

ABSTRACT

Introduction

The novel coronavirus disease 2019 (COVID-19) exposed people to significant and prolonged stress. The psychosocial impacts of the pandemic have been well recognised and reported in high-income countries but it is important to understand some of the unique challenges posed by the COVID-19 pandemic in low and middle-income countries (LMICs) where limited international comparisons have been undertaken. This protocol was therefore devised to study the psychosocial impacts of the COVID-19 pandemic in several LMICs using scales that had been designed for or translated for this purpose.

Methods and analysis

This cross-sectional study uses an online survey to administer a novel measure of COVID-specific exposures and stressors alongside established measures of psychological distress, post-traumatic stress, wellbeing, and post-traumatic growth. All study information and self-report measures will be provided in translations as online supplemental files. Participants will include adults aged above 18 from Indonesia, Iraq, Malaysia, Pakistan, Somalia, and Turkey, with a pragmatic target sample size of 500 in each country.

Data will be analysed descriptively on sociodemographic and study variables. In addition, data will be analysed psychometrically (reliability, validity) to assess the suitability of the use of measures in a given context. Lastly, within-subjects and

For peer review only

between-subjects analyses will be carried out using multi-level mixed-effect models to examine associations between study variables and key sociodemographic factors.

Ethics and dissemination

Ethical approval was granted by the Human Ethics Committee, University of Otago, New Zealand (Ref No. 21/102). In addition, international collaborators obtained local authorisation or ethical approval in their respective host universities before data collection commenced.

Registration details

The study is registered with the Clinical Trial Registry (NCT05052333).

Strengths and limitations of this study

- This observational study will make a significant contribution to understanding the psychosocial impacts of the COVID-19 pandemic in LMICs with different pandemic impacts.
- This study will quantify psychometric measures in countries where there is currently limited access to newly developed scales relating to COVID-19 and associated measures of distress in appropriate languages.
- The findings will validate a newly developed scale which was developed specifically to assess the psychosocial impacts of the pandemic in countries with differing pandemic contexts and including LMICs.
- As samples will not be representative of their national populations, the generalisability of the findings and comparability between groups is limited.

For peer review only

For peer review only

INTRODUCTION

The novel coronavirus disease 2019 (COVID-19) exposed people to significant and prolonged stress[1] from often overlapping concerns; health consequences of COVID-19; impacts of public health restrictions (e.g., lockdowns, social distancing, travel bans); vaccination mandates; and associated societal divisions[2]. Different sampling approaches have been utilized to examine the psychosocial impacts of the pandemic. These include convenience[3] and representative[4] sampling; a focus on potentially vulnerable populations such as the health workforce[5-7], or those with pre-existing risk factors such as having a previous history of mental disorder[8-10]; and large-scale cross-sectional[4] and longitudinal[11] study designs.

To date, the majority of the literature examining the psychosocial impacts of COVID-19 is from studies conducted in China, Europe, and the USA. In comparison, studies investigating the psychosocial impacts in other regions, especially in low-and middle-income countries (LMICs) are scarce, despite many of these areas being severely affected by COVID-19. The difficulties in evaluating pandemic-related psychological impacts in these regions are compounded by the lack of standardised measures in the local languages of these countries, the absence of which limits the comparison of results between countries.

Most studies include self-report psychometric measures of wellbeing, psychological distress, depression, anxiety, and measures of post-traumatic stress disorder (PTSD). However, these do not examine the particular impacts of COVID-19-related

For peer review only

stressors[12,13] and there has been a call for novel COVID-19-specific measures to be used alongside established psychometric scales[14]. In response, several COVID-19-specific measures have been developed which vary in focus and length. For example, the Fear of COVID-19 scale assesses fears associated with infection[12], while others examine a wider range of impacts of the pandemic and public health restrictions on physical health, employment and finances, family and social disruption. The latter scales, in attempting to capture the psychosocial impacts of the COVID-19 pandemic, frequently reflect the context and country in which they were developed, limiting their more general applicability (e.g., CRISIS V0.1 with a more US focus[15]). Researchers have also attempted to develop and validate brief screening tools for clinical research and practice, e.g., the 5-item Coronavirus Anxiety Scale[16] and 7-item COVID-19 Anxiety Scale[17]. In addition, while some positive outcomes from the pandemic have been recognised, few studies formally assess these[18-20] which may limit a comprehensive understanding of the psychosocial impacts.

We developed the Covid Psychosocial Impacts Scale (CPIS) to comprehensively assess the impacts of the pandemic including the adverse and positive personal, social, and economic consequences that followed. Importantly, its applicability for use in a range of countries and cultures was considered in the scale development. The questions were modelled on the Social Readjustment Scale[21] to assess exposure to pandemic-related life events (disruptions to personal, family, employment, faith, education, and daily routines); with the participant being asked to

For peer review only

indicate the resultant level of stress related to that event. Questions examining the positive consequences of the COVID-19 pandemic were also included. This approach to scale construction was informed by our experience of research attempting to understand the psychosocial impacts of the Canterbury Earthquakes[22] and the Mosque Attacks in 2019[23]. The CPIS was refined using the 'group mind' process[24], with colleagues from a range of countries and cultures (Bangladesh, Ghana, Pakistan, Turkey) reviewing a draft of the questionnaire, with iterative improvements made based on their comments.

In addition to developing the CPIS, as part of our work with groups impacted by the Mosque Attacks on March 15, 2019, in Christchurch, New Zealand, the research team also translated the CPIS and several standardised measures of wellbeing and distress into several languages (Arabic, Indonesian, Malay, Somali, Turkish, and Urdu). Some of the self-report measures were already available in some languages, but others were translated by the research team. To ensure that translations captured psychometric meanings in a culturally appropriate way, translations were carried out by proficient bilingual Research Assistants and interpreters who were familiar with the content and context, using parallel and back-to-back translations in an iterative process. These translations were then compared for internal consistency and examined for face validity[23].

This protocol describes our cross-sectional observational study to assess the psychosocial impacts of the COVID-19 pandemic in six LMICs (Indonesia, Iraq, Malaysia, Pakistan, Somalia, and Turkey). It utilises an online survey that includes

For peer review only

the novel CPIS and standardised measures of wellbeing, psychological distress, and posttraumatic growth. Our study will increase understanding of the psychosocial impacts of the COVID-19 pandemic in different countries with differing rates of infection, morbidity, and mortality. Findings will also provide psychometric evidence that could be used to standardise instruments and translations. As a result, our research will contribute to a growing body of mental health measures that can be used for cross-cultural comparison.

METHODS AND ANALYSIS

Study design

This is a cross-sectional observational study examining the psychosocial impacts of the COVID-19 pandemic being conducted in Indonesia, Iraq, Malaysia, Pakistan, Somalia, and Turkey. The survey will be administered online in appropriate languages using the Qualtrics web-based survey application.

Measures

The measures have all been translated into the appropriate language for the country in which they will be administered and can be accessed as online supplemental files. They include the following:

Sociodemographic measures

Sociodemographic measures include age, gender, marital status, religion, ethnicity, languages, self-reported English proficiency, the highest level of education, occupation, study or work status, and monthly income (in local currency). Some of

For peer review only

the demographic questions may vary between locations to ensure contextual appropriateness, on the recommendation of the international collaborators. For example, in Turkey, students do not study part-time so that option was removed from the item. Exposure to previous trauma will also be sought to examine the effect of previous trauma on pandemic-related wellbeing. Participants will be asked to select as many as appropriate from a list of; previous exposure to natural disasters; war or military conflict; childhood adversity; physical or sexual assault; and serious physical accident.

Covid Psychosocial Impacts Scale (CPIS)

This is a novel 27-item measure used to examine the psychosocial impacts of the COVID-19 pandemic. The scale asks participants if they have experienced stressors in response to COVID-19 and then to indicate the magnitude of stress on a six-point scale scored from 0 = 'no', 1 = 'yes, no stress at all' to 5 = 'yes, a lot of stress'. An overall pandemic-related stress score can be calculated ranging from 0 to 135.

WHO Well-Being Index (*WHO-5)*

This is a short 5-item standardised measure assessing subjective psychological wellbeing in the previous two weeks[25]. Each item is scored from 0 = 'at no time' to 5 = 'all of the time'. The raw score range is 0–25; this can be multiplied by 4 to give a final score, with 100 representing the highest subjective wellbeing rating.

Kessler-10 (K10)

For peer review only

This is a 10-item standardised measure assessing symptoms of distress and anxiety in the previous 30 days[26] (Kessler et al., 2003). Each item is scored from 1 = ‘none of the time’ to 5 = ‘all of the time’. The raw score can range from 10 to 50; with scores 30 and above indicating severe mental distress[27].

PTSD Checklist for DSM-5 (*PCL-5)*

This is a 20-item standardised measure that examines symptoms of PTSD in the past month anchored in response to COVID-19 as the potentially traumatic event. Each item is scored from 0 = ‘not at all’ to 4 = ‘extremely’; with a severity score range of 0–80[28].

Posttraumatic Growth Inventory (*PTGI)*

This is a 21-item standardised measure that assesses change following exposure to trauma[29], adapted to reflect how change occurred in your life as a result of COVID-19. Each item is scored on 0 = ‘not at all’ to 5 = ‘a lot’. There are five sub-scales (relating to others, new possibilities, personal strength, spiritual change, and appreciation of life).

Language

The survey will be conducted in the local language of each site.

Participants

For peer review only

Participants are adults (aged 18 years and over) who reside in the country of interest. Anyone aged below 18 years and not currently residing in the country of interest is excluded from the study.

Study Status

Different sites are at different stages of project implementation including ethics approval obtained (Pakistan, Somalia), participant recruitment (Iraq, Malaysia) and data collection (Indonesia, Turkey).

Recruitment

The mode of recruitment will vary from country to country, but will generally involve the distribution of the survey link using a participant mailing list or social media list held by each international collaborator on the host site. For instance, in Indonesia, participants were recruited using purposive sampling to make data as representative as possible considering the diverse population of Indonesia which consists of hundreds of ethnic groups, and to cover range of ages and sociodemographic backgrounds. In this respect, snowball approach was also employed through personal and professional connections using social media platforms (WhatsApp, Facebook). In Turkey, the survey link was distributed using convenient sampling among students and they were encouraged to complete the survey and ask their family and friends to do so using a snowball approach.

Procedure

For peer review only

Participants receive a survey link via the recruitment method above. The online survey comprises a participant information sheet, consent form, and questionnaires. If subjects consent, they are asked to complete the online survey (which includes the self-report measures above) using the Qualtrics XM online survey platform. It takes 15-20 minutes to complete and is provided in the appropriate language for the site. Information is provided about local supports that can be accessed if the self-report questions cause distress.

Patient and Public Involvement

The project has been designed with community and general public involvement. Feedback has also been taken on project design from academics and the general public in the locations of interest.

Data management

To ensure the anonymity of the obtained dataset the following standard operating procedures are in place. Where applicable, only the international collaborators hold contact information for their participant pool. Each site has specific guidelines to preserve anonymity specified in their local ethics application. Following collection, data will be saved on the University of Otago, New Zealand Qualtrics platform using an auto-generated non-identifiable letter-number string. Importantly, the obtained data set will be completely anonymous as no identifiable information is obtained in the survey.

Data analysis

For peer review only

Incomplete survey responses will be removed from the analyses. Data will be analysed using SPSS Version 28.0[30] and Stata Release 17[31]. Statistical analyses will be carried out to examine the psychosocial impacts of the COVID-19 pandemic in different countries with differing rates of infection, morbidity, and mortality and provide psychometric evidence that could be used to standardise measures and translations. Reporting will follow 'Strengthening the Reporting of Observational Studies in Epidemiology' (STROBE) guidelines[32].

Sample size estimation

As this is an exploratory study, and because no prevalence of the psychosocial impacts of the COVID-19 pandemic in the general population of the countries of interest exist, no formal sample size calculations were conducted. The final target sample size of n=500 per country was pragmatically selected balancing the competing demands of maximizing statistical power, expedited cost-effective data collection processes, and international reach while simultaneously minimizing institutional and participant burden[33].

Psychometric analyses

Reliability analyses will be carried out on each measure to determine the suitability of its use, employing item-total correlations and Cronbach's α as a measure of internal homogeneity and consistency. Convergent and discriminant validity of CPIS with other measures of wellbeing and distress will be examined using a correlation matrix.

Within-subjects analyses

For peer review only

Descriptive analysis will be carried out on socio-demographics (e.g., age, gender, ethnicity, religion, marital status, the highest level of education, work status, monthly household income, exposure to previous trauma) and study variables (wellbeing, psychological distress, post-traumatic stress, post-traumatic growth and pandemic-related stress) to inform site-specific sociodemographic profile and psychosocial outcomes. To explore the relationship between socio-demographic and study variables, follow-up analyses will be carried out using a linear mixed model (also known as multi-level modelling) to pool predictor variables. This will be carried out for each of the six sites individually.

Between-subjects analyses

To look at empirical distributions across countries, graphing superimposed Cumulative Distribution Function (CDF) will be used. In addition, to understand the role of demographic and study variables, multi-level modelling will be carried out in which participants will be nested within their countries – countries will be treated as random effects to investigate the association between probable distress and wellbeing indicators and sociodemographic and pandemic-related variables. This approach allows individual and country-level factors to be included[13]. Residual diagnostics and model assumptions will also be checked.

Ethics and dissemination

Ethical approval was granted by the Human Ethics Committee, University of Otago, New Zealand (Ref No. 21/102). In addition, international collaborators obtained local

For peer review only

authorisation or ethical approval in their respective host universities before data collection commenced.

DISCUSSION

This is cross-sectional, observational research that examines the psychosocial impacts of the COVID-19 pandemic across six LMICs using a novel Covid Psychosocial Impacts Scale and measures of wellbeing, psychological distress, and post-traumatic growth. Despite LMICs being significantly impacted by COVID-19, few studies have been published from these regions. This study will therefore provide findings to address this gap in the literature. The multi-centre design of the study will also allow comparisons between the empirical distributions across countries by graphing superimposed CDF. Furthermore, by including individual and country-level factors, multi-level modelling will enable us to understand the role of demographic and study variables. The findings have the potential to contribute to the understanding of the psychosocial impacts of the COVID-19 pandemic in countries that have not been well studied and to allow cross-cultural, multi-site comparisons.

The CPIS is a novel scale which comprehensively assesses the psychosocial impacts of the pandemic. The construction of the scale allows an assessment of both the number of exposures to specific pandemic-related life events (disruptions to personal, family, and employment) and the resultant level of stress related to each of these events. Importantly, the applicability of the CPIS for use in a range of countries and cultures was considered in the scale development through the involvement of

For peer review only

international colleagues. The study will also correlate outcomes on the CPIS with other validated measures of wellbeing and psychological distress and provide essential psychometric information on scale reliability and validity reflecting the suitability of its use in varying contexts. This will contribute to a growing body of mental health measures that can be used for cross-cultural comparison. To date, only a few studies have formally assessed potential positive outcomes associated with the pandemic[18-20] which may limit a comprehensive understanding of the psychosocial impacts. The findings from this study will therefore contribute to this developing literature. A contributing factor to the lack of studies in LMIC has been the lack of availability of measures translated into appropriate languages, which this study addresses.

There are, however, limitations associated with the proposed study design. These include the cross-sectional nature of the survey and lack of comparison with pre-pandemic measures. The study design requires access to device (cell phone, tablet, or computer) with Wi-Fi or cellular internet connection to complete the survey. Although this is widely available in all countries, this may introduce bias in subject participation. There are also limitations associated with the non-representative nature of the samples and differences in sampling techniques utilised in different countries which will limit the comparability and generalisability of the findings. These limitations were mitigated by using multi-level modelling techniques to understand country-wise or cross-cultural predictor variables.

Summary

For peer review only

This cross-sectional observational research examines the psychosocial impacts of the COVID-19 pandemic across six LMICs using a novel Covid Psychosocial Impacts Scale and standardised measures examining wellbeing and distress. The findings will contribute to the understanding of the psychosocial impacts of the COVID-19 pandemic and provide psychometric data to standardise the instruments and translations in LMICs. This is a major contribution as there is currently limited access to newly developed scales relating to COVID-19 and associated measures of distress in appropriate languages. As a result, our research will contribute to a growing trend of establishing mental health measures that allow cross-culturally comparisons. However, given samples will likely be non-representative, generalisability of the findings and comparability between groups is limited.

Acknowledgement

We would also like to thank our colleagues from Bangladesh, Ghana, Pakistan, and Turkey on reviewing the draft of novel CPIS. We would like to thank the Research Assistants from the March 15 Project for their work in translating and back translating study materials.

ABBREVIATIONS

CDF: Cumulative Distribution Function

CPIS: Covid Psychosocial Impacts Scale

K10: Kessler 10

For peer review only

LMIC: Low and middle-income countries

PCL-5: PTSD Checklist for DSM-5

PTGI: Post Traumatic Growth Inventory

WHO-5: WHO Well-Being Index

DECLARATIONS

Author Contributions

ST conceived the study, and CB, RS-H, RP JB, BB, PJS and SD contributed to the study design. ST, CB and RCMS-H developed the protocol and selected the measures. ST was responsible for coordinating instrument translation and setting up the online component. ST, RB, and CB drafted the paper and all authors read, critically revised and approved the final version of the manuscript.

Funding Statement

There has been no funding for this study.

Competing Interests Statement

There are no competing interests.

For peer review only

REFERENCES

1 Cénat J, Blais-Rochette C, Kokou-Kpolou C, et al. Prevalence of symptoms of depression, anxiety, insomnia, posttraumatic stress disorder, and psychological distress among populations affected by the COVID-19 pandemic: a systematic review and meta-analysis. *Psychiatry Res* 2021;295:113599. doi:10.1016/j.psychres.2020.113599

2 Généreux M, Schluter P, Hung K, et al. One virus, four continents, eight countries: an interdisciplinary and international study on the psychosocial impacts of the Covid-19 pandemic among adults. *International Journal of Environmental Research and Public Health* 2020;17(22):8390. doi:10.3390/ijerph17228390

3 Akan Y. Development of the "COVID-19 Psychological Impact Scale": a validity and reliability study. *Current Psychology* 2022. doi:10.1007/s12144-022-02760-5

4 Every-Palmer S, Jenkins M, Gendall P, et al. Psychological distress, anxiety, family violence, suicidality, and wellbeing in New Zealand during the COVID-19 lockdown: a cross-sectional study. *PLoS ONE* 2020;15(11):e0241658. doi:10.1371/journal.pone.0241658

5 Bell C, Williman J, Beaglehole B, et al. Challenges facing essential workers: a cross-sectional survey of the subjective mental health and well-being of New Zealand healthcare and 'other' essential workers during the COVID-19 lockdown. *BMJ Open* 2021;11(7):e048107. doi:10.1136/bmjopen-2020-048107

For peer review only

6 Chew N, Lee G, Tan B, et al. A multinational, multicentre study on the psychological outcomes and associated physical symptoms amongst healthcare workers during COVID-19 outbreak. *Brain Behav Immun* 2020;88:559-565. doi:10.1016/j.bbi.2020.04.049

7 Krishnamoorthy Y, Nagarajan R, Saya G, at al. Prevalence of psychological morbidities among general population, healthcare workers and COVID-19 patients amidst the COVID-19 pandemic: a systematic review and meta-analysis. *Psychiatry Re*s 2020;293:113382. doi:10.1016/j.psychres.2020.113382

8 Bell C, Williman J, Beaglehole B, et al. Psychological distress, loneliness, alcohol use and suicidality in New Zealanders with mental illness during a strict COVID-19 lockdown. *Aust N Z J Psychiatry* 2021;56(7):800-810. doi: 10.1177/00048674211034317

9 Iasevoli F, Fornaro M, D'Urso G, et al. Psychological distress in patients with serious mental illness during the COVID-19 outbreak and one-month mass quarantine in Italy. *Psychol Med* 2020;51(6):1054-1056. doi:10.1017/S0033291720001841

10 Van Rheenen T, Meyer D, Neill E, et al. Mental health status of individuals with a mood-disorder during the COVID-19 pandemic in Australia: initial results from the COLLATE project. *J Affect Disord* 2020;275:69-77. doi:10.1016/j.jad.2020.06.037

For peer review only

11 Fernández R, Crivelli L, Guimet N, et al. Psychological distress and mental health trajectories during the COVID-19 pandemic in Argentina: a longitudinal study. *Scientific Reports* 2022;12(1). doi:10.1038/s41598-022-09663-2

12 Ahorsu D, Lin C, Imani V, et al. The Fear of COVID-19 Scale: development and initial validation. *International Journal of Mental Health and Addiction* 2020;20(3):1537-1545. doi:10.1007/s11469-020-00270-8

13 Généreux M, Schluter P, Landaverde E, et al. The evolution in anxiety and depression with the progression of the pandemic in adult populations from eight countries and four continents. *International Journal of Environmental Research and Public Health* 2021;18(9):4845. doi:10.3390/ijerph18094845

14 COVID-Minds. Recommended Scales 2020. https://www.COVIDminds.org/mental-health-measures

15 CRISIS V0.1. The CoRonavIruS health Impact Survey 2020. http://www.crisissurvey.org/

16 Lee S. Coronavirus Anxiety Scale: a brief mental health screener for COVID-19 related anxiety. *Death Stud* 2020;44(7):393-401. doi:10.1080/07481187.2020.1748481

17 Silva W, de Sampaio Brito T, Pereira C. COVID-19 Anxiety Scale (CAS): development and psychometric properties. *Current Psychology* 2020;41(8):5693-5702. doi:10.1007/s12144-020-01195-0

18 COVID-Minds. The psychological impact of COVID-19 2020. https://www.COVIDminds.org/post/february-update-the-psychological-impact-of-COVID-19

19 Beaglehole B, Williman J, Bell C, et al. Thriving in a pandemic: determinants of excellent wellbeing among New Zealanders during the 2020 COVID-19 lockdown; a cross-sectional survey. *PLoS ONE* 2022;17(3):e0262745. doi:10.1371/journal.pone.0262745

20 Jenkins M, Hoek J, Jenkin G, et al. Silver linings of the COVID-19 lockdown in New Zealand. *PLoS ONE* 2021;16(4):e0249678. doi:10.1371/journal.pone.0249678

21 Holmes T, Rahe R. The Social Readjustment Rating Scale. *J Psychosom Res* 1967;11(2):213-218. doi:10.1016/0022-3999(67)90010-4

22 Fergusson D, Boden J, Horwood L, et al. Perceptions of distress and positive consequences following exposure to a major disaster amongst a well-studied cohort. *Aust N Z J Psychiatry* 2014;49(4):351-359. doi:10.1177/0004867414560652

23 Sulaiman-Hill R, Porter R, Tanveer S, et al. Psychosocial impacts on the Christchurch Muslim community following the 15 March terrorist attacks: a mixed-methods study protocol. *BMJ Open* 2021;11(10):e055413. doi:10.1136/bmjopen-2021-055413

For peer review only

24 Theiner G. A beginner's guide to group minds. In: Sprevak M, Kallestrup J, eds. New Waves in Philosophy of Mind. New Waves in Philosophy: London Palgrave Macmillan 2014. doi:10.1057/9781137286734_15

25 Topp C, Østergaard S, Søndergaard S, et al. The WHO-5 Well-Being Index: a systematic review of the literature. *Psychother Psychosom* 2015;84(3):167-176. doi:10.1159/000376585

26 Kessler R, Barker P, Colpe L, et al. Screening for serious mental illness in the general population. *Arch Gen Psychiatry* 2003;60(2):184. doi:10.1001/archpsyc.60.2.184

27 Andrews G, Slade T. Interpreting scores on the Kessler Psychological Distress Scale (K10). *Aust N Z J Public Health* 2001;25(6):494-497. doi:10.1111/j.1467-842x.2001.tb00310.x

28 Weathers FW, Litz BT, Keane TM, et al. The PTSD Checklist for DSM-5 (PCL-5) – Standard [Measurement instrument]. The National Center for PTSD 2013. Available from https://www.ptsd.va.gov/

29 Tedeschi R, Cann A, Taku K, et al. The Posttraumatic Growth Inventory: a revision integrating existential and spiritual change. *J Trauma Stress* 2017;30(1):11-18. doi:10.1002/jts.22155

30 IBM Corp. IBM SPSS Statistics for Windows, Version 28.0. Armonk, NY: IBM Corp 2021.

For peer review only

31 StataCorp. Stata Statistical Software: Release 17. College Station, TX: StataCorp LLC 2021.

32 von Elm E, Altman DG, Egger M, et al. The Strengthening the Reporting of Observational Studies in Epidemiology (STROBE) statement: guidelines for reporting observational studies. *Lancet* 2007;370(9596):1453-7. doi:10.1016/S0140-6736(07)61602-X

33 Pye V, Taylor N, Clay-Williams R, et al. When is enough, enough? understanding and solving your sample size problems in health services research. *BMC Research Notes* 2016;9(1). doi:10.1186/s13104-016-1893-x

For peer review only

Contents

English Survey....5
Information Sheet....5
Consent....6
Sociodemographic Information....7
Covid Psychosocial Impacts Scale....10
Personal Impacts....10
Family Impacts....12
Employment....16
Income....19
Relationships....20
Changes to Daily Routines and Behaviours....21
Exposure to Information about Covid-19....23
General Subjective Rating....23
K-10....26
PCL-5....28
WHO-5....32
PTGI....33
International Study - Arabic....36
Information Sheet....36
Consent Form....37
Sociodemographic Information....38
Covid Psychosocial Impacts Scale (CPIS)....42
الشخصي التأثير....42
الأسرة تأثير....44
العمل ١٩على-كوفيد جائحة تأثير....48
الدخل....51
العلاقات....52
اليومي الروتين و السلوك في تغيرات....53
١٩-كوفيد يخص فيما معلومات....55
١٩-كوفيد جائحة منذ تغيرات....56

For peer review only - http://bmjopen.bmj.com/site/about/guidelines.xhtml
For peer review only

K10 58

PCL-5 59

WHO-5 61

PGTI 62

Helpline Info 64

International Study - Indonesia 65

Information Sheet 65

Consent Form 67

Sociodemographic Information 68

Covid Psychosocial Impacts Scale 72

Dampak terhadap Pribadi 72

Dampak terhadap Keluarga 74

Pekerjaan 77

Pendapatan 80

Hubungan Keluarga 80

Perubahan dalam kegiatan dan perilaku sehari-hari 82

Paparan terhadap informasi mengenai Covid-19 83

Perubahan sejak Covid-19 84

K-10 86

PCL-5 88

WHO-5 93

PTGI 94

Helpline Information 96

International Study - Malaysia 97

Information Sheet 97

Consent Form 99

Sociodemographic Information 100

Covid Psychosocail Impacts Scale 105

Kesan keatas diri sendiri 105

Kesan kepada keluarga 107

Pekerjaan 111

Pendapatan 114

For peer review only - http://bmjopen.bmj.com/site/about/guidelines.xhtml

Perhubungan (bersama insan lain) 116
Perubahan kepada Rutin dan Tingkah Laku Harian 117
Pendedahan kepada maklumat tentang Covid-19 118
Perubahan sejak Covid-19 119
K-10....122
PCL-5 125
WHO-5 129
PTGI 130
Helpline Information 133
International Study - Somalia....134
Information Sheet....134
Consent Form 135
Sociodemographic Information 136
Covid Psychosocial Impacts Scale 141
Saameynta Shaqsiga 141
Sameeynta Qoyska 143
Shaqo 146
Dakhliga 148
Xiriika 149
Isbeddelka ku yimid nolol maalmeedka iyo dabeecadaha 151
Soo bandhigista macluumaadka ku saabsan Covid-19 153
Isbeddelada ku yimid Covid-19 dartiis....153
K-10....155
PCL-5 157
WHO-5 161
PTGI 162
Helpline Information 165
International Study - Turkish 166
Information Sheet....166
Consent Form 168
Sociodemographic Information 169
Covid Psychosocial Impacts Scale 173

For peer review only

For peer review only - http://bmjopen.bmj.com/site/about/guidelines.xhtml

Aile etkisi 175

İş 178

Gelir 181

İlişkiler....................182

Günlük rutin ve davranışlarda değişiklikler....................183

Covid-19 hakkındaki bilgilere maruz kalma 185

Covid-19'dan bu yana değişiklikler 186

K-10....................189

PCL-5....................191

WHO-5 195

PTGI 196

Helpline Information 198

International Study - Pakistan 198

Information Sheet....................199

Consent Form 200

Sociodemographic Information 201

Covid Psychosocial Impacts Scale 206

ذاتی اثرات.................... 206

خاندان والوں پر اثرات....................208

کام.................... 212

آمدن.................... 215

تعلقات....................216

روزمرہ کے معمولات اور طرزعمل میں تبدیلیاں.................... 217

کوویڈ-19 کے بارے میں معلومات....................219

کوویڈ- 19کے بعد ہونے والی تبدیلیاں.................... 220

K-10....................222

PCL-5....................223

WHO-5 225

PGTI 226

Helpline Information 228

For peer review only - http://bmjopen.bmj.com/site/about/guidelines.xhtml

For peer review only

English Survey

Information Sheet

An International Study to Examine the Psychosocial Impacts of the Covid-19 Pandemic

Thank you for showing an interest in this survey. Please read this information sheet carefully before deciding whether or not to participate. If you decide to participate we thank you. If you decide not to take part there will be no disadvantage to you and we thank you for considering our request.

What is the aim of the research?

The aim of the research is to assess the psychosocial impacts of the Covid-19 pandemic.

Who can participate?

Anyone who is aged 18 and above is eligible to participate in this study.

What will participants be asked to do?

Should you agree to take part in this research, you will be asked to complete a brief 15-20 minutes online survey.

What data or Information will be collected and what use will be made of it?

We will collect demographic data and responses to a series of questionnaires. These data will only be accessed by the research team (Department of Psychological Medicine, University of Otago, Christchurch New Zealand).

The data collected will be securely stored in such a way that only those mentioned above will be able to gain access to it. Data obtained as a result of the research will be retained for **at least 5 years** in secure storage.

No material that could personally identify you will be obtained in this survey. Results of this research may be published and will be available in the University of Otago Library (New Zealand).

Can Participants change their mind and withdraw from the survey?

You may withdraw from the survey, before its completion and without any disadvantage to yourself.

Where can one find support? *(This will be country specific)*

What if Participants have any Questions?

If you have any questions about our study, please feel free to contact:

Dr Caroline Bell
Department of Psychological Medicine
Email Address: caroline.bell@otago.ac.nz

Dr Sandila Tanveer
Department of Psychological Medicine
Email Address: sandila.tanveer@otago.ac.nz

For peer review only

Consent

If you agree to take part in this survey please provide consent for all the statements below.
I have read, or have had this read to me in my first language, and I understand the Participant Information Sheet.
I know who to contact if I have any questions about the research in general.
I understand that taking part in this research is voluntary (my choice) and that I may withdraw at any time without any penalty.
I am provided with the necessary information about the helpline in event of feeling distressed during the survey.
I understand that the data will be retained in secure storage for at least five years.
I understand that the results of the survey may be published and will be available in the University of Otago Library (Dunedin, New Zealand).
I understand that my participation in this research is confidential and that no material that could identify me personally will be obtained or used in any reports on this research.

◯ **I agree to take part in the study.**

◯ **I do not agree to take part in the study.**

For peer review only

Sociodemographic Information

1. Gender

◯ Male

◯ Female

◯ Other

◯ Prefer not to say

2. Age (in years) ______________________________

3. Marital status

◯ Single

◯ In a relationship

◯ Married

◯ Separated

◯ Divorced

◯ Widowed

4. What is your religion?

◯ No religion

◯ Islam

◯ Christianity

◯ Hinduism

◯ Buddhism

◯ Other (please specify) ____

For peer review only

For peer review only - http://bmjopen.bmj.com/site/about/guidelines.xhtml

5. What is your ethnic background? (You can mention more than one)

____

6. In your daily life, which languages do you speak fluently?

____

The following two questions ask specifically about your English language ability. 6a. I can understand English well

◯ Strongly disagree 1

◯ Disagree 2

◯ Neither agree nor disagree 3

◯ Agree 4

◯ Strongly agree 5

6b. I speak English well

◯ Not at all 1

◯ A little 2

◯ Somewhat 3

◯ Quite a lot 4

◯ A lot 5

7. What is your highest level of education?

◯ No formal qualification (attended school, ESOL class)

◯ Secondary school (e.g. NCEA, IB diploma, overseas school qualification)

◯ Tertiary qualification (e.g. Certificate, Diploma or Trade qualification - less than 3 year course)

◯ Bachelor degree (3-4 year course)

◯ Post graduate degree (PG diploma, Master, Doctorate)

For peer review only

8. Current study or work status (Please tick whichever ones apply)

- ☐ Full-time student
- ☐ Part-time student
- ☐ Unemployed / Seeking work
- ☐ Full-time paid work
- ☐ Part-time paid work
- ☐ Full-time self employed
- ☐ Part-time self employed
- ☐ On employer paid parental leave
- ☐ Stay-at-home parent (unpaid)
- ☐ Retired
- ☐ Other (Please specify) ______________________________

9. Occupation

____

10. Monthly household income (in local currency)

____

10a. Compared with the average income, how would you rate the income of your household?

- ○ Below average
- ○ Average
- ○ Above average

11. Are you currently in lockdown or stay-at-home order?

- ○ Yes
- ○ No

For peer review only - http://bmjopen.bmj.com/site/about/guidelines.xhtml

12. **Have you had a Covid-19 vaccine**?

- ◯ Yes
- ◯ No

12a. **If not vaccinated, would you like to get the Covid-19 vaccine in the future**?

- ◯ Yes
- ◯ No

13. We realise that some people may have been exposed to other traumatic incidents in the past which could make it easier or more difficult to deal with new problems.

Before the Covid-19 pandemic, have you ever witnessed or experienced any of the following: (Please tick as many as appropriate)

- ☐ Natural disaster (such as a flood, earthquake etc.)
- ☐ Living in a war zone or being exposed to military conflict
- ☐ Childhood adversity before the age of 16 (such as neglect, bullying, physical or sexual assault)
- ☐ Physical or sexual assault after the age of 16
- ☐ Serious physical accident
- ☐ Other – please specify (optional) ____
- ☐ None of the above

For peer review only

For peer review only - http://bmjopen.bmj.com/site/about/guidelines.xhtml

Covid Psychosocial Impacts Scale

Personal Impacts

For the following statements, please respond with Yes / No if this applies to you in response to Covid-19. If Yes, please indicate the amount of stress that this caused you on the scale below.

1. Do you have an underlying health condition that could make you vulnerable to Covid-19?

- **No 0**
- **Yes** No stress at all 1
- **Yes** A little stress 2
- **Yes** Moderately stressed 3
- **Yes** Quite a lot of stress 4
- **Yes** A lot of stress 5

2. Have you felt at risk of being exposed to someone with Covid-19?

- **No** 0
- **Yes** No stress at all 1
- **Yes** A little stress 2
- **Yes** Moderately stressed 3
- **Yes** Quite a lot of stress 4
- **Yes** A lot of stress 5

3. Do you think you have had Covid-19?

- **No** 0
- **Yes** No stress at all 1
- **Yes** A little stress 2
- **Yes** Moderately stressed 3
- **Yes** Quite a lot of stress 4

For peer review only

- ◯ **Yes** A lot of stress 5

3a. Have you had a positive Covid-19 test?

- ◯ **No** 0
- ◯ **Yes** No stress at all 1
- ◯ **Yes** A little stress 2
- ◯ **Yes** Moderately stressed 3
- ◯ **Yes** Quite a lot of stress 4
- ◯ **Yes** A lot of stress 5

3b. Do you have ongoing symptoms of Covid-19?

- ◯ **No** 0
- ◯ **Yes** No stress at all 1
- ◯ **Yes** A little stress 2
- ◯ **Yes** Moderately stressed 3
- ◯ **Yes** Quite a lot of stress 4
- ◯ **Yes** A lot of stress 5

For peer review only

Family Impacts

For the following statements, please respond with Yes / No if this applies to your close family members in response to Covid-19. If Yes, please indicate the amount of stress that this caused you on the scale below.

1. Does any close family member(s) have an underlying health condition that could make them vulnerable to Covid-19?

- ○ **No** 0
- ○ **Yes** No stress at all 1
- ○ **Yes** A little stress 2
- ○ **Yes** Moderately stressed 3
- ○ **Yes** Quite a lot of stress 4
- ○ **Yes** A lot of stress 5

2. Do you feel any close family member(s) is at risk of being exposed to someone with Covid-19?

- ○ **No** 0
- ○ **Yes** No stress at all 1
- ○ **Yes** A little stress 2
- ○ **Yes** Moderately stressed 3
- ○ **Yes** Quite a lot of stress 4
- ○ **Yes** A lot of stress 5

For peer review only

3. Do you think any close family member(s) have had Covid-19?

- **No** 0
- **Yes** No stress at all 1
- **Yes** A little stress 2
- **Yes** Moderately stressed 3
- **Yes** Quite a lot of stress 4
- **Yes** A lot of stress 5

3a. Has any close family member(s) tested positive for Covid-19?

- **No** 0
- **Yes** No stress at all 1
- **Yes** A little stress 2
- **Yes** Moderately stressed 3
- **Yes** Quite a lot of stress 4
- **Yes** A lot of stress 5

3b. Has any close family member(s) have ongoing symptoms of Covid-19?

- **No** 0
- **Yes** No stress at all 1
- **Yes** A little stress 2
- **Yes** Moderately stressed 3
- **Yes** Quite a lot of stress 4
- **Yes** A lot of stress 5

For peer review only

3c. Has any close family member(s) passed away from Covid-19?

- **No** 0
- **Yes** No stress at all 1
- **Yes** A little stress 2
- **Yes** Moderately stressed 3
- **Yes** Quite a lot of stress 4
- **Yes** A lot of stress 5

4. Do you have any close family member(s) overseas who you are worried are at risk of getting Covid-19?

- **No** 0
- **Yes** No stress at all 1
- **Yes** A little stress 2
- **Yes** Moderately stressed 3
- **Yes** Quite a lot of stress 4
- **Yes** A lot of stress 5

5. Do you have any close family member(s) overseas who you are not able to visit or who cannot visit you?

- **No** 0
- **Yes** No stress at all 1
- **Yes** A little stress 2
- **Yes** Moderately stressed 3
- **Yes** Quite a lot of stress 4
- **Yes** A lot of stress 5

For peer review only - http://bmjopen.bmj.com/site/about/guidelines.xhtml

Employment

For the following statements please respond with Yes / No / Not applicable if this applies to you or anyone in your household in response to Covid-19. If Yes, please indicate the amount of stress that this caused you on the scale below.

1. Are you or anyone in your household considered an essential worker (e.g., in healthcare, law enforcement, emergency services, provider of essential goods/services)?

- ◯ **No** 0
- ◯ **Yes** No stress at all 1
- ◯ **Yes** A little stress 2
- ◯ **Yes** Moderately stressed 3
- ◯ **Yes** Quite a lot stress 4
- ◯ **Yes** A lot of stress 5

2. Have you or anyone in your household had a major change in working hours?

- ◯ **No** 0
- ◯ **Yes** No stress at all 1
- ◯ **Yes** A little stress 2
- ◯ **Yes** Moderately stressed 3
- ◯ **Yes** Quite a lot stress 4
- ◯ **Yes** A lot of stress 5

For peer review only

For peer review only - http://bmjopen.bmj.com/site/about/guidelines.xhtml

3. Have you or anyone in your household had to change the place of work to home?

- **No** 0
- **Yes** No stress at all 1
- **Yes** A little stress 2
- **Yes** Moderately stressed 3
- **Yes** Quite a lot stress 4
- **Yes** A lot of stress 5

4. Have you or anyone in your household had to change to a different type of work?

- **No** 0
- **Yes** No stress at all 1
- **Yes** A little stress 2
- **Yes** Moderately stressed 3
- **Yes** Quite a lot stress 4
- **Yes** A lot of stress 5

For peer review only

5. Have you or anyone in your household lost their job?

- **Not applicable** 9
- **No** 0
- **Yes** No stress at all 1
- **Yes** A little stress 2
- **Yes** Moderately stressed 3
- **Yes** Quite a lot stress 4
- **Yes** A lot of stress 5

6. Is a business you or anyone in your household owns or works in under threat of survival?

- **Not applicable** 9
- **No** 0
- **Yes** No stress at all 1
- **Yes** A little stress 2
- **Yes** Moderately stressed 3
- **Yes** Quite a lot stress 4
- **Yes** A lot of stress 5

For peer review only

Income

For the following statements please respond with Yes / No if this applies to your household in response to Covid-19. If Yes, please indicate the amount of stress that this caused you on the scale below.

1. Has your household had a major deterioration in financial circumstances?

- ○ **No** 0
- ○ **Yes** No stress at all 1
- ○ **Yes** A little stress 2
- ○ **Yes** Moderately stressed 3
- ○ **Yes** Quite a lot of stress 4
- ○ **Yes** A lot of stress 5

2. Has your household had a major deterioration in your ability to pay your bills or for food?

- ○ **No** 0
- ○ **Yes** No stress at all 1
- ○ **Yes** A little stress 2
- ○ **Yes** Moderately stressed 3
- ○ **Yes** Quite a lot of stress 4
- ○ **Yes** A lot of stress 5

For peer review only

Relationships

For the following statements please respond with Yes / No if this applies to you in response to Covid-19. If Yes, please indicate the amount of stress that this caused you on the scale below. In response to Covid-19.....

1. Has it increased difficulty and tension in the family?

- **No** 0
- **Yes** No stress at all 1
- **Yes** A little stress 2
- **Yes** Moderately stressed 3
- **Yes** Quite a lot of stress 4
- **Yes** A lot of stress 5

2. Has this decreased your contact with friends (physical or online)?

- **No** 0
- **Yes** No stress at all 1
- **Yes** A little stress 2
- **Yes** Moderately stressed 3
- **Yes** Quite a lot of stress 4
- **Yes** A lot of stress 5

For peer review only

For peer review only - http://bmjopen.bmj.com/site/about/guidelines.xhtml

Changes to Daily Routines and Behaviours

For the following statements please respond with Yes / No / Not applicable, if this applies to you in response to Covid-19. If Yes, please indicate the amount of stress that this caused you on the scale below. In response to Covid-19…..

1. Has the Covid pandemic led to changes in your daily routine (for example, sleep, exercise, free time, pleasurable activities, or hobbies)?

- **No** 0
- **Yes** No stress at all 1
- **Yes** A little stress 2
- **Yes** Moderately stressed 3
- **Yes** Quite a lot of stress 4
- **Yes** A lot of stress 5

2. Has this caused you to delay / avoid seeking health care when you or a close family member needed it?

- **No** 0
- **Yes** No stress at all 1
- **Yes** A little stress 2
- **Yes** Moderately stressed 3
- **Yes** Quite a lot of stress 4
- **Yes** A lot of stress 5

For peer review only

For peer review only - http://bmjopen.bmj.com/site/about/guidelines.xhtml

3. Has this decreased your involvement with religious activities (in person or on-line)?

- ◯ **Not applicable** 9
- ◯ **No** 0
- ◯ **Yes** No stress at all 1
- ◯ **Yes** A little stress 2
- ◯ **Yes** Moderately stressed 3
- ◯ **Yes** Quite a lot of stress 4
- ◯ **Yes** A lot of stress 5

4. Has there been any other major change in your daily routines.

- ◯ **No** 0
- ◯ **Yes (Please specify)** 1 ______________________________

If Yes, please indicate the amount of stress that this caused you on the scale below.

- ◯ No stress at all 1
- ◯ A little stress 2
- ◯ Moderately stressed 3
- ◯ Quite a lot of stress 4
- ◯ A lot of stress 5

For peer review only

Exposure to Information about Covid-19

1. In regards to Covid-19, what is the source of information for you?
(Please tick as many as appropriate.)

- ☐ News
- ☐ Facebook / Twitter
- ☐ Health websites
- ☐ Word of mouth
- ☐ Others (please specify) ______________________________

2. How much stress is the news about Covid-19 causing you?

- ○ No stress at all 1
- ○ A little stress 2
- ○ Moderately stressed 3
- ○ Quite a lot of stress 4
- ○ A lot of stress 5

For peer review only

General Subjective Rating

For the following statements, please indicate on the given scale. Comparing now with before the Covid-19 pandemic...

1. How would you rate your personal level of stress?

- Considerably less stressed 1
- Somewhat less stressed 2
- About the same 3
- Somewhat more stressed 4
- Considerably more stressed 5

2. How would you rate the overall stress in your household?

- Considerably less stressed 1
- Somewhat less stressed 2
- About the same 3
- Somewhat more stressed 4
- Considerably more stressed 5

3. How would you rate your personal psychological wellbeing?

- Much better 1
- Better 2
- About the same 3
- Worse 4
- Much worse 5

For peer review only

For peer review only - http://bmjopen.bmj.com/site/about/guidelines.xhtml

4. How would you rate the overall psychological wellbeing of your household?

- ◯ Much better 1
- ◯ Better 2
- ◯ About the same 3
- ◯ Worse 4
- ◯ Much worse 5

For peer review only

For peer review only - http://bmjopen.bmj.com/site/about/guidelines.xhtml

K-10

The following ten questions ask about how you have been feeling in the last 4 weeks. For each question, select the option that best describes the amount of time you felt that way.

	**None of the time** 1	**A little of the time** 2	**Some of the time** 3	**Most of the time** 4	**All of the time** 5
1. In the last four weeks, about how often did you feel tired out for no good reason?					
2. In the last four weeks, about how often did you feel nervous?					
3. In the last four weeks, about how often did you feel so nervous that nothing could calm you down?					
4. In the last four weeks, about how often did you feel hopeless?					
5. In the last four weeks, about how often did you feel restless or fidgety?					
6. In the last four weeks, about how often did you feel so restless you could not sit still?					
7. In the last four weeks, about how often did you feel depressed?					
8. In the last four weeks, about how often did you feel that everything was an effort?					
9. In the last four weeks, about how often did you feel so sad that nothing could cheer you up?					

For peer review only

For peer review only - http://bmjopen.bmj.com/site/about/guidelines.xhtml

10. In the last four weeks, about how often did you feel worthless?	○	○	○	○	○

For peer review only

PCL-5

Below is a list of problems which some people have experienced in response to Covid-19. Please read each problem carefully and then indicate how much you have been bothered by that problem in the past month. In the past month, how much were you bothered by:

	Not at all 0	A little bit 1	Moderately 2	Quite a bit 3	Extremely 4
1. Repeated, disturbing, and unwanted memories of the stressful experience?	○	○	○	○	○
2. Repeated, disturbing dreams of the stressful experience?	○	○	○	○	○
3. Suddenly feeling or acting as if the stressful experience were actually happening again (as if you were actually back there reliving it)?	○	○	○	○	○
4. Feeling very upset when something reminded you of the stressful experience?	○	○	○	○	○
5. Having strong physical reactions when something reminded you of the stressful experience (for example, heart pounding, trouble breathing, sweating)?	○	○	○	○	○
6. Avoiding memories, thoughts, or feelings related to the stressful experience?	○	○	○	○	○
7. Avoiding external reminders of the stressful experience (for example, people, places, conversations, activities, objects, or situations)?	○	○	○	○	○
8. Trouble remembering important parts of the stressful experience?	○	○	○	○	○
9. Having strong negative beliefs about yourself, other	○	○	○	○	○

For peer review only

For peer review only - http://bmjopen.bmj.com/site/about/guidelines.xhtml

people, or the world (for example, having thoughts such as: I am bad, there is something seriously wrong with me, no one can be trusted, the world is completely dangerous)?					
10. Blaming yourself or someone else for the stressful experience or what happened after it?	○	○	○	○	○
11. Having strong negative feelings such as fear, horror, anger, guilt, or shame?	○	○	○	○	○
12. Loss of interest in activities that you used to enjoy?	○	○	○	○	○
13. Feeling distant or cut off from other people?	○	○	○	○	○
14. Trouble experiencing positive feelings (for example, being unable to feel happiness or have loving feelings for people close to you)?	○	○	○	○	○
15. Irritable behaviour, angry outbursts, or acting aggressively?	○	○	○	○	○
16. Taking too many risks or doing things that could cause you harm?	○	○	○	○	○
17. Being "super alert" or watchful or on guard?	○	○	○	○	○
18. Feeling jumpy or easily startled?	○	○	○	○	○
19. Having difficulty concentrating?	○	○	○	○	○
20. Trouble falling or staying asleep?	○	○	○	○	○

For peer review only

For peer review only - http://bmjopen.bmj.com/site/about/guidelines.xhtml

For peer review only

For peer review only - http://bmjopen.bmj.com/site/about/guidelines.xhtml

WHO-5

Please indicate for each of the five statements which is closest to how you have been feeling over the last 2 weeks.

	All of the time 5	Most of the time 4	More than half of the time 3	Less than half of the time 2	Some of the time 1	At no time 0
1. I have felt cheerful and in good spirits	○	○	○	○	○	○
2. I have felt calm and relaxed	○	○	○	○	○	○
3. I have felt active and vigorous	○	○	○	○	○	○
4. I woke up feeling fresh and rested	○	○	○	○	○	○
5. My daily life has been filled with things that interest me	○	○	○	○	○	○

For peer review only

For peer review only - http://bmjopen.bmj.com/site/about/guidelines.xhtml

PTGI

Indicate for each of the statements below the degree to which this change occurred in your life as a result of Covid-19, using the following scale.

	Not at all 0	A little bit 1	Somewhat 2	Moderately 3	Quite a bit 4	A lot 5
1. I changed my priorities about what is important in life.	○	○	○	○	○	○
2. I have a greater appreciation for the value of my own life.	○	○	○	○	○	○
3. I developed new interests.	○	○	○	○	○	○
4. I have a greater feeling of self-reliance.	○	○	○	○	○	○
5. I have a better understanding of spiritual matters.	○	○	○	○	○	○
6. I more clearly see that I can count on people in times of trouble.	○	○	○	○	○	○
7. I established a new path for my life.	○	○	○	○	○	○
8. I have a great sense of closeness with others.	○	○	○	○	○	○
9. I am more willing to express my emotions.	○	○	○	○	○	○
10. I know better that I can handle difficulties.	○	○	○	○	○	○
11. I am able to do better things with my life.	○	○	○	○	○	○
12. I am better able to accept the way things work out.	○	○	○	○	○	○

For peer review only

13. I can better appreciate each day.

14. New opportunities are available which wouldn't have been otherwise.

15. I have more compassion for others.

16. I put more effort into my relationships.

17. I am more likely to try to change things which need changing.

18. I have a stronger religious faith.

19. I discovered that I'm stronger than I thought I was.

20. I learned a great deal about how wonderful people are.

21. I better accept needing others.

For peer review only

Remember if you are feeling distressed by any of the content in this survey, please note the following helplines and services.

Local helpline Call: 122

For peer review only - http://bmjopen.bmj.com/site/about/guidelines.xhtml

International Study - Arabic

Information Sheet

دراسة دولية لتحديد الآثار النفسية لجائحة كوفيد-١٩

شكرًا لإبداء اهتمامك بهذه الدراسة. يرجى التمعن بقراءة ورقة معلومات المشارك قبل اتخاذ القرار بالمشاركة أم لا. ستُسعِدنا مشاركتك ولن يكون هناك أي ضرر من عدم المشاركة.

ما هو الهدف من الدراسة؟

تهدف الدراسة الى تقييم الآثار النفسية لجائحة كوفيد.

من يستطيع المشاركة؟

أي شخص يبلغ من العمر 18 عامًا فما فوق مؤهل للمشاركة في هذه الدراسة.

ماذا سيطلب من المشاركين في هذه الدراسة؟

إذا وافقت على المشاركة في هذه الدراسة، فسيُطلب منك إكمال استبيان قصير عبر الإنترنت لمدة 15-20 دقيقة.

ما هي البيانات أو المعلومات التي سيتم جمعها وما الفائدة منها؟

سنجمع البيانات الديموغرافية والردود على سلسلة من الاستبيانات. سيتم الوصول إلى هذه البيانات فقط من قبل فريق البحث (قسم الطب النفسي، جامعة أوتاجو، كرايستشيرش نيوزيلندا).

سيتم تخزين البيانات التي تم جمعها بشكل آمن بحيث لا يتمكن من الوصول إليها إلا المذكورين أدناه. سيتم الإحتفاظ بالبيانات التي تم الحصول عليها من البحث لمدة 5 سنوات على الأقل في مكان آمن.

لن يتم تحديد هويتك في هذه الدراسة.
سيتم نشر نتائج هذا الدراسة وستكون متاحة في مكتبة جامعة أوتاجو (دنيدن، نيوزيلندا).

هل يمكن للمشاركين تغيير رأيهم والانسحاب من الدراسة؟

خط المساعدة المحلي 122

هل لديك أي استفسار حول هذه الدراسة؟

إذا كانت لديك أي أسئلة حول دراستنا، فلا تتردد في الاتصال بـــــــ:

الدكتورة كارولين بيل
قسم الطب النفسي
البريد الإلكتروني caroline.bell@otago.ac.nz

الدكتورة سانديلا تانفير
قسم الطب النفسي
البريد الإلكتروني sandila.tanveer@otago.ac.nz

For peer review only - http://bmjopen.bmj.com/site/about/guidelines.xhtml

Consent Form

إذا كنت توافق على المشاركة في هذه الدراسة، فيرجى تقديم الموافقة على جميع البيانات أدناه.

لقد قرأت، أو تمت قراءة ذلك لي بلغتي الأم، و أفهم ورقة معلومات المشارك.

أعرف من الذي يجب علي الأتصال به إذا كان لديَ أي أسئلة حول الدراسة بشكلٍ عام.

أتفهم أن المشاركة في هذه الدراسة أمر تطوعي) اختياري (و أنه يمكنني الإنسحاب من الدراسة في أي وقت دون عقوبة او جزاء.

لقد تم تزويدي بالمعلومات اللازمة حول خطة المساعدة في حالة الشعور بالضيق أثناء المقابلة.

أتفهم أنه سيتم الاحتفاظ بالبيانات في مكان آمن لمدة خمس سنوات على الأقل.

أتفهم أنه قد يتم الإعلان عن نتائج المشروع وستكون متاحة في مكتبة جامعة أوتاجو (دنيدن، نيوزيلندا).

أتفهم أن مشاركتي في هذه الدراسة سرية و لن يتم استخدام أي مادة، من شأنها أن تحدد شخصيتي، في أي تقارير حول هذه الدراسة.

○ **أوافق على المشاركة في هذه الدراسة.**

○ **لا أرغب في المشاركة في هذه الدراسة.**

For peer review only

Sociodemographic Information

١ـ الجنس

- ذكر
- أنثى
- اخرى
- أفضل عدم الإجابة

٢ـ بالسنوات (الفئة العمرية)

____

٣ـ الحالة الإجتماعية

- أعزب
- مرتبط
- متزوج
- منفصل
- مطلق
- أرمل

For peer review only

For peer review only - http://bmjopen.bmj.com/site/about/guidelines.xhtml

٤- ماهي ديانتك؟

- ليس لدي ديانة
- الإسلام
- المسيحية
- الهندوسية
- البوذية
- أخرى (برجاء الشرح) ____

٥- ما هي أصولك العرقية؟ (يمكن أن تذكر أكثر من واحدة)

____

٦- ماهي اللغات التي تتحدثها بطلاقة في حياتك اليومية؟

____

السؤالان التاليان يحددان مدى قدرتك في اللغة الإنجليزية.
٦أ- يمكنني فهم اللغة الإنجليزية جيداً

- لا أوافق بشدة 1
- لا أوافق 2
- لا اعلم 3
- أوافق 4
- أوافق بشدة 5

For peer review only - http://bmjopen.bmj.com/site/about/guidelines.xhtml

٦ب- يمكنني تحدث الإنجليزية جيداً

- ○ لا على الإطلاق 1
- ○ قليلاً 2
- ○ بقدر معتدل 3
- ○ كثيراً 4
- ○ كثيراً جداً 5

٧- ما هي مؤهلاتك العلمية؟

- ○ لا يوجد مؤهل علمي (التحقت بالمدرسة، فصل تعليم الكبار غير الناطقين بالإنجليزية)
- ○ المرحلة الثانوية (على سبيل المثال، الشهادة الوطنية النيوزيلاندية للتحصيل التعليمي، دبلوم، والمؤهلات المدرسية من خارج نيوزيلاندا)
- ○ مؤهل جامعي (على سبيل المثال، شهادة، دبلوم أو مؤهل تجاري -دورة أقل من 3 سنوات)
- ○ البكالوريوس (دورة 4-3 سنوات)
- ○ الدراسات العليا (دبلوم الدراسات العليا، الماجستير، الدكتوراة)

٨- وضعك الدراسي أو الوظيفي (يرجى وضع علامة على كل الحالات التي تنطبق عليك)

- ▫ يدرس بدوام كامل
- ▫ يدرس بدوام جزئي
- ▫ لا يعمل / يبحث عن عمل
- ▫ يعمل بدوام كامل (وظيفة واحدة او أكثر)
- ▫ يعمل بدوام جزئي (وظيفة واحدة او أكثر)
- ▫ يعمل لحسابة بدوام كامل
- ▫ يعمل لحسابة بدوام جزئي
- ▫ أجازة امومة مدفوعة الأجر
- ▫ رب - ربة منزل (غير مدفوع الأجر)
- ▫ متقاعد
- ▫ أخرى (برجاء الشرح) ______________________________________

For peer review only

For peer review only - http://bmjopen.bmj.com/site/about/guidelines.xhtml

٩ـ الوظيفة

١٠ـ دخل الأسرة الشهري (بالعملة المحلية)

١٠أ- مقارنة بمتوسط الدخل، ما هو تقيمك لدخل أسرتك؟

○ أقل من المتوسط

○ متوسط

○ أعلى من المتوسط

١١ـ هل أنت حالياً في وضع العزل بسبب جائحة كوفيد-١٩؟

○ نعم

○ لا

١٢ـ هل تلقيت مطعوم كوفيد-١٩؟

○ نعم

○ لا

١٢أ- إذا لم تتلقى مطعوم كوفيد-١٩، هل ترغب في الحصول عليه في المستقبل؟

○ نعم

○ لا

For peer review only

For peer review only - http://bmjopen.bmj.com/site/about/guidelines.xhtml

١٣ـ ندرك أن بعض الأشخاص قد تعرضوا إلى أحداث صادمة في الماضي قد تجعل التعامل مع المشكلات الأخرى أسهل أو أصعب في التعامل مع اي مشكلات جديدة. قبل جائحة كوفيد-١٩ هل سبق أن شاهدت أو تعرضت إلى واحدة أو أكثر من هذه المواقف :(يرجى وضع علامة أو اكثر إن وجد)

- ▫ كوارث طبيعية (مثل فيضان، زلزال أو ما إلى ذلك)
- ▫ كنت تعيش في منطقة حروب أو تعرضت إلى نزاع عسكري
- ▫ محنة في الطفولة قبل سن ١٦عاماً (مثل الإهمال، التنمرأو الإعتداء الجنسي)
- ▫ اعتداء بدني أو جنسي بعد سن ١٦عاماً
- ▫ حادث بدني خطير
- ▫ أخرى - برجاء الشرح (أختياري) ____
- ▫ ليس مما سبق

Covid Psychosocial Impacts Scale (CPIS)

التأثير الشخصي

يرجى الإجابة (بنعم / لا) إذا كان لجائحة كوفيد-١٩ أثرعليك من خلال الإسئلة التالية. إذا كانت اجابتك بنعم، يرجى تحديد مقدار الضغط الذي تشعر به من خلال هذا المقياس أدناه.

١ـ هل لديك اي مشاكل صحية تجعلك عرضة لمرض كوفيد-١٩؟

- ◯ **لا 0**
- ◯ نعم لا يوجد ضغط اطلاقاً 1
- ◯ نعم قليلاً 2
- ◯ نعم احيانا 3
- ◯ نعم غالباً جداً 4
- ◯ نعم كثيراً جداً 5

For peer review only - http://bmjopen.bmj.com/site/about/guidelines.xhtml

٢- هل شعرت بخطر التعرض لشخص مصاب بكوفيد-١٩؟

- لا0
- نعم لا يوجد ضغط اطلاقاً1
- نعم قليلاً2
- نعم احيانا3
- نعم غالباً جداً4
- نعم كثيراً جداً5

٣- هل أصبت بمرض كوفيد (كورونا)؟

- لا0
- نعم لا يوجد ضغط اطلاقاً1
- نعم قليلاً2
- نعم احيانا3
- نعم غالباً جداً4
- نعم كثيراً جداً5

٣أ- هل كانت نتيجة فحص أختبار كوفيد-١٩ إيجابية؟

- لا0
- نعم لا يوجد ضغط اطلاقاً1
- نعم قليلاً2
- نعم احيانا3
- نعم غالباً جداً4
- نعم كثيراً جداً5

For peer review only - http://bmjopen.bmj.com/site/about/guidelines.xhtml

٣ب -هل تعاني من أعراض مستمرة بسبب كوفيد-١٩؟

- **لا0**
- نعم لا يوجد ضغط اطلاقاً1
- نعم قليلاً2
- نعم احيانا3
- نعم غالباً جداً4
- نعم كثيراً جداً5

تأثير الأسرة

يرجى الإجابة (بنعم / لا) إذا كان لجائحة كوفيد-١٩ أثرعلى أسرتك من خلال الإسئلة التالية. إذا كانت اجابتك بنعم، يرجى تحديد مقدار الضغط الذي تشعر به من خلال هذا المقياس أدناه.

١- هل يعاني احد أفراد أسرتك من اي مشاكل صحية تجعله عرضة لمرض كوفيد-١٩؟

- **لا0**
- نعم لا يوجد ضغط اطلاقاً1
- نعم قليلاً2
- نعم احيانا3
- نعم غالباً جداً4
- نعم كثيراً جداً5

For peer review only

٢ـ هل تشعر ان احد أفراد أسرتك معرض للاصابة لكوفيد-١٩ من خلال الإختلاط بأحد أفراد العمل؟

- لا0
- نعم لا يوجد ضغط اطلاقاً1
- نعم قليلاً2
- نعم احيانا3
- نعم غالباً جداً4
- نعم كثيراً جداً5

٣- هل أصيب احد أفراد أسرتك بمرض كوفيد-١٩؟

- لا0
- نعم لا يوجد ضغط اطلاقاً1
- نعم قليلاً2
- نعم احيانا3
- نعم غالباً جداً4
- نعم كثيراً جداً5

٣أ- هل كانت نتيجة فحص كوفيد-١٩ إيجابية لأحد أفراد أسرتك؟

- لا0
- نعم لا يوجد ضغط اطلاقاً1
- نعم قليلاً2
- نعم احيانا3
- نعم غالباً جداً4
- نعم كثيراً جداً5

For peer review only

For peer review only - http://bmjopen.bmj.com/site/about/guidelines.xhtml

٣ب - هل أحد أفراد أسرتك مازال يعاني من أعراض كوفيد-١٩؟

- ◯ **لا0**
- ◯ نعم لا يوجد ضغط اطلاقاً1
- ◯ نعم قليلاً2
- ◯ نعم احيانا3
- ◯ نعم غالباً جداً4
- ◯ نعم كثيراً جداً5

٣ج - هل توفى احد أفراد أسرتك كنتيجة لكوفيد-١٩؟

- ◯ **لا0**
- ◯ نعم لا يوجد ضغط اطلاقاً1
- ◯ نعم قليلاً2
- ◯ نعم احيانا3
- ◯ نعم غالباً جداً4
- ◯ نعم كثيراً جداً5

For peer review only

For peer review only - http://bmjopen.bmj.com/site/about/guidelines.xhtml

٤- هل لديك احد من أفراد أسرتك مقيم بالخارج و تشعر بالقلق عليهم من خطر الإصابة بمرض كوفيد-١٩؟

- لا0
- نعم لا يوجد ضغط اطلاقاً1
- نعم قليلاً2
- نعم احيانا3
- نعم غالباً جداً4
- نعم كثيراً جداً5

٥- هل لديك احد من أفراد أسرتك يقيم بالخارج و لا يمكنك زيارتهم و لا يمكنهم زيارتك؟

- لا0
- نعم لا يوجد ضغط اطلاقاً1
- نعم قليلاً2
- نعم احيانا3
- نعم غالباً جداً4
- نعم كثيراً جداً5

For peer review only

تأثير جائحة كوفيد-١٩على العمل

يرجى الإجابة (بنعم / لا / لا أفضل الإجابة) إذا كان لجائحة كوفيد-١٩ أثرعليك او على احد أفراد أسرتك المقيمين معك بالمنزل من خلال الإسئلة التالية. إذا كانت اجابتك بنعم، يرجى تحديد مقدار الضغط الذي تشعر به من خلال هذا المقياس أدناه.

١- هل تعتبر أنت أو احد أفراد أسرتك المقيمين معك بالمنزل من العمال الأساسيين (مثل عمال الرعاية الصحية، شؤون القانون، خدمات الطوارئ أو مزودي السلع و الخدمات الأساسية)؟

- ○ **لا0**
- ○ نعم لا يوجد ضغط اطلاقاً1
- ○ نعم قليلاً2
- ○ نعم احيانا3
- ○ نعم غالباً جداً4
- ○ نعم كثيراً جداً5

٢- هل كان هناك تغير كبير في ساعات العمل لك أو احد أفراد أسرتك المقيمين معك بالمنزل؟

- ○ **لا0**
- ○ نعم لا يوجد ضغط اطلاقاً1
- ○ نعم قليلاً2
- ○ نعم احيانا3
- ○ نعم غالباً جداً4
- ○ نعم كثيراً جداً5

For peer review only

For peer review only - http://bmjopen.bmj.com/site/about/guidelines.xhtml

٣- هل اضطررت أنت أو احد أفراد أسرتك المقيمين معك بالمنزل إلى تغيير مكان العمل للعمل من المنزل؟

- لا0
- نعم لا يوجد ضغط اطلاقاً1
- نعم قليلاً2
- نعم احيانا3
- نعم غالباً جداً4
- نعم كثيراً جداً5

٤- هل اضطررت أنت أو احد أفراد أسرتك المقيمين معك بالمنزل إلى تغيير طبيعة العمل؟

- لا0
- نعم لا يوجد ضغط اطلاقاً1
- نعم قليلاً2
- نعم احيانا3
- نعم غالباً جداً4
- نعم كثيراً جداً5

For peer review only

For peer review only - http://bmjopen.bmj.com/site/about/guidelines.xhtml

٥ -هل فقدت أنت او احد أفراد أسرتك المقيمين معك بالمنزل؟

- لا أفضل الإجابة
- **لا0**
- نعم لا يوجد ضغط اطلاقاً1
- نعم قليلاً2
- نعم احيانا3
- نعم غالباً جداً4
- نعم كثيراً جداً5

٦- هل الأعمال التجارية التي تمتلكها او تعمل بها أنت او احد أفراد أسرتك المقيمين معك بالمنزل أصبحت مهددة بالبقاء؟

- لا أفضل الإجابة
- **لا0**
- نعم لا يوجد ضغط اطلاقاً1
- نعم قليلاً2
- نعم احيانا3
- نعم غالباً جداً4
- نعم كثيراً جداً5

For peer review only

الدخل

يرجى الإجابة (بنعم / لا) إذا حدث ذلك لاحد أفراد أسرتك المقيمين معك بالمنزل كنتيجة لجائحة كوفيد-١٩ من خلال الإسئلة التالية. إذا كانت اجابتك بنعم ، فيرجى تحديد مقدار الضغط الذي تشعر به من خلال هذا المقياس أدناه.

١- هل تعرض أحد من أفراد أسرتك لتدهور كبير في الحالة المالية؟

- **لا0**
- نعم لا يوجد ضغط اطلاقاً1
- نعم قليلاً2
- نعم احيانا3
- نعم غالباً جداً4
- نعم كثيراً جداً5

٢- هل تعرض احد أفراد أسرتك المقيمين لتدهور كبير في عدم القدرة على دفع الفواتير او توفير الإحتياجات الغذائية؟

- **لا0**
- نعم لا يوجد ضغط اطلاقاً1
- نعم قليلاً2
- نعم احيانا3
- نعم غالباً جداً4
- نعم كثيراً جداً5

For peer review only

For peer review only - http://bmjopen.bmj.com/site/about/guidelines.xhtml

العلاقات

يرجى الإجابة (بنعم / لا) إذا كان لجائحة كوفيد-١٩ أثرعليك من خلال الإسئلة التالية.
إذا كانت اجابتك بنعم، يرجى تحديد مقدار الضغط الذي تشعر به من خلال هذا المقياس أدناه. كنتيجة لكوفيد-١٩....
١- هل ادى ذلك لزيادة المشاكل والتوتر داخل العائلة؟

- **لا0**
- نعم لا يوجد ضغط اطلاقاً1
- نعم قليلاً2
- نعم احيانا3
- نعم غالباً جداً4
- نعم كثيراً جداً5

٢- هل ادى ذلك الى انخفاض معدل تواصلك مع أصدقائك سواء تواصل اجتماعياً او من خلال التواصل الإليكتروني؟

- **لا0**
- نعم لا يوجد ضغط اطلاقاً1
- نعم قليلاً2
- نعم احيانا3
- نعم غالباً جداً4
- نعم كثيراً جداً5

For peer review only

For peer review only - http://bmjopen.bmj.com/site/about/guidelines.xhtml

تغيرات في السلوك و الروتين اليومي

يرجى الإجابة (نعم / لا / لا أفضل الإجابة) إذا كان لجائحة كوفيد-١٩ أثرعليك من خلال الإسئلة التالية. إذا كانت اجابتك بنعم، يرجى تحديد مقدار الضغط الذي تشعر به من خلال هذا المقياس أدناه.

١- هل أدت جائحة كوفيد إلى تغير معدل أنشطتك اليومية (على سبيل المثال :معدل النوم، الأنشطة الرياضية، وقت الفراغ أو استمتاعك بالأنشطة أو الهوايات الخاص بك)؟

- ○ **لا0**
- ○ نعم لا يوجد ضغط اطلاقاً1
- ○ نعم قليلاً2
- ○ نعم احيانا3
- ○ نعم غالباً جداً4
- ○ نعم كثيراً جداً5

٢- هل ادى ذلك الى تأخير او تجنب طلب الرعاية الصحية عندما تحتاجها أنت أو أحد أفراد أسرتك؟

- ○ **لا0**
- ○ نعم لا يوجد ضغط اطلاقاً1
- ○ نعم قليلاً2
- ○ نعم احيانا3
- ○ نعم غالباً جداً4
- ○ نعم كثيراً جداً5

For peer review only - http://bmjopen.bmj.com/site/about/guidelines.xhtml

٣- هل ادى ذلك الى انخفاض معدل مشاركتك في الأنشطة الدينية (مثل سواء وجهاً لوجه أو عبر الإنترنت)؟

- لا أفضل الإجابة
- **لا0**
- نعم لا يوجد ضغط اطلاقاً1
- نعم قليلاً2
- نعم احيانا3
- نعم غالباً جداً4
- نعم كثيراً جداً5

٤- هل لديك اى تغيرات كبيرة اخرى في حياتك الروتينية؟

- **لا0**
- نعم (برجاء الشرح)1 ____________________

إذا كانت اجابتك بنعم، يرجى تحديد مقدار الضغط الذي تشعر به من خلال هذا المقياس أدناه.

- لا يوجد ضغط اطلاقاً1
- قليلاً2
- احيانا3
- غالباً جداً4
- كثيراً جداً5

For peer review only

For peer review only - http://bmjopen.bmj.com/site/about/guidelines.xhtml

معلومات فيما يخص كوفيد-١٩

١ـ فيما يخص كوفيد-١٩ ماهو مصدر المعلومات بالنسبة لك؟ (برجاء وضع علامة على أكثر من إختيار.)

- ▫ الأخبار
- ▫ الفيس بوك / تويتر
- ▫ المواقع الصحية المعتمدة
- ▫ كلام شَفهي
- ▫ أخرى (برجاء الشرح) ________________________________

٢ـ ما مدى الضغط النفسي الذي تسببته الأخبار عن كوفيد-١٩؟

- ○ نعم لا يوجد ضغط اطلاقاً 1
- ○ نعم قليلاً 2
- ○ نعم احيانا 3
- ○ نعم غالباً جداً 4
- ○ نعم كثيراً جداً 5

For peer review only

For peer review only - http://bmjopen.bmj.com/site/about/guidelines.xhtml

تغيرات منذ جائحة كوفيد-١٩

من خلال الأسئلة التالية، يرجى الإشارة إلى مقدار الضغط النفسي الذي تأثرت به. مقارنة بين الوضع الحالي و الوضع قبل جائحة كوفيد-١٩....

١- كيف تقيم مستوى ضغطك الشخصي؟

- ○ ضغط قليل جداً 1
- ○ ضغط الى حداً ما 2
- ○ بقى كما هو 3
- ○ ضغط أكثر 4
- ○ ضغط كثير جداً 5

٢- كيف تقيم مستوى الضغط العام في منزلك؟

- ○ ضغط قليل جداً 1
- ○ ضغط الى حداً ما 2
- ○ بقى كما هو 3
- ○ ضغط أكثر 4
- ○ ضغط كثير جداً 5

٣- كيف تقيم صحتك النفسية الشخصية؟

- ○ أفضل بكثير 1
- ○ أفضل 2
- ○ بقى كما هو 3
- ○ أسوء 4
- ○ أسوء بكثير 5

For peer review only - http://bmjopen.bmj.com/site/about/guidelines.xhtml

٤- كيف تُقيم مقدار الصحة النفسية لأفراد أسرتك بشكل عام؟

- ○ أفضل بكثير 1
- ○ أفضل 2
- ○ بقى كما هو 3
- ○ أسوء 4
- ○ أسوء بكثير 5

For peer review only

K10

الأسئلة العشر التالية سوف يتم سؤالك عن ما كنت تشعر به خلال الأربع أسابيع الماضية. يرجى قراءة الأسئلة التالية و اختيار الإجابة الأقرب التي تمثل وضعك الحاليK-10.

	إطلاقاً 1	قليلاً 2	أحياناً 3	غالباً 4	دائماً 5
١ـ خلال الأربع أسابيع الماضية، إلى أي مدى كنت تشعر أنك مجهد بدون أي سبب واضح؟	○	○	○	○	○
٢ـ خلال الأربع أسابيع الماضية، إلى أي مدى كنت تشعر بالعصبية؟	○	○	○	○	○
٣ـ خلال الأربع أسابيع الماضية، إلى أي مدى كنت تشعر بالعصبية إلى حد أن لاشيء يمكنه أن يجعلك تهدئ؟	○	○	○	○	○
٤ـ خلال الأربع أسابيع الماضية، إلى أي مدى كنت تشعر باليأس؟	○	○	○	○	○
٥ـ خلال الأربع أسابيع الماضية، إلى أي مدى كنت تشعر بعدم الراحة أو القلق؟	○	○	○	○	○
٦ـ خلال الأربع أسابيع الماضية، إلى أي مدى كنت تشعر بعدم الراحة لدرجة أنه لايمكنك الجلوس هادئًا؟	○	○	○	○	○
٧ـ خلال الأربع أسابيع الماضية، إلى أي مدى كنت تشعر بالإكتئاب؟	○	○	○	○	○
٨ـ خلال الأربع أسابيع الماضية، إلى أي مدى كنت تشعر إنك تبذل مجهوداً كبيراً عندما تفعل أي شيء؟	○	○	○	○	○
٩ـ خلال الأربع أسابيع الماضية، إلى أي مدى كنت تشعر بالحزن لدرجة إن لاشيء يحسن من معنوياتك؟	○	○	○	○	○
١٠ـ خلال الأربع أسابيع الماضية، إلى أي مدى كنت تشعر بأن حياتك ليست لها قيمة أو معنى؟	○	○	○	○	○

For peer review only

For peer review only - http://bmjopen.bmj.com/site/about/guidelines.xhtml

PCL-5

فيما يلي قائمة بالمشاكل التى قد يعاني منها بعض الناس كأستجابة لكوفيد-١٩. الرجاء قراءة كل مشكلة بعناية ثم توضيح مدى إنزعاجك لهذه المشكلة خلال الشهر الماضي. خلال الشهر الماضي ما مدى إنزعاجك مما يلي:

كثيراً جداً	غالباً	متوسط	قليلاً	أطلاقاً	
**4**	**3**	**2**	**1**	**0**	

For peer review only

١ـ ذكريات متكررة، مزعجة و غير مرغوب فيها نتيجة التجربة المؤلمة التي مررت بها؟

٢ـ أحلام متكررة، مزعجة نتيجة التجربة المؤلمة التي مررت بها؟

٣ ـالشعور أو التصرف المفاجىء كما لو أن التجربة المؤلمة تحدث مرة آخرى (كأنك تعيش الحادث من جديد)؟

٤ـ الشعور بالإستياء الشديد عندما يذكرك شيء ما بالتجربة المؤلمة؟

٥ـ تعرضك لردة فعل جسدية قوية عند حدوث شيء يذكرك بالتجربة المؤلمة (مثل خفقان بالقلب، صعوبة في التنفس أو التعرق)؟

٦ـ تجنب الذكريات، الأفكار أو المشاعر المتعلقة بالتجربة المؤلمة؟

٧ـ تتجنب الأشياء الخارجية التي تذكرك بالتجربة المؤلمة (مثل الأشخاص، الأماكن، المحادثات، الأنشطة، الأشياء أو المواقف)؟

٨ـ تعاني من مشاكل تذكرك بأجزاء مهمة متعلقة بالتجربة المؤلمة؟

٩ـ أصبح لديك معتقدات سلبية قوية متعلقة بك أو عن الأشخاص الأخرين أو العالم الخارجي (مثل لديك أفكار مثل :أنا سيء، هناك شيء خاطىء فيّ، لا يمكن الوثوق بأحد أو العالم بأكمله)؟

١٠ـ إلقاء اللوم على نفسك أو على شخص آخر فيما يخص حدوث التجربة المؤلمة أوتبعاتها؟

١١ـ وجود مشاعر سلبية قوية مثل الخوف، الرعب، الغضب، الشعور بالذنب أو العار؟

١٢ـ فقدان الإهتمام بالأنشطة التي كنت تستمتع بها؟

١٣ـ الشعور بالبعد أو العزلة عن الأشخاص الأخرين؟

١٤ـ صعوبة الإحساس بالمشاعر الإيجابية (مثل عدم التمكن من الشعور بالسعادة أو مشاعر حب تجاه المقربين إليك)؟

For peer review only - http://bmjopen.bmj.com/site/about/guidelines.xhtml

١٥-سرعة الغضب أو المرور بنوبات الغضب أو التصرف بعدوانية؟	○	○	○	○	○	○
١٦ -الخوض في الكثير من المخاطر أو الأشياء التي قد تسبب لك الضرر؟	○	○	○	○	○	○
١٧-البقاء في حالة تأهب قصوى، إحتراس أو حذر شديدين؟	○	○	○	○	○	○
١٨-شعور متقلب أو الإستفزاز بسهولة؟	○	○	○	○	○	○
١٩-صعوبة في التركيز؟	○	○	○	○	○	○
٢٠-مشاكل في الرغبة في النوم أو في البقاء نائماً؟	○	○	○	○	○	○

For peer review only

WHO-5

يرجى وضع إشارة عند كل من الأسئلة الخمس التالية، حيث تمثل الإجابة شعورك خلال الإسبوعين الماضيين.

	دائماً 5	أكثر الأحيان 4	أكثر من نصف الوقت 3	أقل من نصف الوقت 2	بعض الوقت 1	بتاتاً 0
١- كنت سعيداً و بمزاج جيد.	○	○	○	○	○	○
٢- كنت أشعُر بالهدوء و الإسترخاء.	○	○	○	○	○	○
٣- كنت أشعر بالحيوية و النشاط.	○	○	○	○	○	○
٤- كنت أستيقظ نشطاً و مرتاحاً.	○	○	○	○	○	○
٥- كانت أيامي مليئة بأشياء محببة لنفسي.	○	○	○	○	○	○

For peer review only - http://bmjopen.bmj.com/site/about/guidelines.xhtml

PGTI

من خلال الأسئلة التالية يرجى تحديد الدرجة المناسبة لمدى التغيير الذي احدثته جائحة كوفيد-١٩ على حياتك.

كثيراً 5	إلى حد قليل 4	بشكلٍ معتدل 3	إلى حد ما 2	قليلاً 1	لا على الإطلاق 0

For peer review only

For peer review only - http://bmjopen.bmj.com/site/about/guidelines.xhtml

١- قمت بتغيير أولوياتي عما هو مهم في الحياة.

٢- أصبح لديّ تقديراً أكبر لقيمة حياتي.

٣- طوّرت اهتمامات جديدة.

٤- لديّ إحساس أكبر بالإعتماد على الذات.

٥- لديّ فهمًا أفضل للمسائل الروحية.

٦- أرى بوضوح أكثر أنه يمكنني الإعتماد على الأشخاص في أوقات المشاكل.

٧- أنشأت مساراً جديداً لحياتي.

٨- أصبح لديّ إحساس كبير بالقرب من الآخرين.

٩- أصبحت أكثر استعداداً للتعبير عن مشاعري.

١٠- أستطيع التعامل مع الصعوبات.

١١- أستطيع القيام بأشياء أفضل في حياتي.

١٢- أصبحت أفضل تقبلاً للطرق التي تحل بها الأمور.

١٣- يمكنني تقدير كل يوم بشكلٍ أفضل.

١٤- تتوفر فرص جديدة لم تكن ستتوفر بطريقة أخرى.

١٥- أشعر بمزيد من التعاطف تجاه الآخرين.

١٦- أضع مزيداً من الجهد في علاقاتي.

For peer review only

١٧- احاول جاهداً تغيير الأشياء التي تحتاج إلى التغيير.	○	○	○	○	○	○
١٨- إزدادت قوة إيماني.	○	○	○	○	○	○
١٩- اكتشفت أنني أقوى مما كنت أتصور.	○	○	○	○	○	○
٢٠- تعلمت قدراً كبيراً عن مدى حسن خُلق الأشخاص.	○	○	○	○	○	○
٢١- أتقبل بشكلٍ أفضل احتياجي للآخرين .	○	○	○	○	○	○

Helpline Info

إذا شعرت بالضيق بسبب اي محتوى من خلال هذا الإستبيان ,يمكنك إيجاد الدعم من خلال خطوط المساعدة و الخدمات التالية:

خط المساعدة المحلي: 122

For peer review only

For peer review only - http://bmjopen.bmj.com/site/about/guidelines.xhtml

International Study - Indonesia

Information Sheet

Studi Internasional Dampak Psikososial Pandemi Covid-19
Lembar Informasi untuk Partisipan Survei

Terima kasih sudah berminat terhadap penelitian ini. Mohon Bapak/Ibu/Saudara dapat membaca lembar informasi untuk partisipan survei ini dengan baik sebelum memutuskan untuk bersedia atau tidak menjadi partisipan dalam survei ini. Jika Bapak/Ibu/Saudara bersedia untuk berpartisipasi kami mengucapkan terima kasih. Survei ini tidak akan menimbulkan kerugian bagi Bapak/Ibu/Saudara dan kami berterima kasih sudah mempertimbangkan permohonan kami.

Tujuan dari Penelitian?
Untuk menilai dampak psikososial pandemi Covid-19.

Siapa yang dapat berpartisipasi?
Siapapun dengan usia 18 tahun dan ke atas yang layak untuk berpartisipasi dalam penelitian ini.

Apa yang akan diminta dari Partisipan?
Jika Bapak/Ibu/Saudara setuju untuk mengambil bagian dalam penelitian ini, Bapak/Ibu/Saudara akan diminta untuk mengisi survei online singkat selama 15-20 menit.

Data atau informasi apa yang akan dikumpulkan dan untuk kegunaan apa?
Kami akan mengumpulkan data demografi dan jawaban terhadap serangkaian kuesioner. Data ini hanya akan bisa diakses oleh tim peneliti (Departemen Psikologi Klinis, University of Otago, Christchurch, Selandia Baru dan peneliti dari Universitas Gadjah Mada, Indonesia sebagai mitra peneliti).

Data yang dikumpulkan akan disimpan dengan aman dalam tempat penyimpanan yang aman sedemikian rupa sehingga hanya mereka yang disebutkan di bawah ini yang dapat mengaksesnya dan paling tidak akan disimpan selama 5 tahun.

Tidak ada materi yang secara pribadi dapat mengidentifikasi Bapak/Ibu/Saudara dalam penelitian ini. Hasil penelitian ini dapat dipublikasikan dan akan tersedia di Perpustakaan di University of Otago (Selandia Baru).

Apakah Partisipan dapat mengubah pikiran dan mengundurkan dari keterlibatannya dalam survei ini?
Bapak/Ibu/Saudara dapat mengundurkan dari keterlibatannya dalam survei ini, sebelum selesai dan tanpa merugikan Bapak/Ibu/Saudara.

Kemana Bapak/Ibu/Saudara bisa mendapatkan bantuan?
Beberapa pertanyaan mungkin menyangkut hal-hal yang dapat membuat perasaan tidak nyaman bagi Bapak/Ibu/Saudara. Harap diingat bahwa respon Anda akan bersifat anonim. Anda boleh tidak menjawab pertanyaan yang Anda tidak ingin jawab. Apabila Bapak/Ibu/Saudara merasa depresi atau terganggu secara psikis, dan merasa memerlukan bantuan medis/psikologis, silahkan menghubungi fasilitas layanan kesehatan setempat atau melalui platform layanan konseling psikologi online. Berikut adalah beberapa contoh layanan psikologi online (layanan mungkin berbiaya, kami tidak terafiliasi dengan layanan-layanan tersebut):
https://www.ibunda.id/

For peer review only - http://bmjopen.bmj.com/site/about/guidelines.xhtml

https://riliv.co/konseling
https://get-kalm.com/id/
https://pijarpsikologi.org/blog/konsulgratis

Bagaimana jika Partisipan memiliki pertanyaan?

Jika Bapak/Ibu/Saudara memiliki pertanyaan mengenai penelitian ini maka silakan menghubungi:

Dr Caroline Bell
Department of Psychological Medicine
Email Address: caroline.bell@otago.ac.nz
Dr Sandila Tanveer
Department of Psychological Medicine
Email Address: sandila.tanveer@otago.ac.nz
Anggi Rahajeng, M.Ec
Department of Economics and Business, Vocational School, Universitas Gadjah Mada
Email Address: anggi.rahajeng@ugm.ac.id
Irfan Yunianto, M.Sc
Department of Obstetrics and Gynaecology
Email Address: irfan@pbio.uad.ac.id atau yunir564@student.otago.ac.nz

For peer review only

Consent Form

Jika Bapak/Ibu/Saudara setuju untuk berpartisipasi dalam survei penelitian ini maka silakan memberikan persetujuan dengan cara mencentang semua keterangan di bawah ini.

Saya telah membaca atau telah mendapatkan penjelasan mengenai penelitian ini dalam Bahasa pertama saya (Bahasa Indonesia) dan memahami lembar informasi bagi Partisipan.

Saya mengetahui untuk menghubungi siapa jika saya memiliki pertanyaan mengenai penelitian ini secara umum.

Saya memahami bahwa partisipasi dalam survei ini bersifat sukarela (keputusan saya), dan saya dapat mengundurkan diri kapan pun tanpa sanksi.

Jika saya memutuskan untuk mengundurkan diri dari survei ini, saya setuju bahwa informasi yang dikumpulkan mengenai diri saya sampai saat saya menarik dapat terus diproses.

Saya memahami bahwa data akan disimpan dalam tempat penyimpanan yang aman paling tidak selama 5 tahun.

Saya memahami bahwa hasil dari penelitian ini akan dipublikasikan dan akan tersedia di perpustakaan University of Otago (Dunedin, Selandia Baru) dan dapat diakses oleh tim peneliti Otago dan peneliti Universitas Gadjah Mada sebagai mitra peneliti.

Saya memahami bahwa setiap informasi atau opini yang saya berikan akan dijaga kerahasiaannya oleh tim peneliti dan bahwa setiap hasil penelitian yang dipublikasikan atau dilaporkan tidak akan mengidentifikasi partisipan atau institusinya.

- ○ **Saya setuju untuk berpartisipasi dalam penelitian.**
- ○ **Saya tidak setuju untuk berpartisipasi dalam penelitian.**

For peer review only

Sociodemographic Information

1. Jenis Kelamin

◯ Laki-laki

◯ Perempuan

◯ Lainnya

◯ Memilih untuk tidak menjawab

2. Umur (tahun)

____

3. Status pernikahan

◯ Belum menikah

◯ Sedang memiliki hubungan

◯ Menikah

◯ Berpisah

◯ Cerai hidup

◯ Cerai mati

4. Apakah agama yang Anda anut sekarang?

◯ Tidak beragama

◯ Islam

◯ Kristen/Katolik

◯ Hindu

◯ Buddha

◯ Lain-lain (Sebutkan) ____

For peer review only

5. Apa latar belakang etnisitas/suku bangsa yang Anda miliki? (Sebutkan dan Anda boleh menyebutkan lebih dari satu etnis/suku bangsa)

____

6. Pendidikan tertinggi yang ditamatkan?

- ○ Pendidikan non formal (homeschooling, les privat/les Bersama, pelatihan)
- ○ SMA/SMK dan sederajat
- ○ Perguruan Tinggi D3
- ○ Perguruan Tinggi S1/D4
- ○ Perguruan Tinggi S2/S3

7. Status pekerjaan dan pendidikan yang saat ini dilakukan (Beri tanda pada pilihan Anda)

- ☐ Belajar penuh waktu
- ☐ Belajar paruh waktu
- ☐ Tidak bekerja
- ☐ Bekerja penuh waktu (karyawan/pegawai)
- ☐ Bekerja paruh waktu (karyawan/pegawai)
- ☐ Bekerja berusaha sendiri/wiraswasta penuh waktu (Full-time self employed)
- ☐ Bekerja berusaha sendiri/wiraswasta paruh waktu (Part-time self employed)
- ☐ Cuti diluar tanggungan negara (jika Anda berstatus ASN ataupun tugas belajar)
- ☐ Bekerja di rumah, tanpa menerima gaji (misal: ibu rumah tangga)
- ☐ Pensiun/Purna Tugas
- ☐ Lainnya (Sebutkan) ____

8. Pekerjaan (jika saat ini sedang melakukan kerja penuh waktu dan atau kerja paruh waktu)

____

For peer review only

For peer review only - http://bmjopen.bmj.com/site/about/guidelines.xhtml

9. Berapakah pendapatan dan penghasilan Anda (termasuk suami/istri, jika menikah) dalam 1 bulan (per bulan-Rp)

____

9a. Dibandingkan dengan pendapatan rata-rata nasional penduduk Indonesia tahun 2020 berdasarkan data BPS Februari, 2020 (Rp 1,6 juta per bulan), maka bagaimana Anda menilai posisi pendapatan keluarga Anda per bulan?

○ Dibawah rata-rata

○ Rata-rata

○ Diatas rata-rata

10. Apakah saat ini berada pada lingkungan yang melakukan Pemberlakukan Pembatasan Kegiatan Masyarakat (PPKM)?

○ Iya

○ Tidak

11. Apakah Anda sudah mendapatkan vaksin Covid-19?

○ Iya

○ Tidak

11a. Jika belum mendapatkan vaksin, apakah Anda bersedia untuk divaksinasi di kemudian hari?

○ Iya

○ Tidak

For peer review only

For peer review only - http://bmjopen.bmj.com/site/about/guidelines.xhtml

12. Kami menyadari bahwa sebagian orang memiliki pengalaman traumatik di masa lalu yang membuat seseorang akan lebih mudah atau sulit untuk menjalani kehidupan, menghadapi tantangan baru maupun mengatasi permasalahan baru yang muncul di kemudian hari.

Sebelum wabah Covid-19, apakah Anda pernah mengalami ataupun menyaksikan beberapa kejadian seperti di bawah ini: (Beri tanda sebanyak mungkin sesuai yang Anda alami)

☐ Bencana alam (banjir, gempa bumi, tsunami, tanah longsor dsb)

☐ Hidup di zona konflik atau peperangan ataupun daerah darurat militer

☐ Pengalaman tidak menyenangkan ketika masa kecil sebelum usia 15 tahun (diabaikan, ditelantarkan orang tua, dibuli, atau kekerasan fisik-mental/seksual)

☐ Kekerasan fisik-mental/seksual Ketika usia diatas 16 tahun

☐ Kecelakaan fisik yang serius

☐ Lainnya, sebutkan pengalaman lainnya yang menyebabkan traumatik di masa lalu
____

☐ Tidak pernah mengalami pengalaman traumatik di masa lalu

For peer review only

Covid Psychosocial Impacts Scale

Dampak terhadap Pribadi

Berikan respon Ya / Tidak terhadap pertanyaaan di bawah ini, jika hal berikut dialami oleh Anda terkait dengan wabah Covid-19. Jika jawaban anda Ya, mohon berikan indikasi penilaian tekanan (stress) yang dialami seusai dengan skala berikut.

1. Apakah Anda mempunyai masalah kesehatan yang membuat Anda rentan/beresiko terhadap Covid-19?

- ◯ **Tidak 0**
- ◯ **Ya** Tidak membuat stress 1
- ◯ **Ya** Sedikit membuat stress 2
- ◯ **Ya** Cukup membuat stress 3
- ◯ **Ya** Banyak membuat stress 4
- ◯ **Ya** Sangat membuat stress 5

2. Apakah Anda merasa berisiko terpapar dengan orang yang terkena Covid-19?

- ◯ **Tidak0**
- ◯ **Ya** Tidak membuat stress 1
- ◯ **Ya** Sedikit membuat stress 2
- ◯ **Ya** Cukup membuat stress 3
- ◯ **Ya** Banyak membuat stress 4
- ◯ **Ya** Sangat membuat stress 5

For peer review only

3. Apakah Anda merasa pernah terkena Covid-19?

- **Tidak0**
- **Ya** Tidak membuat stress 1
- **Ya** Sedikit membuat stress 2
- **Ya** Cukup membuat stress 3
- **Ya** Banyak membuat stress 4
- **Ya** Sangat membuat stress 5

3a. Apakah Anda pernah mendapatkan hasil positif dari tes Covid-19?

- **Tidak0**
- **Ya** Tidak membuat stress 1
- **Ya** Sedikit membuat stress 2
- **Ya** Cukup membuat stress 3
- **Ya** Banyak membuat stress 4
- **Ya** Sangat membuat stress 5

3b. Apakah saat ini Anda merasa mengalami gejala Covid-19?

- **Tidak0**
- **Ya** Tidak membuat stress 1
- **Ya** Sedikit membuat stress 2
- **Ya** Cukup membuat stress 3
- **Ya** Banyak membuat stress 4
- **Ya** Sangat membuat stress 5

For peer review only - http://bmjopen.bmj.com/site/about/guidelines.xhtml

Dampak terhadap Keluarga

Berikan respon Ya/Tidak terhadap pertanyaan di bawah ini, jika hal berikut dialami oleh anggota keluarga dekat Anda terkait dengan wabah Covid-19. Jika jawaban anda Ya, mohon berikan indikasi penilaian tekanan (stress) yang dialami sesuai dengan skala berikut.

1. Apakah **keluarga dekat Anda mempunyai masalah** kesehatan **yang membuat mereka rentan/berisiko terhadap Covid-19?**

- ◯ **Tidak0**
- ◯ **Ya** Tidak membuat stress 1
- ◯ **Ya** Sedikit membuat stress 2
- ◯ **Ya** Cukup membuat stress 3
- ◯ **Ya** Banyak membuat stress 4
- ◯ **Ya** Sangat membuat stress 5

2. Apakah Anda merasa anggota keluarga Anda berisiko berhadapan dengan orang yang terkena Covid-19?

- ◯ **Tidak** 0
- ◯ **Ya** Tidak membuat stress 1
- ◯ **Ya** Sedikit membuat stress 2
- ◯ **Ya** Cukup membuat stress 3
- ◯ **Ya** Banyak membuat stress 4
- ◯ **Ya** Sangat membuat stress 5

For peer review only

3. Apakah Anda merasa ada anggota keluarga terdekat yang terkena Covid-19?

- **Tidak** 0
- **Ya** Tidak membuat stress 1
- **Ya** Sedikit membuat stress 2
- **Ya** Cukup membuat stress 3
- **Ya** Banyak membuat stress 4
- **Ya** Sangat membuat stress 5

3a. Apakah ada anggota keluarga terdekat anda yang dites hasilnya positif Covid-19?

- **Tidak** 0
- **Ya** Tidak membuat stress 1
- **Ya** Sedikit membuat stress 2
- **Ya** Cukup membuat stress 3
- **Ya** Banyak membuat stress 4
- **Ya** Sangat membuat stress 5

3b. Apakah ada anggota keluarga terdekat anda mengalami gejala Covid-19?

- **Tidak** 0
- **Ya** Tidak membuat stress 1
- **Ya** Sedikit membuat stress 2
- **Ya** Cukup membuat stress 3
- **Ya** Banyak membuat stress 4
- **Ya** Sangat membuat stress 5

For peer review only

3c. Apakah ada anggota keluarga terdekat anda meninggal dunia karena Covid-19?

- **Tidak** 0
- **Ya** Tidak membuat stress 1
- **Ya** Sedikit membuat stress 2
- **Ya** Cukup membuat stress 3
- **Ya** Banyak membuat stress 4
- **Ya** Sangat membuat stress 5

Apakah Anda memiliki anggota keluarga terdekat yang menetap di luar negeri yang Anda khawatirkan berisiko terkena Covid-19?

- **Tidak** 0
- **Ya** Tidak membuat stress 1
- **Ya** Sedikit membuat stress 2
- **Ya** Cukup membuat stress 3
- **Ya** Banyak membuat stress 4
- **Ya** Sangat membuat stress 5

5. Apakah Anda memiliki keluarga dekat di luar negeri yang tidak dapat Anda kunjungi atau mereka tidak dapat mengunjungi Anda saat ini?

- **Tidak** 0
- **Ya** Tidak membuat stress 1
- **Ya** Sedikit membuat stress 2
- **Ya** Cukup membuat stress 3
- **Ya** Banyak membuat stress 4
- **Ya** Sangat membuat stress 5

For peer review only - http://bmjopen.bmj.com/site/about/guidelines.xhtml

Pekerjaan

Berikan respon Ya / Tidak / Tidak berlaku terhadap pertanyaaan di bawah ini, jika hal berikut dialami oleh Anda atau anggota keluarga Anda terkait dengan wabah Covid-19. Jika jawaban anda Ya, mohon berikan indikasi penilaian tekanan (stress) yang dialami sesuai dengan skala berikut.

1. Apakah Anda atau anggota keluarga Anda termasuk pekerja di sektor penting (misalnya bekerja di bidang kesehatan, penegak hukum, keuangan, petugas yang berkaitan dengan kedaruratan atau penyedia jasa layanan penting lainnya)?

- ◯ **Tidak** 0
- ◯ **Ya** Tidak membuat stress 1
- ◯ **Ya** Sedikit membuat stress 2
- ◯ **Ya** Cukup membuat stress 3
- ◯ **Ya** Banyak membuat stress 4
- ◯ **Ya** Sangat membuat stress 5

2. Apakah Anda atau anggota keluarga Anda mengalami perubahan (penambahan/pengurangan) jam kerja?

- ◯ **Tidak** 0
- ◯ **Ya** Tidak membuat stress 1
- ◯ **Ya** Sedikit membuat stress 2
- ◯ **Ya** Cukup membuat stress 3
- ◯ **Ya** Banyak membuat stress 4
- ◯ **Ya** Sangat membuat stress 5

For peer review only

For peer review only - http://bmjopen.bmj.com/site/about/guidelines.xhtml

3. Apakah Anda atau anggota keluarga Anda harus berubah tempat kerjanya menjadi bekerja dari rumah?

- **Tidak** 0
- **Ya** Tidak membuat stress 1
- **Ya** Sedikit membuat stress 2
- **Ya** Cukup membuat stress 3
- **Ya** Banyak membuat stress 4
- **Ya** Sangat membuat stress 5

4. Apakah Anda atau anggota keluarga Anda terpaksa berubah jenis pekerjaannya?

- **Tidak** 0
- **Ya** Tidak membuat stress 1
- **Ya** Sedikit membuat stress 2
- **Ya** Cukup membuat stress 3
- **Ya** Banyak membuat stress 4
- **Ya** Sangat membuat stress 5

For peer review only

5. Apakah Anda atau anggota keluarga Anda kehilangan pekerjaan?

- ◯ **Tidak Berlaku**
- ◯ **Tidak** 0
- ◯ **Ya** Tidak membuat stress 1
- ◯ **Ya** Sedikit membuat stress 2
- ◯ **Ya** Cukup membuat stress 3
- ◯ **Ya** Banyak membuat stress 4
- ◯ **Ya** Sangat membuat stress 5

6. Apakah usaha Anda atau usaha anggota keluarga Anda mengalami kesulitan bertahan saat ini?

- ◯ **Tidak Berlaku**
- ◯ **Tidak** 0
- ◯ **Ya** Tidak membuat stress 1
- ◯ **Ya** Sedikit membuat stress 2
- ◯ **Ya** Cukup membuat stress 3
- ◯ **Ya** Banyak membuat stress 4
- ◯ **Ya** Sangat membuat stress 5

For peer review only

Pendapatan

Berikan respon Ya / Tidak terhadap pertanyaaan di bawah ini, jika hal berikut dialami oleh Anda, keluarga Anda atau anggota keluarga Anda terkait dengan wabah Covid-19. Jika jawaban anda Ya, mohon berikan indikasi penilaian tekanan (stress) yang dialami sesuai dengan skala berikut.

1. Apakah keluarga Anda mengalami penurunan pendapatan?

- ◯ **Tidak** 0
- ◯ **Ya** Tidak membuat stress 1
- ◯ **Ya** Sedikit membuat stress 2
- ◯ **Ya** Cukup membuat stress 3
- ◯ **Ya** Banyak membuat stress 4
- ◯ **Ya** Sangat membuat stress 5

2. Apakah keluarga Anda mengalami penurunan pendapatan yang mengakibatkan Anda kesulitan membayar tagihan atau membeli makanan/bahan pokok makanan?

- ◯ **Tidak** 0
- ◯ **Ya** Tidak membuat stress 1
- ◯ **Ya** Sedikit membuat stress 2
- ◯ **Ya** Cukup membuat stress 3
- ◯ **Ya** Banyak membuat stress 4
- ◯ **Ya** Sangat membuat stress 5

For peer review only

For peer review only - http://bmjopen.bmj.com/site/about/guidelines.xhtml

Hubungan Keluarga

Berikan respon Ya/Tidak terhadap pertanyaan di bawah ini, jika hal berikut dialami oleh Anda terkait dengan wabah Covid-19. Jika jawaban anda Ya, mohon berikan indikasi penilaian tekanan (stress) yang dialami sesuai dengan skala berikut. Akibat dari Covid-19……….

1. Meningkatkan pertengkaran/memperburuk komunikasi/hubungan menjadi renggang-jauh dalam keluarga Anda?

- ◯ **Tidak** 0
- ◯ **Ya** Tidak membuat stress 1
- ◯ **Ya** Sedikit membuat stress 2
- ◯ **Ya** Cukup membuat stress 3
- ◯ **Ya** Banyak membuat stress 4
- ◯ **Ya** Sangat membuat stress 5

2. Hubungan Anda dengan teman/kerabat (baik secara fisik maupun daring/online) menurun?

- ◯ **Tidak** 0
- ◯ **Ya** Tidak membuat stress 1
- ◯ **Ya** Sedikit membuat stress 2
- ◯ **Ya** Cukup membuat stress 3
- ◯ **Ya** Banyak membuat stress 4
- ◯ **Ya** Sangat membuat stress 5

For peer review only

For peer review only - http://bmjopen.bmj.com/site/about/guidelines.xhtml

Perubahan dalam kegiatan dan perilaku sehari-hari

Berikan respon Ya / Tidak / Tidak Berlaku terhadap pertanyaan di bawah ini, jika hal berikut dialami oleh Anda terkait dengan wabah Covid-19. Jika jawaban anda Ya, mohon berikan indikasi penilaian tekanan (stress) yang dialami sesuai dengan skala berikut.

1. Apakah wabah Covid-19 telah merubah kegiatan sehari-hari Anda (Sebagai contoh, tidur, olah raga, waktu bebas, aktifitas yang menyenangkan atau hobi)?

- **Tidak** 0
- **Ya** Tidak membuat stress 1
- **Ya** Sedikit membuat stress 2
- **Ya** Cukup membuat stress 3
- **Ya** Banyak membuat stress 4
- **Ya** Sangat membuat stress 5

2. Membuat Anda menunda/menghindar untuk mencari bantuan kesehatan saat Anda atau keluarga Anda membutuhkannya?

- **Tidak** 0
- **Ya** Tidak membuat stress 1
- **Ya** Sedikit membuat stress 2
- **Ya** Cukup membuat stress 3
- **Ya** Banyak membuat stress 4
- **Ya** Sangat membuat stress 5

For peer review only

3. Menurunkan keterlibatan Anda dalam aktifitas keagamaan (baik tatap muka ataupun online)?

- **Tidak Berlaku**
- **Tidak** 0
- **Ya** Tidak membuat stress 1
- **Ya** Sedikit membuat stress 2
- **Ya** Cukup membuat stress 3
- **Ya** Banyak membuat stress 4
- **Ya** Sangat membuat stress 5

4. Apakah ada perubahan besar terhadap keseharian Anda?

- **Tidak** 0
- **Jika ada, mohon jelaskan** 1 ____

Jika jawaban anda Ya, mohon berikan indikasi penilaian tekanan (stress) yang dialami sesuai dengan skala berikut.

- Tidak membuat stress 1
- Sedikit membuat stress 2
- Cukup membuat stress 3
- Banyak membuat stress 4
- Sangat membuat stress 5

For peer review only

Paparan terhadap informasi mengenai Covid-19

1. Terkait dengan Covid-19, dari manakah Anda mendapatkan informasinya?
(Mohon dipilih sebanyak mungkin sesuai yang Anda alami)

- ☐ Berita
- ☐ Facebook/Twitter
- ☐ Situs kesehatan
- ☐ Dari mulut ke mulut
- ☐ Lain-lain (Mohon dijelaskan) ____

2. Seberapa banyak tekanan (stress) yang Anda alami karena pemberitaan mengenai Covid-19?

- ◯ Tidak membuat stress1
- ◯ Sedikit membuat stress2
- ◯ Cukup membuat stress3
- ◯ Banyak membuat stress4
- ◯ Sangat membuat stress5

Perubahan sejak Covid-19

Dari pertanyaan di bawah ini, pilihlah yang Anda rasakan dengan melingkari nomor yang sesuai dengan situasi Anda. Mohon bandingkan saat ini dengan sebelum wabah Covid-19...

1. Bagaimana Anda menilai tingkat tekanan/stres yang dialami?

- ◯ Sangat tidak merasakan tekanan/stress1
- ◯ Sedikit tidak merasakan tekanan/stress2
- ◯ Tidak ada perubahan3
- ◯ Lebih merasakan tekanan/stress4
- ◯ Sangat merasakan tekanan/stress5

For peer review only

2. Bagaimana Anda menilai tingkat tekanan (stress) yang dirasakan oleh anggota keluarga Anda secara keseluruhan?

- ◯ Sangat tidak merasakan tekanan/stress1
- ◯ Sedikit tidak merasakan tekanan/stress2
- ◯ Tidak ada perubahan3
- ◯ Lebih merasakan tekanan/stress4
- ◯ Sangat merasakan tekanan/stress5

3. Bagaimana Anda menilai kesejahteraan psikologis Anda?

- ◯ Sangat baik1
- ◯ Lebih baik2
- ◯ Tidak ada perubahan3
- ◯ Lebih buruk4
- ◯ Sangat buruk5

4. Bagaimana Anda menilai kesejahteraan psikologis anggota keluarga Anda secara keseluruhan?

- ◯ Sangat baik1
- ◯ Lebih baik2
- ◯ Tidak ada perubahan3
- ◯ Lebih buruk4
- ◯ Sangat buruk5

For peer review only - http://bmjopen.bmj.com/site/about/guidelines.xhtml
For peer review only

K-10

Sepuluh pertanyaan berikut ini menanyakan perasaan Anda dalam 4 minggu terakhir. Untuk setiap pertanyaan, mohon lingkari pilihan yang menjelaskan seringnya Anda merasakan hal tersebut.

	Tidak pernah 1	Jarang 2	Kadang-kadang 3	Hampir selalu 4	Setiap saat 5
1. Dalam kurun waktu empat minggu terakhir, seberapa sering Anda merasa lelah tanpa sebab yang jelas?	○	○	○	○	○
2. Dalam empat minggu terakhir, seberapa sering Anda merasa cemas?	○	○	○	○	○
3. Dalam empat minggu terakhir seberapa sering Anda merasa cemas dan tidak bisa menenangkan diri?	○	○	○	○	○
4. Dalam empat minggu terakhir, seberapa sering Anda merasa tidak punya harapan?	○	○	○	○	○
5. Dalam empat minggu terakhir, seberapa sering Anda merasa gelisah atau resah?	○	○	○	○	○
6. Dalam empat minggu terakhir, seberapa sering Anda merasa gelisah yang menyebabkan Anda tidak dapat duduk diam?	○	○	○	○	○
7. Dalam empat minggu terakhir, seberapa sering Anda merasa tertekan?	○	○	○	○	○
8. Dalam empat minggu terakhir, seberapa sering Anda merasa berat sekali untuk melakukan sesuatu?	○	○	○	○	○
9. Dalam empat minggu terakhir, seberapa sering Anda merasa sedih sehingga	○	○	○	○	○

For peer review only

For peer review only - http://bmjopen.bmj.com/site/about/guidelines.xhtml

tidak ada apapun yang dapat menghibur Anda?					
10. Dalam empat minggu terakhir, seberapa sering Anda merasa tidak berharga sama sekali?	○	○	○	○	○

For peer review only

For peer review only - http://bmjopen.bmj.com/site/about/guidelines.xhtml

PCL-5

Di bawah ini adalah masalah-masalah yang seringkali dialami seseorang dalam menghadapi situasi Covid-19. Mohon dibaca setiap masalah dengan teliti dan kemudian lingkari nomor untuk mengindikasikan seberapa sering Anda terganggu oleh masalah tersebut dalam kurun waktu satu bulan terakhir. Dalam satu Bulan terakhir, seberapa sering Anda terganggu dengan hal berikut:

	**Tidak sama sekali** 0	**Sedikit tergganggu** 1	**Kadang-kadang tergganggu** 2	**Hampir tergganggu** 3	**Sangat tergganggu** 4
1. Terulangnya kenangan yang tidak diinginkan dan membuat tertekan/stres?	○	○	○	○	○
2. Terulangnya mimpi yang menganggu saat mengalami tekanan/stres?	○	○	○	○	○
3. Tiba-tiba merasakan atau bertingkah seakan-akan pengalaman saat tekanan/stres terjadi terulang kembali (seakan-akan Anda kembali ke situasi tersebut?	○	○	○	○	○
4. Merasa marah saat terjadi sesuatu yang mengingatkan Anda pada pengalaman saat tekanan/stres terjadi?	○	○	○	○	○
5. Mengalami reaksi fisik yang kuat saat sesuatu terjadi dimana mengingatkan Anda pada pengalaman saat tekanan/stres terjadi (sebagai contoh, jantung berdebar kencang, mengalami kesusahan bernafas, berkeringat)?	○	○	○	○	○
6. Menghindari kenangan, pikiran ataupun perasaan yang terkait dengan pengalaman saat tekanan/stres terjadi?	○	○	○	○	○

For peer review only

7. Menghindari hal-hal dari pihak luar yang mengingatkan Anda kepada pengalaman saat tekanan/stres terjadi (sebagai contoh, orang, tempat, percakapan, aktifitas, benda atau situasi)?

8. Kesulitan dalam mengingat hal penting dari pengalaman saat tekanan/stres terjadi?

9. Mengalami prasangka negatif yang kuat terhadap diri sendiri, orang lain, atau dunia (sebagai contoh, memiliki pikiran seperti: saya jahat, ada hal yang salah di dalam diri saya, tidak ada yang bisa dipercaya, dunia ini sangat kejam)?

10. Menyalahkan diri sendiri atau orang lain terhadap pengalaman saat tekanan/stres terjadi ataupun terhadap hal yang terjadi setelahnya?

11. Memilik perasaan negatif yang kuat seperti ketakutan, perasaan ngeri, marah, merasa bersalah ataupun malu?

12. Tidak tertarik lagi melakukan kegiatan yang biasanya Anda sukai?

13. Merasa jauh dan tidak memiliki hubungan dengan orang lain?

14. Kesulitan untuk memiliki perasaan positif (sebagai contoh, tidak dapat merasakan kebahagian atau

For peer review only

mencintai orang yang dekat dengan Anda)?					
15. Bertingkah laku yang mengganggu, marah tanpa kendali atau bersikap agresif?	○	○	○	○	○
16. Melakukan hal yang berisiko tinggi ataupun melakukan hal yang dapat membahayakan diri Anda?	○	○	○	○	○
17. Bersikap “sangat waspada” atau berhati-hati atau pun berjaga-jaga?	○	○	○	○	○
18. Merasa gelisah dan mudah terkejut?	○	○	○	○	○
19. Kesulitan dalam berkonsentrasi?	○	○	○	○	○
20. Susah tertidur atau tidur lebih lama?	○	○	○	○	○

For peer review only

For peer review only - http://bmjopen.bmj.com/site/about/guidelines.xhtml

WHO-5

Mohon berikan indikasi untuk ke lima pernyataan di bawah ini yang sesuai dengan yang Anda rasakan selama kurun waktu 2 minggu terakhir. Dalam kurun waktu 2 minggu terakhir

	Setiap waktu 5	Kebanyakan waktu 4	Sebagian waktu 3	Hampir sebagian waktu 2	Sesekali 1	Tidak sama sekali 0
1. Saya merasa gembira dan bahagia	○	○	○	○	○	○
2. Saya merasa tenang dan rileks	○	○	○	○	○	○
3. Saya merasa aktif dan semangat	○	○	○	○	○	○
4. Saya terbangun dengan perasaan segar dan cukup istirahat	○	○	○	○	○	○
5. Kegiatan sehari-hari saya penuh dengan hal-hal yang saya sukai	○	○	○	○	○	○

For peer review only

PTGI

Dari pernyataan di bawah ini, mohon indikasikan penilaian terjadinya perubahan dalam hidup Anda sebagai akibat dari Covid-19 dengan menggunakan skala di bawah ini.

	Tidak sama sekali 0	Sedikit 1	Agak 2	Kadang-kadang 3	Hampir 4	Sangat 5
1. Saya merubah prioritas mengenai apa yang penting dalam hidup.	◯	◯	◯	◯	◯	◯
2. Saya memiliki apresiasi besar terhadap nilai hidup.	◯	◯	◯	◯	◯	◯
3. Saya mengembangkan minat baru.	◯	◯	◯	◯	◯	◯
4. Saya memiliki perasaan besar terhadap kepercayaan diri.	◯	◯	◯	◯	◯	◯
5. Saya memiliki pemahaman yang baik terhadap masalah spiritual.	◯	◯	◯	◯	◯	◯
6. Saya melihat bahwa saya bisa meminta bantuan kepada orang lain di saat kesulitan.	◯	◯	◯	◯	◯	◯
7. Saya menempuh jalan baru bagi hidup saya.	◯	◯	◯	◯	◯	◯
8. Saya memiliki rasa kedekatan dengan orang lain.	◯	◯	◯	◯	◯	◯
9. Saya lebih bisa mengekspresikan emosi.	◯	◯	◯	◯	◯	◯
10. Saya tahu jika saya bisa mengatasi kesulitan.	◯	◯	◯	◯	◯	◯
11. Saya bisa melakukan hal-hal baik dalam hidup saya.	◯	◯	◯	◯	◯	◯

For peer review only

For peer review only - http://bmjopen.bmj.com/site/about/guidelines.xhtml

12. Saya semakin bisa menerima hal-hal yang terjadi.	○	○	○	○	○	○
13. Saya bisa lebih menghargai setiap hari yang saya lalui.	○	○	○	○	○	○
14. Kesempatan baru tersedia dibandingkan sebelumnya.	○	○	○	○	○	○
15. Saya lebih memiliki rasa kasih sayang kepada sesama.	○	○	○	○	○	○
16. Saya lebih memberikan upaya dalam hubungan saya dengan orang lain	○	○	○	○	○	○
17. Saya lebih bisa mengusahakan untuk merubah hal yang memang bisa diubah.	○	○	○	○	○	○
18. Saya memiliki keyakinan yang lebih kuat terhadap agama.	○	○	○	○	○	○
19. Saya menyadari bahwa saya lebih kuat daripada yang saya kira.	○	○	○	○	○	○
20. Saya menyadari bahwa ternyata orang lain itu baik.	○	○	○	○	○	○
21. Saya lebih bisa menerima bahwa saya membutuhkan orang lain.	○	○	○	○	○	○

For peer review only

For peer review only - http://bmjopen.bmj.com/site/about/guidelines.xhtml

Helpline Information

Mohon diingat, jika Anda merasa terganggu/tertekan secara psikis oleh pertanyaan-pertanyaan dalam survei ini, anda dapat menghubungi fasilitas layanan kesehatan setempat atau melalui platform layanan konseling psikologi online. Berikut adalah beberapa contoh layanan psikologi online (layanan mungkin berbiaya, kami tidak terafiliasi dengan layanan-layanan tersebut):

https://www.ibunda.id/
https://riliv.co/konseling
https://get-kalm.com/id/
https://pijarpsikologi.org/blog/konsulgratis

For peer review only

For peer review only - http://bmjopen.bmj.com/site/about/guidelines.xhtml

International Study - Malaysia

Information Sheet

Kajian Antarabangsa Untuk Menilai Kesan Wabak Covid-19 Kepada Psikososial Individu

Terima kasih atas sokongan anda kepada kajian ini. Sila baca maklumat di lampiran ini dengan teliti sebelum membuat keputusan untuk mengambil bahagian dalam kajian ini.

Kami mengucapkan ribuan terima kasih jika anda memutuskan untuk mengambil bahagian dalam kajian ini. Jika ada tidak berminat untuk meneruskan dalam kajian ini, anda tidak akan terkesan dan kami juga beterima kasih kerana sudi mempertimbangkan permintaan kami.

Apakah matlamat kajian ini?
Matlamat kajian ini adalah untuk menilai kesan wabak Covid-19 kepada psikososial individu.

Siapa yang boleh mengambil bahagian?
Individu berumur 18 tahun keatas layak until mengambil bahagian dalan kajian ini.

Apakah yang akan dilakukan oleh peseta kajian ini?
Jika ada bersetuju untuk mengambil bahagian dalam kajian ini, anda akan diminta untuk melengkapkan satu tinjauan dalam talian yang akan mengambil masa dalam 15-20 minit.

Data atau maklumat apa yang akan diambil dan bagaimana ia akan digunakan?
Kami akan mengambil data demografik dan jawapan kepada beberapa soal selidik. Data dan maklumat ini akan hanya disemak oleh ketua-ketua penyelidik (Dr. Caroline Bell dan Dr. Sandila Tanveer, Jabatan Perubatan Psikososial, University of Otago, Christchurch, New Zealand).

Maklumat yang diambil akan disimpan dengan selamat dan hanya nama individu-individu yang tertera dibawah sahaja yang boleh melihat maklumat tersebut. Data yang diperoleh daripada kajian ini akan disimpan di tempat penyimpanan yang selamat tidak kurang daripada lima tahun.

Kajian ini tidak akan mengambil sebarang maklumat peribadi yang akan mengenal pasti peserta yang mengambil bahagian. Keputusan kajian ini berkemungkinan untuk diterbitkan dan cetakan akan berada di Perpustakaan University of Otago (Dunedin, New Zealand).

Bolehkah peserta berubah fikiran dan menarik diri daripada kajian ini?
Ya, anda boleh menarik diri daripada kajian ini sebelum ia tamat dan jika ia tidak akan mendatangkan bahaya kepada diri anda.

Dimana anda boleh mendapatkan bantuan?
Talian Nur Kasih – 15999 Lifeline Association of Malaysia (03-42657995) Befrienders (03-76272929) MIASA (03-79321409) MMHA (0176133039) Talian Sokongan Psikososial Covid-19 (03-88707519/7527)

For peer review only - http://bmjopen.bmj.com/site/about/guidelines.xhtml

Ada sebarang pertanyaan tentang Covid-19?

Hubungi talian tetap Kementerian Kesihatan untuk sebarang nasihat kesihatan dan maklumat tentang Covid-19.
Nombornya adalah 03-77239300/ 03-88810200/03-88820600/03-88810700.
Whatsapp ke: 013-9279454/0310-9699435.
Layari webite https://covidnow.moh.gov.my/ atau cprc@moh.gov.my

Siapa boleh peserta hubungi jika ada sebarang pertanyaan?

Jika anda ada sebarang pertanyaan mengenai kajian ini, sila hubungi individu di bawah:

Prof Madya Dr Amer Siddiq Amer Nordin
Jabatan Perubatan Psikologi, Universiti Malaya
Emel: amersiddiq@um.edu.my

Dr Caroline Bell
Jabatan Perubatan Psikososial
Emel: caroline.bell@otago.ac.nz

Dr Sandila Tanveer
Jabatan Perubatan Psikososial
Emel: sandila.tanveer@otago.ac.nz

For peer review only

Consent Form

Jika ada bersetuju untuk mengambil bahagian dalam kajian ini, sila berikan persetujuan anda untuk semua kenyataan berikut.

- Saya sudah membaca, atau sudah dibacakan dan diterangkan kepada saya dalam bahasa ibunda saya, dan saya telah memahami kandungan di dalam Senarai Maklumat Untuk Peserta Kajian.
- Saya tahu kepada siapa perlu saya rujuk jikalau saya ada sebarang pertanyaan tentang kajian ini.
- Saya faham bahawa penyertaan kepada kajian ini adalah secara sukarela (pilihan saya sendiri) dan saya faham bahawa saya boleh mengundur diri daripada kajian ini bila-bila masa shaja tanpa sebarang hukuman.
- Saya telah diberikan segala maklumat tentang talian hayat sekiranya saya memerlukan sokongan dan pertolongan sekiranya saya berasa cemas ketika sesi temuduga.
- Saya faham bahawa data dan maklumat yang diperolehi akan disimpan di tempat yang selamat tidak kurang daripada lima tahun.
- Saya faham bahawa keputusan kajian ini mungkin akan diterbitkan dan akan berada di Perpustakaan University of Otago (Dunedin, New Zealand).
- Saya faham bahawa penyertaan saya dalam kajian ini adalah sulit dan persendirian. Tiada sebarang maklumat yang diperolehi oleh kajian ini akan dapat mengenal pasti identiti saya atau digunakan dalam sebarang laporan.

○ **Saya bersetuju untuk menyertai kajian ini.**

○ **Saya tidak bersetuju untuk menyertai kajian ini.**

For peer review only

Sociodemographic Information

1. Jantina

- ◯ Lelaki
- ◯ Perempuan
- ◯ Lain-lain
- ◯ Lebih suka tidak diberitahu

2. Umur (dalam tahun)

____

3. Status perkahwinan

- ◯ Bujang
- ◯ Mempunya hubungan
- ◯ Berkahwin
- ◯ Tidak tinggal bersama
- ◯ Bercerai
- ◯ Duda/Janda

For peer review only

4. Apakah agama anda?

- ◯ Tiada agama
- ◯ Islam
- ◯ Kristian
- ◯ Hindu
- ◯ Buddha
- ◯ Lain-lain (Sila nyatakan) ____

5. Apakah bangsa anda? (Anda boleh menyebut lebih daripada satu)

____

6. Apakah Bahasa yang paling fasih digunakan dalam kehidupan seharian?

____

Berikut merupakan dua soalan berkenaan kebolehan anda bertutur di dalam Bahasa Inggeris. 6a. Saya faham Bahasa Inggeris dengan baik

- ◯ Sangat tidak setuju 1
- ◯ Tidak setuju 2
- ◯ Natural 3
- ◯ Setuju 4
- ◯ Sangat Setuju 5

For peer review only

For peer review only - http://bmjopen.bmj.com/site/about/guidelines.xhtml

6b. Saya boleh bertutur Bahasa Inggeris dengan baik

- Tidak langsung 1
- Sedikit 2
- Lebih kurang 3
- Agak banyak 4
- Sangat banyak 5

7. Apakah pendidikan tertinggi anda?

- Tiada Pendidikan formal
- Matrikulasi
- Diploma / Sijil
- Sarjana Muda
- Sarjana / PhD

For peer review only

8. Status belajar/bekerja (tandakan yang berkenaan)

- ☐ Pelajar sepenuh masa
- ☐ Pelajar separuh masa
- ☐ Tidak bekerja/Sedang mencari pekerjaan
- ☐ Bekerja sepenuh masa
- ☐ Bekerja separuh masa
- ☐ Bekerja Sendiri sepenuh masa
- ☐ Bekerja Sendiri separuh masa
- ☐ Cuti bergaji
- ☐ Tinggal bersama ibubapa (tidak bergaji)
- ☐ Bersara
- ☐ Lain-lain (Sila nyatakan) ____

9. Pekerjaan

____

10. Pendapatan bulanan isirumah (Ringgit Malaysia)

____

10a. Apakah kadar pendapatan bulanan isirumah, jika dibandingkan dengan purata pendapatan penduduk Malaysia?

- ○ Bawah purata
- ○ Sama dengan purata
- ○ Atas purata

For peer review only

For peer review only - http://bmjopen.bmj.com/site/about/guidelines.xhtml

11. Adakah anda sekarang berada di dalam perintah kawalan pergerakan/arahan duduk di rumah?

- ◯ Ya
- ◯ Tidak

12. Adakahand telah menerima suntikan vaksin?

- ◯ Ya
- ◯ Tidak

12a. Jika belum menerima suntikan vaksin, adakah anda akan mendapatkan vaksin COVID-19 di masa akan datang?

- ◯ Ya
- ◯ Tidak

13. Kita sedar bahawa sesetengah orang telah didedahkan dengan insiden trauma pada masa lampau yang mana telah memudahkan atau menyusahkan untuk menangani masalah. Sebelum pandemic COVID-19, adakah anda pernah menyaksikan atau mengalami mana-mana perkara berikut: (Sila tanda sebanyak mana yang berkaitan)

- ☐ Bencana alam (seperti banjir, gempa bumi dan lain-lain)
- ☐ Tinggal di dalam zon peperangan atau telah terdedah kepada konflik tetera
- ☐ Kesukaran kanak-kanak sebelum usia 16 tahun (seperti pengabaian, buli, serangan fizikal atau seksual)
- ☐ Serangan fizikal atau seksual selepas usia 16 tahun
- ☐ Kemalangan fizikal yang serius
- ☐ Lain-lain - sila nyatakan (pilihan) ____
- ☐ Tiada satu pun di atas

For peer review only - http://bmjopen.bmj.com/site/about/guidelines.xhtml
For peer review only

Covid Psychosocail Impacts Scale

Kesan keatas diri sendiri

Untuk kenyataan di bawah, sila jawab Ya/Tidak jika kesan daripada Covid-19, berlaku kepada diri anda sendiri. Jika jawapan anda adalah Ya, sila nyatakan tahap tekanan yang dialami, berdasarkan skala dibawah.

1. Adakah anda mengalami masalah kesihatan yang boleh menyebabkan risiko anda untuk dijangkiti Covid-19 meningkat?

- ◯ **Tidak 0**
- ◯ **Ya** Tiada tekanan langsung 1
- ◯ **Ya** Tekanan tahap minima 2
- ◯ **Ya** Tekanan tahap sederhana 3
- ◯ **Ya** Tekanan tahap tinggi 4
- ◯ **Ya** Tekanan yang sangat tinggi 5

2. Adakah anda pernah merasakan bahaya risiko terdedah kepada seseorang yang menghidap Covid-19?

- ◯ **Tidak0**
- ◯ **Ya** Tiada tekanan langsung 1
- ◯ **Ya** Tekanan tahap minima 2
- ◯ **Ya** Tekanan tahap sederhana 3
- ◯ **Ya** Tekanan tahap tinggi 4
- ◯ **Ya** Tekanan yang sangat tinggi 5

For peer review only

For peer review only - http://bmjopen.bmj.com/site/about/guidelines.xhtml

3. Adakah anda fikir bahawa anda telah dijangkiti COVID-19?

- **Tidak 0**
- **Ya** Tiada tekanan langsung 1
- **Ya** Tekanan tahap minima 2
- **Ya** Tekanan tahap sederhana 3
- **Ya** Tekanan tahap tinggi 4
- **Ya** Tekanan yang sangat tinggi 5

3a. Adakah anda pernah mendapat keputusan positif bagi ujian COVID-19?

- **Tidak 0**
- **Ya** Tiada tekanan langsung 1
- **Ya** Tekanan tahap minima 2
- **Ya** Tekanan tahap sederhana 3
- **Ya** Tekanan tahap tinggi 4
- **Ya** Tekanan yang sangat tinggi 5

For peer review only

For peer review only - http://bmjopen.bmj.com/site/about/guidelines.xhtml

3b. Adakah anda mempunyai simptom-simptom COVID-19?

- **Tidak 0**
- **Ya** Tiada tekanan langsung 1
- **Ya** Tekanan tahap minima 2
- **Ya** Tekanan tahap sederhana 3
- **Ya** Tekanan tahap tinggi 4
- **Ya** Tekanan yang sangat tinggi 5

Kesan kepada keluarga

Untuk kenyataan di bawah, sila jawab Ya/Tidak jika kesan daripada Covid-19, berlaku kepada ahli keluarga terdekat anda. Jika jawapan anda adalah Ya, sila nyatakan tahap tekanan yang dialami, berdasarkan skala dibawah.

1. Adakah ahli keluarga terdekat anda mengalami masalah kesihatan yang boleh menyebabkan risiko mereka untuk dijangkiti Covid-19 meningkat?

- **Tidak 0**
- **Ya** Tiada tekanan langsung 1
- **Ya** Tekanan tahap minima 2
- **Ya** Tekanan tahap sederhana 3
- **Ya** Tekanan tahap tinggi 4
- **Ya** Tekanan yang sangat tinggi 5

For peer review only

For peer review only - http://bmjopen.bmj.com/site/about/guidelines.xhtml

2. Adakah anda merasakan bahawa setiap ahli keluarga terdekat anda berisiko untuk terdedah kepada seseorang yang menghidap Covid-19?

- **Tidak 0**
- **Ya** Tiada tekanan langsung 1
- **Ya** Tekanan tahap minima 2
- **Ya** Tekanan tahap sederhana 3
- **Ya** Tekanan tahap tinggi 4
- **Ya** Tekanan yang sangat tinggi 5

For peer review only

3. Adakah ahli keluarga terdekat anda pernah menjalani Covid-19?

- **Tidak 0**
- **Ya** Tiada tekanan langsung 1
- **Ya** Tekanan tahap minima 2
- **Ya** Tekanan tahap sederhana 3
- **Ya** Tekanan tahap tinggi 4
- **Ya** Tekanan yang sangat tinggi 5

3a. Adakah keputusan ahli keluarga terdekat anda positif Covid-19?

- **Tidak 0**
- **Ya** Tiada tekanan langsung 1
- **Ya** Tekanan tahap minima 2
- **Ya** Tekanan tahap sederhana 3
- **Ya** Tekanan tahap tinggi 4
- **Ya** Tekanan yang sangat tinggi 5

For peer review only

For peer review only - http://bmjopen.bmj.com/site/about/guidelines.xhtml

3b. Adakah ahli keluarga terdekat anda mempunyai simptom-simptom Covid-19?

- **Tidak 0**
- **Ya** Tiada tekanan langsung 1
- **Ya** Tekanan tahap minima 2
- **Ya** Tekanan tahap sederhana 3
- **Ya** Tekanan tahap tinggi 4
- **Ya** Tekanan yang sangat tinggi 5

3c. Adakah ahli keluarga terdekat anda meninggal dunia disebabkan oleh Covid-19?

- **Tidak 0**
- **Ya** Tiada tekanan langsung 1
- **Ya** Tekanan tahap minima 2
- **Ya** Tekanan tahap sederhana 3
- **Ya** Tekanan tahap tinggi 4
- **Ya** Tekanan yang sangat tinggi 5

For peer review only

For peer review only - http://bmjopen.bmj.com/site/about/guidelines.xhtml

4. Adakah anda mempunyai ahli keluarga terdekat di luar negara yang dikhuatiri berisiko untuk dijangkiti Covid-19?

- **Tidak 0**
- **Ya** Tiada tekanan langsung 1
- **Ya** Tekanan tahap minima 2
- **Ya** Tekanan tahap sederhana 3
- **Ya** Tekanan tahap tinggi 4
- **Ya** Tekanan yang sangat tinggi 5

For peer review only

5. Adakah anda mempunyai ahli di luar negara yang tidak dapat anda lawati atau tidak dapat melawat anda?

- **Tidak 0**
- **Ya** Tiada tekanan langsung 1
- **Ya** Tekanan tahap minima 2
- **Ya** Tekanan tahap sederhana 3
- **Ya** Tekanan tahap tinggi 4
- **Ya** Tekanan yang sangat tinggi 5

For peer review only - http://bmjopen.bmj.com/site/about/guidelines.xhtml

Pekerjaan

Untuk kenyataan di bawah, sila jawab Ya / Tidak / Tidak berkenaan jika kesan daripada Covid-19, berlaku kepada anda atau sesiapa sahaja di rumah anda. Jika jawapan anda adalah Ya, sila nyatakan tahap tekanan yang dialami, berdasarkan skala dibawah.

1. Adakah anda atau sesiapa sahaja di rumah anda dianggap sebagai pekerja perkhidmatan perlu (Contohnya, bidang perubatan dan penjagaan kesihatan, penguatkuasaan undang-undang, perkhidmatan kecemasan, pembekal barang dan perkhidmatan penting)?

- ◯ **Tidak 0**
- ◯ **Ya** Tiada tekanan langsung 1
- ◯ **Ya** Tekanan tahap minima 2
- ◯ **Ya** Tekanan tahap sederhana 3
- ◯ **Ya** Tekanan tahap tinggi 4
- ◯ **Ya** Tekanan yang sangat tinggi 5

2. Adakah anda atau sesiapa sahaja di rumah anda mengalami perubahan yang besar pada waktu bekerja?

- ◯ **Tidak 0**
- ◯ **Ya** Tiada tekanan langsung 1
- ◯ **Ya** Tekanan tahap minima 2
- ◯ **Ya** Tekanan tahap sederhana 3
- ◯ **Ya** Tekanan tahap tinggi 4
- ◯ **Ya** Tekanan yang sangat tinggi 5

For peer review only - http://bmjopen.bmj.com/site/about/guidelines.xhtml
For peer review only

3. Adakah anda atau sesiapa sahaja di rumah anda perlu bertukar tempat kerja dan bekerja dari rumah?

- ◯ **Tidak 0**
- ◯ **Ya** Tiada tekanan langsung 1
- ◯ **Ya** Tekanan tahap minima 2
- ◯ **Ya** Tekanan tahap sederhana 3
- ◯ **Ya** Tekanan tahap tinggi 4
- ◯ **Ya** Tekanan yang sangat tinggi 5

4. Adakah anda atau sesiapa sahaja di rumah anda perlu bertukar jenis pekerjaan?

- ◯ **Tidak 0**
- ◯ **Ya** Tiada tekanan langsung 1
- ◯ **Ya** Tekanan tahap minima 2
- ◯ **Ya** Tekanan tahap sederhana 3
- ◯ **Ya** Tekanan tahap tinggi 4
- ◯ **Ya** Tekanan yang sangat tinggi 5

For peer review only

For peer review only - http://bmjopen.bmj.com/site/about/guidelines.xhtml

5. Adakah anda atau ahli rumahtangga anda telah hilang punca pendapatan?

- **Tidak berkenaan** 9
- **Tidak 0**
- **Ya** Tiada tekanan langsung 1
- **Ya** Tekanan tahap minima 2
- **Ya** Tekanan tahap sederhana 3
- **Ya** Tekanan tahap tinggi 4
- **Ya** Tekanan yang sangat tinggi 5

6. Adakah perniagaan atau sumber pencarian anda atau ahli rumahtangga anda terjejas dan berkemungkinan untuk tidak beroperasi lagi?

- **Tidak berkenaan** 9
- **Tidak 0**
- **Ya** Tiada tekanan langsung 1
- **Ya** Tekanan tahap minima 2
- **Ya** Tekanan tahap sederhana 3
- **Ya** Tekanan tahap tinggi 4
- **Ya** Tekanan yang sangat tinggi 5

For peer review only

Pendapatan

Untuk kenyataan di bawah, sila jawab Ya/Tidak jika kesan daripada Covid-19 ini telah berlaku kepada ahli rumahtangga anda. Jika jawapan anda adalah Ya, sila nyatakan tahap tekanan yang dialami, berdasarkan skala dibawah.

1. Adakah isu kewangan rumahtangga anda merosot dengan mendadak?

- ○ **Tidak 0**
- ○ **Ya** Tiada tekanan langsung 1
- ○ **Ya** Tekanan tahap minima 2
- ○ **Ya** Tekanan tahap sederhana 3
- ○ **Ya** Tekanan tahap tinggi 4
- ○ **Ya** Tekanan yang sangat tinggi 5

2. Adakah rumahtagga anda menghadapi masalah besar untuk membeli barangan keperluan rumah atau membayar bil-bil semasa?

- ○ **Tidak** 0
- ○ **Ya** Tiada tekanan langsung 1
- ○ **Ya** Tekanan tahap minima 2
- ○ **Ya** Tekanan tahap sederhana 3
- ○ **Ya** Tekanan tahap tinggi 4
- ○ **Ya** Tekanan yang sangat tinggi 5

For peer review only

For peer review only - http://bmjopen.bmj.com/site/about/guidelines.xhtml

Perhubungan (bersama insan lain)

Untuk kenyataan di bawah, sila jawab Ya/Tidak jika kesan daripada Covid-19, berlaku kepada diri anda sendiri. Jika jawapan anda adalah Ya, sila nyatakan tahap tekanan yang dialami, berdasarkan skala dibawah. Sebagai tindak balas kepada Covid-19....

1. Adakah ia telah mendatangkan kesusahan dan tekanan kepada keluarga anda?

- ◯ **Tidak** 0
- ◯ **Ya** Tiada tekanan langsung 1
- ◯ **Ya** Tekanan tahap minima 2
- ◯ **Ya** Tekanan tahap sederhana 3
- ◯ **Ya** Tekanan tahap tinggi 4
- ◯ **Ya** Tekanan yang sangat tinggi 5

2. Adakah ia telah mengurangkan perhubungan antara rakan-rakan anda (sama ada secara fizikal ataupun dalam talian)?

- ◯ **Tidak** 0
- ◯ **Ya** Tiada tekanan langsung 1
- ◯ **Ya** Tekanan tahap minima 2
- ◯ **Ya** Tekanan tahap sederhana 3
- ◯ **Ya** Tekanan tahap tinggi 4
- ◯ **Ya** Tekanan yang sangat tinggi 5

For peer review only

Perubahan kepada Rutin dan Tingkah Laku Harian

Untuk kenyataan di bawah, sila jawab Ya/Tidak/Tidak berkenaan jika kesan daripada Covid-19, berlaku kepada diri anda sendiri. Jika jawapan anda adalah Ya, sila nyatakan tahap tekanan yang dialami, berdasarkan skala dibawah. Sebagai tindak balas kepada Covid-19....

1. Adakah wabak Covid ini telah mencetus perubahan kepada rutin harian anda (Contohnya, jadual tidur, senaman, waktu rehat, waktu riadah, atau hobi)?

- ○ **Tidak 0**
- ○ **Ya** Tiada tekanan langsung 1
- ○ **Ya** Tekanan tahap minima 2
- ○ **Ya** Tekanan tahap sederhana 3
- ○ **Ya** Tekanan tahap tinggi 4
- ○ **Ya** Tekanan yang sangat tinggi 5

2. Adakah ia telah membuatkan anda menangguhkan atau mengelakkan mencari rawatan perubatan sekiranya anda atau ahli keluarga anda memerlukannya?

- ○ **Tidak 0**
- ○ **Ya** Tiada tekanan langsung 1
- ○ **Ya** Tekanan tahap minima 2
- ○ **Ya** Tekanan tahap sederhana 3
- ○ **Ya** Tekanan tahap tinggi 4
- ○ **Ya** Tekanan yang sangat tinggi 5

For peer review only

3. Adakah ia telah mengurangkan penglibatan anda dalam aktiviti keagamaan (sama ada secara peribadi atau dalam talian)?

- ◯ **Tidak berknaan** 9
- ◯ **Tidak 0**
- ◯ **Ya** Tiada tekanan langsung 1
- ◯ **Ya** Tekanan tahap minima 2
- ◯ **Ya** Tekanan tahap sederhana 3
- ◯ **Ya** Tekanan tahap tinggi 4
- ◯ **Ya** Tekanan yang sangat tinggi 5

4. Adakah ia telah menyebabkan sebarang perubahan besar kepada rutin harian anda?

- ◯ **Tidak** 0
- ◯ **Ya (sila jelaskan)** 1 ____

Jika jawapan anda adalah Ya, sila nyatakan tahap tekanan yang dialami, berdasarkan skala dibawah.

- ◯ Tiada tekanan langsung 1
- ◯ Tekanan tahap minima 2
- ◯ Tekanan tahap sederhana 3
- ◯ Tekanan tahap tinggi 4
- ◯ Tekanan yang sangat tinggi 5

For peer review only

Pendedahan kepada maklumat tentang Covid-19

1. Apakah sumber maklumat anda berkenaan dengan Covid-19? (Tandakan seberapa banyak yang mungkin)

- ☐ Berita
- ☐ Facebook atau Twitter
- ☐ Laman web kesihatan
- ☐ daripada orang lain
- ☐ Lain-lain (sila jelaskan) ____

2. Sebanyak manakah anda berasa tertekan membaca berita tentang Covid-19?

- ◯ Tiada tekanan langsung 1
- ◯ Tekanan tahap minima 2
- ◯ Tekanan tahap sederhana 3
- ◯ Tekanan tahap tinggi 4
- ◯ Tekanan yang sangat tinggi 5

For peer review only

For peer review only - http://bmjopen.bmj.com/site/about/guidelines.xhtml

Perubahan sejak Covid-19

Untuk soalan-soalan berikutnya, sila nyatakan berdasarkan skala dibawah. Bandingkan keadaan sekarang dengan keadaan semasa sebelum wabak Covid-19 berlaku....

1. Bagaimanakah anda menilai tahap tekanan peribadi anda?

- ◯ Sangat tidak tertekan 1
- ◯ Kurang tertekan 2
- ◯ Lebih kurang sama 3
- ◯ Lebih tertekan 4
- ◯ Sangat tertekan 5

2. Bagaimanakah anda menilai tahap tekanan ahli rumahtangga anda?

- ◯ Sangat tidak tertekan 1
- ◯ Kurang tertekan 2
- ◯ Lebih kurang sama 3
- ◯ Lebih tertekan 4
- ◯ Sangat tertekan 5

3. Bagaimanakah anda menilai tahap kesihatan psikologi anda?

- ◯ Sangat baik 1
- ◯ Baik 2
- ◯ Lebih kurang sama 3
- ◯ Merosot 4
- ◯ Sangat merosot 5

For peer review only

For peer review only - http://bmjopen.bmj.com/site/about/guidelines.xhtml

4. Bagaimanakah anda menilai tahap kesihatan psikologi ahli rumahtangga anda?

- ◯ Sangat baik 1
- ◯ Baik 2
- ◯ Lebih kurang sama 3
- ◯ Merosot 4
- ◯ Sangat merosot 5

For peer review only

For peer review only - http://bmjopen.bmj.com/site/about/guidelines.xhtml

K-10

Sepuluh soalan berikut bertanyakan tentang perasaan yang anda rasai sejak empat minggu yang lepas. Untuk setiap soalan, sila bulatkan pilihan yang menunjukkan sebanyak mana anda merasai perasaan tersebut.

	**Tidak langsung 1**	**Kadang-kadang 2**	**Sedikit 3**	**Selalu 4**	**Sepanjang masa 5**
1. Dalam tempoh empat minggu yang lepas, berapa kerap anda berasa sangat penat tanpa sebarang sebab yang munasabah?	○	○	○	○	○
2. Dalam tempoh empat minggu yang lepas, berapa kerap anda berasa gugup/gementar?	○	○	○	○	○
3. Dalam tempoh empat minggu yang lepas, berapa kerap anda berasa sangat gugup/gementar sehinggga tiada apa yang boleh menenangkan anda?	○	○	○	○	○
4. Dalam tempoh empat minggu yang lepas, berapa kerap anda berasa putus asa?	○	○	○	○	○
5. Dalam tempoh empat minggu yang lepas, berapa kerap anda berasa gelisah?	○	○	○	○	○
6. Dalam tempoh empat minggu yang lepas, berapa kerap anda berasa sangat gelisah sehingga tidak boleh duduk diam?	○	○	○	○	○
7. Dalam tempoh empat minggu yang lepas, berapa	○	○	○	○	○

For peer review only

For peer review only - http://bmjopen.bmj.com/site/about/guidelines.xhtml

kerap anda mengalami perasaan murung?					
8. Dalam tempoh empat minggu yang lepas, berapa kerap anda berasa seperti segala yang dilakukan memerlukan usaha yang banyak?	○	○	○	○	○
9. Dalam tempoh empat minggu yang lepas, berapa kerap anda berasa sangat sedih sehingga tiada apa boleh menceriakan anda?	○	○	○	○	○
10. Dalam tempoh empat minggu yang lepas, berapa kerap anda merasakan tiada harga diri?	○	○	○	○	○

For peer review only

For peer review only - http://bmjopen.bmj.com/site/about/guidelines.xhtml

PCL-5

Berikut adalah senarai masalah yang sering dihadapi oleh seseorang individu sebagai tindak balas kepada suatu pengalaman yang sangat menekankan. Sila baca setiap soalan dengan teliti dan pilih satu nombor yang tertera di sebelah kanan untuk menunjukkan sebanyak mana anda terjejas dengan masalah tersebut pada bulan lalu.

Pada bulan lepas, sebanyak mana anda terganggu dengan:

	Tidak langsung 0	Sedikit 1	Sederhana 2	Sering 3	Sangat terjejas 4
1. Kenangan yang tidak diingini, berulang, dan mengganggu disebabkan oleh pengalaman buruk yang dialami?	○	○	○	○	○
2. Mimpi buruk yang berulang disebabkan oleh pengalaman buruk yang dialami?	○	○	○	○	○
3. Tiba-tiba terasa atau berperangai seolah-olah pengalaman buruk yang dialami itu berulang lagi (seakan-akan anda kembali ke situasi tersebut)?	○	○	○	○	○
4. Merasa sangat marah dan sedih apabila anda diingatkan dengan pengalaman buruk yang dialami?	○	○	○	○	○
5.Mengalami tindak balas fizikal yang kuat apabila anda diingatkan dengan pengalaman buruk yang dialami? (Contohnya jantung berdegup dengan laju, sukar bernafas, dan berpeluh).	○	○	○	○	○
6. Mengelak daripada mengingati sebarang	○	○	○	○	○

For peer review only

For peer review only - http://bmjopen.bmj.com/site/about/guidelines.xhtml

kenangan, fikiran, atau perasaan yang berkaitan dengan pengalaman buruk yang dialami?

7. Mengelak daripada pengaruh luar yang akan mengingatkan kepada pengalaman buruk yang dialami? (Contohnya mengelak daripada berjumpa dengan orang, tempat, perbualan, aktiviti, benda, atau situasi)

8. Adakah anda berasa susah untuk mengingati hal-hal penting pengalaman buruk yang dialami itu?

9.Mempunyai tanggapan negatif yang kuat terhadap diri sendiri, orang lain, atau dunia (Contohnya, mempunyai pemikiran seperti: Saya jahat, ada sesuatu yang sangat salah dengan diri saya, tiada siapa boleh dipercayai, segalanya di dunia ini merbahaya)?

10. Adakah anda menyalahkan diri sendiri atau orang lain kerana berlakunya pengalaman buruk yang telah berlaku atau apa yang terjadi selepas itu?

11. Adakah anda mempunyai perasaan negatif yang kuat seperti takut/cemas, seram, marah, rasa bersalah, atau rasa malu?

For peer review only

For peer review only - http://bmjopen.bmj.com/site/about/guidelines.xhtml

12. Adakah anda hilang rasa minat kepada aktiviti yang pernah anda gemari?	○	○	○	○	○
13. Adakah anda terasa jauh atau terasing daripada orang lain?	○	○	○	○	○
14. Adakah anda sukar untuk merasakan perasaan positif (Contohnya, sukar atau tidak boleh rasa gembira atau perasaan sayang kepada orang terdekat)?	○	○	○	○	○
15. Adakah anda mudah marah, cepat sentap, atau bertindak ganas?	○	○	○	○	○
16. Adakah anda mengambil banyak risiko atau melakukan sesuatu yang boleh membahayakan diri anda?	○	○	○	○	○
17. Menjadi seorang yang sangat berwaspada atau berjaga-jaga?	○	○	○	○	○
18. Mudah rasa terkejut dan gelisah?	○	○	○	○	○
19. Adakah anda menghadapi masalah untuk menumpukan perhatian?	○	○	○	○	○
20. Adakah anda menghadapi masalah untuk tidur dan kekal tidur?	○	○	○	○	○

For peer review only

For peer review only - http://bmjopen.bmj.com/site/about/guidelines.xhtml

WHO-5

Untuk setiap kenyataan di bawah, sila nyatakan perasaan yang paling dirasai sejak dua minggu yang lepas. Dalam tempoh dua minggu yang lepas

	Sepanjang masa 5	Selalu 4	Seringkali (lebih daripada separuh masa) 3	Kadang-kadang (kurang daripada separuh masa) 2	Jarang 1	Tidak pernah 0
1. Saya berasa ceria dan bersemangat	○	○	○	○	○	○
2. Saya berasa tenang dan santai	○	○	○	○	○	○
3. Saya berasa aktif dan bertenaga	○	○	○	○	○	○
4. Saya berasa segar dan cukup rehat apabila bangun pagi	○	○	○	○	○	○
5. Kehidupan seharian saya dipenuhi dengan perkara yang saya minati	○	○	○	○	○	○

For peer review only

For peer review only - http://bmjopen.bmj.com/site/about/guidelines.xhtml

PTGI

Untuk setiap kenyataan di bawah, sila nyatakan sebanyak mana perubahan yang telah berlaku kepada hidup anda sejak peristiwa Covid-19, menggunakan skala di bawah.

	Tidak langsung 0	Sangat sedikit 1	Agak sedikit 2	Sederhana 3	Agak banyak 4	Sangat banyak 5
1. Saya ubah keutamaan terhadap apa yang penting dalam hidup ini.	○	○	○	○	○	○
2. Saya lebih menghargai nilai hidup saya sendiri.	○	○	○	○	○	○
3. Saya dapat memupuk minat yang baru.	○	○	○	○	○	○
4. Perasaan berdikari saya lebih meningkat.	○	○	○	○	○	○
5. Saya lebih memahami perkara kerohanian.	○	○	○	○	○	○
6. Saya dapat lihat yang saya boleh mengharapkan orang lain ketika saya di dalam kesusahan.	○	○	○	○	○	○
7. Saya memulakan perjalanan hidup yang baru.	○	○	○	○	○	○
8. Saya rasa lebih dekat dan akrab dengan orang lain.	○	○	○	○	○	○
9. Saya lebih bersedia untuk meluahkan emosi saya.	○	○	○	○	○	○

For peer review only

For peer review only - http://bmjopen.bmj.com/site/about/guidelines.xhtml

10. Saya yakin bahawa saya boleh melalui saat kesusahan.	○	○	○	○	○	○
11. Saya boleh melakukan perkara yang lebih bermanfaat dalam hidup saya.	○	○	○	○	○	○
12. Saya lebih senang menerima sebarang keputusan kepada perkara yang telah berlaku.	○	○	○	○	○	○
13. Saya dapat lebih bersyukur dan menghargai setiap hari yang ada.	○	○	○	○	○	○
14. Banyak peluang baru muncul yang sukar untuk didapatkan sebelum ini.	○	○	○	○	○	○
15. Rasa belas kasihan saya kepada orang lain meningkat.	○	○	○	○	○	○
16. Saya tingkatkan usaha saya dalam hubungan (bersama orang lain).	○	○	○	○	○	○
17. Saya lebih cenderung untuk mengubah perkara yang perlu diubah.	○	○	○	○	○	○
18. Kepercayaan kepada agama saya semakin meningkat.	○	○	○	○	○	○
19. Saya dapati yang saya lebih kuat	○	○	○	○	○	○

For peer review only

For peer review only - http://bmjopen.bmj.com/site/about/guidelines.xhtml

daripada yang saya sangkakan.						
20. Saya dapat belajar dan ketahui betapa baiknya orang-orang disekeliling saya.	◯	◯	◯	◯	◯	◯
21. Saya dapat terima dengan lebih baik bahawa saya memerlukan orang lain.	◯	◯	◯	◯	◯	◯

Helpline Information

Ingatlah jika anda merasa tertekan dengan kandungan dalam tinjauan ini, sila perhatikan talian bantuan dan perkhidmatan berikut.

Talian Nur Kasih – 15999
Lifeline Association of Malaysia (03-42657995)
Befrienders (03-76272929)
MIASA (03-79321409)
MMHA (0176133039)

For peer review only

For peer review only - http://bmjopen.bmj.com/site/about/guidelines.xhtml

International Study - Somalia

Information Sheet

Daraasad Caalami ah oo lagu baarayo Saamaynta uu Maskaxda ku yeeshay cabuuqa Korona (Covid-19)

Waad ku mahadsan tahay xiisaha aad u muujisay sahankan. Fadlan si taxadar leh u akhri xaanshida macluumaadka inta aadan go’aansan ka hor inaad ka qaybqaadato iyo in kale. Haddii aad go’aansato inaad ka qaybqaadato waad ku mahadsantahay. Haddii aad go’aansato in aadan ka qaybqaadanin wax qasaaro/dhibaato ah kaama soo gaaridoonaan waana kaaga mahadcelineynaa inaad tixgelisay codsigeena.

Waa maxay ujeedada cilmi-baaristaan?
Ujeedada cilmi-baaristaan ayaa ah in la qiimeeyo saameynta uu cabuuqa korona (COVID-19) ku yeeshay maskaxda.

Yaa ka qayb qaadan kara?
Qof kasta oo 18 sano jira ama kawayn ayaa xaq u leh inuu ka qaybqaato daraasadan.

Maxaa ka-qabygalayaasha la weydiin doona inay sameeyaan?
Haddii aad oggolaato inaad ka qaybqaadato cilmi-baaristaan, waxaa lagu weydiin doona inaad dhamaystirto sahan kooban ee online ah oo 15-20 daqiiqo qaadanaayo.

Waa maxay xogta ama macluumaadka la aruurin doono iyo side loo adeegsan doonaa?
Waxaan aruurindoonaa xogta dadweynaha iyo jawaabaha su’aalaha xog aruurinta ah. Xogtan waxaa heli (arki) dono oo kaliya kooxda cilmibaarista. (Waaxda Daawada Cilmi-nafsiga Jaamacada Otago, qeybteeda Christchurch, New Zealand).

Xogta la aruuriyo si amaan ah ayaa loo kaydin doona si kuwa kor ku xusan oo keliya ay u heli karaan. Xogta laga helo natiijada cilmi-baarista waxaa lagu keydindoonaa meel amaan ah **ugu yaraan 5 sano.**
Ma jiridoonto in wax si shakhsi ahaan laguugu aqoonsan kara lagaa aruuriyo mudada xog-aruurintaan.
Natiijooyinka cilmi-baaristaan waxaa laga yaabaa in la daaboco, waxaana laga heli doona maktabada Jaamacada Otago (New Zealand).

Ka-qaybgalayaasha haddii ay is badalaan maka laaban karaan daraasadan?
Waa laga yaabaa inaad ka baxdid daraasadaan inta la dhamaystirin adigoo dhib kaaga imaanin.

Halkee taageero (caawinaad) laga heli karaa?
Qaar ka mid ah su'aalaha waxay ku saabsan yihiin mowduucyo aad u adag waxaana suurtagal ah inaad la kulanto xoogaa dhibaato ah amaba aad dhibsatid. Sida su'aalaha kale oo dhan, jawaabta su'aalahan waa qarsoodi. Qasab kuguma aha inaad ka jawaabto su'aal kasta oo aadan rabin. Haddii aad dareento murugo, fadlan isticmaal lana xiriir ciwaanka laguugu diyaariyay adeegyada **XXXXX**

Wax su’aal ah maka qabtaa arimaha COVID-19? Qeybtaan wadamada weey ku kala duwnaankaraan

Kawaran haddii ka-qeybgalayaasha wax su’aal ah qabaan?
Haddi aad wax su’aal ah aad ka qabtid daraasadan, fadlan nala soo xiriir:
Dr Caroline Bell
Department of Psychological Medicine

For peer review only - http://bmjopen.bmj.com/site/about/guidelines.xhtml

Imayl: caroline.bell@otago.ac.nz

Dr Sandila Tanveer
Department of Psychological Medicine
Imayl: sandila.tanveer@otago.ac.nz

Consent Form

Haddi aad go'aansatay in aad ka qeybqaadato sahankaan fadlan saxiix ogolaanshaha dhamaan weedhaha hoose.

Waan akhriyay, amaba luqadeyda ayaa laygu akhriyay waana fahansanahay warqada macluumaadka ka-qeybqaataha.
Waan la socdaa cidda aan la xiriiri karo haddi aan wax su'aalo ah ka qabo cilmi baaristaan guud ahaan Waxaan fahansanahay in ka-qaybgalkayga cilmi -baaristaan ay tahay ikhtiyaar (ikhtiyaarkeyga) iyo inaan ka noqon karo waqti kasta iyadoon wax ciqaab/dhibaato iiga imaanaynin.
Waxaa la i siiyay macluumaadka muhiim ah ee ku saabsan khadka daryeelka/caawinta haddii ay dhacdo in aan dareemo murugo inta lagu jiro sahanka/wareeysiga.
Waxaan fahansanahay in xogta lagu hayn doono meel aamin ah ugu yaraan shan sano. Waxaan fahansanahay in natiijooyinka sahanka laga yaabo in la daabaco oo laga heli doono Maktabadda Jaamacadda Otago (Dunedin, New Zealand).
Waxaan fahansanahay in kaqaybgalkayga cilmibaaristan uu yahay mid qarsoodi ah oo aysan jirin wax si shakhsi ahaan laigu aqoonsan karo in la iwaydiin karin amaba loo adeegsan doonin warbixin kasta oo ku saabsan cilmibaaristan.

○ **Waan oggolahay inaan ka qaybqaato daraasadda.**

○ **Ma oggoli inaan ka qayb qaato daraasadda.**

For peer review only - http://bmjopen.bmj.com/site/about/guidelines.xhtml

Sociodemographic Information

1. Jinsiga

- ◯ Lab
- ◯ Dheddig
- ◯ Mid Kale
- ◯ Ma jecli inaa ka hadlo

2. Da'daada (Sanad)

____

3. Xaalada guurka

- ◯ Waligeey ma guursan
- ◯ Qof ayaan xiriir la leeyahay
- ◯ Waa guursaday
- ◯ Kala maqanahay
- ◯ Kala tagnay
- ◯ Leeyga dhintay

4. Apa agama yang Anda?

- ◯ Diin la'aan
- ◯ Islam
- ◯ Kristen
- ◯ Hindu
- ◯ Budha
- ◯ Mid kale (Fadlan qeex) ____

For peer review only

For peer review only - http://bmjopen.bmj.com/site/about/guidelines.xhtml

5. Jinsiyadaada maxeey tahay? (waad sheegi kartaa wax ka badan hal)

____

6. Luqadee uugu hadashaa si aad u fiican nolol maalmeedkaada?

____

Labada su'aalo ee soo socda waxa laguugu weydiinayaa heerka luqadaada Ingiriisiga.
6a. Si fiican ayaan Ingirisiga u fahmayaa

- ◯ Aad ayaan uuga soo horjeedaa1
- ◯ Waan diidanahay 2
- ◯ Mana kuugu raacsani mana diidani 3
- ◯ Waan kuugu raacsanahay 4
- ◯ Aad ayaan kuugu raacsanahay 5

6b. Si fiican ayaan Ingiriisiga uugu hadli karaa

- ◯ Maya haba yaraatee 1
- ◯ Kis yar 2
- ◯ Kistoo yar 3
- ◯ Xoogaa 4
- ◯ In aad u badan 5

For peer review only - http://bmjopen.bmj.com/site/about/guidelines.xhtml

6. Waa maxay heerka kuugu sareeyo ee waxbarashada?

- Ma heysto waxbarasho rasmi ah (waxaan ka qeybgalay dugsiga, fasalka ESOL)
- Dugsiga sare (tusale ahaan NCEA,dibloomada IB, shahaadada dugsiga oo dibada)
- Shahaadada heerka sadexaad (tusaale shahaado, Diblooma ama shahaadda ganacsiga - koorso 3 sano ka ayar)
- Shahaadada bajalarka (koorsada 3-4 sanno)
- Shahaadada jaamacada wixi ka sareeya bajalarka (dibloma PG, Mastarka, Dhaqtarnimada)

7. Heerka waxbarashada ama shaqadaada hada (Fadlan muuji mid kastoo ku quseya)

- Waxaan bartaa waqti buuxo
- Waxaan bartaa waqti barkii
- Baagamuudo
- Shaqo waqti buuxo (Full-time)
- Shaqo waqti barkii (Part-time)
- Iskii u shaqeysta waqti buuxa
- Full-time self employed
- Fasaxa waalidnimada (umusha) oo lacagteydana ii socoto
- Waalid guriga jooga (lacag la'aan)
- Ka fariistay
- Wax kale (fadlan noo sheeg) ____

8. Shaqada

____

9. Dakhliga qoyska ee bil walba (sarifka/lacagaha wadanakaada)

____

For peer review only

For peer review only - http://bmjopen.bmj.com/site/about/guidelines.xhtml

9a. Marka la isla barbardhigo dakhliga dhexdhexaadka (isku celceliska), sidee ayaad u qiimeyn lahayd dakhliga qoyskaada?

- ◯ Wuu ka hooseeya dhexdhexaadka
- ◯ Dhexdhexaad (Waa isku celcelis)
- ◯ Wuu ka sareeyaa dhexdhexaadka

10. Miyaad hadda ku jirtaa bandoow ama laguugu amray in aad guriga joogtid?

- ◯ Haa
- ◯ Maya

11. Talaalka COVID-19 maqaadatay?

- ◯ Haa
- ◯ Maya

11a. Hadii aadan Willi is talaalin, ma jeclaan lahayd in aad is talaashid mustaqbalka?

- ◯ Haa
- ◯ Maya

For peer review only

For peer review only - http://bmjopen.bmj.com/site/about/guidelines.xhtml

12. Muuji baayaan walba ee soo socda heerka isbadal ee COVID-19 ku sababay noloshaada, adiga oo isticmaalaaya qiyaasta soo socoto. Halkan waxaa lagu soo koobay dhibaatooyinka ay dadka qaar kala kulmeen COVID-19: (Fadlan noo sheeg intaa kartid)

- ☐ Masiibo dabiici ah (sida fatahaada, dhulgariirka iwm)
- ☐ Ku noolaashada dhul dagaal kasocda ama goob jooga qilaafka militariga
- ☐ Caruurta ka yar 16 oo rafaad kusoo koray (sida dayacaada, xoogsheegsiga, dilka iyo fara xumeeyn)
- ☐ Dil ama kofsi 16 jir ka weyn
- ☐ Dhaawac weyn oo jirka
- ☐ Kuwa kale — fadlan Sii faahfaahi (hadii aad rabtid) ____________________
- ☐ Waxaan oo dhan ma aha

For peer review only - http://bmjopen.bmj.com/site/about/guidelines.xhtml

Covid Psychosocial Impacts Scale

Saameynta Shaqsiga

Bayaanka soo socda, fadlan kaga jawaab Haa/Maya haddii adiga kuugu dhacday arintaan Covid-19 dartiis. Hadday Haa tahay, fadlan sheeg xaddiga walaaca ay tani ku gaarsiisay adigoo hoos ku qayaaseysid lambarka ku habboon.

1. Ma qabtaa xaalad caafimaad oo kaa dhigi karta mid u nugul Covid-19?

- ○ **Maya** 0
- ○ **Haa** Walaac malahan haba yaraatee 1
- ○ **Haa** Walaac yar 2
- ○ **Haa** Walaac dhexdhexaad ah 3
- ○ **Haa** Walaac badan 4
- ○ **Haa** Walaac tiro badan 5

2. Maka cabsatay inaad la kulantid qof qaba Covid-19?

- ○ **Maya** 0
- ○ **Haa** Walaac malahan haba yaraatee 1
- ○ **Haa** Walaac yar 2
- ○ **Haa** Walaac dhexdhexaad ah 3
- ○ **Haa** Walaac badan 4
- ○ **Haa** Walaac tiro badan 5

For peer review only

3. Ma kulatahay in uu COVID-19 kuugu dhacay?

- ◯ **Maya** 0
- ◯ **Haa** Walaac malahan haba yaraatee 1
- ◯ **Haa** Walaac yar 2
- ◯ **Haa** Walaac dhexdhexaad ah 3
- ◯ **Haa** Walaac badan 4
- ◯ **Haa** Walaac tiro badan 5

3a. Waligaa malaaga helay COVID-19?

- ◯ **Maya** 0
- ◯ **Haa** Walaac malahan haba yaraatee 1
- ◯ **Haa** Walaac yar 2
- ◯ **Haa** Walaac dhexdhexaad ah 3
- ◯ **Haa** Walaac badan 4
- ◯ **Haa** Walaac tiro badan 5

3b. Miyaad leedahay astaamaha COVID-19 oo joogta ah?

- ◯ **Maya** 0
- ◯ **Haa** Walaac malahan haba yaraatee 1
- ◯ **Haa** Walaac yar 2
- ◯ **Haa** Walaac dhexdhexaad ah 3
- ◯ **Haa** Walaac badan 4
- ◯ **Haa** Walaac tiro badan 5

For peer review only - http://bmjopen.bmj.com/site/about/guidelines.xhtml

Sameeynta Qoyska

Bayaanka soo socda, fadlan kaga jawaab Haa/Maya haddii arintaan ku dhacday dadka qoyskaada Covid-19 dartiis. Hadday Haa tahay, fadlan sheeg xaddiga walaaca ay tani ku gaarsiisay adigoo hoos ku qayaaseysid lambarka ku habboon.

1. Qof ka mid ah qoyskaada ma jiraa oo u nugul Covid-19 maadaamo ey jirto xaalad caafimaad?

- ◯ **Maya** 0
- ◯ **Haa** Walaac malahan haba yaraatee 1
- ◯ **Haa** Walaac yar 2
- ◯ **Haa** Walaac dhexdhexaad ah 3
- ◯ **Haa** Walaac badan 4
- ◯ **Haa** Walaac tiro badan 5

2. Ma dareensantahay in qof qoyskaada ka mid ah halis u yahay inuu la kulmo qof qaba Covid-19 markuu shaqada joogo?

- ◯ **Maya** 0
- ◯ **Haa** Walaac malahan haba yaraatee 1
- ◯ **Haa** Walaac yar 2
- ◯ **Haa** Walaac dhexdhexaad ah 3
- ◯ **Haa** Walaac badan 4
- ◯ **Haa** Walaac tiro badan 5

For peer review only

3. Ma kulatahay in qof qoyskaada soke ka mid ah in uu ku dhacay COVID-19?

- ◯ **Maya** 0
- ◯ **Haa** Walaac malahan haba yaraatee 1
- ◯ **Haa** Walaac yar 2
- ◯ **Haa** Walaac dhexdhexaad ah 3
- ◯ **Haa** Walaac badan 4
- ◯ **Haa** Walaac tiro badan 5

3a. Ma jiraa qof qoyskaada soke ka mid ah oo laga helay COVID-19?

- ◯ **Maya** 0
- ◯ **Haa** Walaac malahan haba yaraatee 1
- ◯ **Haa** Walaac yar 2
- ◯ **Haa** Walaac dhexdhexaad ah 3
- ◯ **Haa** Walaac badan 4
- ◯ **Haa** Walaac tiro badan 5

3b. Majiraa qof qoyskaada soke ka mid ah ee leh astaamaha COVID-19 oo joogta ah?

- ◯ **Maya** 0
- ◯ **Haa** Walaac malahan haba yaraatee 1
- ◯ **Haa** Walaac yar 2
- ◯ **Haa** Walaac dhexdhexaad ah 3
- ◯ **Haa** Walaac badan 4
- ◯ **Haa** Walaac tiro badan 5

For peer review only - http://bmjopen.bmj.com/site/about/guidelines.xhtml

3c. Majiraa qof qoyskaada soke ah ee u geeriyooday/dhintay COVID-19?

- **Maya** 0
- **Haa** Walaac malahan haba yaraatee 1
- **Haa** Walaac yar 2
- **Haa** Walaac dhexdhexaad ah 3
- **Haa** Walaac badan 4
- **Haa** Walaac tiro badan 5

4. Majiraan dad qoyskaada soke ka mid ah ee dibada ku maqan oo aad uuga walwalsantahay in COVID-19 uu ku dhoco?

- **Maya** 0
- **Haa** Walaac malahan haba yaraatee 1
- **Haa** Walaac yar 2
- **Haa** Walaac dhexdhexaad ah 3
- **Haa** Walaac badan 4
- **Haa** Walaac tiro badan 5

For peer review only

For peer review only - http://bmjopen.bmj.com/site/about/guidelines.xhtml

5. Ma leedahay qoyska dhow dibedda ku sugan oo aadan awoodin inaad booqato ama aan kusoo booqan Karin adiga?

- **Maya** 0
- **Haa** Walaac malahan haba yaraatee 1
- **Haa** Walaac yar 2
- **Haa** Walaac dhexdhexaad ah 3
- **Haa** Walaac badan 4
- **Haa** Walaac tiro badan 5

Shaqo

Bayaanka soo socda, fadlan kaga jawaab Haa/Maya/Ma i qusayso haddii adiga ama qof kula nool ku dhacday Covid-19 dartiis. Hadday Haa tahay, fadlan sheeg xaddiga walaaca ay tani ku gaarsiisay adigoo hoos ku qayaaseysid lambarka ku habboon.

1. Ma idinkujiraa qoyskiina qof loo tixgaliyay in uu yahay shaqaalaha daruuriga (muhiimka ah) (shaqaalaha daryeelka caafimaadka, fulinta sharciga, adeegyada degdegga ah, kuwa bixiyaha adeegyada daruuriga ah)?

- **Maya** 0
- **Haa** Walaac malahan haba yaraatee 1
- **Haa** Walaac yar 2
- **Haa** Walaac dhexdhexaad ah 3
- **Haa** Walaac badan 4
- **Haa** Walaac tiro badan 5

For peer review only

For peer review only - http://bmjopen.bmj.com/site/about/guidelines.xhtml

2. Isbadal weyn ma ku yimid saacadaha aad adiga ama qof qoyskaada ka tirsan aad shaqeyn jirteen?

- **Maya** 0
- **Haa** Walaac malahan haba yaraatee 1
- **Haa** Walaac yar 2
- **Haa** Walaac dhexdhexaad ah 3
- **Haa** Walaac badan 4
- **Haa** Walaac tiro badan 5

3. Ma dhacday in aad adiga ama qof qoyskaada katirsan in aad ku qasbanaateen in goobtii shaqada isku badashay guriga (guriga adigoo joogta sii wadid shaqadii)?

- **Maya** 0
- **Haa** Walaac malahan haba yaraatee 1
- **Haa** Walaac yar 2
- **Haa** Walaac dhexdhexaad ah 3
- **Haa** Walaac badan 4
- **Haa** Walaac tiro badan 5

4. Majiraa qof qoyskaada ka mid ah ee ku qasbanaaday in uu shaqada badasho?

- **Maya** 0
- **Haa** Walaac malahan haba yaraatee 1
- **Haa** Walaac yar 2
- **Haa** Walaac dhexdhexaad ah 3
- **Haa** Walaac badan 4
- **Haa** Walaac tiro badan 5

For peer review only - http://bmjopen.bmj.com/site/about/guidelines.xhtml

5. Majiraa qof qoykaada ka tirsan ee shaqadiisii waayay?

- **Ma i qusayso**
- **Maya** 0
- **Haa** Walaac malahan haba yaraatee 1
- **Haa** Walaac yar 2
- **Haa** Walaac dhexdhexaad ah 3
- **Haa** Walaac badan 4
- **Haa** Walaac tiro badan 5

6. Ganacsi/bisinis aad adiga ama qof kula nool lahaa am aad ka shaqeeyneeysen miyaa halis u ah inuu sii socon waayo?

- **Ma i qusayso**
- **Maya** 0
- **Haa** Walaac malahan haba yaraatee 1
- **Haa** Walaac yar 2
- **Haa** Walaac dhexdhexaad ah 3
- **Haa** Walaac badan 4
- **Haa** Walaac tiro badan 5

For peer review only - http://bmjopen.bmj.com/site/about/guidelines.xhtml

Dakhliga

Bayaanka soo socda, fadlan kaga jawaab Haa/Maya haddii uu qoyskaada quseeya Covid-19 dartiis. Hadday Haa tahay, fadlan sheeg xaddiga walaaca ay tani ku gaarsiisay adigoo hoos ku qayaaseysid lambarka ku habboon.

1. Dhaqaalaha reerkaada aad miyuu hoos u dhacay?

- ◯ **Maya** 0
- ◯ **Haa** Walaac malahan haba yaraatee 1
- ◯ **Haa** Walaac yar 2
- ◯ **Haa** Walaac dhexdhexaad ah 3
- ◯ **Haa** Walaac badan 4
- ◯ **Haa** Walaac tiro badan 5

2. Qoyskiina miyaad awoodi wayseen inaad bixisaan biilasha ama adeega cuntada?

- ◯ **Maya** 0
- ◯ **Haa** Walaac malahan haba yaraatee 1
- ◯ **Haa** Walaac yar 2
- ◯ **Haa** Walaac dhexdhexaad ah 3
- ◯ **Haa** Walaac badan 4
- ◯ **Haa** Walaac tiro badan 5

For peer review only

Xiriika

Bayaanka soo socda, fadlan kaga jawaab Haa/Maya haddii adiga kuugu dhacday arintaan Covid-19 dartiis. Hadday Haa tahay, fadlan sheeg xaddiga walaaca ay tani ku gaarsiisay adigoo hoos ku qayaaseysid lambarka ku habboon. Covid-19 dartiis.....

1. Dhibaatada qoyska miyeey sii adkaatay?

- ◯ **Maya** 0
- ◯ **Haa** Walaac malahan haba yaraatee 1
- ◯ **Haa** Walaac yar 2
- ◯ **Haa** Walaac dhexdhexaad ah 3
- ◯ **Haa** Walaac badan 4
- ◯ **Haa** Walaac tiro badan 5

2. Xiriirkii aad la lahayd asxaabtaada miyuu soo yaraaday (si toos ah ama dhinaca qadka)?

- ◯ **Maya** 0
- ◯ **Haa** Walaac malahan haba yaraatee 1
- ◯ **Haa** Walaac yar 2
- ◯ **Haa** Walaac dhexdhexaad ah 3
- ◯ **Haa** Walaac badan 4
- ◯ **Haa** Walaac tiro badan 5

For peer review only

Isbeddelka ku yimid nolol maalmeedka iyo dabeecadaha

Bayaanka soo socda, fadlan kaga jawaab Haa/Maya haddii adiga kuugu dhacday arintaan Covid-19 dartiis. Hadday Haa tahay, fadlan sheeg xaddiga walaaca ay tani ku gaarsiisay adigoo hoos ku qayaaseysid lambarka ku habboon. Covid-19 dartiis…..

1. Cabuqa Korona (Covid-19) miyuu isbadal ku keenay nolol maalmeedkaada (sida hurdada, jimicsiga, firaaqada, hiwaayadaha aad xiiseeysid)?

- ◯ **Maya** 0
- ◯ **Haa** Walaac malahan haba yaraatee 1
- ◯ **Haa** Walaac yar 2
- ◯ **Haa** Walaac dhexdhexaad ah 3
- ◯ **Haa** Walaac badan 4
- ◯ **Haa** Walaac tiro badan 5

2. Miyay taasi sababtay inaad dib u dhigto ama iska dhaaftid inaad raadsato daryeel caafimaad mar ad adiga ama qof qoyskaada u baahateen?

- ◯ **Maya** 0
- ◯ **Haa** Walaac malahan haba yaraatee 1
- ◯ **Haa** Walaac yar 2
- ◯ **Haa** Walaac dhexdhexaad ah 3
- ◯ **Haa** Walaac badan 4
- ◯ **Haa** Walaac tiro badan 5

For peer review only

3. Tani miyay yareeysay ka-qayb-qaadashadaada howlaha diinta (shaqsi ahaan ama khadka tooska ah)?

- ◯ **Ma i qusayso**
- ◯ **Maya** 0
- ◯ **Haa** Walaac malahan haba yaraatee 1
- ◯ **Haa** Walaac yar 2
- ◯ **Haa** Walaac dhexdhexaad ah 3
- ◯ **Haa** Walaac badan 4
- ◯ **Haa** Walaac tiro badan 5

4. Miyey jireen isbeddelo kale oo weyn oo ku yimid nidaamka/nolol maalmeedka?

- ◯ **Maya** 0
- ◯ **Haa (Haddii ay sidaas tahay fadlan sheeg)** 1 ____

Hadday Haa tahay, fadlan sheeg xaddiga walaaca ay tani ku gaarsiisay adigoo hoos ku qayaaseysid lambarka ku habboon.

- ◯ Walaac malahan haba yaraatee 1
- ◯ Walaac yar 2
- ◯ Walaac dhexdhexaad ah 3
- ◯ Walaac badan 4
- ◯ Walaac tiro badan 5

For peer review only - http://bmjopen.bmj.com/site/about/guidelines.xhtml

Soo bandhigista macluumaadka ku saabsan Covid-19

1. Xageed kaheshaa warbixinta ku saabsan Covid-19?
(Fadlan qeex mid kasto ad isticmaashid)

- ☐ News/wararka
- ☐ Facebook / Twitter
- ☐ Websites/boga caafimaadka
- ☐ Hadal isgaarsiis
- ☐ Meela kale (Fadlan qeex) ____

2. Culeys ilaa intee ayuu wararka ku saabsan Covid-19 kuu geystay?

- ○ Walaac malahan haba yaraatee 1
- ○ Walaac yar 2
- ○ Walaac dhexdhexaad ah 3
- ○ Walaac badan 4
- ○ Walaac tiro badan 5

Isbeddelada ku yimid Covid-19 dartiis

Arimaha soo socda fadlan sheeg xaddiga walaaca ay tani ku gaarsiisay adigoo hoos ku qayaaseysid lambarka ku habboon. Isbarbardhiga hadda iyo wixii ka horeeyay Covid-19---

1. Sideed u qiimeyn lahayd heerka walaacaaga shaqsi ahaan?

- ○ Walaac yar 1
- ○ Xoogaa walaac yar 2
- ○ Islameeshii 3
- ○ Walwal batay 4
- ○ Walaaca oo aad u batay 5

For peer review only

2. Maxaad ku qiimeyn lahayd walaaca guud ee reerkaada/gurigaada?

- ◯ Walaac yar 1
- ◯ Xoogaa walaac yar 2
- ◯ Islameeshii 3
- ◯ Walwal batay 4
- ◯ Walaaca oo aad u batay 5

3. Sideed u qiimeyn lahayd ladnaantaada nafsiyeed?

- ◯ Aad uuga fiican sidii hore 1
- ◯ Sidii hore ka fiican 2
- ◯ Sideedii 3
- ◯ Sidii ka daran 4
- ◯ Aad uga daran 5

4. Sideed u qiimeyn laheyd wanaagsanaanta nafsiyeed ee dadka qoyskaaga?

- ◯ Aad ufiican 1
- ◯ Fiican 2
- ◯ Ku saabsan isla mid 3
- ◯ Ka xun 4
- ◯ Aad uga xun 5

For peer review only

For peer review only - http://bmjopen.bmj.com/site/about/guidelines.xhtml

K-10

Tobanka su'aalo ee xiggo waxay wax ka weydiinayaan sida aad dareemeysay 4 isbuuc ee la soo dhaafay. Su'aal walba, tilmaam intajeer aad dareentay qaabkaas.

	Marnaba<br>1	Mar dhif<br>2	Mar mar<br>3	Inta badan<br>4	Mar kasta<br>5
1. Afartii isbuuc ee la soo dhaafay, intee jeer ayaad dareentay daal oo aan lahayn sabab wanaagsan?	○	○	○	○	○
2. Afartii isbuuc ee la soo dhaafay, intee jeer ayaad dareentay walwal?	○	○	○	○	○
3. Afartii isbuuc ee la soo dhaafay, intee jeer ayaad dareentay walwal zaa'id ah oo waxba aanan ku dajineyn?	○	○	○	○	○
4. Afartii isbuuc ee la soo dhaafay, intee jeer ayaad dareentay rajo la' aan?	○	○	○	○	○
5. Afartii isbuuc ee la soo dhaafay, intee jeer ayaad dareentay nasasho la'aan ama samir la'aan?	○	○	○	○	○
6. Afartii isbuuc ee la soo dhaafay, intee jeer ayaad dareentay nasasho la'aan oo aadan fadhin karin?	○	○	○	○	○
7. Afartii isbuuc ee la soo dhaafay, intee jeer ayaad dareentay murugo?	○	○	○	○	○
8. Afartii isbuuc ee la soo dhaafay, intee jeer ayaad dareentay in wax walba ay dadaal/halgan ahaayeen?	○	○	○	○	○
9. Afartii isbuuc ee la soo dhaafay, intee jeer ayaad dareentay murugo badan oo waxba aanan ku farxad gelineynin?	○	○	○	○	○

For peer review only

For peer review only - http://bmjopen.bmj.com/site/about/guidelines.xhtml

10. Afartii isbuuc ee la soo dhaafay, intee jeer ayaad dareentay qiimo la'aan?	○	○	○	○	○

For peer review only

For peer review only - http://bmjopen.bmj.com/site/about/guidelines.xhtml

PCL-5

Hoos waa liiska dhibaatooyinka oo dadka mararka qaar ay qabaan oo jawaab u ah waaya aragnimada walbahaarka badan. Fadlan si taxadar ah u aqri dhibaato walba kadibna tilmaam inta ay ku dhibtay dhibaatadaas bishii la soo dhaafay. Bisha la soo dhaafay, intee ayay ku dhibtay:

	Maya gabi ahan 0	Xooga yar 1	Dhexaad 2	Ilaa xad 3	Xad dhaaf ah 4
1. Soo nonoqoshada xasuusta aan la rabin oo khibradaha argagaxa leh?	○	○	○	○	○
2. Soo nonoqoshada riyooyinka jahwareerka ah ee ku saabsan khibradaha argagaxa leh?	○	○	○	○	○
3. Dareen lama filaan ah ama u dhaqanka sidii ineey hada khibradii murugada lahayd dhaceysa markale (sidii ineey hada dhaceeyso oo kale)?	○	○	○	○	○
4. Dareenka murugada aadka ah markii wax ku soo xasuusiyaan waaya aragnimada murugada leh?	○	○	○	○	○
5. Falcelinada jirka xoogan markii wax ku soo xasuusiyaan waaya aragnimadii murugada lahyd (tusaale ahaan, garaaca wadnaha,neefsashada oo dhib noqota iyo dhididka)?	○	○	○	○	○
6. Ka fogaanshaha xasuusta, afkaaraha, ama dareemida la xiriirto waaya aragnimada/khibradaha murugada leh?	○	○	○	○	○
7. Ka fogaanshaha wax kastoo kusoo xasuusiya walbahaarkii (tusaala ahaan, dadka, meelaha, wadasheekeysiga, shaqooyinka, waxyaabaha ama xaaladaha)?	○	○	○	○	○

For peer review only - http://bmjopen.bmj.com/site/about/guidelines.xhtml

8. Miyaad dhib ku qabtaa inaa xasuusatid qeyba muhiim ah ee khibradihii walbahaarka lahaa?

9. Ka qabida aaminaad xun oo xoogan nafsadaada, dadka kale, ama caalamka (tusaale ahaan, lahaanshaha afkaaraha sida; Waan xummahay, wax baa iga qaldan, majiro qof la aamini karo, caalamka oo dhan waa halis)?

10. Ku eedeynta nafsadaada ama qof kale ee khibradihii walbahaarka lahaa ama wixii dhacay markaas kadib?

11. Lahaanshaha dareen xun ee xoogan sida cabsida, naxdinta, xanaaqa, dambiilenimada, ama ceeb?

12. Ka xiisadhicida howlihii aad jeclaan jirtay?

13. Dareemida ka fogaanshaha ama ka go'ida dadka kale?

14. Dhibaato ma ku qabtaa inaad dareento dareen wanaagsan (tusaale ahaan, inaad dareento farxad ama aad jeclaato dadka kuu dhow)?

15. Anshax xumo, xanaaqa, ama ku dhaqmida si dagaal ah?

16. Halis is galis badan ama sameeynta waxyaabaha kuugu sababi karo dhibaato?

17. Ma dareentaa "feejignaan cajiib ah" ama fiirin badan ama illaalo?

18. Miyaad dareentaa xasilaad la'aan ama si sahlan inaad u naxdo?

For peer review only

19. Ma kuugu adagtahay inaad diirad saartid/xogsaartid?	○	○	○	○	○
20. Ma kuugu adagtahay ineey hurdo kaa soo dhacdo ama inaad sii hurudo?	○	○	○	○	○

WHO-5

Fadlan noo tilmaam shanta arimood ee soo socda sida ugu dhow uu dareenkaaga ahaa labadii isbuuc ee la soo dhaafay. Labadii isbuuc ee lasoo dhaafay

	**Marwalba** 5	**Intabadan wakhtiga** 4	**Inkabadan kalabar wakhtiga** 3	**Inkayar kalabar wakhtiga** 2	**Marmar wakhtiga** 1	**Marnaba** 0
1. Waxaan dareemay farxad iyo hami sare.	○	○	○	○	○	○
2. Waxaan dareemay daganaan iyo xasilooni.	○	○	○	○	○	○
3. Waxaan dareemay firfircooni iyo awoodbadan.	○	○	○	○	○	○
4. Waxaan soo kacay anigoo dareemaya nasasho iyo daganaan.	○	○	○	○	○	○
5. Maalintayda oo dhan waxaa buuxiyay waxyalo ixiiso galiya.	○	○	○	○	○	○

For peer review only - http://bmjopen.bmj.com/site/about/guidelines.xhtml

PTGI

Muuji baayaan walba ee soo socda heerka isbadal ee Maarso 15-keedii ku sababay noloshaada, adoo isticmaalaaya qiyaasta soo socoto.

	Marnaba 0	Wax yar 1	Xoogaa Roon 2	Dhexaad 3	In badan 4	Wax badan 5
1. Waxaan badelay muhiimadeyda ku saabsan waxa nolosha muhiim u ah.	○	○	○	○	○	○
2. Waxaan qadarin weyn u leeyahay qiimaha noloshayda.	○	○	○	○	○	○
3. Waxaan hormariyay xiiso/dana cusub.	○	○	○	○	○	○
4. Waxaan dareemayaa isku kalsooni.	○	○	○	○	○	○
5. Waxaan si fiican uu fahamsanahay arrimaha diinta.	○	○	○	○	○	○
6. Waxaan si cad u arkaa inaan ku xisaabtami karo dadka waqtiyada dhibaatada.	○	○	○	○	○	○
7. Waxaan nolosheyda u aasaasay wado cusub.	○	○	○	○	○	○
8. Waxaan dareensanahay inaan dadka aad uugu dhawahay.	○	○	○	○	○	○
9. Waxaan diyaar u ahay inaan muujiyo dareenkeyga.	○	○	○	○	○	○

For peer review only

10. Waxaan si fiican u ogahay in aan wax ka qaban karo dhibaatoyinka.	◯	◯	◯	◯	◯	◯
11. Waxaan awooda in aan noloshayda wax fiican ku sameeyo.	◯	◯	◯	◯	◯	◯
12. Waxaan si fiican u awoodaa inaan aqbalo siday wax u dhacayaan.	◯	◯	◯	◯	◯	◯
13. Waxaan si fiican uga mahadcelin karaa maalin kasta.	◯	◯	◯	◯	◯	◯
14. Fursado cusub ayaa la heli karaa oo awal jirin.	◯	◯	◯	◯	◯	◯
15. Waxaan u qabaa naxariis badan dadka kale.	◯	◯	◯	◯	◯	◯
16. Waxaan dadaal badan geliya cilaaqaadkeyga.	◯	◯	◯	◯	◯	◯
17. Waxay u badan tahay inaan isku dayo inaan beddelo waxyaabaha u baahan badal.	◯	◯	◯	◯	◯	◯
18. Waxaan leeyahay caqiido diineed oo xoog badan.	◯	◯	◯	◯	◯	◯
19. Waxaan ogaaday inaan ka xoog badanahay sidaan u maleeynayay.	◯	◯	◯	◯	◯	◯

For peer review only

20. Waxaan bartay ineey dadka aad u wanaagsan yihiin.	○	○	○	○	○	○
21. Waxaan si fiican u qirraa inaan u baahankaro dadka kale.	○	○	○	○	○	○

Helpline Information

Remember if you are feeling distressed by any of the content in this survey, please feel free to reach out to the following people.

Aaron Moratz, M.A., Dean of Postgraduate Studies, University of Burao, +61 416 234 324

Lori Zoellner, PhD, Department of Psychology, University of Washington, +1 206-685-3126

Websites that provide resources for those who have concerns about harming themselves or others:

Hotline Numbers:

https://www.suicidestop.com/call_a_hotline.html

International Association for Suicide Prevention website:

https://www.iasp.info/resources/Crisis_Centres

For peer review only

For peer review only - http://bmjopen.bmj.com/site/about/guidelines.xhtml

International Study - Turkish

Information Sheet

Covid-19 Pandemisinin Psikososyal Etkilerini İnceleyen Uluslararası Bir Çalışma

Değerli Katılımcılar,
Öncelikle bu çalışmaya gösterdiğiniz ilgi için teşekkür ederiz. Lütfen katılıp katılmamaya karar vermeden önce bu bilgi sayfasını dikkatlice okuyunuz. Çalışmaya katılmanız ya da katılmamanız size herhangi bir sorumluluk getirmeyecektir. Çalışmaya katılarak vereceğiniz destek için şimdiden teşekkür ederiz.

Çalışmanın amacı nedir?
Çalışmanın amacı, Covid-19 pandemisinin psikososyal etkilerini değerlendirmektir.

Kimler katılabilir?
18 yaş ve üzeri herkes bu çalışmaya katılabilir.

Katılımcılardan ne yapmaları istenecek?
Bu çalışmaya katılmayı kabul ederseniz, 15-20 dakika sürecek kısa bir çevrimiçi/online anket doldurmanız istenecektir.

Hangi veriler veya bilgiler toplanacak ve bunlardan ne şekilde yararlanılacak?
Çalışma kapsamında demografik veriler ve bir dizi ankete verilen yanıtlar toplanacaktır. Bu verilere yalnızca araştırma ekibi (Psikolojik Tıp Anabilim Dalı, Otago Üniversitesi, Christchurch Yeni Zelanda) tarafından erişilecektir.

Toplanan verilere, yalnızca aşağıda belirtilen kişiler erişebilecek ve veriler güvenli bir şekilde en az 5 yıl süreyle saklanacaktır.

Bu çalışmada kisisel bilgileriniz istenmeyecektir. Bu araştırmanın sonuçları yayınlanabilecek ve sonuçlar Otago Universitesi Kütüphanesin'de (Dunedin, Yeni Zelanda) bulunacaktır.

Katılımcılar fikirlerini değiştirebilir ve projeden çekilebilir mi?
Katılımcılar anketleri tamamlamadan çalışamadan ayrılma hakkına sahiptir. Çalışmadan ayrılmak size herhangi bir sorumluluk ve ceza getirmemektedir.

Nereden psikososyal destek alınabilir?
Sağlık Bakanlığına ait psikososyal destek hatları ilden ile değişiklik göstermektedir.

Gaziantep İl Sağlık Müdürlüğü: 03422200505/Dahili:1142-1145 / 0342 517 52 58/Dahili:113
Bulunduğunuz ilin psikososyal destek hatlarına Sağlık Bakanlığı'na ait aşağıdaki linkten ulaşabilirsiniz.
https://covid19.saglik.gov.tr/TR-66158/81-il-psikososyal-destek-hat-bilgileri.html

For peer review only

COVID-19 hakkında sorularınız varsa kiminle iletişime geçebilirsiniz?

COVID-19 ile ilgili bilgi ve yardım almak isterseniz Sağlık Bakanlığı İletişim Merkezi'ne ait ALO 184 Koronavirüs Dayanışma hattını arayabilirsiniz.

Katılımcılar soruları için kimlerle iletişime geçebilir?

Çalışmamızla ilgili herhangi bir sorunuz varsa, lütfen bizimle iletişime geçmekten çekinmeyin:

Dr. Saadet YAPAN
Psikoloji Bölümü
E-posta Adresi: saadet.yapan@hku.edu.tr

Arş. Gör. Feyza TOPÇU
Psikoloji Bölümü
E-posta Adresi: feyza.topcu@hku.edu.tr

For peer review only

Consent Form

Bu çalışmaya katılmayı kabul ediyorsanız, lütfen aşağıdaki tüm ifadeler için onay veriniz.

- Bunu ana dilimde okudum veya bana okundu, Katılımcı Bilgi Formunu okudum anlıyorum.
- Genel olarak çalışma hakkında herhangi bir sorum olursa kiminle iletişime geçeceğimi biliyorum.
- Bu çalışmaya katılmanın gönüllü olduğunu (benim seçimim) ve herhangi bir zamanda herhangi bir ceza almadan çalışmadan çekilebileceğimi anlıyorum.
- Anketleri doldururken sıkıntı yaşamam durumunda nereden yardım alabileceğim hakkında gerekli bilgiler tarafıma verildi.
- Verilerin en az beş yıl boyunca güvenli bir şekilde tutulacağını anlıyorum.
- Çalışma sonuçlarının yayınlanabileceğini ve Otago Üniversitesi Kütüphane'sinde (Dunedin, Yeni Zelanda) bulunacağını anlıyorum.
- Bu çalışmaya katılımımın gizli olduğunu ve bu çalışmayla ilgili herhangi bir raporda beni kişisel olarak tanımlayabilecek hiçbir bilginin ve materyalin alınmayacağını veya kullanılmayacağını anlıyorum.

◯ **Çalışmaya katılmayı kabul ediyorum.**

◯ **Çalışmaya katılmayı kabul etmiyorum.**

For peer review only

For peer review only - http://bmjopen.bmj.com/site/about/guidelines.xhtml

Sociodemographic Information

1. Cinsiyet

◯ Erkek

◯ Kadın

2. Yaş

____

3. Medeni Hali

◯ Bekar

◯ İlişkisi var

◯ Evli

◯ Ayrılmış

◯ Boşanmış

◯ Dul

4. Dininiz nedir?

◯ Din yok

◯ İslâm

◯ Hıristiyanlık

◯ Hinduizm

◯ Budizm

◯ Diğer (lütfen belirtiniz) ____

For peer review only

For peer review only - http://bmjopen.bmj.com/site/about/guidelines.xhtml

5. Eğitim düzeyiz nedir?(En son almış olduğunuz derece)?

- ◯ Okur Yazar
- ◯ İlkokul Mezunu
- ◯ Ortaokul Mezunu
- ◯ Lise Mezunu
- ◯ Üniversite Öğrencisi
- ◯ Önlisans Mezunu
- ◯ Lisans Mezunu
- ◯ Yüksek Lisans Mezunu
- ◯ Doktora Mezunu

6. Mevcut Çalışma veya iş durumunuz (Birden fazla işaretleyebilirsiniz.)

- ☐ Öğrenci
- ☐ Çalışmıyor/İş Arıyor
- ☐ Tam zamanlı maaşlı çalışma
- ☐ Yarı zamanlı maaşlı çalışma
- ☐ Tam zamanlı serbest meslek
- ☐ Yarı zamanlı serbest meslek
- ☐ Ücretli ebeveyn izninde
- ☐ Evde ebeveyn yada ücretsiz ebeveyn izninde
- ☐ Emekli
- ☐ Diğer (Lütfen Belirtin) ____

For peer review only

For peer review only - http://bmjopen.bmj.com/site/about/guidelines.xhtml

7. Mesleğiniz

____

8. Aylık hane geliri (yerel para biriminde)

____

9. Ortalama gelirle karşılaştırıldığında, hanenizin gelirini nasıl değerlendirirsiniz?

◯ Ortalamanın altında

◯ Ortalama

◯ Ortalamanın üstünde

10. Şu anda karantinada ya da sokağa çıkma yasağında mısınız?

◯ Evet1

◯ Hayır0

11. Covid-19 aşısı oldunuz mu?

◯ Evet1

◯ Hayır0

11a. Aşı olmadıysanız, gelecekte Covid-19 aşısı olmak ister misiniz?

◯ Evet1

◯ Hayır0

For peer review only

For peer review only - http://bmjopen.bmj.com/site/about/guidelines.xhtml

12. Bazı insanların geçmişte maruz kaldıkları diğer travmatik olayların yeni sorunlarla başa çıkmayı kolaylaştırabilecek ya da zorlaştırabilecek olduğunun farkındayız.

Covid-19 salgınından önce aşağıdakilerden herhangi birine şahit oldunuz mu veya herhangi birini yaşadınız mı?: **(Lütfen uygun olanları işaretleyin)**

- ☐ Doğal afet (sel, deprem, vs.)
- ☐ Savaş bölgesinde yaşamak veya askeri çatışmaya maruz kalmak
- ☐ 16 yaş öncesi çocukluk sıkıntısı (mesela ihmal, zorbalık, fiziksel veya cinsel saldırı, vs.)
- ☐ 16 yaş sonrası fiziksel veya cinsel saldırı
- ☐ Ciddi fiziksel kazalar
- ☐ Diğer – isteğe bağlı – bunun ne olduğunu açıklayabilir misiniz lütfen?
  ____
- ☐ Yukarıdakilerin hiçbiri

For peer review only

For peer review only - http://bmjopen.bmj.com/site/about/guidelines.xhtml

Covid Psychosocial Impacts Scale

Kişisel etki

Aşağıdaki ifadeler için, Covid-19 döneminde sizde böyle bir durum gerçekleştiyse lütfen Evet / Hayır ile yanıt verin. Evet ise, lütfen aşağıdaki ölçekte bunun sizde neden olduğu stres miktarını belirtin.

1. Covid-19'dan daha olumsuz etkilenmenize neden olabilecek bir sağlık probleminiz var mı (örn. Kalp hastalığı, nefes almada zorluk, zayıf bağışıklık sistemi, veya kanser)?

- **Hayır0**
- **Evet** Hiç stresli değil 1
- **Evet** Biraz stres 2
- **Evet** Orta derecede stres 3
- **Evet** Oldukça fazla stres 4
- **Evet** Aşırı derecede stres 5

2. Covid-19 pozitif biriyle temas riski altında olduğunuzu hissettiniz mi?

- **Hayır0**
- **Evet** Hiç stresli değil 1
- **Evet** Biraz stres 2
- **Evet** Orta derecede stres 3
- **Evet** Oldukça Fazla stres 4
- **Evet** Aşırı derecede stres 5

For peer review only

3. Covid-19 geçirdiğinizi düşünüyor musunuz?

- ◯ **Hayır 0**
- ◯ **Evet** Hiç stresli değil 1
- ◯ **Evet** Biraz stres 2
- ◯ **Evet** Orta derecede stres 3
- ◯ **Evet** Oldukça fazla stres 4
- ◯ **Evet** Aşırı derecede stres 5

3a. Covid-19 tanısı aldınız mı?

- ◯ **Hayır 0**
- ◯ **Evet** Hiç stresli değil 1
- ◯ **Evet** Biraz stres 2
- ◯ **Evet** Orta derecede stres 3
- ◯ **Evet** Oldukça fazla stres 4
- ◯ **Evet** Aşırı derecede stres 5

3b. Devam eden Covid-19 semptomlarınız var mı?

- ◯ **Hayır 0**
- ◯ **Evet** Hiç stresli değil 1
- ◯ **Evet** Biraz stres 2
- ◯ **Evet** Orta derecede stres 3
- ◯ **Evet** Oldukça fazla stres 4
- ◯ **Evet** Aşırı derecede stres 5

For peer review only

For peer review only - http://bmjopen.bmj.com/site/about/guidelines.xhtml

Aile etkisi

Aşağıdaki ifadeler için, Covid-19 döneminde yakın aile üyelerinizde böyle bir durum gerçekleştiyse lütfen Evet / Hayır ile yanıt verin. Evet ise, lütfen aşağıdaki ölçekte bunun sizde neden olduğu stres miktarını belirtin.

1. Herhangi bir yakın aile üyesinin Covid-19'dan daha olumsuz etkilenmelerine neden olabilecek bir sağlık problemi var mı? (örn. Kalp hastalığı, nefes almada zorluk, zayıf bağışıklık sistemi, veya kanser)?

- ◯ **Hayır 0**
- ◯ **Evet** Hiç stresli değil 1
- ◯ **Evet** Biraz stres 2
- ◯ **Evet** Orta derecede stres 3
- ◯ **Evet** Oldukça fazla stres 4
- ◯ **Evet** Aşırı derecede stres 5

2. Pandemi sürecinde herhangi bir yakın aile üyesinin, Covid-19 pozitif olan biri ile temas riski altında olduğunu düşündünüz mü/düşünüyor musunuz?

- ◯ **Hayır 0**
- ◯ **Evet** Hiç stresli değil 1
- ◯ **Evet** Biraz stres 2
- ◯ **Evet** Orta derecede stres 3
- ◯ **Evet** Oldukça fazla stres 4
- ◯ **Evet** Aşırı derecede stres 5

For peer review only

3. Yakın aile üyelerinizden herhangi birinin Covid-19 geçirdiğini düşünüyor musunuz?

- ◯ **Hayır 0**
- ◯ **Evet** Hiç stresli değil 1
- ◯ **Evet** Biraz stres 2
- ◯ **Evet** Orta derecede stres 3
- ◯ **Evet** Oldukça fazla stres 4
- ◯ **Evet** Aşırı derecede stres 5

3a. Yakın aile üyelerinin herhangi biri Covid-19 tanısı aldı mı?

- ◯ **Hayır 0**
- ◯ **Evet** Hiç stresli değil 1
- ◯ **Evet** Biraz stres 2
- ◯ **Evet** Orta derecede stres 3
- ◯ **Evet** Oldukça fazla stres 4
- ◯ **Evet** Aşırı derecede stres 5

3b. Yakın aile üyesi/üyelerinde devam eden Covid-19 semptomları var mı?

- ◯ **Hayır 0**
- ◯ **Evet** Hiç stresli değil 1
- ◯ **Evet** Biraz stres 2
- ◯ **Evet** Orta derecede stres 3
- ◯ **Evet** Oldukça fazla stres 4
- ◯ **Evet** Aşırı derecede stres 5

For peer review only - http://bmjopen.bmj.com/site/about/guidelines.xhtml
For peer review only

3c. Yakın bir aile üyesi/üyeleri Covid-19 nedeniyle vefat etti mi?

- ◯ **Hayır 0**
- ◯ **Evet** Hiç stresli değil 1
- ◯ **Evet** Biraz stres 2
- ◯ **Evet** Orta derecede stres 3
- ◯ **Evet** Oldukça fazla stres 4
- ◯ **Evet** Aşırı derecede stres 5

4. Yurtdışında Covid-19'a yakalanma riski altında olmasından endişe duyduğunuz yakın aile üyeniz var mı?

- ◯ **Hayır 0**
- ◯ **Evet** Hiç stresli değil 1
- ◯ **Evet** Biraz stres 2
- ◯ **Evet** Orta derecede stres 3
- ◯ **Evet** Oldukça fazla stres 4
- ◯ **Evet** Aşırı derecede stres 5

For peer review only

For peer review only - http://bmjopen.bmj.com/site/about/guidelines.xhtml

5. Covid-19 pandemisi nedeniyle yurtdışında ziyaret edemediğiniz veya sizi ziyaret edemeyen yakın aile üyeniz var mı?

- **Hayır 0**
- **Evet** Hiç stresli değil 1
- **Evet** Biraz stres 2
- **Evet** Orta derecede stres 3
- **Evet** Oldukça fazla stres 4
- **Evet** Aşırı derecede stres 5

İş

Aşağıdaki ifadeler için, Covid-19 döneminde bu durum sizde veya evinizdeki herhangi birinde gerçekleşmişse (Evet / Hayır / Bana uygun değil) ile yanıt verin. Evet ise, lütfen aşağıdaki ölçekte bunun sizde neden olduğu stres miktarını belirtin.

1. Siz veya evinizdeki herhangi biri temel sektör çalışanı mısınız? (örneğin sağlık hizmetleri, gıda sektörü, acil servisler, temel mal / hizmet sağlayıcıları vb.)?

- **Hayır0**
- **Evet** Hiç stresli değil 1
- **Evet** Biraz stres 2
- **Evet** Orta derecede stres 3
- **Evet** Oldukça fazla stres 4
- **Evet** Aşırı derecede stres 5

For peer review only - http://bmjopen.bmj.com/site/about/guidelines.xhtml

2. Covid-19 pandemisi nedeniyle sizin veya evinizdeki herhangi birinin çalışma saatlerinde büyük bir değişiklik oldu mu?

- **Hayır 0**
- **Evet** Hiç stresli değil 1
- **Evet** Biraz stres 2
- **Evet** Orta derecede stres 3
- **Evet** Oldukça fazla stres 4
- **Evet** Aşırı derecede stres 5

3. Covid-19 pandemisi nedeniyle siz veya evinizdeki herhangi biri evden çalışmak zorunda kaldınız mı?

- **Hayır 0**
- **Evet** Hiç stresli değil 1
- **Evet** Biraz stres 2
- **Evet** Orta derecede stres 3
- **Evet** Oldukça fazla stres 4
- **Evet** Aşırı derecede stres 5

For peer review only

For peer review only - http://bmjopen.bmj.com/site/about/guidelines.xhtml

4. Covid-19 pandemisi nedeniyle siz veya evinizdeki herhangi biri farklı bir iş türüne geçmek zorunda kaldınız mı?

- ◯ **Hayır 0**
- ◯ **Evet** Hiç stresli değil 1
- ◯ **Evet** Biraz stres 2
- ◯ **Evet** Orta derecede stres 3
- ◯ **Evet** Oldukça fazla stres 4
- ◯ **Evet** Aşırı derecede stres 5

5. Covid-19 pandemisi nedeniyle siz veya evinizdeki herhangi biri işini kaybetti mi?

- ◯ **Bana uygun değil**9
- ◯ **Hayır 0**
- ◯ **Evet** Hiç stresli değil 1
- ◯ **Evet** Biraz stres 2
- ◯ **Evet** Orta derecede stres 3
- ◯ **Evet** Oldukça fazla stres 4
- ◯ **Evet** Aşırı derecede stres 5

For peer review only

6. Covid-19 pandemisi nedeniyle sizin veya evinizdeki bir başkasının kendi işletmesi veya çalıştığı şirket kapanma tehdidi altında mı?

- ◯ **Bana uygun değil**9
- ◯ **Hayır 0**
- ◯ **Evet** Hiç stresli değil 1
- ◯ **Evet** Biraz stres 2
- ◯ **Evet** Orta derecede stres 3
- ◯ **Evet** Oldukça fazla stres 4
- ◯ **Evet** Aşırı derece stres 5

Gelir

Aşağıdaki ifadeler için, Covid-19 döneminde bu durum evinizdeki herhangi birinde gerçekleştiyse (Evet / Hayır) ile yanıt verin. Evet ise, lütfen aşağıdaki ölçekte bunun sizde neden olduğu stres miktarını belirtin.

1. Covid-19 pandemisi nedeniyle hanenizde büyük bir maddi zorluk yaşadınız mı?

- ◯ **Hayır 0**
- ◯ **Evet** Hiç stresli değil 1
- ◯ **Evet** Biraz stres 2
- ◯ **Evet** Orta derecede stres 3
- ◯ **Evet** Oldukça fazla stres 4
- ◯ **Evet** Aşırı derecede stres 5

For peer review only

For peer review only - http://bmjopen.bmj.com/site/about/guidelines.xhtml

2. Covid-19 pandemisi nedeniyle hanehalkınız faturalarınızı ödeyebilmeniz veya mutfak masraflarınızı karşılayabilmeniz konusunda büyük bir zorluk yaşadı mı?

- ◯ **Hayır 0**
- ◯ **Evet** Hiç stresli değil 1
- ◯ **Evet** Biraz stres 2
- ◯ **Evet** Orta derecede stres 3
- ◯ **Evet** Oldukça fazla stres 4
- ◯ **Evet** Aşırı derecede stres 5

İlişkiler

Aşağıdaki ifadeler için, Covid-19 döneminde sizde böyle bir durum gerçekleştiyse lütfen Evet / Hayır ile yanıt verin. Evet ise, lütfen aşağıdaki ölçekte bunun sizde neden olduğu stres miktarını belirtin. Covid-19'a yanıt olarak....

1. Covid-19 döneminde aile üyeleri arasında zorluklar ve gerginlikler arttı mı?

- ◯ **Hayır 0**
- ◯ **Evet** Hiç stresli değil 1
- ◯ **Evet** Biraz stres 2
- ◯ **Evet** Orta derecede stres 3
- ◯ **Evet** Oldukça fazla stres 4
- ◯ **Evet** Aşırı derecede stres 5

For peer review only

2. Covid-19 pandemisi arkadaşlarınızla (fiziksel veya çevrimiçi/online) iletişiminizi azalttı mı?

- ◯ **Hayır 0**
- ◯ **Evet** Hiç stresli değil 1
- ◯ **Evet** Biraz stres 2
- ◯ **Evet** Orta derecede stres 3
- ◯ **Evet** Oldukça fazla stres 4
- ◯ **Evet** Aşırı derecede stres 5

Günlük rutin ve davranışlarda değişiklikler

Aşağıdaki ifadeler için, Covid-19 döneminde sizde böyle bir durum gerçekleştiyse lütfen (Evet / Hayır / Bana uygun değil) ile yanıt verin. Evet ise, lütfen aşağıdaki ölçekte bunun sizde neden olduğu stres miktarını belirtin. Covid-19'a yanıt olarak......

1. Covid-19 pandemisi günlük rutininizde değişikliklere yol açtı mı? (örneğin, uyku, egzersiz, boş zaman, keyif veren aktiviteler veya hobiler)

- ◯ **Hayır 0**
- ◯ **Evet** Hiç stresli değil 1
- ◯ **Evet** Biraz stres 2
- ◯ **Evet** Orta derecede stres 3
- ◯ **Evet** Oldukça fazla stres 4
- ◯ **Evet** Aşırı derece stres 5

For peer review only - http://bmjopen.bmj.com/site/about/guidelines.xhtml

2. Covid-19 pandemisi sizin veya bir aile üyenizin ihtiyaç duyduğu sağlık hizmetine başvurmayı ertelemenize / başvurmaktan kaçınmanıza neden oldu mu?

- **Hayır 0**
- **Evet** Hiç stresli değil 1
- **Evet** Biraz stres 2
- **Evet** Orta derecede stres 3
- **Evet** Oldukça fazla stres 4
- **Evet** Aşırı derecede stres 5

3. Covid-19 pandemisi dini faaliyetlere katılımınızı azalttı mı? (yüz yüze veya çevrimiçi/online)

- **Bana uygun değil**9
- **Hayır 0**
- **Evet** Hiç stresli değil 1
- **Evet** Biraz stres 2
- **Evet** Orta derecede stres 3
- **Evet** Oldukça fazla stres 4
- **Evet** Aşırı derecede stres 5

For peer review only

4. Covid-19 pandemisi nedeniyle rutininizde başka büyük değişiklikler oldu mu?

◯ **Hayır 0**

◯ Evet ise lütfen belirtin1 ____

Evet ise, lütfen bunun aşağıdaki ölçekte sizde neden olduğu stres miktarını belirtin.

◯ Hiç stresli değil 1

◯ Biraz stres 2

◯ Orta derecede stres 3

◯ Oldukça fazla stres 4

◯ Aşırı derecede stres 5

Covid-19 hakkındaki bilgilere maruz kalma

1. Covid-19 ile ilgili bilgi almak için hangi kaynakları kullanıyorsunuz? (Lütfen uygun olanları işaretleyin.)

☐ Haberler

☐ Facebook / Twitter

☐ Sağlık web siteleri

☐ Duyum/Başkalarından duyma

☐ Diğerleri (lütfen belirtiniz) ____

For peer review only

For peer review only - http://bmjopen.bmj.com/site/about/guidelines.xhtml

2. Covid-19 ile ilgili haberler sizde ne kadar strese neden oluyor?

- ◯ Hiç stresli değil 1
- ◯ Biraz stres 2
- ◯ Orta derecede stres 3
- ◯ Oldukça fazla stres 4
- ◯ Aşırı derecede stres 5

For peer review only

Covid-19'dan bu yana değişiklikler

Aşağıdaki ifadeler için size uygun olanı lütfen ilgili ölçekte belirtiniz. Şimdi ile Covid-19 salgını öncesini karşılaştırma---

1. Covid-19 pandemisi öncesi ile kıyasladığınızda salgın sonrası kişisel stres seviyenizi nasıl değerlendirirsiniz?

- ◯ Önemli ölçüde daha az stresli 1
- ◯ Biraz daha az stresli 2
- ◯ Aynı sayılır 3
- ◯ Daha stresli 4
- ◯ Önemli ölçüde daha fazla stresli 5

2. Covid-19 pandemisi öncesi ile kıyasladığınızda salgın sonrası evinizdeki genel stres düzeyini nasıl değerlendirirsiniz?

- ◯ Önemli ölçüde daha az stresli 1
- ◯ Biraz daha az stresli 2
- ◯ Aynı sayılır 3
- ◯ Daha stresli 4
- ◯ Önemli ölçüde daha fazla stresli 5

For peer review only

3. Covid-19 pandemisi öncesi ile kıyasladığınızda salgın sonrası kişisel psikolojik iyilik halinizi nasıl değerlendirirsiniz?

- ◯ Çok daha iyi 1
- ◯ Daha iyi 2
- ◯ Aynı sayılır 3
- ◯ Daha kötü 4
- ◯ Çok daha kötü 5

4. Covid-19 pandemisi öncesi ile kıyasladığınızda salgın sonrası hanehalkı üyelerinizin genel psikolojik iyilik halini nasıl değerlendirirsiniz?

- ◯ Çok daha iyi 1
- ◯ Daha iyi 2
- ◯ Ayni sayilir 3
- ◯ Daha kötü 4
- ◯ Çok daha kötü 5

For peer review only

K-10

Aşağıdaki 10 soru son 4 hafta içinde kendinizi nasıl hissettiğinizle ilgilidir. Her soru için, kendinizi o durumda hissettiğiniz zamanın uzunluğunu en iyi şekilde tanımlayan seçeneği seçiniz.

	**Hiçbir zaman 1**	**Kısa bir süre 2**	**Bazen 3**	**Coğu zaman 4**	**Her zaman 5**
1. Son 4 hafta içinde, ne kadar sıklıkla kendinizi sebepsiz yere yorgun hissettiniz?	○	○	○	○	○
2. Son 4 hafta içinde, ne kadar sıklıkla kendinizi gergin hissettiniz?	○	○	○	○	○
3. Son 4 hafta icinde ne kadar sıklıkla sizi hiçbir şeyin sakinleştirmeyeceği kadar gergin hissettiniz?	○	○	○	○	○
4. Son 4 hafta içinde ne kadar sıklıkla kendinizi umutsuz hissettiniz?	○	○	○	○	○
5. Son 4 hafta içinde ne kadar sıklıkla kendinizi tedirgin veya huzursuz hissettiniz?	○	○	○	○	○
6. Son 4 hafta içinde ne kadar sıklıkla yerinde duramayacak kadar huzursuz hissettiniz?	○	○	○	○	○
7. Son 4 hafta içinde ne kadar sıklıkla kendinizi bunalımlı hissettiniz?	○	○	○	○	○
8. Son 4 hafta içinde ne kadar sıklıkla en ufak bir şeyin bile çok çaba gerektirdiğini hissettiniz?	○	○	○	○	○

For peer review only

For peer review only - http://bmjopen.bmj.com/site/about/guidelines.xhtml

9. Son 4 hafta içinde ne kadar sıklıkla kendinizi sizi hiçbir şeyin neşelendiremeyeceği kadar üzgün hissettiniz?	○	○	○	○	○
10. Son 4 hafta icinde ne kadar sıklıkla kendinizi değersiz hissettiniz?	○	○	○	○	○

For peer review only

For peer review only - http://bmjopen.bmj.com/site/about/guidelines.xhtml

PCL-5

Aşağıda, bazı kişilerin Covid-19'a tepki olarak yaşadığı sorunların bir listesi bulunmaktadır. Lütfen her bir sorunu dikkatlice okuyun ve geçen ay bu sorundan ne kadar rahatsız olduğunuzu belirtin. Geçen ay içinde aşağıda yer alan durumlar sizi ne ölçüde bunalttı:

	**Hiç 0**	**Çok az 1**	**Orta derecede 2**	**Oldukça az 3**	**Aşırı 4**
1. Stresli olayın tekrarlayan, rahatsız eden ve istenmeyen anları sizi ne kadar bunalttı?	○	○	○	○	○
2. Stresli olaya ilişkin tekrarlayan, rahatsız eden rüyalar sizi ne kadar bunalttı?	○	○	○	○	○
3. Aniden stresli olayı sanki gerçekten bir daha yaşıyormuş gibi hissetmek veya davranmak (sanki gerçekten olayın yaşandığı ana geri dönmüş yeniden yaşıyor gibi) sizi ne kadar bunalttı?	○	○	○	○	○
4. Bir şeyler size stresli olayı anımsattığı zaman yaşadığınız üzüntü hissi sizi ne kadar bunalttı?	○	○	○	○	○
5. Bir şeyler size stresli olayı anımsattığı zaman güçlü fiziksel tepkiler vermek (örneğin, kalp çarpıntısı, nefes almada güçlük, terleme gibi) sizi ne kadar bunalttı?	○	○	○	○	○
6. Stresli olayla ilişkili anılardan,düşüncelerden ve duygulardan kaçınmaya çalışmak sizi ne kadar bunalttı?	○	○	○	○	○
7. Stresli olayı anımsatan etraftaki hatırlatıcı şeylerden (örneğin, insanlardan, yerlerden, konuşmalardan, etkinliklerden, nesnelerden veya durumlardan)	○	○	○	○	○

For peer review only

kaçınmaya çalışmak sizi ne kadar bunalttı?

8. Stresli olaya ilişkin önemli kısımları hatırlamada yaşanan güçlükler sizi ne kadar bunalttı?

9. Kendiniz, diğer insanlar veya dünya hakkında güçlü olumsuz düşüncelere sahip olmak (örneğin, kötü biriyim, bende ciddi şekilde yanlış olan bir şeyler var, kimseye güvenilmez, dünya tümüyle tehlikeli bir yerdir gibi düşünceler) sizi ne kadar bunalttı?

10. Stresli olay veya bu olayın sonrasında ortaya çıkan durumlar için kendinizi veya bir başkasını suçlamak sizi ne kadar bunalttı?

11. Korku, dehşete kapılma, öfke, suçluluk, veya utanç gibi olumsuz duygular sizi ne kadar bunalttı?

12. Daha önce yapmaktan keyif aldığınız etkinliklere olan ilginizi kaybetmek sizi ne kadar bunalttı?

13. Başka insanlardan uzak veya kopmuş hissetmek sizi ne kadar bunalttı?

14. Olumlu duyguları yaşamak (örneğin, mutluluğu hissetmek vaya size yakın insanlara sevgi dolu hisler duyamamak) sizi ne kadar bunalttı?

15. Asabi davranışlar, öfke patlamaları veya öfkeli hareketler sizi ne kadar bunalttı?

For peer review only

For peer review only - http://bmjopen.bmj.com/site/about/guidelines.xhtml

16. Çok fazla risk almak veya size zarar verebilecek şeyler yapmak sizi ne kadar bunaltı?	○	○	○	○	○
17. Aşırı tetikte olmak veya temkinli davranmak veya hazırda beklemek sizi ne kadar bunalttı?	○	○	○	○	○
18. Yerinden sıçramak veya kolayca irkilmek sizi ne kadar bunalttı?	○	○	○	○	○
19. Dikkati toplamada güçlükler sizi ne kadar bunalttı?	○	○	○	○	○
20. Uykuya dalma veya uykuyu devam ettirme güçlükleri sizi ne kadar bunalttı?	○	○	○	○	○

WHO-5

Aşağıdaki beş tanımlamadan her biri için, son iki hafta süresince kendinizi nasıl hissettiğinize en yakın olan yanıtı veriniz. Son iki hafta boyunca

	**Her zaman 5**	**Çoğu zaman 4**	**Zamanın yarısından çoğunda 3**	**Zamanın yarısından azında 2**	**Bazen 1**	**Hiçbir zaman 0**
1. Kendimi neşeli ve keyifli hissettim	○	○	○	○	○	○
2. Kendimi sakin ve rahatlamış hissettim.	○	○	○	○	○	○
3. Kendimi aktif ve dinç hissettim	○	○	○	○	○	○
4. Sabahları kendimi taze ve dinlenmiş hissederek uyandım	○	○	○	○	○	○
5. Günlük yaşantım beni ilgilendiren şeylerle dolu	○	○	○	○	○	○

For peer review only

For peer review only - http://bmjopen.bmj.com/site/about/guidelines.xhtml

PTGI

COVID-19 sonucunda yaşamınızda ve düşüncelerinizde meydana gelen değişimleri lütfen aşağıda verilen puanlama ölçütlerine göre 0 ve 5 arasında değerlendiriniz.

	Hiç 0	Çok az 1	Az derecede 2	Orta derecede 3	Oldukça fazla 4	Aşırı derecede 5
1. Yaşamda önem verdiğim şeylerin öncelik sırası değişti.	○	○	○	○	○	○
2. Kendi hayatıma verdiğim değerde büyük bir artış oldu.	○	○	○	○	○	○
3. Yeni ilgi alanları keşfettim.	○	○	○	○	○	○
4. Kendime güven hissinde artış oldu.	○	○	○	○	○	○
5. Manevi konuları daha iyi anlamaya başladım.	○	○	○	○	○	○
6. Başım sıkıştığında insanlara güvenebileceğimi daha iyi anladım.	○	○	○	○	○	○
7. Yaşamım için yeni bir yön belirledim.	○	○	○	○	○	○
8. Kendimi diğer insanlarla çok daha yakın hissetmeye başladım.	○	○	○	○	○	○
9. Duygularımı ifade etmeye daha çok istekliyim.	○	○	○	○	○	○
10. Zorlukları göğüsleyebileceğimi daha iyi anladım.	○	○	○	○	○	○
11. Yaşamımda daha iyi şeyler yapabiliyorum.	○	○	○	○	○	○
12. Her şeyi olduğu gibi, daha çok kabullenebiliyorum.	○	○	○	○	○	○

For peer review only

For peer review only - http://bmjopen.bmj.com/site/about/guidelines.xhtml

13. Her günümü daha iyi değerlendirebiliyorum.

14. Daha önce var olmayan yeni olanaklara kavuştum.

15. Diğer insanlara karşı daha şefkatliyim.

16. İlişkilerime daha çok emek sarf etmeye başladım.

17. Değişmesi gereken şeyleri değiştirebilmek için daha çok çaba harcıyorum.

18. Daha güçlü bir inanca sahibim.

19. Düşündüğümden çok daha güçlü olduğumu keşfettim.

20. İnsanların ne kadar mükemmel olabildiklerine dair çok şey öğrendim.

21. Başkalarına ihtiyaç duyuyor olmayı daha çok kabullendim.

Helpline Information

Sağlık Bakanlığına ait psikososyal destek hatları ilden ile değişiklik göstermektedir.

Gaziantep İl Sağlık Müdürlüğü: 03422200505/Dahili:1142-1145 / 0342 517 52 58/Dahili:113
Bulunduğunuz ilin psikososyal destek hatlarına Sağlık Bakanlığı’na ait aşağıdaki linkten ulaşabilirsiniz.
https://covid19.saglik.gov.tr/TR-66158/81-il-psikososyal-destek-hat-bilgileri.html

For peer review only

For peer review only - http://bmjopen.bmj.com/site/about/guidelines.xhtml

International Study - Pakistan

For peer review only

For peer review only - http://bmjopen.bmj.com/site/about/guidelines.xhtml

Information Sheet

کوویڈ 19-وبا کے نفسیاتی اثرات کا بین الاقوامی جائزہ

اس سروے میں آپ کی دلچسپی کا شکریہ۔ شرکت کرنے یا نہ کرنے کا فیصلہ کرنے سے پہلے براہ مہربانی اس معلوماتی شیٹ کو غور سے پڑھیں۔ اگر آپ اس سروے میں حصہ لینے کا فیصلہ کرتے ہیں تو ہم آپ کا شکریہ ادا کرتے ہیں۔ اگر آپ حصہ نہ لینے کا فیصلہ کرتے ہیں تو اس سے آپ کو کوئی نقصان نہیں ہوگا اور ہماری درخواست پر غور کرنے کے لیے ہم آپ کے مشکور ہیں۔

اس سروے کا مقصد کیا ہے؟

اس سروے کا مقصد کوویڈ 19-وبا کے نفسیاتی اثرات کا جائزہ لینا ہے۔

اس سروے میں کون حصہ لے سکتا ہے؟

کوئی بھی شخص جس کی عمر 18 سال یا اس سے زیادہ ہے اس سروے میں حصہ لینے کی اہل ہے۔

شرکاء سے کیا کرنے کو کہا جائے گا؟

اگر آپ اس سروے میں حصہ لینے پر راضی ہیں تو آپ سے 15-20 منٹ کا ایک مختصر سروے مکمل کرنے کو کہا جائے گا۔

کون سی معلومات اکٹھی کی جائیں گی اور ان کا کیا استعمال کیا جائے گا؟

ہم آپ کے بارے میں بنیادی معلومات اور آپ کے سوالناموں کے جوابات جمع کریں گے۔ حاصل کردہ معلومات تک صرف تحقیقاتی ٹیم (ڈیپارٹمنٹ آف سائیکالوجیکل میڈیسن، یونیورسٹی آف اوٹاگو، کرائسٹ چرچ نیوزی لینڈ) کو رسائی حاصل ہوگی۔

تحقیق کے نتیجے میں حاصل کردہ ڈیٹا کو کم از کم 5 سال تک محفوظ رکھا جائے گا۔ اس مطالعہ میں ایسی کوئی معلومات اکٹھی نہیں کی جائے گی جو آپ کی شناخت کا سبب بنے۔ اس تحقیق کے نتائج شائع کیے جاسکتے ہیں اور وہ یونیورسٹی آف اوٹاگو کی لائبریری(ڈنیڈن، نیوزی لینڈ) میں دستیاب ہوں گے۔

کیا شرکاء اپنا ارادہ بدل سکتے ہیں اور اس سروے سے دستبردار ہو سکتے ہیں؟

آپ اس سروے کی تکمیل سے پہلے بغیر کسی نقصان کے دستبردار ہو سکتے ہیں۔

کیا آپ کے کوویڈ۔ 19کے بارے میں سوالات ہیں؟

کوویڈ سے متعلق معلومات کے لیے، براہ کرم /https://covid.gov.pk پر جائیں۔

ہیلپ لائن کے لیے 1166 ڈائل کریں۔

شرکاء کے کسی بھی سوالات کےلئے، مندرجہ ذیل افراد سے رابطہ کریں؟

ڈاکٹر کیرولین بیل۔
شعبہ نفسیاتی طب۔
ای میل ایڈریس caroline.bell@otago.ac.nz

ڈاکٹر صندیلہ تنویر
شعبہ نفسیاتی طب۔
ای میل ایڈریس sandila.tanveer@otago.ac.nz

For peer review only - http://bmjopen.bmj.com/site/about/guidelines.xhtml

Consent Form

اگر آپ اس سروے میں حصہ لینے پر راضی ہیں تو، براہ مہربانی درج ذیل بیانات کے لئے اپنی رضامندی فراہم کریں۔

میں جانتا/جانتی ہوں کہ سروے کے بارے میں سوالات کی صورت میں مجھے کس سے رابطہ کرنا ہے۔
میں جانتا/جانتی ہوں کہ میرا سروے میں حصہ لینا رضاکارانہ (میری مرضی) ہے اور یہ کہ میں کسی بھی وقت اس سروے سے بغیر کسی جرمانہ (penalty) کے دستبردار ہوسکتا/ہوسکتی ہوں۔
سروے کے دوران گھبراہٹ یا پریشانی کی صورت میں، مجھے سپورٹ (support) اور امداد کے بارے میں ضروری معلومات فراہم کی گئی ہیں۔
میں جانتا/جانتی ہوں کہ اس سروے کے ڈیٹا کو کم از کم پانچ سال تک محفوظ رکھا جائے گا۔
میں جانتا/جانتی ہوں کہ اس سروے کے نتائج شائع ہوسکتے ہیں اور یہ نتائج یونیورسٹی آف اوٹاگو (ڈینیڈن، نیوزی لینڈ) کی لائبریری میں دستیاب ہوں گے۔
میں جانتا/جانتی ہوں کہ اس سروے میں میری شرکت خفیہ ہے اور کوئی بھی ایسا مواد جس سے میری شناخت ممکن ہو، نہ تو اسے حاصل کیا جائے گا اور نہ ہی اسے سروے کی کسی رپورٹ میں شائع کیا جائے گا۔

○ **میں سروے میں حصہ لینے پر راضی ہوں۔**

○ **میں سروے میں حصہ لینے پر راضی نہیں ہوں۔**

For peer review only

For peer review only - http://bmjopen.bmj.com/site/about/guidelines.xhtml

Sociodemographic Information

1. جنس

- ○ مرد
- ○ عورت
- ○ دیگر
- ○ جواب نہیں دینا چاہتے

2. عمر (سالوں میں)

____

3. ازدواجی حیثیت

- ○ غیر شادی شدہ
- ○ غیر ازدوجی تعلق
- ○ شادی شدہ
- ○ علیحدگی
- ○ طلاق یافتہ
- ○ بیوہ یا رنڈوا

For peer review only

4. آپ کا دین کیا ہے؟

○ کوئی دین نہیں

○ اسلام

○ عیسائیت

○ ہندومت

○ بدھ مت

○ دیگر (براہ مہربانی وضاحت کریں) ________________________________

5. آپ کا نسلی پس منظر کیا ہے؟ (آپ ایک سے زیادہ درج کرسکتے ہیں)

____

6 .آپ روزمرہ کی زندگی میں کون سی زبانیں روانی سے بولتے ہیں؟

____

درج ذیل دو سوالات آپ کی انگریزی زبان کی اہلیت کے بارے میں ہیں۔

6a. میں انگریزی اچھی طرح سمجھ سکتا/سکتی ہوں؟*

○ بالکل بھی نہیں1

○ تھوڑی سی2

○ درمیانی حد تک3

○ زیادہ4

○ بہت زیادہ5

For peer review only - http://bmjopen.bmj.com/site/about/guidelines.xhtml

.6b میں انگریزی اچھی طرح بول سکتا/سکتی ہوں؟

- بالکل بھی نہیں1
- تھوڑی سی 2
- درمیانی حد تک3
- زیادہ4
- بہت زیادہ5

.7 آپ کی تعلیم کی اعلی ترین سطح کیا ہے؟

- کوئی باضابطہ تعلیم نہیں
- ہائیر سیکنڈری سکول (جیسے ڈپلومہ یا A-level)
- سرٹیفکیٹ یا ڈپلومہ 3 - سال سے کم کورس
- بیچلر ڈگری (3-4 سالہ کورس)
- پوسٹ گریجویٹ ڈگری (پوسٹ گریجویٹ ڈپلومہ، ماسٹر، ڈاکٹریٹ)

For peer review only

7. موجودہ اسٹڈی یا کام کی حیثیت (براہ مہربانی جو بھی لاگو ہوں ان پر نشان لگائیں)

- ▫ فل ٹائم طالب علم
- ▫ پارٹ ٹائم طالب علم
- ▫ بے روزگار/کام کی تلاش
- ▫ فل ٹائم ملازمت
- ▫ پارٹ ٹائم ملازمت
- ▫ فل ٹائم ذاتی کام
- ▫ پارٹ ٹائم ذاتی کام
- ▫ دفتری پیڈ پیرینٹل چھٹی (paid parental leave)
- ▫ ہاؤس وائف یا سٹیے-ایٹ-ہوم والد (stay-at-home dad) بلا معاوضہ
- ▫ ریٹائرڈ
- ▫ دیگر (براہ مہربانی وضاحت کریں) ______________________________

8. پیشہ

____

9. ماہانہ گھریلو آمدنی (مقامی کرنسی میں)

____

10. اوسط آمدن کے مقابلے میں، آپ اپنے گھر کی آمدنی کی درجہ بندی کیسے کریں گے؟

- ○ اوسط سے کم
- ○ اوسط
- ○ اوسط سے زیادہ

10. کیا آپ کوویڈ 19-کی وجہ سے لاک ڈاؤن میں ہیں؟

- ○ ہاں
- ○ نہیں

For peer review only - http://bmjopen.bmj.com/site/about/guidelines.xhtml

11. کیا آپ کو کوویڈ 19-کی ویکسین دی گئی ہے؟

ہاں

نہیں

11a. اگر نہیں، تو کیا آپ مستقبل میں کوویڈ 19-کی ویکسین لینا چاہیں گیں؟

ہاں

نہیں

12. ہم سمجھتے ہیں کہ کچھ لوگوں کو ماضی میں تکلیف دہ واقعات کا سامنا کرنا پڑا ہوا ہو گا جس کی وجہ سے نئی پریشانیوں سے نمٹنے میں آسانی یا دقت پیدا ہو سکتی ہے۔

کوویڈ-19کی وبا سے پہلے، کیا آپ نے کبھی مندرجہ ذیل میں سے کسی واقعہ کا مشاہدہ یا تجربہ کیا ہے: (براہ مہربانی جو بھی لاگو ہوں ان پر نشان لگائیں)

- قدرتی آفات (جیسے سیلاب، زلزلہ، وغیرہ)
- جنگ یا فوجی تنازعات
- 16 سال کی عمر سے پہلے، بچپن کی پریشانیاں (جیسے نظر انداز ہونا (neglect) ،غنڈہ گردی کا شکار (bullying) جسمانی یا جنسی تشدد)
- 16 سال کی عمر کے بعد جسمانی یا جنسی تشدد
- سنگین حادثہ / ایکسیڈنٹ
- دیگر – وضاحت کریں (وضاحت کرنا لازمی نہیں)____________________________________
- مندرجہ بالا میں سے کوئی نہیں

For peer review only

Covid Psychosocial Impacts Scale

ذاتی اثرات

مندرجہ ذیل بیانات کوویڈ-19 کے آپ پر ہونے والے اثرات کے بارے میں ہیں، براہ مہربانی درج ذیل بیانات کے لئے" ہاں / نہیں "میں سے کسی ایک کا انتخاب کریں۔ اگر آپ کا جواب ہاں میں ہے تو، براہ مہربانی نیچے دیے گیے اسکیل پر اس کی وجہ سے ہونے والے دباؤ (stress) کی نشاندہی کریں۔

1. کیا آپ کو کوئی ایسی بیماری لاحق ہے جو آپ کو کوویڈ-19 کی زد میں لا سکتی ہے؟

- ○ نہیں0
- ○ **ہاں** کوئی دباؤ نہیں1
- ○ **ہاں** تھوڑا سا دباؤ 2
- ○ **ہاں** درمیانی حد تک دباؤ 3
- ○ **ہاں** زیادہ دباؤ 4
- ○ **ہاں** بہت زیادہ دباؤ 5

2. کیا آپ کو کسی شخص سے کوویڈ-19 کی زد میں آنے کا خطرہ لاحق ہوا ہے؟

- ○ نہیں0
- ○ **ہاں** کوئی دباؤ نہیں1
- ○ **ہاں** تھوڑا سا دباؤ 2
- ○ **ہاں** درمیانی حد تک دباؤ 3
- ○ **ہاں** زیادہ دباؤ 4
- ○ **ہاں** بہت زیادہ دباؤ 5

For peer review only

.3 کیا آپ کو لگتا ہے کہ آپ کو کوویڈ-19 ہوا تھا؟

- نہیں0
- **ہاں** کوئی دباؤ نہیں1
- **ہاں** تھوڑا سا دباؤ2
- **ہاں** درمیانی حد تک دباؤ3
- **ہاں** زیادہ دباؤ4
- **ہاں** بہت زیادہ دباؤ5

.3a کیا آپ کا کوویڈ-19 کا ٹیسٹ مثبت آیا تھا؟

- نہیں0
- **ہاں** کوئی دباؤ نہیں1
- **ہاں** تھوڑا سا دباؤ2
- **ہاں** درمیانی حد تک دباؤ3
- **ہاں** زیادہ دباؤ4
- **ہاں** بہت زیادہ دباؤ5

For peer review only

For peer review only - http://bmjopen.bmj.com/site/about/guidelines.xhtml

3b. کیا آپ کو آج کل کوویڈ-19 کی علامات ہیں؟

- نہیں0
- ہاں کوئی دباؤ نہیں1
- ہاں تھوڑا سا دباؤ2
- ہاں درمیانی حد تک دباؤ3
- ہاں زیادہ دباؤ4
- ہاں بہت زیادہ دباؤ5

خاندان والوں پر اثرات

مندرجہ ذیل بیانات کوویڈ-19 کے آپ کے قریبی خاندان والوں پر ہونے والے اثرات کے بارے میں ہیں، براہ مہربانی درج ذیل بیانات کے لئے "ہاں / نہیں "میں سے کسی ایک کا انتخاب کریں۔ اگر آپ کا جواب ہاں میں ہے تو، براہ مہربانی نیچے دیے گیے اسکیل پر اس کی وجہ سے ہونے والے دباؤ (stress) کی نشاندہی کریں۔

1. کیا آپ کے خاندان کے کسی قریبی فرد کو کوئی ایسی بیماری ہے جو انہیں کوویڈ-19 کی زد میں لا سکتی ہے؟

- نہیں0
- ہاں کوئی دباؤ نہیں1
- ہاں تھوڑا سا دباؤ2
- ہاں درمیانی حد تک دباؤ3
- ہاں زیادہ دباؤ4
- ہاں بہت زیادہ دباؤ5

For peer review only - http://bmjopen.bmj.com/site/about/guidelines.xhtml

.2 کیا آپ کو خدشہ ہے کہ آپ کے خاندان کے کسی قریبی فرد کو کسی شخص سے کوویڈ-19کی زد میں آنے کا خطرہ لاحق ہوا ہے؟

- نہیں0
- ہاں کوئی دباؤ نہیں1
- ہاں تھوڑا سا دباؤ2
- ہاں درمیانی حد تک دباؤ3
- ہاں زیادہ دباؤ4
- ہاں بہت زیادہ دباؤ5

.3 کیا آپ کو لگتا ہے کہ خاندان کے کسی قریبی فرد کو کوویڈ-19 ہوا تھا؟

- نہیں0
- ہاں کوئی دباؤ نہیں1
- ہاں تھوڑا سا دباؤ2
- ہاں درمیانی حد تک دباؤ3
- ہاں زیادہ دباؤ4
- ہاں بہت زیادہ دباؤ5

For peer review only

For peer review only - http://bmjopen.bmj.com/site/about/guidelines.xhtml

3a. کیا خاندان کے کسی قریبی فرد کا کوویڈ-19 کا ٹیسٹ مثبت آیا تھا؟

- نہیں0
- ہاں کوئی دباؤ نہیں1
- ہاں تھوڑا سا دباؤ2
- ہاں درمیانی حد تک دباؤ3
- ہاں زیادہ دباؤ4
- ہاں بہت زیادہ دباؤ5

3b. کیا خاندان کے کسی قریبی فرد کو آج کل کوویڈ-19 کی علامات ہیں؟

- نہیں0
- ہاں کوئی دباؤ نہیں1
- ہاں تھوڑا سا دباؤ2
- ہاں درمیانی حد تک دباؤ3
- ہاں زیادہ دباؤ4
- ہاں بہت زیادہ دباؤ5

For peer review only

.3c کیا خاندان کے کسی قریبی فرد کا کوویڈ-19 سے انتقال ہوا ہے؟

- نہیں0
- ہاں کوئی دباؤ نہیں1
- ہاں تھوڑا سا دباؤ2
- ہاں درمیانی حد تک دباؤ3
- ہاں زیادہ دباؤ4
- ہاں بہت زیادہ دباؤ5

.4 کیا آپ کو فکر لاحق ہے کہ بیرون ملک مقیم خاندان کے کسی قریبی فرد کو کوویڈ-19 کی زد میں آنے کا امکان ہے؟

- نہیں0
- ہاں کوئی دباؤ نہیں1
- ہاں تھوڑا سا دباؤ2
- ہاں درمیانی حد تک دباؤ3
- ہاں زیادہ دباؤ4
- ہاں بہت زیادہ دباؤ5

For peer review only

For peer review only - http://bmjopen.bmj.com/site/about/guidelines.xhtml

.5 کیا بیرون ملک مقیم خاندان کے کسی قریبی فرد کو آپ کوویڈ-19 کی وجہ سے ملنے نہیں جا سکتے یا وہ آپ کوویڈ-19 کی وجہ سے ملنے نہیں آ سکتے ہیں؟

- نہیں0
- ہاں کوئی دباؤ نہیں1
- ہاں تھوڑا سا دباؤ2
- ہاں درمیانی حد تک دباؤ3
- ہاں زیادہ دباؤ4
- ہاں بہت زیادہ دباؤ5

کام

مندرجہ ذیل بیانات کوویڈ-19 کے آپ اور آپ کے گھرانے پر ہونے والے اثرات کے بارے میں ہیں، براہ مہربانی درج ذیل بیانات کے لئے" ہاں / نہیں / قابل اطلاق نہیں "میں سے کسی ایک کا انتخاب کریں۔ اگر آپ کا جواب ہاں میں ہے تو، براہ مہربانی نیچے دیے گیے اسکیل پر اس کی وجہ سے ہونے والے دباؤ (stress) کی نشاندہی کریں۔

.1 کیا آپ یا آپ کے گھر کا کوئی فرد ایک ضروری ورکر کے طور پر شمار کیا گیا ہے (مثلاً ہیلتھ کیئر، قانون نافذ کرنے والے ادارے، ایمرجنسی سروسز، اسینشل/ضروری سامان/خدمات فراہم کرنے والے اداروں میں)؟

- نہیں0
- ہاں کوئی دباؤ نہیں1
- ہاں تھوڑا سا دباؤ2
- ہاں درمیانی حد تک دباؤ3
- ہاں زیادہ دباؤ4
- ہاں بہت زیادہ دباؤ5

For peer review only - http://bmjopen.bmj.com/site/about/guidelines.xhtml

.2 کیا آپ کے یا آپ کے گھرانے کے کسی فرد کے کام کرنے کے اوقات میں ایک بڑی تبدیلی رونما ہوئی ہے؟

- نہیں0
- ہاں کوئی دباؤ نہیں1
- ہاں تھوڑا سا دباؤ2
- ہاں درمیانی حد تک دباؤ3
- ہاں زیادہ دباؤ4
- ہاں بہت زیادہ دباؤ5

.3 کیا آپ کو یا آپ کے گھرانے کے کسی فرد کو گھر سے کام کرنا پڑرہا ہے؟

- نہیں0
- ہاں کوئی دباؤ نہیں1
- ہاں تھوڑا سا دباؤ2
- ہاں درمیانی حد تک دباؤ3
- ہاں زیادہ دباؤ4
- ہاں بہت زیادہ دباؤ5

For peer review only

.4 کیا آپ کے یا آپ کے گھرانے کے کسی فرد کے کام (job-related work) کی نوعیت بَدَل گئی ہے؟

- نہیں0
- ہاں کوئی دباؤ نہیں1
- ہاں تھوڑا سا دباؤ2
- ہاں درمیانی حد تک دباؤ3
- ہاں زیادہ دباؤ4
- ہاں بہت زیادہ دباؤ5

.5 کیا آپ کی یا آپ کے گھرانے کے کسی فرد کی ملازمت چھوٹ گئی ہے؟

- قابل اطلاق نہیں
- نہیں0
- ہاں کوئی دباؤ نہیں1
- ہاں تھوڑا سا دباؤ2
- ہاں درمیانی حد تک دباؤ3
- ہاں زیادہ دباؤ4
- ہاں بہت زیادہ دباؤ5

For peer review only

.6 کیا ایسا کاروبار جو آپ کی یا آپ کے گھرانے کی ملکیت ہے، یا جس میں آپ یا آپ کے گھر والے کام کرتے ہیں، بقا کے خطرے میں ہے؟

- قابل اطلاق نہیں
- نہیں0
- ہاں کوئی دباؤ نہیں1
- ہاں تھوڑا سا دباؤ2
- ہاں درمیانی حد تک دباؤ3
- ہاں زیادہ دباؤ4
- ہاں بہت زیادہ دباؤ5

آمدن

مندرجہ ذیل بیانات کوویڈ-19 کے آپ کے گھرانے پر ہونے والے اثرات کے بارے میں ہیں، براہ مہربانی درج ذیل بیانات کے لئے" ہاں / نہیں " میں سے کسی ایک کا انتخاب کریں۔ اگر آپ کا جواب ہاں میں ہے تو، براہ مہربانی نیچے دیے گیے اسکیل پر اس کی وجہ سے ہونے والے دباؤ (stress)کی نشاندہی کریں۔

.1 کیا آپ کے گھرانے کے مالی حالات میں واضح کمی واقع ہوئی ہے؟

- نہیں0
- ہاں کوئی دباؤ نہیں1
- ہاں تھوڑا سا دباؤ2
- ہاں درمیانی حد تک دباؤ3
- ہاں زیادہ دباؤ4
- ہاں بہت زیادہ دباؤ5

For peer review only

For peer review only - http://bmjopen.bmj.com/site/about/guidelines.xhtml

2. کیا آپ کے گھرانے کی بلوں کی ادائیگی یا کھانے پینے کے اخراجات کرنے کی صلاحیت میں واضح کمی واقع ہوئی ہے؟

- نہیں0
- ہاں کوئی دباؤ نہیں1
- ہاں تھوڑا سا دباؤ2
- ہاں درمیانی حد تک دباؤ3
- ہاں زیادہ دباؤ4
- ہاں بہت زیادہ دباؤ5

تعلقات

مندرجہ ذیل بیانات کوویڈ-19 کے آپ پر ہونے والے اثرات کے بارے میں ہیں، براہ مہربانی درج ذیل بیانات کے لئے" ہاں / نہیں "میں سے کسی ایک کا انتخاب کریں۔ اگر آپ کا جواب ہاں میں ہے تو، براہ مہربانی نیچے دیے گیے اسکیل پر اس کی وجہ سے ہونے والے دباؤ (stress) کی نشاندہی کریں۔

1. کیا کوویڈ-19 کے نتیجے میں، فیملی کی مشکلات اور باہمی ناچاقی میں اضافہ ہوا ہے؟

- نہیں0
- ہاں کوئی دباؤ نہیں1
- ہاں تھوڑا سا دباؤ2
- ہاں درمیانی حد تک دباؤ3
- ہاں زیادہ دباؤ4
- ہاں بہت زیادہ دباؤ5

For peer review only - http://bmjopen.bmj.com/site/about/guidelines.xhtml

.2 کیا کوویڈ-19 کے نتیجے میں، دوستوں کے ساتھ آپ کے رابطے (روبرو یا آن لائن) میں کمی واقع ہوئی ہے؟

- نہیں0
- ہاں کوئی دباؤ نہیں1
- ہاں تھوڑا سا دباؤ2
- ہاں درمیانی حد تک دباؤ3
- ہاں زیادہ دباؤ4
- ہاں بہت زیادہ دباؤ5

روزمرہ کے معمولات اور طرزعمل میں تبدیلیاں

مندرجہ ذیل بیانات کوویڈ-19 کے آپ پر ہونے والے اثرات کے بارے میں ہیں، براہ مہربانی درج ذیل بیانات کے لئے " ہاں / نہیں" میں سے کسی ایک کا انتخاب کریں۔ اگر آپ کا جواب ہاں میں ہے تو، براہ مہربانی نیچے دیے گیے اسکیل پر اس کی وجہ سے ہونے والے دباؤ (stress) کی نشاندہی کریں۔

.1 کیا کوویڈ-19 کی وجہ سے آپ کے روزمرہ معمولات (مثال کے طور پر، نیند، ورزش، فارغ وقت، خوشگوار سرگرمیوں، یا مشاغل) میں تبدیلی واقع ہوئی ہے؟

- نہیں0
- ہاں کوئی دباؤ نہیں1
- ہاں تھوڑا سا دباؤ2
- ہاں درمیانی حد تک دباؤ3
- ہاں زیادہ دباؤ4
- ہاں بہت زیادہ دباؤ5

For peer review only - http://bmjopen.bmj.com/site/about/guidelines.xhtml

.2 کیا کوویڈ-19 کی وجہ سے آپ نے اپنے یا اپنے گھر والوں کی ہیلتھ کیئر (healthcare) کے حصول میں تاخیر کی ہے؟

- نہیں0
- ہاں کوئی دباؤ نہیں1
- ہاں تھوڑا سا دباؤ2
- ہاں درمیانی حد تک دباؤ3
- ہاں زیادہ دباؤ4
- ہاں بہت زیادہ دباؤ5

.3 کیا کوویڈ-19 کی وجہ سے آپ کی دینی سرگرمیوں(روبرو یا آن لائن) میں شمولیت میں کمی واقع ہوئی ہے؟

- قابل اطلاق نہیں
- نہیں0
- ہاں کوئی دباؤ نہیں1
- ہاں تھوڑا سا دباؤ2
- ہاں درمیانی حد تک دباؤ3
- ہاں زیادہ دباؤ4
- ہاں بہت زیادہ دباؤ5

.4 کیا کوویڈ-19 کی وجہ سے معمول میں کوئی اور بڑی تبدیلی واقع ہوئی ہے؟

- نہیں0
- ہاں (براہ مہربانی وضاحت کریں۔) 1 ______________________________

For peer review only

For peer review only - http://bmjopen.bmj.com/site/about/guidelines.xhtml

اگر آپ کا جواب ہاں میں ہے تو، براہ مہربانی نیچے دیے گیے اسکیل پر اس کی وجہ سے ہونے والے دباؤ (stress) کی نشاندہی کریں.

- ◯ کوئی دباؤ نہیں1
- ◯ تھوڑا سا دباؤ2
- ◯ درمیانی حد تک دباؤ3
- ◯ زیادہ دباؤ4
- ◯ بہت زیادہ دباؤ5

کوویڈ-19 کے بارے میں معلومات

1. کوویڈ-19 کے حوالے سے، آپ کے لئے معلومات کا ذریعہ کیا ہے؟ (براہ مہربانی جو بھی آپ پر لاگو ہوں انہیں منتخب کریں.)

- ▫ خبریں
- ▫ فیس بک / ٹویٹر
- ▫ ہیلتھ ویب سائٹس
- ▫ لوگوں کی باتیں
- ▫ دیگر(براہ مہربانی وضاحت کریں) ______________________________

2. کوویڈ-19 سے متعلق خبریں آپ کے لیے کس قدر دباؤ (stress) کا سبب ہیں؟

- ◯ کوئی دباؤ نہیں1
- ◯ تھوڑا سا دباؤ2
- ◯ درمیانی حد تک دباؤ3
- ◯ زیادہ دباؤ4
- ◯ بہت زیادہ دباؤ5

For peer review only - http://bmjopen.bmj.com/site/about/guidelines.xhtml

For peer review only

کوویڈ- 19کے بعد ہونے والی تبدیلیاں

براہ کرم کوویڈ-19 کی عالمی وبا کا پہلے سے موازنہ کرتے ہوئے، درج ذیل بیانات پر دیئے گئے پیمانے کی مدد سے نشاندہی کریں۔

1. آپ اپنے ذاتی ذہنی دباؤ (stress) کی درجہ بندی کس طرح کریں گیں؟

- پہلے سے بہت کم دباؤ1
- پہلے سے کم دباؤ2
- کوئی تبدیلی نہیں3
- پہلے سے زیادہ دباؤ4
- پہلے سے بہت زیادہ دباؤ5

2. آپ اپنے گھرانے کے ذہنی دباؤ (stress) کی درجہ بندی کس طرح کریں گیں؟

- پہلے سے بہت کم دباؤ1
- پہلے سے کم دباؤ2
- کوئی تبدیلی نہیں3
- پہلے سے زیادہ دباؤ4
- پہلے سے بہت زیادہ دباؤ5

3. آپ اپنی نفسیاتی صحت کی درجہ بندی کس طرح کریں گیں؟

- پہلے سے بہت بہتر1
- پہلے سے بہتر2
- کوئی تبدیلی نہیں3
- پہلے سے بدتر4
- پہلے سے بہت بدتر5

For peer review only

For peer review only - http://bmjopen.bmj.com/site/about/guidelines.xhtml

4. آپ اپنے گھرانے کی نفسیاتی صحت کی درجہ بندی کس طرح کریں گیں؟

- ◯ پہلے سے بہت بہتر1
- ◯ پہلے سے بہتر2
- ◯ کوئی تبدیلی نہیں3
- ◯ پہلے سے بدتر4
- ◯ پہلے سے بہت بدتر5

For peer review only

K-10

نیچے دئیے گئے دس سوالات میں آپ سے پوچھا گیا ہے کہ آپ پچھلے **4** ہفتوں سے کیسا محسوس کر رہے ہیں۔ برائے مہربانی ہر سوال کے لئے نشاندہی کریں جو اس بات کی بہترین وضاحت کرتا ہو کہ آپ نے اس کیفیت کو کتنی مرتبہ محسوس کیا ہے۔

	کبھی نہیں 1	بہت کم وقت 2	کبھی کبھار 3	زیادہ وقت 4	ہر وقت 5
1. پچھلے 4 ہفتوں میں، آپ نے کتنی مرتبہ بغیر کسی معقول وجہ کے تھکاوٹ محسوس کی؟					
2. پچھلے 4 ہفتوں میں، آپ نے کتنی مرتبہ گھبراہٹ محسوس کی؟					
3. پچھلے 4 ہفتوں میں، آپ نے کتنی مرتبہ اتنی گھبراہٹ محسوس کی کہ کوئی بھی چیز آپ کو سکون نہ دے سکی؟					
4. پچھلے 4 ہفتوں میں، آپ نے کتنی مرتبہ نا امُیدی محسوس کی؟					
5. پچھلے 4 ہفتوں میں، آپ نے کتنی مرتبہ بے سکونی یا بے چینی محسوس کی؟					
6. پچھلے 4 ہفتوں میں، آپ نے کتنی مرتبہ اتنی بے چینی محسوس کی کہ آپ ٹک کے نہ بیٹھ سکتے تھے؟					
7. پچھلے 4 ہفتوں میں، آپ نے کتنی مرتبہ افسردگی (ڈپریشن) محسوس کی*					
8. پچھلے 4 ہفتوں میں، آپ نے کتنی مرتبہ محسوس کیا کہ ہر کام کرنے کے لیے کوشش کرنی پڑ رہی ہے؟					
9. پچھلے 4 ہفتوں میں، آپ کتنی مرتبہ اتنے اداُس تھے کہ کوئی بھی چیز آپ کو خوش نہ کر سکی؟					
10. پچھلے 4 ہفتوں میں، آپ نے کتنی مرتبہ خود کو بے وقعت محسوس کیا؟					

For peer review only

For peer review only - http://bmjopen.bmj.com/site/about/guidelines.xhtml

PCL-5

نیچے ان مسائل کی فہرست دی گئی ہے جو لوگوں کو کوویڈ-19 کے نتیجے میں پیش آ سکتے ہیں۔ براہ مہربانی ہر مسئلے کو غور سے پڑھیں اور پھر دیے گئے نمبروں میں سے کسی ایک کو چن کر نشاندہی کریں کہ پچھلے مہینے میں آپ کو اس مسئلے نے کتنا پریشان کیا ہے۔

بالکل بھی نہیں<br>0	تھوڑا سا<br>1	درمیانی حد تک<br>2	زیادہ<br>3	بہت زیادہ<br>4

For peer review only

For peer review only - http://bmjopen.bmj.com/site/about/guidelines.xhtml

1. پریشان کن تجربے کی بار بار آنے والی، تکلیف ده، اور ناپسندیده یادیں؟	○	○	○	○	○
2. پریشان کن تجربے کے بار بار آنے والے، تکلیف ده خواب؟	○	○	○	○	○
3. اچانک محسوس ہونا یا ایسا لگنا کہ پریشان کن تجربہ واقعی دوبارہ ہو رہا ہے (جیسے آپ ماضی میں چلے گئے ہوں اور اس سے دوبارہ گزر رہے ہوں)۔	○	○	○	○	○
4. بہت گھبراہٹ محسوس کرنا جب کوئی چیز آپ کو پریشان کن تجربے کی یاد دلائے۔	○	○	○	○	○
5. سخت جسمانی رد عمل محسوس کرنا جب کوئی چیز آپ کو پریشان کن تجربے کی یاد دلائے (مثلاً، دل کا زور زور سے دھڑکنا، سانس لینے میں دشواری، پسینہ چھوٹنا)؟	○	○	○	○	○
6. پریشان کن تجربے سے متعلق یادوں، خیالات، یا احساسات سے گریز کرنا؟	○	○	○	○	○
7. پریشان کن تجربے کی بیرونی یاد دہانیوں سے گریزکرنا (مثلاً لوگ، مقامات، گفتگو، سرگرمیاں، اشیاء، یا حالات)؟	○	○	○	○	○
8. پریشان کن تجربے کے اہم حصوں کو یاد کرنے میں دقت محسوس کرنا؟	○	○	○	○	○
9. اپنے، دوسرے لوگوں، یا دنیا کے بارے میں سخت منفی خیالات آنا (مثلاً، اس طرح کے خیالات جیسے :میں برا/بری ہوں، میرے ساتھ واقعی میں کچھ مسئلہ ہے، کسی پر بھی اعتبار نہیں کیا جاسکتا، پوری دنیا خطرناک ہے)؟	○	○	○	○	○
10. پریشان کن تجربے کے لئے یا اس کے بعد جو کچھ ہوا اس کے لئے اپنے آپ کو یا کسی اور کو مورد الزام ٹھہرانا؟	○	○	○	○	○
11. سخت منفی جذبات کا ہونا جیسے خوف، وحشت، غصہ، احساس جرم، یا شرمندگی ؟	○	○	○	○	○
12. ان سرگرمیوں میں عدم دلچسپی جن سے آپ کبھی لطف اندوز ہوتے تھے؟	○	○	○	○	○
13. دوسرے لوگوں سے دوری محسوس کرنا یا تعلقات منقطع کرنا؟	○	○	○	○	○
14. مثبت احساسات محسوس کرنے میں دشواری (مثلاً، آپ کا اپنے قریبی لوگوں کے لئے خوشی یا محبت کے جذبات محسوس کرنے سے قاصر ہونا)؟	○	○	○	○	○

For peer review only

For peer review only - http://bmjopen.bmj.com/site/about/guidelines.xhtml

15. چڑچڑاپن، برہمی، یا جارحانہ سلوک؟	○	○	○	○	○
16. بہت زیادہ خطرات مول لینا یا ایسی چیزیں کرنا جو آپ کو نقصان پہنچا سکتی ہیں؟	○	○	○	○	○
17. انتہائی الرٹ/چوکنا یا محتاط رہنا؟	○	○	○	○	○
18. بہت تیزی یا آسانی سے چونک جانا؟	○	○	○	○	○
19. توجہ دینے میں دقت پیش آنا؟	○	○	○	○	○
20. سونے میں دشواری یا نیند کے مسائل؟	○	○	○	○	○

WHO-5

براہِ مہربانی ان پانچ بیانات میں سے ہر ایک کے لئے نشاندہی کریں کہ آپ پچھلے دو ہفتوں سے کیسا محسوس کر رہے ہیں۔

	**ہر وقت 5**	**زیادہ تر وقت 4**	**آدھے سے زیادہ وقت میں 3**	**آدھے سے کم وقت میں 2**	**کچھ وقت 1**	**کسی وقت بھی نہیں 0**
1. میں نے خوشی اور زندہ دلی محسوس کی	○	○	○	○	○	○
2. میں نے سکون اور راحت محسوس کیا	○	○	○	○	○	○
3. میں نے فعال اور چست محسوس کیا	○	○	○	○	○	○
4. میں نے سو کر اٹھتے ہوئے تازہ دم اور آرام دہ محسوس کیا	○	○	○	○	○	○
5. میری روزمرہ زندگی ایسی چیزوں سے بھری پڑی ہے جن میں مجھے دلچسپی ہے	○	○	○	○	○	○

For peer review only

For peer review only - http://bmjopen.bmj.com/site/about/guidelines.xhtml

PGTI

درج ذیل بیانات میں سے ہر ایک کی دیے گئے اسکیل کی مدد سے نشاندہی کریں کہ کوویڈ 19-کے نتیجے میں آپ کی زندگی میں کس درجہ کی تبدیلی واقع ہوئی ہے۔

بہت زیادہ<br>5	زیادہ<br>4	درمیانی حد تک<br>3	تھوڑا<br>2	بہت تھوڑا<br>1	بالکل نہیں<br>0

For peer review only

For peer review only - http://bmjopen.bmj.com/site/about/guidelines.xhtml

○	○	○	○	○	○	1. زندگی میں کیا اہم ہے اس کے بارے میں میں نے اپنی ترجیحات کو تبدیل کر دیا ہے
○	○	○	○	○	○	2. مجھے اپنی زندگی کی قدروقیمت پہلے سے زیادہ ہے
○	○	○	○	○	○	3. میں نے نئی دلچسپیاں بنا لی ہیں
○	○	○	○	○	○	4. مجھے احساس خود انحصاری پہلے سے زیادہ ہے
○	○	○	○	○	○	5. مجھے روحانی امور کی سمجھ بوجھ پہلے سے بہتر ہے
○	○	○	○	○	○	6. میں پہلے سے بہتر طور پر دیکھ سکتا /سکتی ہوں کہ میں مصیبت کے وقت لوگوں پر بھروسہ کر سکتا / سکتی ہوں
○	○	○	○	○	○	7. میں نے اپنی زندگی کی ایک نئی راہ متعین کر لی ہے
○	○	○	○	○	○	8. مجھے دوسروں کے ساتھ قربت کا احساس پہلے سے زیادہ ہے
○	○	○	○	○	○	9. میں اپنے جذبات کا اظہار کرنے کے لیے پہلے سے زیادہ آمادہ ہوں
○	○	○	○	○	○	10. میں پہلے سے بہتر جانتا / جانتی ہوں کہ میں مشکلات کا مقابلہ کر سکتا / سکتی ہوں
○	○	○	○	○	○	11. میں اپنی زندگی میں پہلے سے بہتر کام سَر انجام دے رہا / رہی ہوں
○	○	○	○	○	○	12. میری چیزوں کا انجام قبول کرنے کی صلاحیت پہلے سے بہتر ہے
○	○	○	○	○	○	13. میں ہر دن کی قدر پہلے سے زیادہ کرتا / کرتی ہوں
○	○	○	○	○	○	14. نئے مواقع میسر ہیں جو بصورت دیگر میسر نہ ہوتے
○	○	○	○	○	○	15. مجھ میں دوسروں کے لئے شفقت پہلے سے زیادہ ہے
○	○	○	○	○	○	16. میں اپنے تعلقات نبھانے میں پہلے سے زیادہ محنت کرتا / کرتی ہوں

For peer review only

.17 میں ان چیزوں کو تبدیل کرنے کی پہلے سے زیادہ کوشش کرتا / کرتی ہوں جن کو تبدیل کرنے کی ضرورت ہے	○	○	○	○	○	○
.18 میرا ایمان پہلے سے زیادہ مضبوط ہے	○	○	○	○	○	○
.19 میں نے جان لیا ہے کہ میں اپنی سوچ سے ذیادہ مضبوط ہوں	○	○	○	○	○	○
.20 میں نے پہلے سے بھی ذیادہ سیکھا ہے کہ لوگ کتنے اچھے ہیں	○	○	○	○	○	○
.21 میں دوسروں سے ملنے والی مدد کو قبول کرنے کے لیے پہلے سے زیادہ آمادہ ہوں	○	○	○	○	○	○

Helpline Information

کوویڈ سے متعلق معلومات کے لیے، براہ کرم https://covid.gov.pk/ پر جائیں۔

ہیلپ لائن کے لیے 1166 ڈائل کریں۔

For peer review only - http://bmjopen.bmj.com/site/about/guidelines.xhtml

For peer review only

BMJ Open

Examining the Psychosocial Impacts of the COVID-19 Pandemic: An International Cross-sectional Study Protocol

Journal:	*BMJ Open*
Manuscript ID	bmjopen-2022-067886.R1
Article Type:	Protocol
Date Submitted by the Author:	09-Feb-2023
Complete List of Authors:	tanveer, sandila; University of Otago Christchurch, Department of Psychological Medicine<br>Schluter, Philip; University of Canterbury, School of Health Science; University of Queensland, School of Nursing, Midwifery and Social Work<br>Porter, Richard; University of Otago<br>Boden, Joseph; University of Otago<br>Beaglehole, Ben; University of Otago, Psychological Medicine<br>Sulaiman-Hill, Ruqayya; University of Otago Christchurch, Psychological Medicine<br>Dean, Shaystah; University of Otago Christchurch, Psychological Medicine; University of Otago Wellington Department of Medicine, Psychological Medicine<br>Bell, Romana; Australian National University, Department of Anthropology<br>Al-Hussainni, Wafaa N; Ibn Sina University of Medical and Pharmaceutical Sciences, Basic Sciences Deptartment<br>Arshi, Maliheh ; University of Social Welfare and Rehabilitation Science, Department of Social Work<br>Amer Nordin, Amer Siddiq; University of Malaya, Psychological Medicine<br>Dinç, Mehmet; Hasan Kalyoncu University, Department of Psychology<br>Khan, Mussarat ; International Islamic University, Department of Psychology<br>Khoshnami, Mohammad ; University of Social Welfare and Rehabilitation Science, Department of Social Work,<br>Majid Al-Masoodi, Muthana A; Cultural Relations Mustansiryah University, Department of Scholarships<br>Moghanibashi-Mansourieh, Amir; University of Social Welfare and Rehabilitation Science<br>Noruzi, Sara; Lorestan University of Medical Sciences,<br>Rahajeng, Anggi; Universitas Gadjah Mada<br>Shaikh, Shaista; Islamabad Model College for Girls (PostGraduate), Department of Psychology<br>Tanveer, Nisa; National Defence University, Islamabad Pakistan, Department of Peace and Conflict Sciences<br>Topçu, Feyza; Hasan Kalyoncu University, Department of Psychology<br>Yapan, Saadet; Hasan Kalyoncu University, Department of Psychology<br>Yunianto, Irfan; Faculty of Teacher Training and Education Universitas Ahmad Dahlan<br>Zoellner, Lori; University of Washington<br>Bell, Caroline; University of Otago, Department of Psychological Medicine

**Primary Subject Heading**:	Mental health
Secondary Subject Heading:	Public health
Keywords:	MENTAL HEALTH, COVID-19, PUBLIC HEALTH

SCHOLARONE™ Manuscripts

Examining the Psychosocial Impacts of the COVID-19 Pandemic: An International Cross-sectional Study Protocol

Corresponding Author

Associate Professor Caroline Bell, Department of Psychological Medicine, University of Otago Christchurch, P.O. Box 4345, Christchurch 8140, New Zealand, caroline.bell@otago.ac.nz

Authors

Dr Sandila Tanveer, Department of Psychological Medicine, University of Otago, Christchurch, New Zealand

Professor Philip J. Schluter, Te Kaupeka Oranga, Faculty of Health, Te Whare Wānanga o Waitaha, University of Canterbury, Christchurch, New Zealand; and School of Clinical Medicine, Primary Care Clinical Unit, University of Queensland, Brisbane, Australia

Professor Richard J. Porter, Department of Psychological Medicine, University of Otago, Christchurch, New Zealand

Professor Joseph M. Boden, Department of Psychological Medicine, University of Otago, Christchurch, New Zealand

Dr Ben Beaglehole, Department of Psychological Medicine, University of Otago, Christchurch, New Zealand

For peer review only

For peer review only - http://bmjopen.bmj.com/site/about/guidelines.xhtml

Dr Ruqayya Sulaiman-Hill, Department of Psychological Medicine, University of Otago, Christchurch, New Zealand

Dr Shaystah Dean, Department of Psychological Medicine, University of Otago, Wellington, New Zealand

Romana Bell, Department of Anthropology, Australian National University, Canberra, ACT, Australia

Dr Wafaa N Al-Hussainni, Basic Sciences Department, Ibn Sina University of Medical and Pharmaceutical Sciences, Baghdad, Iraq

Dr Maliheh Arshi, Department of Social Work, University of Social Welfare and Rehabilitation Sciences, Tehran, Iran

Associate Professor Amer Siddiq Bin Amer Nordin, Department of Psychological Medicine, Universiti Malaya, Kuala Lumpur, Malaysia

Dr Mehmet Dinç, Department of Psychology, Hasan Kalyoncu University, Gaziantep, Turkiye

Dr Mussarat Jabeen Khan, Department of Psychology, International Islamic University, Islamabad, Pakistan

Dr Mohammad Sabzi Khoshnami, Department of Social Work, University of Social Welfare and Rehabilitation Sciences, Tehran, Iran

Dr Muthana A Majid Al-Masoodi, Department of Scholarships and Cultural Relations, Mustansiryah University, Baghdad, Iraq

For peer review only

For peer review only - http://bmjopen.bmj.com/site/about/guidelines.xhtml

Dr Amir Moghanibashi-Mansourieh, Department of Social Work, University of Social Welfare and Rehabilitation Sciences, Tehran, Iran

Dr Sara Noruzi, Lorestan University of Medical Sciences, Khorramabad, Iran

Anggi Rahajeng, Faculty of Economics and Business, Universitas Gadjah Mada, Yogyakarta, Indonesia

Shaista Sheikh, Department of Psychology, Islamabad Model College for Girls (Postgraduate), Islamabad, Pakistan

Nisa Tanveer, Department of Peace and Conflict Sciences, National Defence University, Islamabad Pakistan

Feyza Topçu, Department of Psychology, Hasan Kalyoncu University, Gaziantep, Turkiye

Dr Saadet Yapan, Department of Psychology, Hasan Kalyoncu University, Gaziantep, Turkiye

Dr Irfan Yunianto, Faculty of Teacher Training and Education, Universitas Ahmad Dahlan, Yogyakarta, Indonesia

Professor Lori A Zoellner, Department of Psychology, University of Washington, Seattle, USA

Associate Professor Caroline Bell, Department of Psychological Medicine, University of Otago, Christchurch, New Zealand

For peer review only

For peer review only - http://bmjopen.bmj.com/site/about/guidelines.xhtml

Word Count: 3478

Keywords

Psychosocial impacts, mental health, wellbeing, COVID-19, low and middle-income countries, cross-cultural.

For peer review only

For peer review only - http://bmjopen.bmj.com/site/about/guidelines.xhtml

ABSTRACT

Introduction

The novel coronavirus disease 2019 (COVID-19) exposed people to significant and prolonged stress. The psychosocial impacts of the pandemic have been well recognised and reported in high-income countries (HICs) but it is important to understand the unique challenges posed by the COVID-19 pandemic in low and middle-income countries (LMICs) where limited international comparisons have been undertaken. This protocol was therefore devised to study the psychosocial impacts of the COVID-19 pandemic in seven LMICs using scales that had been designed for or translated for this purpose.

Methods and analysis

This cross-sectional study uses an online survey to administer a novel measure of COVID-19-specific stressors alongside established measures of psychological distress, post-traumatic stress, wellbeing, and post-traumatic growth in the appropriate language for the site. Participants will include adults aged 18 years and above, recruited from Indonesia, Iraq, Iran, Malaysia, Pakistan, Somalia, and Turkey, with a pragmatic target sample size of 500 in each country.

Data will be analysed descriptively on sociodemographic and study variables. In addition, CPIS will be analysed psychometrically (for reliability and validity) to assess the suitability of use in a given context. Lastly, within-subjects and between-subjects

For peer review only - http://bmjopen.bmj.com/site/about/guidelines.xhtml

For peer review only

analyses will be carried out using multi-level mixed-effect models to examine associations between key sociodemographic and study variables.

Ethics and dissemination

Ethical approval was granted by the Human Ethics Committee, University of Otago, New Zealand (Ref No. 21/102). In addition, international collaborators obtained local authorisation or ethical approval in their respective host universities before data collection commenced.

Registration details

The study is registered with the Clinical Trial Registry (NCT05052333).

For peer review only

Strengths and limitations of this study

- This cross-sectional observational study will make a significant contribution to understanding the psychosocial impacts of the COVID-19 pandemic in seven LMICs with different pandemic impacts.
- This study will quantify psychometric measures in countries where there is currently limited access to newly developed scales relating to COVID-19 and associated measures of psychological distress, wellbeing, and post-traumatic growth in appropriate languages.
- The findings will provide psychometric information on the novel CPIS in differing pandemic contexts.
- There are limitations associated with selective recruitment and response rate in this study; which may limit the generalisability of the findings and comparability between groups.

For peer review only

For peer review only - http://bmjopen.bmj.com/site/about/guidelines.xhtml

INTRODUCTION

Coronavirus 2019 (COVID-19), was identified as an infectious disease by the World Health Organization (WHO) on 11 February 2020[1]. This new virus rapidly spread and was declared an international public health emergency on 30 January 2020 and a pandemic on 11 March 2020[1]. As of 31 January 2023, there have been 753,497,572 confirmed cases of COVID-19 including 6,813,301 deaths reported by WHO[2]; and likely many more as a result of under-reporting[3]. The COVID-19 pandemic has been complex in its origin, spread, impacts, and consequences. Specifically, COVID-19 exposed people to significant and prolonged stress from often overlapping concerns like health consequences of COVID-19, impacts of public health restrictions (e.g., lockdowns, social distancing, travel bans), financial losses, vaccination mandates, and associated societal divisions[4].

When examining the psychosocial impacts in a pandemic context, researchers experience key challenges not only with the choice of study design and measures but also in providing a multicultural perspective[5]. In this respect, researchers have obtained data using convenience[6,7] or representative sampling[8] by employing either cross-sectional[8] or longitudinal[7,9] design. Those studies have been carried out either on the general population[6-8] or on potentially vulnerable populations (such as the health workforce[10-12] or those with pre-existing risk factors such as having a previous history of mental disorders[9,13,14]). Most studies include self-report measures of wellbeing, psychological distress, depression, anxiety, and post-

traumatic stress disorder (PTSD)[15]. However, these standardised measures do not examine the particular impacts of COVID-19-related stressors and there has been a call for novel COVID-19-specific measures to be used alongside established psychometric scales[16]. In response, several COVID-19-specific scales have been developed which vary in focus and length. For example, one scale assesses fear associated with COVID-19 infection[17] (see also[6]), while the other one examined a wide range of impacts of the pandemic and public health restrictions on physical health, employment and finances, and family and social disruption[18]. Notably, the latter scale, in attempting to capture the wider psychosocial impacts, frequently reflects the context and country in which they were developed, limiting their more general applicability in other contexts. Researchers have also attempted to develop and validate brief screening tools for clinical research and practice; e.g., the 5-item Coronavirus Anxiety Scale[19] and the 7-item COVID-19 Anxiety Scale[20]. In addition, while the positive outcomes of the pandemic have been recognised[21], few studies formally assessed the positive impacts[22,23], which may limit a comprehensive understanding of the psychosocial impacts of COVID-19.

To date, the majority of the literature examining the psychosocial impacts of COVID-19 is from studies conducted in China, Europe, and the United States of America (USA) and a few multinational studies have been carried out among the general population. A multinational study that examined the psychosocial impacts of COVID-19 across several high-income countries (HICs) found generalized anxiety among 21.0% and major depression among 25.5% of their respondents[24]. Another study

For peer review only - http://bmjopen.bmj.com/site/about/guidelines.xhtml

that examined the psychological impacts of COVID-19 across several HICs and low- and middle-income countries (LMICs), reported high rates of anxiety (28.2%), stress (18.3%), and depression (26.6%) in their community-based samples[25] (see also[26] for depression prevalence in LMICs). Some multinational studies have reported significant differences in these psychological impacts across countries [26,27]. In comparison, studies investigating the psychosocial impacts in other regions, especially in LMICs are scarce, despite many of these areas being severely affected by COVID-19. The difficulties in evaluating the pandemic-related psychological impacts in these regions are compounded by the lack of standardised measures in the local languages of these countries, the absence of which limits the comparison of results between countries.

To address the issues identified above, we developed the 50-item COVID Psychosocial Impacts Scale (CPIS) to comprehensively assess the impacts of the pandemic including the adverse and positive personal, social, and economic consequences that followed. The construction of the scale was informed by our team's experience assessing the psychosocial impacts of the Canterbury Earthquakes[28,29] and the Mosque Attacks in 2019[30]. Notably, the existing pandemic-related scales were also consulted in scale development[6,16-20]. The items and subscales of CPIS were modelled on the Social Readjustment Scale[31] assessing exposure to pandemic-related life events (disruptions to personal, family, employment, faith, education, and daily routines) and the resultant level of stress. It also included questions examining the potential positive consequences of the

COVID-19 pandemic. The CPIS was refined using the 'group mind' process[32], with colleagues from a range of countries and cultures (Bangladesh, Ghana, Pakistan, and Turkey) reviewing a draft of the questionnaire, with iterative improvements based on their comments. The applicability of the CPIS for use in a range of countries and cultures was duly considered through collaboration with international colleagues.

The 50-item CPIS has been validated in a non-representative New Zealand sample at two-time points (2020, 2022) when the population exposure to COVID-19 community infections varied markedly[33]. In that study, the CPIS was administered with a measure of psychological distress (Kessler Psychological Distress Scale, K10)[34] and wellbeing (World Health Organization Well-Being Index, WHO-5)[35]. Findings indicate a unidimensional structure within CPIS subscales while it correlates with psychological distress and general wellbeing is not only a proxy for these constructs[33]. Based on findings from the validation study, we found that some items were less important in future iterations of the CPIS. Therefore, we have refined CPIS from 50 to 32 items.

In addition to developing the CPIS, the research team took the opportunity to use resources that had already been deployed for the translation of the research instruments for the March 15th Project[30] to develop the CPIS into several different language versions (Arabic, Indonesian, Malay, Persian, Somali, Turkish, and Urdu). To ensure that translations captured psychometric meanings in a culturally

For peer review only - http://bmjopen.bmj.com/site/about/guidelines.xhtml

appropriate way, translations were carried out by proficient bilingual Research Assistants and interpreters who were familiar with the content and context, using parallel and back-to-back translations in an iterative process. These translations were then compared for internal consistency and examined for face validity.

Our planned programme of work is to examine the 32-item CPIS and translated psychometric measures in the countries of interest. This protocol describes a cross-sectional observational study to assess the psychosocial impacts of the COVID-19 pandemic in seven LMICs (Indonesia, Iran, Iraq, Malaysia, Pakistan, Somalia, and Turkey). It utilises an online survey that includes the 32-item CPIS and standardised measures of psychological distress, post-traumatic stress, wellbeing, and posttraumatic growth. Our findings will increase our understanding of the psychosocial impacts of the COVID-19 pandemic across several LMICs with differing rates of infection, morbidity, and mortality. Findings will also provide psychometric evidence on the novel the CPIS. As a result, our research will contribute to a growing body of mental health measures that can be used for cross-cultural comparison.

For peer review only

METHODS AND ANALYSIS

Study design

This is a cross-sectional observational study examining the psychosocial impacts of the COVID-19 pandemic being conducted in seven LMICs.

Measures

The measures have all been translated into the appropriate language for the country in which they will be administered (see supplementary file). They include the following.

Sociodemographic measures

Sociodemographic measures include age, gender, marital status, religion, ethnicity, self-reported English proficiency, highest level of education, occupation, study or work status, and monthly income (in local currency). On the recommendation of the international collaborators, some of the demographic questions may vary to ensure contextual appropriateness. For example, in Turkey, students do not study part-time so that option was removed from the item.

Prior trauma exposure

The exposure to previous trauma will also be sought to examine the effect of previous trauma on the psychosocial impacts of COVID-19. Participants will be asked to select as many as appropriate from a list of previous exposure to natural

For peer review only

disasters, war or military conflict, childhood adversity, physical or sexual assault, and serious physical accident.

COVID Psychosocial Impacts Scale (CPIS)

This is a novel 32-item measure used to examine the psychosocial impacts of the COVID-19 pandemic. The scale asks participants if they have experienced stressors in response to COVID-19 and then to indicate the magnitude of stress on a six-point scale scored from 0 = ‘no’, 1 = ‘yes, no stress at all’ to 5 = ‘yes, a lot of stress’. An overall pandemic-related stress score can be calculated ranging from 0 to 160.

For peer review only

For peer review only - http://bmjopen.bmj.com/site/about/guidelines.xhtml

Kessler-10 (K10)

This is a 10-item measure assessing symptoms of psychological distress in the previous 30 days[34]. Each item is scored from 1 = 'none of the time' to 5 = 'all of the time'. The raw score can range from 10 to 50; with scores 30 and above indicating severe mental distress[36].

WHO Well-Being Index (*WHO-5)*

This is a short 5-item measure assessing subjective psychological wellbeing in the previous two weeks[35]. Each item is scored from 0 = 'at no time' to 5 = 'all of the time'. The raw score range is 0–25; this can be multiplied by 4 to give a final score, with 100 representing the highest subjective wellbeing rating.

PTSD Checklist for DSM-5 (*PCL-5)*

This is a 20-item measure assessing symptoms of PTSD in the past month anchored in response to COVID-19 as a potentially traumatic event. Each item is scored from 0 = 'not at all' to 4 = 'extremely'; with a severity score range of 0–80[37].

Posttraumatic Growth Inventory (*PTGI)*

This is a 21-item measure assessing change following exposure to trauma[38], adapted to reflect how change occurred in your life as a result of COVID-19. There are five subscales (relating to others, new possibilities, personal strength, spiritual change, and appreciation of life). Each item is scored from 0 = 'not at all' to 5 = 'a lot'

For peer review only - http://bmjopen.bmj.com/site/about/guidelines.xhtml

with a score range of 0–105, with higher scores reflecting positive transformation after the traumatic event.

Participants

Adults (aged 18 years and over) who reside in Indonesia, Iran, Iraq, Malaysia, Pakistan, Somalia, or Turkey will be recruited as participants. Anyone aged below 18 years and those not currently residing in the country of interest will be excluded from the study.

Study Status

Different sites are at different stages of project implementation including ethical approval pending (Iran); ethical approval obtained and recruiting participants (Somalia); and participant recruitment completed (Indonesia, Iraq, Malaysia, Pakistan, and Turkey).

Recruitment

The mode of recruitment will vary between countries, but will generally involve the distribution of the survey link using a participant mailing list or social media list held by each international collaborator at the host site. The local researchers will be using sampling techniques that are appropriate within their cultures and contexts, which for the most part resemble snowball sampling techniques.

Procedure

For peer review only - http://bmjopen.bmj.com/site/about/guidelines.xhtml

For peer review only

Participants receive a survey link via the recruitment method above. The online survey comprises a participant information sheet, consent form, and questionnaires. If a subject consents, they are asked to complete the online survey (which includes the self-report measures described above) using the Qualtrics XM online survey platform. The survey takes approximately 15 to 20 minutes to complete and is provided in the appropriate language for the site. Information is provided about local supports that can be accessed if the self-report questions were to cause distress.

Patient and Public Involvement

The project has been designed with community and general public involvement. Feedback has also been taken on project design from academics and the general public in the countries of interest.

Data management

To ensure the anonymity of the obtained dataset the following standard operating procedures are in place. Where applicable, only the international collaborators hold contact information for their participant pool. Each site has specific guidelines to preserve anonymity specified in their local ethics application. Following collection, data will be saved on the University of Otago, New Zealand Qualtrics platform using an auto-generated non-identifiable letter-number string. Importantly, the obtained data set will be completely anonymous as no identifiable information is obtained in the survey.

For peer review only - http://bmjopen.bmj.com/site/about/guidelines.xhtml

Data analysis

Incomplete survey responses will be removed from the analyses. Statistical analyses will be carried out to examine the psychosocial impacts of the COVID-19 pandemic in different countries and provide psychometric evidence using SPSS Version 28.0[39] and Stata Release 17[40]. Reporting will follow 'Strengthening the Reporting of Observational Studies in Epidemiology' (STROBE) guidelines[41].

Sample size estimation

As no prevalence of the psychosocial impacts of the COVID-19 pandemic in the general population of the countries of interest exists, no formal sample size calculations were conducted. The final target sample size of n=500 per country was pragmatically selected balancing the competing demands of maximizing statistical power, expedited cost-effective data collection processes, and international reach while simultaneously minimizing institutional burden[42].

Psychometric analyses

Reliability analyses will be carried out on each measure to determine the suitability of its use, employing item-total correlations and Cronbach's α as a measure of internal homogeneity and consistency. The construct validity of CPIS with WHO-5 and K10 will be examined using a correlation matrix.

Within-subjects analyses

Descriptive analysis will be carried out on socio-demographics (e.g., age, gender, ethnicity, religion, marital status, the highest level of education, work status, monthly

For peer review only - http://bmjopen.bmj.com/site/about/guidelines.xhtml

household income, exposure to previous trauma) and study variables (wellbeing, psychological distress, post-traumatic stress, post-traumatic growth and pandemic-related stress from the CPIS) to inform site-specific sociodemographic profile and psychosocial outcomes. To explore the relationship between socio-demographic and study variables, follow-up analyses will be carried out using a linear mixed model (also known as multi-level modelling) to pool predictor variables. This will be carried out for each of the seven sites individually.

For peer review only

For peer review only - http://bmjopen.bmj.com/site/about/guidelines.xhtml

Between-subjects analyses

To look at empirical distributions across countries, graphing superimposed Cumulative Distribution Function (cdf) will be used. In addition, to understand the role of demographic and study variables, multi-level modelling will be carried out in which participants will be nested within their countries – which will be treated as random effects to investigate the association between probable distress and wellbeing indicators and sociodemographic and pandemic-related variables. This approach allows individual and country-level factors to be included. Residual diagnostics and model assumptions will also be checked.

DISCUSSION

This is a cross-sectional observational research that examines the psychosocial impacts of the COVID-19 pandemic across seven LMICs using a novel CPIS and measures of wellbeing, psychological distress, post-traumatic stress, and post-traumatic growth. Despite LMICs being significantly impacted by COVID-19, few studies have been published from these regions. This study will therefore provide findings to address this important gap in the literature. The multi-centre design of the study will allow comparisons between the empirical distributions across countries. Furthermore, by including individual and country-level factors, multi-level modelling will enable us to understand the role of demographic and study variables. The findings have the potential to contribute to the understanding of the psychosocial

For peer review only - http://bmjopen.bmj.com/site/about/guidelines.xhtml

impacts of the COVID-19 pandemic in countries that have not been well studied and to allow cross-cultural, multi-site comparisons.

Several multinational studies have been carried out examining the psychosocial impacts of COVID-19 among the general population using either standardised measures alone[11,24,25,27,43] or in combination with novel instruments[26,44]. These studies mostly obtained data from non-representative samples (except one[24]) from HICs[24,45,46], LMICs[26], or across HICs and LMICs[25,27,43,44]. These studies have reported different rates in the prevalence of depression[11,24-27,43], anxiety[24,25,43], stress[11,25,27], and PTSD[11]. Few studies have examined the sociodemographic variables in relation to COVID-19, and have found an association of depression with younger age, gender (female), high levels of exposure, and stigmatization related to COVID-19[26,45]. Others have reported the association of stress with gender (female), marital status (single), no formal education level, religion, being exposed to a confirmed or suspected COVID-19 patient, and being forced to be quarantined[44,46].

Against this background, our study will comprehensively examine the psychosocial impacts of COVID-19 using standardised measures in combination with novel CPIS across seven LMICs examining both socio-demographics and study variables. Our study has the potential to make several contributions to the existing literature. The 32-item CPIS is a novel scale that comprehensively assesses the psychosocial impacts of the pandemic with the scale design allowing assessment of both the number of exposures to specific pandemic-related life events and the associated

For peer review only - http://bmjopen.bmj.com/site/about/guidelines.xhtml

level of stress related to each of these events. The psychosocial impacts of the pandemic-related life events will be examined cross-culturally in this study which distinguishes it from the existing multinational studies that mainly focus on the prevalence of mental health disorders and do not examine the adverse personal, social, and economic consequences that followed COVID-19 pandemic. Importantly, the multi-lingual nature of the study is an important contribution. An underlying factor to the lack of studies in LMIC has been the lack of availability of measures translated into appropriate languages, which this study addresses. Hence, our findings will contribute to a growing body of mental health measures that can be used for cross-cultural comparison.

In addition, the study will also correlate outcomes on the CPIS with other validated measures of wellbeing and psychological distress and provide essential psychometric information on scale reliability and validity. Although, using pandemic-related scales in combination with standardised measures has been adopted by a few studies[26,44], but no formal psychometric analyses of the measures' suitability in varying contexts were undertaken. Additionally, to date, only a few nationwide studies have formally assessed potential positive outcomes associated with the pandemic[21-23]. The findings from this study will therefore contribute for the first time to this developing literature of examining the positive impacts of the COVID-19 pandemic in a cross-cultural context.

There are, however, limitations associated with the proposed study design. This study makes use of a novel CPIS developed during the COVID-19 pandemic and a

For peer review only - http://bmjopen.bmj.com/site/about/guidelines.xhtml

cross-sectional design. As a consequence, there is no pre-pandemic comparison data. Repeated use of the CPIS over time and during different phases of the pandemic is needed to inform whether the CPIS is responsive to changing impacts of the pandemic over time. As the data will be gathered through an online survey, it will only include participants with access to the internet. As a result, the sample might be less representative of persons who are illiterate or have limited access to or proficiency with computers or mobile phones. Since none of the sites systematically recruit, there are limitations associated with selective recruitment. As a result, we are unable to generalize the findings to the population of the relevant sites. In addition, the exposure and response rate will vary between countries, making cross-country comparisons challenging. These limitations will be mitigated with the use of multi-level modelling techniques to understand country-wise or cross-cultural predictor variables to obtain meaningful findings. Even though random sampling would be ideal for providing an unbiased representative sample of the overall population, this is not always possible due to resource and time constraints, as the majority of multinational studies relied on non-representative samples. Lastly, to minimise participant burden, the survey did not include an assessment of certain relevant variables—such as COVID-19-related stigmatisation, resilience, religious coping, or pre-existing mental health conditions.

Summary

This cross-sectional observational research examines the psychosocial impacts of the COVID-19 pandemic across seven LMICs (Indonesia, Iran, Iraq, Malaysia,

For peer review only - http://bmjopen.bmj.com/site/about/guidelines.xhtml

Pakistan, Somalia, and Turkey) using a novel 32-item CPIS and standardised measures examining wellbeing, psychological distress, post-traumatic stress, and post-traumatic growth. The findings will contribute to the understanding of the psychosocial impacts of the COVID-19 pandemic in different LMICs. In addition, it will provide psychometric data regarding CPIS in the countries of interest. This is a major contribution as there is currently limited access to newly developed scales relating to COVID-19 and associated measures of distress in appropriate languages. As a result, our research will contribute to a growing trend of establishing mental health measures that allow cross-cultural comparisons.

Ethics and dissemination

Ethical approval was granted by the Human Ethics Committee, University of Otago, New Zealand (Ref No. 21/102). In addition, international collaborators obtained local authorisation or ethical approval in their respective host universities [Indonesia (Universitas Gadjah Mada Ethics Committee Ref. No. KE/UGM/008/EC/2021); Iraq (Ibn Sina University of Medical and Pharmaceutical Sciences, Institutional Review Board (IRB) - Ethical Committee); Malaysia (Universiti Malaya Research Ethics Committee, Ref. No. UM.TNC 2/UMREC); Pakistan (International Islamic University Islamabad, Institutional Ethical Review Committee, Ref. No. IIUI/ORIC/Ethical-certificate/2021); Somalia (Washington Human Subjects Division (HSD) IRB ID: STUDY00015135); and Turkey (Hasan Kalyoncu Üniversitesi Etik Ethics Committee Ref. No. E-97105791-050.01.01-2342)] before data collection commenced.

For peer review only

Acknowledgement

We would like to thank the Research Assistants from the March 15 Project for their work in translating and back-translating study materials.

ABBREVIATIONS

cdf: Cumulative Distribution Function

CPIS: COVID Psychosocial Impacts Scale

HICs: High-income countries

K10: Kessler 10

LMIC: Low and middle-income countries

PCL-5: PTSD Checklist for DSM-5

PTGI: Post Traumatic Growth Inventory

WHO-5: WHO Well-Being Index

DECLARATIONS

Author Contributions

ST conceived the study, and CB, RS-H, RP JB, BB, PJS and SD contributed to the study design. ST, CB and RS-H developed the protocol and selected the measures. ST was responsible for coordinating instrument translation and setting up the online component. ST, WNA-H, MA, ASBAN, MD, MJK, MSK, MAMA-M, AM-M, SN, AR, SS, NT, FT, SY, IY, LAZ were responsible for obtaining ethics approval and data

For peer review only

acquisition. ST, RB, and CB drafted the paper. All authors read, critically revised, and approved the final version of the manuscript.

Funding Statement

There has been no funding for this study.

Competing Interests Statement

There are no competing interests.

For peer review only

For peer review only - http://bmjopen.bmj.com/site/about/guidelines.xhtml

REFERENCES

1 World Health Organization (2020). Listings of WHO's response to COVID-19. https://www.who.int/news/item/29-06-2020-covidtimeline

2 World Health Organization. WHO Coronavirus (COVID-19) dashboard. https://covid19.who.int/

3 World Health Organization. The true death toll of COVID-19: Estimating global excess mortality. https://www.who.int/data/stories/the-true-death-toll-of-covid-19-estimating-global-excess-mortality

4 Pfefferbaum B, North CS. Mental health and the Covid-19 pandemic. N. Engl. J. Med. 2020;383:510-512. doi:10.1056/NEJMp2008017

5 Bell C, Beaglehole B, Bell R, et al. Learning from previous disasters: potential pitfalls of epidemiological psychosocial research in the COVID-19 environment. Aust N Z J Psychiatry 2021;55(7):646-649. doi: 10.1177/0004867421998783.

6 Akan Y. Development of the "COVID-19 Psychological Impact Scale": a validity and reliability study. Curr Psychol 2022. doi:10.1007/s12144-022-02760-5

7 Fernández R, Crivelli L, Guimet N, et al. Psychological distress and mental health trajectories during the COVID-19 pandemic in Argentina: a longitudinal study. Sci Rep 2022;12(1). doi:10.1038/s41598-022-09663-2

For peer review only

For peer review only - http://bmjopen.bmj.com/site/about/guidelines.xhtml

8 Every-Palmer S, Jenkins M, Gendall P, et al. Psychological distress, anxiety, family violence, suicidality, and wellbeing in New Zealand during the COVID-19 lockdown: a cross-sectional study. PLoS One 2020;15(11):e0241658. doi:10.1371/journal.pone.0241658

9 Van Rheenen T, Meyer D, Neill E, et al. Mental health status of individuals with a mood-disorder during the COVID-19 pandemic in Australia: initial results from the COLLATE project. J Affect Disord 2020;275:69-77. doi:10.1016/j.jad.2020.06.037

10 Bell C, Williman J, Beaglehole B, et al. Challenges facing essential workers: a cross-sectional survey of the subjective mental health and well-being of New Zealand healthcare and 'other' essential workers during the COVID-19 lockdown. BMJ Open 2021;11(7):e048107. doi:10.1136/bmjopen-2020-048107

11 Chew N, Lee G, Tan B, et al. A multinational, multicentre study on the psychological outcomes and associated physical symptoms amongst healthcare workers during COVID-19 outbreak. Brain Behav Immun 2020;88:559-565. doi:10.1016/j.bbi.2020.04.049

12 Krishnamoorthy Y, Nagarajan R, Saya G, at al. Prevalence of psychological morbidities among general population, healthcare workers and COVID-19 patients amidst the COVID-19 pandemic: a systematic review and meta-analysis. Psychiatry Res 2020;293:113382. doi:10.1016/j.psychres.2020.113382

For peer review only

13 Bell C, Williman J, Beaglehole B, et al. Psychological distress, loneliness, alcohol use and suicidality in New Zealanders with mental illness during a strict COVID-19 lockdown. Aust N Z J Psychiatry 2021;56(7):800-810. doi: 10.1177/00048674211034317

14 Iasevoli F, Fornaro M, D'Urso G, et al. Psychological distress in patients with serious mental illness during the COVID-19 outbreak and one-month mass quarantine in Italy. Psychol Med 2020;51(6):1054-1056. doi:10.1017/S0033291720001841

15 Cénat J, Blais-Rochette C, Kokou-Kpolou C, et al. Prevalence of symptoms of depression, anxiety, insomnia, posttraumatic stress disorder, and psychological distress among populations affected by the COVID-19 pandemic: a systematic review and meta-analysis. Psychiatry Res 2021;295:113599. doi:10.1016/j.psychres.2020.113599

16 COVID-Minds. Recommended Scales 2020. https://www.COVIDminds.org/mental-health-measures

17 Ahorsu D, Lin C, Imani V, et al. The Fear of COVID-19 Scale: development and initial validation. Int J Ment Health Addict 2020;20(3):1537-1545. doi:10.1007/s11469-020-00270-8

18 CRISIS V0.1. The CoRonavIruS health Impact Survey 2020. http://www.crisissurvey.org/

For peer review only - http://bmjopen.bmj.com/site/about/guidelines.xhtml

19 Lee S. Coronavirus Anxiety Scale: a brief mental health screener for COVID-19 related anxiety. Death Stud 2020;44(7):393-401. doi:10.1080/07481187.2020.1748481

20 Silva W, de Sampaio Brito T, Pereira C. COVID-19 Anxiety Scale (CAS): development and psychometric properties. Curr Psychol 2020;41(8):5693-5702. doi:10.1007/s12144-020-01195-0

21 COVID-Minds. The psychological impact of COVID-19 2020. https://www.COVIDminds.org/post/february-update-the-psychological-impact-of-COVID-19

22 Beaglehole B, Williman J, Bell C, et al. Thriving in a pandemic: determinants of excellent wellbeing among New Zealanders during the 2020 COVID-19 lockdown; a cross-sectional survey. PLoS One 2022;17(3):e0262745. doi:10.1371/journal.pone.0262745

23 Jenkins M, Hoek J, Jenkin G, et al. Silver linings of the COVID-19 lockdown in New Zealand. PLoS One 2021;16(4):e0249678. doi:10.1371/journal.pone.0249678

24 Généreux M, Schluter PJ, Hung K, et al. One virus, four continents, eight countries: an interdisciplinary and international study on the psychosocial impacts of the Covid-19 pandemic among adults. Int J Environ Res Public Health 2020;17(22):8390. doi:10.3390/ijerph17228390

For peer review only - http://bmjopen.bmj.com/site/about/guidelines.xhtml

25 Chen SX, Ng JCK, Hui BPH, et al. Dual impacts of coronavirus anxiety on mental health in 35 societies. Sci Rep 2021;11(1):8925. doi:10.1038/s41598-021-87771-1

26 Cenat JM, Noorishad PG, Kokou-Kpolou CK, et al. Prevalence and correlates of depression during the COVID-19 pandemic and the major role of stigmatization in low- and middle-income countries: a multinational cross-sectional study. Psych Res 2021;297:113714. doi:10.1016/j.psychres.2021.113714

27 Tee M, Wang C, Tee C, et al. Impact of the COVID-19 pandemic on physical and mental health in lower and upper middle-income Asian countries: a comparison between the Philippines and China. Front Psychiatry 2020;11:568929. doi:10.3389/fpsyt.2020.568929

28 Fergusson D, Boden J, Horwood L, et al. Perceptions of distress and positive consequences following exposure to a major disaster amongst a well-studied cohort. Aust N Z J Psychiatry 2014;49(4):351-359. doi:10.1177/0004867414560652

29 McLeod GFH, Horwood LJ, Fergusson, DM, et al. Life-stress and reactivity by gender in a longitudinal birth cohort at 30 and 35 Years. Soc Psychiatry Psychiatr Epidemiol 2016;51(10):1385-1394. doi:10.1007/s00127-016-1254-z

30 Sulaiman-Hill R, Porter R, Tanveer S, et al. Psychosocial impacts on the Christchurch Muslim community following the 15 March terrorist attacks: a mixed-methods study protocol. BMJ Open 2021;11(10):e055413. doi:10.1136/bmjopen-2021-055413

For peer review only - http://bmjopen.bmj.com/site/about/guidelines.xhtml

31 Holmes T, Rahe R. The Social Readjustment Rating Scale. J Psychosom Res 1967;11(2):213-218. doi:10.1016/0022-3999(67)90010-4

32 Theiner G. A beginner's guide to group minds. In: Sprevak M, Kallestrup J, eds. New Waves in Philosophy of Mind. New Waves in Philosophy: London Palgrave Macmillan 2014. doi:10.1057/9781137286734_15

33 Tanveer S, Schluter PJ, Beaglehole B, et al. The COVID Psychosocial Impacts Scale: a reliable and valid tool to examine the psychosocial impacts of the covid-19 pandemic. Int J Environ Res Public Health (submitted).

34 Kessler R, Barker P, Colpe L, et al. Screening for serious mental illness in the general population. Arch Gen Psychiatry 2003;60(2):184. doi:10.1001/archpsyc.60.2.184

35 Topp C, Østergaard S, Søndergaard S, et al. The WHO-5 Well-Being Index: a systematic review of the literature. Psychother Psychosom 2015;84(3):167-176. doi:10.1159/000376585

36 Andrews G, Slade T. Interpreting scores on the Kessler Psychological Distress Scale (K10). Aust N Z J Public Health 2001;25(6):494-497. doi:10.1111/j.1467-842x.2001.tb00310.x

37 Weathers FW, Litz BT, Keane TM, et al. The PTSD Checklist for DSM-5 (PCL-5) – Standard [Measurement instrument]. The National Center for PTSD 2013. Available from https://www.ptsd.va.gov/

For peer review only - http://bmjopen.bmj.com/site/about/guidelines.xhtml

38 Tedeschi R, Cann A, Taku K, et al. The Posttraumatic Growth Inventory: a revision integrating existential and spiritual change. J Trauma Stress 2017;30(1):11-18. doi:10.1002/jts.22155

39 IBM Corp. IBM SPSS Statistics for Windows, Version 28.0. Armonk, NY: IBM Corp 2021.

40 StataCorp. Stata Statistical Software: Release 17. College Station, TX: StataCorp LLC 2021.

41 von Elm E, Altman DG, Egger M, et al. The Strengthening the Reporting of Observational Studies in Epidemiology (STROBE) statement: guidelines for reporting observational studies. Lancet 2007;370(9596):1453-7. doi:10.1016/S0140-6736(07)61602-X

42 Pye V, Taylor N, Clay-Williams R, et al. When is enough, enough? understanding and solving your sample size problems in health services research. BMC Res Notes 2016;9(1). doi:10.1186/s13104-016-1893-x

43 Ammar A, Mueller P, Trabelsi K, et al. Psychological consequences of COVID-19 home confinement: the ECLB-COVID19 multicenter study. PLoS One 2020;15(11):e0240204. doi:10.1371/journal.pone.0240204

44 TMGH-Global COVID-19 Collaborative. Perceived stress of quarantine and isolation during COVID-19 pandemic: a global survey. Front Psychiatry 2021;12:656664. doi:10.3389/fpsyt.2021.656664

For peer review only

45 Généreux M, Schluter PJ, Landaverde E, et al. The evolution in anxiety and depression with the progression of the pandemic in adult populations from eight countries and four continents. IJERPH 2021;18(9):4845. doi:10.3390/ijerph18094845

46 Schluter PJ, Généreux M, Landaverde E, Chan EYY, Hung KKC, Law R, Mok CPY, Murray V, O'Sullivan T, Qadar Z, Roy M. An eight country cross-sectional study of the psychosocial effects of COVID-19 induced quarantine and/or isolation during the pandemic. Sci Rep. 2022;12(1):13175. doi: 10.1038/s41598-022-16254-8

For peer review only

Contents

English Survey 5

Sociodemographic Information 5

Covid Psychosocial Impacts Scale 9

Personal Impacts 9

Family Impacts 10

Employment 13

Income 15

Relationships 16

Changes to Daily Routines and Behaviours 17

Exposure to Information about Covid-19 18

Personal and Family Stress 19

K-10 21

PCL-5 22

WHO-5 24

PTGI 25

International Study – Iraq (Arabic) 27

Sociodemographic Information 27

Covid Psychosocial Impacts Scale (CPIS) 31

الشخصي التأثير 31

الأسرة تأثير 32

العمل ١٩على-كوفيد جائحة تأثير 35

الدخل 37

العلاقات 38

اليومي الروتين و السلوك في تغيرات 39

١٩-كوفيد يخص فيما معلومات 41

١٩-كوفيد جائحة منذ تغيرات 41

K10 43

PCL-5 44

WHO-5 46

PGTI 47

For peer review only - http://bmjopen.bmj.com/site/about/guidelines.xhtml

International Study – Indonesia (Bahasa Indonesia) 49
Sociodemographic Information 49
Covid Psychosocial Impacts Scale 53
Dampak terhadap Pribadi 53
Dampak terhadap Keluarga 54
Pekerjaan 57
Pendapatan 59
Hubungan Keluarga 60
Perubahan dalam kegiatan dan perilaku sehari-hari 61
Paparan terhadap informasi mengenai Covid-19 63
Perubahan sejak Covid-19 63
K-10 65
PCL-5 66
WHO-5 68
PTGI 69
International Study – Malaysia (Malay) 71
Sociodemographic Information 71
Covid Psychosocail Impacts Scale 76
Kesan keatas diri sendiri 76
Kesan kepada keluarga 78
Pekerjaan 81
Pendapatan 83
Perhubungan (bersama insan lain) 84
Perubahan kepada Rutin dan Tingkah Laku Harian 85
Pendedahan kepada maklumat tentang Covid-19 87
Perubahan sejak Covid-19 87
K-10 89
PCL-5 90
WHO-5 92
PTGI 93
International Study – Somalia (Somali) 95
Sociodemographic Information 95

For peer review only - http://bmjopen.bmj.com/site/about/guidelines.xhtml

Covid Psychosocial Impacts Scale 99
Saameynta Shaqsiga 99
Sameeynta Qoyska 100
Shaqo 103
Dakhliga 105
Xiriika 106
Isbeddelka ku yimid nolol maalmeedka iyo dabeecadaha 107
Soo bandhigista macluumaadka ku saabsan Covid-19 108
Isbeddelada ku yimid Covid-19 dartiis 109
K-10 111
PCL-5 112
WHO-5 114
PTGI 115
International Study - Turkiye (Turkish) 117
Sociodemographic Information 117
Covid Psychosocial Impacts Scale 121
Kişisel etki 121
Aile etkisi 122
İş 125
Gelir 128
İlişkiler 128
Günlük rutin ve davranışlarda değişiklikler 129
Covid-19 hakkındaki bilgilere maruz kalma 131
Covid-19'dan bu yana değişiklikler 132
K-10 134
PCL-5 135
WHO-5 137
PTGI 138
International Study – Pakistan (Urdu) 140
Sociodemographic Information 140
Covid Psychosocial Impacts Scale 144
اثرات ذاتی 144

For peer review only - http://bmjopen.bmj.com/site/about/guidelines.xhtml

اثرات پر والوں خاندان 146
کام..... 148
آمدن..... 151
تعلقات 152
تبدیلیاں میں طرزعمل اور معمولات کے روزمرہ 152
معلومات میں بارے کے19 کوویڈ..... 154
تبدیلیاں والی ہونے بعد کے 19کوویڈ..... 155
K-10 157
PCL-5 158
WHO-5..... 160
PGTI 161
International Study – Iran (Persian)..... 163
Sociodemographic Information 163
Covid Psychosocial Impacts Scale 167
شخصی تاثیرات 167
خانوادگی تاثیرات 168
کووید و کار..... 171
درآمد 173
روابط..... 174
روزانه هایروال و رفتارها تغییر..... 175
۱۹-کاوید درباره اطلاعات معرض در گرفتن قرار 177
۱۹-کووید زمان از تغییرات..... 177
K-10 179
PCL-5 180
WHO-5..... 182
PTGI 183

For peer review only

English Survey

Sociodemographic Information

1. Gender

- ◯ Male
- ◯ Female
- ◯ Other
- ◯ Prefer not to say

2. Age (in years) ____________________________________

3. Marital status

- ◯ Single
- ◯ In a relationship
- ◯ Married
- ◯ Separated
- ◯ Divorced
- ◯ Widowed

4. What is your religion?

- ◯ No religion
- ◯ Islam
- ◯ Christianity
- ◯ Hinduism
- ◯ Buddhism
- ◯ Other (please specify) ____

For peer review only

5. What is your ethnic background? (You can mention more than one)

____

6. In your daily life, which languages do you speak fluently?

____

The following two questions ask specifically about your English language ability. 6a. I can understand English well

- ○ Strongly disagree 1
- ○ Disagree 2
- ○ Neither agree nor disagree 3
- ○ Agree 4
- ○ Strongly agree 5

6b. I speak English well

- ○ Not at all 1
- ○ A little 2
- ○ Somewhat 3
- ○ Quite a lot 4
- ○ A lot 5

7. What is your highest level of education?

- ○ No formal qualification (attended school, ESOL class)
- ○ Secondary school (e.g. NCEA, IB diploma, overseas school qualification)
- ○ Tertiary qualification (e.g. Certificate, Diploma or Trade qualification - less than 3 year course)
- ○ Bachelor degree (3-4 year course)
- ○ Post graduate degree (PG diploma, Master, Doctorate)

For peer review only

8. Current study or work status (Please tick whichever ones apply)

- ☐ Full-time student
- ☐ Part-time student
- ☐ Unemployed / Seeking work
- ☐ Full-time paid work
- ☐ Part-time paid work
- ☐ Full-time self employed
- ☐ Part-time self employed
- ☐ On employer paid parental leave
- ☐ Stay-at-home parent (unpaid)
- ☐ Retired
- ☐ Other (Please specify) ____

9. Occupation

____

10. Monthly household income (in local currency)

____

10a. Compared with the average income, how would you rate the income of your household?

- ○ Below average
- ○ Average
- ○ Above average

For peer review only

For peer review only - http://bmjopen.bmj.com/site/about/guidelines.xhtml

11. Are you currently in lockdown or stay-at-home order?

- ○ Yes
- ○ No

12. Have you had a Covid-19 vaccine?

- ○ Yes
- ○ No

12a. If not vaccinated, would you like to get the Covid-19 vaccine in the future?

- ○ Yes
- ○ No

13. We realise that some people may have been exposed to other traumatic incidents in the past which could make it easier or more difficult to deal with new problems.

Before the Covid-19 pandemic, have you ever witnessed or experienced any of the following: (Please tick as many as appropriate)

- ☐ Natural disaster (such as a flood, earthquake etc.)
- ☐ Living in a war zone or being exposed to military conflict
- ☐ Childhood adversity before the age of 16 (such as neglect, bullying, physical or sexual assault)
- ☐ Physical or sexual assault after the age of 16
- ☐ Serious physical accident
- ☐ Other – please specify (optional) ____
- ☐ None of the above

For peer review only

For peer review only - http://bmjopen.bmj.com/site/about/guidelines.xhtml

Covid Psychosocial Impacts Scale

Personal Impacts

For the following statements, please respond with Yes / No if this applies to you in response to Covid-19. If Yes, please indicate the amount of stress that this caused you on the scale below.

1. Do you have an underlying health condition that could make you vulnerable to Covid-19?

- No 0
- Yes No stress at all 1
- Yes A little stress 2
- Yes Moderately stressed 3
- Yes Quite a lot of stress 4
- Yes A lot of stress 5

2. Have you felt at risk of being exposed to someone with Covid-19?

- No 0
- Yes No stress at all 1
- Yes A little stress 2
- Yes Moderately stressed 3
- Yes Quite a lot of stress 4
- Yes A lot of stress 5

3. Do you think you have had Covid-19?

- No 0
- Yes No stress at all 1
- Yes A little stress 2
- Yes Moderately stressed 3

For peer review only

For peer review only - http://bmjopen.bmj.com/site/about/guidelines.xhtml

- Yes Quite a lot of stress 4
- Yes A lot of stress 5

3a. Have you had a positive Covid-19 test?

- No 0
- Yes No stress at all 1
- Yes A little stress 2
- Yes Moderately stressed 3
- Yes Quite a lot of stress 4
- Yes A lot of stress 5

3b. Do you have ongoing symptoms of Covid-19?

- No 0
- Yes No stress at all 1
- Yes A little stress 2
- Yes Moderately stressed 3
- Yes Quite a lot of stress 4
- Yes A lot of stress 5

Family Impacts

For the following statements, please respond with Yes / No if this applies to your close family members in response to Covid-19. If Yes, please indicate the amount of stress that this caused you on the scale below.

1. Does any close family member(s) have an underlying health condition that could make them vulnerable to Covid-19?

- No 0
- Yes No stress at all 1

For peer review only - http://bmjopen.bmj.com/site/about/guidelines.xhtml

- ◯ **Yes** A little stress 2
- ◯ **Yes** Moderately stressed 3
- ◯ **Yes** Quite a lot of stress 4
- ◯ **Yes** A lot of stress 5

2. Do you feel any close family member(s) is at risk of being exposed to someone with Covid-19?

- ◯ **No** 0
- ◯ **Yes** No stress at all 1
- ◯ **Yes** A little stress 2
- ◯ **Yes** Moderately stressed 3
- ◯ **Yes** Quite a lot of stress 4
- ◯ **Yes** A lot of stress 5

3. Do you think any close family member(s) have had Covid-19?

- ◯ **No** 0
- ◯ **Yes** No stress at all 1
- ◯ **Yes** A little stress 2
- ◯ **Yes** Moderately stressed 3
- ◯ **Yes** Quite a lot of stress 4
- ◯ **Yes** A lot of stress 5

3a. Has any close family member(s) tested positive for Covid-19?

- ◯ **No** 0
- ◯ **Yes** No stress at all 1
- ◯ **Yes** A little stress 2

For peer review only

For peer review only - http://bmjopen.bmj.com/site/about/guidelines.xhtml

○ **Yes** Moderately stressed 3

○ **Yes** Quite a lot of stress 4

○ **Yes** A lot of stress 5

3b. Has any close family member(s) have ongoing symptoms of Covid-19?

○ **No** 0

○ **Yes** No stress at all 1

○ **Yes** A little stress 2

○ **Yes** Moderately stressed 3

○ **Yes** Quite a lot of stress 4

○ **Yes** A lot of stress 5

3c. Has any close family member(s) passed away from Covid-19?

○ **No** 0

○ **Yes** No stress at all 1

○ **Yes** A little stress 2

○ **Yes** Moderately stressed 3

○ **Yes** Quite a lot of stress 4

○ **Yes** A lot of stress 5

4. Do you have any close family member(s) overseas who you are worried are at risk of getting Covid-19?

○ **No** 0

○ **Yes** No stress at all 1

○ **Yes** A little stress 2

○ **Yes** Moderately stressed 3

For peer review only - http://bmjopen.bmj.com/site/about/guidelines.xhtml
For peer review only

○ **Yes** Quite a lot of stress 4

○ **Yes** A lot of stress 5

5. Do you have any close family member(s) overseas who you are not able to visit or who cannot visit you?

○ **No** 0

○ **Yes** No stress at all 1

○ **Yes** A little stress 2

○ **Yes** Moderately stressed 3

○ **Yes** Quite a lot of stress 4

○ **Yes** A lot of stress 5

Employment

For the following statements please respond with Yes / No / Not applicable if this applies to you or anyone in your household in response to Covid-19. If Yes, please indicate the amount of stress that this caused you on the scale below.

1. Are you or anyone in your household considered an essential worker (e.g., in healthcare, law enforcement, emergency services, provider of essential goods/services)?

○ **No** 0

○ **Yes** No stress at all 1

○ **Yes** A little stress 2

○ **Yes** Moderately stressed 3

○ **Yes** Quite a lot stress 4

○ **Yes** A lot of stress 5

2. Have you or anyone in your household had a major change in working hours?

○ **No** 0

○ **Yes** No stress at all 1

For peer review only - http://bmjopen.bmj.com/site/about/guidelines.xhtml

- Yes A little stress 2
- Yes Moderately stressed 3
- Yes Quite a lot stress 4
- Yes A lot of stress 5

3. Have you or anyone in your household had to change the place of work to home?

- No 0
- Yes No stress at all 1
- Yes A little stress 2
- Yes Moderately stressed 3
- Yes Quite a lot stress 4
- Yes A lot of stress 5

4. Have you or anyone in your household had to change to a different type of work?

- No 0
- Yes No stress at all 1
- Yes A little stress 2
- Yes Moderately stressed 3
- Yes Quite a lot stress 4
- Yes A lot of stress 5

5. Have you or anyone in your household lost their job?

- Not applicable 9
- No 0
- Yes No stress at all 1

For peer review only

- Yes A little stress 2
- Yes Moderately stressed 3
- Yes Quite a lot stress 4
- Yes A lot of stress 5

6. Is a business you or anyone in your household owns or works in under threat of survival?

- Not applicable 9
- No 0
- Yes No stress at all 1
- Yes A little stress 2
- Yes Moderately stressed 3
- Yes Quite a lot stress 4
- Yes A lot of stress 5

Income

For the following statements please respond with Yes / No if this applies to your household in response to Covid-19. If Yes, please indicate the amount of stress that this caused you on the scale below.

1. Has your household had a major deterioration in financial circumstances?

- No 0
- Yes No stress at all 1
- Yes A little stress 2
- Yes Moderately stressed 3
- Yes Quite a lot of stress 4
- Yes A lot of stress 5

For peer review only

For peer review only - http://bmjopen.bmj.com/site/about/guidelines.xhtml

2. Has your household had a major deterioration in your ability to pay your bills or for food?

- ○ **No** 0
- ○ **Yes** No stress at all 1
- ○ **Yes** A little stress 2
- ○ **Yes** Moderately stressed 3
- ○ **Yes** Quite a lot of stress 4
- ○ **Yes** A lot of stress 5

Relationships

For the following statements please respond with Yes / No if this applies to you in response to Covid-19. If Yes, please indicate the amount of stress that this caused you on the scale below. In response to Covid-19.....

1. Has it increased difficulty and tension in the family?

- ○ **No** 0
- ○ **Yes** No stress at all 1
- ○ **Yes** A little stress 2
- ○ **Yes** Moderately stressed 3
- ○ **Yes** Quite a lot of stress 4
- ○ **Yes** A lot of stress 5

2. Has this decreased your contact with friends (physical or online)?

- ○ **No** 0
- ○ **Yes** No stress at all 1
- ○ **Yes** A little stress 2
- ○ **Yes** Moderately stressed 3
- ○ **Yes** Quite a lot of stress 4

For peer review only

◯ **Yes** A lot of stress 5

Changes to Daily Routines and Behaviours

For the following statements please respond with Yes / No / Not applicable, if this applies to you in response to Covid-19. If Yes, please indicate the amount of stress that this caused you on the scale below. In response to Covid-19.....

1. Has the Covid pandemic led to changes in your daily routine (for example, sleep, exercise, free time, pleasurable activities, or hobbies)?

◯ **No** 0

◯ **Yes** No stress at all 1

◯ **Yes** A little stress 2

◯ **Yes** Moderately stressed 3

◯ **Yes** Quite a lot of stress 4

◯ **Yes** A lot of stress 5

2. Has this caused you to delay / avoid seeking health care when you or a close family member needed it?

◯ **No** 0

◯ **Yes** No stress at all 1

◯ **Yes** A little stress 2

◯ **Yes** Moderately stressed 3

◯ **Yes** Quite a lot of stress 4

◯ **Yes** A lot of stress 5

3. Has this decreased your involvement with religious activities (in person or on-line)?

◯ **Not applicable** 9

◯ **No** 0

◯ **Yes** No stress at all 1

For peer review only

For peer review only - http://bmjopen.bmj.com/site/about/guidelines.xhtml

○ **Yes** A little stress 2

○ **Yes** Moderately stressed 3

○ **Yes** Quite a lot of stress 4

○ **Yes** A lot of stress 5

4. Has there been any other major change in your daily routines.

○ **No** 0

○ **Yes (Please specify)** 1 ____

If Yes, please indicate the amount of stress that this caused you on the scale below.

○ No stress at all 1

○ A little stress 2

○ Moderately stressed 3

○ Quite a lot of stress 4

○ A lot of stress 5

Exposure to Information about Covid-19

1. In regards to Covid-19, what is the source of information for you? (Please tick as many as appropriate.)

☐ News

☐ Facebook / Twitter

☐ Health websites

☐ Word of mouth

☐ Others (please specify) ____

For peer review only

For peer review only - http://bmjopen.bmj.com/site/about/guidelines.xhtml

2. How much stress is the news about Covid-19 causing you?

- No stress at all 1
- A little stress 2
- Moderately stressed 3
- Quite a lot of stress 4
- A lot of stress 5

Personal and Family Stress

For the following statements, please indicate on the given scale. Comparing now with before the Covid-19 pandemic...

1. How would you rate your personal level of stress?

- Considerably less stressed 1
- Somewhat less stressed 2
- About the same 3
- Somewhat more stressed 4
- Considerably more stressed 5

2. How would you rate the overall stress in your household?

- Considerably less stressed 1
- Somewhat less stressed 2
- About the same 3
- Somewhat more stressed 4
- Considerably more stressed 5

For peer review only

For peer review only - http://bmjopen.bmj.com/site/about/guidelines.xhtml

3. How would you rate your personal psychological wellbeing?

- Much better 1
- Better 2
- About the same 3
- Worse 4
- Much worse 5

4. How would you rate the overall psychological wellbeing of your household?

- Much better 1
- Better 2
- About the same 3
- Worse 4
- Much worse 5

For peer review only

For peer review only - http://bmjopen.bmj.com/site/about/guidelines.xhtml

K-10

The following ten questions ask about how you have been feeling in the last 4 weeks. For each question, select the option that best describes the amount of time you felt that way.

	None of the time 1	A little of the time 2	Some of the time 3	Most of the time 4	All of the time 5
1. In the last four weeks, about how often did you feel tired out for no good reason?	○	○	○	○	○
2. In the last four weeks, about how often did you feel nervous?	○	○	○	○	○
3. In the last four weeks, about how often did you feel so nervous that nothing could calm you down?	○	○	○	○	○
4. In the last four weeks, about how often did you feel hopeless?	○	○	○	○	○
5. In the last four weeks, about how often did you feel restless or fidgety?	○	○	○	○	○
6. In the last four weeks, about how often did you feel so restless you could not sit still?	○	○	○	○	○
7. In the last four weeks, about how often did you feel depressed?	○	○	○	○	○
8. In the last four weeks, about how often did you feel that everything was an effort?	○	○	○	○	○
9. In the last four weeks, about how often did you feel so sad that nothing could cheer you up?	○	○	○	○	○
10. In the last four weeks, about how often did you feel worthless?	○	○	○	○	○

For peer review only

For peer review only - http://bmjopen.bmj.com/site/about/guidelines.xhtml

PCL-5

Below is a list of problems which some people have experienced in response to Covid-19. Please read each problem carefully and then indicate how much you have been bothered by that problem in the past month. In the past month, how much were you bothered by:

	Not at all 0	A little bit 1	Moderately 2	Quite a bit 3	Extremely 4
1. Repeated, disturbing, and unwanted memories of the stressful experience?	○	○	○	○	○
2. Repeated, disturbing dreams of the stressful experience?	○	○	○	○	○
3. Suddenly feeling or acting as if the stressful experience were actually happening again (as if you were actually back there reliving it)?	○	○	○	○	○
4. Feeling very upset when something reminded you of the stressful experience?	○	○	○	○	○
5. Having strong physical reactions when something reminded you of the stressful experience (for example, heart pounding, trouble breathing, sweating)?	○	○	○	○	○
6. Avoiding memories, thoughts, or feelings related to the stressful experience?	○	○	○	○	○
7. Avoiding external reminders of the stressful experience (for example, people, places, conversations, activities, objects, or situations)?	○	○	○	○	○
8. Trouble remembering important parts of the stressful experience?	○	○	○	○	○
9. Having strong negative beliefs about yourself, other people, or the world (for example, having thoughts such as: I am bad, there is something seriously wrong with me, no one can be trusted, the world is completely dangerous)?	○	○	○	○	○
10. Blaming yourself or someone else for the stressful experience or what happened after it?	○	○	○	○	○
11. Having strong negative feelings such as fear, horror, anger, guilt, or shame?	○	○	○	○	○

For peer review only

For peer review only - http://bmjopen.bmj.com/site/about/guidelines.xhtml

12. Loss of interest in activities that you used to enjoy? ○ ○ ○ ○ ○

13. Feeling distant or cut off from other people? ○ ○ ○ ○ ○

14. Trouble experiencing positive feelings (for example, being unable to feel happiness or have loving feelings for people close to you)? ○ ○ ○ ○ ○

15. Irritable behaviour, angry outbursts, or acting aggressively? ○ ○ ○ ○ ○

16. Taking too many risks or doing things that could cause you harm? ○ ○ ○ ○ ○

17. Being "super alert" or watchful or on guard? ○ ○ ○ ○ ○

18. Feeling jumpy or easily startled? ○ ○ ○ ○ ○

19. Having difficulty concentrating? ○ ○ ○ ○ ○

20. Trouble falling or staying asleep? ○ ○ ○ ○ ○

For peer review only

For peer review only - http://bmjopen.bmj.com/site/about/guidelines.xhtml

WHO-5

Please indicate for each of the five statements which is closest to how you have been feeling over the last 2 weeks.

	All of the time 5	Most of the time 4	More than half of the time 3	Less than half of the time 2	Some of the time 1	At no time 0
1. I have felt cheerful and in good spirits	○	○	○	○	○	○
2. I have felt calm and relaxed	○	○	○	○	○	○
3. I have felt active and vigorous	○	○	○	○	○	○
4. I woke up feeling fresh and rested	○	○	○	○	○	○
5. My daily life has been filled with things that interest me	○	○	○	○	○	○

For peer review only

For peer review only - http://bmjopen.bmj.com/site/about/guidelines.xhtml

PTGI

Indicate for each of the statements below the degree to which this change occurred in your life as a result of Covid-19, using the following scale.

	Not at all 0	A little bit 1	Somewhat 2	Moderately 3	Quite a bit 4	A lot 5
1. I changed my priorities about what is important in life.	○	○	○	○	○	○
2. I have a greater appreciation for the value of my own life.	○	○	○	○	○	○
3. I developed new interests.	○	○	○	○	○	○
4. I have a greater feeling of self-reliance.	○	○	○	○	○	○
5. I have a better understanding of spiritual matters.	○	○	○	○	○	○
6. I more clearly see that I can count on people in times of trouble.	○	○	○	○	○	○
7. I established a new path for my life.	○	○	○	○	○	○
8. I have a great sense of closeness with others.	○	○	○	○	○	○
9. I am more willing to express my emotions.	○	○	○	○	○	○
10. I know better that I can handle difficulties.	○	○	○	○	○	○
11. I am able to do better things with my life.	○	○	○	○	○	○
12. I am better able to accept the way things work out.	○	○	○	○	○	○
13. I can better appreciate each day.	○	○	○	○	○	○
14. New opportunities are available which wouldn't have been otherwise.	○	○	○	○	○	○

For peer review only

For peer review only - http://bmjopen.bmj.com/site/about/guidelines.xhtml

15. I have more compassion for others.	◯	◯	◯	◯	◯	◯
16. I put more effort into my relationships.	◯	◯	◯	◯	◯	◯
17. I am more likely to try to change things which need changing.	◯	◯	◯	◯	◯	◯
18. I have a stronger religious faith.	◯	◯	◯	◯	◯	◯
19. I discovered that I'm stronger than I thought I was.	◯	◯	◯	◯	◯	◯
20. I learned a great deal about how wonderful people are.	◯	◯	◯	◯	◯	◯
21. I better accept needing others.	◯	◯	◯	◯	◯	◯

For peer review only

International Study – Iraq (Arabic)

Sociodemographic Information

١ـ الجنس

- ذكر
- أنثى
- اخرى
- أُفَضل عدم الإجابة

٢ـ بالسنوات (الفئة العمرية)

____

٣ـ الحالة الإجتماعية

- أعزب
- مرتبط
- متزوج
- منفصل
- مطلق
- أرمل

٤ـ ماهي ديانتك؟

- ليس لدي ديانة
- الإسلام
- المسيحية
- الهندوسية
- البوذية

For peer review only

○ أخرى (برجاء الشرح) ____

٥- ما هي أُصولك العرقية؟ (يمكن أن تذكر أكثر من واحدة)

____

٦- ماهي اللغات التي تتحدثها بطلاقة في حياتك اليومية؟

____

السؤالان التاليان يحددان مدى قدرتك في اللغة الإنجليزية.

٦أ- يمكنني فهم اللغة الإنجليزية جيداً

○ لا أوافق بشدة 1

○ لا أوافق 2

○ لا اعلم 3

○ أوافق 4

○ أوافق بشدة 5

٦ب- يمكنني تحدث الإنجليزية جيداً

○ لا على الإطلاق 1

○ قليلاً 2

○ بقدر معتدل 3

○ كثيراً 4

○ كثيراً جداً 5

٧- ما هي مؤهلاتك العلمية؟

○ لا يوجد مؤهل علمي (التحقت بالمدرسة، فصل تعليم الكبار غير الناطقين بالإنجليزية)

○ المرحلة الثانوية (على سبيل المثال، الشهادة الوطنية النيوزيلاندية للتحصيل التعليمي، دبلوم، والمؤهلات المدرسية من خارج نيوزيلاندا)

For peer review only - http://bmjopen.bmj.com/site/about/guidelines.xhtml

○ مؤهل جامعي (على سبيل المثال، شهادة، دبلوم أو مؤهل تجاري -دورة أقل من 3 سنوات)

○ البكالوريوس (دورة 4-3 سنوات)

○ الدراسات العليا (دبلوم الدراسات العليا، الماجستير، الدكتوراة)

٨ـ وضعك الدراسي أو الوظيفي (يرجى وضع علامة على كل الحالات التي تنطبق عليك)

- ☐ يدرس بدوام كامل
- ☐ يدرس بدوام جزئي
- ☐ لا يعمل / يبحث عن عمل
- ☐ يعمل بدوام كامل (وظيفة واحدة او أكثر)
- ☐ يعمل بدوام جزئي (وظيفة واحدة او أكثر)
- ☐ يعمل لحسابة بدوام كامل
- ☐ يعمل لحسابة بدوام جزئي
- ☐ أجازة امومة مدفوعة الأجر
- ☐ رب - ربة منزل (غير مدفوع الأجر)
- ☐ متقاعد
- ☐ أخرى (برجاء الشرح) ____

٩ـ الوظيفة

____

١٠ـ دخل الأسرة الشهري (بالعملة المحلية)

____

١٠أـ مقارنة بمتوسط الدخل، ما هو تقيمك لدخل أسرتك؟

○ أقل من المتوسط

○ متوسط

○ أعلى من المتوسط

١١ـ هل أنت حالياً في وضع العزل بسبب جائحة كوفيد-١٩؟

○ نعم

○ لا

For peer review only - http://bmjopen.bmj.com/site/about/guidelines.xhtml

١٢- هل تلقيت مطعوم كوفيد-١٩؟

- ○ نعم
- ○ لا

١٢أ- إذا لم تتلقى مطعوم كوفيد-١٩، هل ترغب في الحصول عليه في المستقبل؟

- ○ نعم
- ○ لا

١٣- ندرك أن بعض الأشخاص قد تعرضوا إلى أحداث صادمة في الماضي قد تجعل التعامل مع المشكلات الأخرى أسهل أو أصعب في التعامل مع اي مشكلات جديدة. قبل جائحة كوفيد-١٩ هل سبق أن شاهدت أو تعرضت إلى واحدة أو أكثر من هذه المواقف :(يرجى وضع علامة أو اكثر إن وجد)

- ☐ كوارث طبيعية (مثل فيضان، زلزال أو ما إلى ذلك)
- ☐ كنت تعيش في منطقة حروب أو تعرضت إلى نزاع عسكري
- ☐ محنة في الطفولة قبل سن ١٦عاماً (مثل الإهمال، التنمرأو الإعتداء الجنسي)
- ☐ اعتداء بدني أو جنسي بعد سن ١٦عاماً
- ☐ حادث بدني خطير
- ☐ أخرى - برجاء الشرح (أختياري) ____
- ☐ ليس مما سبق

For peer review only

For peer review only - http://bmjopen.bmj.com/site/about/guidelines.xhtml

Covid Psychosocial Impacts Scale (CPIS)

التأثير الشخصي

يرجى الإجابة (بنعم / لا) إذا كان لجائحة كوفيد-١٩ أثرعليك من خلال الإسئلة التالية. إذا كانت اجابتك بنعم، يرجى تحديد مقدار الضغط الذي تشعر به من خلال هذا المقياس أدناه.

١- هل لديك اي مشاكل صحية تجعلك عرضة لمرض كوفيد-١٩؟

- لا 0
- نعم لا يوجد ضغط اطلاقاً 1
- نعم قليلاً 2
- نعم احيانا 3
- نعم غالباً جداً 4
- نعم كثيراً جداً 5

٢- هل شعرت بخطر التعرض لشخص مصاب بكوفيد-١٩؟

- لا 0
- نعم لا يوجد ضغط اطلاقاً 1
- نعم قليلاً 2
- نعم احيانا 3
- نعم غالباً جداً 4
- نعم كثيراً جداً 5

٣- هل أُصبت بمرض كوفيد (كورونا)؟

- لا 0
- نعم لا يوجد ضغط اطلاقاً 1
- نعم قليلاً 2
- نعم احيانا 3

For peer review only - http://bmjopen.bmj.com/site/about/guidelines.xhtml

نعم غالباً جداً 4

نعم كثيراً جداً 5

٣أ- هل كانت نتيجة فحص أختبار كوفيد-١٩ إيجابية؟

لا 0

نعم لا يوجد ضغط اطلاقاً 1

نعم قليلاً 2

نعم احيانا 3

نعم غالباً جداً 4

نعم كثيراً جداً 5

٣ب -هل تعاني من أعراض مستمرة بسبب كوفيد-١٩؟

لا 0

نعم لا يوجد ضغط اطلاقاً 1

نعم قليلاً 2

نعم احيانا 3

نعم غالباً جداً 4

نعم كثيراً جداً 5

For peer review only

تأثير الأسرة

يرجى الإجابة (بنعم / لا) إذا كان لجائحة كوفيد-١٩ أثرعلى أسرتك من خلال الإسئلة التالية. إذا كانت اجابتك بنعم، يرجى تحديد مقدار الضغط الذي تشعر به من خلال هذا المقياس أدناه.

١- هل يعاني احد أفراد أسرتك من اي مشاكل صحية تجعله عرضة لمرض كوفيد-١٩؟

لا 0

نعم لا يوجد ضغط اطلاقاً 1

- نعم قليلاً 2
- نعم احيانا 3
- نعم غالباً جداً 4
- نعم كثيراً جداً 5

٢- هل تشعر ان احد أفراد أسرتك معرض للاصابة لكوفيد-١٩ من خلال الإختلاط بأحد أفراد العمل؟

- لا 0
- نعم لا يوجد ضغط اطلاقاً 1
- نعم قليلاً 2
- نعم احيانا 3
- نعم غالباً جداً 4
- نعم كثيراً جداً 5

٣- هل أُصيب احد أفراد أسرتك بمرض كوفيد-١٩؟

- لا 0
- نعم لا يوجد ضغط اطلاقاً 1
- نعم قليلاً 2
- نعم احيانا 3
- نعم غالباً جداً 4
- نعم كثيراً جداً 5

٣أ- هل كانت نتيجة فحص كوفيد-١٩ إيجابية لأحد أفراد أسرتك؟

- لا 0
- نعم لا يوجد ضغط اطلاقاً 1

For peer review only - http://bmjopen.bmj.com/site/about/guidelines.xhtml

نعم قليلاً 2

نعم احيانا 3

نعم غالباً جداً 4

نعم كثيراً جداً 5

٣ب ـ هل أحد أفراد أسرتك مازال يعاني من أعراض كوفيد-١٩؟

لا 0

نعم لا يوجد ضغط اطلاقاً 1

نعم قليلاً 2

نعم احيانا 3

نعم غالباً جداً 4

نعم كثيراً جداً 5

٣ ج - هل توفى احد أفراد أسرتك كنتيجة لكوفيد-١٩؟

لا 0

نعم لا يوجد ضغط اطلاقاً 1

نعم قليلاً 2

نعم احيانا 3

نعم غالباً جداً 4

نعم كثيراً جداً 5

٤- هل لديك احد من أفراد أسرتك مقيم بالخارج و تشعر بالقلق عليهم من خطر الإصابة بمرض كوفيد-١٩؟

لا 0

For peer review only - http://bmjopen.bmj.com/site/about/guidelines.xhtml

- نعم لا يوجد ضغط اطلاقاً 1
- نعم قليلاً 2
- نعم احيانا 3
- نعم غالباً جداً 4
- نعم كثيراً جداً 5

٥- هل لديك احد من أفراد أسرتك يقيم بالخارج و لا يمكنك زيارتهم و لا يمكنهم زيارتك؟

- لا 0
- نعم لا يوجد ضغط اطلاقاً 1
- نعم قليلاً 2
- نعم احيانا 3
- نعم غالباً جداً 4
- نعم كثيراً جداً 5

تأثير جائحة كوفيد-١٩ على العمل

يرجى الإجابة (بنعم / لا / لا أفضل الإجابة) إذا كان لجائحة كوفيد-١٩ أثرعليك او على احد أفراد أسرتك المقيمين معك بالمنزل من خلال الإسئلة التالية. إذا كانت اجابتك بنعم، يرجى تحديد مقدار الضغط الذي تشعر به من خلال هذا المقياس أدناه.

١- هل تعتبر أنت أو احد أفراد أسرتك المقيمين معك بالمنزل من العمال الأساسيين (مثل عمال الرعاية الصحية، شؤون القانون، خدمات الطوارئ أو مزودي السلع و الخدمات الأساسية)؟

- لا 0
- نعم لا يوجد ضغط اطلاقاً 1
- نعم قليلاً 2
- نعم احيانا 3
- نعم غالباً جداً 4

For peer review only - http://bmjopen.bmj.com/site/about/guidelines.xhtml

نعم كثيراً جداً 5

٢- هل كان هناك تغير كبير في ساعات العمل لك أو احد أفراد أسرتك المقيمين معك بالمنزل؟

- لا 0
- نعم لا يوجد ضغط اطلاقاً 1
- نعم قليلاً 2
- نعم احيانا 3
- نعم غالباً جداً 4
- نعم كثيراً جداً 5

٣- هل اضطررت أنت أو احد أفراد أسرتك المقيمين معك بالمنزل إلى تغيير مكان العمل للعمل من المنزل؟

- لا 0
- نعم لا يوجد ضغط اطلاقاً 1
- نعم قليلاً 2
- نعم احيانا 3
- نعم غالباً جداً 4
- نعم كثيراً جداً 5

٤- هل اضطررت أنت أو احد أفراد أسرتك المقيمين معك بالمنزل إلى تغيير طبيعة العمل؟

- لا 0
- نعم لا يوجد ضغط اطلاقاً 1
- نعم قليلاً 2
- نعم احيانا 3
- نعم غالباً جداً 4

For peer review only - http://bmjopen.bmj.com/site/about/guidelines.xhtml

نعم كثيراً جداً 5

٥ -هل فقدت أنت او احد أفراد أسرتك المقيمين معك بالمنزل؟

لا أُفضل الإجابة

لا 0

نعم لا يوجد ضغط اطلاقاً 1

نعم قليلاً 2

نعم احيانا 3

نعم غالباً جداً 4

نعم كثيراً جداً 5

٦- هل الأعمال التجارية التي تمتلكها او تعمل بها أنت او احد أفراد أسرتك المقيمين معك بالمنزل أصبحت مهددة بالبقاء؟

لا أُفضل الإجابة

لا 0

نعم لا يوجد ضغط اطلاقاً 1

نعم قليلاً 2

نعم احيانا 3

نعم غالباً جداً 4

نعم كثيراً جداً 5

الدخل

يرجى الإجابة (بنعم / لا) إذا حدث ذلك لاحد أفراد أسرتك المقيمين معك بالمنزل كنتيجة لجائحة كوفيد-١٩ من خلال الإسئلة التالية. إذا كانت اجابتك بنعم ، فيرجى تحديد مقدار الضغط الذي تشعر به من خلال هذا المقياس أدناه.

١- هل تعرض أحد من أفراد أسرتك لتدهور كبير في الحالة المالية؟

لا 0

For peer review only - http://bmjopen.bmj.com/site/about/guidelines.xhtml

- ○ نعم لا يوجد ضغط اطلاقاً 1
- ○ نعم قليلاً 2
- ○ نعم احيانا 3
- ○ نعم غالباً جداً 4
- ○ نعم كثيراً جداً 5

٢- هل تعرض احد أفراد أسرتك المقيمين لتدهور كبير في عدم القدرة على دفع الفواتير او توفير الإحتياجات الغذائية؟

- ○ لا 0
- ○ نعم لا يوجد ضغط اطلاقاً 1
- ○ نعم قليلاً 2
- ○ نعم احيانا 3
- ○ نعم غالباً جداً 4
- ○ نعم كثيراً جداً 5

العلاقات

يرجى الإجابة (بنعم / لا) إذا كان لجائحة كوفيد-١٩ أثرعليك من خلال الإسئلة التالية.
إذا كانت اجابتك بنعم، يرجى تحديد مقدار الضغط الذي تشعر به من خلال هذا المقياس أدناه. كنتيجة لكوفيد-١٩....
١- هل ادى ذلك لزيادة المشاكل والتوتر داخل العائلة؟

- ○ لا 0
- ○ نعم لا يوجد ضغط اطلاقاً 1
- ○ نعم قليلاً 2
- ○ نعم احيانا 3
- ○ نعم غالباً جداً 4
- ○ نعم كثيراً جداً 5

For peer review only

For peer review only - http://bmjopen.bmj.com/site/about/guidelines.xhtml

٢- هل ادى ذلك الى انخفاض معدل تواصلك مع أصدقائك سواء تواصل اجتماعياً او من خلال التواصل الإليكتروني؟

- لا 0
- نعم لا يوجد ضغط اطلاقاً 1
- نعم قليلاً 2
- نعم احيانا 3
- نعم غالباً جداً 4
- نعم كثيراً جداً 5

تغيرات في السلوك و الروتين اليومي

يرجى الإجابة (نعم / لا / لا أُفضل الإجابة) إذا كان لجائحة كوفيد-١٩ أثرعليك من خلال الإسئلة التالية. إذا كانت اجابتك بنعم، يرجى تحديد مقدار الضغط الذي تشعر به من خلال هذا المقياس أدناه.

١- هل أدت جائحة كوفيد إلى تغير معدل أنشطتك اليومية (على سبيل المثال :معدل النوم، الأنشطة الرياضية، وقت الفراغ أو استمتاعك بالأنشطة أو الهوايات الخاص بك)؟

- لا 0
- نعم لا يوجد ضغط اطلاقاً 1
- نعم قليلاً 2
- نعم احيانا 3
- نعم غالباً جداً 4
- نعم كثيراً جداً 5

٢- هل ادى ذلك الى تأخير او تجنب طلب الرعاية الصحية عندما تحتاجها أنت أو أحد أفراد أسرتك؟

- لا 0
- نعم لا يوجد ضغط اطلاقاً 1
- نعم قليلاً 2
- نعم احيانا 3

For peer review only - http://bmjopen.bmj.com/site/about/guidelines.xhtml

- نعم غالباً جداً 4
- نعم كثيراً جداً 5

٣- هل ادى ذلك الى انخفاض معدل مشاركتك في الأنشطة الدينية (مثل سواء وجهاً لوجه أو عبر الإنترنت)؟

- لا أُفضل الإجابة
- لا 0
- نعم لا يوجد ضغط اطلاقاً 1
- نعم قليلاً 2
- نعم احيانا 3
- نعم غالباً جداً 4
- نعم كثيراً جداً 5

٤- هل لديك اى تغيرات كبيرة اخرى في حياتك الروتينية؟

- لا 0
- نعم (برجاء الشرح) 1 ______________________

إذا كانت اجابتك بنعم، يرجى تحديد مقدار الضغط الذي تشعر به من خلال هذا المقياس أدناه.

- لا يوجد ضغط اطلاقاً 1
- قليلاً 2
- احيانا 3
- غالباً جداً 4
- كثيراً جداً 5

For peer review only

For peer review only - http://bmjopen.bmj.com/site/about/guidelines.xhtml

معلومات فيما يخص كوفيد-١٩

١- فيما يخص كوفيد-١٩ ماهو مصدر المعلومات بالنسبة لك؟ (برجاء وضع علامة على أكثر من إختيار.)

- ☐ الأخبار
- ☐ الفيس بوك / تويتر
- ☐ المواقع الصحية المعتمدة
- ☐ كلام شَفهي
- ☐ أخرى (برجاء الشرح) ______________________

٢- ما مدى الضغط النفسي الذي تسببته الأخبار عن كوفيد-١٩؟

- ○ نعم لا يوجد ضغط اطلاقاً 1
- ○ نعم قليلاً 2
- ○ نعم احيانا 3
- ○ نعم غالباً جداً 4
- ○ نعم كثيراً جداً 5

تغيرات منذ جائحة كوفيد-١٩

من خلال الأسئلة التالية، يرجى الإشارة إلى مقدار الضغط النفسي الذي تأثرت به. مقارنة بين الوضع الحالي و الوضع قبل جائحة كوفيد-١٩....

١- كيف تقيم مستوى ضغطك الشخصي؟

- ○ ضغط قليل جداً 1
- ○ ضغط الى حداً ما 2
- ○ بقى كما هو 3
- ○ ضغط أكثر 4
- ○ ضغط كثير جداً 5

٢- كيف تقيم مستوى الضغط العام في منزلك؟

- ○ ضغط قليل جداً 1
- ○ ضغط الى حداً ما 2

For peer review only - http://bmjopen.bmj.com/site/about/guidelines.xhtml
For peer review only

بقى كما هو 3

ضغط أكثر 4

ضغط كثير جداً 5

٣ـ كيف تقيم صحتك النفسية الشخصية؟

أفضل بكثير 1

أفضل 2

بقى كما هو 3

أسوء 4

أسوء بكثير 5

٤ـ كيف تُقيم مقدار الصحة النفسية لأفراد أسرتك بشكل عام؟

أفضل بكثير 1

أفضل 2

بقى كما هو 3

أسوء 4

أسوء بكثير 5

For peer review only

K10

الأسئلة العشر التالية سوف يتم سؤالك عن ما كنت تشعر به خلال الأربع أسابيع الماضية. يرجى قراءة الأسئلة التالية و اختيار الإجابة الأقرب التي تمثل وضعك الحالي.

	إطلاقاً 1	قليلاً 2	أحياناً 3	غالباً 4	دائماً 5
١ـ خلال الأربع أسابيع الماضية، إلى أي مدى كنت تشعرأنك مجهد بدون أي سبب واضح؟	○	○	○	○	○
٢ـ خلال الأربع أسابيع الماضية، إلى أي مدى كنت تشعر بالعصبية؟	○	○	○	○	○
٣ـ خلال الأربع أسابيع الماضية، إلى أي مدى كنت تشعر بالعصبية إلى حد أن لاشيء يمكنه أن يجعلك تهدئ؟	○	○	○	○	○
٤ـ خلال الأربع أسابيع الماضية، إلى أي مدى كنت تشعر باليأس؟	○	○	○	○	○
٥ـ خلال الأربع أسابيع الماضية، إلى أي مدى كنت تشعر بعدم الراحة أو القلق؟	○	○	○	○	○
٦ـ خلال الأربع أسابيع الماضية، إلى أي مدى كنت تشعر بعدم الراحة لدرجة أنه لايمكنك الجلوس هادئاً؟	○	○	○	○	○
٧ـ خلال الأربع أسابيع الماضية، إلى أي مدى كنت تشعر بالإكتئاب؟	○	○	○	○	○
٨ـ خلال الأربع أسابيع الماضية، إلى أي مدى كنت تشعر إنك تبذل مجهوداً كبيراً عندما تفعل أي شيء؟	○	○	○	○	○
٩ـ خلال الأربع أسابيع الماضية، إلى أي مدى كنت تشعر بالحزن لدرجة إن لاشيء يحسن من معنوياتك؟	○	○	○	○	○
١٠ـ خلال الأربع أسابيع الماضية، إلى أي مدى كنت تشعر بأن حياتك ليست لها قيمة أو معنى؟	○	○	○	○	○

For peer review only

PCL-5

فيما يلي قائمة بالمشاكل التى قد يعاني منها بعض الناس كأستجابة لكوفيد-١٩. الرجاء قراءة كل مشكلة بعناية ثم توضيح مدى إنزعاجك لهذه المشكلة خلال الشهر الماضي. خلال الشهر الماضي ما مدى إنزعاجك مما يلي:

	أطلاقاً 0	قليلاً 1	متوسط 2	غالباً 3	كثيراً جداً 4
١ـ ذكريات متكررة، مزعجة و غير مرغوب فيها نتيجة التجربة المؤلمة التي مررت بها؟	○	○	○	○	○
٢ـ أحلام متكررة، مزعجة نتيجة التجربة المؤلمة التي مررت بها؟	○	○	○	○	○
٣ -الشعور أو التصرف المفاجىء كما لو أن التجربة المؤلمة تحدث مرة آخرى (كأنك تعيش الحادث من جديد)؟	○	○	○	○	○
٤ـ الشعور بالإستياء الشديد عندما يذكرك شيء ما بالتجربة المؤلمة؟	○	○	○	○	○
٥ـ تعرضك لردة فعل جسدية قوية عند حدوث شيء يذكرك بالتجربة المؤلمة (مثل خفقان بالقلب، صعوبة في التنفس أو التعرق)؟	○	○	○	○	○
٦ـ تجنب الذكريات، الأفكار أو المشاعر المتعلقة بالتجربة المؤلمة؟	○	○	○	○	○
٧ـ تتجنب الأشياء الخارجية التي تذكرك بالتجربة المؤلمة (مثل الأشخاص، الأماكن، المحادثات، الأنشطة، الأشياء أو المواقف)؟	○	○	○	○	○
٨ـ تعاني من مشاكل تذكرك بأجزاء مهمة متعلقة بالتجربة المؤلمة؟	○	○	○	○	○
٩ـ أصبح لديك معتقدات سلبية قوية متعلقة بك أو عن الأشخاص الأخرين أو العالم الخارجي (مثل لديك أفكار مثل :أنا سيء، هناك شيء خاطىء فيّ، لا يمكن الوثوق بأحد أو العالم بأكمله)؟	○	○	○	○	○
١٠ـ إلقاء اللوم على نفسك أو على شخص آخر فيما يخص حدوث التجربة المؤلمة أوتبعاتها؟	○	○	○	○	○
١١ـ وجود مشاعر سلبية قوية مثل الخوف، الرعب، الغضب، الشعور بالذنب أو العار؟	○	○	○	○	○
١٢ـ فقدان الإهتمام بالأنشطة التي كنت تستمتع بها؟	○	○	○	○	○

For peer review only

١٣ـ الشعور بالبعد أو العزلة عن الأشخاص الأخرين؟

١٤ـ صعوبة الإحساس بالمشاعر الإيجابية (مثل عدم التمكن من الشعور بالسعادة أو مشاعر حب تجاه المقربين إليك)؟

١٥ـ سرعة الغضب أو المرور بنوبات الغضب أو التصرف بعدوانية؟

١٦ -الخوض في الكثير من المخاطر أو الأشياء التي قد تسبب لك الضرر؟

١٧ـ البقاء في حالة تأهب قصوى، إحتراس أو حذر شديدين؟

١٨ـ شعور متقلب أو الإستفزاز بسهولة؟

١٩ـ صعوبة في التركيز؟

٢٠ـ مشاكل في الرغبة في النوم أو في البقاء نائماً؟

For peer review only

WHO-5

يرجى وضع إشارة عند كل من الأسئلة الخمس التالية، حيث تمثل الإجابة شعورك خلال الإسبوعين الماضيين.

	دائماً 5	أكثر الأحيان 4	أكثر من نصف الوقت 3	أقل من نصف الوقت 2	بعض الوقت 1	بتاتاً 0
١- كنت سعيداً و بمزاجٍ جيد.	○	○	○	○	○	○
٢- كنت أشعُر بالهدوء و الإسترخاء.	○	○	○	○	○	○
٣- كنت أشعر بالحيوية و النشاط.	○	○	○	○	○	○
٤- كنت أستيقظ نشطاً و مرتاحاً.	○	○	○	○	○	○
٥- كانت أيامي مليئةً بأشياء محببة لنفسي.	○	○	○	○	○	○

For peer review only

For peer review only - http://bmjopen.bmj.com/site/about/guidelines.xhtml

PGTI

من خلال الأسئلة التالية يرجى تحديد الدرجة المناسبة لمدى التغيير الذي احدثته جائحة كوفيد-١٩ على حياتك.

	**لا على الإطلاق 0**	**قليلاً 1**	**إلى حد ما 2**	**بشكلٍ معتدل 3**	**إلى حد قليل 4**	**كثيراً 5**
١- قمت بتغيير أولوياتي عما هو مهم في الحياة.	○	○	○	○	○	○
٢- أصبح لديّ تقديراً أكبر لقيمة حياتي.	○	○	○	○	○	○
٣- طوّرت اهتمامات جديدة.	○	○	○	○	○	○
٤- لديّ إحساس أكبر بالإعتماد على الذات.	○	○	○	○	○	○
٥- لديّ فهمًا أفضل للمسائل الروحية.	○	○	○	○	○	○
٦- أرى بوضوح أكثر أنه يمكنني الإعتماد على الأشخاص في أوقات المشاكل.	○	○	○	○	○	○
٧- أنشأت مساراً جديداً لحياتي.	○	○	○	○	○	○
٨- أصبح لديّ إحساس كبير بالقرب من الآخرين.	○	○	○	○	○	○
٩- أصبحت أكثر استعداداً للتعبير عن مشاعري.	○	○	○	○	○	○
١٠- أستطيع التعامل مع الصعوبات.	○	○	○	○	○	○
١١- أستطيع القيام بأشياء أفضل في حياتي.	○	○	○	○	○	○
١٢- أصبحت أفضل تقبلاً للطرق التي تحل بها الأمور.	○	○	○	○	○	○
١٣- يمكنني تقدير كل يوم بشكلٍ أفضل.	○	○	○	○	○	○

For peer review only

○	○	○	○	○	○	١٤- تتوفر فرص جديدة لم تكن ستتوفر بطريقة أخرى.
○	○	○	○	○	○	١٥- أشعر بمزيد من التعاطف تجاه الآخرين.
○	○	○	○	○	○	١٦- أضع مزيداً من الجهد في علاقاتي.
○	○	○	○	○	○	١٧- احاول جاهداً تغيير الأشياء التي تحتاج إلى التغيير.
○	○	○	○	○	○	١٨- إزدادت قوة إيماني.
○	○	○	○	○	○	١٩- اكتشفت أنني أقوى مما كنت أتصور.
○	○	○	○	○	○	٢٠- تعلمت قدراً كبيراً عن مدى حسن خُلق الأشخاص.
○	○	○	○	○	○	٢١- أتقبل بشكلٍ أفضل احتياجي للآخرين .

For peer review only

International Study – Indonesia (Bahasa Indonesia)

Sociodemographic Information

1. Jenis Kelamin

- ◯ Laki-laki
- ◯ Perempuan
- ◯ Lainnya
- ◯ Memilih untuk tidak menjawab

2. Umur (tahun)

____

3. Status pernikahan

- ◯ Belum menikah
- ◯ Sedang memiliki hubungan
- ◯ Menikah
- ◯ Berpisah
- ◯ Cerai hidup
- ◯ Cerai mati

4. Apakah agama yang Anda anut sekarang?

- ◯ Tidak beragama
- ◯ Islam
- ◯ Kristen/Katolik
- ◯ Hindu
- ◯ Buddha

For peer review only

- ◯ Lain-lain (Sebutkan) ____

5. Apa latar belakang etnisitas/suku bangsa yang Anda miliki? (Sebutkan dan Anda boleh menyebutkan lebih dari satu etnis/suku bangsa)

____

6. Pendidikan tertinggi yang ditamatkan?

- ◯ Pendidikan non formal (homeschooling, les privat/les Bersama, pelatihan)
- ◯ SMA/SMK dan sederajat
- ◯ Perguruan Tinggi D3
- ◯ Perguruan Tinggi S1/D4
- ◯ Perguruan Tinggi S2/S3

7. Status pekerjaan dan pendidikan yang saat ini dilakukan (Beri tanda pada pilihan Anda)

- ☐ Belajar penuh waktu
- ☐ Belajar paruh waktu
- ☐ Tidak bekerja
- ☐ Bekerja penuh waktu (karyawan/pegawai)
- ☐ Bekerja paruh waktu (karyawan/pegawai)
- ☐ Bekerja berusaha sendiri/wiraswasta penuh waktu (Full-time self employed)
- ☐ Bekerja berusaha sendiri/wiraswasta paruh waktu (Part-time self employed)
- ☐ Cuti diluar tanggungan negara (jika Anda berstatus ASN ataupun tugas belajar)
- ☐ Bekerja di rumah, tanpa menerima gaji (misal: ibu rumah tangga)
- ☐ Pensiun/Purna Tugas

For peer review only

For peer review only - http://bmjopen.bmj.com/site/about/guidelines.xhtml

☐ Lainnya (Sebutkan) ____

8. Pekerjaan (jika saat ini sedang melakukan kerja penuh waktu dan atau kerja paruh waktu)

____

9. Berapakah pendapatan dan penghasilan Anda (termasuk suami/istri, jika menikah) dalam 1 bulan (per bulan-Rp)

____

9a. Dibandingkan dengan pendapatan rata-rata nasional penduduk Indonesia tahun 2020 berdasarkan data BPS Februari, 2020 (Rp 1,6 juta per bulan), maka bagaimana Anda menilai posisi pendapatan keluarga Anda per bulan?

○ Dibawah rata-rata

○ Rata-rata

○ Diatas rata-rata

10. Apakah saat ini berada pada lingkungan yang melakukan Pemberlakukan Pembatasan Kegiatan Masyarakat (PPKM)?

○ Iya

○ Tidak

11. Apakah Anda sudah mendapatkan vaksin Covid-19?

○ Iya

○ Tidak

11a. Jika belum mendapatkan vaksin, apakah Anda bersedia untuk divaksinasi di kemudian hari?

○ Iya

○ Tidak

For peer review only - http://bmjopen.bmj.com/site/about/guidelines.xhtml

12. Kami menyadari bahwa sebagian orang memiliki pengalaman traumatik di masa lalu yang membuat seseorang akan lebih mudah atau sulit untuk menjalani kehidupan, menghadapi tantangan baru maupun mengatasi permasalahan baru yang muncul di kemudian hari.

Sebelum wabah Covid-19, apakah Anda pernah mengalami ataupun menyaksikan beberapa kejadian seperti di bawah ini: (Beri tanda sebanyak mungkin sesuai yang Anda alami)

☐ Bencana alam (banjir, gempa bumi, tsunami, tanah longsor dsb)

☐ Hidup di zona konflik atau peperangan ataupun daerah darurat militer

☐ Pengalaman tidak menyenangkan ketika masa kecil sebelum usia 15 tahun (diabaikan, ditelantarkan orang tua, dibuli, atau kekerasan fisik-mental/seksual)

☐ Kekerasan fisik-mental/seksual Ketika usia diatas 16 tahun

☐ Kecelakaan fisik yang serius

☐ Lainnya, sebutkan pengalaman lainnya yang menyebabkan traumatik di masa lalu
____

☐ Tidak pernah mengalami pengalaman traumatik di masa lalu

For peer review only

Covid Psychosocial Impacts Scale

Dampak terhadap Pribadi

Berikan respon Ya / Tidak terhadap pertanyaaan di bawah ini, jika hal berikut dialami oleh Anda terkait dengan wabah Covid-19. Jika jawaban anda Ya, mohon berikan indikasi penilaian tekanan (stress) yang dialami seusai dengan skala berikut.

1. Apakah Anda mempunyai masalah kesehatan yang membuat Anda rentan/beresiko terhadap Covid-19?

- ◯ **Tidak 0**
- ◯ **Ya** Tidak membuat stress 1
- ◯ **Ya** Sedikit membuat stress 2
- ◯ **Ya** Cukup membuat stress 3
- ◯ **Ya** Banyak membuat stress 4
- ◯ **Ya** Sangat membuat stress 5

2. Apakah Anda merasa berisiko terpapar dengan orang yang terkena Covid-19?

- ◯ **Tidak 0**
- ◯ **Ya** Tidak membuat stress 1
- ◯ **Ya** Sedikit membuat stress 2
- ◯ **Ya** Cukup membuat stress 3
- ◯ **Ya** Banyak membuat stress 4
- ◯ **Ya** Sangat membuat stress 5

3. Apakah Anda merasa pernah terkena Covid-19?

- ◯ **Tidak 0**
- ◯ **Ya** Tidak membuat stress 1
- ◯ **Ya** Sedikit membuat stress 2
- ◯ **Ya** Cukup membuat stress 3

For peer review only - http://bmjopen.bmj.com/site/about/guidelines.xhtml

For peer review only

- **Ya** Banyak membuat stress 4
- **Ya** Sangat membuat stress 5

3a. Apakah Anda pernah mendapatkan hasil positif dari tes Covid-19?

- **Tidak 0**
- **Ya** Tidak membuat stress 1
- **Ya** Sedikit membuat stress 2
- **Ya** Cukup membuat stress 3
- **Ya** Banyak membuat stress 4
- **Ya** Sangat membuat stress 5

3b. Apakah saat ini Anda merasa mengalami gejala Covid-19?

- **Tidak 0**
- **Ya** Tidak membuat stress 1
- **Ya** Sedikit membuat stress 2
- **Ya** Cukup membuat stress 3
- **Ya** Banyak membuat stress 4
- **Ya** Sangat membuat stress 5

Dampak terhadap Keluarga

Berikan respon Ya/Tidak terhadap pertanyaan di bawah ini, jika hal berikut dialami oleh anggota keluarga dekat Anda terkait dengan wabah Covid-19. Jika jawaban anda Ya, mohon berikan indikasi penilaian tekanan (stress) yang dialami sesuai dengan skala berikut.

1. Apakah **keluarga dekat Anda mempunyai masalah** kesehatan **yang membuat mereka rentan/berisiko terhadap Covid-19?**

- **Tidak 0**

For peer review only - http://bmjopen.bmj.com/site/about/guidelines.xhtml

- **Ya** Tidak membuat stress 1
- **Ya** Sedikit membuat stress 2
- **Ya** Cukup membuat stress 3
- **Ya** Banyak membuat stress 4
- **Ya** Sangat membuat stress 5

2. Apakah Anda merasa anggota keluarga Anda berisiko berhadapan dengan orang yang terkena Covid-19?

- **Tidak** 0
- **Ya** Tidak membuat stress 1
- **Ya** Sedikit membuat stress 2
- **Ya** Cukup membuat stress 3
- **Ya** Banyak membuat stress 4
- **Ya** Sangat membuat stress 5

3. Apakah Anda merasa ada anggota keluarga terdekat yang terkena Covid-19?

- **Tidak** 0
- **Ya** Tidak membuat stress 1
- **Ya** Sedikit membuat stress 2
- **Ya** Cukup membuat stress 3
- **Ya** Banyak membuat stress 4
- **Ya** Sangat membuat stress 5

3a. Apakah ada anggota keluarga terdekat anda yang dites hasilnya positif Covid-19?

- **Tidak** 0

For peer review only

For peer review only - http://bmjopen.bmj.com/site/about/guidelines.xhtml

◯ **Ya** Tidak membuat stress 1

◯ **Ya** Sedikit membuat stress 2

◯ **Ya** Cukup membuat stress 3

◯ **Ya** Banyak membuat stress 4

◯ **Ya** Sangat membuat stress 5

3b. Apakah ada anggota keluarga terdekat anda mengalami gejala Covid-19?

◯ **Tidak** 0

◯ **Ya** Tidak membuat stress 1

◯ **Ya** Sedikit membuat stress 2

◯ **Ya** Cukup membuat stress 3

◯ **Ya** Banyak membuat stress 4

◯ **Ya** Sangat membuat stress 5

3c. Apakah ada anggota keluarga terdekat anda meninggal dunia karena Covid-19?

◯ **Tidak** 0

◯ **Ya** Tidak membuat stress 1

◯ **Ya** Sedikit membuat stress 2

◯ **Ya** Cukup membuat stress 3

◯ **Ya** Banyak membuat stress 4

◯ **Ya** Sangat membuat stress 5

Apakah Anda memiliki anggota keluarga terdekat yang menetap di luar negeri yang Anda khawatirkan berisiko terkena Covid-19?

◯ **Tidak** 0

For peer review only

For peer review only - http://bmjopen.bmj.com/site/about/guidelines.xhtml

- **Ya** Tidak membuat stress 1
- **Ya** Sedikit membuat stress 2
- **Ya** Cukup membuat stress 3
- **Ya** Banyak membuat stress 4
- **Ya** Sangat membuat stress 5

5. Apakah Anda memiliki keluarga dekat di luar negeri yang tidak dapat Anda kunjungi atau mereka tidak dapat mengunjungi Anda saat ini?

- **Tidak** 0
- **Ya** Tidak membuat stress 1
- **Ya** Sedikit membuat stress 2
- **Ya** Cukup membuat stress 3
- **Ya** Banyak membuat stress 4
- **Ya** Sangat membuat stress 5

Pekerjaan

Berikan respon Ya / Tidak / Tidak berlaku terhadap pertanyaaan di bawah ini, jika hal berikut dialami oleh Anda atau anggota keluarga Anda terkait dengan wabah Covid-19. Jika jawaban anda Ya, mohon berikan indikasi penilaian tekanan (stress) yang dialami sesuai dengan skala berikut.

1. Apakah Anda atau anggota keluarga Anda termasuk pekerja di sektor penting (misalnya bekerja di bidang kesehatan, penegak hukum, keuangan, petugas yang berkaitan dengan kedaruratan atau penyedia jasa layanan penting lainnya)?

- **Tidak** 0
- **Ya** Tidak membuat stress 1
- **Ya** Sedikit membuat stress 2
- **Ya** Cukup membuat stress 3

For peer review only - http://bmjopen.bmj.com/site/about/guidelines.xhtml

◯ **Ya** Banyak membuat stress 4

◯ **Ya** Sangat membuat stress 5

2. Apakah Anda atau anggota keluarga Anda mengalami perubahan (penambahan/pengurangan) jam kerja?

◯ **Tidak** 0

◯ **Ya** Tidak membuat stress 1

◯ **Ya** Sedikit membuat stress 2

◯ **Ya** Cukup membuat stress 3

◯ **Ya** Banyak membuat stress 4

◯ **Ya** Sangat membuat stress 5

3. Apakah Anda atau anggota keluarga Anda harus berubah tempat kerjanya menjadi bekerja dari rumah?

◯ **Tidak** 0

◯ **Ya** Tidak membuat stress 1

◯ **Ya** Sedikit membuat stress 2

◯ **Ya** Cukup membuat stress 3

◯ **Ya** Banyak membuat stress 4

◯ **Ya** Sangat membuat stress 5

4. Apakah Anda atau anggota keluarga Anda terpaksa berubah jenis pekerjaannya?

◯ **Tidak** 0

◯ **Ya** Tidak membuat stress 1

◯ **Ya** Sedikit membuat stress 2

◯ **Ya** Cukup membuat stress 3

For peer review only

For peer review only - http://bmjopen.bmj.com/site/about/guidelines.xhtml

- Ya Banyak membuat stress 4
- Ya Sangat membuat stress 5

5. Apakah Anda atau anggota keluarga Anda kehilangan pekerjaan?

- **Tidak Berlaku**
- **Tidak** 0
- **Ya** Tidak membuat stress 1
- **Ya** Sedikit membuat stress 2
- **Ya** Cukup membuat stress 3
- **Ya** Banyak membuat stress 4
- **Ya** Sangat membuat stress 5

6. Apakah usaha Anda atau usaha anggota keluarga Anda mengalami kesulitan bertahan saat ini?

- **Tidak Berlaku**
- **Tidak** 0
- **Ya** Tidak membuat stress 1
- **Ya** Sedikit membuat stress 2
- **Ya** Cukup membuat stress 3
- **Ya** Banyak membuat stress 4
- **Ya** Sangat membuat stress 5

Pendapatan

Berikan respon Ya / Tidak terhadap pertanyaaan di bawah ini, jika hal berikut dialami oleh Anda, keluarga Anda atau anggota keluarga Anda terkait dengan wabah Covid-19. Jika jawaban anda Ya, mohon berikan indikasi penilaian tekanan (stress) yang dialami sesuai dengan skala berikut.

For peer review only - http://bmjopen.bmj.com/site/about/guidelines.xhtml

1. Apakah keluarga Anda mengalami penurunan pendapatan?

- **Tidak** 0
- **Ya** Tidak membuat stress 1
- **Ya** Sedikit membuat stress 2
- **Ya** Cukup membuat stress 3
- **Ya** Banyak membuat stress 4
- **Ya** Sangat membuat stress 5

2. Apakah keluarga Anda mengalami penurunan pendapatan yang mengakibatkan Anda kesulitan membayar tagihan atau membeli makanan/bahan pokok makanan?

- **Tidak** 0
- **Ya** Tidak membuat stress 1
- **Ya** Sedikit membuat stress 2
- **Ya** Cukup membuat stress 3
- **Ya** Banyak membuat stress 4
- **Ya** Sangat membuat stress 5

Hubungan Keluarga

Berikan respon Ya/Tidak terhadap pertanyaan di bawah ini, jika hal berikut dialami oleh Anda terkait dengan wabah Covid-19. Jika jawaban anda Ya, mohon berikan indikasi penilaian tekanan (stress) yang dialami sesuai dengan skala berikut. Akibat dari Covid-19………

1. Meningkatkan pertengkaran/memperburuk komunikasi/hubungan menjadi renggang-jauh dalam keluarga Anda?

- **Tidak** 0
- **Ya** Tidak membuat stress 1
- **Ya** Sedikit membuat stress 2

For peer review only - http://bmjopen.bmj.com/site/about/guidelines.xhtml

- **Ya** Cukup membuat stress 3
- **Ya** Banyak membuat stress 4
- **Ya** Sangat membuat stress 5

2. Hubungan Anda dengan teman/kerabat (baik secara fisik maupun daring/online) menurun?

- **Tidak** 0
- **Ya** Tidak membuat stress 1
- **Ya** Sedikit membuat stress 2
- **Ya** Cukup membuat stress 3
- **Ya** Banyak membuat stress 4
- **Ya** Sangat membuat stress 5

Perubahan dalam kegiatan dan perilaku sehari-hari

Berikan respon Ya / Tidak / Tidak Berlaku terhadap pertanyaan di bawah ini, jika hal berikut dialami oleh Anda terkait dengan wabah Covid-19. Jika jawaban anda Ya, mohon berikan indikasi penilaian tekanan (stress) yang dialami sesuai dengan skala berikut.

1. Apakah wabah Covid-19 telah merubah kegiatan sehari-hari Anda (Sebagai contoh, tidur, olah raga, waktu bebas, aktifitas yang menyenangkan atau hobi)?

- **Tidak** 0
- **Ya** Tidak membuat stress 1
- **Ya** Sedikit membuat stress 2
- **Ya** Cukup membuat stress 3
- **Ya** Banyak membuat stress 4
- **Ya** Sangat membuat stress 5

For peer review only

2. Membuat Anda menunda/menghindar untuk mencari bantuan kesehatan saat Anda atau keluarga Anda membutuhkannya?

- **Tidak** 0
- **Ya** Tidak membuat stress 1
- **Ya** Sedikit membuat stress 2
- **Ya** Cukup membuat stress 3
- **Ya** Banyak membuat stress 4
- **Ya** Sangat membuat stress 5

3. Menurunkan keterlibatan Anda dalam aktifitas keagamaan (baik tatap muka ataupun online)?

- **Tidak Berlaku**
- **Tidak** 0
- **Ya** Tidak membuat stress 1
- **Ya** Sedikit membuat stress 2
- **Ya** Cukup membuat stress 3
- **Ya** Banyak membuat stress 4
- **Ya** Sangat membuat stress 5

4. Apakah ada perubahan besar terhadap keseharian Anda?

- **Tidak** 0
- **Jika ada, mohon jelaskan** 1 ____

Jika jawaban anda Ya, mohon berikan indikasi penilaian tekanan (stress) yang dialami sesuai dengan skala berikut.

- Tidak membuat stress 1
- Sedikit membuat stress 2

For peer review only - http://bmjopen.bmj.com/site/about/guidelines.xhtml

○ Cukup membuat stress 3

○ Banyak membuat stress 4

○ Sangat membuat stress 5

Paparan terhadap informasi mengenai Covid-19

1. Terkait dengan Covid-19, dari manakah Anda mendapatkan informasinya?
(Mohon dipilih sebanyak mungkin sesuai yang Anda alami)

☐ Berita

☐ Facebook/Twitter

☐ Situs kesehatan

☐ Dari mulut ke mulut

☐ Lain-lain (Mohon dijelaskan) ____

2. Seberapa banyak tekanan (stress) yang Anda alami karena pemberitaan mengenai Covid-19?

○ Tidak membuat stress 1

○ Sedikit membuat stress 2

○ Cukup membuat stress 3

○ Banyak membuat stress 4

○ Sangat membuat stress 5

Perubahan sejak Covid-19

Dari pertanyaan di bawah ini, pilihlah yang Anda rasakan dengan melingkari nomor yang sesuai dengan situasi Anda. Mohon bandingkan saat ini dengan sebelum wabah Covid-19...

1. Bagaimana Anda menilai tingkat tekanan/stres yang dialami?

○ Sangat tidak merasakan tekanan/stress 1

○ Sedikit tidak merasakan tekanan/stress 2

For peer review only - http://bmjopen.bmj.com/site/about/guidelines.xhtml

- Tidak ada perubahan 3
- Lebih merasakan tekanan/stress 4
- Sangat merasakan tekanan/stress 5

2. Bagaimana Anda menilai tingkat tekanan (stress) yang dirasakan oleh anggota keluarga Anda secara keseluruhan?

- Sangat tidak merasakan tekanan/stress 1
- Sedikit tidak merasakan tekanan/stress 2
- Tidak ada perubahan 3
- Lebih merasakan tekanan/stress 4
- Sangat merasakan tekanan/stress 5

3. Bagaimana Anda menilai kesejahteraan psikologis Anda?

- Sangat baik 1
- Lebih baik 2
- Tidak ada perubahan 3
- Lebih buruk 4
- Sangat buruk 5

4. Bagaimana Anda menilai kesejahteraan psikologis anggota keluarga Anda secara keseluruhan?

- Sangat baik 1
- Lebih baik 2
- Tidak ada perubahan 3
- Lebih buruk 4
- Sangat buruk 5

For peer review only

K-10

Sepuluh pertanyaan berikut ini menanyakan perasaan Anda dalam 4 minggu terakhir. Untuk setiap pertanyaan, mohon lingkari pilihan yang menjelaskan seringnya Anda merasakan hal tersebut.

	Tidak pernah 1	Jarang 2	Kadang-kadang 3	Hampir selalu 4	Setiap saat 5
1. Dalam kurun waktu empat minggu terakhir, seberapa sering Anda merasa lelah tanpa sebab yang jelas?	○	○	○	○	○
2. Dalam empat minggu terakhir, seberapa sering Anda merasa cemas?	○	○	○	○	○
3. Dalam empat minggu terakhir seberapa sering Anda merasa cemas dan tidak bisa menenangkan diri?	○	○	○	○	○
4. Dalam empat minggu terakhir, seberapa sering Anda merasa tidak punya harapan?	○	○	○	○	○
5. Dalam empat minggu terakhir, seberapa sering Anda merasa gelisah atau resah?	○	○	○	○	○
6. Dalam empat minggu terakhir, seberapa sering Anda merasa gelisah yang menyebabkan Anda tidak dapat duduk diam?	○	○	○	○	○
7. Dalam empat minggu terakhir, seberapa sering Anda merasa tertekan?	○	○	○	○	○
8. Dalam empat minggu terakhir, seberapa sering Anda merasa berat sekali untuk melakukan sesuatu?	○	○	○	○	○
9. Dalam empat minggu terakhir, seberapa sering Anda merasa sedih sehingga tidak ada apapun yang dapat menghibur Anda?	○	○	○	○	○
10. Dalam empat minggu terakhir, seberapa sering Anda merasa tidak berharga sama sekali?	○	○	○	○	○

For peer review only

For peer review only - http://bmjopen.bmj.com/site/about/guidelines.xhtml

PCL-5

Di bawah ini adalah masalah-masalah yang seringkali dialami seseorang dalam menghadapi situasi Covid-19. Mohon dibaca setiap masalah dengan teliti dan kemudian lingkari nomor untuk mengindikasikan seberapa sering Anda terganggu oleh masalah tersebut dalam kurun waktu satu bulan terakhir. Dalam satu Bulan terakhir, seberapa sering Anda terganggu dengan hal berikut:

	**Tidak sama sekali** 0	**Sedikit tergganggu** 1	**Kadang-kadang tergganggu** 2	**Hampir tergganggu** 3	**Sangat tergganggu** 4
1. Terulangnya kenangan yang tidak diinginkan dan membuat tertekan/stres?	○	○	○	○	○
2. Terulangnya mimpi yang menganggu saat mengalami tekanan/stres?	○	○	○	○	○
3. Tiba-tiba merasakan atau bertingkah seakan-akan pengalaman saat tekanan/stres terjadi terulang kembali (seakan-akan Anda kembali ke situasi tersebut?	○	○	○	○	○
4. Merasa marah saat terjadi sesuatu yang mengingatkan Anda pada pengalaman saat tekanan/stres terjadi?	○	○	○	○	○
5. Mengalami reaksi fisik yang kuat saat sesuatu terjadi dimana mengingatkan Anda pada pengalaman saat tekanan/stres terjadi (sebagai contoh, jantung berdebar kencang, mengalami kesusahan bernafas, berkeringat)?	○	○	○	○	○
6. Menghindari kenangan, pikiran ataupun perasaan yang terkait dengan pengalaman saat tekanan/stres terjadi?	○	○	○	○	○
7. Menghindari hal-hal dari pihak luar yang mengingatkan Anda kepada pengalaman saat tekanan/stres terjadi (sebagai contoh, orang, tempat, percakapan, aktifitas, benda atau situasi)?	○	○	○	○	○
8. Kesulitan dalam mengingat hal penting dari pengalaman saat tekanan/stres terjadi?	○	○	○	○	○

For peer review only

9. Mengalami prasangka negatif yang kuat terhadap diri sendiri, orang lain, atau dunia (sebagai contoh, memiliki pikiran seperti: saya jahat, ada hal yang salah di dalam diri saya, tidak ada yang bisa dipercaya, dunia ini sangat kejam)?	○	○	○	○	○
10. Menyalahkan diri sendiri atau orang lain terhadap pengalaman saat tekanan/stres terjadi ataupun terhadap hal yang terjadi setelahnya?	○	○	○	○	○
11. Memilik perasaan negatif yang kuat seperti ketakutan, perasaan ngeri, marah, merasa bersalah ataupun malu?	○	○	○	○	○
12. Tidak tertarik lagi melakukan kegiatan yang biasanya Anda sukai?	○	○	○	○	○
13. Merasa jauh dan tidak memiliki hubungan dengan orang lain?	○	○	○	○	○
14. Kesulitan untuk memiliki perasaan positif (sebagai contoh, tidak dapat merasakan kebahagian atau mencintai orang yang dekat dengan Anda)?	○	○	○	○	○
15. Bertingkah laku yang mengganggu, marah tanpa kendali atau bersikap agresif?	○	○	○	○	○
16. Melakukan hal yang berisiko tinggi ataupun melakukan hal yang dapat membahayakan diri Anda?	○	○	○	○	○
17. Bersikap “sangat waspada” atau berhati-hati atau pun berjaga-jaga?	○	○	○	○	○
18. Merasa gelisah dan mudah terkejut?	○	○	○	○	○
19. Kesulitan dalam berkonsentrasi?	○	○	○	○	○
20. Susah tertidur atau tidur lebih lama?	○	○	○	○	○

For peer review only

For peer review only - http://bmjopen.bmj.com/site/about/guidelines.xhtml

WHO-5

Mohon berikan indikasi untuk ke lima pernyataan di bawah ini yang sesuai dengan yang Anda rasakan selama kurun waktu 2 minggu terakhir. Dalam kurun waktu 2 minggu terakhir

	**Setiap waktu 5**	**Kebanyakan waktu 4**	**Sebagian waktu 3**	**Hampir sebagian waktu 2**	**Sesekali 1**	**Tidak sama sekali 0**
1. Saya merasa gembira dan bahagia	○	○	○	○	○	○
2. Saya merasa tenang dan rileks	○	○	○	○	○	○
3. Saya merasa aktif dan semangat	○	○	○	○	○	○
4. Saya terbangun dengan perasaan segar dan cukup istirahat	○	○	○	○	○	○
5. Kegiatan sehari-hari saya penuh dengan hal-hal yang saya sukai	○	○	○	○	○	○

For peer review only

PTGI

Dari pernyataan di bawah ini, mohon indikasikan penilaian terjadinya perubahan dalam hidup Anda sebagai akibat dari Covid-19 dengan menggunakan skala di bawah ini.

	Tidak sama sekali 0	Sedikit 1	Agak 2	Kadang-kadang 3	Hampir 4	Sangat 5
1. Saya merubah prioritas mengenai apa yang penting dalam hidup.	○	○	○	○	○	○
2. Saya memiliki apresiasi besar terhadap nilai hidup.	○	○	○	○	○	○
3. Saya mengembangkan minat baru.	○	○	○	○	○	○
4. Saya memiliki perasaan besar terhadap kepercayaan diri.	○	○	○	○	○	○
5. Saya memiliki pemahaman yang baik terhadap masalah spiritual.	○	○	○	○	○	○
6. Saya melihat bahwa saya bisa meminta bantuan kepada orang lain di saat kesulitan.	○	○	○	○	○	○
7. Saya menempuh jalan baru bagi hidup saya.	○	○	○	○	○	○
8. Saya memiliki rasa kedekatan dengan orang lain.	○	○	○	○	○	○
9. Saya lebih bisa mengekspresikan emosi.	○	○	○	○	○	○
10. Saya tahu jika saya bisa mengatasi kesulitan.	○	○	○	○	○	○
11. Saya bisa melakukan hal-hal baik dalam hidup saya.	○	○	○	○	○	○
12. Saya semakin bisa menerima hal-hal yang terjadi.	○	○	○	○	○	○
13. Saya bisa lebih menghargai setiap hari yang saya lalui.	○	○	○	○	○	○

For peer review only

14. Kesempatan baru tersedia dibandingkan sebelumnya.	○	○	○	○	○	○
15. Saya lebih memiliki rasa kasih sayang kepada sesama.	○	○	○	○	○	○
16. Saya lebih memberikan upaya dalam hubungan saya dengan orang lain	○	○	○	○	○	○
17. Saya lebih bisa mengusahakan untuk merubah hal yang memang bisa diubah.	○	○	○	○	○	○
18. Saya memiliki keyakinan yang lebih kuat terhadap agama.	○	○	○	○	○	○
19. Saya menyadari bahwa saya lebih kuat daripada yang saya kira.	○	○	○	○	○	○
20. Saya menyadari bahwa ternyata orang lain itu baik.	○	○	○	○	○	○
21. Saya lebih bisa menerima bahwa saya membutuhkan orang lain.	○	○	○	○	○	○

For peer review only

International Study – Malaysia (Malay)

Sociodemographic Information

1. Jantina

- ◯ Lelaki
- ◯ Perempuan
- ◯ Lain-lain
- ◯ Lebih suka tidak diberitahu

2. Umur (dalam tahun)

____

3. Status perkahwinan

- ◯ Bujang
- ◯ Mempunya hubungan
- ◯ Berkahwin
- ◯ Tidak tinggal bersama
- ◯ Bercerai
- ◯ Duda/Janda

4. Apakah agama anda?

- ◯ Tiada agama
- ◯ Islam
- ◯ Kristian
- ◯ Hindu

For peer review only

○ Buddha

○ Lain-lain (Sila nyatakan) ____

5. Apakah bangsa anda? (Anda boleh menyebut lebih daripada satu)

____

6. Apakah Bahasa yang paling fasih digunakan dalam kehidupan seharian?

____

Berikut merupakan dua soalan berkenaan kebolehan anda bertutur di dalam Bahasa Inggeris. 6a. Saya faham Bahasa Inggeris dengan baik

○ Sangat tidak setuju 1

○ Tidak setuju 2

○ Natural 3

○ Setuju 4

○ Sangat Setuju 5

6b. Saya boleh bertutur Bahasa Inggeris dengan baik

○ Tidak langsung 1

○ Sedikit 2

○ Lebih kurang 3

○ Agak banyak 4

○ Sangat banyak 5

7. Apakah pendidikan tertinggi anda?

○ Tiada Pendidikan formal

For peer review only - http://bmjopen.bmj.com/site/about/guidelines.xhtml

○ Matrikulasi

○ Diploma / Sijil

○ Sarjana Muda

○ Sarjana / PhD

8. Status belajar/bekerja (tandakan yang berkenaan)

☐ Pelajar sepenuh masa

☐ Pelajar separuh masa

☐ Tidak bekerja/Sedang mencari pekerjaan

☐ Bekerja sepenuh masa

☐ Bekerja separuh masa

☐ Bekerja Sendiri sepenuh masa

☐ Bekerja Sendiri separuh masa

☐ Cuti bergaji

☐ Tinggal bersama ibubapa (tidak bergaji)

☐ Bersara

☐ Lain-lain (Sila nyatakan) ____

9. Pekerjaan

____

10. Pendapatan bulanan isirumah (Ringgit Malaysia)

____

For peer review only - http://bmjopen.bmj.com/site/about/guidelines.xhtml

10a. Apakah kadar pendapatan bulanan isirumah, jika dibandingkan dengan purata pendapatan penduduk Malaysia?

- ○ Bawah purata
- ○ Sama dengan purata
- ○ Atas purata

11. Adakah anda sekarang berada di dalam perintah kawalan pergerakan/arahan duduk di rumah?

- ○ Ya
- ○ Tidak

12. Adakahand telah menerima suntikan vaksin?

- ○ Ya
- ○ Tidak

12a. Jika belum menerima suntikan vaksin, adakah anda akan mendapatkan vaksin COVID-19 di masa akan datang?

- ○ Ya
- ○ Tidak

13. Kita sedar bahawa sesetengah orang telah didedahkan dengan insiden trauma pada masa lampau yang mana telah memudahkan atau menyusahkan untuk menangani masalah. Sebelum pandemic COVID-19, adakah anda pernah menyaksikan atau mengalami mana-mana perkara berikut: (Sila tanda sebanyak mana yang berkaitan)

- ☐ Bencana alam (seperti banjir, gempa bumi dan lain-lain)
- ☐ Tinggal di dalam zon peperangan atau telah terdedah kepada konflik tetera
- ☐ Kesukaran kanak-kanak sebelum usia 16 tahun (seperti pengabaian, buli, serangan fizikal atau seksual)

For peer review only - http://bmjopen.bmj.com/site/about/guidelines.xhtml

For peer review only

☐ Serangan fizikal atau seksual selepas usia 16 tahun

☐ Kemalangan fizikal yang serius

☐ Lain-lain - sila nyatakan (pilihan) ____

☐ Tiada satu pun di atas

For peer review only

For peer review only - http://bmjopen.bmj.com/site/about/guidelines.xhtml

Covid Psychosocail Impacts Scale

Kesan keatas diri sendiri

Untuk kenyataan di bawah, sila jawab Ya/Tidak jika kesan daripada Covid-19, berlaku kepada diri anda sendiri. Jika jawapan anda adalah Ya, sila nyatakan tahap tekanan yang dialami, berdasarkan skala dibawah.

1. Adakah anda mengalami masalah kesihatan yang boleh menyebabkan risiko anda untuk dijangkiti Covid-19 meningkat?

- **Tidak** 0
- **Ya** Tiada tekanan langsung 1
- **Ya** Tekanan tahap minima 2
- **Ya** Tekanan tahap sederhana 3
- **Ya** Tekanan tahap tinggi 4
- **Ya** Tekanan yang sangat tinggi 5

2. Adakah anda pernah merasakan bahaya risiko terdedah kepada seseorang yang menghidap Covid-19?

- **Tidak** 0
- **Ya** Tiada tekanan langsung 1
- **Ya** Tekanan tahap minima 2
- **Ya** Tekanan tahap sederhana 3
- **Ya** Tekanan tahap tinggi 4
- **Ya** Tekanan yang sangat tinggi 5

3. Adakah anda fikir bahawa anda telah dijangkiti COVID-19?

- **Tidak** 0

For peer review only

For peer review only - http://bmjopen.bmj.com/site/about/guidelines.xhtml

- **Ya** Tiada tekanan langsung 1
- **Ya** Tekanan tahap minima 2
- **Ya** Tekanan tahap sederhana 3
- **Ya** Tekanan tahap tinggi 4
- **Ya** Tekanan yang sangat tinggi 5

3a. Adakah anda pernah mendapat keputusan positif bagi ujian COVID-19?

- **Tidak** 0
- **Ya** Tiada tekanan langsung 1
- **Ya** Tekanan tahap minima 2
- **Ya** Tekanan tahap sederhana 3
- **Ya** Tekanan tahap tinggi 4
- **Ya** Tekanan yang sangat tinggi 5

3b. Adakah anda mempunyai simptom-simptom COVID-19?

- **Tidak** 0
- **Ya** Tiada tekanan langsung 1
- **Ya** Tekanan tahap minima 2
- **Ya** Tekanan tahap sederhana 3
- **Ya** Tekanan tahap tinggi 4
- **Ya** Tekanan yang sangat tinggi 5

For peer review only

Kesan kepada keluarga

Untuk kenyataan di bawah, sila jawab Ya/Tidak jika kesan daripada Covid-19, berlaku kepada ahli keluarga terdekat anda. Jika jawapan anda adalah Ya, sila nyatakan tahap tekanan yang dialami, berdasarkan skala dibawah.

1. Adakah ahli keluarga terdekat anda mengalami masalah kesihatan yang boleh menyebabkan risiko mereka untuk dijangkiti Covid-19 meningkat?

- ○ **Tidak** 0
- ○ **Ya** Tiada tekanan langsung 1
- ○ **Ya** Tekanan tahap minima 2
- ○ **Ya** Tekanan tahap sederhana 3
- ○ **Ya** Tekanan tahap tinggi 4
- ○ **Ya** Tekanan yang sangat tinggi 5

2. Adakah anda merasakan bahawa setiap ahli keluarga terdekat anda berisiko untuk terdedah kepada seseorang yang menghidap Covid-19?

- ○ **Tidak** 0
- ○ **Ya** Tiada tekanan langsung 1
- ○ **Ya** Tekanan tahap minima 2
- ○ **Ya** Tekanan tahap sederhana 3
- ○ **Ya** Tekanan tahap tinggi 4
- ○ **Ya** Tekanan yang sangat tinggi 5

3. Adakah ahli keluarga terdekat anda pernah menjalani Covid-19?

- ○ **Tidak** 0
- ○ **Ya** Tiada tekanan langsung 1

For peer review only - http://bmjopen.bmj.com/site/about/guidelines.xhtml

- **Ya** Tekanan tahap minima 2
- **Ya** Tekanan tahap sederhana 3
- **Ya** Tekanan tahap tinggi 4
- **Ya** Tekanan yang sangat tinggi 5

3a. Adakah keputusan ahli keluarga terdekat anda positif Covid-19?

- **Tidak** 0
- **Ya** Tiada tekanan langsung 1
- **Ya** Tekanan tahap minima 2
- **Ya** Tekanan tahap sederhana 3
- **Ya** Tekanan tahap tinggi 4
- **Ya** Tekanan yang sangat tinggi 5

3b. Adakah ahli keluarga terdekat anda mempunyai simptom-simptom Covid-19?

- **Tidak** 0
- **Ya** Tiada tekanan langsung 1
- **Ya** Tekanan tahap minima 2
- **Ya** Tekanan tahap sederhana 3
- **Ya** Tekanan tahap tinggi 4
- **Ya** Tekanan yang sangat tinggi 5

3c. Adakah ahli keluarga terdekat anda meninggal dunia disebabkan oleh Covid-19?

- **Tidak** 0

For peer review only - http://bmjopen.bmj.com/site/about/guidelines.xhtml
For peer review only

◯ **Ya** Tiada tekanan langsung 1

◯ **Ya** Tekanan tahap minima 2

◯ **Ya** Tekanan tahap sederhana 3

◯ **Ya** Tekanan tahap tinggi 4

◯ **Ya** Tekanan yang sangat tinggi 5

4. Adakah anda mempunyai ahli keluarga terdekat di luar negara yang dikhuatiri berisiko untuk dijangkiti Covid-19?

◯ **Tidak** 0

◯ **Ya** Tiada tekanan langsung 1

◯ **Ya** Tekanan tahap minima 2

◯ **Ya** Tekanan tahap sederhana 3

◯ **Ya** Tekanan tahap tinggi 4

◯ **Ya** Tekanan yang sangat tinggi 5

5. Adakah anda mempunyai ahli di luar negara yang tidak dapat anda lawati atau tidak dapat melawat anda?

◯ **Tidak** 0

◯ **Ya** Tiada tekanan langsung 1

◯ **Ya** Tekanan tahap minima 2

◯ **Ya** Tekanan tahap sederhana 3

◯ **Ya** Tekanan tahap tinggi 4

◯ **Ya** Tekanan yang sangat tinggi 5

For peer review only

For peer review only - http://bmjopen.bmj.com/site/about/guidelines.xhtml

Pekerjaan

Untuk kenyataan di bawah, sila jawab Ya / Tidak / Tidak berkenaan jika kesan daripada Covid-19, berlaku kepada anda atau sesiapa sahaja di rumah anda. Jika jawapan anda adalah Ya, sila nyatakan tahap tekanan yang dialami, berdasarkan skala dibawah.

1. Adakah anda atau sesiapa sahaja di rumah anda dianggap sebagai pekerja perkhidmatan perlu (Contohnya, bidang perubatan dan penjagaan kesihatan, penguatkuasaan undang-undang, perkhidmatan kecemasan, pembekal barang dan perkhidmatan penting)?

- ○ **Tidak** 0
- ○ **Ya** Tiada tekanan langsung 1
- ○ **Ya** Tekanan tahap minima 2
- ○ **Ya** Tekanan tahap sederhana 3
- ○ **Ya** Tekanan tahap tinggi 4
- ○ **Ya** Tekanan yang sangat tinggi 5

For peer review only

2. Adakah anda atau sesiapa sahaja di rumah anda mengalami perubahan yang besar pada waktu bekerja?

- ○ **Tidak** 0
- ○ **Ya** Tiada tekanan langsung 1
- ○ **Ya** Tekanan tahap minima 2
- ○ **Ya** Tekanan tahap sederhana 3
- ○ **Ya** Tekanan tahap tinggi 4
- ○ **Ya** Tekanan yang sangat tinggi 5

3. Adakah anda atau sesiapa sahaja di rumah anda perlu bertukar tempat kerja dan bekerja dari rumah?

- ○ **Tidak** 0

For peer review only - http://bmjopen.bmj.com/site/about/guidelines.xhtml

Ya Tiada tekanan langsung 1

Ya Tekanan tahap minima 2

Ya Tekanan tahap sederhana 3

Ya Tekanan tahap tinggi 4

Ya Tekanan yang sangat tinggi 5

4. Adakah anda atau sesiapa sahaja di rumah anda perlu bertukar jenis pekerjaan?

Tidak 0

Ya Tiada tekanan langsung 1

Ya Tekanan tahap minima 2

Ya Tekanan tahap sederhana 3

Ya Tekanan tahap tinggi 4

Ya Tekanan yang sangat tinggi 5

5. Adakah anda atau ahli rumahtangga anda telah hilang punca pendapatan?

Tidak berkenaan

Tidak 0

Ya Tiada tekanan langsung 1

Ya Tekanan tahap minima 2

Ya Tekanan tahap sederhana 3

Ya Tekanan tahap tinggi 4

Ya Tekanan yang sangat tinggi 5

For peer review only

6. Adakah perniagaan atau sumber pencarian anda atau ahli rumahtangga anda terjejas dan berkemungkinan untuk tidak beroperasi lagi?

- **Tidak berkenaan**
- **Tidak** 0
- **Ya** Tiada tekanan langsung 1
- **Ya** Tekanan tahap minima 2
- **Ya** Tekanan tahap sederhana 3
- **Ya** Tekanan tahap tinggi 4
- **Ya** Tekanan yang sangat tinggi 5

For peer review only

Pendapatan

Untuk kenyataan di bawah, sila jawab Ya/Tidak jika kesan daripada Covid-19 ini telah berlaku kepada ahli rumahtangga anda. Jika jawapan anda adalah Ya, sila nyatakan tahap tekanan yang dialami, berdasarkan skala dibawah.

1. Adakah isu kewangan rumahtangga anda merosot dengan mendadak?

- **Tidak** 0
- **Ya** Tiada tekanan langsung 1
- **Ya** Tekanan tahap minima 2
- **Ya** Tekanan tahap sederhana 3
- **Ya** Tekanan tahap tinggi 4
- **Ya** Tekanan yang sangat tinggi 5

2. Adakah rumahtagga anda menghadapi masalah besar untuk membeli barangan keperluan rumah atau membayar bil-bil semasa?

For peer review only - http://bmjopen.bmj.com/site/about/guidelines.xhtml

- **Tidak** 0
- **Ya** Tiada tekanan langsung 1
- **Ya** Tekanan tahap minima 2
- **Ya** Tekanan tahap sederhana 3
- **Ya** Tekanan tahap tinggi 4
- **Ya** Tekanan yang sangat tinggi 5

Perhubungan (bersama insan lain)

Untuk kenyataan di bawah, sila jawab Ya/Tidak jika kesan daripada Covid-19, berlaku kepada diri anda sendiri. Jika jawapan anda adalah Ya, sila nyatakan tahap tekanan yang dialami, berdasarkan skala dibawah. Sebagai tindak balas kepada Covid-19....

1. Adakah ia telah mendatangkan kesusahan dan tekanan kepada keluarga anda?

- **Tidak** 0
- **Ya** Tiada tekanan langsung 1
- **Ya** Tekanan tahap minima 2
- **Ya** Tekanan tahap sederhana 3
- **Ya** Tekanan tahap tinggi 4
- **Ya** Tekanan yang sangat tinggi 5

2. Adakah ia telah mengurangkan perhubungan antara rakan-rakan anda (sama ada secara fizikal ataupun dalam talian)?

- **Tidak** 0
- **Ya** Tiada tekanan langsung 1
- **Ya** Tekanan tahap minima 2

For peer review only - http://bmjopen.bmj.com/site/about/guidelines.xhtml

- **Ya** Tekanan tahap sederhana 3
- **Ya** Tekanan tahap tinggi 4
- **Ya** Tekanan yang sangat tinggi 5

Perubahan kepada Rutin dan Tingkah Laku Harian

Untuk kenyataan di bawah, sila jawab Ya/Tidak/Tidak berkenaan jika kesan daripada Covid-19, berlaku kepada diri anda sendiri. Jika jawapan anda adalah Ya, sila nyatakan tahap tekanan yang dialami, berdasarkan skala dibawah. Sebagai tindak balas kepada Covid-19....

1. Adakah wabak Covid ini telah mencetus perubahan kepada rutin harian anda (Contohnya, jadual tidur, senaman, waktu rehat, waktu riadah, atau hobi)?

- **Tidak** 0
- **Ya** Tiada tekanan langsung 1
- **Ya** Tekanan tahap minima 2
- **Ya** Tekanan tahap sederhana 3
- **Ya** Tekanan tahap tinggi 4
- **Ya** Tekanan yang sangat tinggi 5

2. Adakah ia telah membuatkan anda menangguhkan atau mengelakkan mencari rawatan perubatan sekiranya anda atau ahli keluarga anda memerlukannya?

- **Tidak** 0
- **Ya** Tiada tekanan langsung 1
- **Ya** Tekanan tahap minima 2
- **Ya** Tekanan tahap sederhana 3
- **Ya** Tekanan tahap tinggi 4

For peer review only - http://bmjopen.bmj.com/site/about/guidelines.xhtml

○ **Ya** Tekanan yang sangat tinggi 5

3. Adakah ia telah mengurangkan penglibatan anda dalam aktiviti keagamaan (sama ada secara peribadi atau dalam talian)?

○ **Tidak berknaan**

○ **Tidak** 0

○ **Ya** Tiada tekanan langsung 1

○ **Ya** Tekanan tahap minima 2

○ **Ya** Tekanan tahap sederhana 3

○ **Ya** Tekanan tahap tinggi 4

○ **Ya** Tekanan yang sangat tinggi 5

4. Adakah ia telah menyebabkan sebarang perubahan besar kepada rutin harian anda?

○ **Tidak** 0

○ **Ya (sila jelaskan)** 1 ____

Jika jawapan anda adalah Ya, sila nyatakan tahap tekanan yang dialami, berdasarkan skala dibawah.

○ Tiada tekanan langsung 1

○ Tekanan tahap minima 2

○ Tekanan tahap sederhana 3

○ Tekanan tahap tinggi 4

○ Tekanan yang sangat tinggi 5

For peer review only - http://bmjopen.bmj.com/site/about/guidelines.xhtml

Pendedahan kepada maklumat tentang Covid-19

1. Apakah sumber maklumat anda berkenaan dengan Covid-19? (Tandakan seberapa banyak yang mungkin)

- ☐ Berita
- ☐ Facebook atau Twitter
- ☐ Laman web kesihatan
- ☐ daripada orang lain
- ☐ Lain-lain (sila jelaskan) ____

2. Sebanyak manakah anda berasa tertekan membaca berita tentang Covid-19?

- ○ Tiada tekanan langsung 1
- ○ Tekanan tahap minima 2
- ○ Tekanan tahap sederhana 3
- ○ Tekanan tahap tinggi 4
- ○ Tekanan yang sangat tinggi 5

Perubahan sejak Covid-19

Untuk soalan-soalan berikutnya, sila nyatakan berdasarkan skala dibawah. Bandingkan keadaan sekarang dengan keadaan semasa sebelum wabak Covid-19 berlaku....

1. Bagaimanakah anda menilai tahap tekanan peribadi anda?

- ○ Sangat tidak tertekan 1
- ○ Kurang tertekan 2
- ○ Lebih kurang sama 3

For peer review only

- Lebih tertekan 4
- Sangat tertekan 5

2. Bagaimanakah anda menilai tahap tekanan ahli rumahtangga anda?

- Sangat tidak tertekan 1
- Kurang tertekan 2
- Lebih kurang sama 3
- Lebih tertekan 4
- Sangat tertekan 5

3. Bagaimanakah anda menilai tahap kesihatan psikologi anda?

- Sangat baik 1
- Baik 2
- Lebih kurang sama 3
- Merosot 4
- Sangat merosot 5

4. Bagaimanakah anda menilai tahap kesihatan psikologi ahli rumahtangga anda?

- Sangat baik 1
- Baik 2
- Lebih kurang sama 3
- Merosot 4
- Sangat merosot 5

For peer review only - http://bmjopen.bmj.com/site/about/guidelines.xhtml

K-10

Sepuluh soalan berikut bertanyakan tentang perasaan yang anda rasai sejak empat minggu yang lepas. Untuk setiap soalan, sila bulatkan pilihan yang menunjukkan sebanyak mana anda merasai perasaan tersebut.

	**Tidak langsung 1**	**Kadang-kadang 2**	**Sedikit 3**	**Selalu 4**	**Sepanjang masa 5**
1. Dalam tempoh empat minggu yang lepas, berapa kerap anda berasa sangat penat tanpa sebarang sebab yang munasabah?	○	○	○	○	○
2. Dalam tempoh empat minggu yang lepas, berapa kerap anda berasa gugup/gementar?	○	○	○	○	○
3. Dalam tempoh empat minggu yang lepas, berapa kerap anda berasa sangat gugup/gementar sehinggga tiada apa yang boleh menenangkan anda?	○	○	○	○	○
4. Dalam tempoh empat minggu yang lepas, berapa kerap anda berasa putus asa?	○	○	○	○	○
5. Dalam tempoh empat minggu yang lepas, berapa kerap anda berasa gelisah?	○	○	○	○	○
6. Dalam tempoh empat minggu yang lepas, berapa kerap anda berasa sangat gelisah sehingga tidak boleh duduk diam?	○	○	○	○	○
7. Dalam tempoh empat minggu yang lepas, berapa kerap anda mengalami perasaan murung?	○	○	○	○	○
8. Dalam tempoh empat minggu yang lepas, berapa kerap anda berasa seperti segala yang dilakukan memerlukan usaha yang banyak?	○	○	○	○	○
9. Dalam tempoh empat minggu yang lepas, berapa kerap anda berasa sangat sedih sehingga tiada apa boleh menceriakan anda?	○	○	○	○	○
10. Dalam tempoh empat minggu yang lepas, berapa kerap anda merasakan tiada harga diri?	○	○	○	○	○

For peer review only

For peer review only - http://bmjopen.bmj.com/site/about/guidelines.xhtml

PCL-5

Berikut adalah senarai masalah yang sering dihadapi oleh seseorang individu sebagai tindak balas kepada suatu pengalaman yang sangat menekankan. Sila baca setiap soalan dengan teliti dan pilih satu nombor yang tertera di sebelah kanan untuk menunjukkan sebanyak mana anda terjejas dengan masalah tersebut pada bulan lalu.

Pada bulan lepas, sebanyak mana anda terganggu dengan:

	Tidak langsung 0	Sedikit 1	Sederhana 2	Sering 3	Sangat terjejas 4
1. Kenangan yang tidak diingini, berulang, dan mengganggu disebabkan oleh pengalaman buruk yang dialami?	○	○	○	○	○
2. Mimpi buruk yang berulang disebabkan oleh pengalaman buruk yang dialami?	○	○	○	○	○
3. Tiba-tiba terasa atau berperangai seolah-olah pengalaman buruk yang dialami itu berulang lagi (seakan-akan anda kembali ke situasi tersebut)?	○	○	○	○	○
4. Merasa sangat marah dan sedih apabila anda diingatkan dengan pengalaman buruk yang dialami?	○	○	○	○	○
5.Mengalami tindak balas fizikal yang kuat apabila anda diingatkan dengan pengalaman buruk yang dialami? (Contohnya jantung berdegup dengan laju, sukar bernafas, dan berpeluh).	○	○	○	○	○
6. Mengelak daripada mengingati sebarang kenangan, fikiran, atau perasaan yang berkaitan dengan pengalaman buruk yang dialami?	○	○	○	○	○
7. Mengelak daripada pengaruh luar yang akan mengingatkan kepada pengalaman buruk yang dialami? (Contohnya mengelak daripada berjumpa dengan orang, tempat, perbualan, aktiviti, benda, atau situasi)	○	○	○	○	○
8. Adakah anda berasa susah untuk mengingati hal-hal penting pengalaman buruk yang dialami itu?	○	○	○	○	○

For peer review only - http://bmjopen.bmj.com/site/about/guidelines.xhtml

9.Mempunyai tanggapan negatif yang kuat terhadap diri sendiri, orang lain, atau dunia (Contohnya, mempunyai pemikiran seperti: Saya jahat, ada sesuatu yang sangat salah dengan diri saya, tiada siapa boleh dipercayai, segalanya di dunia ini merbahaya)?

10. Adakah anda menyalahkan diri sendiri atau orang lain kerana berlakunya pengalaman buruk yang telah berlaku atau apa yang terjadi selepas itu?

11. Adakah anda mempunyai perasaan negatif yang kuat seperti takut/cemas, seram, marah, rasa bersalah, atau rasa malu?

12. Adakah anda hilang rasa minat kepada aktiviti yang pernah anda gemari?

13. Adakah anda terasa jauh atau terasing daripada orang lain?

14. Adakah anda sukar untuk merasakan perasaan positif (Contohnya, sukar atau tidak boleh rasa gembira atau perasaan sayang kepada orang terdekat)?

15. Adakah anda mudah marah, cepat sentap, atau bertindak ganas?

16. Adakah anda mengambil banyak risiko atau melakukan sesuatu yang boleh membahayakan diri anda?

17. Menjadi seorang yang sangat berwaspada atau berjaga-jaga?

18. Mudah rasa terkejut dan gelisah?

19. Adakah anda menghadapi masalah untuk menumpukan perhatian?

20. Adakah anda menghadapi masalah untuk tidur dan kekal tidur?

For peer review only

For peer review only - http://bmjopen.bmj.com/site/about/guidelines.xhtml

WHO-5

Untuk setiap kenyataan di bawah, sila nyatakan perasaan yang paling dirasai sejak dua minggu yang lepas. Dalam tempoh dua minggu yang lepas

	Sepanjang masa 5	Selalu 4	Seringkali (lebih daripada separuh masa) 3	Kadang-kadang (kurang daripada separuh masa) 2	Jarang 1	Tidak pernah 0
1. Saya berasa ceria dan bersemangat	○	○	○	○	○	○
2. Saya berasa tenang dan santai	○	○	○	○	○	○
3. Saya berasa aktif dan bertenaga	○	○	○	○	○	○
4. Saya berasa segar dan cukup rehat apabila bangun pagi	○	○	○	○	○	○
5. Kehidupan seharian saya dipenuhi dengan perkara yang saya minati	○	○	○	○	○	○

For peer review only

For peer review only - http://bmjopen.bmj.com/site/about/guidelines.xhtml

PTGI

Untuk setiap kenyataan di bawah, sila nyatakan sebanyak mana perubahan yang telah berlaku kepada hidup anda sejak peristiwa Covid-19, menggunakan skala di bawah.

	Tidak langsung 0	Sangat sedikit 1	Agak sedikit 2	Sederhana 3	Agak banyak 4	Sangat banyak 5
1. Saya ubah keutamaan terhadap apa yang penting dalam hidup ini.	○	○	○	○	○	○
2. Saya lebih menghargai nilai hidup saya sendiri.	○	○	○	○	○	○
3. Saya dapat memupuk minat yang baru.	○	○	○	○	○	○
4. Perasaan berdikari saya lebih meningkat.	○	○	○	○	○	○
5. Saya lebih memahami perkara kerohanian.	○	○	○	○	○	○
6. Saya dapat lihat yang saya boleh mengharapkan orang lain ketika saya di dalam kesusahan.	○	○	○	○	○	○
7. Saya memulakan perjalanan hidup yang baru.	○	○	○	○	○	○
8. Saya rasa lebih dekat dan akrab dengan orang lain.	○	○	○	○	○	○
9. Saya lebih bersedia untuk meluahkan emosi saya.	○	○	○	○	○	○
10. Saya yakin bahawa saya boleh melalui saat kesusahan.	○	○	○	○	○	○
11. Saya boleh melakukan perkara yang lebih bermanfaat dalam hidup saya.	○	○	○	○	○	○
12. Saya lebih senang menerima sebarang keputusan kepada perkara yang telah berlaku.	○	○	○	○	○	○

For peer review only

13. Saya dapat lebih bersyukur dan menghargai setiap hari yang ada.	○	○	○	○	○	○
14. Banyak peluang baru muncul yang sukar untuk didapatkan sebelum ini.	○	○	○	○	○	○
15. Rasa belas kasihan saya kepada orang lain meningkat.	○	○	○	○	○	○
16. Saya tingkatkan usaha saya dalam hubungan (bersama orang lain).	○	○	○	○	○	○
17. Saya lebih cenderung untuk mengubah perkara yang perlu diubah.	○	○	○	○	○	○
18. Kepercayaan kepada agama saya semakin meningkat.	○	○	○	○	○	○
19. Saya dapati yang saya lebih kuat daripada yang saya sangkakan.	○	○	○	○	○	○
20. Saya dapat belajar dan ketahui betapa baiknya orang-orang disekeliling saya.	○	○	○	○	○	○
21. Saya dapat terima dengan lebih baik bahawa saya memerlukan orang lain.	○	○	○	○	○	○

For peer review only

For peer review only - http://bmjopen.bmj.com/site/about/guidelines.xhtml

International Study – Somalia (Somali)

Sociodemographic Information

1. Jinsiga

- ◯ Lab
- ◯ Dheddig
- ◯ Mid Kale
- ◯ Ma jecli inaa ka hadlo

2. Da'daada (Sanad)

____

3. Xaalada guurka

- ◯ Waligeey ma guursan
- ◯ Qof ayaan xiriir la leeyahay
- ◯ Waa guursaday
- ◯ Kala maqanahay
- ◯ Kala tagnay
- ◯ Leeyga dhintay

4. Apa agama yang Anda?

- ◯ Diin la'aan
- ◯ Islam
- ◯ Kristen
- ◯ Hindu
- ◯ Budha

For peer review only

◯ Mid kale (Fadlan qeex) ____

5. Jinsiyadaada maxeey tahay? (waad sheegi kartaa wax ka badan hal)

____

6. Luqadee uugu hadashaa si aad u fiican nolol maalmeedkaada?

____

Labada su'aalo ee soo socda waxa laguugu weydiinayaa heerka luqadaada Ingiriisiga.
6a. Si fiican ayaan Ingirisiga u fahmayaa

◯ Aad ayaan uuga soo horjeedaa1

◯ Waan diidanahay 2

◯ Mana kuugu raacsani mana diidani 3

◯ Waan kuugu raacsanahay 4

◯ Aad ayaan kuugu raacsanahay 5

6b. Si fiican ayaan Ingiriisiga uugu hadli karaa

◯ Maya haba yaraatee 1

◯ Kis yar 2

◯ Kistoo yar 3

◯ Xoogaa 4

◯ In aad u badan 5

6. Waa maxay heerka kuugu sareeyo ee waxbarashada?

◯ Ma heysto waxbarasho rasmi ah (waxaan ka qeybgalay dugsiga, fasalka ESOL)

◯ Dugsiga sare (tusale ahaan NCEA,dibloomada IB, shahaadada dugsiga oo dibada)

◯ Shahaadada heerka sadexaad (tusaale shahaado, Diblooma ama shahaadda ganacsiga - koorso 3 sano ka ayar)

For peer review only - http://bmjopen.bmj.com/site/about/guidelines.xhtml

○ Shahaadada bajalarka (koorsada 3-4 sanno)

○ Shahaadada jaamacada wixi ka sareeya bajalarka (dibloma PG, Mastarka, Dhaqtarnimada)

7. Heerka waxbarashada ama shaqadaada hada (Fadlan muuji mid kastoo ku quseya)

☐ Waxaan bartaa waqti buuxo

☐ Waxaan bartaa waqti barkii

☐ Baagamuudo

☐ Shaqo waqti buuxo (Full-time)

☐ Shaqo waqti barkii (Part-time)

☐ Iskii u shaqeysta waqti buuxa

☐ Full-time self employed

☐ Fasaxa waalidnimada (umusha) oo lacagteydana ii socoto

☐ Waalid guriga jooga (lacag la'aan)

☐ Ka fariistay

☐ Wax kale (fadlan noo sheeg) ____

8. Shaqada

____

9. Dakhliga qoyska ee bil walba (sarifka/lacagaha wadanakaada)

____

9a. Marka la isla barbardhigo dakhliga dhexdhexaadka (isku celceliska), sidee ayaad u qiimeyn lahayd dakhliga qoyskaada?

○ Wuu ka hooseeya dhexdhexaadka

○ Dhexdhexaad (Waa isku celcelis)

For peer review only - http://bmjopen.bmj.com/site/about/guidelines.xhtml

○ Wuu ka sareeyaa dhexdhexaadka

10. Miyaad hadda ku jirtaa bandoow ama laguugu amray in aad guriga joogtid?

○ Haa

○ Maya

11. Talaalka COVID-19 maqaadatay?

○ Haa

○ Maya

11a. Hadii aadan Willi is talaalin, ma jeclaan lahayd in aad is talaashid mustaqbalka?

○ Haa

○ Maya

12. Muuji baayaan walba ee soo socda heerka isbadal ee COVID-19 ku sababay noloshaada, adiga oo isticmaalaaya qiyaasta soo socoto. Halkan waxaa lagu soo koobay dhibaatooyinka ay dadka qaar kala kulmeen COVID-19: (Fadlan noo sheeg intaa kartid)

☐ Masiibo dabiici ah (sida fatahaada, dhulgariirka iwm)

☐ Ku noolaashada dhul dagaal kasocda ama goob jooga qilaafka militariga

☐ Caruurta ka yar 16 oo rafaad kusoo koray (sida dayacaada, xoogsheegsiga, dilka iyo fara xumeeyn)

☐ Dil ama kofsi 16 jir ka weyn

☐ Dhaawac weyn oo jirka

☐ Kuwa kale — fadlan Sii faahfaahi (hadii aad rabtid)

____

☐ Waxaan oo dhan ma aha

For peer review only - http://bmjopen.bmj.com/site/about/guidelines.xhtml

Covid Psychosocial Impacts Scale

Saameynta Shaqsiga

Bayaanka soo socda, fadlan kaga jawaab Haa/Maya haddii adiga kuugu dhacday arintaan Covid-19 dartiis. Hadday Haa tahay, fadlan sheeg xaddiga walaaca ay tani ku gaarsiisay adigoo hoos ku qayaaseysid lambarka ku habboon.

1. Ma qabtaa xaalad caafimaad oo kaa dhigi karta mid u nugul Covid-19?

- **Maya** 0
- **Haa** Walaac malahan haba yaraatee 1
- **Haa** Walaac yar 2
- **Haa** Walaac dhexdhexaad ah 3
- **Haa** Walaac badan 4
- **Haa** Walaac tiro badan 5

2. Maka cabsatay inaad la kulantid qof qaba Covid-19?

- **Maya** 0
- **Haa** Walaac malahan haba yaraatee 1
- **Haa** Walaac yar 2
- **Haa** Walaac dhexdhexaad ah 3
- **Haa** Walaac badan 4
- **Haa** Walaac tiro badan 5

3. Ma kulatahay in uu COVID-19 kuugu dhacay?

- **Maya** 0
- **Haa** Walaac malahan haba yaraatee 1
- **Haa** Walaac yar 2
- **Haa** Walaac dhexdhexaad ah 3

For peer review only - http://bmjopen.bmj.com/site/about/guidelines.xhtml

◯ **Haa** Walaac badan 4

◯ **Haa** Walaac tiro badan 5

3a. Waligaa malaaga helay COVID-19?

◯ **Maya** 0

◯ **Haa** Walaac malahan haba yaraatee 1

◯ **Haa** Walaac yar 2

◯ **Haa** Walaac dhexdhexaad ah 3

◯ **Haa** Walaac badan 4

◯ **Haa** Walaac tiro badan 5

3b. Miyaad leedahay astaamaha COVID-19 oo joogta ah?

◯ **Maya** 0

◯ **Haa** Walaac malahan haba yaraatee 1

◯ **Haa** Walaac yar 2

◯ **Haa** Walaac dhexdhexaad ah 3

◯ **Haa** Walaac badan 4

◯ **Haa** Walaac tiro badan 5

Sameeynta Qoyska

Bayaanka soo socda, fadlan kaga jawaab Haa/Maya haddii arintaan ku dhacday dadka qoyskaada Covid-19 dartiis. Hadday Haa tahay, fadlan sheeg xaddiga walaaca ay tani ku gaarsiisay adigoo hoos ku qayaaseysid lambarka ku habboon.

1. Qof ka mid ah qoyskaada ma jiraa oo u nugul Covid-19 maadaamo ey jirto xaalad caafimaad?

◯ **Maya** 0

◯ **Haa** Walaac malahan haba yaraatee 1

For peer review only - http://bmjopen.bmj.com/site/about/guidelines.xhtml

For peer review only

- **Haa** Walaac yar 2
- **Haa** Walaac dhexdhexaad ah 3
- **Haa** Walaac badan 4
- **Haa** Walaac tiro badan 5

2. Ma dareensantahay in qof qoyskaada ka mid ah halis u yahay inuu la kulmo qof qaba Covid-19 markuu shaqada joogo?

- **Maya** 0
- **Haa** Walaac malahan haba yaraatee 1
- **Haa** Walaac yar 2
- **Haa** Walaac dhexdhexaad ah 3
- **Haa** Walaac badan 4
- **Haa** Walaac tiro badan 5

3. Ma kulatahay in qof qoyskaada soke ka mid ah in uu ku dhacay COVID-19?

- **Maya** 0
- **Haa** Walaac malahan haba yaraatee 1
- **Haa** Walaac yar 2
- **Haa** Walaac dhexdhexaad ah 3
- **Haa** Walaac badan 4
- **Haa** Walaac tiro badan 5

3a. Ma jiraa qof qoyskaada soke ka mid ah oo laga helay COVID-19?

- **Maya** 0
- **Haa** Walaac malahan haba yaraatee 1

For peer review only

For peer review only - http://bmjopen.bmj.com/site/about/guidelines.xhtml

- **Haa** Walaac yar 2
- **Haa** Walaac dhexdhexaad ah 3
- **Haa** Walaac badan 4
- **Haa** Walaac tiro badan 5

3b. Majiraa qof qoyskaada soke ka mid ah ee leh astaamaha COVID-19 oo joogta ah?

- **Maya** 0
- **Haa** Walaac malahan haba yaraatee 1
- **Haa** Walaac yar 2
- **Haa** Walaac dhexdhexaad ah 3
- **Haa** Walaac badan 4
- **Haa** Walaac tiro badan 5

3c. Majiraa qof qoyskaada soke ah ee u geeriyooday/dhintay COVID-19?

- **Maya** 0
- **Haa** Walaac malahan haba yaraatee 1
- **Haa** Walaac yar 2
- **Haa** Walaac dhexdhexaad ah 3
- **Haa** Walaac badan 4
- **Haa** Walaac tiro badan 5

4. Majiraan dad qoyskaada soke ka mid ah ee dibada ku maqan oo aad uuga walwalsantahay in COVID-19 uu ku dhoco?

- **Maya** 0
- **Haa** Walaac malahan haba yaraatee 1

For peer review only

For peer review only - http://bmjopen.bmj.com/site/about/guidelines.xhtml

- **Haa** Walaac yar 2
- **Haa** Walaac dhexdhexaad ah 3
- **Haa** Walaac badan 4
- **Haa** Walaac tiro badan 5

5. Ma leedahay qoyska dhow dibedda ku sugan oo aadan awoodin inaad booqato ama aan kusoo booqan Karin adiga?

- **Maya** 0
- **Haa** Walaac malahan haba yaraatee 1
- **Haa** Walaac yar 2
- **Haa** Walaac dhexdhexaad ah 3
- **Haa** Walaac badan 4
- **Haa** Walaac tiro badan 5

Shaqo

Bayaanka soo socda, fadlan kaga jawaab Haa/Maya/Ma i qusayso haddii adiga ama qof kula nool ku dhacday Covid-19 dartiis. Hadday Haa tahay, fadlan sheeg xaddiga walaaca ay tani ku gaarsiisay adigoo hoos ku qayaaseysid lambarka ku habboon.

1. Ma idinkujiraa qoyskiina qof loo tixgaliyay in uu yahay shaqaalaha daruuriga (muhiimka ah) (shaqaalaha daryeelka caafimaadka, fulinta sharciga, adeegyada degdegga ah, kuwa bixiyaha adeegyada daruuriga ah)?

- **Maya** 0
- **Haa** Walaac malahan haba yaraatee 1
- **Haa** Walaac yar 2
- **Haa** Walaac dhexdhexaad ah 3
- **Haa** Walaac badan 4
- **Haa** Walaac tiro badan 5

For peer review only - http://bmjopen.bmj.com/site/about/guidelines.xhtml

2. Isbadal weyn ma ku yimid saacadaha aad adiga ama qof qoyskaada ka tirsan aad shaqeyn jirteen?

- **Maya** 0
- **Haa** Walaac malahan haba yaraatee 1
- **Haa** Walaac yar 2
- **Haa** Walaac dhexdhexaad ah 3
- **Haa** Walaac badan 4
- **Haa** Walaac tiro badan 5

3. Ma dhacday in aad adiga ama qof qoyskaada katirsan in aad ku qasbanaateen in goobtii shaqada isku badashay guriga (guriga adigoo joogta sii wadid shaqadii)?

- **Maya** 0
- **Haa** Walaac malahan haba yaraatee 1
- **Haa** Walaac yar 2
- **Haa** Walaac dhexdhexaad ah 3
- **Haa** Walaac badan 4
- **Haa** Walaac tiro badan 5

4. Majiraa qof qoyskaada ka mid ah ee ku qasbanaaday in uu shaqada badasho?

- **Maya** 0
- **Haa** Walaac malahan haba yaraatee 1
- **Haa** Walaac yar 2
- **Haa** Walaac dhexdhexaad ah 3
- **Haa** Walaac badan 4
- **Haa** Walaac tiro badan 5

For peer review only - http://bmjopen.bmj.com/site/about/guidelines.xhtml

5. Majiraa qof qoykaada ka tirsan ee shaqadiisii waayay?

- **Ma i qusayso**
- **Maya** 0
- **Haa** Walaac malahan haba yaraatee 1
- **Haa** Walaac yar 2
- **Haa** Walaac dhexdhexaad ah 3
- **Haa** Walaac badan 4
- **Haa** Walaac tiro badan 5

6. Ganacsi/bisinis aad adiga ama qof kula nool lahaa am aad ka shaqeeyneeyseen miyaa halis u ah inuu sii socon waayo?

- **Ma i qusayso**
- **Maya** 0
- **Haa** Walaac malahan haba yaraatee 1
- **Haa** Walaac yar 2
- **Haa** Walaac dhexdhexaad ah 3
- **Haa** Walaac badan 4
- **Haa** Walaac tiro badan 5

Dakhliga

Bayaanka soo socda, fadlan kaga jawaab Haa/Maya haddii uu qoyskaada quseeya Covid-19 dartiis. Hadday Haa tahay, fadlan sheeg xaddiga walaaca ay tani ku gaarsiisay adigoo hoos ku qayaaseysid lambarka ku habboon.

1. Dhaqaalaha reerkaada aad miyuu hoos u dhacay?

- **Maya** 0
- **Haa** Walaac malahan haba yaraatee 1

For peer review only - http://bmjopen.bmj.com/site/about/guidelines.xhtml

- Haa Walaac yar 2
- Haa Walaac dhexdhexaad ah 3
- Haa Walaac badan 4
- Haa Walaac tiro badan 5

2. Qoyskiina miyaad awoodi wayseen inaad bixisaan biilasha ama adeega cuntada?

- **Maya** 0
- **Haa** Walaac malahan haba yaraatee 1
- **Haa** Walaac yar 2
- **Haa** Walaac dhexdhexaad ah 3
- **Haa** Walaac badan 4
- **Haa** Walaac tiro badan 5

Xiriika

Bayaanka soo socda, fadlan kaga jawaab Haa/Maya haddii adiga kuugu dhacday arintaan Covid-19 dartiis. Hadday Haa tahay, fadlan sheeg xaddiga walaaca ay tani ku gaarsiisay adigoo hoos ku qayaaseysid lambarka ku habboon. Covid-19 dartiis…..

1. Dhibaatada qoyska miyeey sii adkaatay?

- **Maya** 0
- **Haa** Walaac malahan haba yaraatee 1
- **Haa** Walaac yar 2
- **Haa** Walaac dhexdhexaad ah 3
- **Haa** Walaac badan 4
- **Haa** Walaac tiro badan 5

2. Xiriirkii aad la lahayd asxaabtaada miyuu soo yaraaday (si toos ah ama dhinaca qadka)?

For peer review only - http://bmjopen.bmj.com/site/about/guidelines.xhtml

- **Maya** 0
- **Haa** Walaac malahan haba yaraatee 1
- **Haa** Walaac yar 2
- **Haa** Walaac dhexdhexaad ah 3
- **Haa** Walaac badan 4
- **Haa** Walaac tiro badan 5

Isbeddelka ku yimid nolol maalmeedka iyo dabeecadaha

Bayaanka soo socda, fadlan kaga jawaab Haa/Maya haddii adiga kuugu dhacday arintaan Covid-19 dartiis. Hadday Haa tahay, fadlan sheeg xaddiga walaaca ay tani ku gaarsiisay adigoo hoos ku qayaaseysid lambarka ku habboon. Covid-19 dartiis.....

1. Cabuqa Korona (Covid-19) miyuu isbadal ku keenay nolol maalmeedkaada (sida hurdada, jimicsiga, firaaqada, hiwaayadaha aad xiiseeysid)?

- **Maya** 0
- **Haa** Walaac malahan haba yaraatee 1
- **Haa** Walaac yar 2
- **Haa** Walaac dhexdhexaad ah 3
- **Haa** Walaac badan 4
- **Haa** Walaac tiro badan 5

2. Miyay taasi sababtay inaad dib u dhigto ama iska dhaaftid inaad raadsato daryeel caafimaad mar ad adiga ama qof qoyskaada u baahateen?

- **Maya** 0
- **Haa** Walaac malahan haba yaraatee 1
- **Haa** Walaac yar 2
- **Haa** Walaac dhexdhexaad ah 3

For peer review only - http://bmjopen.bmj.com/site/about/guidelines.xhtml

○ **Haa** Walaac badan 4

○ **Haa** Walaac tiro badan 5

3. Tani miyay yareeysay ka-qayb-qaadashadaada howlaha diinta (shaqsi ahaan ama khadka tooska ah)?

○ **Ma i qusayso**

○ **Maya** 0

○ **Haa** Walaac malahan haba yaraatee 1

○ **Haa** Walaac yar 2

○ **Haa** Walaac dhexdhexaad ah 3

○ **Haa** Walaac badan 4

○ **Haa** Walaac tiro badan 5

4. Miyey jireen isbeddelo kale oo weyn oo ku yimid nidaamka/nolol maalmeedka?

○ **Maya** 0

○ **Haa (Haddii ay sidaas tahay fadlan sheeg)** 1 ____

Hadday Haa tahay, fadlan sheeg xaddiga walaaca ay tani ku gaarsiisay adigoo hoos ku qayaaseysid lambarka ku habboon.

○ Walaac malahan haba yaraatee 1

○ Walaac yar 2

○ Walaac dhexdhexaad ah 3

○ Walaac badan 4

○ Walaac tiro badan 5

Soo bandhigista macluumaadka ku saabsan Covid-19

1. Xageed kaheshaa warbixinta ku saabsan Covid-19?
(Fadlan qeex mid kasto ad isticmaashid)

For peer review only - http://bmjopen.bmj.com/site/about/guidelines.xhtml

- [ ] News/wararka
- [ ] Facebook / Twitter
- [ ] Websites/boga caafimaadka
- [ ] Hadal isgaarsiis
- [ ] Meela kale (Fadlan qeex) ____

2. Culeys ilaa intee ayuu wararka ku saabsan Covid-19 kuu geystay?

- ◯ Walaac malahan haba yaraatee 1
- ◯ Walaac yar 2
- ◯ Walaac dhexdhexaad ah 3
- ◯ Walaac badan 4
- ◯ Walaac tiro badan 5

Isbeddelada ku yimid Covid-19 dartiis

Arimaha soo socda fadlan sheeg xaddiga walaaca ay tani ku gaarsiisay adigoo hoos ku qayaaseysid lambarka ku habboon. Isbarbardhiga hadda iyo wixii ka horeeyay Covid-19---

1. Sideed u qiimeyn lahayd heerka walaacaaga shaqsi ahaan?

- ◯ Walaac yar 1
- ◯ Xoogaa walaac yar 2
- ◯ Islameeshii 3
- ◯ Walwal batay 4
- ◯ Walaaca oo aad u batay 5

2. Maxaad ku qiimeyn lahayd walaaca guud ee reerkaada/gurigaada?

- ◯ Walaac yar 1

For peer review only - http://bmjopen.bmj.com/site/about/guidelines.xhtml
For peer review only

◯ Xoogaa walaac yar 2

◯ Islameeshii 3

◯ Walwal batay 4

◯ Walaaca oo aad u batay 5

3. Sideed u qiimeyn lahayd ladnaantaada nafsiyeed?

◯ Aad uuga fiican sidii hore 1

◯ Sidii hore ka fiican 2

◯ Sideedii 3

◯ Sidii ka daran 4

◯ Aad uga daran 5

4. Sideed u qiimeyn laheyd wanaagsanaanta nafsiyeed ee dadka qoyskaaga?

◯ Aad ufiican 1

◯ Fiican 2

◯ Ku saabsan isla mid 3

◯ Ka xun 4

◯ Aad uga xun 5

For peer review only - http://bmjopen.bmj.com/site/about/guidelines.xhtml

K-10

Tobanka su'aalo ee xiggo waxay wax ka weydiinayaan sida aad dareemeysay 4 isbuuc ee la soo dhaafay. Su'aal walba, tilmaam intajeer aad dareentay qaabkaas.

	Marnaba 1	Mar dhif 2	Mar mar 3	Inta badan 4	Mar kasta 5
1. Afartii isbuuc ee la soo dhaafay, intee jeer ayaad dareentay daal oo aan lahayn sabab wanaagsan?	○	○	○	○	○
2. Afartii isbuuc ee la soo dhaafay, intee jeer ayaad dareentay walwal?	○	○	○	○	○
3. Afartii isbuuc ee la soo dhaafay, intee jeer ayaad dareentay walwal zaa'id ah oo waxba aanan ku dajineyn?	○	○	○	○	○
4. Afartii isbuuc ee la soo dhaafay, intee jeer ayaad dareentay rajo la' aan?	○	○	○	○	○
5. Afartii isbuuc ee la soo dhaafay, intee jeer ayaad dareentay nasasho la'aan ama samir la'aan?	○	○	○	○	○
6. Afartii isbuuc ee la soo dhaafay, intee jeer ayaad dareentay nasasho la'aan oo aadan fadhin karin?	○	○	○	○	○
7. Afartii isbuuc ee la soo dhaafay, intee jeer ayaad dareentay murugo?	○	○	○	○	○
8. Afartii isbuuc ee la soo dhaafay, intee jeer ayaad dareentay in wax walba ay dadaal/halgan ahaayeen?	○	○	○	○	○
9. Afartii isbuuc ee la soo dhaafay, intee jeer ayaad dareentay murugo badan oo waxba aanan ku farxad gelineynin?	○	○	○	○	○
10. Afartii isbuuc ee la soo dhaafay, intee jeer ayaad dareentay qiimo la'aan?	○	○	○	○	○

For peer review only

For peer review only - http://bmjopen.bmj.com/site/about/guidelines.xhtml

PCL-5

Hoos waa liiska dhibaatooyinka oo dadka mararka qaar ay qabaan oo jawaab u ah waaya aragnimada walbahaarka badan. Fadlan si taxadar ah u aqri dhibaato walba kadibna tilmaam inta ay ku dhibtay dhibaatadaas bishii la soo dhaafay. Bisha la soo dhaafay, intee ayay ku dhibtay:

	Maya gabi ahan 0	Xooga yar 1	Dhexaad 2	Ilaa xad 3	Xad dhaaf ah 4
1. Soo nonoqoshada xasuusta aan la rabin oo khibradaha argagaxa leh?	○	○	○	○	○
2. Soo nonoqoshada riyooyinka jahwareerka ah ee ku saabsan khibradaha argagaxa leh?	○	○	○	○	○
3. Dareen lama filaan ah ama u dhaqanka sidii ineey hada khibradii murugada lahayd dhaceysa markale (sidii ineey hada dhaceeyso oo kale)?	○	○	○	○	○
4. Dareenka murugada aadka ah markii wax ku soo xasuusiyaan waaya aragnimada murugada leh?	○	○	○	○	○
5. Falcelinada jirka xoogan markii wax ku soo xasuusiyaan waaya aragnimadii murugada lahyd (tusaale ahaan, garaaca wadnaha, neefsashada oo dhib noqota iyo dhididka)?	○	○	○	○	○
6. Ka fogaanshaha xasuusta, afkaaraha, ama dareemida la xiriirto waaya aragnimada/khibradaha murugada leh?	○	○	○	○	○
7. Ka fogaanshaha wax kastoo kusoo xasuusiya walbahaarkii (tusaala ahaan, dadka, meelaha, wadasheekeysiga, shaqooyinka, waxyaabaha ama xaaladaha)?	○	○	○	○	○
8. Miyaad dhib ku qabtaa inaa xasuusatid qeyba muhiim ah ee khibradihii walbahaarka lahaa?	○	○	○	○	○
9. Ka qabida aaminaad xun oo xoogan nafsadaada, dadka kale, ama caalamka (tusaale ahaan, lahaanshaha afkaaraha sida; Waan xummahay, wax baa iga qaldan,	○	○	○	○	○

For peer review only

For peer review only - http://bmjopen.bmj.com/site/about/guidelines.xhtml

majiro qof la aamini karo, caalamka oo dhan waa halis)?

10. Ku eedeynta nafsadaada ama qof kale ee khibradihii walbahaarka lahaa ama wixii dhacay markaas kadib?

11. Lahaanshaha dareen xun ee xoogan sida cabsida, naxdinta, xanaaqa, dambiilenimada, ama ceeb?

12. Ka xiisadhicida howlihii aad jeclaan jirtay?

13. Dareemida ka fogaanshaha ama ka go'ida dadka kale?

14. Dhibaato ma ku qabtaa inaad dareento dareen wanaagsan (tusaale ahaan, inaad dareento farxad ama aad jeclaato dadka kuu dhow)?

15. Anshax xumo, xanaaqa, ama ku dhaqmida si dagaal ah?

16. Halis is galis badan ama sameeynta waxyaabaha kuugu sababi karo dhibaato?

17. Ma dareentaa "feejignaan cajiib ah” ama fiirin badan ama illaalo?

18. Miyaad dareentaa xasilaad la'aan ama si sahlan inaad u naxdo?

19. Ma kuugu adagtahay inaad diirad saartid/xogsaartid?

20. Ma kuugu adagtahay ineey hurdo kaa soo dhacdo ama inaad sii hurudo?

For peer review only

For peer review only - http://bmjopen.bmj.com/site/about/guidelines.xhtml

WHO-5

Fadlan noo tilmaam shanta arimood ee soo socda sida ugu dhow uu dareenkaaga ahaa labadii isbuuc ee la soo dhaafay. Labadii isbuuc ee lasoo dhaafay

	**Marwalba** 5	**Intabadan wakhtiga** 4	**Inkabadan kalabar wakhtiga** 3	**Inkayar kalabar wakhtiga** 2	**Marmar wakhtiga** 1	**Marnaba** 0
1. Waxaan dareemay farxad iyo hami sare.	○	○	○	○	○	○
2. Waxaan dareemay daganaan iyo xasilooni.	○	○	○	○	○	○
3. Waxaan dareemay firfircooni iyo awoodbadan.	○	○	○	○	○	○
4. Waxaan soo kacay anigoo dareemaya nasasho iyo daganaan.	○	○	○	○	○	○
5. Maalintayda oo dhan waxaa buuxiyay waxyalo ixiiso galiya.	○	○	○	○	○	○

For peer review only

PTGI

Muuji baayaan walba ee soo socda heerka isbadal ee Maarso 15-keedii ku sababay noloshaada, adoo isticmaalaaya qiyaasta soo socoto.

	Marnaba 0	Wax yar 1	Xoogaa Roon 2	Dhexaad 3	In badan 4	Wax badan 5
1. Waxaan badelay muhiimadeyda ku saabsan waxa nolosha muhiim u ah.	○	○	○	○	○	○
2. Waxaan qadarin weyn u leeyahay qiimaha noloshayda.	○	○	○	○	○	○
3. Waxaan hormariyay xiiso/dana cusub.	○	○	○	○	○	○
4. Waxaan dareemayaa isku kalsooni.	○	○	○	○	○	○
5. Waxaan si fiican uu fahamsanahay arrimaha diinta.	○	○	○	○	○	○
6. Waxaan si cad u arkaa inaan ku xisaabtami karo dadka waqtiyada dhibaatada.	○	○	○	○	○	○
7. Waxaan nolosheyda u aasaasay wado cusub.	○	○	○	○	○	○
8. Waxaan dareensanahay inaan dadka aad uugu dhawahay.	○	○	○	○	○	○
9. Waxaan diyaar u ahay inaan muujiyo dareenkeyga.	○	○	○	○	○	○
10. Waxaan si fiican u ogahay in aan wax ka qaban karo dhibaatoyinka.	○	○	○	○	○	○
11. Waxaan awooda in aan noloshayda wax fiican ku sameeyo.	○	○	○	○	○	○
12. Waxaan si fiican u awoodaa inaan aqbalo siday wax u dhacayaan.	○	○	○	○	○	○

For peer review only

For peer review only - http://bmjopen.bmj.com/site/about/guidelines.xhtml

13. Waxaan si fiican uga mahadcelin karaa maalin kasta.	○	○	○	○	○	○
14. Fursado cusub ayaa la heli karaa oo awal jirin.	○	○	○	○	○	○
15. Waxaan u qabaa naxariis badan dadka kale.	○	○	○	○	○	○
16. Waxaan dadaal badan geliya cilaaqaadkeyga.	○	○	○	○	○	○
17. Waxay u badan tahay inaan isku dayo inaan beddelo waxyaabaha u baahan badal.	○	○	○	○	○	○
18. Waxaan leeyahay caqiido diineed oo xoog badan.	○	○	○	○	○	○
19. Waxaan ogaaday inaan ka xoog badanahay sidaan u maleeynayay.	○	○	○	○	○	○
20. Waxaan bartay ineey dadka aad u wanaagsan yihiin.	○	○	○	○	○	○
21. Waxaan si fiican u qirraa inaan u baahankaro dadka kale.	○	○	○	○	○	○

For peer review only

For peer review only - http://bmjopen.bmj.com/site/about/guidelines.xhtml

International Study - Turkiye (Turkish)

Sociodemographic Information

1. Cinsiyet

○ Erkek

○ Kadın

2. Yaş

____

3. Medeni Hali

○ Bekar

○ İlişkisi var

○ Evli

○ Ayrılmış

○ Boşanmış

○ Dul

4. Dininiz nedir?

○ Din yok

○ İslâm

○ Hıristiyanlık

○ Hinduizm

○ Budizm

○ Diğer (lütfen belirtiniz) ____

For peer review only

5. Eğitim düzeyiz nedir?(En son almış olduğunuz derece)?

- Okur Yazar
- İlkokul Mezunu
- Ortaokul Mezunu
- Lise Mezunu
- Üniversite Öğrencisi
- Önlisans Mezunu
- Lisans Mezunu
- Yüksek Lisans Mezunu
- Doktora Mezunu

6. Mevcut Çalışma veya iş durumunuz (Birden fazla işaretleyebilirsiniz.)

- Öğrenci
- Çalışmıyor/İş Arıyor
- Tam zamanlı maaşlı çalışma
- Yarı zamanlı maaşlı çalışma
- Tam zamanlı serbest meslek
- Yarı zamanlı serbest meslek
- Ücretli ebeveyn izninde
- Evde ebeveyn yada ücretsiz ebeveyn izninde
- Emekli
- Diğer (Lütfen Belirtin) ____

For peer review only

For peer review only - http://bmjopen.bmj.com/site/about/guidelines.xhtml

7. Mesleğiniz

____

8. Aylık hane geliri (yerel para biriminde)

____

9. Ortalama gelirle karşılaştırıldığında, hanenizin gelirini nasıl değerlendirirsiniz?

○ Ortalamanın altında

○ Ortalama

○ Ortalamanın üstünde

10. Şu anda karantinada ya da sokağa çıkma yasağında mısınız?

○ Evet1

○ Hayır 0

11. Covid-19 aşısı oldunuz mu?

○ Evet1

○ Hayır 0

11a. Aşı olmadıysanız, gelecekte Covid-19 aşısı olmak ister misiniz?

○ Evet1

○ Hayır 0

12. Bazı insanların geçmişte maruz kaldıkları diğer travmatik olayların yeni sorunlarla başa çıkmayı kolaylaştırabilecek ya da zorlaştırabilecek olduğunun farkındayız.

Covid-19 salgınından önce aşağıdakilerden herhangi birine şahit oldunuz mu veya herhangi birini yaşadınız mı?: **(Lütfen uygun olanları işaretleyin)**

☐ Doğal afet (sel, deprem, vs.)

For peer review only

☐ Savaş bölgesinde yaşamak veya askeri çatışmaya maruz kalmak

☐ 16 yaş öncesi çocukluk sıkıntısı (mesela ihmal, zorbalık, fiziksel veya cinsel saldırı, vs.)

☐ 16 yaş sonrası fiziksel veya cinsel saldırı

☐ Ciddi fiziksel kazalar

☐ Diğer – isteğe bağlı – bunun ne olduğunu açıklayabilir misiniz lütfen?
____

☐ Yukarıdakilerin hiçbiri

For peer review only

For peer review only - http://bmjopen.bmj.com/site/about/guidelines.xhtml

Covid Psychosocial Impacts Scale

Kişisel etki

Aşağıdaki ifadeler için, Covid-19 döneminde sizde böyle bir durum gerçekleştiyse lütfen Evet / Hayır ile yanıt verin. Evet ise, lütfen aşağıdaki ölçekte bunun sizde neden olduğu stres miktarını belirtin.

1. Covid-19'dan daha olumsuz etkilenmenize neden olabilecek bir sağlık probleminiz var mı (örn. Kalp hastalığı, nefes almada zorluk, zayıf bağışıklık sistemi, veya kanser)?

- **Hayır** 0
- **Evet** Hiç stresli değil 1
- **Evet** Biraz stres 2
- **Evet** Orta derecede stres 3
- **Evet** Oldukça fazla stres 4
- **Evet** Aşırı derecede stres 5

2. Covid-19 pozitif biriyle temas riski altında olduğunuzu hissettiniz mi?

- **Hayır** 0
- **Evet** Hiç stresli değil 1
- **Evet** Biraz stres 2
- **Evet** Orta derecede stres 3
- **Evet** Oldukça Fazla stres 4
- **Evet** Aşırı derecede stres 5

3. Covid-19 geçirdiğinizi düşünüyor musunuz?

- **Hayır** 0
- **Evet** Hiç stresli değil 1
- **Evet** Biraz stres 2

For peer review only - http://bmjopen.bmj.com/site/about/guidelines.xhtml

◯ **Evet** Orta derecede stres 3

◯ **Evet** Oldukça fazla stres 4

◯ **Evet** Aşırı derecede stres 5

3a. Covid-19 tanısı aldınız mı?

◯ **Hayır** 0

◯ **Evet** Hiç stresli değil 1

◯ **Evet** Biraz stres 2

◯ **Evet** Orta derecede stres 3

◯ **Evet** Oldukça fazla stres 4

◯ **Evet** Aşırı derecede stres 5

3b. Devam eden Covid-19 semptomlarınız var mı?

◯ **Hayır** 0

◯ **Evet** Hiç stresli değil 1

◯ **Evet** Biraz stres 2

◯ **Evet** Orta derecede stres 3

◯ **Evet** Oldukça fazla stres 4

◯ **Evet** Aşırı derecede stres 5

Aile etkisi

Aşağıdaki ifadeler için, Covid-19 döneminde yakın aile üyelerinizde böyle bir durum gerçekleştiyse lütfen Evet / Hayır ile yanıt verin. Evet ise, lütfen aşağıdaki ölçekte bunun sizde neden olduğu stres miktarını belirtin.

1. Herhangi bir yakın aile üyesinin Covid-19'dan daha olumsuz etkilenmelerine neden olabilecek bir sağlık problemi var mı? (örn. Kalp hastalığı, nefes almada zorluk, zayıf bağışıklık sistemi, veya kanser)?

For peer review only

- **Hayır** 0
- **Evet** Hiç stresli değil 1
- **Evet** Biraz stres 2
- **Evet** Orta derecede stres 3
- **Evet** Oldukça fazla stres 4
- **Evet** Aşırı derecede stres 5

2. Pandemi sürecinde herhangi bir yakın aile üyesinin, Covid-19 pozitif olan biri ile temas riski altında olduğunu düşündünüz mü/düşünüyor musunuz?

- **Hayır** 0
- **Evet** Hiç stresli değil 1
- **Evet** Biraz stres 2
- **Evet** Orta derecede stres 3
- **Evet** Oldukça fazla stres 4
- **Evet** Aşırı derecede stres 5

3. Yakın aile üyelerinizden herhangi birinin Covid-19 geçirdiğini düşünüyor musunuz?

- **Hayır** 0
- **Evet** Hiç stresli değil 1
- **Evet** Biraz stres 2
- **Evet** Orta derecede stres 3
- **Evet** Oldukça fazla stres 4
- **Evet** Aşırı derecede stres 5

For peer review only

3a. Yakın aile üyelerinin herhangi biri Covid-19 tanısı aldı mı?

- **Hayır** 0
- **Evet** Hiç stresli değil 1
- **Evet** Biraz stres 2
- **Evet** Orta derecede stres 3
- **Evet** Oldukça fazla stres 4
- **Evet** Aşırı derecede stres 5

3b. Yakın aile üyesi/üyelerinde devam eden Covid-19 semptomları var mı?

- **Hayır** 0
- **Evet** Hiç stresli değil 1
- **Evet** Biraz stres 2
- **Evet** Orta derecede stres 3
- **Evet** Oldukça fazla stres 4
- **Evet** Aşırı derecede stres 5

3c. Yakın bir aile üyesi/üyeleri Covid-19 nedeniyle vefat etti mi?

- **Hayır** 0
- **Evet** Hiç stresli değil 1
- **Evet** Biraz stres 2
- **Evet** Orta derecede stres 3
- **Evet** Oldukça fazla stres 4
- **Evet** Aşırı derecede stres 5

For peer review only

4. Yurtdışında Covid-19'a yakalanma riski altında olmasından endişe duyduğunuz yakın aile üyeniz var mı?

- **Hayır** 0
- **Evet** Hiç stresli değil 1
- **Evet** Biraz stres 2
- **Evet** Orta derecede stres 3
- **Evet** Oldukça fazla stres 4
- **Evet** Aşırı derecede stres 5

5. Covid-19 pandemisi nedeniyle yurtdışında ziyaret edemediğiniz veya sizi ziyaret edemeyen yakın aile üyeniz var mı?

- **Hayır** 0
- **Evet** Hiç stresli değil 1
- **Evet** Biraz stres 2
- **Evet** Orta derecede stres 3
- **Evet** Oldukça fazla stres 4
- **Evet** Aşırı derecede stres 5

İş

Aşağıdaki ifadeler için, Covid-19 döneminde bu durum sizde veya evinizdeki herhangi birinde gerçekleşmişse (Evet / Hayır / Bana uygun değil) ile yanıt verin. Evet ise, lütfen aşağıdaki ölçekte bunun sizde neden olduğu stres miktarını belirtin.

1. Siz veya evinizdeki herhangi biri temel sektör çalışanı mısınız? (örneğin sağlık hizmetleri, gıda sektörü, acil servisler, temel mal / hizmet sağlayıcıları vb.)?

- **Hayır** 0
- **Evet** Hiç stresli değil 1

For peer review only - http://bmjopen.bmj.com/site/about/guidelines.xhtml

○ **Evet** Biraz stres 2

○ **Evet** Orta derecede stres 3

○ **Evet** Oldukça fazla stres 4

○ **Evet** Aşırı derecede stres 5

2. Covid-19 pandemisi nedeniyle sizin veya evinizdeki herhangi birinin çalışma saatlerinde büyük bir değişiklik oldu mu?

○ **Hayır** 0

○ **Evet** Hiç stresli değil 1

○ **Evet** Biraz stres 2

○ **Evet** Orta derecede stres 3

○ **Evet** Oldukça fazla stres 4

○ **Evet** Aşırı derecede stres 5

3. Covid-19 pandemisi nedeniyle siz veya evinizdeki herhangi biri evden çalışmak zorunda kaldınız mı?

○ **Hayır** 0

○ **Evet** Hiç stresli değil 1

○ **Evet** Biraz stres 2

○ **Evet** Orta derecede stres 3

○ **Evet** Oldukça fazla stres 4

○ **Evet** Aşırı derecede stres 5

4. Covid-19 pandemisi nedeniyle siz veya evinizdeki herhangi biri farklı bir iş türüne geçmek zorunda kaldınız mı?

○ **Hayır** 0

For peer review only - http://bmjopen.bmj.com/site/about/guidelines.xhtml

- **Evet** Hiç stresli değil 1
- **Evet** Biraz stres 2
- **Evet** Orta derecede stres 3
- **Evet** Oldukça fazla stres 4
- **Evet** Aşırı derecede stres 5

5. Covid-19 pandemisi nedeniyle siz veya evinizdeki herhangi biri işini kaybetti mi?

- **Bana uygun değil**
- **Hayır** 0
- **Evet** Hiç stresli değil 1
- **Evet** Biraz stres 2
- **Evet** Orta derecede stres 3
- **Evet** Oldukça fazla stres 4
- **Evet** Aşırı derecede stres 5

6. Covid-19 pandemisi nedeniyle sizin veya evinizdeki bir başkasının kendi işletmesi veya çalıştığı şirket kapanma tehdidi altında mı?

- **Bana uygun değil**
- **Hayır** 0
- **Evet** Hiç stresli değil 1
- **Evet** Biraz stres 2
- **Evet** Orta derecede stres 3
- **Evet** Oldukça fazla stres 4

For peer review only

◯ **Evet** Aşırı derece stres 5

Gelir

Aşağıdaki ifadeler için, Covid-19 döneminde bu durum evinizdeki herhangi birinde gerçekleştiyse (Evet / Hayır) ile yanıt verin. Evet ise, lütfen aşağıdaki ölçekte bunun sizde neden olduğu stres miktarını belirtin.

1. Covid-19 pandemisi nedeniyle hanenizde büyük bir maddi zorluk yaşadınız mı?

◯ **Hayır** 0

◯ **Evet** Hiç stresli değil 1

◯ **Evet** Biraz stres 2

◯ **Evet** Orta derecede stres 3

◯ **Evet** Oldukça fazla stres 4

◯ **Evet** Aşırı derecede stres 5

2. Covid-19 pandemisi nedeniyle hanehalkınız faturalarınızı ödeyebilmeniz veya mutfak masraflarınızı karşılayabilmeniz konusunda büyük bir zorluk yaşadı mı?

◯ **Hayır** 0

◯ **Evet** Hiç stresli değil 1

◯ **Evet** Biraz stres 2

◯ **Evet** Orta derecede stres 3

◯ **Evet** Oldukça fazla stres 4

◯ **Evet** Aşırı derecede stres 5

İlişkiler

Aşağıdaki ifadeler için, Covid-19 döneminde sizde böyle bir durum gerçekleştiyse lütfen Evet / Hayır ile yanıt verin. Evet ise, lütfen aşağıdaki ölçekte bunun sizde neden olduğu stres miktarını belirtin. Covid-19'a yanıt olarak....

For peer review only - http://bmjopen.bmj.com/site/about/guidelines.xhtml

1. Covid-19 döneminde aile üyeleri arasında zorluklar ve gerginlikler arttı mı?

- ◯ **Hayır** 0
- ◯ **Evet** Hiç stresli değil 1
- ◯ **Evet** Biraz stres 2
- ◯ **Evet** Orta derecede stres 3
- ◯ **Evet** Oldukça fazla stres 4
- ◯ **Evet** Aşırı derecede stres 5

2. Covid-19 pandemisi arkadaşlarınızla (fiziksel veya çevrimiçi/online) iletişiminizi azalttı mı?

- ◯ **Hayır** 0
- ◯ **Evet** Hiç stresli değil 1
- ◯ **Evet** Biraz stres 2
- ◯ **Evet** Orta derecede stres 3
- ◯ **Evet** Oldukça fazla stres 4
- ◯ **Evet** Aşırı derecede stres 5

Günlük rutin ve davranışlarda değişiklikler

Aşağıdaki ifadeler için, Covid-19 döneminde sizde böyle bir durum gerçekleştiyse lütfen (Evet / Hayır / Bana uygun değil) ile yanıt verin. Evet ise, lütfen aşağıdaki ölçekte bunun sizde neden olduğu stres miktarını belirtin. Covid-19'a yanıt olarak......

1. Covid-19 pandemisi günlük rutininizde değişikliklere yol açtı mı? (örneğin, uyku, egzersiz, boş zaman, keyif veren aktiviteler veya hobiler)

- ◯ **Hayır** 0
- ◯ **Evet** Hiç stresli değil 1
- ◯ **Evet** Biraz stres 2

For peer review only - http://bmjopen.bmj.com/site/about/guidelines.xhtml

◯ **Evet** Orta derecede stres 3

◯ **Evet** Oldukça fazla stres 4

◯ **Evet** Aşırı derece stres 5

2. Covid-19 pandemisi sizin veya bir aile üyenizin ihtiyaç duyduğu sağlık hizmetine başvurmayı ertelemenize / başvurmaktan kaçınmanıza neden oldu mu?

◯ **Hayır** 0

◯ **Evet** Hiç stresli değil 1

◯ **Evet** Biraz stres 2

◯ **Evet** Orta derecede stres 3

◯ **Evet** Oldukça fazla stres 4

◯ **Evet** Aşırı derecede stres 5

3. Covid-19 pandemisi dini faaliyetlere katılımınızı azalttı mı? (yüz yüze veya çevrimiçi/online)

◯ **Bana uygun değil**

◯ **Hayır** 0

◯ **Evet** Hiç stresli değil 1

◯ **Evet** Biraz stres 2

◯ **Evet** Orta derecede stres 3

◯ **Evet** Oldukça fazla stres 4

◯ **Evet** Aşırı derecede stres 5

4. Covid-19 pandemisi nedeniyle rutininizde başka büyük değişiklikler oldu mu?

◯ **Hayır** 0

For peer review only

- ○ Evet ise lütfen belirtin1 ____

Evet ise, lütfen bunun aşağıdaki ölçekte sizde neden olduğu stres miktarını belirtin.

- ○ Hiç stresli değil 1
- ○ Biraz stres 2
- ○ Orta derecede stres 3
- ○ Oldukça fazla stres 4
- ○ Aşırı derecede stres 5

Covid-19 hakkındaki bilgilere maruz kalma

1. Covid-19 ile ilgili bilgi almak için hangi kaynakları kullanıyorsunuz? (Lütfen uygun olanları işaretleyin.)

- ☐ Haberler
- ☐ Facebook / Twitter
- ☐ Sağlık web siteleri
- ☐ Duyum/Başkalarından duyma
- ☐ Diğerleri (lütfen belirtiniz) ____

2. Covid-19 ile ilgili haberler sizde ne kadar strese neden oluyor?

- ○ Hiç stresli değil 1
- ○ Biraz stres 2
- ○ Orta derecede stres 3
- ○ Oldukça fazla stres 4
- ○ Aşırı derecede stres 5

For peer review only

For peer review only - http://bmjopen.bmj.com/site/about/guidelines.xhtml

Covid-19'dan bu yana değişiklikler

Aşağıdaki ifadeler için size uygun olanı lütfen ilgili ölçekte belirtiniz. Şimdi ile Covid-19 salgını öncesini karşılaştırma---

1. Covid-19 pandemisi öncesi ile kıyasladığınızda salgın sonrası kişisel stres seviyenizi nasıl değerlendirirsiniz?

- ○ Önemli ölçüde daha az stresli 1
- ○ Biraz daha az stresli 2
- ○ Aynı sayılır 3
- ○ Daha stresli 4
- ○ Önemli ölçüde daha fazla stresli 5

2. Covid-19 pandemisi öncesi ile kıyasladığınızda salgın sonrası evinizdeki genel stres düzeyini nasıl değerlendirirsiniz?

- ○ Önemli ölçüde daha az stresli 1
- ○ Biraz daha az stresli 2
- ○ Aynı sayılır 3
- ○ Daha stresli 4
- ○ Önemli ölçüde daha fazla stresli 5

3. Covid-19 pandemisi öncesi ile kıyasladığınızda salgın sonrası kişisel psikolojik iyilik halinizi nasıl değerlendirirsiniz?

- ○ Çok daha iyi 1
- ○ Daha iyi 2
- ○ Aynı sayılır 3
- ○ Daha kötü 4

For peer review only

For peer review only - http://bmjopen.bmj.com/site/about/guidelines.xhtml

- Çok daha kötü 5

4. Covid-19 pandemisi öncesi ile kıyasladığınızda salgın sonrası hanehalkı üyelerinizin genel psikolojik iyilik halini nasıl değerlendirirsiniz?

- Çok daha iyi 1
- Daha iyi 2
- Ayni sayilir 3
- Daha kötü 4
- Çok daha kötü 5

For peer review only

K-10

Aşağıdaki 10 soru son 4 hafta içinde kendinizi nasıl hissettiğinizle ilgilidir. Her soru için, kendinizi o durumda hissettiğiniz zamanın uzunluğunu en iyi şekilde tanımlayan seçeneği seçiniz.

	**Hiçbir zaman 1**	**Kısa bir süre 2**	**Bazen 3**	**Coğu zaman 4**	**Her zaman 5**
1. Son 4 hafta içinde, ne kadar sıklıkla kendinizi sebepsiz yere yorgun hissettiniz?	○	○	○	○	○
2. Son 4 hafta içinde, ne kadar sıklıkla kendinizi gergin hissettiniz?	○	○	○	○	○
3. Son 4 hafta icinde ne kadar sıklıkla sizi hiçbir şeyin sakinleştirmeyeceği kadar gergin hissettiniz?	○	○	○	○	○
4. Son 4 hafta içinde ne kadar sıklıkla kendinizi umutsuz hissettiniz?	○	○	○	○	○
5. Son 4 hafta içinde ne kadar sıklıkla kendinizi tedirgin veya huzursuz hissettiniz?	○	○	○	○	○
6. Son 4 hafta içinde ne kadar sıklıkla yerinde duramayacak kadar huzursuz hissettiniz?	○	○	○	○	○
7. Son 4 hafta içinde ne kadar sıklıkla kendinizi bunalımlı hissettiniz?	○	○	○	○	○
8. Son 4 hafta içinde ne kadar sıklıkla en ufak bir şeyin bile çok çaba gerektirdiğini hissettiniz?	○	○	○	○	○
9. Son 4 hafta içinde ne kadar sıklıkla kendinizi sizi hiçbir şeyin neşelendiremeyeceği kadar üzgün hissettiniz?	○	○	○	○	○
10. Son 4 hafta icinde ne kadar sıklıkla kendinizi değersiz hissettiniz?	○	○	○	○	○

For peer review only

For peer review only - http://bmjopen.bmj.com/site/about/guidelines.xhtml

PCL-5

Aşağıda, bazı kişilerin Covid-19'a tepki olarak yaşadığı sorunların bir listesi bulunmaktadır. Lütfen her bir sorunu dikkatlice okuyun ve geçen ay bu sorundan ne kadar rahatsız olduğunuzu belirtin. Geçen ay içinde aşağıda yer alan durumlar sizi ne ölçüde bunalttı:

	Hiç 0	Çok az 1	Orta derecede 2	Oldukça az 3	Aşırı 4
1. Stresli olayın tekrarlayan, rahatsız eden ve istenmeyen anları sizi ne kadar bunalttı?	○	○	○	○	○
2. Stresli olaya ilişkin tekrarlayan, rahatsız eden rüyalar sizi ne kadar bunalttı?	○	○	○	○	○
3. Aniden stresli olayı sanki gerçekten bir daha yaşıyormuş gibi hissetmek veya davranmak (sanki gerçekten olayın yaşandığı ana geri dönmüş yeniden yaşıyor gibi) sizi ne kadar bunalttı?	○	○	○	○	○
4. Bir şeyler size stresli olayı anımsattığı zaman yaşadığınız üzüntü hissi sizi ne kadar bunalttı?	○	○	○	○	○
5. Bir şeyler size stresli olayı anımsattığı zaman güçlü fiziksel tepkiler vermek (örneğin, kalp çarpıntısı, nefes almada güçlük, terleme gibi) sizi ne kadar bunalttı?	○	○	○	○	○
6. Stresli olayla ilişkili anılardan,düşüncelerden ve duygulardan kaçınmaya çalışmak sizi ne kadar bunalttı?	○	○	○	○	○
7. Stresli olayı anımsatan etraftaki hatırlatıcı şeylerden (örneğin, insanlardan, yerlerden, konuşmalardan, etkinliklerden, nesnelerden veya durumlardan) kaçınmaya çalışmak sizi ne kadar bunalttı?	○	○	○	○	○
8. Stresli olaya ilişkin önemli kısımları hatırlamada yaşanan güçlükler sizi ne kadar bunalttı?	○	○	○	○	○
9. Kendiniz, diğer insanlar veya dünya hakkında güçlü olumsuz düşüncelere sahip olmak (örneğin, kötü biriyim, bende ciddi şekilde yanlış olan bir şeyler var, kimseye güvenilmez, dünya tümüyle tehlikeli bir yerdir gibi düşünceler) sizi ne kadar bunalttı?	○	○	○	○	○

For peer review only

For peer review only - http://bmjopen.bmj.com/site/about/guidelines.xhtml

10. Stresli olay veya bu olayın sonrasında ortaya çıkan durumlar için kendinizi veya bir başkasını suçlamak sizi ne kadar bunalttı?

11. Korku, dehşete kapılma, öfke, suçluluk, veya utanç gibi olumsuz duygular sizi ne kadar bunalttı?

12. Daha önce yapmaktan keyif aldığınız etkinliklere olan ilginizi kaybetmek sizi ne kadar bunalttı?

13. Başka insanlardan uzak veya kopmuş hissetmek sizi ne kadar bunalttı?

14. Olumlu duyguları yaşamak (örneğin, mutluluğu hissetmek vaya size yakın insanlara sevgi dolu hisler duyamamak) sizi ne kadar bunalttı?

15. Asabi davranışlar, öfke patlamaları veya öfkeli hareketler sizi ne kadar bunalttı?

16. Çok fazla risk almak veya size zarar verebilecek şeyler yapmak sizi ne kadar bunaltı?

17. Aşırı tetikte olmak veya temkinli davranmak veya hazırda beklemek sizi ne kadar bunalttı?

18. Yerinden sıçramak veya kolayca irkilmek sizi ne kadar bunalttı?

19. Dikkati toplamada güçlükler sizi ne kadar bunalttı?

20. Uykuya dalma veya uykuyu devam ettirme güçlükleri sizi ne kadar bunalttı?

For peer review only

For peer review only - http://bmjopen.bmj.com/site/about/guidelines.xhtml

WHO-5

Aşağıdaki beş tanımlamadan her biri için, son iki hafta süresince kendinizi nasıl hissettiğinize en yakın olan yanıtı veriniz. Son iki hafta boyunca

	Her zaman 5	Çoğu zaman 4	Zamanın yarısından çoğunda 3	Zamanın yarısından azında 2	Bazen 1	Hiçbir zaman 0
1. Kendimi neşeli ve keyifli hissettim	○	○	○	○	○	○
2. Kendimi sakin ve rahatlamış hissettim.	○	○	○	○	○	○
3. Kendimi aktif ve dinç hissettim	○	○	○	○	○	○
4. Sabahları kendimi taze ve dinlenmiş hissederek uyandım	○	○	○	○	○	○
5. Günlük yaşantım beni ilgilendiren şeylerle dolu	○	○	○	○	○	○

For peer review only

For peer review only - http://bmjopen.bmj.com/site/about/guidelines.xhtml

PTGI

COVID-19 sonucunda yaşamınızda ve düşüncelerinizde meydana gelen değişimleri lütfen aşağıda verilen puanlama ölçütlerine göre 0 ve 5 arasında değerlendiriniz.

	Hiç 0	Çok az 1	Az derecede 2	Orta derecede 3	Oldukça fazla 4	Aşırı derecede 5
1. Yaşamda önem verdiğim şeylerin öncelik sırası değişti.	○	○	○	○	○	○
2. Kendi hayatıma verdiğim değerde büyük bir artış oldu.	○	○	○	○	○	○
3. Yeni ilgi alanları keşfettim.	○	○	○	○	○	○
4. Kendime güven hissinde artış oldu.	○	○	○	○	○	○
5. Manevi konuları daha iyi anlamaya başladım.	○	○	○	○	○	○
6. Başım sıkıştığında insanlara güvenebileceğimi daha iyi anladım.	○	○	○	○	○	○
7. Yaşamım için yeni bir yön belirledim.	○	○	○	○	○	○
8. Kendimi diğer insanlarla çok daha yakın hissetmeye başladım.	○	○	○	○	○	○
9. Duygularımı ifade etmeye daha çok istekliyim.	○	○	○	○	○	○
10. Zorlukları göğüsleyebileceğimi daha iyi anladım.	○	○	○	○	○	○
11. Yaşamımda daha iyi şeyler yapabiliyorum.	○	○	○	○	○	○
12. Her şeyi olduğu gibi, daha çok kabullenebiliyorum.	○	○	○	○	○	○
13. Her günümü daha iyi değerlendirebiliyorum.	○	○	○	○	○	○

For peer review only

For peer review only - http://bmjopen.bmj.com/site/about/guidelines.xhtml

14. Daha önce var olmayan yeni olanaklara kavuştum.	○	○	○	○	○	○
15. Diğer insanlara karşı daha şefkatliyim.	○	○	○	○	○	○
16. İlişkilerime daha çok emek sarf etmeye başladım.	○	○	○	○	○	○
17. Değişmesi gereken şeyleri değiştirebilmek için daha çok çaba harcıyorum.	○	○	○	○	○	○
18. Daha güçlü bir inanca sahibim.	○	○	○	○	○	○
19. Düşündüğümden çok daha güçlü olduğumu keşfettim.	○	○	○	○	○	○
20. İnsanların ne kadar mükemmel olabildiklerine dair çok şey öğrendim.	○	○	○	○	○	○
21. Başkalarına ihtiyaç duyuyor olmayı daha çok kabullendim.	○	○	○	○	○	○

For peer review only

For peer review only - http://bmjopen.bmj.com/site/about/guidelines.xhtml

International Study – Pakistan (Urdu)

Sociodemographic Information

1. جنس

- ◯ مرد
- ◯ عورت
- ◯ دیگر
- ◯ جواب نہیں دینا چاہتے

2. عمر (سالوں میں)

____

3. ازدواجی حیثیت

- ◯ غیر شادی شدہ
- ◯ غیر ازدوجی تعلق
- ◯ شادی شدہ
- ◯ علیحدگی
- ◯ طلاق یافتہ
- ◯ بیوہ یا رنڈوا

4. آپ کا دین کیا ہے؟

- ◯ کوئی دین نہیں
- ◯ اسلام
- ◯ عیسائیت
- ◯ ہندومت
- ◯ بدھ مت

For peer review only - http://bmjopen.bmj.com/site/about/guidelines.xhtml

○ دیگر (براہ مہربانی وضاحت کریں) ___________________________________

5. آپ کا نسلی پس منظر کیا ہے؟ (آپ ایک سے زیادہ درج کرسکتے ہیں)

____

6. آپ روزمرہ کی زندگی میں کون سی زبانیں روانی سے بولتے ہیں؟

____

درج ذیل دو سوالات آپ کی انگریزی زبان کی اہلیت کے بارے میں ہیں۔
6a. میں انگریزی اچھی طرح سمجھ سکتا/سکتی ہوں؟*

○ بالکل بھی نہیں1

○ تھوڑی سی2

○ درمیانی حد تک3

○ زیادہ4

○ بہت زیادہ5

6b. میں انگریزی اچھی طرح بول سکتا/سکتی ہوں؟

○ بالکل بھی نہیں1

○ تھوڑی سی 2

○ درمیانی حد تک3

○ زیادہ4

○ بہت زیادہ5

7. آپ کی تعلیم کی اعلی ترین سطح کیا ہے؟

○ کوئی باضابطہ تعلیم نہیں

○ ہائیر سیکنڈری سکول (جیسے ڈپلومہ یا A-level)

○ سرٹیفکیٹ یا ڈپلومہ 3 - سال سے کم کورس

For peer review only - http://bmjopen.bmj.com/site/about/guidelines.xhtml

○ بیچلر ڈگری (3-4 سالہ کورس)

○ پوسٹ گریجویٹ ڈگری (پوسٹ گریجویٹ ڈپلومہ، ماسٹر، ڈاکٹریٹ)

8. موجودہ اسٹڈی یا کام کی حیثیت (براہ مہربانی جو بھی لاگو ہوں ان پر نشان لگائیں)

☐ فل ٹائم طالب علم

☐ پارٹ ٹائم طالب علم

☐ بے روزگار/کام کی تلاش

☐ فل ٹائم ملازمت

☐ پارٹ ٹائم ملازمت

☐ فل ٹائم ذاتی کام

☐ پارٹ ٹائم ذاتی کام

☐ دفتری پیڈ پیرینٹل چھٹی (paid parental leave)

☐ ہاؤس وائف یا سٹیے-ایٹ-ہوم والد (stay-at-home dad) بلا معاوضہ

☐ ریٹائرڈ

☐ دیگر (براہ مہربانی وضاحت کریں) ___________________________________

9. پیشہ

____

10. ماہانہ گھریلو آمدنی (مقامی کرنسی میں)

____

10a. اوسط آمدن کے مقابلے میں، آپ اپنے گھر کی آمدنی کی درجہ بندی کیسے کریں گے؟

○ اوسط سے کم

○ اوسط

○ اوسط سے زیادہ

11. کیا آپ کوویڈ 19-کی وجہ سے لاک ڈاؤن میں ہیں؟

○ ہاں

○ نہیں

For peer review only - http://bmjopen.bmj.com/site/about/guidelines.xhtml

.12 کیا آپ کو کوویڈ 19-کی ویکسین دی گئی ہے؟

- ہاں
- نہیں

.12a اگر نہیں، تو کیا آپ مستقبل میں کوویڈ 19-کی ویکسین لینا چاہیں گیں؟

- ہاں
- نہیں

.13 ہم سمجھتے ہیں کہ کچھ لوگوں کو ماضی میں تکلیف دہ واقعات کا سامنا کرنا پڑا ہوا ہو گا جس کی وجہ سے نئی پریشانیوں سے نمٹنے میں آسانی یا دقت پیدا ہو سکتی ہے۔

کوویڈ-19کی وبا سے پہلے، کیا آپ نے کبھی مندرجہ ذیل میں سے کسی واقعہ کا مشاہدہ یا تجربہ کیا ہے: (براہ مہربانی جو بھی لاگو ہوں ان پر نشان لگائیں)

- ☐ قدرتی آفات (جیسے سیلاب، زلزلہ، وغیرہ)
- ☐ جنگ یا فوجی تنازعات
- ☐ 16 سال کی عمر سے پہلے، بچپن کی پریشانیاں (جیسے نظر انداز ہونا (neglect)، غنڈہ گردی کا شکار (bullying) جسمانی یا جنسی تشدد)
- ☐ 16 سال کی عمر کے بعد جسمانی یا جنسی تشدد
- ☐ سنگین حادثہ / ایکسیڈنٹ
- ☐ دیگر – وضاحت کریں (وضاحت کرنا لازمی نہیں)____
- ☐ مندرجہ بالا میں سے کوئی نہیں

For peer review only

Covid Psychosocial Impacts Scale

ذاتی اثرات

مندرجہ ذیل بیانات کوویڈ-19 کے آپ پر ہونے والے اثرات کے بارے میں ہیں، براہ مہربانی درج ذیل بیانات کے لئے" ہاں / نہیں "میں سے کسی ایک کا انتخاب کریں۔ اگر آپ کا جواب ہاں میں ہے تو، براہ مہربانی نیچے دیے گیے اسکیل پر اس کی وجہ سے ہونے والے دباؤ (stress) کی نشاندہی کریں۔

1. کیا آپ کو کوئی ایسی بیماری لاحق ہے جو آپ کو کوویڈ-19 کی زِد میں لا سکتی ہے؟

- نہیں 0
- **ہاں** کوئی دباؤ نہیں 1
- **ہاں** تھوڑا سا دباؤ 2
- **ہاں** درمیانی حد تک دباؤ 3
- **ہاں** زیادہ دباؤ 4
- **ہاں** بہت زیادہ دباؤ 5

2. کیا آپ کو کسی شخص سے کوویڈ-19 کی زِد میں آنے کا خطرہ لاحق ہوا ہے؟

- نہیں 0
- **ہاں** کوئی دباؤ نہیں 1
- **ہاں** تھوڑا سا دباؤ 2
- **ہاں** درمیانی حد تک دباؤ 3
- **ہاں** زیادہ دباؤ 4
- **ہاں** بہت زیادہ دباؤ 5

3. کیا آپ کو لگتا ہے کہ آپ کو کوویڈ-19 ہوا تھا؟

- نہیں 0
- **ہاں** کوئی دباؤ نہیں 1

For peer review only - http://bmjopen.bmj.com/site/about/guidelines.xhtml

- ہاں تھوڑا سا دباؤ 2
- ہاں درمیانی حد تک دباؤ 3
- ہاں زیادہ دباؤ 4
- ہاں بہت زیادہ دباؤ 5

3a. کیا آپ کا کوویڈ-19 کا ٹیسٹ مثبت آیا تھا؟

- نہیں 0
- ہاں کوئی دباؤ نہیں 1
- ہاں تھوڑا سا دباؤ 2
- ہاں درمیانی حد تک دباؤ 3
- ہاں زیادہ دباؤ 4
- ہاں بہت زیادہ دباؤ 5

3b. کیا آپ کو آج کل کوویڈ-19 کی علامات ہیں؟

- نہیں 0
- ہاں کوئی دباؤ نہیں 1
- ہاں تھوڑا سا دباؤ 2
- ہاں درمیانی حد تک دباؤ 3
- ہاں زیادہ دباؤ 4
- ہاں بہت زیادہ دباؤ 5

For peer review only

For peer review only - http://bmjopen.bmj.com/site/about/guidelines.xhtml

خاندان والوں پر اثرات

مندرجہ ذیل بیانات کوویڈ-19 کے آپ کے قریبی خاندان والوں پر ہونے والے اثرات کے بارے میں ہیں، براہ مہربانی درج ذیل بیانات کے لئے "ہاں / نہیں "میں سے کسی ایک کا انتخاب کریں۔ اگر آپ کا جواب ہاں میں ہے تو، براہ مہربانی نیچے دیے گیے اسکیل پر اس کی وجہ سے ہونے والے دباؤ (stress) کی نشاندہی کریں۔

1. کیا آپ کے خاندان کے کسی قریبی فرد کو کوئی ایسی بیماری ہے جو انہیں کوویڈ-19 کی زِد میں لا سکتی ہے؟

- نہیں 0
- **ہاں** کوئی دباؤ نہیں 1
- **ہاں** تھوڑا سا دباؤ 2
- **ہاں** درمیانی حد تک دباؤ 3
- **ہاں** زیادہ دباؤ 4
- **ہاں** بہت زیادہ دباؤ 5

2. کیا آپ کو خدشہ ہے کہ آپ کے خاندان کے کسی قریبی فرد کو کسی شخص سے کوویڈ-19کی زِد میں آنے کا خطرہ لاحق ہوا ہے؟

- نہیں 0
- **ہاں** کوئی دباؤ نہیں 1
- **ہاں** تھوڑا سا دباؤ 2
- **ہاں** درمیانی حد تک دباؤ 3
- **ہاں** زیادہ دباؤ 4
- **ہاں** بہت زیادہ دباؤ 5

3. کیا آپ کو لگتا ہے کہ خاندان کے کسی قریبی فرد کو کوویڈ-19 ہوا تھا؟

- نہیں 0
- **ہاں** کوئی دباؤ نہیں 1
- **ہاں** تھوڑا سا دباؤ 2

For peer review only - http://bmjopen.bmj.com/site/about/guidelines.xhtml

- **ہاں** درمیانی حد تک دباؤ 3
- **ہاں** زیادہ دباؤ 4
- **ہاں** بہت زیادہ دباؤ 5

3a. کیا خاندان کے کسی قریبی فرد کا کوویڈ-19 کا ٹیسٹ مثبت آیا تھا؟

- **نہیں** 0
- **ہاں** کوئی دباؤ نہیں 1
- **ہاں** تھوڑا سا دباؤ 2
- **ہاں** درمیانی حد تک دباؤ 3
- **ہاں** زیادہ دباؤ 4
- **ہاں** بہت زیادہ دباؤ 5

3b. کیا خاندان کے کسی قریبی فرد کو آج کل کوویڈ-19 کی علامات ہیں؟

- **نہیں** 0
- **ہاں** کوئی دباؤ نہیں 1
- **ہاں** تھوڑا سا دباؤ 2
- **ہاں** درمیانی حد تک دباؤ 3
- **ہاں** زیادہ دباؤ 4
- **ہاں** بہت زیادہ دباؤ 5

3c. کیا خاندان کے کسی قریبی فرد کا کوویڈ-19 سے انتقال ہوا ہے؟

- **نہیں** 0
- **ہاں** کوئی دباؤ نہیں 1

For peer review only - http://bmjopen.bmj.com/site/about/guidelines.xhtml
For peer review only

ہاں تھوڑا سا دباؤ 2

ہاں درمیانی حد تک دباؤ 3

ہاں زیادہ دباؤ 4

ہاں بہت زیادہ دباؤ 5

4. کیا آپ کو فکر لاحق ہے کہ بیرون ملک مقیم خاندان کے کسی قریبی فرد کو کوویڈ-19 کی زِد میں آنے کا امکان ہے؟

نہیں 0

ہاں کوئی دباؤ نہیں 1

ہاں تھوڑا سا دباؤ 2

ہاں درمیانی حد تک دباؤ 3

ہاں زیادہ دباؤ 4

ہاں بہت زیادہ دباؤ 5

5. کیا بیرون ملک مقیم خاندان کے کسی قریبی فرد کو آپ کوویڈ-19 کی وجہ سے ملنے نہیں جا سکتے یا وہ آپ کوویڈ-19 کی وجہ سے ملنے نہیں آ سکتے ہیں؟

نہیں 0

ہاں کوئی دباؤ نہیں 1

ہاں تھوڑا سا دباؤ 2

ہاں درمیانی حد تک دباؤ 3

ہاں زیادہ دباؤ 4

ہاں بہت زیادہ دباؤ 5

کام

مندرجہ ذیل بیانات کوویڈ-19 کے آپ اور آپ کے گھرانے پر ہونے والے اثرات کے بارے میں ہیں، براہ مہربانی درج ذیل بیانات کے لئے " ہاں / نہیں / قابل اطلاق نہیں "میں سے کسی ایک کا انتخاب کریں۔ اگر آپ کا جواب ہاں میں ہے تو، براہ مہربانی نیچے دیے گیے اسکیل پر اس کی وجہ سے ہونے والے دباؤ (stress) کی نشاندہی کریں۔

For peer review only - http://bmjopen.bmj.com/site/about/guidelines.xhtml

1. کیا آپ یا آپ کے گھر کا کوئی فرد ایک ضروری ورکر کے طور پر شمار کیا گیا ہے (مثلاً ہیلتھ کیئر، قانون نافذ کرنے والے ادارے، ایمرجنسی سروسز، اسینشل/ضروری سامان/خدمات فراہم کرنے والے اداروں میں)؟

- ○ نہیں 0
- ○ ہاں کوئی دباؤ نہیں 1
- ○ ہاں تھوڑا سا دباؤ 2
- ○ ہاں درمیانی حد تک دباؤ 3
- ○ ہاں زیادہ دباؤ 4
- ○ ہاں بہت زیادہ دباؤ 5

2. کیا آپ کے یا آپ کے گھرانے کے کسی فرد کے کام کرنے کے اوقات میں ایک بڑی تبدیلی رونما ہوئی ہے؟

- ○ نہیں 0
- ○ ہاں کوئی دباؤ نہیں 1
- ○ ہاں تھوڑا سا دباؤ 2
- ○ ہاں درمیانی حد تک دباؤ 3
- ○ ہاں زیادہ دباؤ 4
- ○ ہاں بہت زیادہ دباؤ 5

3. کیا آپ کو یا آپ کے گھرانے کے کسی فرد کو گھر سے کام کرنا پڑرہا ہے؟

- ○ نہیں 0
- ○ ہاں کوئی دباؤ نہیں 1
- ○ ہاں تھوڑا سا دباؤ 2
- ○ ہاں درمیانی حد تک دباؤ 3
- ○ ہاں زیادہ دباؤ 4

For peer review only

For peer review only - http://bmjopen.bmj.com/site/about/guidelines.xhtml

○ ہاں بہت زیادہ دباؤ 5

4. کیا آپ کے یا آپ کے گھرانے کے کسی فرد کے کام (job-related work) کی نوعیت بدَل گئی ہے؟

○ نہیں 0

○ ہاں کوئی دباؤ نہیں 1

○ ہاں تھوڑا سا دباؤ 2

○ ہاں درمیانی حد تک دباؤ 3

○ ہاں زیادہ 4

○ ہاں بہت زیادہ دباؤ 5

5. کیا آپ کی یا آپ کے گھرانے کے کسی فرد کی ملازمت چھوٹ گئی ہے؟

○ قابل اطلاق نہیں

○ نہیں 0

○ ہاں کوئی دباؤ نہیں 1

○ ہاں تھوڑا سا دباؤ 2

○ ہاں درمیانی حد تک دباؤ 3

○ ہاں زیادہ دباؤ 4

○ ہاں بہت زیادہ دباؤ 5

6. کیا ایسا کاروبار جو آپ کی یا آپ کے گھرانے کی ملکیت ہے، یا جس میں آپ یا آپ کے گھر والے کام کرتے ہیں، بقا کے خطرے میں ہے؟

○ قابل اطلاق نہیں

○ نہیں 0

○ ہاں کوئی دباؤ نہیں 1

For peer review only - http://bmjopen.bmj.com/site/about/guidelines.xhtml

ہاں تھوڑا سا دباؤ 2

ہاں درمیانی حد تک دباؤ 3

ہاں زیادہ دباؤ 4

ہاں بہت زیادہ دباؤ 5

آمدن

مندرجہ ذیل بیانات کوویڈ-19 کے آپ کے گھرانے پر ہونے والے اثرات کے بارے میں ہیں، براہ مہربانی درج ذیل بیانات کے لئے" ہاں / نہیں " میں سے کسی ایک کا انتخاب کریں۔ اگر آپ کا جواب ہاں میں ہے تو، براہ مہربانی نیچے دیے گیے اسکیل پر اس کی وجہ سے ہونے والے دباؤ (stress) کی نشاندہی کریں۔

1. کیا آپ کے گھرانے کے مالی حالات میں واضح کمی واقع ہوئی ہے؟

نہیں 0

ہاں کوئی دباؤ نہیں 1

ہاں تھوڑا سا دباؤ 2

ہاں درمیانی حد تک دباؤ 3

ہاں زیادہ دباؤ 4

ہاں بہت زیادہ دباؤ 5

2. کیا آپ کے گھرانے کی بلوں کی ادائیگی یا کھانے پینے کے اخراجات کرنے کی صلاحیت میں واضح کمی واقع ہوئی ہے؟

نہیں 0

ہاں کوئی دباؤ نہیں 1

ہاں تھوڑا سا دباؤ 2

ہاں درمیانی حد تک دباؤ 3

ہاں زیادہ دباؤ 4

ہاں بہت زیادہ دباؤ 5

For peer review only - http://bmjopen.bmj.com/site/about/guidelines.xhtml
For peer review only

تعلقات

مندرجہ ذیل بیانات کوویڈ-19 کے آپ پر ہونے والے اثرات کے بارے میں ہیں، براہ مہربانی درج ذیل بیانات کے لئے" ہاں / نہیں "میں سے کسی ایک کا انتخاب کریں۔ اگر آپ کا جواب ہاں میں ہے تو، براہ مہربانی نیچے دیے گیے اسکیل پر اس کی وجہ سے ہونے والے دباؤ (stress) کی نشاندہی کریں۔

1. کیا کوویڈ-19 کے نتیجے میں، فیملی کی مشکلات اور باہمی ناچاقی میں اضافہ ہوا ہے؟

- ○ نہیں 0
- ○ **ہاں** کوئی دباؤ نہیں 1
- ○ **ہاں** تھوڑا سا دباؤ 2
- ○ **ہاں** درمیانی حد تک دباؤ 3
- ○ **ہاں** زیادہ دباؤ 4
- ○ **ہاں** بہت زیادہ دباؤ 5

2. کیا کوویڈ-19 کے نتیجے میں، دوستوں کے ساتھ آپ کے رابطے (روبرو یا آن لائن) میں کمی واقع ہوئی ہے؟

- ○ نہیں 0
- ○ **ہاں** کوئی دباؤ نہیں 1
- ○ **ہاں** تھوڑا سا دباؤ 2
- ○ **ہاں** درمیانی حد تک دباؤ 3
- ○ **ہاں** زیادہ دباؤ 4
- ○ **ہاں** بہت زیادہ دباؤ 5

روزمرہ کے معمولات اور طرزعمل میں تبدیلیاں

مندرجہ ذیل بیانات کوویڈ-19 کے آپ پر ہونے والے اثرات کے بارے میں ہیں، براہ مہربانی درج ذیل بیانات کے لئے" ہاں / نہیں" میں سے کسی ایک کا انتخاب کریں۔ اگر آپ کا جواب ہاں میں ہے تو، براہ مہربانی نیچے دیے گیے اسکیل پر اس کی وجہ سے ہونے والے دباؤ (stress) کی نشاندہی کریں۔

1. کیا کوویڈ-19 کی وجہ سے آپ کے روزمرہ معمولات (مثال کے طور پر، نیند، ورزش، فارغ وقت، خوشگوار سرگرمیوں، یا مشاغل) میں تبدیلی واقع ہوئی ہے؟

For peer review only - http://bmjopen.bmj.com/site/about/guidelines.xhtml
For peer review only

- نہیں 0
- **ہاں** کوئی دباؤ نہیں 1
- **ہاں** تھوڑا سا دباؤ 2
- **ہاں** درمیانی حد تک دباؤ 3
- **ہاں** زیادہ دباؤ 4
- **ہاں** بہت زیادہ دباؤ 5

2. کیا کوویڈ-19 کی وجہ سے آپ نے اپنے یا اپنے گھر والوں کی ہیلتھ کیئر (healthcare) کے حصول میں تاخیر کی ہے؟

- نہیں 0
- **ہاں** کوئی دباؤ نہیں 1
- **ہاں** تھوڑا سا دباؤ 2
- **ہاں** درمیانی حد تک دباؤ 3
- **ہاں** زیادہ دباؤ 4
- **ہاں** بہت زیادہ دباؤ 5

3. کیا کوویڈ-19 کی وجہ سے آپ کی دینی سرگرمیوں(روبرو یا آن لائن) میں شمولیت میں کمی واقع ہوئی ہے؟

- **قابل اطلاق نہیں**
- نہیں 0
- **ہاں** کوئی دباؤ نہیں 1
- **ہاں** تھوڑا سا دباؤ 2
- **ہاں** درمیانی حد تک دباؤ 3
- **ہاں** زیادہ دباؤ 4

For peer review only - http://bmjopen.bmj.com/site/about/guidelines.xhtml

◯ ہاں بہت زیادہ دباؤ 5

4. کیا کوویڈ-19 کی وجہ سے معمول میں کوئی اور بڑی تبدیلی واقع ہوئی ہے؟

◯ نہیں 0

◯ ہاں (براہ مہربانی وضاحت کریں۔) 1 ________________________________

اگر آپ کا جواب ہاں میں ہے تو، براہ مہربانی نیچے دیے گیے اسکیل پر اس کی وجہ سے ہونے والے دباؤ (stress) کی نشاندہی کریں۔

◯ کوئی دباؤ نہیں 1

◯ تھوڑا سا دباؤ 2

◯ درمیانی حد تک دباؤ 3

◯ زیادہ دباؤ 4

◯ بہت زیادہ دباؤ 5

کوویڈ-19 کے بارے میں معلومات

1. کوویڈ۔19 کے حوالے سے، آپ کے لئے معلومات کا ذریعہ کیا ہے؟ (براہ مہربانی جو بھی آپ پر لاگو ہوں انہیں منتخب کریں۔)

☐ خبریں

☐ فیس بک / ٹویٹر

☐ ہیلتھ ویب سائٹس

☐ لوگوں کی باتیں

☐ دیگر(براہ مہربانی وضاحت کریں) ________________________________

2. کوویڈ-19 سے متعلق خبریں آپ کے لیے کس قدر دباؤ (stress) کا سبب ہیں؟

◯ کوئی دباؤ نہیں 1

◯ تھوڑا سا دباؤ 2

◯ درمیانی حد تک دباؤ 3

◯ زیادہ دباؤ 4

◯ بہت زیادہ دباؤ 5

For peer review only - http://bmjopen.bmj.com/site/about/guidelines.xhtml

کوویڈ- 19کے بعد ہونے والی تبدیلیاں

براہ کرم کوویڈ-19 کی عالمی وبا کا پہلے سے موازنہ کرتے ہوئے، درج ذیل بیانات پر دیئے گئے پیمانے کی مدد سے نشاندہی کریں۔

1. آپ اپنے ذاتی ذہنی دباؤ (stress) کی درجہ بندی کس طرح کریں گیں؟

- پہلے سے بہت کم دباؤ 1
- پہلے سے کم دباؤ 2
- کوئی تبدیلی نہیں 3
- پہلے سے زیادہ دباؤ 4
- پہلے سے بہت زیادہ دباؤ 5

2. آپ اپنے گھرانے کے ذہنی دباؤ (stress) کی درجہ بندی کس طرح کریں گیں؟

- پہلے سے بہت کم دباؤ 1
- پہلے سے کم دباؤ 2
- کوئی تبدیلی نہیں 3
- پہلے سے زیادہ دباؤ 4
- پہلے سے بہت زیادہ دباؤ 5

3. آپ اپنی نفسیاتی صحت کی درجہ بندی کس طرح کریں گیں؟

- پہلے سے بہت بہتر 1
- پہلے سے بہتر 2
- کوئی تبدیلی نہیں 3
- پہلے سے بدتر 4
- پہلے سے بہت بدتر 5

For peer review only - http://bmjopen.bmj.com/site/about/guidelines.xhtml

4. آپ اپنے گھرانے کی نفسیاتی صحت کی درجہ بندی کس طرح کریں گیں؟

- پہلے سے بہت بہتر 1
- پہلے سے بہتر 2
- کوئی تبدیلی نہیں 3
- پہلے سے بدتر 4
- پہلے سے بہت بدتر 5

For peer review only

For peer review only - http://bmjopen.bmj.com/site/about/guidelines.xhtml

K-10

نیچے دئیے گئے دس سوالات میں آپ سے پوچھا گیا ہے کہ آپ پچھلے **4** ہفتوں سے کیسا محسوس کر رہے ہیں۔ برائے مہربانی ہر سوال کے لئے نشاندہی کریں جو اس بات کی بہترین وضاحت کرتا ہو کہ آپ نے اس کیفیت کو کتنی مرتبہ محسوس کیا ہے۔

	**کبھی نہیں 1**	**بہت کم وقت 2**	**کبھی کبھار 3**	**زیادہ وقت 4**	**ہر وقت 5**
1. پچھلے 4 ہفتوں میں، آپ نے کتنی مرتبہ بغیر کسی معقول وجہ کے تھکاوٹ محسوس کی؟	○	○	○	○	○
2. پچھلے 4 ہفتوں میں، آپ نے کتنی مرتبہ گھبراہٹ محسوس کی؟	○	○	○	○	○
3. پچھلے 4 ہفتوں میں، آپ نے کتنی مرتبہ اتنی گھبراہٹ محسوس کی کہ کوئی بھی چیز آپ کو سکون نہ دے سکی؟	○	○	○	○	○
4. پچھلے 4 ہفتوں میں، آپ نے کتنی مرتبہ نا اُمیدی محسوس کی؟	○	○	○	○	○
5. پچھلے 4 ہفتوں میں، آپ نے کتنی مرتبہ بے سکونی یا بے چینی محسوس کی؟	○	○	○	○	○
6. پچھلے 4 ہفتوں میں، آپ نے کتنی مرتبہ اتنی بے چینی محسوس کی کہ آپ ٹک کے نہ بیٹھ سکتے تھے؟	○	○	○	○	○
7. پچھلے 4 ہفتوں میں، آپ نے کتنی مرتبہ افسردگی (ڈپریشن) محسوس کی*	○	○	○	○	○
8. پچھلے 4 ہفتوں میں، آپ نے کتنی مرتبہ محسوس کیا کہ ہر کام کرنے کے لیے کوشش کرنی پڑ رہی ہے؟	○	○	○	○	○
9. پچھلے 4 ہفتوں میں، آپ کتنی مرتبہ اتنے اُداس تھے کہ کوئی بھی چیز آپ کو خوش نہ کر سکی؟	○	○	○	○	○
10. پچھلے 4 ہفتوں میں، آپ نے کتنی مرتبہ خود کو بے وقعت محسوس کیا؟	○	○	○	○	○

For peer review only

For peer review only - http://bmjopen.bmj.com/site/about/guidelines.xhtml

PCL-5

نیچے ان مسائل کی فہرست دی گئی ہے جو لوگوں کو کوویڈ-19 کے نتیجے میں پیش آ سکتے ہیں۔ براہ مہربانی ہر مسئلے کو غور سے پڑھیں اور پھر دیے گئے نمبروں میں سے کسی ایک کو چن کر نشاندہی کریں کہ پچھلے مہینے میں آپ کو اس مسئلے نے کتنا پریشان کیا ہے۔

	بالکل بھی نہیں 0	تھوڑا سا 1	درمیانی حد تک 2	زیادہ 3	بہت زیادہ 4
1. پریشان کن تجربے کی بار بار آنے والی، تکلیف دہ، اور ناپسندیدہ یادیں؟	○	○	○	○	○
2. پریشان کن تجربے کے بار بار آنے والے، تکلیف دہ خواب؟	○	○	○	○	○
3. اچانک محسوس ہونا یا ایسا لگنا کہ پریشان کن تجربہ واقعی دوبارہ ہو رہا ہے (جیسے آپ ماضی میں چلے گئے ہوں اور اس سے دوبارہ گزر رہے ہوں)۔	○	○	○	○	○
4. بہت گھبراہٹ محسوس کرنا جب کوئی چیز آپ کو پریشان کن تجربے کی یاد دلائے۔	○	○	○	○	○
5. سخت جسمانی رد عمل محسوس کرنا جب کوئی چیز آپ کو پریشان کن تجربے کی یاد دلائے (مثلاً، دل کا زور زور سے دھڑکنا، سانس لینے میں دشواری، پسینہ چھوٹنا)؟	○	○	○	○	○
6. پریشان کن تجربے سے متعلق یادوں، خیالات، یا احساسات سے گریز کرنا؟	○	○	○	○	○
7. پریشان کن تجربے کی بیرونی یاد دہانیوں سے گریزکرنا (مثلاً لوگ، مقامات، گفتگو، سرگرمیاں، اشیاء، یا حالات)؟	○	○	○	○	○
8. پریشان کن تجربے کے اہم حصوں کو یاد کرنے میں دقت محسوس کرنا؟	○	○	○	○	○
9. اپنے، دوسرے لوگوں، یا دنیا کے بارے میں سخت منفی خیالات آنا (مثلاً، اس طرح کے خیالات جیسے: میں برا/بری ہوں، میرے ساتھ واقعی میں کچھ مسئلہ ہے، کسی پر بھی اعتبار نہیں کیا جاسکتا، پوری دنیا خطرناک ہے)؟	○	○	○	○	○
10. پریشان کن تجربے کے لئے یا اس کے بعد جو کچھ ہوا اس کے لئے اپنے آپ کو یا کسی اور کو مورد الزام ٹھہرانا؟	○	○	○	○	○
11. سخت منفی جذبات کا ہونا جیسے خوف، وحشت، غصہ، احساس جرم، یا شرمندگی ؟	○	○	○	○	○
12. ان سرگرمیوں میں عدم دلچسپی جن سے آپ کبھی لطف اندوز ہوتے تھے؟	○	○	○	○	○
13. دوسرے لوگوں سے دوری محسوس کرنا یا تعلقات منقطع کرنا؟	○	○	○	○	○

For peer review only

For peer review only - http://bmjopen.bmj.com/site/about/guidelines.xhtml

14. مثبت احساسات محسوس کرنے میں دشواری (مثلاً، آپ کا اپنے قریبی لوگوں کے لئے خوشی یا محبت کے جذبات محسوس کرنے سے قاصر ہونا)؟	○	○	○	○	○
15. چڑچڑاپن، برہمی، یا جارحانہ سلوک؟	○	○	○	○	○
16. بہت زیادہ خطرات مول لینا یا ایسی چیزیں کرنا جو آپ کو نقصان پہنچا سکتی ہیں؟	○	○	○	○	○
17. انتہائی الرٹ/چوکنا یا محتاط رہنا؟	○	○	○	○	○
18. بہت تیزی یا آسانی سے چونک جانا؟	○	○	○	○	○
19. توجہ دینے میں دقت پیش آنا؟	○	○	○	○	○
20. سونے میں دشواری یا نیند کے مسائل؟	○	○	○	○	○

For peer review only

For peer review only - http://bmjopen.bmj.com/site/about/guidelines.xhtml

WHO-5

براہِ مہربانی ان پانچ بیانات میں سے ہر ایک کے لئے نشاندہی کریں کہ آپ پچھلے دو ہفتوں سے کیسا محسوس کر رہے ہیں۔

	ہر وقت 5	زیادہ تر وقت 4	آدھے سے زیادہ وقت میں 3	آدھے سے کم وقت میں 2	کچھ وقت 1	کسی وقت بھی نہیں 0
.1 میں نے خوشی اور زندہ دلی محسوس کی	○	○	○	○	○	○
.2 میں نے سکون اور راحت محسوس کیا	○	○	○	○	○	○
.3 میں نے فعال اور چست محسوس کیا	○	○	○	○	○	○
.4 میں نے سو کر اٹھتے ہوئے تازہ دم اور آرام دہ محسوس کیا	○	○	○	○	○	○
.5 میری روزمرہ زندگی ایسی چیزوں سے بھری پڑی ہے جن میں مجھے دلچسپی ہے	○	○	○	○	○	○

For peer review only

PGTI

درج ذیل بیانات میں سے ہر ایک کی دیے گئے اسکیل کی مدد سے نشاندہی کریں کہ کوویڈ 19-کے نتیجے میں آپ کی زندگی میں کس درجہ کی تبدیلی واقع ہوئی ہے۔

	بالکل نہیں 0	بہت تھوڑا 1	تھوڑا 2	درمیانی حد تک 3	زیادہ 4	بہت زیادہ 5
1. زندگی میں کیا اہم ہے اس کے بارے میں میں نے اپنی ترجیحات کو تبدیل کر دیا ہے	○	○	○	○	○	○
2. مجھے اپنی زندگی کی قدروقیمت پہلے سے زیادہ ہے	○	○	○	○	○	○
3. میں نے نئی دلچسپیاں بنا لی ہیں	○	○	○	○	○	○
4. مجھے احساس خود انحصاری پہلے سے زیادہ ہے	○	○	○	○	○	○
5. مجھے روحانی امور کی سمجھ بوجھ پہلے سے بہتر ہے	○	○	○	○	○	○
6. میں پہلے سے بہتر طور پر دیکھ سکتا /سکتی ہوں کہ میں مصیبت کے وقت لوگوں پر بھروسہ کر سکتا / سکتی ہوں	○	○	○	○	○	○
7. میں نے اپنی زندگی کی ایک نئی راہ متعین کر لی ہے	○	○	○	○	○	○
8. مجھے دوسروں کے ساتھ قربت کا احساس پہلے سے زیادہ ہے	○	○	○	○	○	○
9. میں اپنے جذبات کا اظہار کرنے کے لیے پہلے سے زیادہ آمادہ ہوں	○	○	○	○	○	○
10. میں پہلے سے بہتر جانتا / جانتی ہوں کہ میں مشکلات کا مقابلہ کر سکتا / سکتی ہوں	○	○	○	○	○	○
11. میں اپنی زندگی میں پہلے سے بہتر کام سَر انجام دے رہا / رہی ہوں	○	○	○	○	○	○
12. میری چیزوں کا انجام قبول کرنے کی صلاحیت پہلے سے بہتر ہے	○	○	○	○	○	○
13. میں ہر دن کی قدر پہلے سے زیادہ کرتا / کرتی ہوں	○	○	○	○	○	○

For peer review only

For peer review only - http://bmjopen.bmj.com/site/about/guidelines.xhtml

14. نئے مواقع میسر ہیں جو بصورت دیگر میسر نہ ہوتے	○	○	○	○	○	○
15. مجھ میں دوسروں کے لئے شفقت پہلے سے زیادہ ہے	○	○	○	○	○	○
16. میں اپنے تعلقات نبھانے میں پہلے سے زیادہ محنت کرتا / کرتی ہوں	○	○	○	○	○	○
17. میں ان چیزوں کو تبدیل کرنے کی پہلے سے زیادہ کوشش کرتا / کرتی ہوں جن کو تبدیل کرنے کی ضرورت ہے	○	○	○	○	○	○
18. میرا ایمان پہلے سے زیادہ مضبوط ہے	○	○	○	○	○	○
19. میں نے جان لیا ہے کہ میں اپنی سوچ سے ذیادہ مضبوط ہوں	○	○	○	○	○	○
20. میں نے پہلے سے بھی ذیادہ سیکھا ہے کہ لوگ کتنے اچھے ہیں	○	○	○	○	○	○
21. میں دوسروں سے ملنے والی مدد کو قبول کرنے کے لیے پہلے سے زیادہ آمادہ ہوں	○	○	○	○	○	○

For peer review only

International Study – Iran (Persian)

Sociodemographic Information

۱. جنسیت:

- ◯ مرد
- ◯ زن
- ◯ دیگر
- ◯ ترجیح می‌دهم بیان نکنم

۲. سن (به سال)

____

۳. وضعیت تأهل

- ◯ هرگز ازدواج نکرده ام
- ◯ با کسی در رابطه‌ام(پارتنر دارم)
- ◯ ازدواج کرده‌ام
- ◯ متارکه
- ◯ مطلقه
- ◯ بیوه

۴. دین شما چیست؟

- ◯ بی‌دین
- ◯ اسلام
- ◯ مسیحیت
- ◯ هندو
- ◯ بودایی

For peer review only

For peer review only - http://bmjopen.bmj.com/site/about/guidelines.xhtml

○ ادیان یگر (لطفا مشخص فرمایید)____

۵. پیشینه قومی شما چیست؟ (می توانید بیش از یک مورد ذکر کنید)

____

۶. بطور روزمره به چه زبانی صحبت می‌کنید؟

____

دو پرسش زیر بطور خاص درباره مهارت شما در به کارگیری زبان انگلیسی می‌پرسد.

۶الف. من انگلیسی را خوب می‌فهمم

○ کاملا مخالف

○ مخالف

○ نه موافق نه مخالف

○ موافق

○ کاملا موافق

۶ب. من خوب انگلیسی حرف می‌زنم

○ هیچی

○ کمی

○ تا حدودی

○ خیلی

○ خیلی زیاد

۷. بالاترین مدرک تحصیلی‌تان چیست؟

○ فاقد مدرک رسمی (حضور در مدرسه، کلاس انگلیسی برای گویشوران به زبان های دیگر ESOL)

○ دبیرستان (مثلاً مدرک ان سی ای ایNational Certificate of Educational Achievement or NCEA ، دیپلم آی بی International Baccalaureate or IB، مدرک مدارس هر جایی)

For peer review only

For peer review only - http://bmjopen.bmj.com/site/about/guidelines.xhtml

○ مدرک دانشگاهی (مثل، گواهی نامه، دیپلم یا مدرک فنی – دوره های کمتر از 3 سال)

○ مدرک لیسانس(دوره ۳-۴ ساله)

○ مدرک تحصیلات تکمیل (دیپلم تحصیلات تکمیلی، کارشناسی ارشد، دکترا)

---

۸. وضعیت تحصیلی، کاری و شغلی فعلی (لطفاً گزینه های مربوط را علامت بزنید)

- ☐ تحصیل تمام وقت
- ☐ تحصیل پاره وقت
- ☐ بیکار
- ☐ استخدام تمام وقت
- ☐ استخدام پاره وقت
- ☐ بطور تمام وقت خوداشتغالم (کار و کاسبی خودم را دارم)
- ☐ بطور پاره‌وقت خوداشتغالم
- ☐ در مرخصی با حقوق پس از فرزنددار شدن بسر می‌برم
- ☐ والد خانه‌نشینم (سر کار نمی‌روم و بدون حقوقم)
- ☐ بازنشسته‌ام
- ☐ دیگر (لطفا مشخص کنید)________________________________

۹. شغل

____

۱۰. درآمد ماهانه خانوار (به واحد پول محلی)

____

۱۰الف. در مقایسه با متوسط درآمد، شما درآمد خانوار خودتان را چطور ارزیابی می‌کنید؟

○ زیرمتوسط

○ متوسط

○ بالای متوسط

۱۱. آیا شما در حال حاضر در قرنطینه‌اید؟

For peer review only

○ بلی

○ خیر

۱۲. آیا واکسن کووید-۱۹ زده‌اید؟

○ بلی

○ خیر

۱۲الف. اگر هنوز واکسن نزده‌اید، آیا دوست دارید در آینده واکسن کووید-۱۹ بزنید؟

○ بلی

○ خیر

۱۳. ما می‌دانیم که برخی افراد در گذشته قربانی حوادث آسیب‌زا شده‌اند که ممکن است کنار آمدن با مشکلات جدید را برایشان ساده‌تر یا سخت‌تر کند.

پیش از همه‌گیری کووید-۱۹ هرگز شاهد هیچ‌یک از موارد زیر بوده‌اید؟ یا خودتان هیچ‌کدام را تجربه کرده‌اید:
(لطفاً گزینه‌های انتخابی را تیک بزنید)

☐ بلایای طبیعی (مانند سیل، زمین لرزه و از این قبیل موارد)

☐ زندگی در یک منطقه جنگی یا در معرض درگیری نظامی

☐ بدبیاری دوران کودکی پیش از ۱۶ سالگی(نظیر عدم رسیدگی مناسب، قلدری، آزار جسمی یا جنسی)

☐ آزار جسمی یا جنسی پس از ۱۶ سالگی

☐ حادثه فیزیکی جدی

☐ سایر – لطفاً بگویید چه بوده است (اختیاری) ____

☐ هیچکدام از موارد بال

For peer review only

For peer review only - http://bmjopen.bmj.com/site/about/guidelines.xhtml

Covid Psychosocial Impacts Scale

تاثیرات شخصی

لطفا در صورتی که هر یک از موارد زیر در مواجهه با کووید-۱۹ برای شما اتفاق افتاده است به صورت بلی/خیر پاسخ دهید.
اگر پاسخ شما به هر موردی بلی است لطفا میزان استرسی که آن مورد برای شما داشته را بر روی طیف مربوط علامت بزنید.

۱. آیا شما به لحاظ شرایط خاصی دارید که ممکن است شما را دربرابر کووید-۱۹ آسیب‌پذیر سازد؟

- **خیر** ۰
- **بلی** هیچ استرسی ۱
- **بلی** استرس کم ۲
- **بلی** استرس متوسط ۳
- **بلی** استرس زیاد ۴
- **بلی** استرس خیلی زیاد ۵

۲. هیچ‌وقت احساس کرده‌اید که در خطر تماس با اشخاص آلوده به کووید-۱۹ قرار دارید؟

- **خیر** ۰
- **بلی** هیچ استرسی ۱
- **بلی** استرس کم ۲
- **بلی** استرس متوسط ۳
- **بلی** استرس زیاد ۴
- **بلی** استرس خیلی زیاد ۵

۳. فکر می‌کنید به کووید-۱۹ آلوده شده‌اید؟

- **خیر** ۰
- **بلی** هیچ استرسی ۱
- **بلی** استرس کم ۲
- **بلی** استرس متوسط ۳

For peer review only - http://bmjopen.bmj.com/site/about/guidelines.xhtml
For peer review only

○ **بلی** استرس زیاد ۴

○ **بلی** استرس خیلی زیاد ۵

۳الف. آیا تست کووید-۱۹ مثبت داشته‌اید؟

○ **خیر ۰**

○ **بلی** هیچ استرسی ۱

○ **بلی** استرس کم ۲

○ **بلی** استرس متوسط ۳

○ **بلی** استرس زیاد ۴

○ استرس خیلی زیاد

۳ب. آیا در حال حاضر نشانگان آلودگی به کووید-۱۹ دارید؟

○ **خیر ۰**

○ **بلی** هیچ استرسی ۱

○ **بلی** استرس کم ۲

○ **بلی** استرس متوسط ۳

○ **بلی** استرس زیاد ۴

○ **بلی** استرس خیلی زیاد ۵

تاثیرات خانوادگی

در صورتی که هر یک از موارد زیر برای اعضای خانواده شما در مواجهه با کووید-۱۹ اتفاق افتاده است لطفا با بلی/خیر پاسخ دهید.
اگر پاسخ شما به هر موردی بلی است، لطفا میزان استرسی که آن مورد برای شما داشته را بر روی طیف مربوط علامت بزنید.

۱. آیاهیچیک از اعضای درجه یک خانواده شما شرایط خاص دارد که آنان را نسبت به کووید-۱۹ آسیب‌پذیر بسازد؟

○ **خیر ۰**

○ **بلی** هیچ استرسی ۱

For peer review only - http://bmjopen.bmj.com/site/about/guidelines.xhtml

- **بلی** استرس کم ۲
- **بلی** استرس متوسط ۳
- **بلی** استرس زیاد ۴
- **بلی** استرس خیلی زیاد ۵

۲. آیا احساس می‌کنید هیچ‌یک از اعضای درجه یک خانواده شما به خاطر کارش در خطر تماس با افراد آلوده به کووید-۱۹ قرار دارد؟

- **خیر** ۰
- **بلی** هیچ استرسی ۱
- **بلی** استرس کم ۲
- **بلی** استرس متوسط ۳
- **بلی** استرس زیاد ۴
- **بلی** استرس خیلی زیاد ۵

۳. آیا فکر می‌کنید هیچ‌یک از اعضای درجه یک خانواده شما به کووید-۱۹ آلوده شده باشد؟

- **خیر** ۰
- **بلی** هیچ استرسی ۱
- **بلی** استرس کم ۲
- **بلی** استرس متوسط ۳
- **بلی** استرس زیاد ۴
- **بلی** استرس خیلی زیاد ۵

۳الف. هیچ‌یک از اعضای درجه یک خانواده شما تست کووید-۱۹ مثبت داشته است؟

- **خیر** ۰

For peer review only - http://bmjopen.bmj.com/site/about/guidelines.xhtml

- **بلی** هیچ استرسی ۱
- **بلی** استرس کم ۲
- **بلی** استرس متوسط ۳
- **بلی** استرس زیاد ۴
- **بلی** استرس خیلی زیاد ۵

۳ب. هیچیک از اعضای درجه یک خانواده شما در حال حاضر نشانگان آلودگی به کووید-۱۹ دارد؟

- **خیر ۰**
- **بلی** هیچ استرسی ۱
- **بلی** استرس کم ۲
- **بلی** استرس متوسط ۳
- **بلی** استرس زیاد ۴
- **بلی** استرس خیلی زیاد ۵

۳ج. هیچیک از اعضای درجه یک خانواده شما در اثر آلودگی به کووید-۱۹ درگذشته است؟

- **خیر ۰**
- **بلی** هیچ استرسی ۱
- **بلی** استرس کم ۲
- **بلی** استرس متوسط ۳
- **بلی** استرس زیاد ۴
- **بلی** استرس خیلی زیاد ۵

۴. آیا هیچیک از اعضای درجه یک خانواده شما در خارج از کشور هست که نگران باشید در خطر آلودگی به کووید-۱۹ باشد؟

- **خیر ۰**

For peer review only

For peer review only - http://bmjopen.bmj.com/site/about/guidelines.xhtml

- **بلی** هیچ استرسی ۱
- **بلی** استرس کم ۲
- **بلی** استرس متوسط ۳
- **بلی** استرس زیاد ۴
- **بلی** استرس خیلی زیاد ۵

۵. آیا هیچ‌یک از اعضای درجه یک خانواده شما در خارج از کشور هست که قادر به دیدن آنان نباشید یا آنان قادر به دیدار شما نباشند؟

- **خیر** ۰
- **بلی** هیچ استرسی ۱
- **بلی** استرس کم ۲
- **بلی** استرس متوسط ۳
- **بلی** استرس زیاد ۴
- **بلی** استرس خیلی زیاد ۵

کار و کووید

در صورتی که هر یک از موارد زیر در مواجهه با کاوید۱۹ برای شما یا برای هر یک از افراد خانوار شما (یعنی افرادی که با شما زیر یک سقف زندگی می‌کنند) اتفاق افتاده است، لطفا به صورت بلی/خیر/نامربوط پاسخ دهید.
اگر پاسخ شما به هر موردی بلی است لطفا میزان استرسی که آن مورد برای شما داشته را بر روی طیف مربوط علامت بزنید.

۱ .آیا شما یا هیچ‌یک از اعضای خانوارتان در مشاغلی که ضروری (مثلا در بخش خدمات درمانی، انتظامات، خدمات اضطراری، ارائه‌دهنده کالاها یا خدمات حیاتی) محسوب شده شاغل است؟

- **خیر** ۰
- **بلی** هیچ استرسی ۱
- **بلی** استرس کم ۲
- **بلی** استرس متوسط ۳
- **بلی** استرس زیاد ۴

For peer review only - http://bmjopen.bmj.com/site/about/guidelines.xhtml

- بلی استرس خیلی زیاد ۵

۲. آیا شما یا هیچ‌یک از اعضای خانوارتان تغییر عمده‌ای در ساعات کارش داشته است؟

- **خیر** ۰
- **بلی** هیچ استرسی ۱
- **بلی** استرس کم ۲
- **بلی** استرس متوسط ۳
- **بلی** استرس زیاد ۴
- **بلی** استرس خیلی زیاد ۵

۳. آیا شما یا هیچ‌یک از اعضای خانوارتان مجبور شده است که محل کارش را به خانه تغییر دهد؟

- **خیر** ۰
- **بلی** هیچ استرسی ۱
- **بلی** استرس کم ۲
- **بلی** استرس متوسط ۳
- **بلی** استرس زیاد ۴
- **بلی** استرس خیلی زیاد ۵

۴. آیا شما یا هیچ‌یک از اعضای خانوارتان مجبور شده است که نوع کارش را تغییر بدهد؟

- **خیر** ۰
- **بلی** هیچ استرسی ۱
- **بلی** استرس کم ۲
- **بلی** استرس متوسط ۳
- **بلی** استرس زیاد ۴

For peer review only - http://bmjopen.bmj.com/site/about/guidelines.xhtml

- **بلی** استرس خیلی زیاد ۵

۵. آیا شما یا هیچ‌یک از افراد خانوار شما از کار بی‌کار شده‌اید؟

- **نامربوط**
- **خیر** ۰
- **بلی** هیچ استرسی ۱
- **بلی** استرس کم ۲
- **بلی** استرس متوسط ۳
- **بلی** استرس زیاد ۴
- **بلی** استرس خیلی زیاد ۵

۶. آیا کسب‌وکار شما یا یکی از افراد خانوار شما یا کسب‌وکاری که شما یا یکی از افراد خانوار شما در آن کار می‌کنید در معرض تعطیلی قرار گرفته است؟

- **نامربوط**
- **خیر** ۰
- **بلی** هیچ استرسی ۱
- **بلی** استرس کم ۲
- **بلی** استرس متوسط ۳
- **بلی** استرس زیاد ۴
- **بلی** استرس خیلی زیاد ۵

در آمد

در صورتی که هر یک از موارد زیر برای خانوار شما در مواجهه با کووید-۱۹ اتفاق افتاده است لطفا با بلی/خیر پاسخ بدهید.
اگر پاسخ شما به هر موردی بلی است، لطفا میزان استرسی که آن مورد برای شما داشته را بر روی طیف مربوط علامت بزنید.

۱. خانوار شما از حیث مالی تنگنای عمده‌ای داشته است؟

- **خیر** ۰

For peer review only - http://bmjopen.bmj.com/site/about/guidelines.xhtml

○ **بلی** هیچ استرسی ۱

○ **بلی** استرس کم ۲

○ **بلی** استرس متوسط ۳

○ **بلی** استرس زیاد ۴

○ **بلی** استرس خیلی زیاد ۵

۲. آیا خانوار در پرداخت قبوض (برق، تلفن و از این قبیل) یا خرید اقلام غذایی بطور جدی دچار مشکل شده است؟

○ **خیر ۰**

○ **بلی** هیچ استرسی ۱

○ **بلی** استرس کم ۲

○ **بلی** استرس متوسط ۳

○ **بلی** استرس زیاد ۴

○ **بلی** استرس خیلی زیاد ۵

روابط

لطفا در صورتی که هر یک از موارد زیر در مواجهه با کووید-۱۹ برای شما اتفاق افتاده است به صورت بلی/خیر پاسخ دهید.
اگر پاسخ شما به هر موردی بلی است لطفا میزان استرسی که آن مورد برای شما داشته را بر روی طیف مربوط علامت بزنید.
در مواجهه با کووید-۱۹
۱. آیا کووید-۱۹ مشکلات و تنش‌ها را در خانواده افزایش داده است؟

○ **خیر ۰**

○ **بلی** هیچ استرسی ۱

○ **بلی** استرس کم ۲

○ **بلی** استرس متوسط ۳

○ **بلی** استرس زیاد ۴

○ **بلی** استرس خیلی زیاد ۵

For peer review only - http://bmjopen.bmj.com/site/about/guidelines.xhtml

در مواجهه با کووید-۱۹

۲. آیا کووید-۱۹ تماس (فیزیکی یا برخط/آنلاین) شما را با دوستان کاهش داده است؟

- **خیر** ۰
- **بلی** هیچ استرسی ۱
- **بلی** استرس کم ۲
- **بلی** استرس متوسط ۳
- **بلی** استرس زیاد ۴
- **بلی** استرس خیلی زیاد ۵

تغییر رفتارها و رویه‌های روزانه

لطفا در صورتی که هر یک از موارد زیر در مواجهه با کووید-۱۹ برای شما اتفاق افتاده است به صورت بلی/خیر پاسخ دهید.
اگر پاسخ شما به هر موردی بلی است لطفا میزان استرسی که آن مورد برای شما داشته را بر روی طیف مربوط علامت بزنید.

در مواجهه با کووید-۱۹

۱. همه‌گیری کووید-۱۹ منجر به تغییراتی در روال عادی زندگی روزمره شما (برای مثال، خواب، ورزش، اوقات فراغت، فعالیت‌های تفریحی یا سرگرمی) گشته است؟

- **خیر** ۰
- **بلی** هیچ استرسی ۱
- **بلی** استرس کم ۲
- **بلی** استرس متوسط ۳
- **بلی** استرس زیاد ۴
- **بلی** استرس خیلی زیاد ۵

در مواجهه با کووید-۱۹

۲. آیا کووید-۱۹ موجب شده است هنگام نیاز به خدمات بهداشتی و درمانی برای خودتان یا هر یک از اعضای خانواده، اقدامی نکنید یا با تاخیر اقدام کنید؟

- **خیر** ۰

For peer review only - http://bmjopen.bmj.com/site/about/guidelines.xhtml

○ **بلی** هیچ استرسی ۱

○ **بلی** استرس کم ۲

○ **بلی** استرس متوسط ۳

○ **بلی** استرس زیاد ۴

○ **بلی** استرس خیلی زیاد ۵

در مواجهه با کووید-۱۹
۳. آیا این شرایط مشارکت شما را در فعالیت‌های دینی (از طریق حضور فیزیکی یا برخط/آنلاین) کاهش داده است؟

○ **نامربوط**

○ **خیر ۰**

○ **بلی** هیچ استرسی ۱

○ **بلی** استرس کم ۲

○ **بلی** استرس متوسط ۳

○ **بلی** استرس زیاد ۴

○ **بلی** استرس خیلی زیاد ۵

در مواجهه با کووید-۱۹
۴. آیا تغییر عمده دیگری در عادات زندگی شما روی داده است؟

○ **خیر ۰**

○ **بلی (لطفا بفرمایید چه تغییر یا تغییراتی)۱** ____

اگر پاسخ شما به هر موردی بلی است لطفا میزان استرسی که آن مورد برای شما داشته را بر روی طیف مربوط علامت بزنید.

○ هیچ استرسی ۱

○ استرس کم ۲

○ استرس متوسط ۳

For peer review only - http://bmjopen.bmj.com/site/about/guidelines.xhtml

○ استرس زیاد ۴

○ استرس خیلی زیاد ۵

قرار گرفتن در معرض اطلاعات درباره کاوید-۱۹

۱. در رابطه با کووید-۱۹، از کدام منبع اطلاعاتی استفاده می‌کنید؟

☐ اخبار
☐ فیس‌بوک / توییتر
☐ وب‌سایت‌های بهداشتی
☐ گپ و گفت با دیگران
☐ منابع دیگر (لطفا مشخص فرمایید)____

۲. اخبار کووید-۱۹ چقدر موجب استرس شما می‌شود؟

○ هیچ استرسی ۱

○ استرس کم ۲

○ استرس متوسط ۳

○ استرس زیاد ۴

○ استرس خیلی زیاد ۵

تغییرات از زمان کووید-۱۹

لطفا برای موارد زیر میزان استرس‌تان را بر روی مقیاس زیر مشخص کنید.

مقایسه حال حاضر با پیش از همه‌گیری کووید-۱۹ ....
۱. سطح استرس خود را چطور ارزیابی می‌کنید؟

○ استرس بسیار کمتر ۱

○ استرس کمتر ۲

○ تقریبا به همان میزان ۳

○ استرس بیشتر ۴

○ استرس بسیار بیشتر ۵

For peer review only - http://bmjopen.bmj.com/site/about/guidelines.xhtml

مقایسه حال حاضر با پیش از همه‌گیری کووید-۱۹....
۲. بطور کلی سطح استرس در خانوار را چطور ارزیابی می‌کنید؟

- ○ استرس بسیار کمتر ۱
- ○ استرس کمتر ۲
- ○ تقریبا به همان میزان ۳
- ○ استرس بیشتر ۴
- ○ استرس بسیار بیشتر ۵

مقایسه حال حاضر با پیش از همه‌گیری کووید-۱۹.....
۳. سلامت روانی خود را چطور ارزیابی می‌کنید؟

- ○ خیلی بهتر ۱
- ○ بهتر ۲
- ○ تقریبا همانطور که بود ۳
- ○ بدتر ۴
- ○ خیلی بدتر ۵

مقایسه حال حاضر با پیش از همه‌گیری کووید-۱۹...
۴. بطور کلی سلامت روانی اعضای خانوار را چطور ارزیابی می‌کنید؟

- ○ خیلی بهتر ۱
- ○ بهتر ۲
- ○ تقریبا همانطور که بود ۳
- ○ بدتر ۴
- ○ خیلی بدتر ۵

For peer review only - http://bmjopen.bmj.com/site/about/guidelines.xhtml
For peer review only

K-10

در چهار هفته گذشته چه حس و حالی داشته اید؟

	هیچگاه<br>۱	به ندرت<br>۲	گاهی اوقات<br>۳	بیشتر وقت ها<br>۴	همیشه<br>۵
۱. در چهار هفته گذشته چقدر بی‌دلیل احساس خستگی کرده اید؟	○	○	○	○	○
۲. در چهار هفته گذشته چقدر احساس عصبی بودن کرده‌اید؟	○	○	○	○	○
۳. در چهار هفته گذشته هیچگاه شده است آنقدر عصبی باشید که هیچ چیز نتواند شما را آرام کند؟	○	○	○	○	○
۴. در چهار هفته گذشته چقدر احساس نومیدی کرده‌اید؟	○	○	○	○	○
۵. در چهار هفته گذشته چقدر احساس ناآرامی و بی‌قراری کرده‌اید؟	○	○	○	○	○
٦. در چهار هفته گذشته هیچگاه شده است آنقدر احساس ناآرامی کنید که نتوانید بی‌حرکت یک جا بنشینید؟	○	○	○	○	○
۷. در چهار هفته گذشته چقدر احساس افسردگی کرده‌اید؟	○	○	○	○	○
۸. در چهار هفته گذشته چقدر احساس کرده‌اید که همه کارها را دارید به زور انجام می‌دهید؟	○	○	○	○	○
۹. در چهار هفته گذشته چقدر احساس کرده‌اید که چنان غمگینید که هیچ چیز نمی‌تواند شما را سر حال بیاورد؟	○	○	○	○	○
۱۰. در چهار هفته گذشته چقدر احساس بی‌ارزش بودن کرده اید؟	○	○	○	○	○

For peer review only

PCL-5

فهرست زیر حاوی مشکلاتی است که بعضی از افراد در مواجهه با کووید-۱۹ تجربه کرده‌اند .لطفاً هر مشکل را با دقت بخوانید و سپس مشخص کنید که در یک ماه گذشته چقدر از آن مشکل رنجیده‌اید.

درماه گذشته چقدر موارد زیر شما را آزرده است؟

	اصلا ۰	بسیار کم ۱	متوسط ۲	زیاد ۳	بسیار زیاد ۴
۱- خاطرات تکراری، آزاردهنده و ناخواسته از آن تجربه استرس‌زا داشته‌اید؟	○	○	○	○	○
۲- کابوس‌های تکراری و آزاردهنده از آن تجربه استرس‌زا دیده اید؟	○	○	○	○	○
۳- احساس و عمل ناگهانی داشته‌اید به طوری که انگار آن تجربه استرس‌زا دوباره اتفاق افتاده باشد (در حالی که شما انگار واقعا آنجا حضور داشته‌اید و تجربه‌اش کرده‌اید)؟	○	○	○	○	○
۴- وقتی که یک اتفاق، آن تجربه استرس‌زا را در ذهن شما تداعی کرده احساس ناراحتی زیادی داشته‌اید؟	○	○	○	○	○
۵- هر گاه اتفاق یا چیزی، آن تجربه استرس‌زا را در ذهن شما تداعی کرده، واکنش فیزیکی شدیدی (مثل تپش قلب، مشکل تنفس و عرق کردن) داشته‌اید؟	○	○	○	○	○
٦- از خاطرات، افکار و احساسات مرتبط با آن تجربه استرس زا دوری کرده‌اید؟	○	○	○	○	○
۷- از نشانه‌های یادآور آن تجربه استرس‌زا (مثل اشخاص، مکان ها، گفتوگو ها، فعالیت‌ها، اشیا و یا موقعیت‌ها) دوری کرده‌اید؟	○	○	○	○	○
۸- بخش‌های مهم آن تجربه استرس‌زا را به سختی به یاد می آورید؟	○	○	○	○	○
۹- در مورد خودتان، بقیه مردم و یا جهان، عقاید خیلی منفی (مثل اینکه من بد هستم، یا مشکل جدی دارم، یا به هیچ کس نمی شود اعتماد کرد، یا جهان خیلی خطرناک است) داشته‌اید؟	○	○	○	○	○
۱۰- خود یا دیگری را در آن تجربه استرس‌زا و یا اتفاقات بعد از آن مقصر دانسته‌اید؟	○	○	○	○	○
۱۱- احساس‌های به شدت منفی مانند ترس، وحشت، خشم، گناه و خجالت داشته‌اید؟	○	○	○	○	○
۱۲- علاقه‌تان به فعالیتی که قبلا از آن لذت می‌بردید از بین رفته است؟	○	○	○	○	○
۱۳- احساس فاصله گرفتن و قطع ارتباط با افراد دیگر را داشته اید؟	○	○	○	○	○

For peer review only

۱۴- در تجربه کردن احساسات مثبت و خوب دچار مشکل شده اید؟ مثل ناتوانی در احساس شادی یا احساس دوست داشتن نزدیکان؟

۱۵- بدخلق و تحریک‌پذیر، دارای خشم شدید و رفتاری تهاجمی بوده‌اید؟

۱۶- بیش از حد ریسک‌پذیر بوده و یا کارهایی را که ممکن بود به شما آسیب برساند انجام داده‌اید؟

۱۷- بیش از حد محتاط «یا مراقب و یا گارد گرفته» بوده‌اید؟

۱۸- مضطرب بوده و به راحتی از جا می‌پریدید؟

۱۹- به سختی تمرکز داشته‌اید؟

۲۰- بد خوابیده و خواب خوب و سنگینی نداشته‌اید؟

For peer review only

For peer review only - http://bmjopen.bmj.com/site/about/guidelines.xhtml

WHO-5

در دو هفته گذشته چه حس و حالی داشته‌اید؟

**گذشته هفته ۲ طی**	**تمام اوقات**<br>**۵**	**بیشتر اوقات**<br>**۴**	**کمی بیشتر از نیمی از اوقات**<br>**۳**	**کمی کمتر از نیمی از اوقات**<br>**۲**	**بعضی وقت ها**<br>**۱**	**هیچ وقت**<br>**۰**
۱. شاد و خوش‌خلق بوده‌ام .	○	○	○	○	○	○
۲. احساس آرامش و راحتی داشته‌ام .	○	○	○	○	○	○
۳. احساس می‌کنم فعال و پرانرژی بوده‌ام .	○	○	○	○	○	○
۴. تر و تازه و سرحال بیدار شده‌ام .	○	○	○	○	○	○
۵. زندگی روزمره‌ام مملو از چیزهای مورد علاقه‌ام بوده است .	○	○	○	○	○	○

For peer review only

PTGI

هر یک از عبارات زیر به وقوع تغییری در زندگی شما اشاره دارد .با استفاده از مقیاس ارائه شده نشان دهید که آن تغییر تا چه اندازه در مواجهه با کووید-۱۹ حادث شده است.

	اصلاً هیچ ۰	خیلی کم ۱	تا حدودی ۲	به طور متوسط ۳	تقریبا زیاد ۴	بسیار زیاد ۵
۱. من اولویت‌هایم را درباره اینکه چه چیز در زندگی مهم است تغییر دادم.	○	○	○	○	○	○
۲. من برای زندگی خودم بیشتر ارزش قائلم.	○	○	○	○	○	○
۳. علائق جدیدی پیدا کردم.	○	○	○	○	○	○
۴. بیشتر احساس می‌کنم به خودم متکی ام.	○	○	○	○	○	○
۵. درک بهتری از موضوعات معنوی دارم.	○	○	○	○	○	○
٦. هر چه بیشتر احساس می کنم که می‌توانم در زمان گرفتاری‌ها روی مردم حساب کنم.	○	○	○	○	○	○
۷. مسیر جدیدی برای زندگی‌ام ایجاد کردم.	○	○	○	○	○	○
۸. با دیگران احساس نزدیکی بیشتری می‌کنم.	○	○	○	○	○	○
۹. بیشتر تمایل دارم که ابراز احساسات کنم.	○	○	○	○	○	○
۱۰. به خوبی می‌دانم که می‌توانم از پس سختی‌ها برآیم.	○	○	○	○	○	○
۱۱. می‌توانم کارهای بهتری در زندگی‌ام انجام دهم.	○	○	○	○	○	○
۱۲. بهتر می‌توانم بپذیرم که مسائل چگونه حل می‌شوند.	○	○	○	○	○	○
۱۳. بهتر می توانم قدر هر روز را بدانم.	○	○	○	○	○	○

For peer review only

For peer review only - http://bmjopen.bmj.com/site/about/guidelines.xhtml

۱۳. فرصت‌های جدیدی پدید آمده‌اند که در غیر این صورت هرگز پیش نمی آمدند.	○	○	○	○	○	○
۱۵. حس دلسوزی بیشتری نسبت به دیگران دارم.	○	○	○	○	○	○
۱٦. برای [حفظ] روابطم بیشتر تلاش می‌کنم.	○	○	○	○	○	○
۱٤. بیشتر احتمال دارد تلاش کنم چیزهایی که باید عوض شوند را عوض کنم.	○	○	○	○	○	○
۱۸. من ایمان مذهبی قوی‌تری دارم.	○	○	○	○	○	○
۱۹. کشف کردم از آنچه که فکرش را می‌کردم قوی‌ترم.	○	○	○	○	○	○
۲۰. کلی درباره اینکه.	○	○	○	○	○	○
۲۱. من راحت تر می‌پذیرم که به دیگران نیاز دارم.	○	○	○	○	○	○

For peer review only

STROBE Statement—checklist of items that should be included in reports of observational studies

	Item No	Recommendation	Page No
**Title and abstract**	1	(*a*) Indicate the study's design with a commonly used term in the title or the abstract	1,2
		(*b*) Provide in the abstract an informative and balanced summary of what was done and what was found	2
**Introduction**			
Background/rationale	2	Explain the scientific background and rationale for the investigation being reported	7
Objectives	3	State specific objectives, including any prespecified hypotheses	8
**Methods**			
Study design	4	Present key elements of study design early in the paper	9
Setting	5	Describe the setting, locations, and relevant dates, including periods of recruitment, exposure, follow-up, and data collection	10
Participants	6	(*a*) *Cohort study*—Give the eligibility criteria, and the sources and methods of selection of participants. Describe methods of follow-up<br>*Case-control study*—Give the eligibility criteria, and the sources and methods of case ascertainment and control selection. Give the rationale for the choice of cases and controls<br>*Cross-sectional study*—Give the eligibility criteria, and the sources and methods of selection of participants	9-11
		(*b*) *Cohort study*—For matched studies, give matching criteria and number of exposed and unexposed<br>*Case-control study*—For matched studies, give matching criteria and the number of controls per case	
Variables	7	Clearly define all outcomes, exposures, predictors, potential confounders, and effect modifiers. Give diagnostic criteria, if applicable	12
Data sources/ measurement	8*	For each variable of interest, give sources of data and details of methods of assessment (measurement). Describe comparability of assessment methods if there is more than one group	13
Bias	9	Describe any efforts to address potential sources of bias	15
Study size	10	Explain how the study size was arrived at	12
Quantitative variables	11	Explain how quantitative variables were handled in the analyses. If applicable, describe which groupings were chosen and why	12,13
Statistical methods	12	(*a*) Describe all statistical methods, including those used to control for confounding	12,13
		(*b*) Describe any methods used to examine subgroups and interactions	13
		(*c*) Explain how missing data were addressed	12
		(*d*) *Cohort study*—If applicable, explain how loss to follow-up was addressed<br>*Case-control study*—If applicable, explain how matching of cases and controls was addressed<br>*Cross-sectional study*—If applicable, describe analytical methods taking account of sampling strategy	12
		(*e*) Describe any sensitivity analyses	13

Continued on next page

For peer review only - http://bmjopen.bmj.com/site/about/guidelines.xhtml

**Results**			
Participants	13*	(a) Report numbers of individuals at each stage of study—eg numbers potentially eligible, examined for eligibility, confirmed eligible, included in the study, completing follow-up, and analysed	Not applicable
		(b) Give reasons for non-participation at each stage	Not applicable
		(c) Consider use of a flow diagram	Not applicable
Descriptive data	14*	(a) Give characteristics of study participants (eg demographic, clinical, social) and information on exposures and potential confounders	Not applicable
		(b) Indicate number of participants with missing data for each variable of interest	Not applicable
		(c) *Cohort study*—Summarise follow-up time (eg, average and total amount)	Not applicable
Outcome data	15*	*Cohort study*—Report numbers of outcome events or summary measures over time	Not applicable
		Case-control study—Report numbers in each exposure category, or summary measures of exposure	Not applicable
		Cross-sectional study—Report numbers of outcome events or summary measures	Not applicable
Main results	16	(*a*) Give unadjusted estimates and, if applicable, confounder-adjusted estimates and their precision (eg, 95% confidence interval). Make clear which confounders were adjusted for and why they were included	Not applicable
		(*b*) Report category boundaries when continuous variables were categorized	Not applicable
		(*c*) If relevant, consider translating estimates of relative risk into absolute risk for a meaningful time period	Not applicable
Other analyses	17	Report other analyses done—eg analyses of subgroups and interactions, and sensitivity analyses	Not applicable
**Discussion**			
Key results	18	Summarise key results with reference to study objectives	13
Limitations	19	Discuss limitations of the study, taking into account sources of potential bias or imprecision. Discuss both direction and magnitude of any potential bias	14.15
Interpretation	20	Give a cautious overall interpretation of results considering objectives, limitations, multiplicity of analyses, results from similar studies, and other relevant evidence	13,14
Generalisability	21	Discuss the generalisability (external validity) of the study results	15
**Other information**			
Funding	22	Give the source of funding and the role of the funders for the present study and, if applicable, for the original study on which the present article is based	17

*Give information separately for cases and controls in case-control studies and, if applicable, for exposed and unexposed groups in cohort and cross-sectional studies.

Note: An Explanation and Elaboration article discusses each checklist item and gives methodological background and published examples of transparent reporting. The STROBE checklist is best used in conjunction with this article (freely available on the Web sites of PLoS Medicine at http://www.plosmedicine.org/, Annals of Internal Medicine at

For peer review only - http://bmjopen.bmj.com/site/about/guidelines.xhtml

http://www.annals.org/, and Epidemiology at http://www.epidem.com/). Information on the STROBE Initiative is available at www.strobe-statement.org.

For peer review only

BMJ Open

Examining the Psychosocial Impacts of the COVID-19 Pandemic: An International Cross-sectional Study Protocol

Journal:	*BMJ Open*
Manuscript ID	bmjopen-2022-067886.R2
Article Type:	Protocol
Date Submitted by the Author:	14-Mar-2023

Complete List of Authors:	tanveer, sandila; University of Otago Christchurch, Department of Psychological Medicine<br>Schluter, Philip; University of Canterbury, School of Health Science; University of Queensland, School of Nursing, Midwifery and Social Work<br>Porter, Richard; University of Otago<br>Boden, Joseph; University of Otago<br>Beaglehole, Ben; University of Otago, Psychological Medicine<br>Sulaiman-Hill, Ruqayya; University of Otago Christchurch, Psychological Medicine<br>Dean, Shaystah; University of Otago Christchurch, Psychological Medicine; University of Otago Wellington Department of Medicine, Psychological Medicine<br>Bell, Romana; Australian National University, Department of Anthropology<br>Al-Hussainni, Wafaa N; Ibn Sina University of Medical and Pharmaceutical Sciences, Basic Sciences Deptartment<br>Arshi, Maliheh ; University of Social Welfare and Rehabilitation Science, Department of Social Work<br>Amer Nordin, Amer Siddiq; University of Malaya, Psychological Medicine<br>Dinç, Mehmet; Hasan Kalyoncu University, Department of Psychology<br>Khan, Mussarat ; International Islamic University, Department of Psychology<br>Khoshnami, Mohammad ; University of Social Welfare and Rehabilitation Science, Department of Social Work,<br>Majid Al-Masoodi, Muthana A; Cultural Relations Mustansiryah University, Department of Scholarships<br>Moghanibashi-Mansourieh, Amir; University of Social Welfare and Rehabilitation Science<br>Noruzi, Sara; Lorestan University of Medical Sciences,<br>Rahajeng, Anggi; Universitas Gadjah Mada<br>Shaikh, Shaista; Islamabad Model College for Girls (PostGraduate), Department of Psychology<br>Tanveer, Nisa; National Defence University, Islamabad Pakistan, Department of Peace and Conflict Sciences<br>Topçu, Feyza; Hasan Kalyoncu University, Department of Psychology<br>Yapan, Saadet; Hasan Kalyoncu University, Department of Psychology<br>Yunianto, Irfan; Faculty of Teacher Training and Education Universitas Ahmad Dahlan<br>Zoellner, Lori; University of Washington<br>Bell, Caroline; University of Otago, Department of Psychological Medicine

**Primary Subject Heading**:	Mental health
Secondary Subject Heading:	Public health
Keywords:	MENTAL HEALTH, COVID-19, PUBLIC HEALTH

Examining the Psychosocial Impacts of the COVID-19 Pandemic: An International Cross-sectional Study Protocol

Corresponding Author

Associate Professor Caroline Bell, Department of Psychological Medicine, University of Otago Christchurch, P.O. Box 4345, Christchurch 8140, New Zealand, caroline.bell@otago.ac.nz

Authors

Dr Sandila Tanveer, Department of Psychological Medicine, University of Otago, Christchurch, New Zealand

Professor Philip J. Schluter, Te Kaupeka Oranga, Faculty of Health, Te Whare Wānanga o Waitaha, University of Canterbury, Christchurch, New Zealand; and School of Clinical Medicine, Primary Care Clinical Unit, University of Queensland, Brisbane, Australia

Professor Richard J. Porter, Department of Psychological Medicine, University of Otago, Christchurch, New Zealand

Professor Joseph M. Boden, Department of Psychological Medicine, University of Otago, Christchurch, New Zealand

Dr Ben Beaglehole, Department of Psychological Medicine, University of Otago, Christchurch, New Zealand

For peer review only

For peer review only - http://bmjopen.bmj.com/site/about/guidelines.xhtml

Dr Ruqayya Sulaiman-Hill, Department of Psychological Medicine, University of Otago, Christchurch, New Zealand

Dr Shaystah Dean, Department of Psychological Medicine, University of Otago, Wellington, New Zealand

Romana Bell, Department of Anthropology, Australian National University, Canberra, ACT, Australia

Dr Wafaa N Al-Hussainni, Basic Sciences Department, Ibn Sina University of Medical and Pharmaceutical Sciences, Baghdad, Iraq

Dr Maliheh Arshi, Department of Social Work, University of Social Welfare and Rehabilitation Sciences, Tehran, Iran

Associate Professor Amer Siddiq Amer Nordin, Department of Psychological Medicine, Universiti Malaya, Kuala Lumpur, Malaysia

Dr Mehmet Dinç, Department of Psychology, Hasan Kalyoncu University, Gaziantep, Turkiye

Dr Mussarat Jabeen Khan, Department of Psychology, International Islamic University, Islamabad, Pakistan

Dr Mohammad Sabzi Khoshnami, Department of Social Work, University of Social Welfare and Rehabilitation Sciences, Tehran, Iran

Dr Muthana A Majid Al-Masoodi, Department of Scholarships and Cultural Relations, Mustansiryah University, Baghdad, Iraq

For peer review only

Dr Amir Moghanibashi-Mansourieh, Department of Social Work, University of Social Welfare and Rehabilitation Sciences, Tehran, Iran

Dr Sara Noruzi, Lorestan University of Medical Sciences, Khorramabad, Iran

Anggi Rahajeng, Faculty of Economics and Business, Universitas Gadjah Mada, Yogyakarta, Indonesia

Shaista Shaikh, Department of Psychology, Islamabad Model College for Girls (Postgraduate), Islamabad, Pakistan

Nisa Tanveer, Department of Peace and Conflict Sciences, National Defence University, Islamabad Pakistan

Feyza Topçu, Department of Psychology, Hasan Kalyoncu University, Gaziantep, Turkiye

Dr Saadet Yapan, Department of Psychology, Hasan Kalyoncu University, Gaziantep, Turkiye

Dr Irfan Yunianto, Faculty of Teacher Training and Education, Universitas Ahmad Dahlan, Yogyakarta, Indonesia

Professor Lori A Zoellner, Department of Psychology, University of Washington, Seattle, USA

Associate Professor Caroline Bell, Department of Psychological Medicine, University of Otago, Christchurch, New Zealand

For peer review only

For peer review only - http://bmjopen.bmj.com/site/about/guidelines.xhtml

Word Count: 3538

Keywords

Psychosocial impacts, mental health, wellbeing, COVID-19, low and middle-income countries, cross-cultural.

For peer review only

ABSTRACT

Introduction

The coronavirus disease (COVID-19) exposed people to significant and prolonged stress. The psychosocial impacts of the pandemic have been well recognised and reported in high-income countries (HICs) but it is important to understand the unique challenges posed by the COVID-19 in low and middle-income countries (LMICs) where limited international comparisons have been undertaken. This protocol was therefore devised to study the psychosocial impacts of the COVID-19 pandemic in seven LMICs using scales that had been designed for or translated for this purpose.

Methods and analysis

This cross-sectional study uses an online survey to administer a novel COVID Psychosocial Impacts Scale (CPIS) alongside established measures of psychological distress, post-traumatic stress, wellbeing, and post-traumatic growth in the appropriate language. Participants will include adults aged 18 years and above, recruited from Indonesia, Iraq, Iran, Malaysia, Pakistan, Somalia, and Turkey, with a pragmatic target sample size of 500 in each country.

Data will be analysed descriptively on sociodemographic and study variables. In addition, CPIS will be analysed psychometrically (for reliability and validity) to assess the suitability of use in a given context. Lastly, within-subjects and between-subjects analyses will be carried out using multi-level mixed-effect models to examine associations between key sociodemographic and study variables.

For peer review only - http://bmjopen.bmj.com/site/about/guidelines.xhtml

Ethics and dissemination

Ethical approval was granted by the Human Ethics Committee, University of Otago, New Zealand (Ref No. 21/102). In addition, international collaborators obtained local authorisation or ethical approval in their respective host universities before data collection commenced.

Participants will give informed consent before taking part. Data will be collected and stored securely on the University of Otago, New Zealand Qualtrics platform using an auto-generated non-identifiable letter-number string. Data will be available upon reasonable request. Findings will be disseminated by publications in scientific journals and/or conference presentations.

Registration details

The study is registered with Clinical Trial Registry (NCT05052333).

For peer review only

Strengths and limitations of this study

- This cross-sectional observational study will make a significant contribution to understanding the psychosocial impacts of the COVID-19 pandemic in seven LMICs with different pandemic impacts.
- This study will quantify psychometric measures in countries where there is currently limited access to newly developed scales relating to COVID-19 and associated measures of psychological distress, wellbeing, and post-traumatic growth in appropriate languages.
- The findings will provide psychometric information on the novel CPIS in differing pandemic contexts.
- There are limitations associated with selective recruitment and response rate in this study; which may limit the generalisability of the findings and comparability between groups.

For peer review only

INTRODUCTION

Coronavirus 2019 (COVID-19), was identified as an infectious disease by the World Health Organization (WHO) on 11 February 2020[1]. This new virus rapidly spread and was declared an international public health emergency on 30 January 2020 and a pandemic on 11 March 2020[1]. As of 31 January 2023, there have been 753,497,572 confirmed cases of COVID-19 including 6,813,301 deaths reported by WHO[2]; and likely many more as a result of under-reporting[3]. The COVID-19 pandemic has been complex in its origin, spread, impacts, and consequences. Specifically, COVID-19 exposed people to significant and prolonged stress from often overlapping concerns like health consequences of COVID-19, impacts of public health restrictions (e.g., lockdowns, social distancing, travel bans), financial losses, vaccination mandates, and associated societal divisions[4].

When examining the psychosocial impacts in a pandemic context, researchers experience key challenges not only with the choice of study design and measures but also in providing a multicultural perspective[5]. In this respect, researchers have obtained data using convenience[6,7] or representative sampling[8] by employing either cross-sectional[8] or longitudinal[7,9] design. Those studies have been carried out either on the general population[6-8] or on potentially vulnerable populations (such as the health workforce[10-12] or those with pre-existing risk factors such as having a previous history of mental disorders[9,13,14]). Most studies include self-report measures of wellbeing, psychological distress, depression, anxiety, and post-

traumatic stress disorder (PTSD)[15]. However, these standardised measures do not examine the particular impacts of COVID-19-related stressors and there has been a call for novel COVID-19-specific measures to be used alongside established psychometric scales[16]. In response, several COVID-19-specific scales have been developed which vary in focus and length. For example, one scale assesses fear associated with COVID-19 infection[17] (see also[6]), while the other one examined a wide range of impacts of the pandemic and public health restrictions on physical health, employment and finances, and family and social disruption[18]. Notably, the latter scale, in attempting to capture the wider psychosocial impacts, frequently reflects the context and country in which they were developed, limiting their more general applicability in other contexts. Researchers have also attempted to develop and validate brief screening tools for clinical research and practice; e.g., the 5-item Coronavirus Anxiety Scale[19] and the 7-item COVID-19 Anxiety Scale[20]. In addition, while the positive outcomes of the pandemic have been recognised[21], few studies formally assessed the positive impacts[22,23], which may limit a comprehensive understanding of the psychosocial impacts of COVID-19.

To date, the majority of the literature examining the psychosocial impacts of COVID-19 is from studies conducted in China, Europe, and the United States of America (USA) and a few multinational studies have been carried out among the general population. A multinational study that examined the psychosocial impacts of COVID-19 across several high-income countries (HICs) found generalized anxiety among 21.0% and major depression among 25.5% of their respondents[24]. Another study

For peer review only - http://bmjopen.bmj.com/site/about/guidelines.xhtml

that examined the psychological impacts of COVID-19 across several HICs and low- and middle-income countries (LMICs), reported high rates of anxiety (28.2%), stress (18.3%), and depression (26.6%) in their community-based samples[25] (see also[26] for depression prevalence in LMICs). Some multinational studies have reported significant differences in these psychological impacts across countries [26,27]. In comparison, studies investigating the psychosocial impacts in other regions, especially in LMICs are scarce, despite many of these areas being severely affected by COVID-19. The difficulties in evaluating the pandemic-related psychological impacts in these regions are compounded by the lack of standardised measures in the local languages of these countries, the absence of which limits the comparison of results between countries.

To address the issues identified above, we developed the 50-item COVID Psychosocial Impacts Scale (CPIS) to comprehensively assess the impacts of the pandemic including the adverse and positive personal, social, and economic consequences that followed. The construction of the scale was informed by our team’s experience assessing the psychosocial impacts of the Canterbury Earthquakes[28,29] and the Mosque Attacks in 2019[30]. Notably, the existing pandemic-related scales were also consulted in scale development[6,16-20]. The items and subscales of CPIS were modelled on the Social Readjustment Scale[31] assessing exposure to pandemic-related life events (disruptions to personal, family, employment, faith, education, and daily routines) and the resultant level of stress. It also included questions examining the potential positive consequences of the

COVID-19 pandemic. The CPIS was refined using the ‘group mind’ process[32], with colleagues from a range of countries and cultures (Bangladesh, Ghana, Pakistan, and Turkey) reviewing a draft of the questionnaire, with iterative improvements based on their comments. The applicability of the CPIS for use in a range of countries and cultures was duly considered through collaboration with international colleagues.

The 50-item CPIS has been validated in a non-representative New Zealand sample at two-time points (2020, 2022) when the population exposure to COVID-19 community infections varied markedly[33]. In that study, the CPIS was administered with a measure of psychological distress (Kessler Psychological Distress Scale, K10)[34] and wellbeing (World Health Organization Well-Being Index, WHO-5)[35]. Findings indicate a unidimensional structure within CPIS subscales while it correlates with psychological distress and general wellbeing is not only a proxy for these constructs[33]. Based on findings from the validation study, we found that some items were less important in future iterations of the CPIS. Therefore, we have refined CPIS from 50 to 32 items.

In addition to developing the CPIS, the research team took the opportunity to use resources that had already been deployed for the translation of the research instruments for the March 15th Project[30] to develop the CPIS into several different language versions (Arabic, Indonesian, Malay, Persian, Somali, Turkish, and Urdu). To ensure that translations captured psychometric meanings in a culturally

For peer review only - http://bmjopen.bmj.com/site/about/guidelines.xhtml

appropriate way, translations were carried out by proficient bilingual Research Assistants and interpreters who were familiar with the content and context, using parallel and back-to-back translations in an iterative process. These translations were then compared for internal consistency and examined for face validity.

Our planned programme of work is to examine the 32-item CPIS and translated psychometric measures in the countries of interest. This protocol describes a cross-sectional observational study to assess the psychosocial impacts of the COVID-19 pandemic in seven LMICs (Indonesia, Iran, Iraq, Malaysia, Pakistan, Somalia, and Turkey). It utilises an online survey that includes the 32-item CPIS and standardised measures of psychological distress, post-traumatic stress, wellbeing, and posttraumatic growth. Our findings will increase our understanding of the psychosocial impacts of the COVID-19 pandemic across several LMICs with differing rates of infection, morbidity, and mortality. Findings will also provide psychometric evidence on the novel the CPIS. As a result, our research will contribute to a growing body of mental health measures that can be used for cross-cultural comparison.

For peer review only

METHODS AND ANALYSIS

Study design

This is a cross-sectional observational study examining the psychosocial impacts of the COVID-19 pandemic being conducted in seven LMICs.

Measures

The measures have all been translated into the appropriate language for the country in which they will be administered (see supplementary file). They include the following.

Sociodemographic measures

Sociodemographic measures include age, gender, marital status, religion, ethnicity, self-reported English proficiency, highest level of education, occupation, study or work status, and monthly income (in local currency). On the recommendation of the international collaborators, some of the demographic questions may vary to ensure contextual appropriateness. For example, in Turkey, students do not study part-time so that option was removed from the item.

Prior trauma exposure

The exposure to previous trauma will also be sought to examine the effect of previous trauma on the psychosocial impacts of COVID-19. Participants will be asked to select as many as appropriate from a list of previous exposure to natural

For peer review only

disasters, war or military conflict, childhood adversity, physical or sexual assault, and serious physical accident.

COVID Psychosocial Impacts Scale (CPIS)

This is a novel 32-item measure used to examine the psychosocial impacts of the COVID-19 pandemic. The scale asks participants if they have experienced stressors in response to COVID-19 and then to indicate the magnitude of stress on a six-point scale scored from 0 = 'no', 1 = 'yes, no stress at all' to 5 = 'yes, a lot of stress'. An overall pandemic-related stress score can be calculated ranging from 0 to 160.

For peer review only

Kessler-10 (K10)

This is a 10-item measure assessing symptoms of psychological distress in the previous 30 days[34]. Each item is scored from 1 = ‘none of the time’ to 5 = ‘all of the time’. The raw score can range from 10 to 50; with scores 30 and above indicating severe mental distress[36].

WHO Well-Being Index (WHO-5)

This is a short 5-item measure assessing subjective psychological wellbeing in the previous two weeks[35]. Each item is scored from 0 = ‘at no time’ to 5 = ‘all of the time’. The raw score range is 0–25; this can be multiplied by 4 to give a final score, with 100 representing the highest subjective wellbeing rating.

PTSD Checklist for DSM-5 (PCL-5)

This is a 20-item measure assessing symptoms of PTSD in the past month anchored in response to COVID-19 as a potentially traumatic event. Each item is scored from 0 = ‘not at all’ to 4 = ‘extremely’; with a severity score range of 0–80[37].

Posttraumatic Growth Inventory (PTGI)

This is a 21-item measure assessing change following exposure to trauma[38], adapted to reflect how change occurred in your life as a result of COVID-19. There are five subscales (relating to others, new possibilities, personal strength, spiritual change, and appreciation of life). Each item is scored from 0 = ‘not at all’ to 5 = ‘a lot’

For peer review only

with a score range of 0–105, with higher scores reflecting positive transformation after the traumatic event.

Participants

Adults (aged 18 years and over) who reside in Indonesia, Iran, Iraq, Malaysia, Pakistan, Somalia, or Turkey will be recruited as participants. Anyone aged below 18 years and those not currently residing in the country of interest will be excluded from the study.

Study Status

Different sites are at different stages of project implementation including ethical approval pending (Iran); ethical approval obtained and recruiting participants (Somalia); and participant recruitment completed (Indonesia, Iraq, Malaysia, Pakistan, and Turkey).

Recruitment

The mode of recruitment will vary between countries, but will generally involve the distribution of the survey link using a participant mailing list or social media list held by each international collaborator at the host site. The local researchers will be using sampling techniques that are appropriate within their cultures and contexts, which for the most part resemble snowball sampling techniques.

Procedure

For peer review only - http://bmjopen.bmj.com/site/about/guidelines.xhtml

Participants receive a survey link via the recruitment method above. The online survey comprises a participant information sheet, consent form, and questionnaires. If a subject consents, they are asked to complete the online survey (which includes the self-report measures described above) using the Qualtrics XM online survey platform. The survey takes approximately 15 to 20 minutes to complete and is provided in the appropriate language for the site. Information is provided about local supports that can be accessed if the self-report questions were to cause distress.

Patient and Public Involvement

The project has been designed with community and general public involvement. Feedback has also been taken on project design from academics and the general public in the countries of interest.

Data management

To ensure the anonymity of the obtained dataset the following standard operating procedures are in place. Where applicable, only the international collaborators hold contact information for their participant pool. Each site has specific guidelines to preserve anonymity specified in their local ethics application. Following collection, data will be saved on the University of Otago, New Zealand Qualtrics platform using an auto-generated non-identifiable letter-number string. Importantly, the obtained data set will be completely anonymous as no identifiable information is obtained in the survey.

For peer review only - http://bmjopen.bmj.com/site/about/guidelines.xhtml

Data analysis

Incomplete survey responses will be removed from the analyses. Statistical analyses will be carried out to examine the psychosocial impacts of the COVID-19 pandemic in different countries and provide psychometric evidence using SPSS Version 28.0[39] and Stata Release 17[40]. Reporting will follow 'Strengthening the Reporting of Observational Studies in Epidemiology' (STROBE) guidelines[41].

Sample size estimation

As no prevalence of the psychosocial impacts of the COVID-19 pandemic in the general population of the countries of interest exists, no formal sample size calculations were conducted. The final target sample size of n=500 per country was pragmatically selected balancing the competing demands of maximizing statistical power, expedited cost-effective data collection processes, and international reach while simultaneously minimizing institutional burden[42].

Psychometric analyses

Reliability analyses will be carried out on each measure to determine the suitability of its use, employing item-total correlations and Cronbach's α as a measure of internal homogeneity and consistency. The construct validity of CPIS with WHO-5 and K10 will be examined using a correlation matrix.

Within-subjects analyses

Descriptive analysis will be carried out on socio-demographics (e.g., age, gender, ethnicity, religion, marital status, the highest level of education, work status, monthly

For peer review only - http://bmjopen.bmj.com/site/about/guidelines.xhtml

household income, exposure to previous trauma) and study variables (wellbeing, psychological distress, post-traumatic stress, post-traumatic growth and pandemic-related stress from the CPIS) to inform site-specific sociodemographic profile and psychosocial outcomes. To explore the relationship between socio-demographic and study variables, follow-up analyses will be carried out using a linear mixed model (also known as multi-level modelling) to pool predictor variables. This will be carried out for each of the seven sites individually.

For peer review only

Between-subjects analyses

To look at empirical distributions across countries, graphing superimposed Cumulative Distribution Function (cdf) will be used. In addition, to understand the role of demographic and study variables, multi-level modelling will be carried out in which participants will be nested within their countries – which will be treated as random effects to investigate the association between probable distress and wellbeing indicators and sociodemographic and pandemic-related variables. This approach allows individual and country-level factors to be included. Residual diagnostics and model assumptions will also be checked.

DISCUSSION

This is a cross-sectional observational research that examines the psychosocial impacts of the COVID-19 pandemic across seven LMICs using a novel CPIS and measures of wellbeing, psychological distress, post-traumatic stress, and post-traumatic growth. Despite LMICs being significantly impacted by COVID-19, few studies have been published from these regions. This study will therefore provide findings to address this important gap in the literature. The multi-centre design of the study will allow comparisons between the empirical distributions across countries. Furthermore, by including individual and country-level factors, multi-level modelling will enable us to understand the role of demographic and study variables. The findings have the potential to contribute to the understanding of the psychosocial

For peer review only - http://bmjopen.bmj.com/site/about/guidelines.xhtml

impacts of the COVID-19 pandemic in countries that have not been well studied and to allow cross-cultural, multi-site comparisons.

Several multinational studies have been carried out examining the psychosocial impacts of COVID-19 among the general population using either standardised measures alone[11,24,25,27,43] or in combination with novel instruments[26,44]. These studies mostly obtained data from non-representative samples (except one[24]) from HICs[24,45,46], LMICs[26], or across HICs and LMICs[25,27,43,44]. These studies have reported different rates in the prevalence of depression[11,24-27,43], anxiety[24,25,43], stress[11,25,27], and PTSD[11]. Few studies have examined the sociodemographic variables in relation to COVID-19, and have found an association of depression with younger age, gender (female), high levels of exposure, and stigmatization related to COVID-19[26,45]. Others have reported the association of stress with gender (female), marital status (single), no formal education level, religion, being exposed to a confirmed or suspected COVID-19 patient, and being forced to be quarantined[44,46].

Against this background, our study will comprehensively examine the psychosocial impacts of COVID-19 using standardised measures in combination with novel CPIS across seven LMICs examining both socio-demographics and study variables. Our study has the potential to make several contributions to the existing literature. The 32-item CPIS is a novel scale that comprehensively assesses the psychosocial impacts of the pandemic with the scale design allowing assessment of both the number of exposures to specific pandemic-related life events and the associated

For peer review only - http://bmjopen.bmj.com/site/about/guidelines.xhtml

level of stress related to each of these events. The psychosocial impacts of the pandemic-related life events will be examined cross-culturally in this study which distinguishes it from the existing multinational studies that mainly focus on the prevalence of mental health disorders and do not examine the adverse personal, social, and economic consequences that followed COVID-19 pandemic. Importantly, the multi-lingual nature of the study is an important contribution. An underlying factor to the lack of studies in LMIC has been the lack of availability of measures translated into appropriate languages, which this study addresses. Hence, our findings will contribute to a growing body of mental health measures that can be used for cross-cultural comparison.

In addition, the study will also correlate outcomes on the CPIS with other validated measures of wellbeing and psychological distress and provide essential psychometric information on scale reliability and validity. Although, using pandemic-related scales in combination with standardised measures has been adopted by a few studies[26,44], but no formal psychometric analyses of the measures' suitability in varying contexts were undertaken. Additionally, to date, only a few nationwide studies have formally assessed potential positive outcomes associated with the pandemic[21-23]. The findings from this study will therefore contribute for the first time to this developing literature of examining the positive impacts of the COVID-19 pandemic in a cross-cultural context.

There are, however, limitations associated with the proposed study design. This study makes use of a novel CPIS developed during the COVID-19 pandemic and a

For peer review only - http://bmjopen.bmj.com/site/about/guidelines.xhtml

cross-sectional design. As a consequence, there is no pre-pandemic comparison data. Repeated use of the CPIS over time and during different phases of the pandemic is needed to inform whether the CPIS is responsive to changing impacts of the pandemic over time. As the data will be gathered through an online survey, it will only include participants with access to the internet. As a result, the sample might be less representative of persons who are illiterate or have limited access to or proficiency with computers or mobile phones. Since none of the sites systematically recruit, there are limitations associated with selective recruitment. As a result, we are unable to generalize the findings to the population of the relevant sites. In addition, the exposure and response rate will vary between countries, making cross-country comparisons challenging. These limitations will be mitigated with the use of multi-level modelling techniques to understand country-wise or cross-cultural predictor variables to obtain meaningful findings. Even though random sampling would be ideal for providing an unbiased representative sample of the overall population, this is not always possible due to resource and time constraints, as the majority of multinational studies relied on non-representative samples. Lastly, to minimise participant burden, the survey did not include an assessment of certain relevant variables—such as COVID-19-related stigmatisation, resilience, religious coping, or pre-existing mental health conditions.

Summary

This cross-sectional observational research examines the psychosocial impacts of the COVID-19 pandemic across seven LMICs (Indonesia, Iran, Iraq, Malaysia,

For peer review only - http://bmjopen.bmj.com/site/about/guidelines.xhtml

Pakistan, Somalia, and Turkey) using a novel 32-item CPIS and standardised measures examining wellbeing, psychological distress, post-traumatic stress, and post-traumatic growth. The findings will contribute to the understanding of the psychosocial impacts of the COVID-19 pandemic in different LMICs. In addition, it will provide psychometric data regarding CPIS in the countries of interest. This is a major contribution as there is currently limited access to newly developed scales relating to COVID-19 and associated measures of distress in appropriate languages. As a result, our research will contribute to a growing trend of establishing mental health measures that allow cross-cultural comparisons.

Ethics and dissemination

Ethical approval was granted by the Human Ethics Committee, University of Otago, New Zealand (Ref No. 21/102). In addition, international collaborators obtained local authorisation or ethical approval in their respective host universities [Indonesia (Universitas Gadjah Mada Ethics Committee Ref. No. KE/UGM/008/EC/2021); Iraq (Ibn Sina University of Medical and Pharmaceutical Sciences, Institutional Review Board (IRB) - Ethical Committee); Malaysia (Universiti Malaya Research Ethics Committee, Ref. No. UM.TNC 2/UMREC); Pakistan (International Islamic University Islamabad, Institutional Ethical Review Committee, Ref. No. IIUI/ORIC/Ethical-certificate/2021); Somalia (Washington Human Subjects Division (HSD) IRB ID: STUDY00015135); and Turkey (Hasan Kalyoncu Üniversitesi Etik Ethics Committee Ref. No. E-97105791-050.01.01-2342)] before data collection commenced.

For peer review only

Participants will give informed consent to participate in the study before taking part. All data will be fully anonymized and collected and stored securely on the University of Otago, New Zealand Qualtrics platform using an auto-generated non-identifiable letter-number string. Data will be available upon reasonable request. Findings will be disseminated by publication in scientific journals and/or conference presentations.

Acknowledgement

We would like to thank the Research Assistants from the March 15 Project for their work in translating and back-translating study materials.

ABBREVIATIONS

cdf: Cumulative Distribution Function

CPIS: COVID Psychosocial Impacts Scale

HICs: High-income countries

K10: Kessler 10

LMIC: Low and middle-income countries

PCL-5: PTSD Checklist for DSM-5

PTGI: Post Traumatic Growth Inventory

WHO-5: WHO Well-Being Index

DECLARATIONS

Author Contributions

For peer review only - http://bmjopen.bmj.com/site/about/guidelines.xhtml

ST conceived the study, and CB, RS-H, RP, JB, BB, PJS, and SD contributed to the study design. ST, CB, and RS-H developed the protocol and selected the measures. ST was responsible for coordinating instrument translation and setting up the online component. ST, WNA-H, MA, ASAN, MD, MJK, MSK, MAMA-M, AM-M, SN, AR, SS, NT, FT, SY, IY, and LAZ were responsible for obtaining ethics approval and data acquisition. ST, RB, and CB drafted the paper. All authors read, critically revised, and approved the final version of the manuscript.

Funding Statement

There has been no funding for this study.

Competing Interests Statement

There are no competing interests.

For peer review only

REFERENCES

1 World Health Organization (2020). Listings of WHO's response to COVID-19. https://www.who.int/news/item/29-06-2020-covidtimeline

2 World Health Organization. WHO Coronavirus (COVID-19) dashboard. https://covid19.who.int/

3 World Health Organization. The true death toll of COVID-19: Estimating global excess mortality. https://www.who.int/data/stories/the-true-death-toll-of-covid-19-estimating-global-excess-mortality

4 Pfefferbaum B, North CS. Mental health and the Covid-19 pandemic. N. Engl. J. Med. 2020;383:510-512. doi:10.1056/NEJMp2008017

5 Bell C, Beaglehole B, Bell R, et al. Learning from previous disasters: potential pitfalls of epidemiological psychosocial research in the COVID-19 environment. Aust N Z J Psychiatry 2021;55(7):646-649. doi: 10.1177/0004867421998783.

6 Akan Y. Development of the "COVID-19 Psychological Impact Scale": a validity and reliability study. Curr Psychol 2022. doi:10.1007/s12144-022-02760-5

7 Fernández R, Crivelli L, Guimet N, et al. Psychological distress and mental health trajectories during the COVID-19 pandemic in Argentina: a longitudinal study. Sci Rep 2022;12(1). doi:10.1038/s41598-022-09663-2

For peer review only - http://bmjopen.bmj.com/site/about/guidelines.xhtml

8 Every-Palmer S, Jenkins M, Gendall P, et al. Psychological distress, anxiety, family violence, suicidality, and wellbeing in New Zealand during the COVID-19 lockdown: a cross-sectional study. PLoS One 2020;15(11):e0241658. doi:10.1371/journal.pone.0241658

9 Van Rheenen T, Meyer D, Neill E, et al. Mental health status of individuals with a mood-disorder during the COVID-19 pandemic in Australia: initial results from the COLLATE project. J Affect Disord 2020;275:69-77. doi:10.1016/j.jad.2020.06.037

10 Bell C, Williman J, Beaglehole B, et al. Challenges facing essential workers: a cross-sectional survey of the subjective mental health and well-being of New Zealand healthcare and 'other' essential workers during the COVID-19 lockdown. BMJ Open 2021;11(7):e048107. doi:10.1136/bmjopen-2020-048107

11 Chew N, Lee G, Tan B, et al. A multinational, multicentre study on the psychological outcomes and associated physical symptoms amongst healthcare workers during COVID-19 outbreak. Brain Behav Immun 2020;88:559-565. doi:10.1016/j.bbi.2020.04.049

12 Krishnamoorthy Y, Nagarajan R, Saya G, at al. Prevalence of psychological morbidities among general population, healthcare workers and COVID-19 patients amidst the COVID-19 pandemic: a systematic review and meta-analysis. Psychiatry Res 2020;293:113382. doi:10.1016/j.psychres.2020.113382

For peer review only - http://bmjopen.bmj.com/site/about/guidelines.xhtml

13 Bell C, Williman J, Beaglehole B, et al. Psychological distress, loneliness, alcohol use and suicidality in New Zealanders with mental illness during a strict COVID-19 lockdown. Aust N Z J Psychiatry 2021;56(7):800-810. doi: 10.1177/00048674211034317

14 Iasevoli F, Fornaro M, D'Urso G, et al. Psychological distress in patients with serious mental illness during the COVID-19 outbreak and one-month mass quarantine in Italy. Psychol Med 2020;51(6):1054-1056. doi:10.1017/S0033291720001841

15 Cénat J, Blais-Rochette C, Kokou-Kpolou C, et al. Prevalence of symptoms of depression, anxiety, insomnia, posttraumatic stress disorder, and psychological distress among populations affected by the COVID-19 pandemic: a systematic review and meta-analysis. Psychiatry Res 2021;295:113599. doi:10.1016/j.psychres.2020.113599

16 COVID-Minds. Recommended Scales 2020. https://www.COVIDminds.org/mental-health-measures

17 Ahorsu D, Lin C, Imani V, et al. The Fear of COVID-19 Scale: development and initial validation. Int J Ment Health Addict 2020;20(3):1537-1545. doi:10.1007/s11469-020-00270-8

18 CRISIS V0.1. The CoRonavIruS health Impact Survey 2020. http://www.crisissurvey.org/

For peer review only - http://bmjopen.bmj.com/site/about/guidelines.xhtml

19 Lee S. Coronavirus Anxiety Scale: a brief mental health screener for COVID-19 related anxiety. Death Stud 2020;44(7):393-401. doi:10.1080/07481187.2020.1748481

20 Silva W, de Sampaio Brito T, Pereira C. COVID-19 Anxiety Scale (CAS): development and psychometric properties. Curr Psychol 2020;41(8):5693-5702. doi:10.1007/s12144-020-01195-0

21 COVID-Minds. The psychological impact of COVID-19 2020. https://www.COVIDminds.org/post/february-update-the-psychological-impact-of-COVID-19

22 Beaglehole B, Williman J, Bell C, et al. Thriving in a pandemic: determinants of excellent wellbeing among New Zealanders during the 2020 COVID-19 lockdown; a cross-sectional survey. PLoS One 2022;17(3):e0262745. doi:10.1371/journal.pone.0262745

23 Jenkins M, Hoek J, Jenkin G, et al. Silver linings of the COVID-19 lockdown in New Zealand. PLoS One 2021;16(4):e0249678. doi:10.1371/journal.pone.0249678

24 Généreux M, Schluter PJ, Hung K, et al. One virus, four continents, eight countries: an interdisciplinary and international study on the psychosocial impacts of the Covid-19 pandemic among adults. Int J Environ Res Public Health 2020;17(22):8390. doi:10.3390/ijerph17228390

For peer review only

For peer review only - http://bmjopen.bmj.com/site/about/guidelines.xhtml

25 Chen SX, Ng JCK, Hui BPH, et al. Dual impacts of coronavirus anxiety on mental health in 35 societies. Sci Rep 2021;11(1):8925. doi:10.1038/s41598-021-87771-1

26 Cenat JM, Noorishad PG, Kokou-Kpolou CK, et al. Prevalence and correlates of depression during the COVID-19 pandemic and the major role of stigmatization in low- and middle-income countries: a multinational cross-sectional study. Psych Res 2021;297:113714. doi:10.1016/j.psychres.2021.113714

27 Tee M, Wang C, Tee C, et al. Impact of the COVID-19 pandemic on physical and mental health in lower and upper middle-income Asian countries: a comparison between the Philippines and China. Front Psychiatry 2020;11:568929. doi:10.3389/fpsyt.2020.568929

28 Fergusson D, Boden J, Horwood L, et al. Perceptions of distress and positive consequences following exposure to a major disaster amongst a well-studied cohort. Aust N Z J Psychiatry 2014;49(4):351-359. doi:10.1177/0004867414560652

29 McLeod GFH, Horwood LJ, Fergusson, DM, et al. Life-stress and reactivity by gender in a longitudinal birth cohort at 30 and 35 Years. Soc Psychiatry Psychiatr Epidemiol 2016;51(10):1385-1394. doi:10.1007/s00127-016-1254-z

30 Sulaiman-Hill R, Porter R, Tanveer S, et al. Psychosocial impacts on the Christchurch Muslim community following the 15 March terrorist attacks: a mixed-methods study protocol. BMJ Open 2021;11(10):e055413. doi:10.1136/bmjopen-2021-055413

For peer review only

31 Holmes T, Rahe R. The Social Readjustment Rating Scale. J Psychosom Res 1967;11(2):213-218. doi:10.1016/0022-3999(67)90010-4

32 Theiner G. A beginner's guide to group minds. In: Sprevak M, Kallestrup J, eds. New Waves in Philosophy of Mind. New Waves in Philosophy: London Palgrave Macmillan 2014. doi:10.1057/9781137286734_15

33 Tanveer S, Schluter PJ, Beaglehole B, et al. The COVID Psychosocial Impacts Scale: a reliable and valid tool to examine the psychosocial impacts of the covid-19 pandemic. Int J Environ Res Public Health (submitted).

34 Kessler R, Barker P, Colpe L, et al. Screening for serious mental illness in the general population. Arch Gen Psychiatry 2003;60(2):184. doi:10.1001/archpsyc.60.2.184

35 Topp C, Østergaard S, Søndergaard S, et al. The WHO-5 Well-Being Index: a systematic review of the literature. Psychother Psychosom 2015;84(3):167-176. doi:10.1159/000376585

36 Andrews G, Slade T. Interpreting scores on the Kessler Psychological Distress Scale (K10). Aust N Z J Public Health 2001;25(6):494-497. doi:10.1111/j.1467-842x.2001.tb00310.x

37 Weathers FW, Litz BT, Keane TM, et al. The PTSD Checklist for DSM-5 (PCL-5) – Standard [Measurement instrument]. The National Center for PTSD 2013. Available from https://www.ptsd.va.gov/

For peer review only

For peer review only - http://bmjopen.bmj.com/site/about/guidelines.xhtml

38 Tedeschi R, Cann A, Taku K, et al. The Posttraumatic Growth Inventory: a revision integrating existential and spiritual change. J Trauma Stress 2017;30(1):11-18. doi:10.1002/jts.22155

39 IBM Corp. IBM SPSS Statistics for Windows, Version 28.0. Armonk, NY: IBM Corp 2021.

40 StataCorp. Stata Statistical Software: Release 17. College Station, TX: StataCorp LLC 2021.

41 von Elm E, Altman DG, Egger M, et al. The Strengthening the Reporting of Observational Studies in Epidemiology (STROBE) statement: guidelines for reporting observational studies. Lancet 2007;370(9596):1453-7. doi:10.1016/S0140-6736(07)61602-X

42 Pye V, Taylor N, Clay-Williams R, et al. When is enough, enough? understanding and solving your sample size problems in health services research. BMC Res Notes 2016;9(1). doi:10.1186/s13104-016-1893-x

43 Ammar A, Mueller P, Trabelsi K, et al. Psychological consequences of COVID-19 home confinement: the ECLB-COVID19 multicenter study. PLoS One 2020;15(11):e0240204. doi:10.1371/journal.pone.0240204

44 TMGH-Global COVID-19 Collaborative. Perceived stress of quarantine and isolation during COVID-19 pandemic: a global survey. Front Psychiatry 2021;12:656664. doi:10.3389/fpsyt.2021.656664

For peer review only

For peer review only - http://bmjopen.bmj.com/site/about/guidelines.xhtml

45 Généreux M, Schluter PJ, Landaverde E, et al. The evolution in anxiety and depression with the progression of the pandemic in adult populations from eight countries and four continents. IJERPH 2021;18(9):4845. doi:10.3390/ijerph18094845

46 Schluter PJ, Généreux M, Landaverde E, Chan EYY, Hung KKC, Law R, Mok CPY, Murray V, O'Sullivan T, Qadar Z, Roy M. An eight country cross-sectional study of the psychosocial effects of COVID-19 induced quarantine and/or isolation during the pandemic. Sci Rep. 2022;12(1):13175. doi: 10.1038/s41598-022-16254-8

For peer review only

For peer review only - http://bmjopen.bmj.com/site/about/guidelines.xhtml

Contents

English Survey 5

Sociodemographic Information 5

Covid Psychosocial Impacts Scale 9

Personal Impacts 9

Family Impacts 10

Employment 13

Income 15

Relationships 16

Changes to Daily Routines and Behaviours 17

Exposure to Information about Covid-19 18

Personal and Family Stress 19

K-10 21

PCL-5 22

WHO-5 24

PTGI 25

International Study – Iraq (Arabic) 27

Sociodemographic Information 27

Covid Psychosocial Impacts Scale (CPIS) 31

الشخصي التأثير 31

الأسرة تأثير 32

العمل ١٩على-كوفيد جائحة تأثير 35

الدخل 37

العلاقات 38

اليومي الروتين و السلوك في تغيرات 39

١٩-كوفيد يخص فيما معلومات 41

١٩-كوفيد جائحة منذ تغيرات 41

K10 43

PCL-5 44

WHO-5 46

PGTI 47

For peer review only - http://bmjopen.bmj.com/site/about/guidelines.xhtml

International Study – Indonesia (Bahasa Indonesia) 49
Sociodemographic Information 49
Covid Psychosocial Impacts Scale 53
Dampak terhadap Pribadi 53
Dampak terhadap Keluarga 54
Pekerjaan 57
Pendapatan 59
Hubungan Keluarga 60
Perubahan dalam kegiatan dan perilaku sehari-hari 61
Paparan terhadap informasi mengenai Covid-19 63
Perubahan sejak Covid-19 63
K-10 65
PCL-5 66
WHO-5 68
PTGI 69
International Study – Malaysia (Malay) 71
Sociodemographic Information 71
Covid Psychosocail Impacts Scale 76
Kesan keatas diri sendiri 76
Kesan kepada keluarga 78
Pekerjaan 81
Pendapatan 83
Perhubungan (bersama insan lain) 84
Perubahan kepada Rutin dan Tingkah Laku Harian 85
Pendedahan kepada maklumat tentang Covid-19 87
Perubahan sejak Covid-19 87
K-10 89
PCL-5 90
WHO-5 92
PTGI 93
International Study – Somalia (Somali) 95
Sociodemographic Information 95

For peer review only - http://bmjopen.bmj.com/site/about/guidelines.xhtml

Covid Psychosocial Impacts Scale 99
Saameynta Shaqsiga 99
Sameeynta Qoyska 100
Shaqo 103
Dakhliga 105
Xiriika 106
Isbeddelka ku yimid nolol maalmeedka iyo dabeecadaha 107
Soo bandhigista macluumaadka ku saabsan Covid-19 108
Isbeddelada ku yimid Covid-19 dartiis 109
K-10 111
PCL-5 112
WHO-5 114
PTGI 115
International Study - Turkiye (Turkish) 117
Sociodemographic Information 117
Covid Psychosocial Impacts Scale 121
Kişisel etki 121
Aile etkisi 122
İş 125
Gelir 128
İlişkiler 128
Günlük rutin ve davranışlarda değişiklikler 129
Covid-19 hakkındaki bilgilere maruz kalma 131
Covid-19'dan bu yana değişiklikler 132
K-10 134
PCL-5 135
WHO-5 137
PTGI 138
International Study – Pakistan (Urdu) 140
Sociodemographic Information 140
Covid Psychosocial Impacts Scale 144
اثرات ذاتی 144

For peer review only - http://bmjopen.bmj.com/site/about/guidelines.xhtml

اثرات پر والوں خاندان 146
کام...... 148
آمدن...... 151
تعلقات 152
تبدیلیاں میں طرز عمل اور معمولات کے روزمرہ 152
معلومات میں بارے کے19 کوویڈ...... 154
تبدیلیاں والی ہونے بعد کے 19کوویڈ...... 155
K-10 157
PCL-5 158
WHO-5...... 160
PGTI 161
International Study – Iran (Persian)...... 163
Sociodemographic Information 163
Covid Psychosocial Impacts Scale 167
شخصی تاثیرات 167
خانوادگی تاثیرات 168
کووید و کار...... 171
درآمد 173
روابط...... 174
روزانه هایروبه و رفتارها تغییر...... 175
۱۹-کاوید درباره اطلاعات معرض در گرفتن قرار 177
۱۹-کووید زمان از تغییرات...... 177
K-10 179
PCL-5 180
WHO-5...... 182
PTGI 183

For peer review only

For peer review only - http://bmjopen.bmj.com/site/about/guidelines.xhtml

English Survey

Sociodemographic Information

1. Gender

- ◯ Male
- ◯ Female
- ◯ Other
- ◯ Prefer not to say

2. Age (in years) ______________________________

3. Marital status

- ◯ Single
- ◯ In a relationship
- ◯ Married
- ◯ Separated
- ◯ Divorced
- ◯ Widowed

4. What is your religion?

- ◯ No religion
- ◯ Islam
- ◯ Christianity
- ◯ Hinduism
- ◯ Buddhism
- ◯ Other (please specify) ____

For peer review only

5. What is your ethnic background? (You can mention more than one)

____

6. In your daily life, which languages do you speak fluently?

____

The following two questions ask specifically about your English language ability. 6a. I can understand English well

- ○ Strongly disagree 1
- ○ Disagree 2
- ○ Neither agree nor disagree 3
- ○ Agree 4
- ○ Strongly agree 5

6b. I speak English well

- ○ Not at all 1
- ○ A little 2
- ○ Somewhat 3
- ○ Quite a lot 4
- ○ A lot 5

7. What is your highest level of education?

- ○ No formal qualification (attended school, ESOL class)
- ○ Secondary school (e.g. NCEA, IB diploma, overseas school qualification)
- ○ Tertiary qualification (e.g. Certificate, Diploma or Trade qualification - less than 3 year course)
- ○ Bachelor degree (3-4 year course)
- ○ Post graduate degree (PG diploma, Master, Doctorate)

For peer review only - http://bmjopen.bmj.com/site/about/guidelines.xhtml

8. Current study or work status (Please tick whichever ones apply)

- ☐ Full-time student
- ☐ Part-time student
- ☐ Unemployed / Seeking work
- ☐ Full-time paid work
- ☐ Part-time paid work
- ☐ Full-time self employed
- ☐ Part-time self employed
- ☐ On employer paid parental leave
- ☐ Stay-at-home parent (unpaid)
- ☐ Retired
- ☐ Other (Please specify) ____

9. Occupation

____

10. Monthly household income (in local currency)

____

10a. Compared with the average income, how would you rate the income of your household?

- ○ Below average
- ○ Average
- ○ Above average

For peer review only

11. Are you currently in lockdown or stay-at-home order?

- ◯ Yes
- ◯ No

12. Have you had a Covid-19 vaccine?

- ◯ Yes
- ◯ No

12a. If not vaccinated, would you like to get the Covid-19 vaccine in the future?

- ◯ Yes
- ◯ No

13. We realise that some people may have been exposed to other traumatic incidents in the past which could make it easier or more difficult to deal with new problems.

Before the Covid-19 pandemic, have you ever witnessed or experienced any of the following: (Please tick as many as appropriate)

- ☐ Natural disaster (such as a flood, earthquake etc.)
- ☐ Living in a war zone or being exposed to military conflict
- ☐ Childhood adversity before the age of 16 (such as neglect, bullying, physical or sexual assault)
- ☐ Physical or sexual assault after the age of 16
- ☐ Serious physical accident
- ☐ Other – please specify (optional) ____
- ☐ None of the above

For peer review only

For peer review only - http://bmjopen.bmj.com/site/about/guidelines.xhtml

Covid Psychosocial Impacts Scale

Personal Impacts

For the following statements, please respond with Yes / No if this applies to you in response to Covid-19. If Yes, please indicate the amount of stress that this caused you on the scale below.

1. Do you have an underlying health condition that could make you vulnerable to Covid-19?

- No 0
- Yes No stress at all 1
- Yes A little stress 2
- Yes Moderately stressed 3
- Yes Quite a lot of stress 4
- Yes A lot of stress 5

2. Have you felt at risk of being exposed to someone with Covid-19?

- No 0
- Yes No stress at all 1
- Yes A little stress 2
- Yes Moderately stressed 3
- Yes Quite a lot of stress 4
- Yes A lot of stress 5

3. Do you think you have had Covid-19?

- No 0
- Yes No stress at all 1
- Yes A little stress 2
- Yes Moderately stressed 3

For peer review only

- Yes Quite a lot of stress 4
- Yes A lot of stress 5

3a. Have you had a positive Covid-19 test?

- No 0
- Yes No stress at all 1
- Yes A little stress 2
- Yes Moderately stressed 3
- Yes Quite a lot of stress 4
- Yes A lot of stress 5

3b. Do you have ongoing symptoms of Covid-19?

- No 0
- Yes No stress at all 1
- Yes A little stress 2
- Yes Moderately stressed 3
- Yes Quite a lot of stress 4
- Yes A lot of stress 5

Family Impacts

For the following statements, please respond with Yes / No if this applies to your close family members in response to Covid-19. If Yes, please indicate the amount of stress that this caused you on the scale below.

1. Does any close family member(s) have an underlying health condition that could make them vulnerable to Covid-19?

- No 0
- Yes No stress at all 1

For peer review only - http://bmjopen.bmj.com/site/about/guidelines.xhtml

- **Yes** A little stress 2
- **Yes** Moderately stressed 3
- **Yes** Quite a lot of stress 4
- **Yes** A lot of stress 5

2. Do you feel any close family member(s) is at risk of being exposed to someone with Covid-19?

- **No** 0
- **Yes** No stress at all 1
- **Yes** A little stress 2
- **Yes** Moderately stressed 3
- **Yes** Quite a lot of stress 4
- **Yes** A lot of stress 5

3. Do you think any close family member(s) have had Covid-19?

- **No** 0
- **Yes** No stress at all 1
- **Yes** A little stress 2
- **Yes** Moderately stressed 3
- **Yes** Quite a lot of stress 4
- **Yes** A lot of stress 5

3a. Has any close family member(s) tested positive for Covid-19?

- **No** 0
- **Yes** No stress at all 1
- **Yes** A little stress 2

For peer review only - http://bmjopen.bmj.com/site/about/guidelines.xhtml

For peer review only

- Yes Moderately stressed 3
- Yes Quite a lot of stress 4
- Yes A lot of stress 5

3b. Has any close family member(s) have ongoing symptoms of Covid-19?

- No 0
- Yes No stress at all 1
- Yes A little stress 2
- Yes Moderately stressed 3
- Yes Quite a lot of stress 4
- Yes A lot of stress 5

3c. Has any close family member(s) passed away from Covid-19?

- No 0
- Yes No stress at all 1
- Yes A little stress 2
- Yes Moderately stressed 3
- Yes Quite a lot of stress 4
- Yes A lot of stress 5

4. Do you have any close family member(s) overseas who you are worried are at risk of getting Covid-19?

- No 0
- Yes No stress at all 1
- Yes A little stress 2
- Yes Moderately stressed 3

For peer review only - http://bmjopen.bmj.com/site/about/guidelines.xhtml

For peer review only

○ **Yes** Quite a lot of stress 4

○ **Yes** A lot of stress 5

5. Do you have any close family member(s) overseas who you are not able to visit or who cannot visit you?

○ **No** 0

○ **Yes** No stress at all 1

○ **Yes** A little stress 2

○ **Yes** Moderately stressed 3

○ **Yes** Quite a lot of stress 4

○ **Yes** A lot of stress 5

Employment

For the following statements please respond with Yes / No / Not applicable if this applies to you or anyone in your household in response to Covid-19. If Yes, please indicate the amount of stress that this caused you on the scale below.

1. Are you or anyone in your household considered an essential worker (e.g., in healthcare, law enforcement, emergency services, provider of essential goods/services)?

○ **No** 0

○ **Yes** No stress at all 1

○ **Yes** A little stress 2

○ **Yes** Moderately stressed 3

○ **Yes** Quite a lot stress 4

○ **Yes** A lot of stress 5

2. Have you or anyone in your household had a major change in working hours?

○ **No** 0

○ **Yes** No stress at all 1

For peer review only - http://bmjopen.bmj.com/site/about/guidelines.xhtml

○ **Yes** A little stress 2

○ **Yes** Moderately stressed 3

○ **Yes** Quite a lot stress 4

○ **Yes** A lot of stress 5

3. Have you or anyone in your household had to change the place of work to home?

○ **No** 0

○ **Yes** No stress at all 1

○ **Yes** A little stress 2

○ **Yes** Moderately stressed 3

○ **Yes** Quite a lot stress 4

○ **Yes** A lot of stress 5

4. Have you or anyone in your household had to change to a different type of work?

○ **No** 0

○ **Yes** No stress at all 1

○ **Yes** A little stress 2

○ **Yes** Moderately stressed 3

○ **Yes** Quite a lot stress 4

○ **Yes** A lot of stress 5

5. Have you or anyone in your household lost their job?

○ **Not applicable** 9

○ **No** 0

○ **Yes** No stress at all 1

For peer review only

- Yes A little stress 2
- Yes Moderately stressed 3
- Yes Quite a lot stress 4
- Yes A lot of stress 5

6. Is a business you or anyone in your household owns or works in under threat of survival?

- Not applicable 9
- No 0
- Yes No stress at all 1
- Yes A little stress 2
- Yes Moderately stressed 3
- Yes Quite a lot stress 4
- Yes A lot of stress 5

Income

For the following statements please respond with Yes / No if this applies to your household in response to Covid-19. If Yes, please indicate the amount of stress that this caused you on the scale below.

1. Has your household had a major deterioration in financial circumstances?

- No 0
- Yes No stress at all 1
- Yes A little stress 2
- Yes Moderately stressed 3
- Yes Quite a lot of stress 4
- Yes A lot of stress 5

For peer review only - http://bmjopen.bmj.com/site/about/guidelines.xhtml

2. Has your household had a major deterioration in your ability to pay your bills or for food?

- ○ **No** 0
- ○ **Yes** No stress at all 1
- ○ **Yes** A little stress 2
- ○ **Yes** Moderately stressed 3
- ○ **Yes** Quite a lot of stress 4
- ○ **Yes** A lot of stress 5

Relationships

For the following statements please respond with Yes / No if this applies to you in response to Covid-19. If Yes, please indicate the amount of stress that this caused you on the scale below. In response to Covid-19.....

1. Has it increased difficulty and tension in the family?

- ○ **No** 0
- ○ **Yes** No stress at all 1
- ○ **Yes** A little stress 2
- ○ **Yes** Moderately stressed 3
- ○ **Yes** Quite a lot of stress 4
- ○ **Yes** A lot of stress 5

2. Has this decreased your contact with friends (physical or online)?

- ○ **No** 0
- ○ **Yes** No stress at all 1
- ○ **Yes** A little stress 2
- ○ **Yes** Moderately stressed 3
- ○ **Yes** Quite a lot of stress 4

For peer review only

For peer review only - http://bmjopen.bmj.com/site/about/guidelines.xhtml

- ◯ **Yes** A lot of stress 5

Changes to Daily Routines and Behaviours

For the following statements please respond with Yes / No / Not applicable, if this applies to you in response to Covid-19. If Yes, please indicate the amount of stress that this caused you on the scale below. In response to Covid-19.....

1. Has the Covid pandemic led to changes in your daily routine (for example, sleep, exercise, free time, pleasurable activities, or hobbies)?

- ◯ **No** 0
- ◯ **Yes** No stress at all 1
- ◯ **Yes** A little stress 2
- ◯ **Yes** Moderately stressed 3
- ◯ **Yes** Quite a lot of stress 4
- ◯ **Yes** A lot of stress 5

2. Has this caused you to delay / avoid seeking health care when you or a close family member needed it?

- ◯ **No** 0
- ◯ **Yes** No stress at all 1
- ◯ **Yes** A little stress 2
- ◯ **Yes** Moderately stressed 3
- ◯ **Yes** Quite a lot of stress 4
- ◯ **Yes** A lot of stress 5

3. Has this decreased your involvement with religious activities (in person or on-line)?

- ◯ **Not applicable** 9
- ◯ **No** 0
- ◯ **Yes** No stress at all 1

For peer review only

For peer review only - http://bmjopen.bmj.com/site/about/guidelines.xhtml

○ **Yes** A little stress 2

○ **Yes** Moderately stressed 3

○ **Yes** Quite a lot of stress 4

○ **Yes** A lot of stress 5

4. Has there been any other major change in your daily routines.

○ **No** 0

○ **Yes (Please specify)** 1 ____

If Yes, please indicate the amount of stress that this caused you on the scale below.

○ No stress at all 1

○ A little stress 2

○ Moderately stressed 3

○ Quite a lot of stress 4

○ A lot of stress 5

Exposure to Information about Covid-19

1. In regards to Covid-19, what is the source of information for you? (Please tick as many as appropriate.)

☐ News

☐ Facebook / Twitter

☐ Health websites

☐ Word of mouth

☐ Others (please specify) ____

For peer review only

For peer review only - http://bmjopen.bmj.com/site/about/guidelines.xhtml

2. How much stress is the news about Covid-19 causing you?

- No stress at all 1
- A little stress 2
- Moderately stressed 3
- Quite a lot of stress 4
- A lot of stress 5

Personal and Family Stress

For the following statements, please indicate on the given scale. Comparing now with before the Covid-19 pandemic...

1. How would you rate your personal level of stress?

- Considerably less stressed 1
- Somewhat less stressed 2
- About the same 3
- Somewhat more stressed 4
- Considerably more stressed 5

2. How would you rate the overall stress in your household?

- Considerably less stressed 1
- Somewhat less stressed 2
- About the same 3
- Somewhat more stressed 4
- Considerably more stressed 5

For peer review only

3. How would you rate your personal psychological wellbeing?

- ◯ Much better 1
- ◯ Better 2
- ◯ About the same 3
- ◯ Worse 4
- ◯ Much worse 5

4. How would you rate the overall psychological wellbeing of your household?

- ◯ Much better 1
- ◯ Better 2
- ◯ About the same 3
- ◯ Worse 4
- ◯ Much worse 5

For peer review only

For peer review only - http://bmjopen.bmj.com/site/about/guidelines.xhtml

K-10

The following ten questions ask about how you have been feeling in the last 4 weeks. For each question, select the option that best describes the amount of time you felt that way.

	None of the time 1	A little of the time 2	Some of the time 3	Most of the time 4	All of the time 5
1. In the last four weeks, about how often did you feel tired out for no good reason?	○	○	○	○	○
2. In the last four weeks, about how often did you feel nervous?	○	○	○	○	○
3. In the last four weeks, about how often did you feel so nervous that nothing could calm you down?	○	○	○	○	○
4. In the last four weeks, about how often did you feel hopeless?	○	○	○	○	○
5. In the last four weeks, about how often did you feel restless or fidgety?	○	○	○	○	○
6. In the last four weeks, about how often did you feel so restless you could not sit still?	○	○	○	○	○
7. In the last four weeks, about how often did you feel depressed?	○	○	○	○	○
8. In the last four weeks, about how often did you feel that everything was an effort?	○	○	○	○	○
9. In the last four weeks, about how often did you feel so sad that nothing could cheer you up?	○	○	○	○	○
10. In the last four weeks, about how often did you feel worthless?	○	○	○	○	○

For peer review only

PCL-5

Below is a list of problems which some people have experienced in response to Covid-19. Please read each problem carefully and then indicate how much you have been bothered by that problem in the past month. In the past month, how much were you bothered by:

	Not at all 0	A little bit 1	Moderately 2	Quite a bit 3	Extremely 4
1. Repeated, disturbing, and unwanted memories of the stressful experience?	○	○	○	○	○
2. Repeated, disturbing dreams of the stressful experience?	○	○	○	○	○
3. Suddenly feeling or acting as if the stressful experience were actually happening again (as if you were actually back there reliving it)?	○	○	○	○	○
4. Feeling very upset when something reminded you of the stressful experience?	○	○	○	○	○
5. Having strong physical reactions when something reminded you of the stressful experience (for example, heart pounding, trouble breathing, sweating)?	○	○	○	○	○
6. Avoiding memories, thoughts, or feelings related to the stressful experience?	○	○	○	○	○
7. Avoiding external reminders of the stressful experience (for example, people, places, conversations, activities, objects, or situations)?	○	○	○	○	○
8. Trouble remembering important parts of the stressful experience?	○	○	○	○	○
9. Having strong negative beliefs about yourself, other people, or the world (for example, having thoughts such as: I am bad, there is something seriously wrong with me, no one can be trusted, the world is completely dangerous)?	○	○	○	○	○
10. Blaming yourself or someone else for the stressful experience or what happened after it?	○	○	○	○	○
11. Having strong negative feelings such as fear, horror, anger, guilt, or shame?	○	○	○	○	○

For peer review only

For peer review only - http://bmjopen.bmj.com/site/about/guidelines.xhtml

12. Loss of interest in activities that you used to enjoy?	○	○	○	○	○
13. Feeling distant or cut off from other people?	○	○	○	○	○
14. Trouble experiencing positive feelings (for example, being unable to feel happiness or have loving feelings for people close to you)?	○	○	○	○	○
15. Irritable behaviour, angry outbursts, or acting aggressively?	○	○	○	○	○
16. Taking too many risks or doing things that could cause you harm?	○	○	○	○	○
17. Being “super alert” or watchful or on guard?	○	○	○	○	○
18. Feeling jumpy or easily startled?	○	○	○	○	○
19. Having difficulty concentrating?	○	○	○	○	○
20. Trouble falling or staying asleep?	○	○	○	○	○

For peer review only

For peer review only - http://bmjopen.bmj.com/site/about/guidelines.xhtml

WHO-5

Please indicate for each of the five statements which is closest to how you have been feeling over the last 2 weeks.

	All of the time 5	Most of the time 4	More than half of the time 3	Less than half of the time 2	Some of the time 1	At no time 0
1. I have felt cheerful and in good spirits	○	○	○	○	○	○
2. I have felt calm and relaxed	○	○	○	○	○	○
3. I have felt active and vigorous	○	○	○	○	○	○
4. I woke up feeling fresh and rested	○	○	○	○	○	○
5. My daily life has been filled with things that interest me	○	○	○	○	○	○

For peer review only

For peer review only - http://bmjopen.bmj.com/site/about/guidelines.xhtml

PTGI

Indicate for each of the statements below the degree to which this change occurred in your life as a result of Covid-19, using the following scale.

	Not at all 0	A little bit 1	Somewhat 2	Moderately 3	Quite a bit 4	A lot 5
1. I changed my priorities about what is important in life.	○	○	○	○	○	○
2. I have a greater appreciation for the value of my own life.	○	○	○	○	○	○
3. I developed new interests.	○	○	○	○	○	○
4. I have a greater feeling of self-reliance.	○	○	○	○	○	○
5. I have a better understanding of spiritual matters.	○	○	○	○	○	○
6. I more clearly see that I can count on people in times of trouble.	○	○	○	○	○	○
7. I established a new path for my life.	○	○	○	○	○	○
8. I have a great sense of closeness with others.	○	○	○	○	○	○
9. I am more willing to express my emotions.	○	○	○	○	○	○
10. I know better that I can handle difficulties.	○	○	○	○	○	○
11. I am able to do better things with my life.	○	○	○	○	○	○
12. I am better able to accept the way things work out.	○	○	○	○	○	○
13. I can better appreciate each day.	○	○	○	○	○	○
14. New opportunities are available which wouldn't have been otherwise.	○	○	○	○	○	○

For peer review only

For peer review only - http://bmjopen.bmj.com/site/about/guidelines.xhtml

15. I have more compassion for others.
16. I put more effort into my relationships.
17. I am more likely to try to change things which need changing.
18. I have a stronger religious faith.
19. I discovered that I'm stronger than I thought I was.
20. I learned a great deal about how wonderful people are.
21. I better accept needing others.

For peer review only

International Study – Iraq (Arabic)

Sociodemographic Information

١- الجنس

- ذكر
- أنثى
- اخرى
- أُفَضل عدم الإجابة

٢- بالسنوات (الفئة العمرية)

____

٣- الحالة الإجتماعية

- أعزب
- مرتبط
- متزوج
- منفصل
- مطلق
- أرمل

٤- ماهي ديانتك؟

- ليس لدي ديانة
- الإسلام
- المسيحية
- الهندوسية
- البوذية

For peer review only

○ أخرى (برجاء الشرح) ___________________________

٥- ما هي أُصولك العرقية؟ (يمكن أن تذكر أكثر من واحدة)

____

٦- ماهي اللغات التي تتحدثها بطلاقة في حياتك اليومية؟

____

السؤالان التاليان يحددان مدى قدرتك في اللغة الإنجليزية.
٦أ- يمكنني فهم اللغة الإنجليزية جيداً

○ لا أوافق بشدة 1

○ لا أوافق 2

○ لا اعلم 3

○ أوافق 4

○ أوافق بشدة 5

٦ب- يمكنني تحدث الإنجليزية جيداً

○ لا على الإطلاق 1

○ قليلاً 2

○ بقدر معتدل 3

○ كثيراً 4

○ كثيراً جداً 5

٧- ما هي مؤهلاتك العلمية؟

○ لا يوجد مؤهل علمي (التحقت بالمدرسة، فصل تعليم الكبار غير الناطقين بالإنجليزية)

○ المرحلة الثانوية (على سبيل المثال، الشهادة الوطنية النيوزيلاندية للتحصيل التعليمي، دبلوم، والمؤهلات المدرسية من خارج نيوزيلاندا)

For peer review only - http://bmjopen.bmj.com/site/about/guidelines.xhtml

- ○ مؤهل جامعي (على سبيل المثال، شهادة، دبلوم أو مؤهل تجاري -دورة أقل من 3 سنوات)
- ○ البكالوريوس (دورة 4-3 سنوات)
- ○ الدراسات العليا (دبلوم الدراسات العليا، الماجستير، الدكتوراة)

٨ـ وضعك الدراسي أو الوظيفي (يرجى وضع علامة على كل الحالات التي تنطبق عليك)

- ☐ يدرس بدوام كامل
- ☐ يدرس بدوام جزئي
- ☐ لا يعمل / يبحث عن عمل
- ☐ يعمل بدوام كامل (وظيفة واحدة او أكثر)
- ☐ يعمل بدوام جزئي (وظيفة واحدة او أكثر)
- ☐ يعمل لحسابة بدوام كامل
- ☐ يعمل لحسابة بدوام جزئي
- ☐ أجازة امومة مدفوعة الأجر
- ☐ رب - ربة منزل (غير مدفوع الأجر)
- ☐ متقاعد
- ☐ أخرى (برجاء الشرح) ______________________________

٩ـ الوظيفة

____

١٠ـ دخل الأسرة الشهري (بالعملة المحلية)

____

١٠أـ مقارنة بمتوسط الدخل، ما هو تقيمك لدخل أسرتك؟

- ○ أقل من المتوسط
- ○ متوسط
- ○ أعلى من المتوسط

١١ـ هل أنت حالياً في وضع العزل بسبب جائحة كوفيد-١٩؟

- ○ نعم
- ○ لا

For peer review only - http://bmjopen.bmj.com/site/about/guidelines.xhtml

١٢- هل تلقيت مطعوم كوفيد-١٩؟

○ نعم

○ لا

١٢أ- إذا لم تتلقى مطعوم كوفيد-١٩، هل ترغب في الحصول عليه في المستقبل؟

○ نعم

○ لا

١٣- ندرك أن بعض الأشخاص قد تعرضوا إلى أحداث صادمة في الماضي قد تجعل التعامل مع المشكلات الأخرى أسهل أو أصعب في التعامل مع اي مشكلات جديدة. قبل جائحة كوفيد-١٩ هل سبق أن شاهدت أو تعرضت إلى واحدة أو أكثر من هذه المواقف :(يرجى وضع علامة أو اكثر إن وجد)

- ☐ كوارث طبيعية (مثل فيضان، زلزال أو ما إلى ذلك)
- ☐ كنت تعيش في منطقة حروب أو تعرضت إلى نزاع عسكري
- ☐ محنة في الطفولة قبل سن ١٦عاماً (مثل الإهمال، التنمرأو الإعتداء الجنسي)
- ☐ اعتداء بدني أو جنسي بعد سن ١٦عاماً
- ☐ حادث بدني خطير
- ☐ أخرى - برجاء الشرح (أختياري) ________________________________
- ☐ ليس مما سبق

For peer review only

For peer review only - http://bmjopen.bmj.com/site/about/guidelines.xhtml

Covid Psychosocial Impacts Scale (CPIS)

التأثير الشخصي

يرجى الإجابة (بنعم / لا) إذا كان لجائحة كوفيد-١٩ أثرعليك من خلال الإسئلة التالية. إذا كانت اجابتك بنعم، يرجى تحديد مقدار الضغط الذي تشعر به من خلال هذا المقياس أدناه.

١- هل لديك اي مشاكل صحية تجعلك عرضة لمرض كوفيد-١٩؟

- لا 0
- نعم لا يوجد ضغط اطلاقاً 1
- نعم قليلاً 2
- نعم احيانا 3
- نعم غالباً جداً 4
- نعم كثيراً جداً 5

٢- هل شعرت بخطر التعرض لشخص مصاب بكوفيد-١٩؟

- لا 0
- نعم لا يوجد ضغط اطلاقاً 1
- نعم قليلاً 2
- نعم احيانا 3
- نعم غالباً جداً 4
- نعم كثيراً جداً 5

٣- هل أُصبت بمرض كوفيد (كورونا)؟

- لا 0
- نعم لا يوجد ضغط اطلاقاً 1
- نعم قليلاً 2
- نعم احيانا 3

For peer review only - http://bmjopen.bmj.com/site/about/guidelines.xhtml

نعم غالباً جداً 4

نعم كثيراً جداً 5

٣أ- هل كانت نتيجة فحص أختبار كوفيد-١٩ إيجابية؟

لا 0

نعم لا يوجد ضغط اطلاقاً 1

نعم قليلاً 2

نعم احيانا 3

نعم غالباً جداً 4

نعم كثيراً جداً 5

٣ب -هل تعاني من أعراض مستمرة بسبب كوفيد-١٩؟

لا 0

نعم لا يوجد ضغط اطلاقاً 1

نعم قليلاً 2

نعم احيانا 3

نعم غالباً جداً 4

نعم كثيراً جداً 5

تأثير الأسرة

يرجى الإجابة (بنعم / لا) إذا كان لجائحة كوفيد-١٩ أثرعلى أسرتك من خلال الإسئلة التالية. إذا كانت اجابتك بنعم، يرجى تحديد مقدار الضغط الذي تشعر به من خلال هذا المقياس أدناه.

١- هل يعاني احد أفراد أسرتك من اي مشاكل صحية تجعله عرضة لمرض كوفيد-١٩؟

لا 0

نعم لا يوجد ضغط اطلاقاً 1

For peer review only - http://bmjopen.bmj.com/site/about/guidelines.xhtml

○ نعم قليلاً 2

○ نعم احيانا 3

○ نعم غالباً جداً 4

○ نعم كثيراً جداً 5

٢ـ هل تشعر ان احد أفراد أسرتك معرض للاصابة لكوفيد-١٩ من خلال الإختلاط بأحد أفراد العمل؟

○ لا 0

○ نعم لا يوجد ضغط اطلاقاً 1

○ نعم قليلاً 2

○ نعم احيانا 3

○ نعم غالباً جداً 4

○ نعم كثيراً جداً 5

٣- هل أُصيب احد أفراد أسرتك بمرض كوفيد-١٩؟

○ لا 0

○ نعم لا يوجد ضغط اطلاقاً 1

○ نعم قليلاً 2

○ نعم احيانا 3

○ نعم غالباً جداً 4

○ نعم كثيراً جداً 5

٣أ- هل كانت نتيجة فحص كوفيد-١٩ إيجابية لأحد أفراد أسرتك؟

○ لا 0

○ نعم لا يوجد ضغط اطلاقاً 1

For peer review only - http://bmjopen.bmj.com/site/about/guidelines.xhtml

نعم قليلاً 2

نعم احيانا 3

نعم غالباً جداً 4

نعم كثيراً جداً 5

٣ب - هل أحد أفراد أسرتك مازال يعاني من أعراض كوفيد-١٩؟

لا 0

نعم لا يوجد ضغط اطلاقاً 1

نعم قليلاً 2

نعم احيانا 3

نعم غالباً جداً 4

نعم كثيراً جداً 5

٣ج - هل توفى احد أفراد أسرتك كنتيجة لكوفيد-١٩؟

لا 0

نعم لا يوجد ضغط اطلاقاً 1

نعم قليلاً 2

نعم احيانا 3

نعم غالباً جداً 4

نعم كثيراً جداً 5

٤- هل لديك احد من أفراد أسرتك مقيم بالخارج و تشعر بالقلق عليهم من خطر الإصابة بمرض كوفيد-١٩؟

لا 0

For peer review only - http://bmjopen.bmj.com/site/about/guidelines.xhtml

- ○ نعم لا يوجد ضغط اطلاقاً 1
- ○ نعم قليلاً 2
- ○ نعم احيانا 3
- ○ نعم غالباً جداً 4
- ○ نعم كثيراً جداً 5

٥- هل لديك احد من أفراد أسرتك يقيم بالخارج و لا يمكنك زيارتهم و لا يمكنهم زيارتك؟

- ○ لا 0
- ○ نعم لا يوجد ضغط اطلاقاً 1
- ○ نعم قليلاً 2
- ○ نعم احيانا 3
- ○ نعم غالباً جداً 4
- ○ نعم كثيراً جداً 5

تأثير جائحة كوفيد-١٩ على العمل

يرجى الإجابة (بنعم / لا / لا أفضل الإجابة) إذا كان لجائحة كوفيد-١٩ أثرعليك او على احد أفراد أسرتك المقيمين معك بالمنزل من خلال الإسئلة التالية. إذا كانت اجابتك بنعم، يرجى تحديد مقدار الضغط الذي تشعر به من خلال هذا المقياس أدناه.

١- هل تعتبر أنت أو احد أفراد أسرتك المقيمين معك بالمنزل من العمال الأساسيين (مثل عمال الرعاية الصحية، شؤون القانون، خدمات الطوارئ أو مزودي السلع و الخدمات الأساسية)؟

- ○ لا 0
- ○ نعم لا يوجد ضغط اطلاقاً 1
- ○ نعم قليلاً 2
- ○ نعم احيانا 3
- ○ نعم غالباً جداً 4

For peer review only - http://bmjopen.bmj.com/site/about/guidelines.xhtml

نعم كثيراً جداً 5

٢- هل كان هناك تغير كبير في ساعات العمل لك أو احد أفراد أسرتك المقيمين معك بالمنزل؟

- لا 0
- نعم لا يوجد ضغط اطلاقاً 1
- نعم قليلاً 2
- نعم احيانا 3
- نعم غالباً جداً 4
- نعم كثيراً جداً 5

٣- هل اضطررت أنت أو احد أفراد أسرتك المقيمين معك بالمنزل إلى تغيير مكان العمل للعمل من المنزل؟

- لا 0
- نعم لا يوجد ضغط اطلاقاً 1
- نعم قليلاً 2
- نعم احيانا 3
- نعم غالباً جداً 4
- نعم كثيراً جداً 5

٤- هل اضطررت أنت أو احد أفراد أسرتك المقيمين معك بالمنزل إلى تغيير طبيعة العمل؟

- لا 0
- نعم لا يوجد ضغط اطلاقاً 1
- نعم قليلاً 2
- نعم احيانا 3
- نعم غالباً جداً 4

For peer review only - http://bmjopen.bmj.com/site/about/guidelines.xhtml

- نعم كثيراً جداً 5

٥ -هل فقدت أنت او احد أفراد أسرتك المقيمين معك بالمنزل؟

- لا أُفضل الإجابة
- لا 0
- نعم لا يوجد ضغط اطلاقاً 1
- نعم قليلاً 2
- نعم احيانا 3
- نعم غالباً جداً 4
- نعم كثيراً جداً 5

٦- هل الأعمال التجارية التي تمتلكها او تعمل بها أنت او احد أفراد أسرتك المقيمين معك بالمنزل أصبحت مهددة بالبقاء؟

- لا أُفضل الإجابة
- لا 0
- نعم لا يوجد ضغط اطلاقاً 1
- نعم قليلاً 2
- نعم احيانا 3
- نعم غالباً جداً 4
- نعم كثيراً جداً 5

الدخل

يرجى الإجابة (بنعم / لا) إذا حدث ذلك لاحد أفراد أسرتك المقيمين معك بالمنزل كنتيجة لجائحة كوفيد-١٩ من خلال الإسئلة التالية. إذا كانت اجابتك بنعم ، فيرجى تحديد مقدار الضغط الذي تشعر به من خلال هذا المقياس أدناه.

١- هل تعرض أحد من أفراد أسرتك لتدهور كبير في الحالة المالية؟

- لا 0

For peer review only - http://bmjopen.bmj.com/site/about/guidelines.xhtml

نعم لا يوجد ضغط اطلاقاً 1

نعم قليلاً 2

نعم احيانا 3

نعم غالباً جداً 4

نعم كثيراً جداً 5

٢- هل تعرض احد أفراد أسرتك المقيمين لتدهور كبير في عدم القدرة على دفع الفواتير او توفير الإحتياجات الغذائية؟

لا 0

نعم لا يوجد ضغط اطلاقاً 1

نعم قليلاً 2

نعم احيانا 3

نعم غالباً جداً 4

نعم كثيراً جداً 5

العلاقات

يرجى الإجابة (بنعم / لا) إذا كان لجائحة كوفيد-١٩ أثرعليك من خلال الإسئلة التالية.
إذا كانت اجابتك بنعم، يرجى تحديد مقدار الضغط الذي تشعر به من خلال هذا المقياس أدناه. كنتيجة لكوفيد-١٩....
١- هل ادى ذلك لزيادة المشاكل والتوتر داخل العائلة؟

لا 0

نعم لا يوجد ضغط اطلاقاً 1

نعم قليلاً 2

نعم احيانا 3

نعم غالباً جداً 4

نعم كثيراً جداً 5

For peer review only - http://bmjopen.bmj.com/site/about/guidelines.xhtml

٢- هل ادى ذلك الى انخفاض معدل تواصلك مع أصدقائك سواء تواصل اجتماعياً او من خلال التواصل الإليكتروني؟

- لا 0
- نعم لا يوجد ضغط اطلاقاً 1
- نعم قليلاً 2
- نعم احيانا 3
- نعم غالباً جداً 4
- نعم كثيراً جداً 5

تغيرات في السلوك و الروتين اليومي

يرجى الإجابة (نعم / لا / لا أُفضل الإجابة) إذا كان لجائحة كوفيد-١٩ أثرعليك من خلال الإسئلة التالية. إذا كانت اجابتك بنعم، يرجى تحديد مقدار الضغط الذي تشعر به من خلال هذا المقياس أدناه.

١- هل أدت جائحة كوفيد إلى تغير معدل أنشطتك اليومية (على سبيل المثال :معدل النوم، الأنشطة الرياضية، وقت الفراغ أو استمتاعك بالأنشطة أو الهوايات الخاص بك)؟

- لا 0
- نعم لا يوجد ضغط اطلاقاً 1
- نعم قليلاً 2
- نعم احيانا 3
- نعم غالباً جداً 4
- نعم كثيراً جداً 5

٢- هل ادى ذلك الى تأخير او تجنب طلب الرعاية الصحية عندما تحتاجها أنت أو أحد أفراد أسرتك؟

- لا 0
- نعم لا يوجد ضغط اطلاقاً 1
- نعم قليلاً 2
- نعم احيانا 3

For peer review only - http://bmjopen.bmj.com/site/about/guidelines.xhtml

○ نعم غالباً جداً 4

○ نعم كثيراً جداً 5

٣- هل ادى ذلك الى انخفاض معدل مشاركتك في الأنشطة الدينية (مثل سواء وجهاً لوجه أو عبر الإنترنت)؟

○ لا أُفضل الإجابة

○ لا 0

○ نعم لا يوجد ضغط اطلاقاً 1

○ نعم قليلاً 2

○ نعم احيانا 3

○ نعم غالباً جداً 4

○ نعم كثيراً جداً 5

٤- هل لديك اى تغيرات كبيرة اخرى في حياتك الروتينية؟

○ لا 0

○ نعم (برجاء الشرح) 1 ____________________

إذا كانت اجابتك بنعم، يرجى تحديد مقدار الضغط الذي تشعر به من خلال هذا المقياس أدناه.

○ لا يوجد ضغط اطلاقاً 1

○ قليلاً 2

○ احيانا 3

○ غالباً جداً 4

○ كثيراً جداً 5

For peer review only - http://bmjopen.bmj.com/site/about/guidelines.xhtml

معلومات فيما يخص كوفيد-١٩

١- فيما يخص كوفيد-١٩ ماهو مصدر المعلومات بالنسبة لك؟ (برجاء وضع علامة على أكثر من إختيار.)

- ☐ الأخبار
- ☐ الفيس بوك / تويتر
- ☐ المواقع الصحية المعتمدة
- ☐ كلام شَفهي
- ☐ أخرى (برجاء الشرح) ________________________________

٢- ما مدى الضغط النفسي الذي تسببته الأخبار عن كوفيد-١٩؟

- ○ نعم لا يوجد ضغط اطلاقاً 1
- ○ نعم قليلاً 2
- ○ نعم احيانا 3
- ○ نعم غالباً جداً 4
- ○ نعم كثيراً جداً 5

تغيرات منذ جائحة كوفيد-١٩

من خلال الأسئلة التالية، يرجى الإشارة إلى مقدار الضغط النفسي الذي تأثرت به. مقارنة بين الوضع الحالي و الوضع قبل جائحة كوفيد-١٩....

١- كيف تقيم مستوى ضغطك الشخصي؟

- ○ ضغط قليل جداً 1
- ○ ضغط الى حداً ما 2
- ○ بقى كما هو 3
- ○ ضغط أكثر 4
- ○ ضغط كثير جداً 5

٢- كيف تقيم مستوى الضغط العام في منزلك؟

- ○ ضغط قليل جداً 1
- ○ ضغط الى حداً ما 2

For peer review only - http://bmjopen.bmj.com/site/about/guidelines.xhtml

بقى كما هو 3

ضغط أكثر 4

ضغط كثير جداً 5

٣ـ كيف تقيم صحتك النفسية الشخصية؟

أفضل بكثير 1

أفضل 2

بقى كما هو 3

أسوء 4

أسوء بكثير 5

٤ـ كيف تُقيم مقدار الصحة النفسية لأفراد أسرتك بشكل عام؟

أفضل بكثير 1

أفضل 2

بقى كما هو 3

أسوء 4

أسوء بكثير 5

For peer review only

For peer review only - http://bmjopen.bmj.com/site/about/guidelines.xhtml

K10

الأسئلة العشر التالية سوف يتم سؤالك عن ما كنت تشعر به خلال الأربع أسابيع الماضية. يرجى قراءة الأسئلة التالية و اختيار الإجابة الأقرب التي تمثل وضعك الحالي.

	إطلاقاً 1	قليلاً 2	أحياناً 3	غالباً 4	دائماً 5
١ـ خلال الأربع أسابيع الماضية، إلى أي مدى كنت تشعرأنك مجهد بدون أي سبب واضح؟					
٢ـ خلال الأربع أسابيع الماضية، إلى أي مدى كنت تشعر بالعصبية؟					
٣ـ خلال الأربع أسابيع الماضية، إلى أي مدى كنت تشعر بالعصبية إلى حد أن لاشيء يمكنه أن يجعلك تهدئ؟					
٤ـ خلال الأربع أسابيع الماضية، إلى أي مدى كنت تشعر باليأس؟					
٥ـ خلال الأربع أسابيع الماضية، إلى أي مدى كنت تشعر بعدم الراحة أو القلق؟					
٦ـ خلال الأربع أسابيع الماضية، إلى أي مدى كنت تشعر بعدم الراحة لدرجة أنه لايمكنك الجلوس هادئاً؟					
٧ـ خلال الأربع أسابيع الماضية، إلى أي مدى كنت تشعر بالإكتئاب؟					
٨ـ خلال الأربع أسابيع الماضية، إلى أي مدى كنت تشعر إنك تبذل مجهوداً كبيراً عندما تفعل أي شيء؟					
٩ـ خلال الأربع أسابيع الماضية، إلى أي مدى كنت تشعر بالحزن لدرجة إن لاشيء يحسن من معنوياتك؟					
١٠ـ خلال الأربع أسابيع الماضية، إلى أي مدى كنت تشعر بأن حياتك ليست لها قيمة أو معنى؟					

For peer review only

PCL-5

فيما يلي قائمة بالمشاكل التى قد يعاني منها بعض الناس كأستجابة لكوفيد-١٩. الرجاء قراءة كل مشكلة بعناية ثم توضيح مدى إنزعاجك لهذه المشكلة خلال الشهر الماضي. خلال الشهر الماضي ما مدى إنزعاجك مما يلي:

	أطلاقاً 0	قليلاً 1	متوسط 2	غالباً 3	كثيراً جداً 4
١ـ ذكريات متكررة، مزعجة و غير مرغوب فيها نتيجة التجربة المؤلمة التي مررت بها؟	○	○	○	○	○
٢ـ أحلام متكررة، مزعجة نتيجة التجربة المؤلمة التي مررت بها؟	○	○	○	○	○
٣ -الشعور أو التصرف المفاجيء كما لو أن التجربة المؤلمة تحدث مرة آخرى (كأنك تعيش الحادث من جديد)؟	○	○	○	○	○
٤ـ الشعور بالإستياء الشديد عندما يذكرك شيء ما بالتجربة المؤلمة؟	○	○	○	○	○
٥ـ تعرضك لردة فعل جسدية قوية عند حدوث شيء يذكرك بالتجربة المؤلمة (مثل خفقان بالقلب، صعوبة في التنفس أو التعرق)؟	○	○	○	○	○
٦ـ تجنب الذكريات، الأفكار أو المشاعر المتعلقة بالتجربة المؤلمة؟	○	○	○	○	○
٧ـ تتجنب الأشياء الخارجية التي تذكرك بالتجربة المؤلمة (مثل الأشخاص، الأماكن، المحادثات، الأنشطة، الأشياء أو المواقف)؟	○	○	○	○	○
٨ـ تعاني من مشاكل تذكرك بأجزاء مهمة متعلقة بالتجربة المؤلمة؟	○	○	○	○	○
٩ـ أصبح لديك معتقدات سلبية قوية متعلقة بك أو عن الأشخاص الأخرين أو العالم الخارجي (مثل لديك أفكار مثل :أنا سيء، هناك شيء خاطىء فيّ، لا يمكن الوثوق بأحد أو العالم بأكمله)؟	○	○	○	○	○
١٠ـ إلقاء اللوم على نفسك أو على شخص آخر فيما يخص حدوث التجربة المؤلمة أوتبعاتها؟	○	○	○	○	○
١١ـ وجود مشاعر سلبية قوية مثل الخوف، الرعب، الغضب، الشعور بالذنب أو العار؟	○	○	○	○	○
١٢ـ فقدان الإهتمام بالأنشطة التي كنت تستمتع بها؟	○	○	○	○	○

For peer review only

For peer review only - http://bmjopen.bmj.com/site/about/guidelines.xhtml

١٣ـ الشعور بالبعد أو العزلة عن الأشخاص الأخرين؟

١٤ـ صعوبة الإحساس بالمشاعر الإيجابية (مثل عدم التمكن من الشعور بالسعادة أو مشاعر حب تجاه المقربين إليك)؟

١٥ـ سرعة الغضب أو المرور بنوبات الغضب أو التصرف بعدوانية؟

١٦ -الخوض في الكثير من المخاطر أو الأشياء التي قد تسبب لك الضرر؟

١٧ـ البقاء في حالة تأهب قصوى، إحتراس أو حذر شديدين؟

١٨ـ شعور متقلب أو الإستفزاز بسهولة؟

١٩ـ صعوبة في التركيز؟

٢٠ـ مشاكل في الرغبة في النوم أو في البقاء نائماً؟

For peer review only

For peer review only - http://bmjopen.bmj.com/site/about/guidelines.xhtml

WHO-5

يرجى وضع إشارة عند كل من الأسئلة الخمس التالية، حيث تمثل الإجابة شعورك خلال الإسبوعين الماضيين.

	دائماً 5	أكثر الأحيان 4	أكثر من نصف الوقت 3	أقل من نصف الوقت 2	بعض الوقت 1	بتاتاً 0
١- كنت سعيداً و بمزاجٍ جيد.	○	○	○	○	○	○
٢- كنت أشعُر بالهدوء و الإسترخاء.	○	○	○	○	○	○
٣- كنت أشعر بالحيوية و النشاط.	○	○	○	○	○	○
٤- كنت أستيقظ نشطاً و مرتاحاً.	○	○	○	○	○	○
٥- كانت أيامي مليئةً بأشياء محببة لنفسي.	○	○	○	○	○	○

For peer review only

PGTI

من خلال الأسئلة التالية يرجى تحديد الدرجة المناسبة لمدى التغيير الذي احدثته جائحة كوفيد-١٩ على حياتك.

	لا على الإطلاق 0	قليلاً 1	إلى حد ما 2	بشكلٍ معتدل 3	إلى حد قليل 4	كثيراً 5
١- قمت بتغيير أولوياتي عما هو مهم في الحياة.	○	○	○	○	○	○
٢- أصبح لديَّ تقديراً أكبر لقيمة حياتي.	○	○	○	○	○	○
٣- طوّرت اهتمامات جديدة.	○	○	○	○	○	○
٤- لديّ إحساس أكبر بالإعتماد على الذات.	○	○	○	○	○	○
٥- لديّ فهمًا أفضل للمسائل الروحية.	○	○	○	○	○	○
٦- أرى بوضوح أكثر أنه يمكنني الإعتماد على الأشخاص في أوقات المشاكل.	○	○	○	○	○	○
٧- أنشأت مساراً جديداً لحياتي.	○	○	○	○	○	○
٨- أصبح لديّ إحساس كبير بالقرب من الآخرين.	○	○	○	○	○	○
٩- أصبحت أكثر استعداداً للتعبير عن مشاعري.	○	○	○	○	○	○
١٠- أستطيع التعامل مع الصعوبات.	○	○	○	○	○	○
١١- أستطيع القيام بأشياء أفضل في حياتي.	○	○	○	○	○	○
١٢- أصبحت أفضل تقبلاً للطرق التي تحل بها الأمور.	○	○	○	○	○	○
١٣- يمكنني تقدير كل يوم بشكلٍ أفضل.	○	○	○	○	○	○

For peer review only

١٤- تتوفر فرص جديدة لم تكن ستتوفر بطريقة أخرى.	○	○	○	○	○	○
١٥- أشعر بمزيد من التعاطف تجاه الآخرين.	○	○	○	○	○	○
١٦- أضع مزيداً من الجهد في علاقاتي.	○	○	○	○	○	○
١٧- احاول جاهداً تغيير الأشياء التي تحتاج إلى التغيير.	○	○	○	○	○	○
١٨- إزدادت قوة إيماني.	○	○	○	○	○	○
١٩- اكتشفت أنني أقوى مما كنت أتصور.	○	○	○	○	○	○
٢٠- تعلمت قدراً كبيراً عن مدى حسن خُلق الأشخاص.	○	○	○	○	○	○
٢١- أتقبل بشكلٍ أفضل احتياجي للآخرين .	○	○	○	○	○	○

For peer review only

International Study – Indonesia (Bahasa Indonesia)

Sociodemographic Information

1. Jenis Kelamin

◯ Laki-laki

◯ Perempuan

◯ Lainnya

◯ Memilih untuk tidak menjawab

2. Umur (tahun)

____

3. Status pernikahan

◯ Belum menikah

◯ Sedang memiliki hubungan

◯ Menikah

◯ Berpisah

◯ Cerai hidup

◯ Cerai mati

4. Apakah agama yang Anda anut sekarang?

◯ Tidak beragama

◯ Islam

◯ Kristen/Katolik

◯ Hindu

◯ Buddha

For peer review only

- ○ Lain-lain (Sebutkan) ____

5. Apa latar belakang etnisitas/suku bangsa yang Anda miliki? (Sebutkan dan Anda boleh menyebutkan lebih dari satu etnis/suku bangsa)

____

6. Pendidikan tertinggi yang ditamatkan?

- ○ Pendidikan non formal (homeschooling, les privat/les Bersama, pelatihan)
- ○ SMA/SMK dan sederajat
- ○ Perguruan Tinggi D3
- ○ Perguruan Tinggi S1/D4
- ○ Perguruan Tinggi S2/S3

7. Status pekerjaan dan pendidikan yang saat ini dilakukan (Beri tanda pada pilihan Anda)

- ☐ Belajar penuh waktu
- ☐ Belajar paruh waktu
- ☐ Tidak bekerja
- ☐ Bekerja penuh waktu (karyawan/pegawai)
- ☐ Bekerja paruh waktu (karyawan/pegawai)
- ☐ Bekerja berusaha sendiri/wiraswasta penuh waktu (Full-time self employed)
- ☐ Bekerja berusaha sendiri/wiraswasta paruh waktu (Part-time self employed)
- ☐ Cuti diluar tanggungan negara (jika Anda berstatus ASN ataupun tugas belajar)
- ☐ Bekerja di rumah, tanpa menerima gaji (misal: ibu rumah tangga)
- ☐ Pensiun/Purna Tugas

For peer review only

For peer review only - http://bmjopen.bmj.com/site/about/guidelines.xhtml

☐ Lainnya (Sebutkan) ____

8. Pekerjaan (jika saat ini sedang melakukan kerja penuh waktu dan atau kerja paruh waktu)

____

9. Berapakah pendapatan dan penghasilan Anda (termasuk suami/istri, jika menikah) dalam 1 bulan (per bulan-Rp)

____

9a. Dibandingkan dengan pendapatan rata-rata nasional penduduk Indonesia tahun 2020 berdasarkan data BPS Februari, 2020 (Rp 1,6 juta per bulan), maka bagaimana Anda menilai posisi pendapatan keluarga Anda per bulan?

○ Dibawah rata-rata

○ Rata-rata

○ Diatas rata-rata

10. Apakah saat ini berada pada lingkungan yang melakukan Pemberlakukan Pembatasan Kegiatan Masyarakat (PPKM)?

○ Iya

○ Tidak

11. Apakah Anda sudah mendapatkan vaksin Covid-19?

○ Iya

○ Tidak

11a. Jika belum mendapatkan vaksin, apakah Anda bersedia untuk divaksinasi di kemudian hari?

○ Iya

○ Tidak

For peer review only - http://bmjopen.bmj.com/site/about/guidelines.xhtml

For peer review only

12. Kami menyadari bahwa sebagian orang memiliki pengalaman traumatik di masa lalu yang membuat seseorang akan lebih mudah atau sulit untuk menjalani kehidupan, menghadapi tantangan baru maupun mengatasi permasalahan baru yang muncul di kemudian hari.

Sebelum wabah Covid-19, apakah Anda pernah mengalami ataupun menyaksikan beberapa kejadian seperti di bawah ini: (Beri tanda sebanyak mungkin sesuai yang Anda alami)

☐ Bencana alam (banjir, gempa bumi, tsunami, tanah longsor dsb)

☐ Hidup di zona konflik atau peperangan ataupun daerah darurat militer

☐ Pengalaman tidak menyenangkan ketika masa kecil sebelum usia 15 tahun (diabaikan, ditelantarkan orang tua, dibuli, atau kekerasan fisik-mental/seksual)

☐ Kekerasan fisik-mental/seksual Ketika usia diatas 16 tahun

☐ Kecelakaan fisik yang serius

☐ Lainnya, sebutkan pengalaman lainnya yang menyebabkan traumatik di masa lalu
____

☐ Tidak pernah mengalami pengalaman traumatik di masa lalu

For peer review only

Covid Psychosocial Impacts Scale

Dampak terhadap Pribadi

Berikan respon Ya / Tidak terhadap pertanyaaan di bawah ini, jika hal berikut dialami oleh Anda terkait dengan wabah Covid-19. Jika jawaban anda Ya, mohon berikan indikasi penilaian tekanan (stress) yang dialami seusai dengan skala berikut.

1. Apakah Anda mempunyai masalah kesehatan yang membuat Anda rentan/beresiko terhadap Covid-19?

- ○ **Tidak 0**
- ○ **Ya** Tidak membuat stress 1
- ○ **Ya** Sedikit membuat stress 2
- ○ **Ya** Cukup membuat stress 3
- ○ **Ya** Banyak membuat stress 4
- ○ **Ya** Sangat membuat stress 5

2. Apakah Anda merasa berisiko terpapar dengan orang yang terkena Covid-19?

- ○ **Tidak 0**
- ○ **Ya** Tidak membuat stress 1
- ○ **Ya** Sedikit membuat stress 2
- ○ **Ya** Cukup membuat stress 3
- ○ **Ya** Banyak membuat stress 4
- ○ **Ya** Sangat membuat stress 5

3. Apakah Anda merasa pernah terkena Covid-19?

- ○ **Tidak 0**
- ○ **Ya** Tidak membuat stress 1
- ○ **Ya** Sedikit membuat stress 2
- ○ **Ya** Cukup membuat stress 3

For peer review only

◯ **Ya** Banyak membuat stress 4

◯ **Ya** Sangat membuat stress 5

3a. Apakah Anda pernah mendapatkan hasil positif dari tes Covid-19?

◯ **Tidak 0**

◯ **Ya** Tidak membuat stress 1

◯ **Ya** Sedikit membuat stress 2

◯ **Ya** Cukup membuat stress 3

◯ **Ya** Banyak membuat stress 4

◯ **Ya** Sangat membuat stress 5

3b. Apakah saat ini Anda merasa mengalami gejala Covid-19?

◯ **Tidak 0**

◯ **Ya** Tidak membuat stress 1

◯ **Ya** Sedikit membuat stress 2

◯ **Ya** Cukup membuat stress 3

◯ **Ya** Banyak membuat stress 4

◯ **Ya** Sangat membuat stress 5

Dampak terhadap Keluarga

Berikan respon Ya/Tidak terhadap pertanyaan di bawah ini, jika hal berikut dialami oleh anggota keluarga dekat Anda terkait dengan wabah Covid-19. Jika jawaban anda Ya, mohon berikan indikasi penilaian tekanan (stress) yang dialami sesuai dengan skala berikut.

1. Apakah **keluarga dekat Anda mempunyai masalah** kesehatan **yang membuat mereka rentan/berisiko terhadap Covid-19?**

◯ **Tidak 0**

For peer review only

For peer review only - http://bmjopen.bmj.com/site/about/guidelines.xhtml

◯ **Ya** Tidak membuat stress 1

◯ **Ya** Sedikit membuat stress 2

◯ **Ya** Cukup membuat stress 3

◯ **Ya** Banyak membuat stress 4

◯ **Ya** Sangat membuat stress 5

2. Apakah Anda merasa anggota keluarga Anda berisiko berhadapan dengan orang yang terkena Covid-19?

◯ **Tidak** 0

◯ **Ya** Tidak membuat stress 1

◯ **Ya** Sedikit membuat stress 2

◯ **Ya** Cukup membuat stress 3

◯ **Ya** Banyak membuat stress 4

◯ **Ya** Sangat membuat stress 5

3. Apakah Anda merasa ada anggota keluarga terdekat yang terkena Covid-19?

◯ **Tidak** 0

◯ **Ya** Tidak membuat stress 1

◯ **Ya** Sedikit membuat stress 2

◯ **Ya** Cukup membuat stress 3

◯ **Ya** Banyak membuat stress 4

◯ **Ya** Sangat membuat stress 5

3a. Apakah ada anggota keluarga terdekat anda yang dites hasilnya positif Covid-19?

◯ **Tidak** 0

For peer review only - http://bmjopen.bmj.com/site/about/guidelines.xhtml

- **Ya** Tidak membuat stress 1
- **Ya** Sedikit membuat stress 2
- **Ya** Cukup membuat stress 3
- **Ya** Banyak membuat stress 4
- **Ya** Sangat membuat stress 5

3b. Apakah ada anggota keluarga terdekat anda mengalami gejala Covid-19?

- **Tidak** 0
- **Ya** Tidak membuat stress 1
- **Ya** Sedikit membuat stress 2
- **Ya** Cukup membuat stress 3
- **Ya** Banyak membuat stress 4
- **Ya** Sangat membuat stress 5

3c. Apakah ada anggota keluarga terdekat anda meninggal dunia karena Covid-19?

- **Tidak** 0
- **Ya** Tidak membuat stress 1
- **Ya** Sedikit membuat stress 2
- **Ya** Cukup membuat stress 3
- **Ya** Banyak membuat stress 4
- **Ya** Sangat membuat stress 5

Apakah Anda memiliki anggota keluarga terdekat yang menetap di luar negeri yang Anda khawatirkan berisiko terkena Covid-19?

- **Tidak** 0

For peer review only

- **Ya** Tidak membuat stress 1
- **Ya** Sedikit membuat stress 2
- **Ya** Cukup membuat stress 3
- **Ya** Banyak membuat stress 4
- **Ya** Sangat membuat stress 5

5. Apakah Anda memiliki keluarga dekat di luar negeri yang tidak dapat Anda kunjungi atau mereka tidak dapat mengunjungi Anda saat ini?

- **Tidak** 0
- **Ya** Tidak membuat stress 1
- **Ya** Sedikit membuat stress 2
- **Ya** Cukup membuat stress 3
- **Ya** Banyak membuat stress 4
- **Ya** Sangat membuat stress 5

Pekerjaan

Berikan respon Ya / Tidak / Tidak berlaku terhadap pertanyaaan di bawah ini, jika hal berikut dialami oleh Anda atau anggota keluarga Anda terkait dengan wabah Covid-19. Jika jawaban anda Ya, mohon berikan indikasi penilaian tekanan (stress) yang dialami sesuai dengan skala berikut.

1. Apakah Anda atau anggota keluarga Anda termasuk pekerja di sektor penting (misalnya bekerja di bidang kesehatan, penegak hukum, keuangan, petugas yang berkaitan dengan kedaruratan atau penyedia jasa layanan penting lainnya)?

- **Tidak** 0
- **Ya** Tidak membuat stress 1
- **Ya** Sedikit membuat stress 2
- **Ya** Cukup membuat stress 3

For peer review only - http://bmjopen.bmj.com/site/about/guidelines.xhtml

- **Ya** Banyak membuat stress 4
- **Ya** Sangat membuat stress 5

2. Apakah Anda atau anggota keluarga Anda mengalami perubahan (penambahan/pengurangan) jam kerja?

- **Tidak** 0
- **Ya** Tidak membuat stress 1
- **Ya** Sedikit membuat stress 2
- **Ya** Cukup membuat stress 3
- **Ya** Banyak membuat stress 4
- **Ya** Sangat membuat stress 5

3. Apakah Anda atau anggota keluarga Anda harus berubah tempat kerjanya menjadi bekerja dari rumah?

- **Tidak** 0
- **Ya** Tidak membuat stress 1
- **Ya** Sedikit membuat stress 2
- **Ya** Cukup membuat stress 3
- **Ya** Banyak membuat stress 4
- **Ya** Sangat membuat stress 5

4. Apakah Anda atau anggota keluarga Anda terpaksa berubah jenis pekerjaannya?

- **Tidak** 0
- **Ya** Tidak membuat stress 1
- **Ya** Sedikit membuat stress 2
- **Ya** Cukup membuat stress 3

For peer review only

For peer review only - http://bmjopen.bmj.com/site/about/guidelines.xhtml

- Ya Banyak membuat stress 4
- Ya Sangat membuat stress 5

5. Apakah Anda atau anggota keluarga Anda kehilangan pekerjaan?

- **Tidak Berlaku**
- **Tidak** 0
- **Ya** Tidak membuat stress 1
- **Ya** Sedikit membuat stress 2
- **Ya** Cukup membuat stress 3
- **Ya** Banyak membuat stress 4
- **Ya** Sangat membuat stress 5

6. Apakah usaha Anda atau usaha anggota keluarga Anda mengalami kesulitan bertahan saat ini?

- **Tidak Berlaku**
- **Tidak** 0
- **Ya** Tidak membuat stress 1
- **Ya** Sedikit membuat stress 2
- **Ya** Cukup membuat stress 3
- **Ya** Banyak membuat stress 4
- **Ya** Sangat membuat stress 5

Pendapatan

Berikan respon Ya / Tidak terhadap pertanyaaan di bawah ini, jika hal berikut dialami oleh Anda, keluarga Anda atau anggota keluarga Anda terkait dengan wabah Covid-19. Jika jawaban anda Ya, mohon berikan indikasi penilaian tekanan (stress) yang dialami sesuai dengan skala berikut.

For peer review only

For peer review only - http://bmjopen.bmj.com/site/about/guidelines.xhtml

1. Apakah keluarga Anda mengalami penurunan pendapatan?

- **Tidak** 0
- **Ya** Tidak membuat stress 1
- **Ya** Sedikit membuat stress 2
- **Ya** Cukup membuat stress 3
- **Ya** Banyak membuat stress 4
- **Ya** Sangat membuat stress 5

2. Apakah keluarga Anda mengalami penurunan pendapatan yang mengakibatkan Anda kesulitan membayar tagihan atau membeli makanan/bahan pokok makanan?

- **Tidak** 0
- **Ya** Tidak membuat stress 1
- **Ya** Sedikit membuat stress 2
- **Ya** Cukup membuat stress 3
- **Ya** Banyak membuat stress 4
- **Ya** Sangat membuat stress 5

Hubungan Keluarga

Berikan respon Ya/Tidak terhadap pertanyaan di bawah ini, jika hal berikut dialami oleh Anda terkait dengan wabah Covid-19. Jika jawaban anda Ya, mohon berikan indikasi penilaian tekanan (stress) yang dialami sesuai dengan skala berikut. Akibat dari Covid-19……...

1. Meningkatkan pertengkaran/memperburuk komunikasi/hubungan menjadi renggang-jauh dalam keluarga Anda?

- **Tidak** 0
- **Ya** Tidak membuat stress 1
- **Ya** Sedikit membuat stress 2

For peer review only - http://bmjopen.bmj.com/site/about/guidelines.xhtml

Ya Cukup membuat stress 3

Ya Banyak membuat stress 4

Ya Sangat membuat stress 5

2. Hubungan Anda dengan teman/kerabat (baik secara fisik maupun daring/online) menurun?

Tidak 0

Ya Tidak membuat stress 1

Ya Sedikit membuat stress 2

Ya Cukup membuat stress 3

Ya Banyak membuat stress 4

Ya Sangat membuat stress 5

Perubahan dalam kegiatan dan perilaku sehari-hari

Berikan respon Ya / Tidak / Tidak Berlaku terhadap pertanyaan di bawah ini, jika hal berikut dialami oleh Anda terkait dengan wabah Covid-19. Jika jawaban anda Ya, mohon berikan indikasi penilaian tekanan (stress) yang dialami sesuai dengan skala berikut.

1. Apakah wabah Covid-19 telah merubah kegiatan sehari-hari Anda (Sebagai contoh, tidur, olah raga, waktu bebas, aktifitas yang menyenangkan atau hobi)?

Tidak 0

Ya Tidak membuat stress 1

Ya Sedikit membuat stress 2

Ya Cukup membuat stress 3

Ya Banyak membuat stress 4

Ya Sangat membuat stress 5

For peer review only - http://bmjopen.bmj.com/site/about/guidelines.xhtml

2. Membuat Anda menunda/menghindar untuk mencari bantuan kesehatan saat Anda atau keluarga Anda membutuhkannya?

- **Tidak** 0
- **Ya** Tidak membuat stress 1
- **Ya** Sedikit membuat stress 2
- **Ya** Cukup membuat stress 3
- **Ya** Banyak membuat stress 4
- **Ya** Sangat membuat stress 5

3. Menurunkan keterlibatan Anda dalam aktifitas keagamaan (baik tatap muka ataupun online)?

- **Tidak Berlaku**
- **Tidak** 0
- **Ya** Tidak membuat stress 1
- **Ya** Sedikit membuat stress 2
- **Ya** Cukup membuat stress 3
- **Ya** Banyak membuat stress 4
- **Ya** Sangat membuat stress 5

4. Apakah ada perubahan besar terhadap keseharian Anda?

- **Tidak** 0
- **Jika ada, mohon jelaskan** 1 ____

Jika jawaban anda Ya, mohon berikan indikasi penilaian tekanan (stress) yang dialami sesuai dengan skala berikut.

- Tidak membuat stress 1
- Sedikit membuat stress 2

For peer review only

For peer review only - http://bmjopen.bmj.com/site/about/guidelines.xhtml

◯ Cukup membuat stress 3

◯ Banyak membuat stress 4

◯ Sangat membuat stress 5

Paparan terhadap informasi mengenai Covid-19

1. Terkait dengan Covid-19, dari manakah Anda mendapatkan informasinya? (Mohon dipilih sebanyak mungkin sesuai yang Anda alami)

☐ Berita

☐ Facebook/Twitter

☐ Situs kesehatan

☐ Dari mulut ke mulut

☐ Lain-lain (Mohon dijelaskan) ____

2. Seberapa banyak tekanan (stress) yang Anda alami karena pemberitaan mengenai Covid-19?

◯ Tidak membuat stress 1

◯ Sedikit membuat stress 2

◯ Cukup membuat stress 3

◯ Banyak membuat stress 4

◯ Sangat membuat stress 5

Perubahan sejak Covid-19

Dari pertanyaan di bawah ini, pilihlah yang Anda rasakan dengan melingkari nomor yang sesuai dengan situasi Anda. Mohon bandingkan saat ini dengan sebelum wabah Covid-19...

1. Bagaimana Anda menilai tingkat tekanan/stres yang dialami?

◯ Sangat tidak merasakan tekanan/stress 1

◯ Sedikit tidak merasakan tekanan/stress 2

For peer review only - http://bmjopen.bmj.com/site/about/guidelines.xhtml

- Tidak ada perubahan 3
- Lebih merasakan tekanan/stress 4
- Sangat merasakan tekanan/stress 5

2. Bagaimana Anda menilai tingkat tekanan (stress) yang dirasakan oleh anggota keluarga Anda secara keseluruhan?

- Sangat tidak merasakan tekanan/stress 1
- Sedikit tidak merasakan tekanan/stress 2
- Tidak ada perubahan 3
- Lebih merasakan tekanan/stress 4
- Sangat merasakan tekanan/stress 5

3. Bagaimana Anda menilai kesejahteraan psikologis Anda?

- Sangat baik 1
- Lebih baik 2
- Tidak ada perubahan 3
- Lebih buruk 4
- Sangat buruk 5

4. Bagaimana Anda menilai kesejahteraan psikologis anggota keluarga Anda secara keseluruhan?

- Sangat baik 1
- Lebih baik 2
- Tidak ada perubahan 3
- Lebih buruk 4
- Sangat buruk 5

For peer review only

K-10

Sepuluh pertanyaan berikut ini menanyakan perasaan Anda dalam 4 minggu terakhir. Untuk setiap pertanyaan, mohon lingkari pilihan yang menjelaskan seringnya Anda merasakan hal tersebut.

	Tidak pernah 1	Jarang 2	Kadang-kadang 3	Hampir selalu 4	Setiap saat 5
1. Dalam kurun waktu empat minggu terakhir, seberapa sering Anda merasa lelah tanpa sebab yang jelas?	○	○	○	○	○
2. Dalam empat minggu terakhir, seberapa sering Anda merasa cemas?	○	○	○	○	○
3. Dalam empat minggu terakhir seberapa sering Anda merasa cemas dan tidak bisa menenangkan diri?	○	○	○	○	○
4. Dalam empat minggu terakhir, seberapa sering Anda merasa tidak punya harapan?	○	○	○	○	○
5. Dalam empat minggu terakhir, seberapa sering Anda merasa gelisah atau resah?	○	○	○	○	○
6. Dalam empat minggu terakhir, seberapa sering Anda merasa gelisah yang menyebabkan Anda tidak dapat duduk diam?	○	○	○	○	○
7. Dalam empat minggu terakhir, seberapa sering Anda merasa tertekan?	○	○	○	○	○
8. Dalam empat minggu terakhir, seberapa sering Anda merasa berat sekali untuk melakukan sesuatu?	○	○	○	○	○
9. Dalam empat minggu terakhir, seberapa sering Anda merasa sedih sehingga tidak ada apapun yang dapat menghibur Anda?	○	○	○	○	○
10. Dalam empat minggu terakhir, seberapa sering Anda merasa tidak berharga sama sekali?	○	○	○	○	○

For peer review only

For peer review only - http://bmjopen.bmj.com/site/about/guidelines.xhtml

PCL-5

Di bawah ini adalah masalah-masalah yang seringkali dialami seseorang dalam menghadapi situasi Covid-19. Mohon dibaca setiap masalah dengan teliti dan kemudian lingkari nomor untuk mengindikasikan seberapa sering Anda terganggu oleh masalah tersebut dalam kurun waktu satu bulan terakhir. Dalam satu Bulan terakhir, seberapa sering Anda terganggu dengan hal berikut:

	**Tidak sama sekali** 0	**Sedikit tergganggu** 1	**Kadang-kadang tergganggu** 2	**Hampir tergganggu** 3	**Sangat tergganggu** 4
1. Terulangnya kenangan yang tidak diinginkan dan membuat tertekan/stres?	○	○	○	○	○
2. Terulangnya mimpi yang menganggu saat mengalami tekanan/stres?	○	○	○	○	○
3. Tiba-tiba merasakan atau bertingkah seakan-akan pengalaman saat tekanan/stres terjadi terulang kembali (seakan-akan Anda kembali ke situasi tersebut?	○	○	○	○	○
4. Merasa marah saat terjadi sesuatu yang mengingatkan Anda pada pengalaman saat tekanan/stres terjadi?	○	○	○	○	○
5. Mengalami reaksi fisik yang kuat saat sesuatu terjadi dimana mengingatkan Anda pada pengalaman saat tekanan/stres terjadi (sebagai contoh, jantung berdebar kencang, mengalami kesusahan bernafas, berkeringat)?	○	○	○	○	○
6. Menghindari kenangan, pikiran ataupun perasaan yang terkait dengan pengalaman saat tekanan/stres terjadi?	○	○	○	○	○
7. Menghindari hal-hal dari pihak luar yang mengingatkan Anda kepada pengalaman saat tekanan/stres terjadi (sebagai contoh, orang, tempat, percakapan, aktifitas, benda atau situasi)?	○	○	○	○	○
8. Kesulitan dalam mengingat hal penting dari pengalaman saat tekanan/stres terjadi?	○	○	○	○	○

For peer review only

9. Mengalami prasangka negatif yang kuat terhadap diri sendiri, orang lain, atau dunia (sebagai contoh, memiliki pikiran seperti: saya jahat, ada hal yang salah di dalam diri saya, tidak ada yang bisa dipercaya, dunia ini sangat kejam)?	○	○	○	○	○
10. Menyalahkan diri sendiri atau orang lain terhadap pengalaman saat tekanan/stres terjadi ataupun terhadap hal yang terjadi setelahnya?	○	○	○	○	○
11. Memilik perasaan negatif yang kuat seperti ketakutan, perasaan ngeri, marah, merasa bersalah ataupun malu?	○	○	○	○	○
12. Tidak tertarik lagi melakukan kegiatan yang biasanya Anda sukai?	○	○	○	○	○
13. Merasa jauh dan tidak memiliki hubungan dengan orang lain?	○	○	○	○	○
14. Kesulitan untuk memiliki perasaan positif (sebagai contoh, tidak dapat merasakan kebahagian atau mencintai orang yang dekat dengan Anda)?	○	○	○	○	○
15. Bertingkah laku yang mengganggu, marah tanpa kendali atau bersikap agresif?	○	○	○	○	○
16. Melakukan hal yang berisiko tinggi ataupun melakukan hal yang dapat membahayakan diri Anda?	○	○	○	○	○
17. Bersikap “sangat waspada” atau berhati-hati atau pun berjaga-jaga?	○	○	○	○	○
18. Merasa gelisah dan mudah terkejut?	○	○	○	○	○
19. Kesulitan dalam berkonsentrasi?	○	○	○	○	○
20. Susah tertidur atau tidur lebih lama?	○	○	○	○	○

For peer review only

For peer review only - http://bmjopen.bmj.com/site/about/guidelines.xhtml

WHO-5

Mohon berikan indikasi untuk ke lima pernyataan di bawah ini yang sesuai dengan yang Anda rasakan selama kurun waktu 2 minggu terakhir. Dalam kurun waktu 2 minggu terakhir

	**Setiap waktu 5**	**Kebanyakan waktu 4**	**Sebagian waktu 3**	**Hampir sebagian waktu 2**	**Sesekali 1**	**Tidak sama sekali 0**
1. Saya merasa gembira dan bahagia	○	○	○	○	○	○
2. Saya merasa tenang dan rileks	○	○	○	○	○	○
3. Saya merasa aktif dan semangat	○	○	○	○	○	○
4. Saya terbangun dengan perasaan segar dan cukup istirahat	○	○	○	○	○	○
5. Kegiatan sehari-hari saya penuh dengan hal-hal yang saya sukai	○	○	○	○	○	○

For peer review only

PTGI

Dari pernyataan di bawah ini, mohon indikasikan penilaian terjadinya perubahan dalam hidup Anda sebagai akibat dari Covid-19 dengan menggunakan skala di bawah ini.

	Tidak sama sekali 0	Sedikit 1	Agak 2	Kadang-kadang 3	Hampir 4	Sangat 5
1. Saya merubah prioritas mengenai apa yang penting dalam hidup.	○	○	○	○	○	○
2. Saya memiliki apresiasi besar terhadap nilai hidup.	○	○	○	○	○	○
3. Saya mengembangkan minat baru.	○	○	○	○	○	○
4. Saya memiliki perasaan besar terhadap kepercayaan diri.	○	○	○	○	○	○
5. Saya memiliki pemahaman yang baik terhadap masalah spiritual.	○	○	○	○	○	○
6. Saya melihat bahwa saya bisa meminta bantuan kepada orang lain di saat kesulitan.	○	○	○	○	○	○
7. Saya menempuh jalan baru bagi hidup saya.	○	○	○	○	○	○
8. Saya memiliki rasa kedekatan dengan orang lain.	○	○	○	○	○	○
9. Saya lebih bisa mengekspresikan emosi.	○	○	○	○	○	○
10. Saya tahu jika saya bisa mengatasi kesulitan.	○	○	○	○	○	○
11. Saya bisa melakukan hal-hal baik dalam hidup saya.	○	○	○	○	○	○
12. Saya semakin bisa menerima hal-hal yang terjadi.	○	○	○	○	○	○
13. Saya bisa lebih menghargai setiap hari yang saya lalui.	○	○	○	○	○	○

For peer review only

14. Kesempatan baru tersedia dibandingkan sebelumnya.	○	○	○	○	○	○
15. Saya lebih memiliki rasa kasih sayang kepada sesama.	○	○	○	○	○	○
16. Saya lebih memberikan upaya dalam hubungan saya dengan orang lain	○	○	○	○	○	○
17. Saya lebih bisa mengusahakan untuk merubah hal yang memang bisa diubah.	○	○	○	○	○	○
18. Saya memiliki keyakinan yang lebih kuat terhadap agama.	○	○	○	○	○	○
19. Saya menyadari bahwa saya lebih kuat daripada yang saya kira.	○	○	○	○	○	○
20. Saya menyadari bahwa ternyata orang lain itu baik.	○	○	○	○	○	○
21. Saya lebih bisa menerima bahwa saya membutuhkan orang lain.	○	○	○	○	○	○

For peer review only

For peer review only - http://bmjopen.bmj.com/site/about/guidelines.xhtml

International Study – Malaysia (Malay)

Sociodemographic Information

1. Jantina

- Lelaki
- Perempuan
- Lain-lain
- Lebih suka tidak diberitahu

2. Umur (dalam tahun)

____

3. Status perkahwinan

- Bujang
- Mempunya hubungan
- Berkahwin
- Tidak tinggal bersama
- Bercerai
- Duda/Janda

4. Apakah agama anda?

- Tiada agama
- Islam
- Kristian
- Hindu

For peer review only

For peer review only - http://bmjopen.bmj.com/site/about/guidelines.xhtml

○ Buddha

○ Lain-lain (Sila nyatakan) ____

5. Apakah bangsa anda? (Anda boleh menyebut lebih daripada satu)

____

6. Apakah Bahasa yang paling fasih digunakan dalam kehidupan seharian?

____

Berikut merupakan dua soalan berkenaan kebolehan anda bertutur di dalam Bahasa Inggeris. 6a. Saya faham Bahasa Inggeris dengan baik

○ Sangat tidak setuju 1

○ Tidak setuju 2

○ Natural 3

○ Setuju 4

○ Sangat Setuju 5

6b. Saya boleh bertutur Bahasa Inggeris dengan baik

○ Tidak langsung 1

○ Sedikit 2

○ Lebih kurang 3

○ Agak banyak 4

○ Sangat banyak 5

7. Apakah pendidikan tertinggi anda?

○ Tiada Pendidikan formal

For peer review only - http://bmjopen.bmj.com/site/about/guidelines.xhtml

- ○ Matrikulasi
- ○ Diploma / Sijil
- ○ Sarjana Muda
- ○ Sarjana / PhD

8. Status belajar/bekerja (tandakan yang berkenaan)

- ☐ Pelajar sepenuh masa
- ☐ Pelajar separuh masa
- ☐ Tidak bekerja/Sedang mencari pekerjaan
- ☐ Bekerja sepenuh masa
- ☐ Bekerja separuh masa
- ☐ Bekerja Sendiri sepenuh masa
- ☐ Bekerja Sendiri separuh masa
- ☐ Cuti bergaji
- ☐ Tinggal bersama ibubapa (tidak bergaji)
- ☐ Bersara
- ☐ Lain-lain (Sila nyatakan) ____

9. Pekerjaan

____

10. Pendapatan bulanan isirumah (Ringgit Malaysia)

____

For peer review only

For peer review only - http://bmjopen.bmj.com/site/about/guidelines.xhtml

10a. Apakah kadar pendapatan bulanan isirumah, jika dibandingkan dengan purata pendapatan penduduk Malaysia?

- ○ Bawah purata
- ○ Sama dengan purata
- ○ Atas purata

11. Adakah anda sekarang berada di dalam perintah kawalan pergerakan/arahan duduk di rumah?

- ○ Ya
- ○ Tidak

12. Adakahand telah menerima suntikan vaksin?

- ○ Ya
- ○ Tidak

12a. Jika belum menerima suntikan vaksin, adakah anda akan mendapatkan vaksin COVID-19 di masa akan datang?

- ○ Ya
- ○ Tidak

13. Kita sedar bahawa sesetengah orang telah didedahkan dengan insiden trauma pada masa lampau yang mana telah memudahkan atau menyusahkan untuk menangani masalah. Sebelum pandemic COVID-19, adakah anda pernah menyaksikan atau mengalami mana-mana perkara berikut: (Sila tanda sebanyak mana yang berkaitan)

- ☐ Bencana alam (seperti banjir, gempa bumi dan lain-lain)
- ☐ Tinggal di dalam zon peperangan atau telah terdedah kepada konflik tetera
- ☐ Kesukaran kanak-kanak sebelum usia 16 tahun (seperti pengabaian, buli, serangan fizikal atau seksual)

For peer review only - http://bmjopen.bmj.com/site/about/guidelines.xhtml

For peer review only

- Serangan fizikal atau seksual selepas usia 16 tahun
- Kemalangan fizikal yang serius
- Lain-lain - sila nyatakan (pilihan) ____
- Tiada satu pun di atas

For peer review only

For peer review only - http://bmjopen.bmj.com/site/about/guidelines.xhtml

Covid Psychosocail Impacts Scale

Kesan keatas diri sendiri

Untuk kenyataan di bawah, sila jawab Ya/Tidak jika kesan daripada Covid-19, berlaku kepada diri anda sendiri. Jika jawapan anda adalah Ya, sila nyatakan tahap tekanan yang dialami, berdasarkan skala dibawah.

1. Adakah anda mengalami masalah kesihatan yang boleh menyebabkan risiko anda untuk dijangkiti Covid-19 meningkat?

- **Tidak** 0
- **Ya** Tiada tekanan langsung 1
- **Ya** Tekanan tahap minima 2
- **Ya** Tekanan tahap sederhana 3
- **Ya** Tekanan tahap tinggi 4
- **Ya** Tekanan yang sangat tinggi 5

2. Adakah anda pernah merasakan bahaya risiko terdedah kepada seseorang yang menghidap Covid-19?

- **Tidak** 0
- **Ya** Tiada tekanan langsung 1
- **Ya** Tekanan tahap minima 2
- **Ya** Tekanan tahap sederhana 3
- **Ya** Tekanan tahap tinggi 4
- **Ya** Tekanan yang sangat tinggi 5

3. Adakah anda fikir bahawa anda telah dijangkiti COVID-19?

- **Tidak** 0

For peer review only

For peer review only - http://bmjopen.bmj.com/site/about/guidelines.xhtml

- ◯ **Ya** Tiada tekanan langsung 1
- ◯ **Ya** Tekanan tahap minima 2
- ◯ **Ya** Tekanan tahap sederhana 3
- ◯ **Ya** Tekanan tahap tinggi 4
- ◯ **Ya** Tekanan yang sangat tinggi 5

3a. Adakah anda pernah mendapat keputusan positif bagi ujian COVID-19?

- ◯ **Tidak** 0
- ◯ **Ya** Tiada tekanan langsung 1
- ◯ **Ya** Tekanan tahap minima 2
- ◯ **Ya** Tekanan tahap sederhana 3
- ◯ **Ya** Tekanan tahap tinggi 4
- ◯ **Ya** Tekanan yang sangat tinggi 5

3b. Adakah anda mempunyai simptom-simptom COVID-19?

- ◯ **Tidak** 0
- ◯ **Ya** Tiada tekanan langsung 1
- ◯ **Ya** Tekanan tahap minima 2
- ◯ **Ya** Tekanan tahap sederhana 3
- ◯ **Ya** Tekanan tahap tinggi 4
- ◯ **Ya** Tekanan yang sangat tinggi 5

For peer review only

Kesan kepada keluarga

Untuk kenyataan di bawah, sila jawab Ya/Tidak jika kesan daripada Covid-19, berlaku kepada ahli keluarga terdekat anda. Jika jawapan anda adalah Ya, sila nyatakan tahap tekanan yang dialami, berdasarkan skala dibawah.

1. Adakah ahli keluarga terdekat anda mengalami masalah kesihatan yang boleh menyebabkan risiko mereka untuk dijangkiti Covid-19 meningkat?

- ○ **Tidak** 0
- ○ **Ya** Tiada tekanan langsung 1
- ○ **Ya** Tekanan tahap minima 2
- ○ **Ya** Tekanan tahap sederhana 3
- ○ **Ya** Tekanan tahap tinggi 4
- ○ **Ya** Tekanan yang sangat tinggi 5

2. Adakah anda merasakan bahawa setiap ahli keluarga terdekat anda berisiko untuk terdedah kepada seseorang yang menghidap Covid-19?

- ○ **Tidak** 0
- ○ **Ya** Tiada tekanan langsung 1
- ○ **Ya** Tekanan tahap minima 2
- ○ **Ya** Tekanan tahap sederhana 3
- ○ **Ya** Tekanan tahap tinggi 4
- ○ **Ya** Tekanan yang sangat tinggi 5

3. Adakah ahli keluarga terdekat anda pernah menjalani Covid-19?

- ○ **Tidak** 0
- ○ **Ya** Tiada tekanan langsung 1

For peer review only - http://bmjopen.bmj.com/site/about/guidelines.xhtml

- **Ya** Tekanan tahap minima 2
- **Ya** Tekanan tahap sederhana 3
- **Ya** Tekanan tahap tinggi 4
- **Ya** Tekanan yang sangat tinggi 5

3a. Adakah keputusan ahli keluarga terdekat anda positif Covid-19?

- **Tidak** 0
- **Ya** Tiada tekanan langsung 1
- **Ya** Tekanan tahap minima 2
- **Ya** Tekanan tahap sederhana 3
- **Ya** Tekanan tahap tinggi 4
- **Ya** Tekanan yang sangat tinggi 5

3b. Adakah ahli keluarga terdekat anda mempunyai simptom-simptom Covid-19?

- **Tidak** 0
- **Ya** Tiada tekanan langsung 1
- **Ya** Tekanan tahap minima 2
- **Ya** Tekanan tahap sederhana 3
- **Ya** Tekanan tahap tinggi 4
- **Ya** Tekanan yang sangat tinggi 5

3c. Adakah ahli keluarga terdekat anda meninggal dunia disebabkan oleh Covid-19?

- **Tidak** 0

For peer review only

- Ya Tiada tekanan langsung 1
- Ya Tekanan tahap minima 2
- Ya Tekanan tahap sederhana 3
- Ya Tekanan tahap tinggi 4
- Ya Tekanan yang sangat tinggi 5

4. Adakah anda mempunyai ahli keluarga terdekat di luar negara yang dikhuatiri berisiko untuk dijangkiti Covid-19?

- **Tidak** 0
- **Ya** Tiada tekanan langsung 1
- **Ya** Tekanan tahap minima 2
- **Ya** Tekanan tahap sederhana 3
- **Ya** Tekanan tahap tinggi 4
- **Ya** Tekanan yang sangat tinggi 5

5. Adakah anda mempunyai ahli di luar negara yang tidak dapat anda lawati atau tidak dapat melawat anda?

- **Tidak** 0
- **Ya** Tiada tekanan langsung 1
- **Ya** Tekanan tahap minima 2
- **Ya** Tekanan tahap sederhana 3
- **Ya** Tekanan tahap tinggi 4
- **Ya** Tekanan yang sangat tinggi 5

For peer review only - http://bmjopen.bmj.com/site/about/guidelines.xhtml

Pekerjaan

Untuk kenyataan di bawah, sila jawab Ya / Tidak / Tidak berkenaan jika kesan daripada Covid-19, berlaku kepada anda atau sesiapa sahaja di rumah anda. Jika jawapan anda adalah Ya, sila nyatakan tahap tekanan yang dialami, berdasarkan skala dibawah.

1. Adakah anda atau sesiapa sahaja di rumah anda dianggap sebagai pekerja perkhidmatan perlu (Contohnya, bidang perubatan dan penjagaan kesihatan, penguatkuasaan undang-undang, perkhidmatan kecemasan, pembekal barang dan perkhidmatan penting)?

- ○ **Tidak** 0
- ○ **Ya** Tiada tekanan langsung 1
- ○ **Ya** Tekanan tahap minima 2
- ○ **Ya** Tekanan tahap sederhana 3
- ○ **Ya** Tekanan tahap tinggi 4
- ○ **Ya** Tekanan yang sangat tinggi 5

2. Adakah anda atau sesiapa sahaja di rumah anda mengalami perubahan yang besar pada waktu bekerja?

- ○ **Tidak** 0
- ○ **Ya** Tiada tekanan langsung 1
- ○ **Ya** Tekanan tahap minima 2
- ○ **Ya** Tekanan tahap sederhana 3
- ○ **Ya** Tekanan tahap tinggi 4
- ○ **Ya** Tekanan yang sangat tinggi 5

3. Adakah anda atau sesiapa sahaja di rumah anda perlu bertukar tempat kerja dan bekerja dari rumah?

- ○ **Tidak** 0

For peer review only - http://bmjopen.bmj.com/site/about/guidelines.xhtml

- **Ya** Tiada tekanan langsung 1
- **Ya** Tekanan tahap minima 2
- **Ya** Tekanan tahap sederhana 3
- **Ya** Tekanan tahap tinggi 4
- **Ya** Tekanan yang sangat tinggi 5

4. Adakah anda atau sesiapa sahaja di rumah anda perlu bertukar jenis pekerjaan?

- **Tidak** 0
- **Ya** Tiada tekanan langsung 1
- **Ya** Tekanan tahap minima 2
- **Ya** Tekanan tahap sederhana 3
- **Ya** Tekanan tahap tinggi 4
- **Ya** Tekanan yang sangat tinggi 5

5. Adakah anda atau ahli rumahtangga anda telah hilang punca pendapatan?

- **Tidak berkenaan**
- **Tidak** 0
- **Ya** Tiada tekanan langsung 1
- **Ya** Tekanan tahap minima 2
- **Ya** Tekanan tahap sederhana 3
- **Ya** Tekanan tahap tinggi 4
- **Ya** Tekanan yang sangat tinggi 5

For peer review only

6. Adakah perniagaan atau sumber pencarian anda atau ahli rumahtangga anda terjejas dan berkemungkinan untuk tidak beroperasi lagi?

- **Tidak berkenaan**
- **Tidak** 0
- **Ya** Tiada tekanan langsung 1
- **Ya** Tekanan tahap minima 2
- **Ya** Tekanan tahap sederhana 3
- **Ya** Tekanan tahap tinggi 4
- **Ya** Tekanan yang sangat tinggi 5

For peer review only

Pendapatan

Untuk kenyataan di bawah, sila jawab Ya/Tidak jika kesan daripada Covid-19 ini telah berlaku kepada ahli rumahtangga anda. Jika jawapan anda adalah Ya, sila nyatakan tahap tekanan yang dialami, berdasarkan skala dibawah.

1. Adakah isu kewangan rumahtangga anda merosot dengan mendadak?

- **Tidak** 0
- **Ya** Tiada tekanan langsung 1
- **Ya** Tekanan tahap minima 2
- **Ya** Tekanan tahap sederhana 3
- **Ya** Tekanan tahap tinggi 4
- **Ya** Tekanan yang sangat tinggi 5

2. Adakah rumahtagga anda menghadapi masalah besar untuk membeli barangan keperluan rumah atau membayar bil-bil semasa?

For peer review only - http://bmjopen.bmj.com/site/about/guidelines.xhtml

- ◯ **Tidak** 0
- ◯ **Ya** Tiada tekanan langsung 1
- ◯ **Ya** Tekanan tahap minima 2
- ◯ **Ya** Tekanan tahap sederhana 3
- ◯ **Ya** Tekanan tahap tinggi 4
- ◯ **Ya** Tekanan yang sangat tinggi 5

Perhubungan (bersama insan lain)

Untuk kenyataan di bawah, sila jawab Ya/Tidak jika kesan daripada Covid-19, berlaku kepada diri anda sendiri. Jika jawapan anda adalah Ya, sila nyatakan tahap tekanan yang dialami, berdasarkan skala dibawah. Sebagai tindak balas kepada Covid-19....

1. Adakah ia telah mendatangkan kesusahan dan tekanan kepada keluarga anda?

- ◯ **Tidak** 0
- ◯ **Ya** Tiada tekanan langsung 1
- ◯ **Ya** Tekanan tahap minima 2
- ◯ **Ya** Tekanan tahap sederhana 3
- ◯ **Ya** Tekanan tahap tinggi 4
- ◯ **Ya** Tekanan yang sangat tinggi 5

2. Adakah ia telah mengurangkan perhubungan antara rakan-rakan anda (sama ada secara fizikal ataupun dalam talian)?

- ◯ **Tidak** 0
- ◯ **Ya** Tiada tekanan langsung 1
- ◯ **Ya** Tekanan tahap minima 2

For peer review only - http://bmjopen.bmj.com/site/about/guidelines.xhtml

- **Ya** Tekanan tahap sederhana 3
- **Ya** Tekanan tahap tinggi 4
- **Ya** Tekanan yang sangat tinggi 5

Perubahan kepada Rutin dan Tingkah Laku Harian

Untuk kenyataan di bawah, sila jawab Ya/Tidak/Tidak berkenaan jika kesan daripada Covid-19, berlaku kepada diri anda sendiri. Jika jawapan anda adalah Ya, sila nyatakan tahap tekanan yang dialami, berdasarkan skala dibawah. Sebagai tindak balas kepada Covid-19....

1. Adakah wabak Covid ini telah mencetus perubahan kepada rutin harian anda (Contohnya, jadual tidur, senaman, waktu rehat, waktu riadah, atau hobi)?

- **Tidak** 0
- **Ya** Tiada tekanan langsung 1
- **Ya** Tekanan tahap minima 2
- **Ya** Tekanan tahap sederhana 3
- **Ya** Tekanan tahap tinggi 4
- **Ya** Tekanan yang sangat tinggi 5

2. Adakah ia telah membuatkan anda menangguhkan atau mengelakkan mencari rawatan perubatan sekiranya anda atau ahli keluarga anda memerlukannya?

- **Tidak** 0
- **Ya** Tiada tekanan langsung 1
- **Ya** Tekanan tahap minima 2
- **Ya** Tekanan tahap sederhana 3
- **Ya** Tekanan tahap tinggi 4

For peer review only - http://bmjopen.bmj.com/site/about/guidelines.xhtml

○ **Ya** Tekanan yang sangat tinggi 5

3. Adakah ia telah mengurangkan penglibatan anda dalam aktiviti keagamaan (sama ada secara peribadi atau dalam talian)?

○ **Tidak berknaan**

○ **Tidak** 0

○ **Ya** Tiada tekanan langsung 1

○ **Ya** Tekanan tahap minima 2

○ **Ya** Tekanan tahap sederhana 3

○ **Ya** Tekanan tahap tinggi 4

○ **Ya** Tekanan yang sangat tinggi 5

4. Adakah ia telah menyebabkan sebarang perubahan besar kepada rutin harian anda?

○ **Tidak** 0

○ **Ya (sila jelaskan)** 1 ____

Jika jawapan anda adalah Ya, sila nyatakan tahap tekanan yang dialami, berdasarkan skala dibawah.

○ Tiada tekanan langsung 1

○ Tekanan tahap minima 2

○ Tekanan tahap sederhana 3

○ Tekanan tahap tinggi 4

○ Tekanan yang sangat tinggi 5

For peer review only - http://bmjopen.bmj.com/site/about/guidelines.xhtml

Pendedahan kepada maklumat tentang Covid-19

1. Apakah sumber maklumat anda berkenaan dengan Covid-19? (Tandakan seberapa banyak yang mungkin)

- ☐ Berita
- ☐ Facebook atau Twitter
- ☐ Laman web kesihatan
- ☐ daripada orang lain
- ☐ Lain-lain (sila jelaskan) ____

2. Sebanyak manakah anda berasa tertekan membaca berita tentang Covid-19?

- ○ Tiada tekanan langsung 1
- ○ Tekanan tahap minima 2
- ○ Tekanan tahap sederhana 3
- ○ Tekanan tahap tinggi 4
- ○ Tekanan yang sangat tinggi 5

Perubahan sejak Covid-19

Untuk soalan-soalan berikutnya, sila nyatakan berdasarkan skala dibawah. Bandingkan keadaan sekarang dengan keadaan semasa sebelum wabak Covid-19 berlaku....

1. Bagaimanakah anda menilai tahap tekanan peribadi anda?

- ○ Sangat tidak tertekan 1
- ○ Kurang tertekan 2
- ○ Lebih kurang sama 3

For peer review only - http://bmjopen.bmj.com/site/about/guidelines.xhtml

○ Lebih tertekan 4

○ Sangat tertekan 5

2. Bagaimanakah anda menilai tahap tekanan ahli rumahtangga anda?

○ Sangat tidak tertekan 1

○ Kurang tertekan 2

○ Lebih kurang sama 3

○ Lebih tertekan 4

○ Sangat tertekan 5

3. Bagaimanakah anda menilai tahap kesihatan psikologi anda?

○ Sangat baik 1

○ Baik 2

○ Lebih kurang sama 3

○ Merosot 4

○ Sangat merosot 5

4. Bagaimanakah anda menilai tahap kesihatan psikologi ahli rumahtangga anda?

○ Sangat baik 1

○ Baik 2

○ Lebih kurang sama 3

○ Merosot 4

○ Sangat merosot 5

For peer review only - http://bmjopen.bmj.com/site/about/guidelines.xhtml

K-10

Sepuluh soalan berikut bertanyakan tentang perasaan yang anda rasai sejak empat minggu yang lepas. Untuk setiap soalan, sila bulatkan pilihan yang menunjukkan sebanyak mana anda merasai perasaan tersebut.

	Tidak langsung 1	Kadang-kadang 2	Sedikit 3	Selalu 4	Sepanjang masa 5
1. Dalam tempoh empat minggu yang lepas, berapa kerap anda berasa sangat penat tanpa sebarang sebab yang munasabah?	○	○	○	○	○
2. Dalam tempoh empat minggu yang lepas, berapa kerap anda berasa gugup/gementar?	○	○	○	○	○
3. Dalam tempoh empat minggu yang lepas, berapa kerap anda berasa sangat gugup/gementar sehinggga tiada apa yang boleh menenangkan anda?	○	○	○	○	○
4. Dalam tempoh empat minggu yang lepas, berapa kerap anda berasa putus asa?	○	○	○	○	○
5. Dalam tempoh empat minggu yang lepas, berapa kerap anda berasa gelisah?	○	○	○	○	○
6. Dalam tempoh empat minggu yang lepas, berapa kerap anda berasa sangat gelisah sehingga tidak boleh duduk diam?	○	○	○	○	○
7. Dalam tempoh empat minggu yang lepas, berapa kerap anda mengalami perasaan murung?	○	○	○	○	○
8. Dalam tempoh empat minggu yang lepas, berapa kerap anda berasa seperti segala yang dilakukan memerlukan usaha yang banyak?	○	○	○	○	○
9. Dalam tempoh empat minggu yang lepas, berapa kerap anda berasa sangat sedih sehingga tiada apa boleh menceriakan anda?	○	○	○	○	○
10. Dalam tempoh empat minggu yang lepas, berapa kerap anda merasakan tiada harga diri?	○	○	○	○	○

For peer review only

For peer review only - http://bmjopen.bmj.com/site/about/guidelines.xhtml

PCL-5

Berikut adalah senarai masalah yang sering dihadapi oleh seseorang individu sebagai tindak balas kepada suatu pengalaman yang sangat menekankan. Sila baca setiap soalan dengan teliti dan pilih satu nombor yang tertera di sebelah kanan untuk menunjukkan sebanyak mana anda terjejas dengan masalah tersebut pada bulan lalu.

Pada bulan lepas, sebanyak mana anda terganggu dengan:

	Tidak langsung 0	Sedikit 1	Sederhana 2	Sering 3	Sangat terjejas 4
1. Kenangan yang tidak diingini, berulang, dan mengganggu disebabkan oleh pengalaman buruk yang dialami?	○	○	○	○	○
2. Mimpi buruk yang berulang disebabkan oleh pengalaman buruk yang dialami?	○	○	○	○	○
3. Tiba-tiba terasa atau berperangai seolah-olah pengalaman buruk yang dialami itu berulang lagi (seakan-akan anda kembali ke situasi tersebut)?	○	○	○	○	○
4. Merasa sangat marah dan sedih apabila anda diingatkan dengan pengalaman buruk yang dialami?	○	○	○	○	○
5.Mengalami tindak balas fizikal yang kuat apabila anda diingatkan dengan pengalaman buruk yang dialami? (Contohnya jantung berdegup dengan laju, sukar bernafas, dan berpeluh).	○	○	○	○	○
6. Mengelak daripada mengingati sebarang kenangan, fikiran, atau perasaan yang berkaitan dengan pengalaman buruk yang dialami?	○	○	○	○	○
7. Mengelak daripada pengaruh luar yang akan mengingatkan kepada pengalaman buruk yang dialami? (Contohnya mengelak daripada berjumpa dengan orang, tempat, perbualan, aktiviti, benda, atau situasi)	○	○	○	○	○
8. Adakah anda berasa susah untuk mengingati hal-hal penting pengalaman buruk yang dialami itu?	○	○	○	○	○

For peer review only

For peer review only - http://bmjopen.bmj.com/site/about/guidelines.xhtml

9.Mempunyai tanggapan negatif yang kuat terhadap diri sendiri, orang lain, atau dunia (Contohnya, mempunyai pemikiran seperti: Saya jahat, ada sesuatu yang sangat salah dengan diri saya, tiada siapa boleh dipercayai, segalanya di dunia ini merbahaya)?	○	○	○	○	○
10. Adakah anda menyalahkan diri sendiri atau orang lain kerana berlakunya pengalaman buruk yang telah berlaku atau apa yang terjadi selepas itu?	○	○	○	○	○
11. Adakah anda mempunyai perasaan negatif yang kuat seperti takut/cemas, seram, marah, rasa bersalah, atau rasa malu?	○	○	○	○	○
12. Adakah anda hilang rasa minat kepada aktiviti yang pernah anda gemari?	○	○	○	○	○
13. Adakah anda terasa jauh atau terasing daripada orang lain?	○	○	○	○	○
14. Adakah anda sukar untuk merasakan perasaan positif (Contohnya, sukar atau tidak boleh rasa gembira atau perasaan sayang kepada orang terdekat)?	○	○	○	○	○
15. Adakah anda mudah marah, cepat sentap, atau bertindak ganas?	○	○	○	○	○
16. Adakah anda mengambil banyak risiko atau melakukan sesuatu yang boleh membahayakan diri anda?	○	○	○	○	○
17. Menjadi seorang yang sangat berwaspada atau berjaga-jaga?	○	○	○	○	○
18. Mudah rasa terkejut dan gelisah?	○	○	○	○	○
19. Adakah anda menghadapi masalah untuk menumpukan perhatian?	○	○	○	○	○
20. Adakah anda menghadapi masalah untuk tidur dan kekal tidur?	○	○	○	○	○

For peer review only

For peer review only - http://bmjopen.bmj.com/site/about/guidelines.xhtml

WHO-5

Untuk setiap kenyataan di bawah, sila nyatakan perasaan yang paling dirasai sejak dua minggu yang lepas. Dalam tempoh dua minggu yang lepas

	Sepanjang masa 5	Selalu 4	Seringkali (lebih daripada separuh masa) 3	Kadang-kadang (kurang daripada separuh masa) 2	Jarang 1	Tidak pernah 0
1. Saya berasa ceria dan bersemangat	○	○	○	○	○	○
2. Saya berasa tenang dan santai	○	○	○	○	○	○
3. Saya berasa aktif dan bertenaga	○	○	○	○	○	○
4. Saya berasa segar dan cukup rehat apabila bangun pagi	○	○	○	○	○	○
5. Kehidupan seharian saya dipenuhi dengan perkara yang saya minati	○	○	○	○	○	○

For peer review only

For peer review only - http://bmjopen.bmj.com/site/about/guidelines.xhtml

PTGI

Untuk setiap kenyataan di bawah, sila nyatakan sebanyak mana perubahan yang telah berlaku kepada hidup anda sejak peristiwa Covid-19, menggunakan skala di bawah.

	Tidak langsung 0	Sangat sedikit 1	Agak sedikit 2	Sederhana 3	Agak banyak 4	Sangat banyak 5
1. Saya ubah keutamaan terhadap apa yang penting dalam hidup ini.	○	○	○	○	○	○
2. Saya lebih menghargai nilai hidup saya sendiri.	○	○	○	○	○	○
3. Saya dapat memupuk minat yang baru.	○	○	○	○	○	○
4. Perasaan berdikari saya lebih meningkat.	○	○	○	○	○	○
5. Saya lebih memahami perkara kerohanian.	○	○	○	○	○	○
6. Saya dapat lihat yang saya boleh mengharapkan orang lain ketika saya di dalam kesusahan.	○	○	○	○	○	○
7. Saya memulakan perjalanan hidup yang baru.	○	○	○	○	○	○
8. Saya rasa lebih dekat dan akrab dengan orang lain.	○	○	○	○	○	○
9. Saya lebih bersedia untuk meluahkan emosi saya.	○	○	○	○	○	○
10. Saya yakin bahawa saya boleh melalui saat kesusahan.	○	○	○	○	○	○
11. Saya boleh melakukan perkara yang lebih bermanfaat dalam hidup saya.	○	○	○	○	○	○
12. Saya lebih senang menerima sebarang keputusan kepada perkara yang telah berlaku.	○	○	○	○	○	○

For peer review only

For peer review only - http://bmjopen.bmj.com/site/about/guidelines.xhtml

13. Saya dapat lebih bersyukur dan menghargai setiap hari yang ada.	○	○	○	○	○	○
14. Banyak peluang baru muncul yang sukar untuk didapatkan sebelum ini.	○	○	○	○	○	○
15. Rasa belas kasihan saya kepada orang lain meningkat.	○	○	○	○	○	○
16. Saya tingkatkan usaha saya dalam hubungan (bersama orang lain).	○	○	○	○	○	○
17. Saya lebih cenderung untuk mengubah perkara yang perlu diubah.	○	○	○	○	○	○
18. Kepercayaan kepada agama saya semakin meningkat.	○	○	○	○	○	○
19. Saya dapati yang saya lebih kuat daripada yang saya sangkakan.	○	○	○	○	○	○
20. Saya dapat belajar dan ketahui betapa baiknya orang-orang disekeliling saya.	○	○	○	○	○	○
21. Saya dapat terima dengan lebih baik bahawa saya memerlukan orang lain.	○	○	○	○	○	○

For peer review only

International Study – Somalia (Somali)

Sociodemographic Information

1. Jinsiga

- ◯ Lab
- ◯ Dheddig
- ◯ Mid Kale
- ◯ Ma jecli inaa ka hadlo

2. Da'daada (Sanad)

____

3. Xaalada guurka

- ◯ Waligeey ma guursan
- ◯ Qof ayaan xiriir la leeyahay
- ◯ Waa guursaday
- ◯ Kala maqanahay
- ◯ Kala tagnay
- ◯ Leeyga dhintay

4. Apa agama yang Anda?

- ◯ Diin la'aan
- ◯ Islam
- ◯ Kristen
- ◯ Hindu
- ◯ Budha

For peer review only - http://bmjopen.bmj.com/site/about/guidelines.xhtml

For peer review only

○ Mid kale (Fadlan qeex) ____

5. Jinsiyadaada maxeey tahay? (waad sheegi kartaa wax ka badan hal)

____

6. Luqadee uugu hadashaa si aad u fiican nolol maalmeedkaada?

____

Labada su'aalo ee soo socda waxa laguugu weydiinayaa heerka luqadaada Ingiriisiga.
6a. Si fiican ayaan Ingirisiga u fahmayaa

○ Aad ayaan uuga soo horjeedaa1

○ Waan diidanahay 2

○ Mana kuugu raacsani mana diidani 3

○ Waan kuugu raacsanahay 4

○ Aad ayaan kuugu raacsanahay 5

6b. Si fiican ayaan Ingiriisiga uugu hadli karaa

○ Maya haba yaraatee 1

○ Kis yar 2

○ Kistoo yar 3

○ Xoogaa 4

○ In aad u badan 5

6. Waa maxay heerka kuugu sareeyo ee waxbarashada?

○ Ma heysto waxbarasho rasmi ah (waxaan ka qeybgalay dugsiga, fasalka ESOL)

○ Dugsiga sare (tusale ahaan NCEA,dibloomada IB, shahaadada dugsiga oo dibada)

○ Shahaadada heerka sadexaad (tusaale shahaado, Diblooma ama shahaadda ganacsiga - koorso 3 sano ka ayar)

For peer review only - http://bmjopen.bmj.com/site/about/guidelines.xhtml

○ Shahaadada bajalarka (koorsada 3-4 sanno)

○ Shahaadada jaamacada wixi ka sareeya bajalarka (dibloma PG, Mastarka, Dhaqtarnimada)

7. Heerka waxbarashada ama shaqadaada hada (Fadlan muuji mid kastoo ku quseya)

☐ Waxaan bartaa waqti buuxo

☐ Waxaan bartaa waqti barkii

☐ Baagamuudo

☐ Shaqo waqti buuxo (Full-time)

☐ Shaqo waqti barkii (Part-time)

☐ Iskii u shaqeysta waqti buuxa

☐ Full-time self employed

☐ Fasaxa waalidnimada (umusha) oo lacagteydana ii socoto

☐ Waalid guriga jooga (lacag la'aan)

☐ Ka fariistay

☐ Wax kale (fadlan noo sheeg) ____

8. Shaqada

____

9. Dakhliga qoyska ee bil walba (sarifka/lacagaha wadanakaada)

____

9a. Marka la isla barbardhigo dakhliga dhexdhexaadka (isku celceliska), sidee ayaad u qiimeyn lahayd dakhliga qoyskaada?

○ Wuu ka hooseeya dhexdhexaadka

○ Dhexdhexaad (Waa isku celcelis)

For peer review only - http://bmjopen.bmj.com/site/about/guidelines.xhtml

◯ Wuu ka sareeyaa dhexdhexaadka

10. Miyaad hadda ku jirtaa bandoow ama laguugu amray in aad guriga joogtid?

◯ Haa

◯ Maya

11. Talaalka COVID-19 maqaadatay?

◯ Haa

◯ Maya

11a. Hadii aadan Willi is talaalin, ma jeclaan lahayd in aad is talaashid mustaqbalka?

◯ Haa

◯ Maya

12. Muuji baayaan walba ee soo socda heerka isbadal ee COVID-19 ku sababay noloshaada, adiga oo isticmaalaaya qiyaasta soo socoto. Halkan waxaa lagu soo koobay dhibaatooyinka ay dadka qaar kala kulmeen COVID-19: (Fadlan noo sheeg intaa kartid)

☐ Masiibo dabiici ah (sida fatahaada, dhulgariirka iwm)

☐ Ku noolaashada dhul dagaal kasocda ama goob jooga qilaafka militariga

☐ Caruurta ka yar 16 oo rafaad kusoo koray (sida dayacaada, xoogsheegsiga, dilka iyo fara xumeeyn)

☐ Dil ama kofsi 16 jir ka weyn

☐ Dhaawac weyn oo jirka

☐ Kuwa kale — fadlan Sii faahfaahi (hadii aad rabtid)

____

☐ Waxaan oo dhan ma aha

For peer review only

For peer review only - http://bmjopen.bmj.com/site/about/guidelines.xhtml

Covid Psychosocial Impacts Scale

Saameynta Shaqsiga

Bayaanka soo socda, fadlan kaga jawaab Haa/Maya haddii adiga kuugu dhacday arintaan Covid-19 dartiis. Hadday Haa tahay, fadlan sheeg xaddiga walaaca ay tani ku gaarsiisay adigoo hoos ku qayaaseysid lambarka ku habboon.

1. Ma qabtaa xaalad caafimaad oo kaa dhigi karta mid u nugul Covid-19?

- **Maya** 0
- **Haa** Walaac malahan haba yaraatee 1
- **Haa** Walaac yar 2
- **Haa** Walaac dhexdhexaad ah 3
- **Haa** Walaac badan 4
- **Haa** Walaac tiro badan 5

2. Maka cabsatay inaad la kulantid qof qaba Covid-19?

- **Maya** 0
- **Haa** Walaac malahan haba yaraatee 1
- **Haa** Walaac yar 2
- **Haa** Walaac dhexdhexaad ah 3
- **Haa** Walaac badan 4
- **Haa** Walaac tiro badan 5

3. Ma kulatahay in uu COVID-19 kuugu dhacay?

- **Maya** 0
- **Haa** Walaac malahan haba yaraatee 1
- **Haa** Walaac yar 2
- **Haa** Walaac dhexdhexaad ah 3

For peer review only - http://bmjopen.bmj.com/site/about/guidelines.xhtml

- **Haa** Walaac badan 4
- **Haa** Walaac tiro badan 5

3a. Waligaa malaaga helay COVID-19?

- **Maya** 0
- **Haa** Walaac malahan haba yaraatee 1
- **Haa** Walaac yar 2
- **Haa** Walaac dhexdhexaad ah 3
- **Haa** Walaac badan 4
- **Haa** Walaac tiro badan 5

For peer review only

3b. Miyaad leedahay astaamaha COVID-19 oo joogta ah?

- **Maya** 0
- **Haa** Walaac malahan haba yaraatee 1
- **Haa** Walaac yar 2
- **Haa** Walaac dhexdhexaad ah 3
- **Haa** Walaac badan 4
- **Haa** Walaac tiro badan 5

Sameeynta Qoyska

Bayaanka soo socda, fadlan kaga jawaab Haa/Maya haddii arintaan ku dhacday dadka qoyskaada Covid-19 dartiis. Hadday Haa tahay, fadlan sheeg xaddiga walaaca ay tani ku gaarsiisay adigoo hoos ku qayaaseysid lambarka ku habboon.

1. Qof ka mid ah qoyskaada ma jiraa oo u nugul Covid-19 maadaamo ey jirto xaalad caafimaad?

- **Maya** 0
- **Haa** Walaac malahan haba yaraatee 1

For peer review only - http://bmjopen.bmj.com/site/about/guidelines.xhtml

- **Haa** Walaac yar 2
- **Haa** Walaac dhexdhexaad ah 3
- **Haa** Walaac badan 4
- **Haa** Walaac tiro badan 5

2. Ma dareensantahay in qof qoyskaada ka mid ah halis u yahay inuu la kulmo qof qaba Covid-19 markuu shaqada joogo?

- **Maya** 0
- **Haa** Walaac malahan haba yaraatee 1
- **Haa** Walaac yar 2
- **Haa** Walaac dhexdhexaad ah 3
- **Haa** Walaac badan 4
- **Haa** Walaac tiro badan 5

3. Ma kulatahay in qof qoyskaada soke ka mid ah in uu ku dhacay COVID-19?

- **Maya** 0
- **Haa** Walaac malahan haba yaraatee 1
- **Haa** Walaac yar 2
- **Haa** Walaac dhexdhexaad ah 3
- **Haa** Walaac badan 4
- **Haa** Walaac tiro badan 5

3a. Ma jiraa qof qoyskaada soke ka mid ah oo laga helay COVID-19?

- **Maya** 0
- **Haa** Walaac malahan haba yaraatee 1

For peer review only - http://bmjopen.bmj.com/site/about/guidelines.xhtml

- Haa Walaac yar 2
- Haa Walaac dhexdhexaad ah 3
- Haa Walaac badan 4
- Haa Walaac tiro badan 5

3b. Majiraa qof qoyskaada soke ka mid ah ee leh astaamaha COVID-19 oo joogta ah?

- **Maya** 0
- **Haa** Walaac malahan haba yaraatee 1
- **Haa** Walaac yar 2
- **Haa** Walaac dhexdhexaad ah 3
- **Haa** Walaac badan 4
- **Haa** Walaac tiro badan 5

3c. Majiraa qof qoyskaada soke ah ee u geeriyooday/dhintay COVID-19?

- **Maya** 0
- **Haa** Walaac malahan haba yaraatee 1
- **Haa** Walaac yar 2
- **Haa** Walaac dhexdhexaad ah 3
- **Haa** Walaac badan 4
- **Haa** Walaac tiro badan 5

4. Majiraan dad qoyskaada soke ka mid ah ee dibada ku maqan oo aad uuga walwalsantahay in COVID-19 uu ku dhoco?

- **Maya** 0
- **Haa** Walaac malahan haba yaraatee 1

For peer review only

- **Haa** Walaac yar 2
- **Haa** Walaac dhexdhexaad ah 3
- **Haa** Walaac badan 4
- **Haa** Walaac tiro badan 5

5. Ma leedahay qoyska dhow dibedda ku sugan oo aadan awoodin inaad booqato ama aan kusoo booqan Karin adiga?

- **Maya** 0
- **Haa** Walaac malahan haba yaraatee 1
- **Haa** Walaac yar 2
- **Haa** Walaac dhexdhexaad ah 3
- **Haa** Walaac badan 4
- **Haa** Walaac tiro badan 5

Shaqo

Bayaanka soo socda, fadlan kaga jawaab Haa/Maya/Ma i qusayso haddii adiga ama qof kula nool ku dhacday Covid-19 dartiis. Hadday Haa tahay, fadlan sheeg xaddiga walaaca ay tani ku gaarsiisay adigoo hoos ku qayaaseysid lambarka ku habboon.

1. Ma idinkujiraa qoyskiina qof loo tixgaliyay in uu yahay shaqaalaha daruuriga (muhiimka ah) (shaqaalaha daryeelka caafimaadka, fulinta sharciga, adeegyada degdegga ah, kuwa bixiyaha adeegyada daruuriga ah)?

- **Maya** 0
- **Haa** Walaac malahan haba yaraatee 1
- **Haa** Walaac yar 2
- **Haa** Walaac dhexdhexaad ah 3
- **Haa** Walaac badan 4
- **Haa** Walaac tiro badan 5

For peer review only - http://bmjopen.bmj.com/site/about/guidelines.xhtml

2. Isbadal weyn ma ku yimid saacadaha aad adiga ama qof qoyskaada ka tirsan aad shaqeyn jirteen?

- **Maya** 0
- **Haa** Walaac malahan haba yaraatee 1
- **Haa** Walaac yar 2
- **Haa** Walaac dhexdhexaad ah 3
- **Haa** Walaac badan 4
- **Haa** Walaac tiro badan 5

3. Ma dhacday in aad adiga ama qof qoyskaada katirsan in aad ku qasbanaateen in goobtii shaqada isku badashay guriga (guriga adigoo joogta sii wadid shaqadii)?

- **Maya** 0
- **Haa** Walaac malahan haba yaraatee 1
- **Haa** Walaac yar 2
- **Haa** Walaac dhexdhexaad ah 3
- **Haa** Walaac badan 4
- **Haa** Walaac tiro badan 5

4. Majiraa qof qoyskaada ka mid ah ee ku qasbanaaday in uu shaqada badasho?

- **Maya** 0
- **Haa** Walaac malahan haba yaraatee 1
- **Haa** Walaac yar 2
- **Haa** Walaac dhexdhexaad ah 3
- **Haa** Walaac badan 4
- **Haa** Walaac tiro badan 5

For peer review only - http://bmjopen.bmj.com/site/about/guidelines.xhtml

5. Majiraa qof qoykaada ka tirsan ee shaqadiisii waayay?

- **Ma i qusayso**
- **Maya** 0
- **Haa** Walaac malahan haba yaraatee 1
- **Haa** Walaac yar 2
- **Haa** Walaac dhexdhexaad ah 3
- **Haa** Walaac badan 4
- **Haa** Walaac tiro badan 5

6. Ganacsi/bisinis aad adiga ama qof kula nool lahaa am aad ka shaqeeyneeyseen miyaa halis u ah inuu sii socon waayo?

- **Ma i qusayso**
- **Maya** 0
- **Haa** Walaac malahan haba yaraatee 1
- **Haa** Walaac yar 2
- **Haa** Walaac dhexdhexaad ah 3
- **Haa** Walaac badan 4
- **Haa** Walaac tiro badan 5

Dakhliga

Bayaanka soo socda, fadlan kaga jawaab Haa/Maya haddii uu qoyskaada quseeya Covid-19 dartiis. Hadday Haa tahay, fadlan sheeg xaddiga walaaca ay tani ku gaarsiisay adigoo hoos ku qayaaseysid lambarka ku habboon.

1. Dhaqaalaha reerkaada aad miyuu hoos u dhacay?

- **Maya** 0
- **Haa** Walaac malahan haba yaraatee 1

For peer review only - http://bmjopen.bmj.com/site/about/guidelines.xhtml

- Haa Walaac yar 2
- Haa Walaac dhexdhexaad ah 3
- Haa Walaac badan 4
- Haa Walaac tiro badan 5

2. Qoyskiina miyaad awoodi wayseen inaad bixisaan biilasha ama adeega cuntada?

- **Maya** 0
- **Haa** Walaac malahan haba yaraatee 1
- **Haa** Walaac yar 2
- **Haa** Walaac dhexdhexaad ah 3
- **Haa** Walaac badan 4
- **Haa** Walaac tiro badan 5

Xiriika

Bayaanka soo socda, fadlan kaga jawaab Haa/Maya haddii adiga kuugu dhacday arintaan Covid-19 dartiis. Hadday Haa tahay, fadlan sheeg xaddiga walaaca ay tani ku gaarsiisay adigoo hoos ku qayaaseysid lambarka ku habboon. Covid-19 dartiis…..

1. Dhibaatada qoyska miyeey sii adkaatay?

- **Maya** 0
- **Haa** Walaac malahan haba yaraatee 1
- **Haa** Walaac yar 2
- **Haa** Walaac dhexdhexaad ah 3
- **Haa** Walaac badan 4
- **Haa** Walaac tiro badan 5

2. Xiriirkii aad la lahayd asxaabtaada miyuu soo yaraaday (si toos ah ama dhinaca qadka)?

For peer review only

- **Maya** 0
- **Haa** Walaac malahan haba yaraatee 1
- **Haa** Walaac yar 2
- **Haa** Walaac dhexdhexaad ah 3
- **Haa** Walaac badan 4
- **Haa** Walaac tiro badan 5

Isbeddelka ku yimid nolol maalmeedka iyo dabeecadaha

Bayaanka soo socda, fadlan kaga jawaab Haa/Maya haddii adiga kuugu dhacday arintaan Covid-19 dartiis. Hadday Haa tahay, fadlan sheeg xaddiga walaaca ay tani ku gaarsiisay adigoo hoos ku qayaaseysid lambarka ku habboon. Covid-19 dartiis.....

1. Cabuqa Korona (Covid-19) miyuu isbadal ku keenay nolol maalmeedkaada (sida hurdada, jimicsiga, firaaqada, hiwaayadaha aad xiiseeysid)?

- **Maya** 0
- **Haa** Walaac malahan haba yaraatee 1
- **Haa** Walaac yar 2
- **Haa** Walaac dhexdhexaad ah 3
- **Haa** Walaac badan 4
- **Haa** Walaac tiro badan 5

2. Miyay taasi sababtay inaad dib u dhigto ama iska dhaaftid inaad raadsato daryeel caafimaad mar ad adiga ama qof qoyskaada u baahateen?

- **Maya** 0
- **Haa** Walaac malahan haba yaraatee 1
- **Haa** Walaac yar 2
- **Haa** Walaac dhexdhexaad ah 3

For peer review only - http://bmjopen.bmj.com/site/about/guidelines.xhtml

- Haa Walaac badan 4
- Haa Walaac tiro badan 5

3. Tani miyay yareeysay ka-qayb-qaadashadaada howlaha diinta (shaqsi ahaan ama khadka tooska ah)?

- **Ma i qusayso**
- **Maya** 0
- **Haa** Walaac malahan haba yaraatee 1
- **Haa** Walaac yar 2
- **Haa** Walaac dhexdhexaad ah 3
- **Haa** Walaac badan 4
- **Haa** Walaac tiro badan 5

4. Miyey jireen isbeddelo kale oo weyn oo ku yimid nidaamka/nolol maalmeedka?

- **Maya** 0
- **Haa (Haddii ay sidaas tahay fadlan sheeg)** 1 ____

Hadday Haa tahay, fadlan sheeg xaddiga walaaca ay tani ku gaarsiisay adigoo hoos ku qayaaseysid lambarka ku habboon.

- Walaac malahan haba yaraatee 1
- Walaac yar 2
- Walaac dhexdhexaad ah 3
- Walaac badan 4
- Walaac tiro badan 5

Soo bandhigista macluumaadka ku saabsan Covid-19

1. Xageed kaheshaa warbixinta ku saabsan Covid-19?
(Fadlan qeex mid kasto ad isticmaashid)

For peer review only - http://bmjopen.bmj.com/site/about/guidelines.xhtml

- News/wararka
- Facebook / Twitter
- Websites/boga caafimaadka
- Hadal isgaarsiis
- Meela kale (Fadlan qeex) ____

2. Culeys ilaa intee ayuu wararka ku saabsan Covid-19 kuu geystay?

- Walaac malahan haba yaraatee 1
- Walaac yar 2
- Walaac dhexdhexaad ah 3
- Walaac badan 4
- Walaac tiro badan 5

Isbeddelada ku yimid Covid-19 dartiis

Arimaha soo socda fadlan sheeg xaddiga walaaca ay tani ku gaarsiisay adigoo hoos ku qayaaseysid lambarka ku habboon. Isbarbardhiga hadda iyo wixii ka horeeyay Covid-19---

1. Sideed u qiimeyn lahayd heerka walaacaaga shaqsi ahaan?

- Walaac yar 1
- Xoogaa walaac yar 2
- Islameeshii 3
- Walwal batay 4
- Walaaca oo aad u batay 5

2. Maxaad ku qiimeyn lahayd walaaca guud ee reerkaada/gurigaada?

- Walaac yar 1

For peer review only - http://bmjopen.bmj.com/site/about/guidelines.xhtml

- Xoogaa walaac yar 2
- Islameeshii 3
- Walwal batay 4
- Walaaca oo aad u batay 5

3. Sideed u qiimeyn lahayd ladnaantaada nafsiyeed?

- Aad uuga fiican sidii hore 1
- Sidii hore ka fiican 2
- Sideedii 3
- Sidii ka daran 4
- Aad uga daran 5

4. Sideed u qiimeyn laheyd wanaagsanaanta nafsiyeed ee dadka qoyskaaga?

- Aad ufiican 1
- Fiican 2
- Ku saabsan isla mid 3
- Ka xun 4
- Aad uga xun 5

For peer review only - http://bmjopen.bmj.com/site/about/guidelines.xhtml

K-10

Tobanka su'aalo ee xiggo waxay wax ka weydiinayaan sida aad dareemeysay 4 isbuuc ee la soo dhaafay. Su'aal walba, tilmaam intajeer aad dareentay qaabkaas.

	Marnaba 1	Mar dhif 2	Mar mar 3	Inta badan 4	Mar kasta 5
1. Afartii isbuuc ee la soo dhaafay, intee jeer ayaad dareentay daal oo aan lahayn sabab wanaagsan?	○	○	○	○	○
2. Afartii isbuuc ee la soo dhaafay, intee jeer ayaad dareentay walwal?	○	○	○	○	○
3. Afartii isbuuc ee la soo dhaafay, intee jeer ayaad dareentay walwal zaa'id ah oo waxba aanan ku dajineyn?	○	○	○	○	○
4. Afartii isbuuc ee la soo dhaafay, intee jeer ayaad dareentay rajo la' aan?	○	○	○	○	○
5. Afartii isbuuc ee la soo dhaafay, intee jeer ayaad dareentay nasasho la'aan ama samir la'aan?	○	○	○	○	○
6. Afartii isbuuc ee la soo dhaafay, intee jeer ayaad dareentay nasasho la'aan oo aadan fadhin karin?	○	○	○	○	○
7. Afartii isbuuc ee la soo dhaafay, intee jeer ayaad dareentay murugo?	○	○	○	○	○
8. Afartii isbuuc ee la soo dhaafay, intee jeer ayaad dareentay in wax walba ay dadaal/halgan ahaayeen?	○	○	○	○	○
9. Afartii isbuuc ee la soo dhaafay, intee jeer ayaad dareentay murugo badan oo waxba aanan ku farxad gelineynin?	○	○	○	○	○
10. Afartii isbuuc ee la soo dhaafay, intee jeer ayaad dareentay qiimo la'aan?	○	○	○	○	○

For peer review only

For peer review only - http://bmjopen.bmj.com/site/about/guidelines.xhtml

PCL-5

Hoos waa liiska dhibaatooyinka oo dadka mararka qaar ay qabaan oo jawaab u ah waaya aragnimada walbahaarka badan. Fadlan si taxadar ah u aqri dhibaato walba kadibna tilmaam inta ay ku dhibtay dhibaatadaas bishii la soo dhaafay. Bisha la soo dhaafay, intee ayay ku dhibtay:

	Maya gabi ahan 0	Xooga yar 1	Dhexaad 2	Ilaa xad 3	Xad dhaaf ah 4
1. Soo nonoqoshada xasuusta aan la rabin oo khibradaha argagaxa leh?	○	○	○	○	○
2. Soo nonoqoshada riyooyinka jahwareerka ah ee ku saabsan khibradaha argagaxa leh?	○	○	○	○	○
3. Dareen lama filaan ah ama u dhaqanka sidii ineey hada khibradii murugada lahayd dhaceysa markale (sidii ineey hada dhaceeyso oo kale)?	○	○	○	○	○
4. Dareenka murugada aadka ah markii wax ku soo xasuusiyaan waaya aragnimada murugada leh?	○	○	○	○	○
5. Falcelinada jirka xoogan markii wax ku soo xasuusiyaan waaya aragnimadii murugada lahyd (tusaale ahaan, garaaca wadnaha, neefsashada oo dhib noqota iyo dhididka)?	○	○	○	○	○
6. Ka fogaanshaha xasuusta, afkaaraha, ama dareemida la xiriirto waaya aragnimada/khibradaha murugada leh?	○	○	○	○	○
7. Ka fogaanshaha wax kastoo kusoo xasuusiya walbahaarkii (tusaala ahaan, dadka, meelaha, wadasheekeysiga, shaqooyinka, waxyaabaha ama xaaladaha)?	○	○	○	○	○
8. Miyaad dhib ku qabtaa inaa xasuusatid qeyba muhiim ah ee khibradihii walbahaarka lahaa?	○	○	○	○	○
9. Ka qabida aaminaad xun oo xoogan nafsadaada, dadka kale, ama caalamka (tusaale ahaan, lahaanshaha afkaaraha sida; Waan xummahay, wax baa iga qaldan,	○	○	○	○	○

For peer review only

For peer review only - http://bmjopen.bmj.com/site/about/guidelines.xhtml

majiro qof la aamini karo, caalamka oo dhan waa halis)?					
10. Ku eedeynta nafsadaada ama qof kale ee khibradihii walbahaarka lahaa ama wixii dhacay markaas kadib?	○	○	○	○	○
11. Lahaanshaha dareen xun ee xoogan sida cabsida, naxdinta, xanaaqa, dambiilenimada, ama ceeb?	○	○	○	○	○
12. Ka xiisadhicida howlihii aad jeclaan jirtay?	○	○	○	○	○
13. Dareemida ka fogaanshaha ama ka go'ida dadka kale?	○	○	○	○	○
14. Dhibaato ma ku qabtaa inaad dareento dareen wanaagsan (tusaale ahaan, inaad dareento farxad ama aad jeclaato dadka kuu dhow)?	○	○	○	○	○
15. Anshax xumo, xanaaqa, ama ku dhaqmida si dagaal ah?	○	○	○	○	○
16. Halis is galis badan ama sameeynta waxyaabaha kuugu sababi karo dhibaato?	○	○	○	○	○
17. Ma dareentaa "feejignaan cajiib ah" ama fiirin badan ama illaalo?	○	○	○	○	○
18. Miyaad dareentaa xasilaad la'aan ama si sahlan inaad u naxdo?	○	○	○	○	○
19. Ma kuugu adagtahay inaad diirad saartid/xogsaartid?	○	○	○	○	○
20. Ma kuugu adagtahay ineey hurdo kaa soo dhacdo ama inaad sii hurudo?	○	○	○	○	○

For peer review only

For peer review only - http://bmjopen.bmj.com/site/about/guidelines.xhtml

WHO-5

Fadlan noo tilmaam shanta arimood ee soo socda sida ugu dhow uu dareenkaaga ahaa labadii isbuuc ee la soo dhaafay. Labadii isbuuc ee lasoo dhaafay

	Marwalba 5	Intabadan wakhtiga 4	Inkabadan kalabar wakhtiga 3	Inkayar kalabar wakhtiga 2	Marmar wakhtiga 1	Marnaba 0
1. Waxaan dareemay farxad iyo hami sare.	○	○	○	○	○	○
2. Waxaan dareemay daganaan iyo xasilooni.	○	○	○	○	○	○
3. Waxaan dareemay firfircooni iyo awoodbadan.	○	○	○	○	○	○
4. Waxaan soo kacay anigoo dareemaya nasasho iyo daganaan.	○	○	○	○	○	○
5. Maalintayda oo dhan waxaa buuxiyay waxyalo ixiiso galiya.	○	○	○	○	○	○

For peer review only

For peer review only - http://bmjopen.bmj.com/site/about/guidelines.xhtml

PTGI

Muuji baayaan walba ee soo socda heerka isbadal ee Maarso 15-keedii ku sababay noloshaada, adoo isticmaalaaya qiyaasta soo socoto.

	Marnaba 0	Wax yar 1	Xoogaa Roon 2	Dhexaad 3	In badan 4	Wax badan 5
1. Waxaan badelay muhiimadeyda ku saabsan waxa nolosha muhiim u ah.	○	○	○	○	○	○
2. Waxaan qadarin weyn u leeyahay qiimaha noloshayda.	○	○	○	○	○	○
3. Waxaan hormariyay xiiso/dana cusub.	○	○	○	○	○	○
4. Waxaan dareemayaa isku kalsooni.	○	○	○	○	○	○
5. Waxaan si fiican uu fahamsanahay arrimaha diinta.	○	○	○	○	○	○
6. Waxaan si cad u arkaa inaan ku xisaabtami karo dadka waqtiyada dhibaatada.	○	○	○	○	○	○
7. Waxaan nolosheyda u aasaasay wado cusub.	○	○	○	○	○	○
8. Waxaan dareensanahay inaan dadka aad uugu dhawahay.	○	○	○	○	○	○
9. Waxaan diyaar u ahay inaan muujiyo dareenkeyga.	○	○	○	○	○	○
10. Waxaan si fiican u ogahay in aan wax ka qaban karo dhibaatoyinka.	○	○	○	○	○	○
11. Waxaan awooda in aan noloshayda wax fiican ku sameeyo.	○	○	○	○	○	○
12. Waxaan si fiican u awoodaa inaan aqbalo siday wax u dhacayaan.	○	○	○	○	○	○

For peer review only

13. Waxaan si fiican uga mahadcelin karaa maalin kasta.

14. Fursado cusub ayaa la heli karaa oo awal jirin.

15. Waxaan u qabaa naxariis badan dadka kale.

16. Waxaan dadaal badan geliya cilaaqaadkeyga.

17. Waxay u badan tahay inaan isku dayo inaan beddelo waxyaabaha u baahan badal.

18. Waxaan leeyahay caqiido diineed oo xoog badan.

19. Waxaan ogaaday inaan ka xoog badanahay sidaan u maleeynayay.

20. Waxaan bartay ineey dadka aad u wanaagsan yihiin.

21. Waxaan si fiican u qirraa inaan u baahankaro dadka kale.

For peer review only

International Study - Turkiye (Turkish)

Sociodemographic Information

1. Cinsiyet

- ◯ Erkek
- ◯ Kadın

2. Yaş

____

3. Medeni Hali

- ◯ Bekar
- ◯ İlişkisi var
- ◯ Evli
- ◯ Ayrılmış
- ◯ Boşanmış
- ◯ Dul

4. Dininiz nedir?

- ◯ Din yok
- ◯ İslâm
- ◯ Hıristiyanlık
- ◯ Hinduizm
- ◯ Budizm
- ◯ Diğer (lütfen belirtiniz) ____

For peer review only

5. Eğitim düzeyiz nedir?(En son almış olduğunuz derece)?

- ◯ Okur Yazar
- ◯ İlkokul Mezunu
- ◯ Ortaokul Mezunu
- ◯ Lise Mezunu
- ◯ Üniversite Öğrencisi
- ◯ Önlisans Mezunu
- ◯ Lisans Mezunu
- ◯ Yüksek Lisans Mezunu
- ◯ Doktora Mezunu

6. Mevcut Çalışma veya iş durumunuz (Birden fazla işaretleyebilirsiniz.)

- ☐ Öğrenci
- ☐ Çalışmıyor/İş Arıyor
- ☐ Tam zamanlı maaşlı çalışma
- ☐ Yarı zamanlı maaşlı çalışma
- ☐ Tam zamanlı serbest meslek
- ☐ Yarı zamanlı serbest meslek
- ☐ Ücretli ebeveyn izninde
- ☐ Evde ebeveyn yada ücretsiz ebeveyn izninde
- ☐ Emekli
- ☐ Diğer (Lütfen Belirtin) ____

For peer review only

7. Mesleğiniz

____

8. Aylık hane geliri (yerel para biriminde)

____

9. Ortalama gelirle karşılaştırıldığında, hanenizin gelirini nasıl değerlendirirsiniz?

○ Ortalamanın altında

○ Ortalama

○ Ortalamanın üstünde

10. Şu anda karantinada ya da sokağa çıkma yasağında mısınız?

○ Evet1

○ Hayır 0

11. Covid-19 aşısı oldunuz mu?

○ Evet1

○ Hayır 0

11a. Aşı olmadıysanız, gelecekte Covid-19 aşısı olmak ister misiniz?

○ Evet1

○ Hayır 0

12. Bazı insanların geçmişte maruz kaldıkları diğer travmatik olayların yeni sorunlarla başa çıkmayı kolaylaştırabilecek ya da zorlaştırabilecek olduğunun farkındayız.

Covid-19 salgınından önce aşağıdakilerden herhangi birine şahit oldunuz mu veya herhangi birini yaşadınız mı?: **(Lütfen uygun olanları işaretleyin)**

☐ Doğal afet (sel, deprem, vs.)

For peer review only - http://bmjopen.bmj.com/site/about/guidelines.xhtml

- ☐ Savaş bölgesinde yaşamak veya askeri çatışmaya maruz kalmak
- ☐ 16 yaş öncesi çocukluk sıkıntısı (mesela ihmal, zorbalık, fiziksel veya cinsel saldırı, vs.)
- ☐ 16 yaş sonrası fiziksel veya cinsel saldırı
- ☐ Ciddi fiziksel kazalar
- ☐ Diğer – isteğe bağlı – bunun ne olduğunu açıklayabilir misiniz lütfen? ____
- ☐ Yukarıdakilerin hiçbiri

For peer review only

For peer review only - http://bmjopen.bmj.com/site/about/guidelines.xhtml

Covid Psychosocial Impacts Scale

Kişisel etki

Aşağıdaki ifadeler için, Covid-19 döneminde sizde böyle bir durum gerçekleştiyse lütfen Evet / Hayır ile yanıt verin. Evet ise, lütfen aşağıdaki ölçekte bunun sizde neden olduğu stres miktarını belirtin.

1. Covid-19'dan daha olumsuz etkilenmenize neden olabilecek bir sağlık probleminiz var mı (örn. Kalp hastalığı, nefes almada zorluk, zayıf bağışıklık sistemi, veya kanser)?

- **Hayır** 0
- **Evet** Hiç stresli değil 1
- **Evet** Biraz stres 2
- **Evet** Orta derecede stres 3
- **Evet** Oldukça fazla stres 4
- **Evet** Aşırı derecede stres 5

2. Covid-19 pozitif biriyle temas riski altında olduğunuzu hissettiniz mi?

- **Hayır** 0
- **Evet** Hiç stresli değil 1
- **Evet** Biraz stres 2
- **Evet** Orta derecede stres 3
- **Evet** Oldukça Fazla stres 4
- **Evet** Aşırı derecede stres 5

3. Covid-19 geçirdiğinizi düşünüyor musunuz?

- **Hayır** 0
- **Evet** Hiç stresli değil 1
- **Evet** Biraz stres 2

For peer review only

For peer review only - http://bmjopen.bmj.com/site/about/guidelines.xhtml

- Evet Orta derecede stres 3
- Evet Oldukça fazla stres 4
- Evet Aşırı derecede stres 5

3a. Covid-19 tanısı aldınız mı?

- **Hayır** 0
- **Evet** Hiç stresli değil 1
- **Evet** Biraz stres 2
- **Evet** Orta derecede stres 3
- **Evet** Oldukça fazla stres 4
- **Evet** Aşırı derecede stres 5

3b. Devam eden Covid-19 semptomlarınız var mı?

- **Hayır** 0
- **Evet** Hiç stresli değil 1
- **Evet** Biraz stres 2
- **Evet** Orta derecede stres 3
- **Evet** Oldukça fazla stres 4
- **Evet** Aşırı derecede stres 5

Aile etkisi

Aşağıdaki ifadeler için, Covid-19 döneminde yakın aile üyelerinizde böyle bir durum gerçekleştiyse lütfen Evet / Hayır ile yanıt verin. Evet ise, lütfen aşağıdaki ölçekte bunun sizde neden olduğu stres miktarını belirtin.

1. Herhangi bir yakın aile üyesinin Covid-19'dan daha olumsuz etkilenmelerine neden olabilecek bir sağlık problemi var mı? (örn. Kalp hastalığı, nefes almada zorluk, zayıf bağışıklık sistemi, veya kanser)?

For peer review only - http://bmjopen.bmj.com/site/about/guidelines.xhtml

- **Hayır** 0
- **Evet** Hiç stresli değil 1
- **Evet** Biraz stres 2
- **Evet** Orta derecede stres 3
- **Evet** Oldukça fazla stres 4
- **Evet** Aşırı derecede stres 5

For peer review only

2. Pandemi sürecinde herhangi bir yakın aile üyesinin, Covid-19 pozitif olan biri ile temas riski altında olduğunu düşündünüz mü/düşünüyor musunuz?

- **Hayır** 0
- **Evet** Hiç stresli değil 1
- **Evet** Biraz stres 2
- **Evet** Orta derecede stres 3
- **Evet** Oldukça fazla stres 4
- **Evet** Aşırı derecede stres 5

3. Yakın aile üyelerinizden herhangi birinin Covid-19 geçirdiğini düşünüyor musunuz?

- **Hayır** 0
- **Evet** Hiç stresli değil 1
- **Evet** Biraz stres 2
- **Evet** Orta derecede stres 3
- **Evet** Oldukça fazla stres 4
- **Evet** Aşırı derecede stres 5

For peer review only - http://bmjopen.bmj.com/site/about/guidelines.xhtml

3a. Yakın aile üyelerinin herhangi biri Covid-19 tanısı aldı mı?

- **Hayır** 0
- **Evet** Hiç stresli değil 1
- **Evet** Biraz stres 2
- **Evet** Orta derecede stres 3
- **Evet** Oldukça fazla stres 4
- **Evet** Aşırı derecede stres 5

3b. Yakın aile üyesi/üyelerinde devam eden Covid-19 semptomları var mı?

- **Hayır** 0
- **Evet** Hiç stresli değil 1
- **Evet** Biraz stres 2
- **Evet** Orta derecede stres 3
- **Evet** Oldukça fazla stres 4
- **Evet** Aşırı derecede stres 5

3c. Yakın bir aile üyesi/üyeleri Covid-19 nedeniyle vefat etti mi?

- **Hayır** 0
- **Evet** Hiç stresli değil 1
- **Evet** Biraz stres 2
- **Evet** Orta derecede stres 3
- **Evet** Oldukça fazla stres 4
- **Evet** Aşırı derecede stres 5

For peer review only

For peer review only - http://bmjopen.bmj.com/site/about/guidelines.xhtml

4. Yurtdışında Covid-19'a yakalanma riski altında olmasından endişe duyduğunuz yakın aile üyeniz var mı?

- **Hayır** 0
- **Evet** Hiç stresli değil 1
- **Evet** Biraz stres 2
- **Evet** Orta derecede stres 3
- **Evet** Oldukça fazla stres 4
- **Evet** Aşırı derecede stres 5

5. Covid-19 pandemisi nedeniyle yurtdışında ziyaret edemediğiniz veya sizi ziyaret edemeyen yakın aile üyeniz var mı?

- **Hayır** 0
- **Evet** Hiç stresli değil 1
- **Evet** Biraz stres 2
- **Evet** Orta derecede stres 3
- **Evet** Oldukça fazla stres 4
- **Evet** Aşırı derecede stres 5

İş

Aşağıdaki ifadeler için, Covid-19 döneminde bu durum sizde veya evinizdeki herhangi birinde gerçekleşmişse (Evet / Hayır / Bana uygun değil) ile yanıt verin. Evet ise, lütfen aşağıdaki ölçekte bunun sizde neden olduğu stres miktarını belirtin.

1. Siz veya evinizdeki herhangi biri temel sektör çalışanı mısınız? (örneğin sağlık hizmetleri, gıda sektörü, acil servisler, temel mal / hizmet sağlayıcıları vb.)?

- **Hayır** 0
- **Evet** Hiç stresli değil 1

For peer review only

○ **Evet** Biraz stres 2

○ **Evet** Orta derecede stres 3

○ **Evet** Oldukça fazla stres 4

○ **Evet** Aşırı derecede stres 5

2. Covid-19 pandemisi nedeniyle sizin veya evinizdeki herhangi birinin çalışma saatlerinde büyük bir değişiklik oldu mu?

○ **Hayır** 0

○ **Evet** Hiç stresli değil 1

○ **Evet** Biraz stres 2

○ **Evet** Orta derecede stres 3

○ **Evet** Oldukça fazla stres 4

○ **Evet** Aşırı derecede stres 5

3. Covid-19 pandemisi nedeniyle siz veya evinizdeki herhangi biri evden çalışmak zorunda kaldınız mı?

○ **Hayır** 0

○ **Evet** Hiç stresli değil 1

○ **Evet** Biraz stres 2

○ **Evet** Orta derecede stres 3

○ **Evet** Oldukça fazla stres 4

○ **Evet** Aşırı derecede stres 5

4. Covid-19 pandemisi nedeniyle siz veya evinizdeki herhangi biri farklı bir iş türüne geçmek zorunda kaldınız mı?

○ **Hayır** 0

For peer review only

- **Evet** Hiç stresli değil 1
- **Evet** Biraz stres 2
- **Evet** Orta derecede stres 3
- **Evet** Oldukça fazla stres 4
- **Evet** Aşırı derecede stres 5

5. Covid-19 pandemisi nedeniyle siz veya evinizdeki herhangi biri işini kaybetti mi?

- **Bana uygun değil**
- **Hayır** 0
- **Evet** Hiç stresli değil 1
- **Evet** Biraz stres 2
- **Evet** Orta derecede stres 3
- **Evet** Oldukça fazla stres 4
- **Evet** Aşırı derecede stres 5

6. Covid-19 pandemisi nedeniyle sizin veya evinizdeki bir başkasının kendi işletmesi veya çalıştığı şirket kapanma tehdidi altında mı?

- **Bana uygun değil**
- **Hayır** 0
- **Evet** Hiç stresli değil 1
- **Evet** Biraz stres 2
- **Evet** Orta derecede stres 3
- **Evet** Oldukça fazla stres 4

For peer review only - http://bmjopen.bmj.com/site/about/guidelines.xhtml

○ **Evet** Aşırı derece stres 5

Gelir

Aşağıdaki ifadeler için, Covid-19 döneminde bu durum evinizdeki herhangi birinde gerçekleştiyse (Evet / Hayır) ile yanıt verin. Evet ise, lütfen aşağıdaki ölçekte bunun sizde neden olduğu stres miktarını belirtin.

1. Covid-19 pandemisi nedeniyle hanenizde büyük bir maddi zorluk yaşadınız mı?

○ **Hayır** 0

○ **Evet** Hiç stresli değil 1

○ **Evet** Biraz stres 2

○ **Evet** Orta derecede stres 3

○ **Evet** Oldukça fazla stres 4

○ **Evet** Aşırı derecede stres 5

2. Covid-19 pandemisi nedeniyle hanehalkınız faturalarınızı ödeyebilmeniz veya mutfak masraflarınızı karşılayabilmeniz konusunda büyük bir zorluk yaşadı mı?

○ **Hayır** 0

○ **Evet** Hiç stresli değil 1

○ **Evet** Biraz stres 2

○ **Evet** Orta derecede stres 3

○ **Evet** Oldukça fazla stres 4

○ **Evet** Aşırı derecede stres 5

İlişkiler

Aşağıdaki ifadeler için, Covid-19 döneminde sizde böyle bir durum gerçekleştiyse lütfen Evet / Hayır ile yanıt verin. Evet ise, lütfen aşağıdaki ölçekte bunun sizde neden olduğu stres miktarını belirtin. Covid-19'a yanıt olarak....

For peer review only - http://bmjopen.bmj.com/site/about/guidelines.xhtml

1. Covid-19 döneminde aile üyeleri arasında zorluklar ve gerginlikler arttı mı?

- **Hayır** 0
- **Evet** Hiç stresli değil 1
- **Evet** Biraz stres 2
- **Evet** Orta derecede stres 3
- **Evet** Oldukça fazla stres 4
- **Evet** Aşırı derecede stres 5

2. Covid-19 pandemisi arkadaşlarınızla (fiziksel veya çevrimiçi/online) iletişiminizi azalttı mı?

- **Hayır** 0
- **Evet** Hiç stresli değil 1
- **Evet** Biraz stres 2
- **Evet** Orta derecede stres 3
- **Evet** Oldukça fazla stres 4
- **Evet** Aşırı derecede stres 5

Günlük rutin ve davranışlarda değişiklikler

Aşağıdaki ifadeler için, Covid-19 döneminde sizde böyle bir durum gerçekleştiyse lütfen (Evet / Hayır / Bana uygun değil) ile yanıt verin. Evet ise, lütfen aşağıdaki ölçekte bunun sizde neden olduğu stres miktarını belirtin. Covid-19'a yanıt olarak......

1. Covid-19 pandemisi günlük rutininizde değişikliklere yol açtı mı? (örneğin, uyku, egzersiz, boş zaman, keyif veren aktiviteler veya hobiler)

- **Hayır** 0
- **Evet** Hiç stresli değil 1
- **Evet** Biraz stres 2

For peer review only - http://bmjopen.bmj.com/site/about/guidelines.xhtml

◯ **Evet** Orta derecede stres 3

◯ **Evet** Oldukça fazla stres 4

◯ **Evet** Aşırı derece stres 5

2. Covid-19 pandemisi sizin veya bir aile üyenizin ihtiyaç duyduğu sağlık hizmetine başvurmayı ertelemenize / başvurmaktan kaçınmanıza neden oldu mu?

◯ **Hayır** 0

◯ **Evet** Hiç stresli değil 1

◯ **Evet** Biraz stres 2

◯ **Evet** Orta derecede stres 3

◯ **Evet** Oldukça fazla stres 4

◯ **Evet** Aşırı derecede stres 5

3. Covid-19 pandemisi dini faaliyetlere katılımınızı azalttı mı? (yüz yüze veya çevrimiçi/online)

◯ **Bana uygun değil**

◯ **Hayır** 0

◯ **Evet** Hiç stresli değil 1

◯ **Evet** Biraz stres 2

◯ **Evet** Orta derecede stres 3

◯ **Evet** Oldukça fazla stres 4

◯ **Evet** Aşırı derecede stres 5

4. Covid-19 pandemisi nedeniyle rutininizde başka büyük değişiklikler oldu mu?

◯ **Hayır** 0

For peer review only

For peer review only - http://bmjopen.bmj.com/site/about/guidelines.xhtml

○ Evet ise lütfen belirtin1 ____

Evet ise, lütfen bunun aşağıdaki ölçekte sizde neden olduğu stres miktarını belirtin.

○ Hiç stresli değil 1

○ Biraz stres 2

○ Orta derecede stres 3

○ Oldukça fazla stres 4

○ Aşırı derecede stres 5

Covid-19 hakkındaki bilgilere maruz kalma

1. Covid-19 ile ilgili bilgi almak için hangi kaynakları kullanıyorsunuz? (Lütfen uygun olanları işaretleyin.)

☐ Haberler

☐ Facebook / Twitter

☐ Sağlık web siteleri

☐ Duyum/Başkalarından duyma

☐ Diğerleri (lütfen belirtiniz) ____

2. Covid-19 ile ilgili haberler sizde ne kadar strese neden oluyor?

○ Hiç stresli değil 1

○ Biraz stres 2

○ Orta derecede stres 3

○ Oldukça fazla stres 4

○ Aşırı derecede stres 5

For peer review only - http://bmjopen.bmj.com/site/about/guidelines.xhtml

Covid-19'dan bu yana değişiklikler

Aşağıdaki ifadeler için size uygun olanı lütfen ilgili ölçekte belirtiniz. Şimdi ile Covid-19 salgını öncesini karşılaştırma---

1. Covid-19 pandemisi öncesi ile kıyasladığınızda salgın sonrası kişisel stres seviyenizi nasıl değerlendirirsiniz?

- ◯ Önemli ölçüde daha az stresli 1
- ◯ Biraz daha az stresli 2
- ◯ Aynı sayılır 3
- ◯ Daha stresli 4
- ◯ Önemli ölçüde daha fazla stresli 5

2. Covid-19 pandemisi öncesi ile kıyasladığınızda salgın sonrası evinizdeki genel stres düzeyini nasıl değerlendirirsiniz?

- ◯ Önemli ölçüde daha az stresli 1
- ◯ Biraz daha az stresli 2
- ◯ Aynı sayılır 3
- ◯ Daha stresli 4
- ◯ Önemli ölçüde daha fazla stresli 5

3. Covid-19 pandemisi öncesi ile kıyasladığınızda salgın sonrası kişisel psikolojik iyilik halinizi nasıl değerlendirirsiniz?

- ◯ Çok daha iyi 1
- ◯ Daha iyi 2
- ◯ Aynı sayılır 3
- ◯ Daha kötü 4

For peer review only - http://bmjopen.bmj.com/site/about/guidelines.xhtml

◯ Çok daha kötü 5

4. Covid-19 pandemisi öncesi ile kıyasladığınızda salgın sonrası hanehalkı üyelerinizin genel psikolojik iyilik halini nasıl değerlendirirsiniz?

◯ Çok daha iyi 1

◯ Daha iyi 2

◯ Ayni sayilir 3

◯ Daha kötü 4

◯ Çok daha kötü 5

For peer review only

K-10

Aşağıdaki 10 soru son 4 hafta içinde kendinizi nasıl hissettiğinizle ilgilidir. Her soru için, kendinizi o durumda hissettiğiniz zamanın uzunluğunu en iyi şekilde tanımlayan seçeneği seçiniz.

	**Hiçbir zaman 1**	**Kısa bir süre 2**	**Bazen 3**	**Coğu zaman 4**	**Her zaman 5**
1. Son 4 hafta içinde, ne kadar sıklıkla kendinizi sebepsiz yere yorgun hissettiniz?	○	○	○	○	○
2. Son 4 hafta içinde, ne kadar sıklıkla kendinizi gergin hissettiniz?	○	○	○	○	○
3. Son 4 hafta icinde ne kadar sıklıkla sizi hiçbir şeyin sakinleştirmeyeceği kadar gergin hissettiniz?	○	○	○	○	○
4. Son 4 hafta içinde ne kadar sıklıkla kendinizi umutsuz hissettiniz?	○	○	○	○	○
5. Son 4 hafta içinde ne kadar sıklıkla kendinizi tedirgin veya huzursuz hissettiniz?	○	○	○	○	○
6. Son 4 hafta içinde ne kadar sıklıkla yerinde duramayacak kadar huzursuz hissettiniz?	○	○	○	○	○
7. Son 4 hafta içinde ne kadar sıklıkla kendinizi bunalımlı hissettiniz?	○	○	○	○	○
8. Son 4 hafta içinde ne kadar sıklıkla en ufak bir şeyin bile çok çaba gerektirdiğini hissettiniz?	○	○	○	○	○
9. Son 4 hafta içinde ne kadar sıklıkla kendinizi sizi hiçbir şeyin neşelendiremeyeceği kadar üzgün hissettiniz?	○	○	○	○	○
10. Son 4 hafta icinde ne kadar sıklıkla kendinizi değersiz hissettiniz?	○	○	○	○	○

For peer review only

For peer review only - http://bmjopen.bmj.com/site/about/guidelines.xhtml

PCL-5

Aşağıda, bazı kişilerin Covid-19'a tepki olarak yaşadığı sorunların bir listesi bulunmaktadır. Lütfen her bir sorunu dikkatlice okuyun ve geçen ay bu sorundan ne kadar rahatsız olduğunuzu belirtin. Geçen ay içinde aşağıda yer alan durumlar sizi ne ölçüde bunalttı:

	**Hiç 0**	**Çok az 1**	**Orta derecede 2**	**Oldukça az 3**	**Aşırı 4**
1. Stresli olayın tekrarlayan, rahatsız eden ve istenmeyen anları sizi ne kadar bunalttı?	○	○	○	○	○
2. Stresli olaya ilişkin tekrarlayan, rahatsız eden rüyalar sizi ne kadar bunalttı?	○	○	○	○	○
3. Aniden stresli olayı sanki gerçekten bir daha yaşıyormuş gibi hissetmek veya davranmak (sanki gerçekten olayın yaşandığı ana geri dönmüş yeniden yaşıyor gibi) sizi ne kadar bunalttı?	○	○	○	○	○
4. Bir şeyler size stresli olayı anımsattığı zaman yaşadığınız üzüntü hissi sizi ne kadar bunalttı?	○	○	○	○	○
5. Bir şeyler size stresli olayı anımsattığı zaman güçlü fiziksel tepkiler vermek (örneğin, kalp çarpıntısı, nefes almada güçlük, terleme gibi) sizi ne kadar bunalttı?	○	○	○	○	○
6. Stresli olayla ilişkili anılardan,düşüncelerden ve duygulardan kaçınmaya çalışmak sizi ne kadar bunalttı?	○	○	○	○	○
7. Stresli olayı anımsatan etraftaki hatırlatıcı şeylerden (örneğin, insanlardan, yerlerden, konuşmalardan, etkinliklerden, nesnelerden veya durumlardan) kaçınmaya çalışmak sizi ne kadar bunalttı?	○	○	○	○	○
8. Stresli olaya ilişkin önemli kısımları hatırlamada yaşanan güçlükler sizi ne kadar bunalttı?	○	○	○	○	○
9. Kendiniz, diğer insanlar veya dünya hakkında güçlü olumsuz düşüncelere sahip olmak (örneğin, kötü biriyim, bende ciddi şekilde yanlış olan bir şeyler var, kimseye güvenilmez, dünya tümüyle tehlikeli bir yerdir gibi düşünceler) sizi ne kadar bunalttı?	○	○	○	○	○

For peer review only

For peer review only - http://bmjopen.bmj.com/site/about/guidelines.xhtml

10. Stresli olay veya bu olayın sonrasında ortaya çıkan durumlar için kendinizi veya bir başkasını suçlamak sizi ne kadar bunalttı?	○	○	○	○	○
11. Korku, dehşete kapılma, öfke, suçluluk, veya utanç gibi olumsuz duygular sizi ne kadar bunalttı?	○	○	○	○	○
12. Daha önce yapmaktan keyif aldığınız etkinliklere olan ilginizi kaybetmek sizi ne kadar bunalttı?	○	○	○	○	○
13. Başka insanlardan uzak veya kopmuş hissetmek sizi ne kadar bunalttı?	○	○	○	○	○
14. Olumlu duyguları yaşamak (örneğin, mutluluğu hissetmek vaya size yakın insanlara sevgi dolu hisler duyamamak) sizi ne kadar bunalttı?	○	○	○	○	○
15. Asabi davranışlar, öfke patlamaları veya öfkeli hareketler sizi ne kadar bunalttı?	○	○	○	○	○
16. Çok fazla risk almak veya size zarar verebilecek şeyler yapmak sizi ne kadar bunaltı?	○	○	○	○	○
17. Aşırı tetikte olmak veya temkinli davranmak veya hazırda beklemek sizi ne kadar bunalttı?	○	○	○	○	○
18. Yerinden sıçramak veya kolayca irkilmek sizi ne kadar bunalttı?	○	○	○	○	○
19. Dikkati toplamada güçlükler sizi ne kadar bunalttı?	○	○	○	○	○
20. Uykuya dalma veya uykuyu devam ettirme güçlükleri sizi ne kadar bunalttı?	○	○	○	○	○

For peer review only

For peer review only - http://bmjopen.bmj.com/site/about/guidelines.xhtml

WHO-5

Aşağıdaki beş tanımlamadan her biri için, son iki hafta süresince kendinizi nasıl hissettiğinize en yakın olan yanıtı veriniz. Son iki hafta boyunca

	**Her zaman 5**	**Çoğu zaman 4**	**Zamanın yarısından çoğunda 3**	**Zamanın yarısından azında 2**	**Bazen 1**	**Hiçbir zaman 0**
1. Kendimi neşeli ve keyifli hissettim	○	○	○	○	○	○
2. Kendimi sakin ve rahatlamış hissettim.	○	○	○	○	○	○
3. Kendimi aktif ve dinç hissettim	○	○	○	○	○	○
4. Sabahları kendimi taze ve dinlenmiş hissederek uyandım	○	○	○	○	○	○
5. Günlük yaşantım beni ilgilendiren şeylerle dolu	○	○	○	○	○	○

For peer review only

For peer review only - http://bmjopen.bmj.com/site/about/guidelines.xhtml

PTGI

COVID-19 sonucunda yaşamınızda ve düşüncelerinizde meydana gelen değişimleri lütfen aşağıda verilen puanlama ölçütlerine göre 0 ve 5 arasında değerlendiriniz.

	Hiç 0	Çok az 1	Az derecede 2	Orta derecede 3	Oldukça fazla 4	Aşırı derecede 5
1. Yaşamda önem verdiğim şeylerin öncelik sırası değişti.	○	○	○	○	○	○
2. Kendi hayatıma verdiğim değerde büyük bir artış oldu.	○	○	○	○	○	○
3. Yeni ilgi alanları keşfettim.	○	○	○	○	○	○
4. Kendime güven hissinde artış oldu.	○	○	○	○	○	○
5. Manevi konuları daha iyi anlamaya başladım.	○	○	○	○	○	○
6. Başım sıkıştığında insanlara güvenebileceğimi daha iyi anladım.	○	○	○	○	○	○
7. Yaşamım için yeni bir yön belirledim.	○	○	○	○	○	○
8. Kendimi diğer insanlarla çok daha yakın hissetmeye başladım.	○	○	○	○	○	○
9. Duygularımı ifade etmeye daha çok istekliyim.	○	○	○	○	○	○
10. Zorlukları göğüsleyebileceğimi daha iyi anladım.	○	○	○	○	○	○
11. Yaşamımda daha iyi şeyler yapabiliyorum.	○	○	○	○	○	○
12. Her şeyi olduğu gibi, daha çok kabullenebiliyorum.	○	○	○	○	○	○
13. Her günümü daha iyi değerlendirebiliyorum.	○	○	○	○	○	○

For peer review only

For peer review only - http://bmjopen.bmj.com/site/about/guidelines.xhtml

14. Daha önce var olmayan yeni olanaklara kavuştum.	○	○	○	○	○	○
15. Diğer insanlara karşı daha şefkatliyim.	○	○	○	○	○	○
16. İlişkilerime daha çok emek sarf etmeye başladım.	○	○	○	○	○	○
17. Değişmesi gereken şeyleri değiştirebilmek için daha çok çaba harcıyorum.	○	○	○	○	○	○
18. Daha güçlü bir inanca sahibim.	○	○	○	○	○	○
19. Düşündüğümden çok daha güçlü olduğumu keşfettim.	○	○	○	○	○	○
20. İnsanların ne kadar mükemmel olabildiklerine dair çok şey öğrendim.	○	○	○	○	○	○
21. Başkalarına ihtiyaç duyuyor olmayı daha çok kabullendim.	○	○	○	○	○	○

For peer review only

For peer review only - http://bmjopen.bmj.com/site/about/guidelines.xhtml

International Study – Pakistan (Urdu)

Sociodemographic Information

1. جنس

- ◯ مرد
- ◯ عورت
- ◯ دیگر
- ◯ جواب نہیں دینا چاہتے

2. عمر (سالوں میں)

____

3. ازدواجی حیثیت

- ◯ غیر شادی شدہ
- ◯ غیر ازدوجی تعلق
- ◯ شادی شدہ
- ◯ علیحدگی
- ◯ طلاق یافتہ
- ◯ بیوہ یا رنڈوا

4. آپ کا دین کیا ہے؟

- ◯ کوئی دین نہیں
- ◯ اسلام
- ◯ عیسائیت
- ◯ ہندومت
- ◯ بدھ مت

For peer review only - http://bmjopen.bmj.com/site/about/guidelines.xhtml

For peer review only

○ دیگر (براہ مہربانی وضاحت کریں) ________________________________

5. آپ کا نسلی پس منظر کیا ہے؟ (آپ ایک سے زیادہ درج کرسکتے ہیں)

____

6. آپ روزمرہ کی زندگی میں کون سی زبانیں روانی سے بولتے ہیں؟

____

درج ذیل دو سوالات آپ کی انگریزی زبان کی اہلیت کے بارے میں ہیں۔
6a. میں انگریزی اچھی طرح سمجھ سکتا/سکتی ہوں؟*

○ بالکل بھی نہیں1

○ تھوڑی سی2

○ درمیانی حد تک3

○ زیادہ4

○ بہت زیادہ5

6b. میں انگریزی اچھی طرح بول سکتا/سکتی ہوں؟

○ بالکل بھی نہیں1

○ تھوڑی سی 2

○ درمیانی حد تک3

○ زیادہ4

○ بہت زیادہ5

7. آپ کی تعلیم کی اعلی ترین سطح کیا ہے؟

○ کوئی باضابطہ تعلیم نہیں

○ ہائیر سیکنڈری سکول (جیسے ڈپلومہ یا A-level)

○ سرٹیفکیٹ یا ڈپلومہ 3 - سال سے کم کورس

For peer review only - http://bmjopen.bmj.com/site/about/guidelines.xhtml

○ بیچلر ڈگری (3-4 سالہ کورس)

○ پوسٹ گریجویٹ ڈگری (پوسٹ گریجویٹ ڈپلومہ، ماسٹر، ڈاکٹریٹ)

8. موجودہ اسٹڈی یا کام کی حیثیت (براہ مہربانی جو بھی لاگو ہوں ان پر نشان لگائیں)

☐ فل ٹائم طالب علم
☐ پارٹ ٹائم طالب علم
☐ بے روزگار/کام کی تلاش
☐ فل ٹائم ملازمت
☐ پارٹ ٹائم ملازمت
☐ فل ٹائم ذاتی کام
☐ پارٹ ٹائم ذاتی کام
☐ دفتری پیڈ پیرینٹل چھٹی (paid parental leave)
☐ ہاؤس وائف یا سٹیے-ایٹ-ہوم والد (stay-at-home dad) بلا معاوضہ
☐ ریٹائرڈ
☐ دیگر (براہ مہربانی وضاحت کریں) ________________________________

9. پیشہ

____

10. ماہانہ گھریلو آمدنی (مقامی کرنسی میں)

____

10a. اوسط آمدن کے مقابلے میں، آپ اپنے گھر کی آمدنی کی درجہ بندی کیسے کریں گے؟

○ اوسط سے کم

○ اوسط

○ اوسط سے زیادہ

11. کیا آپ کوویڈ-19 کی وجہ سے لاک ڈاؤن میں ہیں؟

○ ہاں

○ نہیں

For peer review only - http://bmjopen.bmj.com/site/about/guidelines.xhtml
For peer review only

12. کیا آپ کو کوویڈ 19-کی ویکسین دی گئی ہے؟

ہاں

نہیں

12a. اگر نہیں، تو کیا آپ مستقبل میں کوویڈ 19-کی ویکسین لینا چاہیں گیں؟

ہاں

نہیں

13. ہم سمجھتے ہیں کہ کچھ لوگوں کو ماضی میں تکلیف دہ واقعات کا سامنا کرنا پڑا ہوا ہو گا جس کی وجہ سے نئی پریشانیوں سے نمٹنے میں آسانی یا دقت پیدا ہو سکتی ہے۔

کوویڈ-19کی وبا سے پہلے، کیا آپ نے کبھی مندرجہ ذیل میں سے کسی واقعہ کا مشاہدہ یا تجربہ کیا ہے: (براہ مہربانی جو بھی لاگو ہوں ان پر نشان لگائیں)

- قدرتی آفات (جیسے سیلاب، زلزلہ، وغیرہ)
- جنگ یا فوجی تنازعات
- 16 سال کی عمر سے پہلے، بچپن کی پریشانیاں (جیسے نظر انداز ہونا (neglect)، غنڈہ گردی کا شکار (bullying) جسمانی یا جنسی تشدد)
- 16 سال کی عمر کے بعد جسمانی یا جنسی تشدد
- سنگین حادثہ / ایکسیڈنٹ
- دیگر – وضاحت کریں (وضاحت کرنا لازمی نہیں)______________________________________
- مندرجہ بالا میں سے کوئی نہیں

For peer review only

For peer review only - http://bmjopen.bmj.com/site/about/guidelines.xhtml

Covid Psychosocial Impacts Scale

ذاتی اثرات

مندرجہ ذیل بیانات کوویڈ-19 کے آپ پر ہونے والے اثرات کے بارے میں ہیں، براہ مہربانی درج ذیل بیانات کے لئے" ہاں / نہیں "میں سے کسی ایک کا انتخاب کریں۔ اگر آپ کا جواب ہاں میں ہے تو، براہ مہربانی نیچے دیے گیے اسکیل پر اس کی وجہ سے ہونے والے دباؤ (stress) کی نشاندہی کریں۔

1. کیا آپ کو کوئی ایسی بیماری لاحق ہے جو آپ کو کوویڈ-19 کی زِد میں لا سکتی ہے؟

- نہیں 0
- **ہاں** کوئی دباؤ نہیں 1
- **ہاں** تھوڑا سا دباؤ 2
- **ہاں** درمیانی حد تک دباؤ 3
- **ہاں** زیادہ دباؤ 4
- **ہاں** بہت زیادہ دباؤ 5

2. کیا آپ کو کسی شخص سے کوویڈ-19 کی زِد میں آنے کا خطرہ لاحق ہوا ہے؟

- نہیں 0
- **ہاں** کوئی دباؤ نہیں 1
- **ہاں** تھوڑا سا دباؤ 2
- **ہاں** درمیانی حد تک دباؤ 3
- **ہاں** زیادہ دباؤ 4
- **ہاں** بہت زیادہ دباؤ 5

3. کیا آپ کو لگتا ہے کہ آپ کو کوویڈ-19 ہوا تھا؟

- نہیں 0
- **ہاں** کوئی دباؤ نہیں 1

For peer review only - http://bmjopen.bmj.com/site/about/guidelines.xhtml

ہاں تھوڑا سا دباؤ 2

ہاں درمیانی حد تک دباؤ 3

ہاں زیادہ دباؤ 4

ہاں بہت زیادہ دباؤ 5

3a. کیا آپ کا کوویڈ-19 کا ٹیسٹ مثبت آیا تھا؟

نہیں 0

ہاں کوئی دباؤ نہیں 1

ہاں تھوڑا سا دباؤ 2

ہاں درمیانی حد تک دباؤ 3

ہاں زیادہ دباؤ 4

ہاں بہت زیادہ دباؤ 5

3b. کیا آپ کو آج کل کوویڈ-19 کی علامات ہیں؟

نہیں 0

ہاں کوئی دباؤ نہیں 1

ہاں تھوڑا سا دباؤ 2

ہاں درمیانی حد تک دباؤ 3

ہاں زیادہ دباؤ 4

ہاں بہت زیادہ دباؤ 5

For peer review only

For peer review only - http://bmjopen.bmj.com/site/about/guidelines.xhtml

خاندان والوں پر اثرات

مندرجہ ذیل بیانات کوویڈ-19 کے آپ کے قریبی خاندان والوں پر ہونے والے اثرات کے بارے میں ہیں، براہ مہربانی درج ذیل بیانات کے لئے "ہاں / نہیں "میں سے کسی ایک کا انتخاب کریں۔ اگر آپ کا جواب ہاں میں ہے تو، براہ مہربانی نیچے دیے گیے اسکیل پر اس کی وجہ سے ہونے والے دباؤ (stress) کی نشاندہی کریں۔

1. کیا آپ کے خاندان کے کسی قریبی فرد کو کوئی ایسی بیماری ہے جو انہیں کوویڈ-19 کی زِد میں لا سکتی ہے؟

- نہیں 0
- ہاں کوئی دباؤ نہیں 1
- ہاں تھوڑا سا دباؤ 2
- ہاں درمیانی حد تک دباؤ 3
- ہاں زیادہ دباؤ 4
- ہاں بہت زیادہ دباؤ 5

2. کیا آپ کو خدشہ ہے کہ آپ کے خاندان کے کسی قریبی فرد کو کسی شخص سے کوویڈ-19کی زِد میں آنے کا خطرہ لاحق ہوا ہے؟

- نہیں 0
- ہاں کوئی دباؤ نہیں 1
- ہاں تھوڑا سا دباؤ 2
- ہاں درمیانی حد تک دباؤ 3
- ہاں زیادہ دباؤ 4
- ہاں بہت زیادہ دباؤ 5

3. کیا آپ کو لگتا ہے کہ خاندان کے کسی قریبی فرد کو کوویڈ-19 ہوا تھا؟

- نہیں 0
- ہاں کوئی دباؤ نہیں 1
- ہاں تھوڑا سا دباؤ 2

For peer review only - http://bmjopen.bmj.com/site/about/guidelines.xhtml

○ **ہاں** درمیانی حد تک دباؤ 3

○ **ہاں** زیادہ دباؤ 4

○ **ہاں** بہت زیادہ دباؤ 5

3a. کیا خاندان کے کسی قریبی فرد کا کوویڈ-19 کا ٹیسٹ مثبت آیا تھا؟

○ **نہیں** 0

○ **ہاں** کوئی دباؤ نہیں 1

○ **ہاں** تھوڑا سا دباؤ 2

○ **ہاں** درمیانی حد تک دباؤ 3

○ **ہاں** زیادہ دباؤ 4

○ **ہاں** بہت زیادہ دباؤ 5

3b. کیا خاندان کے کسی قریبی فرد کو آج کل کوویڈ-19 کی علامات ہیں؟

○ **نہیں** 0

○ **ہاں** کوئی دباؤ نہیں 1

○ **ہاں** تھوڑا سا دباؤ 2

○ **ہاں** درمیانی حد تک دباؤ 3

○ **ہاں** زیادہ دباؤ 4

○ **ہاں** بہت زیادہ دباؤ 5

3c. کیا خاندان کے کسی قریبی فرد کا کوویڈ-19 سے انتقال ہوا ہے؟

○ **نہیں** 0

○ **ہاں** کوئی دباؤ نہیں 1

For peer review only - http://bmjopen.bmj.com/site/about/guidelines.xhtml

ہاں تھوڑا سا دباؤ 2

ہاں درمیانی حد تک دباؤ 3

ہاں زیادہ دباؤ 4

ہاں بہت زیادہ دباؤ 5

4. کیا آپ کو فکر لاحق ہے کہ بیرون ملک مقیم خاندان کے کسی قریبی فرد کو کوویڈ-19 کی زِد میں آنے کا امکان ہے؟

نہیں 0

ہاں کوئی دباؤ نہیں 1

ہاں تھوڑا سا دباؤ 2

ہاں درمیانی حد تک دباؤ 3

ہاں زیادہ دباؤ 4

ہاں بہت زیادہ دباؤ 5

5. کیا بیرون ملک مقیم خاندان کے کسی قریبی فرد کو آپ کوویڈ-19 کی وجہ سے ملنے نہیں جا سکتے یا وہ آپ کوویڈ-19 کی وجہ سے ملنے نہیں آ سکتے ہیں؟

نہیں 0

ہاں کوئی دباؤ نہیں 1

ہاں تھوڑا سا دباؤ 2

ہاں درمیانی حد تک دباؤ 3

ہاں زیادہ دباؤ 4

ہاں بہت زیادہ دباؤ 5

کام

مندرجہ ذیل بیانات کوویڈ-19 کے آپ اور آپ کے گھرانے پر ہونے والے اثرات کے بارے میں ہیں، براہ مہربانی درج ذیل بیانات کے لئے " ہاں / نہیں / قابل اطلاق نہیں "میں سے کسی ایک کا انتخاب کریں۔ اگر آپ کا جواب ہاں میں ہے تو، براہ مہربانی نیچے دیے گیے اسکیل پر اس کی وجہ سے ہونے والے دباؤ (stress) کی نشاندہی کریں۔

For peer review only - http://bmjopen.bmj.com/site/about/guidelines.xhtml

1. کیا آپ یا آپ کے گھر کا کوئی فرد ایک ضروری ورکر کے طور پر شمار کیا گیا ہے (مثلاً ہیلتھ کیئر، قانون نافذ کرنے والے ادارے، ایمرجنسی سروسز، اسینشل/ضروری سامان/خدمات فراہم کرنے والے اداروں میں)؟

- نہیں 0
- ہاں کوئی دباؤ نہیں 1
- ہاں تھوڑا سا دباؤ 2
- ہاں درمیانی حد تک دباؤ 3
- ہاں زیادہ دباؤ 4
- ہاں بہت زیادہ دباؤ 5

2. کیا آپ کے یا آپ کے گھرانے کے کسی فرد کے کام کرنے کے اوقات میں ایک بڑی تبدیلی رونما ہوئی ہے؟

- نہیں 0
- ہاں کوئی دباؤ نہیں 1
- ہاں تھوڑا سا دباؤ 2
- ہاں درمیانی حد تک دباؤ 3
- ہاں زیادہ دباؤ 4
- ہاں بہت زیادہ دباؤ 5

3. کیا آپ کو یا آپ کے گھرانے کے کسی فرد کو گھر سے کام کرنا پڑرہا ہے؟

- نہیں 0
- ہاں کوئی دباؤ نہیں 1
- ہاں تھوڑا سا دباؤ 2
- ہاں درمیانی حد تک دباؤ 3
- ہاں زیادہ دباؤ 4

For peer review only - http://bmjopen.bmj.com/site/about/guidelines.xhtml

○ **ہاں** بہت زیادہ دباؤ 5

4. کیا آپ کے یا آپ کے گھرانے کے کسی فرد کے کام (job-related work) کی نوعیت بدَل گئی ہے؟

○ **نہیں** 0

○ **ہاں** کوئی دباؤ نہیں 1

○ **ہاں** تھوڑا سا دباؤ 2

○ **ہاں** درمیانی حد تک دباؤ 3

○ **ہاں** زیادہ 4

○ **ہاں** بہت زیادہ دباؤ 5

5. کیا آپ کی یا آپ کے گھرانے کے کسی فرد کی ملازمت چھوٹ گئی ہے؟

○ **قابل اطلاق نہیں**

○ **نہیں** 0

○ **ہاں** کوئی دباؤ نہیں 1

○ **ہاں** تھوڑا سا دباؤ 2

○ **ہاں** درمیانی حد تک دباؤ 3

○ **ہاں** زیادہ دباؤ 4

○ **ہاں** بہت زیادہ دباؤ 5

6. کیا ایسا کاروبار جو آپ کی یا آپ کے گھرانے کی ملکیت ہے، یا جس میں آپ یا آپ کے گھر والے کام کرتے ہیں، بقا کے خطرے میں ہے؟

○ **قابل اطلاق نہیں**

○ **نہیں** 0

○ **ہاں** کوئی دباؤ نہیں 1

For peer review only - http://bmjopen.bmj.com/site/about/guidelines.xhtml
For peer review only

○ ہاں تھوڑا سا دباؤ 2

○ ہاں درمیانی حد تک دباؤ 3

○ ہاں زیادہ دباؤ 4

○ ہاں بہت زیادہ دباؤ 5

آمدن

مندرجہ ذیل بیانات کوویڈ-19 کے آپ کے گھرانے پر ہونے والے اثرات کے بارے میں ہیں، براہ مہربانی درج ذیل بیانات کے لئے" ہاں / نہیں " میں سے کسی ایک کا انتخاب کریں۔ اگر آپ کا جواب ہاں میں ہے تو، براہ مہربانی نیچے دیے گیے اسکیل پر اس کی وجہ سے ہونے والے دباؤ (stress) کی نشاندہی کریں۔

1. کیا آپ کے گھرانے کے مالی حالات میں واضح کمی واقع ہوئی ہے؟

○ نہیں 0

○ ہاں کوئی دباؤ نہیں 1

○ ہاں تھوڑا سا دباؤ 2

○ ہاں درمیانی حد تک دباؤ 3

○ ہاں زیادہ دباؤ 4

○ ہاں بہت زیادہ دباؤ 5

2. کیا آپ کے گھرانے کی بلوں کی ادائیگی یا کھانے پینے کے اخراجات کرنے کی صلاحیت میں واضح کمی واقع ہوئی ہے؟

○ نہیں 0

○ ہاں کوئی دباؤ نہیں 1

○ ہاں تھوڑا سا دباؤ 2

○ ہاں درمیانی حد تک دباؤ 3

○ ہاں زیادہ دباؤ 4

○ ہاں بہت زیادہ دباؤ 5

For peer review only

تعلقات

مندرجہ ذیل بیانات کوویڈ-19 کے آپ پر ہونے والے اثرات کے بارے میں ہیں، براہ مہربانی درج ذیل بیانات کے لئے" ہاں / نہیں "میں سے کسی ایک کا انتخاب کریں۔ اگر آپ کا جواب ہاں میں ہے تو، براہ مہربانی نیچے دیے گیے اسکیل پر اس کی وجہ سے ہونے والے دباؤ (stress) کی نشاندہی کریں۔

1. کیا کوویڈ-19 کے نتیجے میں، فیملی کی مشکلات اور باہمی ناچاقی میں اضافہ ہوا ہے؟

- ○ نہیں 0
- ○ ہاں کوئی دباؤ نہیں 1
- ○ ہاں تھوڑا سا دباؤ 2
- ○ ہاں درمیانی حد تک دباؤ 3
- ○ ہاں زیادہ دباؤ 4
- ○ ہاں بہت زیادہ دباؤ 5

2. کیا کوویڈ-19 کے نتیجے میں، دوستوں کے ساتھ آپ کے رابطے (روبرو یا آن لائن) میں کمی واقع ہوئی ہے؟

- ○ نہیں 0
- ○ ہاں کوئی دباؤ نہیں 1
- ○ ہاں تھوڑا سا دباؤ 2
- ○ ہاں درمیانی حد تک دباؤ 3
- ○ ہاں زیادہ دباؤ 4
- ○ ہاں بہت زیادہ دباؤ 5

روزمرہ کے معمولات اور طرز عمل میں تبدیلیاں

مندرجہ ذیل بیانات کوویڈ-19 کے آپ پر ہونے والے اثرات کے بارے میں ہیں، براہ مہربانی درج ذیل بیانات کے لئے" ہاں / نہیں" میں سے کسی ایک کا انتخاب کریں۔ اگر آپ کا جواب ہاں میں ہے تو، براہ مہربانی نیچے دیے گیے اسکیل پر اس کی وجہ سے ہونے والے دباؤ (stress) کی نشاندہی کریں۔

1. کیا کوویڈ-19 کی وجہ سے آپ کے روزمرہ معمولات (مثال کے طور پر، نیند، ورزش، فارغ وقت، خوشگوار سرگرمیوں، یا مشاغل) میں تبدیلی واقع ہوئی ہے؟

For peer review only - http://bmjopen.bmj.com/site/about/guidelines.xhtml

نہیں 0

ہاں کوئی دباؤ نہیں 1

ہاں تھوڑا سا دباؤ 2

ہاں درمیانی حد تک دباؤ 3

ہاں زیادہ دباؤ 4

ہاں بہت زیادہ دباؤ 5

2. کیا کوویڈ-19 کی وجہ سے آپ نے اپنے یا اپنے گھر والوں کی ہیلتھ کیئر (healthcare) کے حصول میں تاخیر کی ہے؟

نہیں 0

ہاں کوئی دباؤ نہیں 1

ہاں تھوڑا سا دباؤ 2

ہاں درمیانی حد تک دباؤ 3

ہاں زیادہ دباؤ 4

ہاں بہت زیادہ دباؤ 5

3. کیا کوویڈ-19 کی وجہ سے آپ کی دینی سرگرمیوں(روبرو یا آن لائن) میں شمولیت میں کمی واقع ہوئی ہے؟

قابل اطلاق نہیں

نہیں 0

ہاں کوئی دباؤ نہیں 1

ہاں تھوڑا سا دباؤ 2

ہاں درمیانی حد تک دباؤ 3

ہاں زیادہ دباؤ 4

For peer review only - http://bmjopen.bmj.com/site/about/guidelines.xhtml

○ ہاں بہت زیادہ دباؤ 5

4. کیا کوویڈ-19 کی وجہ سے معمول میں کوئی اور بڑی تبدیلی واقع ہوئی ہے؟

○ نہیں 0

○ ہاں (براہ مہربانی وضاحت کریں۔) 1 ________________________________

اگر آپ کا جواب ہاں میں ہے تو، براہ مہربانی نیچے دیے گیے اسکیل پر اس کی وجہ سے ہونے والے دباؤ (stress) کی نشاندہی کریں۔

○ کوئی دباؤ نہیں 1

○ تھوڑا سا دباؤ 2

○ درمیانی حد تک دباؤ 3

○ زیادہ دباؤ 4

○ بہت زیادہ دباؤ 5

کوویڈ-19 کے بارے میں معلومات

1. کوویڈ-19 کے حوالے سے، آپ کے لئے معلومات کا ذریعہ کیا ہے؟ (براہ مہربانی جو بھی آپ پر لاگو ہوں انہیں منتخب کریں۔)

- ☐ خبریں
- ☐ فیس بک / ٹویٹر
- ☐ ہیلتھ ویب سائٹس
- ☐ لوگوں کی باتیں
- ☐ دیگر (براہ مہربانی وضاحت کریں) ________________________________

2. کوویڈ-19 سے متعلق خبریں آپ کے لیے کس قدر دباؤ (stress) کا سبب ہیں؟

○ کوئی دباؤ نہیں 1

○ تھوڑا سا دباؤ 2

○ درمیانی حد تک دباؤ 3

○ زیادہ دباؤ 4

○ بہت زیادہ دباؤ 5

For peer review only - http://bmjopen.bmj.com/site/about/guidelines.xhtml

کوویڈ- 19کے بعد ہونے والی تبدیلیاں

براہ کرم کوویڈ-19 کی عالمی وبا کا پہلے سے موازنہ کرتے ہوئے، درج ذیل بیانات پر دیئے گئے پیمانے کی مدد سے نشاندہی کریں۔

1. آپ اپنے ذاتی ذہنی دباؤ (stress) کی درجہ بندی کس طرح کریں گیں؟

- پہلے سے بہت کم دباؤ 1
- پہلے سے کم دباؤ 2
- کوئی تبدیلی نہیں 3
- پہلے سے زیادہ دباؤ 4
- پہلے سے بہت زیادہ دباؤ 5

2. آپ اپنے گھرانے کے ذہنی دباؤ (stress) کی درجہ بندی کس طرح کریں گیں؟

- پہلے سے بہت کم دباؤ 1
- پہلے سے کم دباؤ 2
- کوئی تبدیلی نہیں 3
- پہلے سے زیادہ دباؤ 4
- پہلے سے بہت زیادہ دباؤ 5

3. آپ اپنی نفسیاتی صحت کی درجہ بندی کس طرح کریں گیں؟

- پہلے سے بہت بہتر 1
- پہلے سے بہتر 2
- کوئی تبدیلی نہیں 3
- پہلے سے بدتر 4
- پہلے سے بہت بدتر 5

For peer review only - http://bmjopen.bmj.com/site/about/guidelines.xhtml

4. آپ اپنے گھرانے کی نفسیاتی صحت کی درجہ بندی کس طرح کریں گیں؟

- پہلے سے بہت بہتر 1
- پہلے سے بہتر 2
- کوئی تبدیلی نہیں 3
- پہلے سے بدتر 4
- پہلے سے بہت بدتر 5

For peer review only

For peer review only - http://bmjopen.bmj.com/site/about/guidelines.xhtml

K-10

نیچے دئیے گئے دس سوالات میں آپ سے پوچھا گیا ہے کہ آپ پچھلے **4** ہفتوں سے کیسا محسوس کر رہے ہیں۔ برائے مہربانی ہر سوال کے لئے نشاندہی کریں جو اس بات کی بہترین وضاحت کرتا ہو کہ آپ نے اس کیفیت کو کتنی مرتبہ محسوس کیا ہے۔

	**کبھی نہیں 1**	**بہت کم وقت 2**	**کبھی کبھار 3**	**زیادہ وقت 4**	**ہر وقت 5**
1. پچھلے 4 ہفتوں میں، آپ نے کتنی مرتبہ بغیر کسی معقول وجہ کے تھکاوٹ محسوس کی؟	○	○	○	○	○
2. پچھلے 4 ہفتوں میں، آپ نے کتنی مرتبہ گھبراہٹ محسوس کی؟	○	○	○	○	○
3. پچھلے 4 ہفتوں میں، آپ نے کتنی مرتبہ اتنی گھبراہٹ محسوس کی کہ کوئی بھی چیز آپ کو سکون نہ دے سکی؟	○	○	○	○	○
4. پچھلے 4 ہفتوں میں، آپ نے کتنی مرتبہ نا امُیدی محسوس کی؟	○	○	○	○	○
5. پچھلے 4 ہفتوں میں، آپ نے کتنی مرتبہ بے سکونی یا بے چینی محسوس کی؟	○	○	○	○	○
6. پچھلے 4 ہفتوں میں، آپ نے کتنی مرتبہ اتنی بے چینی محسوس کی کہ آپ ٹک کے نہ بیٹھ سکتے تھے؟	○	○	○	○	○
7. پچھلے 4 ہفتوں میں، آپ نے کتنی مرتبہ افسردگی (ڈپریشن) محسوس کی*	○	○	○	○	○
8. پچھلے 4 ہفتوں میں، آپ نے کتنی مرتبہ محسوس کیا کہ ہر کام کرنے کے لیے کوشش کرنی پڑ رہی ہے؟	○	○	○	○	○
9. پچھلے 4 ہفتوں میں، آپ کتنی مرتبہ اتنے اُداس تھے کہ کوئی بھی چیز آپ کو خوش نہ کر سکی؟	○	○	○	○	○
10. پچھلے 4 ہفتوں میں، آپ نے کتنی مرتبہ خود کو بے وقعت محسوس کیا؟	○	○	○	○	○

For peer review only

PCL-5

نیچے ان مسائل کی فہرست دی گئی ہے جو لوگوں کو کوویڈ-19 کے نتیجے میں پیش آ سکتے ہیں۔ براہ مہربانی ہر مسئلے کو غور سے پڑھیں اور پھر دیے گئے نمبروں میں سے کسی ایک کو چن کر نشاندہی کریں کہ پچھلے مہینے میں آپ کو اس مسئلے نے کتنا پریشان کیا ہے۔

	بالکل بھی نہیں 0	تھوڑا سا 1	درمیانی حد تک 2	زیادہ 3	بہت زیادہ 4
1. پریشان کن تجربے کی بار بار آنے والی، تکلیف دہ، اور ناپسندیدہ یادیں؟					
2. پریشان کن تجربے کے بار بار آنے والے، تکلیف دہ خواب؟					
3. اچانک محسوس ہونا یا ایسا لگنا کہ پریشان کن تجربہ واقعی دوبارہ ہو رہا ہے (جیسے آپ ماضی میں چلے گئے ہوں اور اس سے دوبارہ گزر رہے ہوں)۔					
4. بہت گھبراہٹ محسوس کرنا جب کوئی چیز آپ کو پریشان کن تجربے کی یاد دلائے۔					
5. سخت جسمانی رد عمل محسوس کرنا جب کوئی چیز آپ کو پریشان کن تجربے کی یاد دلائے (مثلاً، دل کا زور زور سے دھڑکنا، سانس لینے میں دشواری، پسینہ چھوٹنا)؟					
6. پریشان کن تجربے سے متعلق یادوں، خیالات، یا احساسات سے گریز کرنا؟					
7. پریشان کن تجربے کی بیرونی یاد دہانیوں سے گریزکرنا (مثلاً لوگ، مقامات، گفتگو، سرگرمیاں، اشیاء، یا حالات)؟					
8. پریشان کن تجربے کے اہم حصوں کو یاد کرنے میں دقت محسوس کرنا؟					
9. اپنے، دوسرے لوگوں، یا دنیا کے بارے میں سخت منفی خیالات آنا (مثلاً، اس طرح کے خیالات جیسے: میں برا/بری ہوں، میرے ساتھ واقعی میں کچھ مسئلہ ہے، کسی پر بھی اعتبار نہیں کیا جاسکتا، پوری دنیا خطرناک ہے)؟					
10. پریشان کن تجربے کے لئے یا اس کے بعد جو کچھ ہوا اس کے لئے اپنے آپ کو یا کسی اور کو مورد الزام ٹھہرانا؟					
11. سخت منفی جذبات کا ہونا جیسے خوف، وحشت، غصہ، احساس جرم، یا شرمندگی ؟					
12. ان سرگرمیوں میں عدم دلچسپی جن سے آپ کبھی لطف اندوز ہوتے تھے؟					
13. دوسرے لوگوں سے دوری محسوس کرنا یا تعلقات منقطع کرنا؟					

For peer review only

For peer review only - http://bmjopen.bmj.com/site/about/guidelines.xhtml

14. مثبت احساسات محسوس کرنے میں دشواری (مثلاً، آپ کا اپنے قریبی لوگوں کے لئے خوشی یا محبت کے جذبات محسوس کرنے سے قاصر ہونا)؟

15. چڑچڑاپن، برہمی، یا جارحانہ سلوک؟

16. بہت زیادہ خطرات مول لینا یا ایسی چیزیں کرنا جو آپ کو نقصان پہنچا سکتی ہیں؟

17. انتہائی الرٹ/چوکنا یا محتاط رہنا؟

18. بہت تیزی یا آسانی سے چونک جانا؟

19. توجہ دینے میں دقت پیش آنا؟

20. سونے میں دشواری یا نیند کے مسائل؟

For peer review only

For peer review only - http://bmjopen.bmj.com/site/about/guidelines.xhtml

WHO-5

براہِ مہربانی ان پانچ بیانات میں سے ہر ایک کے لئے نشاندہی کریں کہ آپ پچھلے دو ہفتوں سے کیسا محسوس کر رہے ہیں۔

	ہر وقت 5	زیادہ تر وقت 4	آدھے سے زیادہ وقت میں 3	آدھے سے کم وقت میں 2	کچھ وقت 1	کسی وقت بھی نہیں 0
1. میں نے خوشی اور زندہ دلی محسوس کی	○	○	○	○	○	○
2. میں نے سکون اور راحت محسوس کیا	○	○	○	○	○	○
3. میں نے فعال اور چست محسوس کیا	○	○	○	○	○	○
4. میں نے سو کر اٹھتے ہوئے تازہ دم اور آرام دہ محسوس کیا	○	○	○	○	○	○
5. میری روزمرہ زندگی ایسی چیزوں سے بھری پڑی ہے جن میں مجھے دلچسپی ہے	○	○	○	○	○	○

For peer review only

PGTI

درج ذیل بیانات میں سے ہر ایک کی دیے گئے اسکیل کی مدد سے نشاندہی کریں کہ کوویڈ 19-کے نتیجے میں آپ کی زندگی میں کس درجہ کی تبدیلی واقع ہوئی ہے۔

	بالکل نہیں 0	بہت تھوڑا 1	تھوڑا 2	درمیانی حد تک 3	زیادہ 4	بہت زیادہ 5
1. زندگی میں کیا اہم ہے اس کے بارے میں میں نے اپنی ترجیحات کو تبدیل کر دیا ہے	○	○	○	○	○	○
2. مجھے اپنی زندگی کی قدروقیمت پہلے سے زیادہ ہے	○	○	○	○	○	○
3. میں نے نئی دلچسپیاں بنا لی ہیں	○	○	○	○	○	○
4. مجھے احساس خود انحصاری پہلے سے زیادہ ہے	○	○	○	○	○	○
5. مجھے روحانی امور کی سمجھ بوجھ پہلے سے بہتر ہے	○	○	○	○	○	○
6. میں پہلے سے بہتر طور پر دیکھ سکتا /سکتی ہوں کہ میں مصیبت کے وقت لوگوں پر بھروسہ کر سکتا / سکتی ہوں	○	○	○	○	○	○
7. میں نے اپنی زندگی کی ایک نئی راہ متعین کر لی ہے	○	○	○	○	○	○
8. مجھے دوسروں کے ساتھ قربت کا احساس پہلے سے زیادہ ہے	○	○	○	○	○	○
9. میں اپنے جذبات کا اظہار کرنے کے لیے پہلے سے زیادہ آمادہ ہوں	○	○	○	○	○	○
10. میں پہلے سے بہتر جانتا / جانتی ہوں کہ میں مشکلات کا مقابلہ کر سکتا / سکتی ہوں	○	○	○	○	○	○
11. میں اپنی زندگی میں پہلے سے بہتر کام سَر انجام دے رہا / رہی ہوں	○	○	○	○	○	○
12. میری چیزوں کا انجام قبول کرنے کی صلاحیت پہلے سے بہتر ہے	○	○	○	○	○	○
13. میں ہر دن کی قدر پہلے سے زیادہ کرتا / کرتی ہوں	○	○	○	○	○	○

For peer review only - http://bmjopen.bmj.com/site/about/guidelines.xhtml

14. نئے مواقع میسر ہیں جو بصورت دیگر میسر نہ ہوتے	○	○	○	○	○	○
15. مجھ میں دوسروں کے لئے شفقت پہلے سے زیادہ ہے	○	○	○	○	○	○
16. میں اپنے تعلقات نبھانے میں پہلے سے زیادہ محنت کرتا / کرتی ہوں	○	○	○	○	○	○
17. میں ان چیزوں کو تبدیل کرنے کی پہلے سے زیادہ کوشش کرتا / کرتی ہوں جن کو تبدیل کرنے کی ضرورت ہے	○	○	○	○	○	○
18. میرا ایمان پہلے سے زیادہ مضبوط ہے	○	○	○	○	○	○
19. میں نے جان لیا ہے کہ میں اپنی سوچ سے ذیادہ مضبوط ہوں	○	○	○	○	○	○
20. میں نے پہلے سے بھی ذیادہ سیکھا ہے کہ لوگ کتنے اچھے ہیں	○	○	○	○	○	○
21. میں دوسروں سے ملنے والی مدد کو قبول کرنے کے لیے پہلے سے زیادہ آمادہ ہوں	○	○	○	○	○	○

For peer review only

For peer review only - http://bmjopen.bmj.com/site/about/guidelines.xhtml

International Study – Iran (Persian)

Sociodemographic Information

۱. جنسیت:

- ◯ مرد
- ◯ زن
- ◯ دیگر
- ◯ ترجیح می‌دهم بیان نکنم

۲. سن (به سال)

____

۳. وضعیت تأهل

- ◯ هرگز ازدواج نکرده ام
- ◯ با کسی در رابطه‌ام(پارتنر دارم)
- ◯ ازدواج کرده‌ام
- ◯ متارکه
- ◯ مطلقه
- ◯ بیوه

۴. دین شما چیست؟

- ◯ بی‌دین
- ◯ اسلام
- ◯ مسیحیت
- ◯ هندو
- ◯ بودایی

For peer review only

○ ادیان یگر (لطفا مشخص فرمایید)____

۵. پیشینه قومی شما چیست؟ (می توانید بیش از یک مورد ذکر کنید)

____

۶. بطور روزمره به چه زبانی صحبت می‌کنید؟

____

دو پرسش زیر بطور خاص درباره مهارت شما در به کارگیری زبان انگلیسی می‌پرسد.

۶الف. من انگلیسی را خوب می‌فهمم

○ کاملا مخالف

○ مخالف

○ نه موافق نه مخالف

○ موافق

○ کاملا موافق

۶ب. من خوب انگلیسی حرف می‌زنم

○ هیچی

○ کمی

○ تا حدودی

○ خیلی

○ خیلی زیاد

۷. بالاترین مدرک تحصیلی‌تان چیست؟

○ فاقد مدرک رسمی (حضور در مدرسه، کلاس انگلیسی برای گویشوران به زبان های دیگر ESOL)

○ دبیرستان (مثلاً مدرک ان سی ای ایNational Certificate of Educational Achievement or NCEA ، دیپلم آی بی International Baccalaureate or IB، مدرک مدارس هر جایی)

For peer review only - http://bmjopen.bmj.com/site/about/guidelines.xhtml

- ◯ مدرک دانشگاهی (مثل، گواهی نامه، دیپلم یا مدرک فنی – دوره های کمتر از 3 سال)
- ◯ مدرک لیسانس(دوره ۳-۴ ساله)
- ◯ مدرک تحصیلات تکمیل (دیپلم تحصیلات تکمیلی، کارشناسی ارشد، دکترا)

---

۸. وضعیت تحصیلی، کاری و شغلی فعلی (لطفاً گزینه های مربوط را علامت بزنید)

- ☐ تحصیل تمام وقت
- ☐ تحصیل پاره وقت
- ☐ بیکار
- ☐ استخدام تمام وقت
- ☐ استخدام پاره وقت
- ☐ بطور تمام وقت خوداشتغالم (کار و کاسبی خودم را دارم)
- ☐ بطور پاره‌وقت خوداشتغالم
- ☐ در مرخصی با حقوق پس از فرزنددار شدن بسر می‌برم
- ☐ والد خانه‌نشینم (سر کار نمی‌روم و بدون حقوقم)
- ☐ بازنشسته‌ام
- ☐ دیگر (لطفا مشخص کنید)____

۹. شغل

____

۱۰. درآمد ماهانه خانوار (به واحد پول محلی)

____

۱۰الف. در مقایسه با متوسط درآمد، شما درآمد خانوار خودتان را چطور ارزیابی می‌کنید؟

- ◯ زیرمتوسط
- ◯ متوسط
- ◯ بالای متوسط

۱۱. آیا شما در حال حاضر در قرنطینه‌اید؟

For peer review only - http://bmjopen.bmj.com/site/about/guidelines.xhtml

○ بلی

○ خیر

۱۲. آیا واکسن کووید-۱۹ زده‌اید؟

○ بلی

○ خیر

۱۲الف. اگر هنوز واکسن نزده‌اید، آیا دوست دارید در آینده واکسن کووید-۱۹ بزنید؟

○ بلی

○ خیر

۱۳. ما می‌دانیم که برخی افراد در گذشته قربانی حوادث آسیب‌زا شده‌اند که ممکن است کنار آمدن با مشکلات جدید را برایشان ساده‌تر یا سخت‌تر کند.

پیش از همه‌گیری کووید-۱۹ هرگز شاهد هیچ‌یک از موارد زیر بوده‌اید؟ یا خودتان هیچ‌کدام را تجربه کرده‌اید:
(لطفاً گزینه‌های انتخابی را تیک بزنید)

- ☐ بلایای طبیعی (مانند سیل، زمین لرزه و از این قبیل موارد)
- ☐ زندگی در یک منطقه جنگی یا در معرض درگیری نظامی
- ☐ بدبیاری دوران کودکی پیش از ۱۶ سالگی(نظیر عدم رسیدگی مناسب، قلدری، آزار جسمی یا جنسی)
- ☐ آزار جسمی یا جنسی پس از ۱۶ سالگی
- ☐ حادثه فیزیکی جدی
- ☐ سایر – لطفاً بگویید چه بوده است (اختیاری) ____
- ☐ هیچکدام از موارد بال

For peer review only

For peer review only - http://bmjopen.bmj.com/site/about/guidelines.xhtml

Covid Psychosocial Impacts Scale

تاثیرات شخصی

لطفا در صورتی که هر یک از موارد زیر در مواجهه با کووید-۱۹ برای شما اتفاق افتاده است به صورت بلی/خیر پاسخ دهید.
اگر پاسخ شما به هر موردی بلی است لطفا میزان استرسی که آن مورد برای شما داشته را بر روی طیف مربوط علامت بزنید.

۱. آیا شما به لحاظ شرایط خاصی دارید که ممکن است شما را دربرابر کووید-۱۹ آسیب‌پذیر سازد؟

- **خیر** ۰
- **بلی** هیچ استرسی ۱
- **بلی** استرس کم ۲
- **بلی** استرس متوسط ۳
- **بلی** استرس زیاد ۴
- **بلی** استرس خیلی زیاد ۵

۲. هیچ‌وقت احساس کرده‌اید که در خطر تماس با اشخاص آلوده به کووید-۱۹ قرار دارید؟

- **خیر** ۰
- **بلی** هیچ استرسی ۱
- **بلی** استرس کم ۲
- **بلی** استرس متوسط ۳
- **بلی** استرس زیاد ۴
- **بلی** استرس خیلی زیاد ۵

۳. فکر می‌کنید به کووید-۱۹ آلوده شده‌اید؟

- **خیر** ۰
- **بلی** هیچ استرسی ۱
- **بلی** استرس کم ۲
- **بلی** استرس متوسط ۳

For peer review only - http://bmjopen.bmj.com/site/about/guidelines.xhtml
For peer review only

- **بلی** استرس زیاد ۴
- **بلی** استرس خیلی زیاد ۵

۳الف. آیا تست کووید-۱۹ مثبت داشته‌اید؟

- **خیر ۰**
- **بلی** هیچ استرسی ۱
- **بلی** استرس کم ۲
- **بلی** استرس متوسط ۳
- **بلی** استرس زیاد ۴
- استرس خیلی زیاد

۳ب. آیا در حال حاضر نشانگان آلودگی به کووید-۱۹ دارید؟

- **خیر ۰**
- **بلی** هیچ استرسی ۱
- **بلی** استرس کم ۲
- **بلی** استرس متوسط ۳
- **بلی** استرس زیاد ۴
- **بلی** استرس خیلی زیاد ۵

تاثیرات خانوادگی

در صورتی که هر یک از موارد زیر برای اعضای خانواده شما در مواجهه با کووید-۱۹ اتفاق افتاده است لطفا با بلی/خیر پاسخ دهید. اگر پاسخ شما به هر موردی بلی است، لطفا میزان استرسی که آن مورد برای شما داشته را بر روی طیف مربوط علامت بزنید.

۱. آیاهیچیک از اعضای درجه یک خانواده شما شرایط خاص دارد که آنان را نسبت به کووید-۱۹ آسیب‌پذیر بسازد؟

- **خیر ۰**
- **بلی** هیچ استرسی ۱

For peer review only - http://bmjopen.bmj.com/site/about/guidelines.xhtml

- بلی استرس کم ۲
- بلی استرس متوسط ۳
- بلی استرس زیاد ۴
- بلی استرس خیلی زیاد ۵

۲. آیا احساس می‌کنید هیچ‌یک از اعضای درجه یک خانواده شما به خاطر کارش در خطر تماس با افراد آلوده به کووید-۱۹ قرار دارد؟

- خیر ۰
- بلی هیچ استرسی ۱
- بلی استرس کم ۲
- بلی استرس متوسط ۳
- بلی استرس زیاد ۴
- بلی استرس خیلی زیاد ۵

۳. آیا فکر می‌کنید هیچ‌یک از اعضای درجه یک خانواده شما به کووید-۱۹ آلوده شده باشد؟

- خیر ۰
- بلی هیچ استرسی ۱
- بلی استرس کم ۲
- بلی استرس متوسط ۳
- بلی استرس زیاد ۴
- بلی استرس خیلی زیاد ۵

۳الف. هیچ‌یک از اعضای درجه یک خانواده شما تست کووید-۱۹ مثبت داشته است؟

- خیر ۰

For peer review only

For peer review only - http://bmjopen.bmj.com/site/about/guidelines.xhtml

- **بلی** هیچ استرسی ۱
- **بلی** استرس کم ۲
- **بلی** استرس متوسط ۳
- **بلی** استرس زیاد ۴
- **بلی** استرس خیلی زیاد ۵

۳ب. هیچیک از اعضای درجه یک خانواده شما در حال حاضر نشانگان آلودگی به کووید-۱۹ دارد؟

- **خیر** ۰
- **بلی** هیچ استرسی ۱
- **بلی** استرس کم ۲
- **بلی** استرس متوسط ۳
- **بلی** استرس زیاد ۴
- **بلی** استرس خیلی زیاد ۵

۳ج. هیچیک از اعضای درجه یک خانواده شما در اثر آلودگی به کووید-۱۹ درگذشته است؟

- **خیر** ۰
- **بلی** هیچ استرسی ۱
- **بلی** استرس کم ۲
- **بلی** استرس متوسط ۳
- **بلی** استرس زیاد ۴
- **بلی** استرس خیلی زیاد ۵

۴. آیا هیچیک از اعضای درجه یک خانواده شما در خارج از کشور هست که نگران باشید در خطر آلودگی به کووید-۱۹ باشد؟

- **خیر** ۰

For peer review only - http://bmjopen.bmj.com/site/about/guidelines.xhtml

- بلی هیچ استرسی ۱
- بلی استرس کم ۲
- بلی استرس متوسط ۳
- بلی استرس زیاد ۴
- بلی استرس خیلی زیاد ۵

۵. آیا هیچ‌یک از اعضای درجه یک خانواده شما در خارج از کشور هست که قادر به دیدن آنان نباشید یا آنان قادر به دیدار شما نباشند؟

- خیر ۰
- بلی هیچ استرسی ۱
- بلی استرس کم ۲
- بلی استرس متوسط ۳
- بلی استرس زیاد ۴
- بلی استرس خیلی زیاد ۵

کار و کووید

در صورتی که هر یک از موارد زیر در مواجهه با کاوید ۱۹ برای شما یا برای هر یک از افراد خانوار شما (یعنی افرادی که با شما زیر یک سقف زندگی می‌کنند) اتفاق افتاده است، لطفا به صورت بلی/خیر/نامربوط پاسخ دهید.
اگر پاسخ شما به هر موردی بلی است لطفا میزان استرسی که آن مورد برای شما داشته را بر روی طیف مربوط علامت بزنید.

۱ .آیا شما یا هیچ‌یک از اعضای خانوارتان در مشاغلی که ضروری (مثلا در بخش خدمات درمانی، انتظامات، خدمات اضطراری، ارائه‌دهنده کالاها یا خدمات حیاتی) محسوب شده شاغل است؟

- خیر ۰
- بلی هیچ استرسی ۱
- بلی استرس کم ۲
- بلی استرس متوسط ۳
- بلی استرس زیاد ۴

For peer review only - http://bmjopen.bmj.com/site/about/guidelines.xhtml

- **بلی** استرس خیلی زیاد ۵

۲. آیا شما یا هیچ‌یک از اعضای خانوارتان تغییر عمده‌ای در ساعات کارش داشته است؟

- **خیر** ۰
- **بلی** هیچ استرسی ۱
- **بلی** استرس کم ۲
- **بلی** استرس متوسط ۳
- **بلی** استرس زیاد ۴
- **بلی** استرس خیلی زیاد ۵

۳. آیا شما یا هیچ‌یک از اعضای خانوارتان مجبور شده است که محل کارش را به خانه تغییر دهد؟

- **خیر** ۰
- **بلی** هیچ استرسی ۱
- **بلی** استرس کم ۲
- **بلی** استرس متوسط ۳
- **بلی** استرس زیاد ۴
- **بلی** استرس خیلی زیاد ۵

۴. آیا شما یا هیچ‌یک از اعضای خانوارتان مجبور شده است که نوع کارش را تغییر بدهد؟

- **خیر** ۰
- **بلی** هیچ استرسی ۱
- **بلی** استرس کم ۲
- **بلی** استرس متوسط ۳
- **بلی** استرس زیاد ۴

For peer review only - http://bmjopen.bmj.com/site/about/guidelines.xhtml
For peer review only

- **بلی** استرس خیلی زیاد ۵

۵. آیا شما یا هیچ‌یک از افراد خانوار شما از کار بی‌کار شده‌اید؟

- **نامربوط**
- **خیر** ۰
- **بلی** هیچ استرسی ۱
- **بلی** استرس کم ۲
- **بلی** استرس متوسط ۳
- **بلی** استرس زیاد ۴
- **بلی** استرس خیلی زیاد ۵

۶. آیا کسب‌وکار شما یا یکی از افراد خانوار شما یا کسب‌وکاری که شما یا یکی از افراد خانوار شما در آن کار می‌کنید در معرض تعطیلی قرار گرفته است؟

- **نامربوط**
- **خیر** ۰
- **بلی** هیچ استرسی ۱
- **بلی** استرس کم ۲
- **بلی** استرس متوسط ۳
- **بلی** استرس زیاد ۴
- **بلی** استرس خیلی زیاد ۵

در آمد

در صورتی که هر یک از موارد زیر برای خانوار شما در مواجهه با کووید-۱۹ اتفاق افتاده است لطفا با بلی/خیر پاسخ بدهید.
اگر پاسخ شما به هر موردی بلی است، لطفا میزان استرسی که آن مورد برای شما داشته را بر روی طیف مربوط علامت بزنید.

۱. خانوار شما از حیث مالی تنگنای عمده‌ای داشته است؟

- **خیر** ۰

For peer review only - http://bmjopen.bmj.com/site/about/guidelines.xhtml

- **بلی** هیچ استرسی ۱
- **بلی** استرس کم ۲
- **بلی** استرس متوسط ۳
- **بلی** استرس زیاد ۴
- **بلی** استرس خیلی زیاد ۵

۲. آیا خانوار در پرداخت قبوض (برق، تلفن و از این قبیل) یا خرید اقلام غذایی بطور جدی دچار مشکل شده است؟

- **خیر ۰**
- **بلی** هیچ استرسی ۱
- **بلی** استرس کم ۲
- **بلی** استرس متوسط ۳
- **بلی** استرس زیاد ۴
- **بلی** استرس خیلی زیاد ۵

روابط

لطفا در صورتی که هر یک از موارد زیر در مواجهه با کووید-۱۹ برای شما اتفاق افتاده است به صورت بلی/خیر پاسخ دهید.
اگر پاسخ شما به هر موردی بلی است لطفا میزان استرسی که آن مورد برای شما داشته را بر روی طیف مربوط علامت بزنید.
در مواجهه با کووید-۱۹

۱. آیا کووید-۱۹ مشکلات و تنش‌ها را در خانواده افزایش داده است؟

- **خیر ۰**
- **بلی** هیچ استرسی ۱
- **بلی** استرس کم ۲
- **بلی** استرس متوسط ۳
- **بلی** استرس زیاد ۴
- **بلی** استرس خیلی زیاد ۵

For peer review only

For peer review only - http://bmjopen.bmj.com/site/about/guidelines.xhtml

در مواجهه با کووید-۱۹

۲. آیا کووید-۱۹ تماس (فیزیکی یا برخط/آنلاین) شما را با دوستان کاهش داده است؟

- ○ **خیر** ۰
- ○ **بلی** هیچ استرسی ۱
- ○ **بلی** استرس کم ۲
- ○ **بلی** استرس متوسط ۳
- ○ **بلی** استرس زیاد ۴
- ○ **بلی** استرس خیلی زیاد ۵

تغییر رفتارها و رویه‌های روزانه

لطفا در صورتی که هر یک از موارد زیر در مواجهه با کووید-۱۹ برای شما اتفاق افتاده است به صورت بلی/خیر پاسخ دهید.
اگر پاسخ شما به هر موردی بلی است لطفا میزان استرسی که آن مورد برای شما داشته را بر روی طیف مربوط علامت بزنید.

در مواجهه با کووید-۱۹

۱. همه‌گیری کووید-۱۹ منجر به تغییراتی در روال عادی زندگی روزمره شما (برای مثال، خواب، ورزش، اوقات فراغت، فعالیت‌های تفریحی یا سرگرمی) گشته است؟

- ○ **خیر** ۰
- ○ **بلی** هیچ استرسی ۱
- ○ **بلی** استرس کم ۲
- ○ **بلی** استرس متوسط ۳
- ○ **بلی** استرس زیاد ۴
- ○ **بلی** استرس خیلی زیاد ۵

در مواجهه با کووید-۱۹

۲. آیا کووید-۱۹ موجب شده است هنگام نیاز به خدمات بهداشتی و درمانی برای خودتان یا هر یک از اعضای خانواده، اقدامی نکنید یا با تاخیر اقدام کنید؟

- ○ **خیر** ۰

For peer review only - http://bmjopen.bmj.com/site/about/guidelines.xhtml

○ **بلی** هیچ استرسی ۱

○ **بلی** استرس کم ۲

○ **بلی** استرس متوسط ۳

○ **بلی** استرس زیاد ۴

○ **بلی** استرس خیلی زیاد ۵

در مواجهه با کووید-۱۹
۳. آیا این شرایط مشارکت شما را در فعالیت‌های دینی (از طریق حضور فیزیکی یا برخط/آنلاین) کاهش داده است؟

○ **نامربوط**

○ **خیر ۰**

○ **بلی** هیچ استرسی ۱

○ **بلی** استرس کم ۲

○ **بلی** استرس متوسط ۳

○ **بلی** استرس زیاد ۴

○ **بلی** استرس خیلی زیاد ۵

در مواجهه با کووید-۱۹
۴. آیا تغییر عمده دیگری در عادات زندگی شما روی داده است؟

○ **خیر ۰**

○ **بلی (لطفا بفرمایید چه تغییر یا تغییراتی)۱** ____

اگر پاسخ شما به هر موردی بلی است لطفا میزان استرسی که آن مورد برای شما داشته را بر روی طیف مربوط علامت بزنید.

○ هیچ استرسی ۱

○ استرس کم ۲

○ استرس متوسط ۳

For peer review only - http://bmjopen.bmj.com/site/about/guidelines.xhtml

- استرس زیاد ۴
- استرس خیلی زیاد ۵

قرار گرفتن در معرض اطلاعات درباره کاوید-۱۹

۱. در رابطه با کووید-۱۹، از کدام منبع اطلاعاتی استفاده می‌کنید؟

- ☐ اخبار
- ☐ فیس‌بوک / توییتر
- ☐ وب‌سایت‌های بهداشتی
- ☐ گپ و گفت با دیگران
- ☐ منابع دیگر (لطفا مشخص فرمایید)____

۲. اخبار کووید-۱۹ چقدر موجب استرس شما می‌شود؟

- هیچ استرسی ۱
- استرس کم ۲
- استرس متوسط ۳
- استرس زیاد ۴
- استرس خیلی زیاد ۵

تغییرات از زمان کووید-۱۹

لطفا برای موارد زیر میزان استرس‌تان را بر روی مقیاس زیر مشخص کنید.

مقایسه حال حاضر با پیش از همه‌گیری کووید-۱۹ ....
۱. سطح استرس خود را چطور ارزیابی می‌کنید؟

- استرس بسیار کمتر ۱
- استرس کمتر ۲
- تقریبا به همان میزان ۳
- استرس بیشتر ۴
- استرس بسیار بیشتر ۵

For peer review only - http://bmjopen.bmj.com/site/about/guidelines.xhtml
For peer review only

مقایسه حال حاضر با پیش از همه‌گیری کووید-۱۹....
۲. بطور کلی سطح استرس در خانوار را چطور ارزیابی می‌کنید؟

- ○ استرس بسیار کمتر ۱
- ○ استرس کمتر ۲
- ○ تقریبا به همان میزان ۳
- ○ استرس بیشتر ۴
- ○ استرس بسیار بیشتر ۵

مقایسه حال حاضر با پیش از همه‌گیری کووید-۱۹.....
۳. سلامت روانی خود را چطور ارزیابی می‌کنید؟

- ○ خیلی بهتر ۱
- ○ بهتر ۲
- ○ تقریبا همانطور که بود ۳
- ○ بدتر ۴
- ○ خیلی بدتر ۵

مقایسه حال حاضر با پیش از همه‌گیری کووید-۱۹...
۴. بطور کلی سلامت روانی اعضای خانوار را چطور ارزیابی می‌کنید؟

- ○ خیلی بهتر ۱
- ○ بهتر ۲
- ○ تقریبا همانطور که بود ۳
- ○ بدتر ۴
- ○ خیلی بدتر ۵

For peer review only

For peer review only - http://bmjopen.bmj.com/site/about/guidelines.xhtml

K-10

در چهار هفته گذشته چه حس و حالی داشته اید؟

	**هیچگاه** ۱	**به ندرت** ۲	**گاهی اوقات** ۳	**بیشتر وقت ها** ۴	**همیشه** ۵
۱. در چهار هفته گذشته چقدر بی‌دلیل احساس خستگی کرده اید؟	○	○	○	○	○
۲. در چهار هفته گذشته چقدر احساس عصبی بودن کرده‌اید؟	○	○	○	○	○
۳. در چهار هفته گذشته هیچگاه شده است آنقدر عصبی باشید که هیچ چیز نتواند شما را آرام کند؟	○	○	○	○	○
۴. در چهار هفته گذشته چقدر احساس نومیدی کرده‌اید؟	○	○	○	○	○
۵. در چهار هفته گذشته چقدر احساس ناآرامی و بی‌قراری کرده‌اید؟	○	○	○	○	○
٦. در چهار هفته گذشته هیچگاه شده است آنقدر احساس ناآرامی کنید که نتوانید بی‌حرکت یک جا بنشینید؟	○	○	○	○	○
٧. در چهار هفته گذشته چقدر احساس افسردگی کرده‌اید؟	○	○	○	○	○
۸. در چهار هفته گذشته چقدر احساس کرده‌اید که همه کارها را دارید به زور انجام می‌دهید؟	○	○	○	○	○
۹. در چهار هفته گذشته چقدر احساس کرده‌اید که چنان غمگینید که هیچ چیز نمی‌تواند شما را سر حال بیاورد؟	○	○	○	○	○
۱۰. در چهار هفته گذشته چقدر احساس بی‌ارزش بودن کرده اید؟	○	○	○	○	○

For peer review only

PCL-5

فهرست زیر حاوی مشکلاتی است که بعضی از افراد در مواجهه با کووید-۱۹ تجربه کرده‌اند .لطفاً هر مشکل را با دقت بخوانید و سپس مشخص کنید که در یک ماه گذشته چقدر از آن مشکل رنجیده‌اید.

درماه گذشته چقدر موارد زیر شما را آزرده است؟

	اصلا ۰	بسیار کم ۱	متوسط ۲	زیاد ۳	بسیار زیاد ۴
۱- خاطرات تکراری، آزاردهنده و ناخواسته از آن تجربه استرس‌زا داشته‌اید؟	○	○	○	○	○
۲- کابوس‌های تکراری و آزاردهنده از آن تجربه استرس‌زا دیده اید؟	○	○	○	○	○
۳- احساس و عمل ناگهانی داشته‌اید به طوری که انگار آن تجربه استرس‌زا دوباره اتفاق افتاده باشد (در حالی که شما انگار واقعا آنجا حضور داشته‌اید و تجربه‌اش کرده‌اید)؟	○	○	○	○	○
۴- وقتی که یک اتفاق، آن تجربه استرس‌زا را در ذهن شما تداعی کرده احساس ناراحتی زیادی داشته‌اید؟	○	○	○	○	○
۵- هر گاه اتفاق یا چیزی، آن تجربه استرس‌زا را در ذهن شما تداعی کرده، واکنش فیزیکی شدیدی (مثل تپش قلب، مشکل تنفس و عرق کردن) داشته‌اید؟	○	○	○	○	○
٦- از خاطرات، افکار و احساسات مرتبط با آن تجربه استرس زا دوری کرده‌اید؟	○	○	○	○	○
٧- از نشانه‌های یادآور آن تجربه استرس‌زا (مثل اشخاص، مکان ها، گفت‌وگوها، فعالیت‌ها، اشیا و یا موقعیت‌ها) دوری کرده‌اید؟	○	○	○	○	○
۸- بخش‌های مهم آن تجربه استرس‌زا را به سختی به یاد می آورید؟	○	○	○	○	○
۹- در مورد خودتان، بقیه مردم و یا جهان، عقاید خیلی منفی (مثل اینکه من بد هستم، یا مشکل جدی دارم، یا به هیچ کس نمی شود اعتماد کرد، یا جهان خیلی خطرناک است) داشته‌اید؟	○	○	○	○	○
۱۰- خود یا دیگری را در آن تجربه استرس‌زا و یا اتفاقات بعد از آن مقصر دانسته‌اید؟	○	○	○	○	○
۱۱- احساس‌های به شدت منفی مانند ترس، وحشت، خشم، گناه و خجالت داشته‌اید؟	○	○	○	○	○
۱۲- علاقه‌تان به فعالیتی که قبلا از آن لذت می‌بردید از بین رفته است؟	○	○	○	○	○
۱۳- احساس فاصله گرفتن و قطع ارتباط با افراد دیگر را داشته اید؟	○	○	○	○	○

For peer review only

۱۳- در تجربه کردن احساسات مثبت و خوب دچار مشکل شده اید؟ مثل ناتوانی در احساس شادی یا احساس دوست داشتن نزدیکان؟

۱۵- بدخلق و تحریک‌پذیر، دارای خشم شدید و رفتاری تهاجمی بوده‌اید؟

۱٦- بیش از حد ریسک‌پذیر بوده و یا کارهایی را که ممکن بود به شما آسیب برساند انجام داده‌اید؟

۱۷- بیش از حد محتاط «یا مراقب و یا گارد گرفته» بوده‌اید؟

۱۸- مضطرب بوده و به راحتی از جا می‌پریدید؟

۱۹- به سختی تمرکز داشته‌اید؟

۲۰- بد خوابیده و خواب خوب و سنگینی نداشته‌اید؟

For peer review only

WHO-5

در دو هفته گذشته چه حس و حالی داشته‌اید؟

گذشته هفته ۲ طی	تمام اوقات ۵	بیشتر اوقات ۴	کمی بیشتر از نیمی از اوقات ۳	کمی کمتر از نیمی از اوقات ۲	بعضی وقت ها ۱	هیچ وقت ۰
۱. شاد و خوش‌خلق بوده‌ام .	○	○	○	○	○	○
۲. احساس آرامش و راحتی داشته‌ام .	○	○	○	○	○	○
۳. احساس می‌کنم فعال و پرانرژی بوده‌ام .	○	○	○	○	○	○
۴. تر و تازه و سرحال بیدار شده‌ام .	○	○	○	○	○	○
۵. زندگی روزمره‌ام مملو از چیزهای مورد علاقه‌ام بوده است .	○	○	○	○	○	○

For peer review only

PTGI

هر یک از عبارات زیر به وقوع تغییری در زندگی شما اشاره دارد .با استفاده از مقیاس ارائه شده نشان دهید که آن تغییر تا چه اندازه در مواجهه با کووید-۱۹ حادث شده است.

	اصلاً هیچ ۰	خیلی کم ۱	تا حدودی ۲	به طور متوسط ۳	تقریبا زیاد ۴	بسیار زیاد ۵
۱. من اولویت‌هایم را درباره اینکه چه چیز در زندگی مهم است تغییر دادم.	○	○	○	○	○	○
۲. من برای زندگی خودم بیشتر ارزش قائلم.	○	○	○	○	○	○
۳. علائق جدیدی پیدا کردم.	○	○	○	○	○	○
۴. بیشتر احساس می‌کنم به خودم متکی ام.	○	○	○	○	○	○
۵. درک بهتری از موضوعات معنوی دارم.	○	○	○	○	○	○
٦. هر چه بیشتر احساس می کنم که می‌توانم در زمان گرفتاری‌ها روی مردم حساب کنم.	○	○	○	○	○	○
۷. مسیر جدیدی برای زندگی‌ام ایجاد کردم.	○	○	○	○	○	○
۸. با دیگران احساس نزدیکی بیشتری می‌کنم.	○	○	○	○	○	○
۹. بیشتر تمایل دارم که ابراز احساسات کنم.	○	○	○	○	○	○
۱۰. به خوبی می‌دانم که می‌توانم از پس سختی‌ها برآیم.	○	○	○	○	○	○
۱۱. می‌توانم کارهای بهتری در زندگی‌ام انجام دهم.	○	○	○	○	○	○
۱۲. بهتر می‌توانم بپذیرم که مسائل چگونه حل می‌شوند.	○	○	○	○	○	○
۱۳. بهتر می توانم قدر هر روز را بدانم.	○	○	○	○	○	○

For peer review only

For peer review only - http://bmjopen.bmj.com/site/about/guidelines.xhtml

۱۴. فرصت‌های جدیدی پدید آمده‌اند که در غیر این صورت هرگز پیش نمی آمدند.	○	○	○	○	○	○
۱۵. حس دلسوزی بیشتری نسبت به دیگران دارم.	○	○	○	○	○	○
۱۶. برای [حفظ] روابطم بیشتر تلاش می‌کنم.	○	○	○	○	○	○
۱٧. بیشتر احتمال دارد تلاش کنم چیزهایی که باید عوض شوند را عوض کنم.	○	○	○	○	○	○
۱۸. من ایمان مذهبی قوی‌تری دارم.	○	○	○	○	○	○
۱۹. کشف کردم از آنچه که فکرش را می‌کردم قوی‌ترم.	○	○	○	○	○	○
۲۰. کلی درباره اینکه.	○	○	○	○	○	○
۲۱. من راحت تر می‌پذیرم که به دیگران نیاز دارم.	○	○	○	○	○	○

For peer review only

For peer review only - http://bmjopen.bmj.com/site/about/guidelines.xhtml

STROBE Statement—checklist of items that should be included in reports of observational studies

	Item No	Recommendation	Page No
**Title and abstract**	1	(*a*) Indicate the study's design with a commonly used term in the title or the abstract	1,2
		(*b*) Provide in the abstract an informative and balanced summary of what was done and what was found	2
**Introduction**			
Background/rationale	2	Explain the scientific background and rationale for the investigation being reported	7
Objectives	3	State specific objectives, including any prespecified hypotheses	8
**Methods**			
Study design	4	Present key elements of study design early in the paper	9
Setting	5	Describe the setting, locations, and relevant dates, including periods of recruitment, exposure, follow-up, and data collection	10
Participants	6	(*a*) *Cohort study*—Give the eligibility criteria, and the sources and methods of selection of participants. Describe methods of follow-up<br>*Case-control study*—Give the eligibility criteria, and the sources and methods of case ascertainment and control selection. Give the rationale for the choice of cases and controls<br>*Cross-sectional study*—Give the eligibility criteria, and the sources and methods of selection of participants	9-11
		(*b*) *Cohort study*—For matched studies, give matching criteria and number of exposed and unexposed<br>*Case-control study*—For matched studies, give matching criteria and the number of controls per case	
Variables	7	Clearly define all outcomes, exposures, predictors, potential confounders, and effect modifiers. Give diagnostic criteria, if applicable	12
Data sources/ measurement	8*	For each variable of interest, give sources of data and details of methods of assessment (measurement). Describe comparability of assessment methods if there is more than one group	13
Bias	9	Describe any efforts to address potential sources of bias	15
Study size	10	Explain how the study size was arrived at	12
Quantitative variables	11	Explain how quantitative variables were handled in the analyses. If applicable, describe which groupings were chosen and why	12,13
Statistical methods	12	(*a*) Describe all statistical methods, including those used to control for confounding	12,13
		(*b*) Describe any methods used to examine subgroups and interactions	13
		(*c*) Explain how missing data were addressed	12
		(*d*) *Cohort study*—If applicable, explain how loss to follow-up was addressed<br>*Case-control study*—If applicable, explain how matching of cases and controls was addressed<br>*Cross-sectional study*—If applicable, describe analytical methods taking account of sampling strategy	12
		(*e*) Describe any sensitivity analyses	13

Continued on next page

For peer review only - http://bmjopen.bmj.com/site/about/guidelines.xhtml

**Results**			
Participants	13*	(a) Report numbers of individuals at each stage of study—eg numbers potentially eligible, examined for eligibility, confirmed eligible, included in the study, completing follow-up, and analysed	Not applicable
		(b) Give reasons for non-participation at each stage	Not applicable
		(c) Consider use of a flow diagram	Not applicable
Descriptive data	14*	(a) Give characteristics of study participants (eg demographic, clinical, social) and information on exposures and potential confounders	Not applicable
		(b) Indicate number of participants with missing data for each variable of interest	Not applicable
		(c) *Cohort study*—Summarise follow-up time (eg, average and total amount)	Not applicable
Outcome data	15*	*Cohort study*—Report numbers of outcome events or summary measures over time	Not applicable
		Case-control study—Report numbers in each exposure category, or summary measures of exposure	Not applicable
		Cross-sectional study—Report numbers of outcome events or summary measures	Not applicable
Main results	16	(*a*) Give unadjusted estimates and, if applicable, confounder-adjusted estimates and their precision (eg, 95% confidence interval). Make clear which confounders were adjusted for and why they were included	Not applicable
		(*b*) Report category boundaries when continuous variables were categorized	Not applicable
		(*c*) If relevant, consider translating estimates of relative risk into absolute risk for a meaningful time period	Not applicable
Other analyses	17	Report other analyses done—eg analyses of subgroups and interactions, and sensitivity analyses	Not applicable
**Discussion**			
Key results	18	Summarise key results with reference to study objectives	13
Limitations	19	Discuss limitations of the study, taking into account sources of potential bias or imprecision. Discuss both direction and magnitude of any potential bias	14.15
Interpretation	20	Give a cautious overall interpretation of results considering objectives, limitations, multiplicity of analyses, results from similar studies, and other relevant evidence	13,14
Generalisability	21	Discuss the generalisability (external validity) of the study results	15
**Other information**			
Funding	22	Give the source of funding and the role of the funders for the present study and, if applicable, for the original study on which the present article is based	17

*Give information separately for cases and controls in case-control studies and, if applicable, for exposed and unexposed groups in cohort and cross-sectional studies.

Note: An Explanation and Elaboration article discusses each checklist item and gives methodological background and published examples of transparent reporting. The STROBE checklist is best used in conjunction with this article (freely available on the Web sites of PLoS Medicine at http://www.plosmedicine.org/, Annals of Internal Medicine at

For peer review only

http://www.annals.org/, and Epidemiology at http://www.epidem.com/). Information on the STROBE Initiative is available at www.strobe-statement.org.

For peer review only

For peer review only - http://bmjopen.bmj.com/site/about/guidelines.xhtml
